# Supplementary material for: Photoredox cobalt-catalyzed regio-, diastereo- and enantioselective propargylation of aldehydes via propargyl radicals
Source: Nat Commun. 2023 Aug 10;14:4825. doi: 10.1038/s41467-023-40488-3 (PMC10415309; doi:10.1038/s41467-023-40488-3)
Supplement: Supplementary file 1 — Supplementary Information [file 41467_2023_40488_MOESM1_ESM.pdf]

Photoredox Cobalt-Catalyzed Regio-, Diastereo- and Enantioselective Propargylation of Aldehydes via Propargyl Radicals

Lei Wang,[1] Chuiyi Lin,[1] Qinglei Chong,[1]* Zhihan Zhang[2],* and Fanke Meng[1,3,4,]*

[1]State Key Laboratory of Organometallic Chemistry, Center for Excellence in Molecular Synthesis, Shanghai Institute of Organic Chemistry, University of Chinese Academy of Sciences, 345 Lingling Road, Shanghai, 200032, China

[2]CCNU-uOttawa Joint Research Center, Key Laboratory of Pesticide & Chemical Biology, Ministry of Education, College of Chemistry, Central China Normal University, 152 Louyu Road, Wuhan, Hubei, 430079, China

[3]State Key Laboratory of Elemento-Organic Chemistry, Nankai University

[4]School of Chemistry and Materials Science, Hangzhou Institute for Advanced Study, University of Chinese Academy of Sciences, 1 Sub-lane Xiangshan, Hangzhou, 310024, China

*Correspondence: chongql@sioc.ac.cn (Q. C.), zhihanzhang@ccnu.edu.cn (Z. Z.), mengf@sioc.ac.cn (F. M.)

Supplementary Information

Table of Contents

General Information……………………………………………………………...S3

Reagents and Staring Substrates ………………………………………………...S4- S5

Characterization of Unknown Substrates……………………………………... S5–S10

Representative Experimental Procedure for Synthesis of **3a**………..……........….S10-S11

Additional Optimization of Reaction Conditions………...………...............….S11-S13

Characterization of Product………………………………….………………...S13–S56

Experimental Procedure of Gram-Scale Synthesis of **3a**………..…...……..….S56-S57

Experimental Procedure and Characterization of Functionalization……..........S57–S63

Experimental Procedure and Characterization for Mechanistic Studies...……...S63-S69

Computational Details……………………….……………..........................S69–S99

Proof of Stereochemistry: X-ray Characterization Data…………………….S99- S108

NMR Spectra………………..………………………………......................S109-S221

Supplementary Reference……………………………………………..S221–S222

■ General Information

Infrared (IR) spectra were recorded on a BRUKER TENSOR 27 FT-IR spectrometer, λ_{max} in cm^{-1}. Bands are characterized as broad (br), strong (s), medium (m), and weak (w). ^1H NMR spectra were recorded on a Bruker 400 MHz, Agilent 400 MHz or Varian 400 MHz spectrometers. Chemical shifts are reported in ppm with the solvent resonance as the internal standard ($CDCl_3$: δ 7.26 ppm), or tetramethylsilane as internal standard (TMS: δ 0.00 ppm). Data are reported as follows: chemical shift, integration, multiplicity (s = singlet, d = doublet, t = triplet, q = quartet, m = multiplet), and coupling constant (Hz). ^{13}C NMR spectra were recorded on a Bruker 100 MHz Agilent 100 MHz or Varian 100 MHz spectrometers with complete proton decoupling. Chemical shifts are reported in ppm from tetramethylsilane with the solvent resonance as the internal standard ($CDCl_3$: δ 77.00 pm). EI-HRMS and ESI-HRMS spectra were obtained on a Waters Premier GC-TOF MS and a Thermo Scientific Q Exactive HF Orbitrap-FTMS, respectively. FI-HRMS and DART-HRMS spectra were obtained on a JEOL-AccuTOF-GCv4G-GCT MS and a Thermo Fisher Scientific LTQ FTICR-MS, respectively. Enantiomer ratios were determined by high-performance liquid chromatography (HPLC) or supercritical fluid chromatography (SFC) from Shimadzu Corporation. (Chiralpak IA (4.6 x 250 mm), Chiralpak IB (4.6 x 250 mm), Chiralpak IC (4.6 x 250 mm), Chiralpak ID (4.6 x 250 mm), Chiralpak IE (4.6 x 250 mm), Chiralpak IF (4.6 x 250 mm), Chiralpak IG (4.6 x 250 mm), Chiralpak IJ-3 (4.6 x 250 mm), Chiralcel OJ-H (4.6 x 250 mm), Chiralcel OZ-H (4.6 x 250 mm), Chiralcel OD-H (4.6 x 250 mm), Chiralpak AZ-H (4.6 x 250 mm), Chiralpak AD-H (4.6 x 250 mm)) in comparison with authentic racemic materials. Specific rotations were measured on a Rudolph Research Analytical Autopol VI Polarimeter and Autopol I Polarimeter. Melting points were measured on a RY-I apparatus and uncorrected. Unless otherwise noted, all reactions were carried out with distilled and degassed solvents under an atmosphere of dry N_2 in oven- (135 °C) or flame-dried glassware with standard dry box or vacuum-line techniques. Anhydrous THF (J&K Chemicals Inc. and used as received.) were used without further purification. All work-up and purification procedures were carried out with reagent grade solvents (purchased from Adamas Reagent, Ltd.) in air.

■ Reagents and Staring substrates

Cobalt salts, ligands: purchased from Strem Chemicals Inc. and used as received.

4CzIPN, Hantzsch's ester, (*i*Pr)$_2$NEt: purchased from J&K Chemicals Inc. and used as received.

Aldehydes: purchased from TCI Chemicals Inc. and purified by flash column chromatography before used.

Propargylic carbonates:

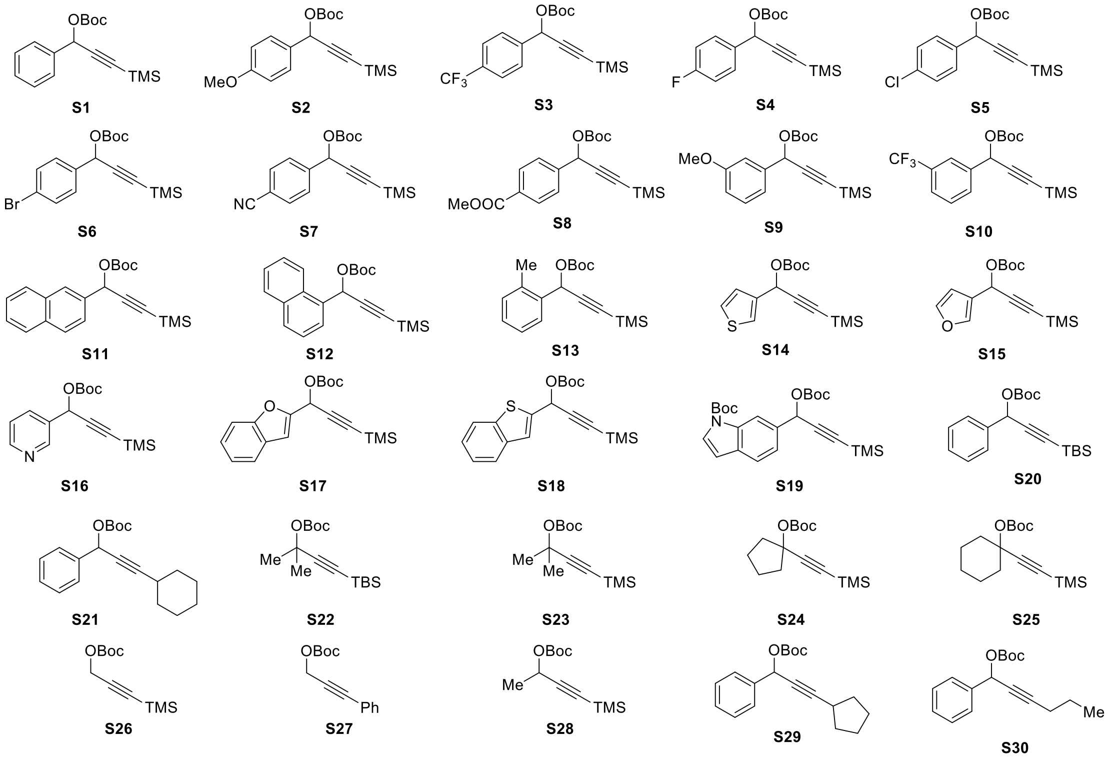

General procedure for synthesis of Propargylic carbonates: prepared according to a previous reported procedure[1]

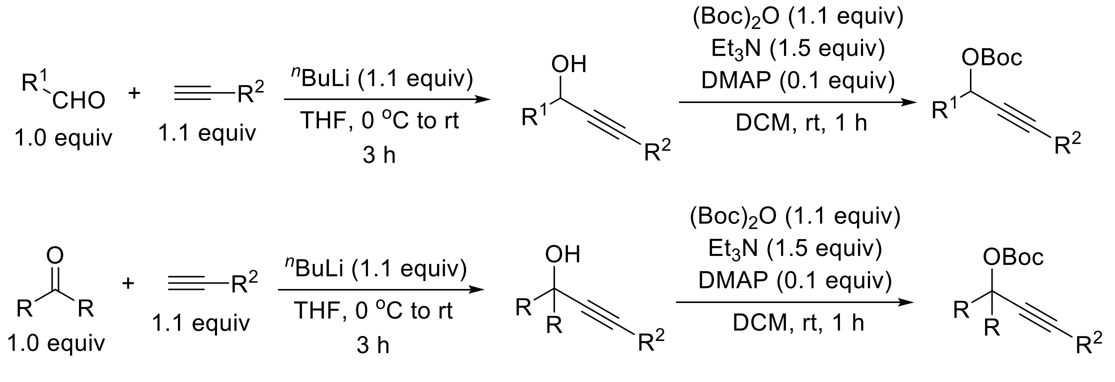

nBuLi (2.5 M in hexane, 4.4 mL, 1.1 equiv) was added dropwise to a solution of alkyne (11 mmol, 1.1 equiv) in anhydrous THF (20 mL) at 0 °C under N_2. After stirring at 0 °C for 1 h, aldehyde or ketone (10 mmol, 1.0 equiv) was added to the mixture. The reaction mixture was allowed to warm to room temperature and stirred for 3 h. The reaction mixture was quenched with H_2O (15 mL) and the resulting aqueous phase was extracted with EA (3×30 mL). The combined organic phase was dried over Na_2SO_4.

After removal of the solvent, the desired propargylic alcohol was obtained as a crude reaction mixture and the was used next step without further purification.

A 50-ml round-bottomed flask equipped with a magnetic stir bar was charged with the crude propargylic alcohol (10 mmol, 1.0 equiv), DMAP (122 mg, 1 mmol, 0.1 equiv), Et_3N (1.518 g, 15 mmol, 1.5 equiv) and DCM (15 mL). $(Boc)_2O$ (2.400 g, 11 mmol, 1.1 equiv) was added dropwise to the mixture and the resulting reaction mixture was stirred for 1 h at room temperature. After removal of solvent, the resulting crude mixture was purified by silica gel column chromatography to afford the pure product.

■ Characterization of Unknown Substrates

tert-butyl (1-phenyl-3-(trimethylsilyl)prop-2-yn-1-yl) carbonate (S1)

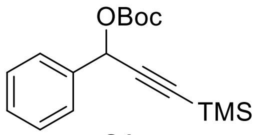

S1

Colorless oil. **IR (neat):** 2969 (w), 2187 (w), 1742 (s), 1319 (s), 1250 (s), 1159 (s), 843 (s), 759 (m), 697 (m) cm^{-1}; **^{1}H NMR (400 MHz, $CDCl_3$):** δ 7.54 (d, *J* = 5.6 Hz, 2H), 7.41 – 7.30 (m, 3H), 6.26 (s, 1H), 1.47 (s, 9H), 0.19 (s, 9H); **^{13}C NMR (100 MHz, $CDCl_3$):** δ 152.4, 136.6, 128.9, 128.5, 127.8, 101.0, 92.9, 82.8, 68.8, 27.6, -0.4; **HRMS (ESI^+) $[M+Na]^+$** Calcd for $C_{17}H_{24}O_3NaSi$: 327.1387 m/z, Found: 327.1381 m/z.

tert-butyl (1-(4-methoxyphenyl)-3-(trimethylsilyl)prop-2-yn-1-yl) carbonate (S2)

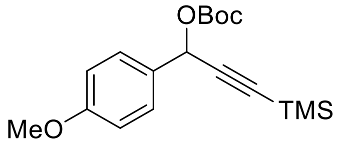

S2

Colorless oil. **IR (neat):** 2969 (w), 2181 (w), 1733 (s), 1370 (m), 1249 (s), 1160 (s), 835 (s), 765 (m) cm^{-1}; **^{1}H NMR (400 MHz, $CDCl_3$):** δ 7.48 (d, *J* = 8.8 Hz, 2H), 6.89 (d, *J* = 8.4 Hz, 2H), 6.20 (s, 1H), 3.81 (s, 3H), 1.48 (s, 9H), 0.19 (s, 9H); **^{13}C NMR (100 MHz, $CDCl_3$):** δ 160.1, 152.5, 129.5, 128.9, 113.9, 101.2, 92.7, 82.8, 68.6, 55.2, 27.7, -0.3; **HRMS (ESI^+) $[M+Na]^+$** Calcd for $C_{18}H_{26}O_4NaSi$: 357.1493 m/z, Found: 357.1489 m/z.

tert-butyl (1-(4-(trifluoromethyl)phenyl)-3-(trimethylsilyl)prop-2-yn-1-yl) carbonate (S3)

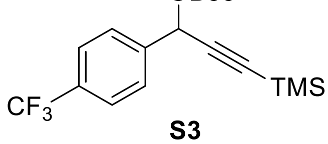

S3

Colorless oil. **IR (neat):** 2985 (s), 2902(m), 1784 (m), 1332 (m), 1106 (s), 1066 (s), 839 (m), 765 (s) cm^{-1}; **^{1}H NMR (400 MHz, $CDCl_3$):** δ 8.00 – 7.31 (m, 4H), 6.29 (s, 1H), 1.49 (s, 9H), 0.19 (s, 9H); **^{13}C NMR (100 MHz, $CDCl_3$):** δ 152.2, 140.6, 131.0 (q, *J* = 32.0 Hz), 128.0, 125.6 (q, *J* = 3.8 Hz), 123.9 (q, *J* = 271 Hz), 100.1, 93.9, 83.4, 68.0, 27.6, -0.4; **^{19}F NMR (376 MHz, $CDCl_3$):** δ -63.6; **HRMS (ESI^+) $[M+Na]^+$** Calcd for $C_{18}H_{23}O_3F_3NaSi$: 395.1261 m/z, Found: 395.1260 m/z.

tert-butyl (1-(4-fluorophenyl)-3-(trimethylsilyl)prop-2-yn-1-yl) carbonate (S4)

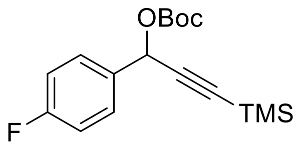

S4

Colorless oil. **IR (neat):** 3672(m), 2985 (s), 2902 (s), 1745 (m), 1394 (m), 1250 (s), 1154 (m), 1069 (s), 836 (m) cm^{-1}; **^{1}H NMR (400 MHz, $CDCl_3$):** δ 7.52 (dd, *J* = 8.8, 5.6 Hz, 2H), 7.05 (t, *J* = 8.8 Hz, 2H), 6.22 (s, 1H), 1.47 (s, 9H), 0.19 (s, 9H); **^{13}C NMR (100 MHz, $CDCl_3$):** δ 163.0 (d, *J* = 248 Hz), 152.3, 132.6 (d, *J* = 3.0 Hz), 129.8 (d, *J* = 8.5 Hz), 115.5 (d, *J* = 21.7 Hz), 100.7, 93.2, 83.0, 68.1, 27.7, -0.4; **^{19}F NMR (376 MHz, $CDCl_3$):** δ -113.5;

HRMS (ESI$^+$) [M+Na]$^+$ Calcd for $C_{17}H_{23}O_3FNaSi$: 345.1293 m/z, Found: 345.1293 m/z.

***tert*-butyl (1-(4-chlorophenyl)-3-(trimethylsilyl)prop-2-yn-1-yl) carbonate (S5)**

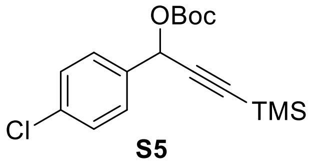

S5

Colorless oil. **IR (neat):** 3673 (m), 2972 (s), 2902 (s), 1744 (s), 1322 (m), 1249 (s), 1158 (m), 1070 (s), 788 (s) cm^{-1};**^{1}H NMR (400 MHz, CDCl$_3$):** δ 7.47 (d, *J* = 8.4 Hz, 2H), 7.34 (d, *J* = 8.4 Hz, 2H), 6.21 (s, 1H), 1.47 (s, 9H), 0.18 (s, 9H); **^{13}C NMR (100 MHz, CDCl$_3$):** δ 152.2, 135.3, 134.9, 129.2, 128.8, 100.5, 93.4, 83.1, 68.0, 27.7, -0.4; **HRMS (ESI$^+$) [M+Na]$^+$** Calcd for $C_{17}H_{23}O_3NaSiCl$: 361.0997 m/z, Found: 361.0998 m/z.

1-(4-bromophenyl)-3-(trimethylsilyl)prop-2-yn-1-yl *tert*-butyl carbonate (S6)

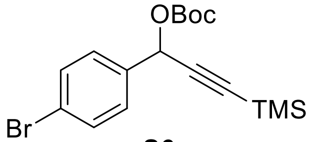

S6

Colorless oil. **IR (neat):** 2980 (w), 1742 (s), 1251 (s), 1153 (m), 1078 (m), 843 (m) cm^{-1}; **^{1}H NMR (400 MHz, CDCl$_3$):** δ 7.50 (d, *J* = 8.4 Hz, 2H), 7.41 (d, *J* = 8.8 Hz, 2H), 6.19 (s, 1H), 1.47 (s, 9H), 0.18 (s, 9H); **^{13}C NMR (100 MHz, CDCl$_3$):** δ 152.2, 135.8, 131.7, 129.5, 123.1, 100.4, 93.4, 83.1, 68.1, 27.6, -0.4; **HRMS (ESI$^+$) [M+Na]$^+$** Calcd for $C_{17}H_{23}O_3NaSiBr$: 405.0492 m/z, Found: 405.0494 m/z.

***tert*-butyl (1-(4-cyanophenyl)-3-(trimethylsilyl)prop-2-yn-1-yl) carbonate (S7)**

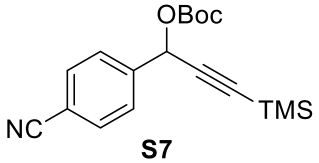

S7

Colorless oil. **IR (neat):** 2973 (w), 1741 (m), 1370 (w), 1151 (m), 1074 (m), 841 (s) cm^{-1}; **^{1}H NMR (400 MHz, CDCl$_3$):** δ 7.6 (q, *J* = 8.4 Hz, 4H), 6.24 (s, 1H), 1.46 (s, 9H), 0.16 (s, 9H); **^{13}C NMR (100 MHz, CDCl$_3$):** δ 152.0, 141.6, 132.4, 128.2, 118.3, 112.7, 99.6, 94.2, 83.5, 67.7, 27.6, -0.5; **HRMS (ESI$^+$) [M+Na]$^+$** Calcd for $C_{18}H_{23}NO_3NaSi$: 352.1339 m/z, Found: 352.1337 m/z.

Methyl 4-(1-((*tert*-butoxycarbonyl)oxy)-3-(trimethylsilyl)prop-2-yn-1-yl)benzoate (S8)

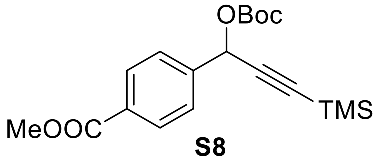

S8

Colorless oil. **IR (neat):** 3046 (w), 1719 (m), 1251 (s), 1157 (m), 845 (s) cm^{-1}; **^{1}H NMR (400 MHz, CDCl$_3$):** δ 8.03 (d, *J* = 8.4 Hz, 2H), 7.58 (d, *J* = 8.0 Hz, 2H), 6.26 (s, 1H), 3.89 (s, 3H), 1.46 (s, 9H), 0.16 (s, 9H); **^{13}C NMR (100 MHz, CDCl$_3$):** δ 166.5, 152.2, 141.4, 130.5, 129.8, 127.5, 100.3, 93.6, 83.2, 68.1, 52.1, 27.6, -0.4; **HRMS (ESI$^+$) [M+Na]$^+$** Calcd for $C_{19}H_{26}O_5NaSi$: 385.1442 m/z, Found: 385.1432 m/z.

***tert*-butyl (1-(3-methoxyphenyl)-3-(trimethylsilyl)prop-2-yn-1-yl) carbonate (S9)**

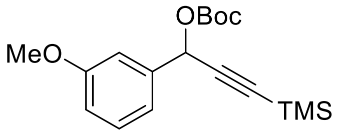

S9

Colorless oil. **IR (neat):** 2961 (w), 1783 (s), 1252 (s), 1151 (m), 1156 (s), 1033 (m), 840 (s) cm^{-1}; **^{1}H NMR (400 MHz, CDCl$_3$):** δ 7.39 (t, *J* = 8.0 Hz, 1H), 7.22 (d, *J* = 6.4 Hz, 2H), 7.00 (dd, *J* = 7.6, 2.0 Hz, 1H), 6.34 (s, 1H), 3.91 (s, 3H), 1.60 (s, 9H), 0.30 (s, 9H); **^{13}C NMR (100 MHz, CDCl$_3$):** δ 159.6, 152.4, 138.0, 129.5, 120.1, 114.8, 112.9, 100.9, 92.9, 82.9, 68.6, 55.1, 27.6, -0.4; **HRMS (ESI$^+$) [M+Na]$^+$** Calcd for $C_{18}H_{26}O_4NaSi$: 357.1493 m/z, Found: 357.1494 m/z.

tert-butyl (1-(3-(trifluoromethyl)phenyl)-3-(trimethylsilyl)prop-2-yn-1-yl) carbonate (S10)

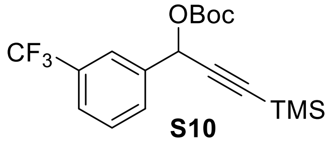

S10

Colorless oil. **IR (neat):** 2938 (w), 1748 (s), 1254 (s), 1164 (s), 1121 (s), 844 (s) cm^{-1}; **^{1}H NMR (400 MHz, $CDCl_3$):** δ 7.83 (s, 1H), 7.72 (d, *J* = 7.6 Hz, 1H), 7.61 (d, *J* = 7.6 Hz, 1H), 7.50 (t, *J* = 8.0 Hz, 1H), 6.29 (s, 1H), 1.49 (s, 9H), 0.20 (s, 9H); **^{13}C NMR (100 MHz, $CDCl_3$):** δ 152.2, 137.7, 131.1, 131.0 (q, *J* = 31.7 Hz), 129.1, 125.8 (q, *J* = 3.8 Hz), 124.7 (q, *J* = 3.9 Hz), 123.8 (q, *J* = 271 Hz), 100.2, 94.0, 83.4, 68.0, 27.6, -0.4; **^{19}F NMR (376 MHz, $CDCl_3$):** δ -63.7; **HRMS (ESI^+) $[M+Na]^+$** Calcd for $C_{18}H_{23}O_3F_3NaSi$: 395.1261 m/z, Found: 395.1261 m/z.

tert-butyl (1-(naphthalen-2-yl)-3-(trimethylsilyl)prop-2-yn-1-yl) carbonate (S11)

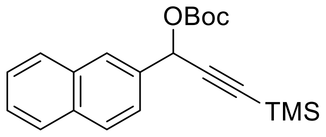

S11

White solid, m.p. 105 – 106 °C; **IR (neat):** 2966 (w), 1736 (s), 1272 (m), 1252 (m), 840 (s), 760 (m) cm^{-1}; **^{1}H NMR (400 MHz, $CDCl_3$):** δ 8.03 (s, 1H), 7.93 – 7.83 (m, 3H), 7.69 (d, *J* = 8.4 Hz, 1H), 7.60 – 7.44 (m, 2H), 6.46 (s, 1H), 1.51 (s, 9H), 0.24 (s, 9H); **^{13}C NMR (100 MHz, $CDCl_3$):** δ 152.4, 134.1, 133.4, 132.9, 128.5, 128.3, 127.6, 127.3, 126.6, 126.3, 125.1, 101.1, 93.3, 82.9, 69.0, 27.7, -0.3; **HRMS (ESI^+) $[M+Na]^+$** Calcd for $C_{21}H_{26}O_3NaSi$: 377.1543 m/z, Found: 377.1537 m/z.

tert-butyl (1-(naphthalen-1-yl)-3-(trimethylsilyl)prop-2-yn-1-yl) carbonate (S12)

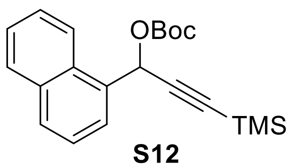

S12

Colorless oil. **IR (neat):** 3048 (w), 1732 (m), 1252 (m), 1156 (m), 843 (s) cm^{-1}; **^{1}H NMR (400 MHz, $CDCl_3$):** δ 8.27 (d, *J* = 8.4 Hz, 1H), 8.04 – 7.76 (m, 3H), 7.70 – 7.40 (m, 3H), 6.93 (s, 1H), 1.51 (s, 9H), 0.21 (s, 9H); **^{13}C NMR (100 MHz, $CDCl_3$):** δ 152.5, 133.9, 132.0, 130.6, 129.9, 128.7, 126.7, 126.5, 125.9, 125.1, 123.8, 101.0, 93.6, 83.0, 67.2, 27.7, -0.4; **HRMS (ESI^+) $[M+Na]^+$** Calcd for $C_{21}H_{26}O_3NaSi$: 377.1543 m/z, Found: 377.1545 m/z.

tert-butyl (1-(o-tolyl)-3-(trimethylsilyl)prop-2-yn-1-yl) carbonate (S13)

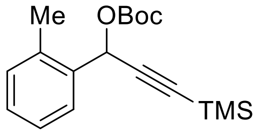

S13

Colorless oil. **IR (neat):** 2827 (w), 1742 (s), 1251 (s), 1158 (m), 843 (s) cm^{-1}; **^{1}H NMR (400 MHz, $CDCl_3$):** δ 7.64 – 7.56 (m, 1H), 7.30 – 7.20 (m, 2H), 7.20 – 7.14 (m, 1H), 6.38 (s, 1H), 2.42 (s, 3H), 1.48 (s, 9H), 0.17 (s, 9H); **^{13}C NMR (100 MHz, $CDCl_3$):** δ 152.4, 136.3, 134.7, 130.7, 128.9, 127.9, 126.2, 100.8, 92.8, 82.8, 66.8, 27.7, 19.0, -0.3; **HRMS (ESI^+) $[M+Na]^+$** Calcd for $C_{18}H_{26}O_3NaSi$: 341.1543 m/z, Found: 341.1545 m/z.

tert-butyl (1-(thiophen-3-yl)-3-(trimethylsilyl)prop-2-yn-1-yl) carbonate (S14)

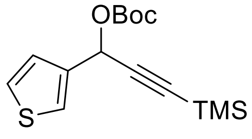

S14

Colorless oil. **IR (neat):** 1739 (s), 1252 (s), 1162 (m), 1082 (m), 1045 (m), 842 (s) cm^{-1}; **^{1}H NMR (400 MHz, $CDCl_3$):** δ 7.48 (d, *J* = 3.2 Hz, 1H), 7.29 (dd, *J* = 5.2, 3.2 Hz, 1H), 7.20 (d, *J* = 4.8 Hz, 1H), 6.31 (s, 1H), 1.49 (s, 9H), 0.20 (s, 9H); **^{13}C NMR (100 MHz, $CDCl_3$):** δ 152.4, 137.4, 126.8, 126.2, 125.0, 100.7, 92.1, 82.9, 64.1, 27.7, -0.4; **HRMS (ESI^+) $[M+Na]^+$** Calcd for $C_{15}H_{22}O_3NaSiS$: 333.0951 m/z, Found: 333.0960 m/z.

tert-butyl (1-(furan-3-yl)-3-(trimethylsilyl)prop-2-yn-1-yl) carbonate (S15)

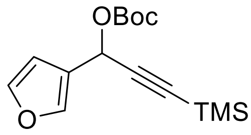

S15

Colorless oil. **IR (neat):** 2942 (w), 1735 (s), 1317 (m), 1155 (m), 1040 (m), 842 (s) cm^{-1}; **^{1}H NMR (400 MHz, $CDCl_3$):** δ 7.57 (s, 1H), 7.37 (s, 1H), 6.51 (s, 1H), 6.19 (s, 1H), 1.48 (s, 9H), 0.18 (s, 9H); **^{13}C NMR (100 MHz, $CDCl_3$):** δ 152.4, 143.4, 141.9, 122.4, 109.6, 100.2, 91.5, 82.9, 61.3, 27.7, -0.4; **HRMS (ESI^+) $[M+Na]^+$** Calcd for $C_{15}H_{22}O_4NaSi$: 317.1180 m/z, Found: 317.1185 m/z.

***tert*-butyl (1-(pyridin-3-yl)-3-(trimethylsilyl)prop-2-yn-1-yl) carbonate (S16)**

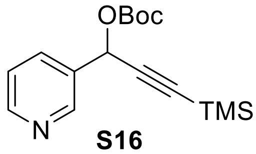

S16

Colorless oil. **IR (neat):** 2936 (w), 1744 (m), 1252 (s), 1159 (m), 1080 (m), 845 (s) cm^{-1}; **^{1}H NMR (400 MHz, $CDCl_3$):** δ 8.79 (d, *J* = 2.4 Hz, 1H), 8.61 (d, *J* = 4.8 Hz, 1H), 7.87 (d, *J* = 8.0 Hz, 1H), 7.33 (dd, *J* = 8.0, 4.8 Hz, 1H), 6.28 (s, 1H), 1.49 (s, 9H), 0.20 (s, 9H); **^{13}C NMR (100 MHz, $CDCl_3$):** δ 152.2, 150.2, 149.4, 135.4, 132.6, 123.5, 99.7, 94.2, 83.5, 66.6, 27.7, -0.4; **HRMS (ESI^+) $[M+H]^+$** Calcd for $C_{16}H_{24}NO_3Si$: 306.1520 m/z, Found: 306.1526 m/z.

1-(benzofuran-2-yl)-3-(trimethylsilyl)prop-2-yn-1-yl *tert*-butyl carbonate (S17)

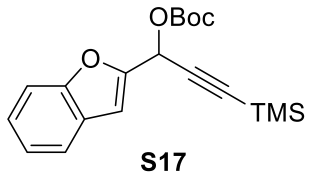

S17

White solid, m.p. 65 –66 °C; **IR (neat):** 2975 (w), 1738 (m), 1252 (s), 1155 (m), 1045 (m), 847 (s) cm^{-1}; **^{1}H NMR (400 MHz, $CDCl_3$):** δ 7.57 (d, *J* = 7.6 Hz, 1H), 7.48 (d, *J* = 8.0 Hz, 1H), 7.30 (t, *J* = 7.6 Hz, 1H), 7.22 (t, *J* = 7.6 Hz, 1H), 6.93 (s, 1H), 6.44 (s, 1H), 1.51 (s, 9H), 0.22 (s, 9H); **^{13}C NMR (100 MHz, $CDCl_3$):** δ 155.4, 152.1, 151.5, 127.4, 125.1, 123.0, 121.5, 111.6, 106.8, 97.9, 93.1, 83.4, 62.1, 27.6, -0.4; **HRMS (ESI^+) $[M+Na]^+$** Calcd for $C_{19}H_{24}O_4NaSi$: 367.1336 m/z, Found: 367.1330 m/z.

1-(benzo[*b*]thiophen-2-yl)-3-(trimethylsilyl)prop-2-yn-1-yl *tert*-butyl carbonate (S18)

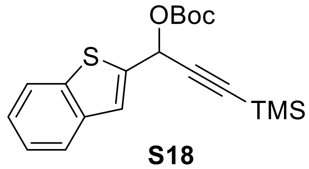

S18

White solid, m.p. 101 – 102 °C; **IR (neat):** 2973 (w), 1739 (m), 1316 (w), 1252 (m), 1155 (m), 1074 (m), 843 (s) cm^{-1}; **^{1}H NMR (400 MHz, $CDCl_3$):** δ 7.86 – 7.71 (m, 2H), 7.47 (s, 1H), 7.38 – 7.25 (m, 2H), 6.56 (s, 1H), 1.49 (s, 9H), 0.22 (s, 9H); **^{13}C NMR (100 MHz, $CDCl_3$):** δ 152.2, 140.4, 140.1, 138.8, 124.9, 124.4, 124.4, 124.1, 122.4, 99.6, 93.4, 83.3, 64.4, 27.7, -0.4 ; **HRMS (ESI^+) $[M+Na]^+$** Calcd for $C_{19}H_{24}O_3NaSiS$: 383.1108 m/z, Found: 383.1116 m/z.

***tert*-butyl 6-(1-((*tert*-butoxycarbonyl)oxy)-3-(trimethylsilyl)prop-2-yn-1-yl)-1H-indole-1-carboxylate (S19)**

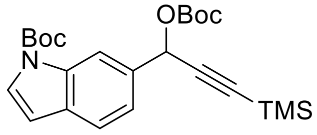

S19

Colorless oil. **IR (neat):** 2979 (w), 1738 (s), 1370 (m), 1250 (s), 1148 (m), 1128 (m), 844 (s) cm^{-1}; **^{1}H NMR (400 MHz, $CDCl_3$):** δ 8.36 (s, 1H), 7.63 (d, *J* = 3.6 Hz, 1H), 7.56 (d, *J* = 8.0 Hz, 1H), 7.45 (d, *J* = 8.0 Hz, 1H), 6.56 (d, *J* = 3.6 Hz, 1H), 6.38 (s, 1H), 1.69 (s, 9H), 1.48 (s, 9H), 0.20 (s, 9H); **^{13}C NMR (100 MHz, $CDCl_3$):** δ 152.4, 149.5, 134.9, 132.8, 131.1, 122.5, 121.0, 115.2, 107.0, 101.6, 92.7, 83.8, 82.7, 69.5, 28.2, 27.7, -0.3; **HRMS (ESI^+) $[M+Na]^+$** Calcd for $C_{24}H_{33}NO_5NaSi$: 466.2020 m/z, Found: 466.2010 m/z.

***tert*-butyl (3-(*tert*-butyldimethylsilyl)-1-phenylprop-2-yn-1-yl) carbonate (S20)**

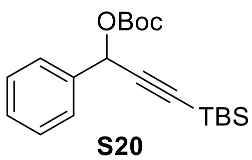

S20

Colorless oil. **IR (neat):** 2954 (w), 1744 (m), 1251 (s), 1160 (m), 1082 (m), 827 (s) cm^{-1}; **^{1}H NMR (400 MHz, $CDCl_3$):** δ 7.60 – 7.50 (m, 2H), 7.42 – 7.32 (m, 3H), 6.24 (s, 1H), 1.49 (s, 9H), 0.94 (s, 9H), 0.13 (s, 6H); **^{13}C NMR (100 MHz, $CDCl_3$):** δ 152.4, 136.6, 128.9, 128.5, 127.9, 101.7, 91.4, 82.8, 68.9, 27.7, 26.0, 16.6, -4.9; **HRMS (ESI$^+$) [M+Na]$^+$** Calcd for $C_{20}H_{30}O_3NaSi$: 369.1856 m/z, Found: 369.1861 m/z.

***tert*-butyl (3-cyclohexyl-1-phenylprop-2-yn-1-yl) carbonate (S21)**

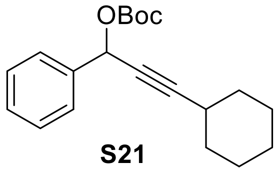

S21

Colorless oil. **IR (neat):** 2931 (w), 1739 (m), 1270 (m), 1251 (m), 1149 (s), 863 (m) cm^{-1}; **^{1}H NMR (400 MHz, $CDCl_3$):** δ 7.61 – 7.51 (m, 2H), 7.45 – 7.29 (m, 3H), 6.27 (s, 1H), 2.52 – 2.32 (m, 1H), 1.92 – 1.75 (m, 2H), 1.75 – 1.67 (m, 2H), 1.49 (app.s, 12H), 1.39 – 1.24 (m, 3H); **^{13}C NMR (100 MHz, $CDCl_3$):** δ 152.6, 137.4, 128.7, 128.4, 127.7, 92.8, 82.6, 76.4, 69.0, 32.2, 29.0, 27.7, 25.8, 24.6; **HRMS (ESI$^+$) [M+Na]$^+$** Calcd for $C_{20}H_{26}O_3Na$: 337.1774 m/z, Found: 337.1774 m/z.

***tert*-butyl (4-(*tert*-butyldimethylsilyl)-2-methylbut-3-yn-2-yl) carbonate (S22)**

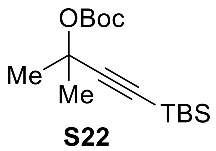

S22

Colorless oil. **IR (neat):** 2932 (w), 1749 (s), 1282 (m), 1249 (m), 1132 (s), 864 (w) cm^{-1}; **^{1}H NMR (400 MHz, $CDCl_3$):** δ 1.66 (s, 6H), 1.47 (s, 9H), 0.91 (s, 9H), 0.07 (s, 6H); **^{13}C NMR (100 MHz, $CDCl_3$):** δ 151.1, 106.6, 86.8, 81.8, 73.5, 29.0, 27.8, 26.0, 16.6, -4.8; **HRMS (ESI$^+$) [M+Na]$^+$** Calcd for $C_{16}H_{30}O_3NaSi$: 321.1856 m/z, Found: 321.1859 m/z.

***tert*-butyl (4-(*tert*-butyldimethylsilyl)-2-methylbut-3-yn-2-yl) carbonate (S23)**

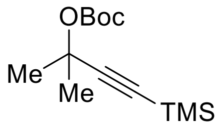

S23

Colorless oil. **IR (neat):** 2961 (w), 1750 (s), 1282 (m), 1248 (m), 869 (m), 843 (s) cm^{-1}; **^{1}H NMR (400 MHz, $CDCl_3$):** δ 1.65 (s, 6H), 1.47 (s, 9H), 0.14 (s, 9H); **^{13}C NMR (100 MHz, $CDCl_3$):** δ 151.2, 106.0, 88.4, 81.9, 73.6, 28.9, 27.7, -0.2; **HRMS (ESI$^+$) [M+Na]$^+$** Calcd for $C_{13}H_{24}O_3NaSi$: 279.1387 m/z, Found: 279.1396 m/z.

***tert*-butyl (1-((trimethylsilyl)ethynyl)cyclopentyl) carbonate (S24)**

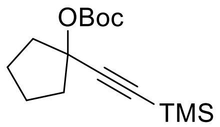

S24

Colorless oil. **IR (neat):** 2960 (w), 1742 (s), 1278 (m), 1249 (m), 1151 (s), 843 (s) cm^{-1}; **^{1}H NMR (400 MHz, $CDCl_3$):** δ 2.37 – 2.14 (m, 2H), 2.16 – 2.00 (m, 2H), 1.80 – 1.63 (m, 4H), 1.47 (s, 9H), 0.12 (s, 9H); **^{13}C NMR (100 MHz, $CDCl_3$):** δ 151.6, 105.6, 89.0, 82.4, 82.0, 40.4, 27.7, 23.4, -0.2; **HRMS (ESI$^+$) [M+Na]$^+$** Calcd for $C_{15}H_{26}O_3NaSi$: 305.1543 m/z, Found: 305.1551 m/z.

***tert*-butyl (1-((trimethylsilyl)ethynyl)cyclohexyl) carbonate (S25)**

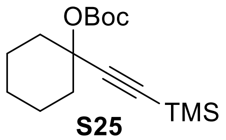

S25

Colorless oil. **IR (neat):** 3045 (w), 1748 (s), 1281 (m), 1247 (m), 1160 (s), 864 (s), 843 (s) cm^{-1}; **^{1}H NMR (400 MHz, $CDCl_3$):** δ 2.23 – 2.07 (m, 2H), 1.90 – 1.71 (m, 2H), 1.67 – 1.54 (m, 5H), 1.48 (s, 9H), 1.38 – 1.18 (m, 1H), 0.15 (s, 9H); **^{13}C NMR (100 MHz, $CDCl_3$):** δ 151.1, 105.0, 90.9, 81.8, 77.4, 37.0, 27.8, 25.1, 22.8, -0.1; **HRMS (ESI$^+$) [M+Na]$^+$** Calcd for $C_{16}H_{28}O_3NaSi$: 319.1700 m/z, Found: 319.1702 m/z.

***tert*-butyl (3-cyclopentyl-1-phenylprop-2-yn-1-yl) carbonate (S29)**

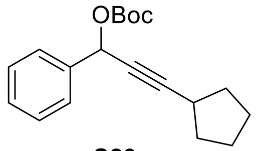

S29

Colorless oil. **IR (neat):**; 2938 (w), 1743 (m), 1269 (m), 1253 (m), 1150 (s), 855 (m) cm^{-1} **^{1}H NMR (400 MHz, CDCl$_3$):** δ 7.53 (d, *J* = 6.8 Hz, 2H), 7.43 – 7.27 (m, 3H), 6.26 (s, 1H), 2.68 (p, *J* = 7.2 Hz, 1H), 1.95 – 1.82 (m, 2H), 1.78 – 1.58 (m, 4H), 1.56 – 1.50 (m, 2H), 1.46 (s, 9H); **^{13}C NMR (100 MHz, CDCl$_3$):** δ 152.5, 137.4, 128.6, 128.3, 127.6, 92.9, 82.4, 75.9, 68.9, 33.4, 30.0, 27.6, 24.8; **HRMS (ESI$^+$) [M+Na]$^+$** Calcd for $C_{17}H_{22}O_3Na$: 297.1461 m/z, Found: 297.1458 m/z.

***tert*-butyl (1-phenylhex-2-yn-1-yl) carbonate (S30)**

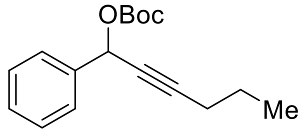

S30

Colorless oil. **IR (neat):**; 2933 (w), 1740 (m), 1265 (m), 1254 (m), 1148 (s), 861 (m) cm^{-1} **^{1}H NMR (400 MHz, CDCl$_3$):** δ 7.66 – 7.44 (m, 2H), 7.41 – 7.26 (m, 3H), 6.24 (s, 1H), 2.24 (td, *J* = 7.2, 2.0 Hz, 2H), 1.62 – 1.51 (m, 2H), 1.48 (s, 9H), 0.98 (t, *J* = 7.2 Hz, 3H); **^{13}C NMR (100 MHz, CDCl$_3$):** δ 152.6, 137.4, 128.8, 128.5, 127.7, 88.8, 82.7, 69.0, 27.7, 21.8, 20.8, 13.4; **HRMS (ESI$^+$) [M+Na]$^+$** Calcd for $C_{19}H_{24}O_3Na$: 323.1618 m/z, Found: 323.1616 m/z.

■ Representative Experimental Procedure for Synthesis of 3a

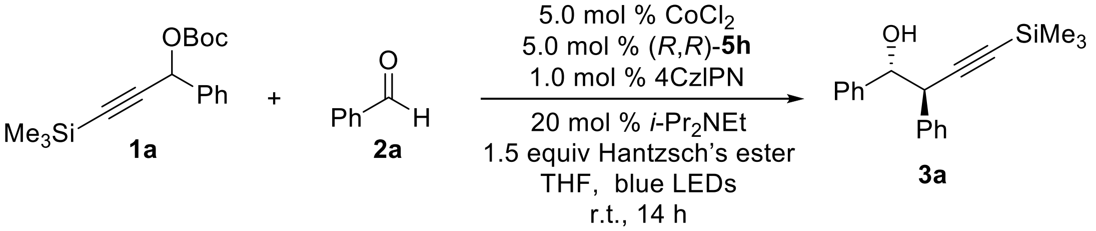

In a N_2-filled glove-box, an oven-dried vial (8 mL) equipped with a magnetic stir bar was charged with $CoCl_2$ (2.6 mg, 0.02 mmol, 5.0 mol %), (*R*, *R*)-**5h** (6.2 mg, 0.02 mmol, 5.0 mol %). THF (3.0 mL) was added, then the mixture was allowed to stir at room temperature for 20 min. **2a** (42.3 mg, 0.4 mmol, 1.0 equiv), **1a** (182.6 mg, 0.6 mmol, 1.5 equiv), *i*-Pr_2NEt (10.3 mg, 0.08 mmol, 0.2 equiv), 4CzIPN (3.1 mg, 0.004 mmol, 1.0 mol %) and Hantzsch's ester (151.9 mg, 0.6 mmol, 1.5 equiv) were added to the solution. The vial was sealed with a cap (phenolic open top cap with red PTFE/white silicone septum) and taken out of the glove box. It was irradiated by 40 W blue LEDs (450-455 nm, λ_{max} = 454 nm) and allowed to stir at room temperature (about 22 ^{o}C) for 14 h with cooling fans.

Workup: The mixture is filtered through a short plug of 100-200 mesh silica gel eluting with diethyl ether (3×30 mL). The filtrate is concentrated under reduced pressure and the residue was purified by silica-gel column chromatography (eluent: Petroleum ether/ diethyl ether = 12:1) to afford the **3a** as white solid (108.3 mg, 0.37 mmol, 92%).

The blue LEDs (**Supplementary Figure 1**) were purchased online from XuZhou Aijia Electronic Technology Co., Ltd.

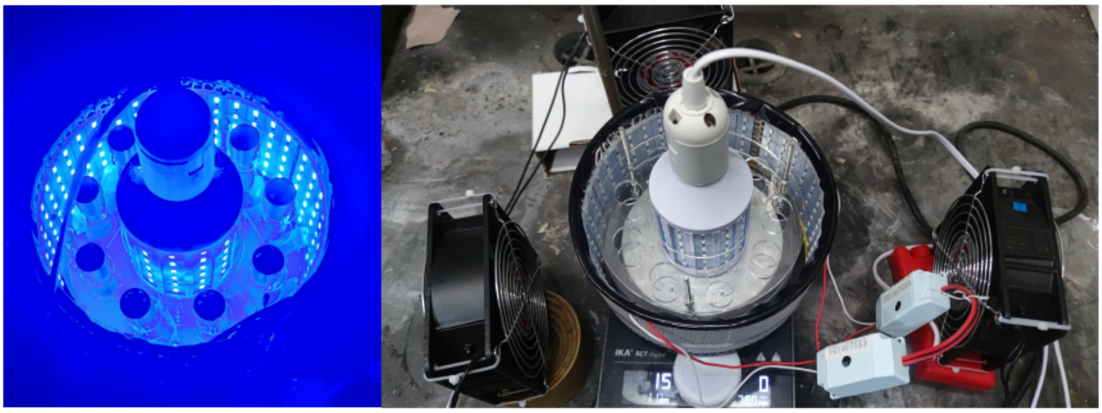

Supplementary Figure 1. Photochemical setup

■ Additional Optimization of Reaction Conditions.

Supplementary Table 1. Screen of photocatalysts

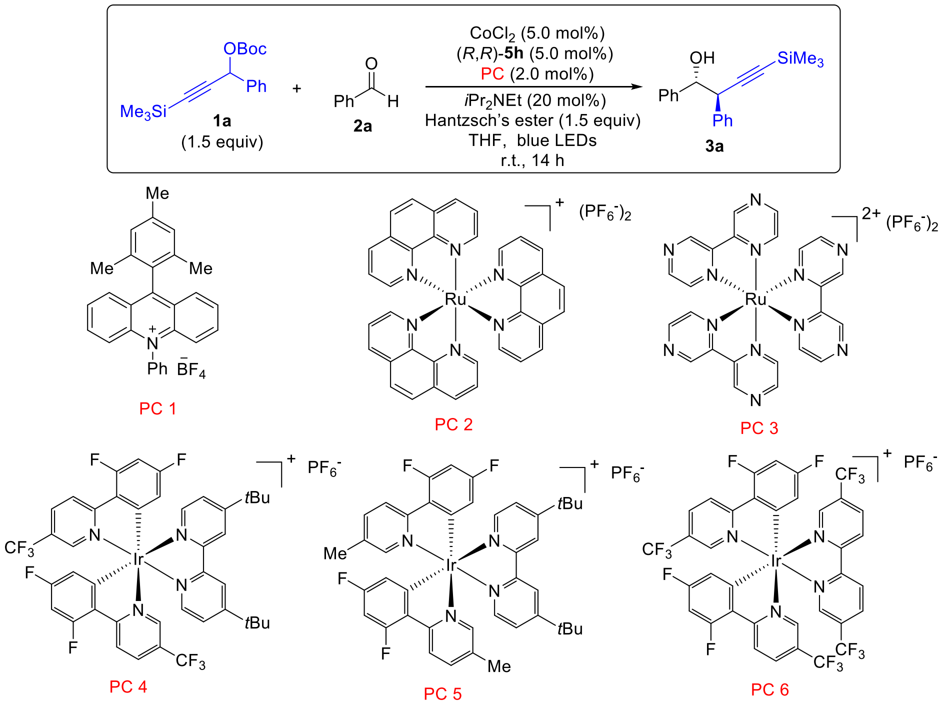

PC	Yield (%)	dr	er
PC 1	<5	NA	NA
PC 2	<5	NA	NA
PC 3	<5	NA	NA
PC 4	74	>95:5	96:4
PC 5	87	>95:5	96:4
PC 6	<5	NA	NA

Supplementary Table 2. Screen of bases

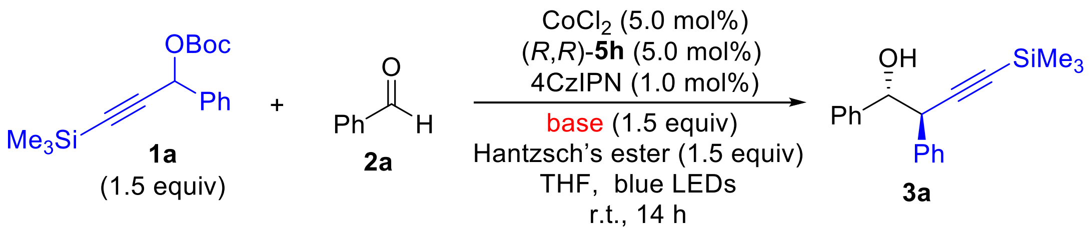

Base	Yield (%)	dr	er
DABCO	34	>95:5	85:15
DBU	61	>95:5	87:13
Et_3N	88	>95:5	95:5
iPr_2NEt	92	>95:5	97:3
Pyridine	<5	NA	NA
DMAP	49	>95:5	85:15
Proton sponge	<5	NA	NA
Cs_2CO_3	<5	NA	NA

Supplementary Table 3. The effect of *i*Pr_2NEt equivalent and HE equivalent

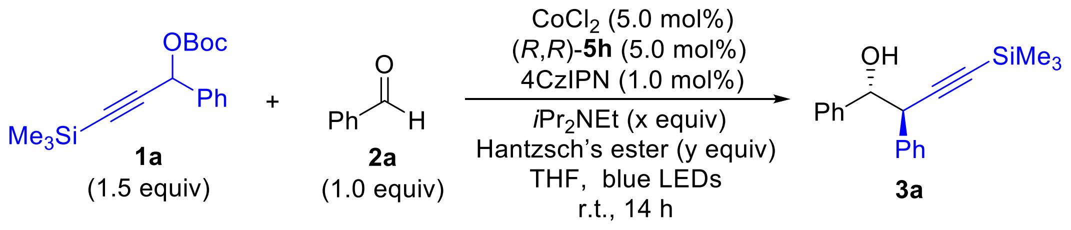

Equivalent of *i*Pr_2NEt (x)	Equivalent of HE (y)	Yield (%)	dr	er
1.5	1.5	94	>95:5	96:4
1.0	1.5	92	>95:5	97:3
0.5	1.5	95	>95:5	96:4
0.2	1.5	92	>95:5	97:3
0	1.5	<5	NA	NA
1.5	0	<5	NA	NA
0.2	1.2	84	>95:5	96:4

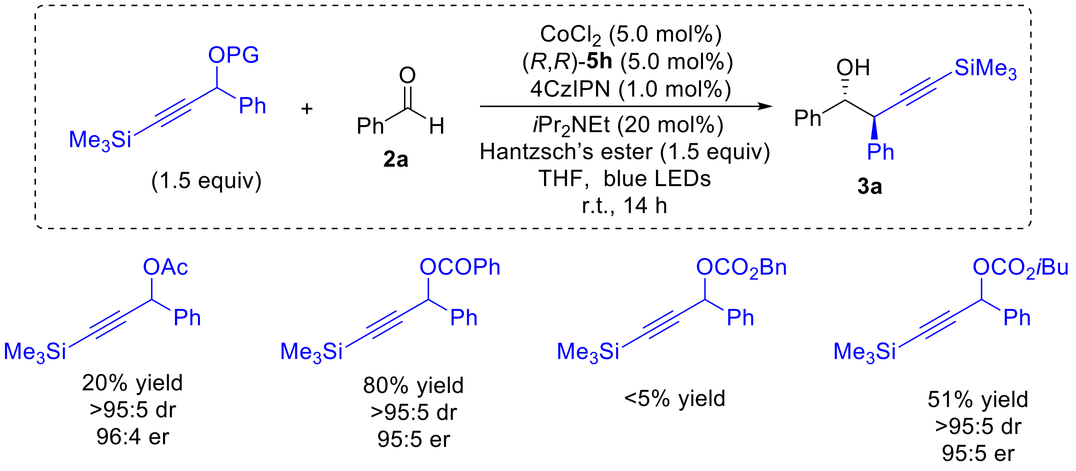

Supplementary Figure 2. The effect of protecting group

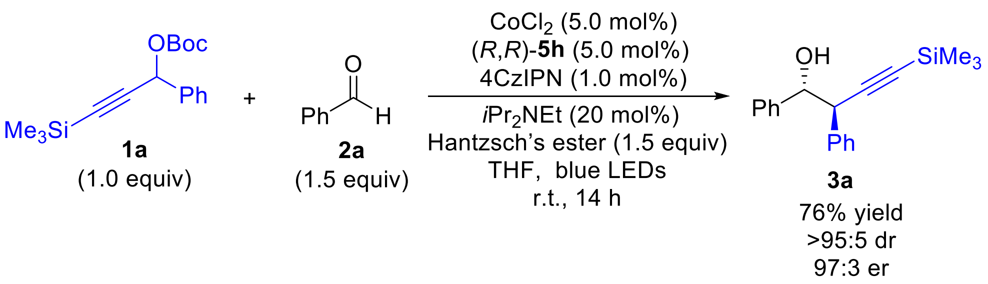

Supplementary Figure 3. Effect of ratio of 1a/2a

■ Characterization of Product

(1*R*,2*R*)-1,2-diphenyl-4-(trimethylsilyl)but-3-yn-1-ol (3a)

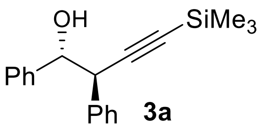

White solid, m.p. 39–40 °C; **IR (neat):** 3354 (w), 2176 (w), 1250 (m), 1042 (m), 841 (s), 764 (m), 697 (m), 675 (m) cm^{-1}; **^{1}H NMR (400 MHz, $CDCl_3$):** δ 7.27 – 7.19 (m, 6H), 7.19 – 7.12 (m, 4H), 4.74 (dd, *J* = 6.4, 3.6 Hz, 1H), 3.93 (d, *J* = 6.4 Hz, 1H), 2.76 (d, *J* = 3.6 Hz, 1H), 0.20 (s, 9H); **^{13}C NMR (100 MHz, $CDCl_3$):** δ 140.6, 137.3, 128.5, 128.2, 127.8, 127.8, 127.2, 126.6, 104.5, 90.7, 78.0, 49.0, -0.0; **HRMS (ESI$^+$) [M+Na]$^+$** Calcd for $C_{19}H_{22}ONaSi$: 317.1332 m/z, Found: 317.1336 m/z; **Specific rotation**: $[\alpha]_D^{20}$ -19.72 (*c* 1.00, $CHCl_3$) for an enantiomerically enriched sample of 97:3 e.r.

Enantiomeric purity of **3a** was determined by HPLC analysis in comparison with authentic racemic material (97:3 e.r. shown; Chiralpak IF column, 99:1 *n*-hexane / *i*-PrOH, 0.6 mL/min, 220 nm).

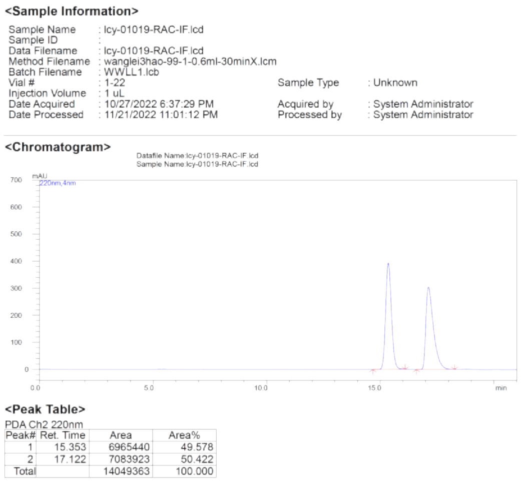

<Sample Information>

Sample Name : lcy-01019-RAC-IF.lcd
Sample ID :
Data Filename : lcy-01019-RAC-IF.lcd
Method Filename : wanglei3hao-99-1-0.6ml-30minX.lcm
Batch Filename : WWLL1.lcb
Vial # : 1-22　Sample Type : Unknown
Injection Volume : 1 uL
Date Acquired : 10/27/2022 6:37:29 PM　Acquired by : System Administrator
Date Processed : 11/21/2022 11:01:12 PM　Processed by : System Administrator

<Chromatogram>

<Peak Table>

PDA Ch2 220nm

Peak#	Ret. Time	Area	Area%
1	15.353	6965440	49.578
2	17.122	7083923	50.422
Total		14049363	100.000

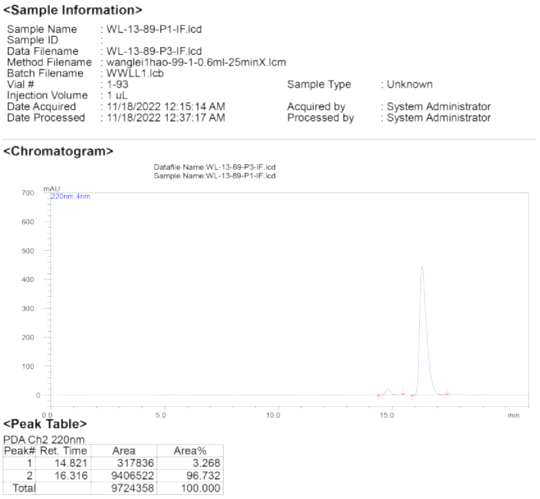

<Sample Information>

Sample Name : WL-13-89-P1-IF.lcd
Sample ID :
Data Filename : WL-13-89-P3-IF.lcd
Method Filename : wanglei1hao-99-1-0.6ml-25minX.lcm
Batch Filename : WWLL1.lcb
Vial # : 1-93　Sample Type : Unknown
Injection Volume : 1 uL
Date Acquired : 11/18/2022 12:15:14 AM　Acquired by : System Administrator
Date Processed : 11/18/2022 12:37:17 AM　Processed by : System Administrator

<Chromatogram>

<Peak Table>

PDA Ch2 220nm

Peak#	Ret. Time	Area	Area%
1	14.821	317836	3.268
2	16.316	9406522	96.732
Total		9724358	100.000

(1*R*,2*R*)-2-phenyl-1-(4-(trifluoromethyl)phenyl)-4-(trimethylsilyl)but-3-yn-1-ol (3b)

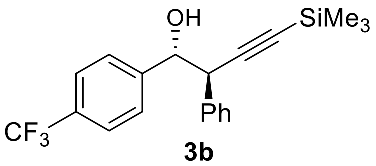

3b

White solid, m.p. 45–47°C; **IR (neat):** 3352 (w), 2177 (w), 1326 (s), 1127 (m), 1066 (m), 838 (s), 761 (m) cm^{-1}; **^{1}H NMR (400 MHz, $CDCl_3$):** δ 7.49 (d, *J* = 8.0 Hz, 2H), 7.31 – 7.20 (m, 5H), 7.20 – 7.09 (m, 2H), 4.80 (dd, *J* = 6.4, 3.6 Hz, 1H), 3.91 (d, *J* = 6.4 Hz, 1H), 2.81 (d, *J* = 3.6 Hz, 1H), 0.19 (s, 9H); **^{13}C NMR (100 MHz, $CDCl_3$) :** δ 144.6, 136.7, 129.9 (q, *J* = 32.3 Hz), 128.5, 128.4, 127.6, 127.0, 124.7 (q, *J* = 3.8 Hz), 124.1 (q, *J* = 270 Hz), 103.7, 91.3, 77.3, 48.9, -0.1; **^{19}F NMR (376 MHz, $CDCl_3$):** δ -62.5; **HRMS (EI$^+$) [M-H_2O]$^+$**Calcd for $C_{20}H_{19}F_3Si$: 344.1203 m/z, Found: 344.1207 m/z; **Specific rotation**: $[\alpha]_D^{20}$ -19.39 (*c* 2.00, $CHCl_3$) for an enantiomerically enriched sample of 95:5 e.r.

Enantiomeric purity of **3b** was determined by SFC analysis in comparison with authentic racemic material (95:5 e.r. shown; Chiralcel OZ-H column, 95:5 CO_2 / *i*-PrOH, 0.8 mL/min, 220 nm).

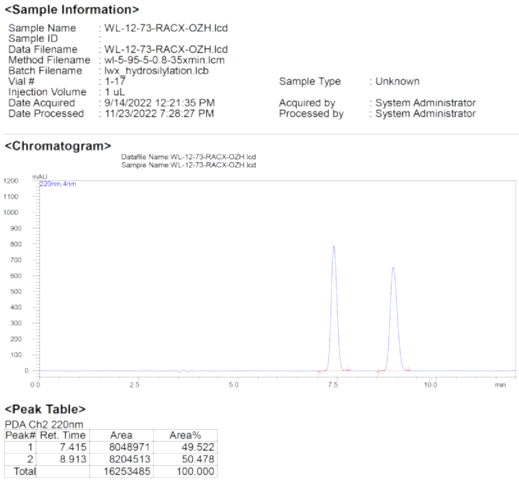

<Sample Information>

Sample Name : WL-12-73-RACX-OZH.lcd
Sample ID :
Data Filename : WL-12-73-RACX-OZH.lcd
Method Filename : wl-5-95-5-0.8-35xmin.lcm
Batch Filename : lwx_hydrosilylation.lcb
Vial # : 1-17　Sample Type : Unknown
Injection Volume : 1 uL
Date Acquired : 9/14/2022 12:21:35 PM　Acquired by : System Administrator
Date Processed : 11/23/2022 7:28:27 PM　Processed by : System Administrator

<Chromatogram>

<Peak Table>

PDA Ch2 220nm

Peak#	Ret. Time	Area	Area%
1	7.415	8048971	49.522
2	8.913	8204513	50.478
Total		16253485	100.000

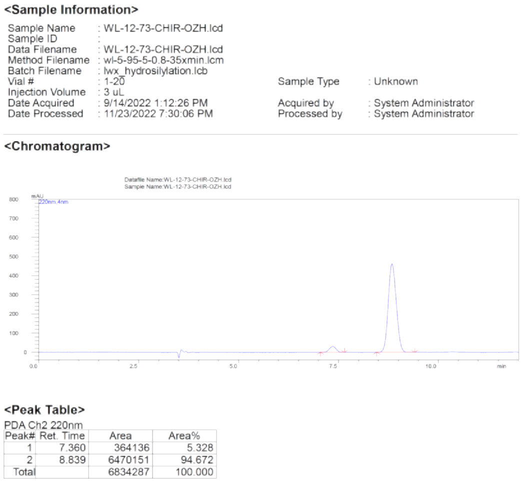

<Sample Information>

Sample Name : WL-12-73-CHIR-OZH.lcd
Sample ID :
Data Filename : WL-12-73-CHIR-OZH.lcd
Method Filename : wl-5-95-5-0.8-35xmin.lcm
Batch Filename : lwx_hydrosilylation.lcb
Vial # : 1-20　Sample Type : Unknown
Injection Volume : 3 uL
Date Acquired : 9/14/2022 1:12:26 PM　Acquired by : System Administrator
Date Processed : 11/23/2022 7:30:06 PM　Processed by : System Administrator

<Chromatogram>

<Peak Table>

PDA Ch2 220nm

Peak#	Ret. Time	Area	Area%
1	7.360	364136	5.328
2	8.839	6470151	94.672
Total		6834287	100.000

4-((1*R*,2*R*)-1-hydroxy-2-phenyl-4-(trimethylsilyl)but-3-yn-1-yl)benzonitrile (3c)

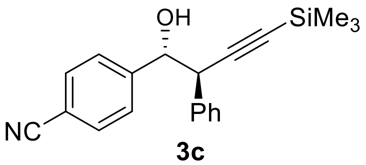

3c

White solid, m.p. 111–113 °C; **IR (neat):** 3461 (w), 2237 (w), 1670 (m), 1249 (w), 1067 (w), 842 (s), 778 (m), 712 (m) cm^{-1};**^{1}H NMR (400 MHz, $CDCl_3$):** δ 7.52 (d, J = 8.4 Hz, 2H), 7.31 – 7.21 (m, 5H), 7.16 – 7.11 (m, 2H), 4.82 (d, J = 4.8 Hz, 1H), 3.90 (d, J = 6.4 Hz, 1H), 2.83 (s, 1H), 0.19 (s, 9H); **^{13}C NMR (100 MHz, $CDCl_3$) :** δ 145.8, 136.3, 131.5, 128.4, 127.6, 127.4, 118.7, 111.4, 103.4, 91.4, 77.1, 48.6, -0.1; **HRMS (DART$^+$) [M+H]$^+$** Calcd for $C_{20}H_{22}ONSi$: 320.1465 m/z, Found: 320.1467 m/z; **Specific rotation**: $[\alpha]_D^{20}$ -37.15 (c 1.00, $CHCl_3$) for an enantiomerically enriched sample of 95:5 e.r.

Enantiomeric purity of **3c** was determined by HPLC analysis in comparison with authentic racemic material (95:5 e.r. shown; Chiralpak IF column, 99:1 n-hexane / i-PrOH, 0.8 mL/min, 220 nm).

<Sample Information>

Sample Name : wanglei-12-100-RAC-IF.lcd
Sample ID :
Data Filename : wanglei-12-100-RAC2X-IF.lcd
Method Filename : wanglei5hao-99-1-0.8ml-60minX.lcm
Batch Filename : WWLL1.lcb
Vial # : 1-92 Sample Type : Unknown
Injection Volume : 6 uL
Date Acquired : 10/2/2022 1:01:05 PM Acquired by : System Administrator
Date Processed : 11/21/2022 11:44:54 PM Processed by : System Administrator

<Chromatogram>

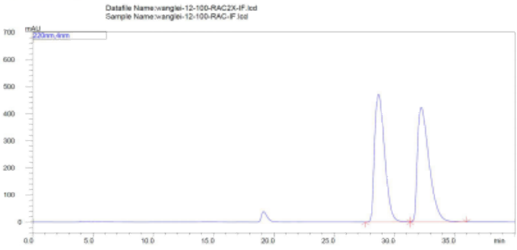

<Peak Table>

PDA Ch2 220nm

Peak#	Ret. Time	Area	Area%
1	28.686	27386610	49.034
2	32.233	28466112	50.966
Total		55852723	100.000

<Sample Information>

Sample Name : wanglei-12-100-RAC-IF.lcd
Sample ID :
Data Filename : wanglei-12-100-CHIR-IF.lcd
Method Filename : wanglei5hao-99-1-0.8ml-60minX.lcm
Batch Filename : WWLL1.lcb
Vial # : 1-98 Sample Type : Unknown
Injection Volume : 2 uL
Date Acquired : 10/2/2022 5:27:17 PM Acquired by : System Administrator
Date Processed : 11/21/2022 11:47:46 PM Processed by : System Administrator

<Chromatogram>

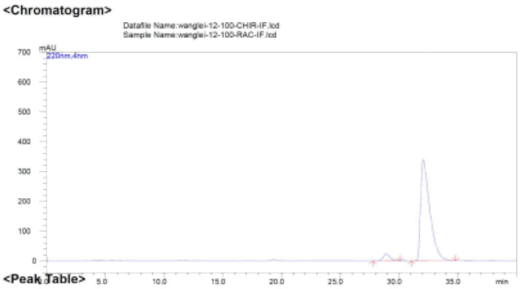

<Peak Table>

PDA Ch2 220nm

Peak#	Ret. Time	Area	Area%
1	28.890	940168	4.821
2	32.058	18560740	95.179
Total		19500908	100.000

Methyl 4-((1*R*,2*R*)-1-hydroxy-2-phenyl-4-(trimethylsilyl)but-3-yn-1-yl)benzoate (3d)

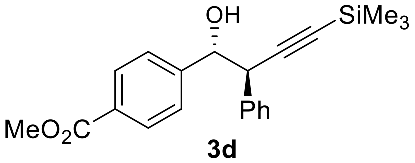

3d

White solid, m.p. 114–116 °C; **IR (neat):** 3474 (w), 2181 (w), 1698 (s), 1437 (w), 1285 (s), 874 (s), 756 (s), 723 (m) cm^{-1};**^{1}H NMR (400 MHz, $CDCl_3$):** δ 7.91 (d, J = 8.4 Hz, 2H), 7.32 – 7.19 (m, 5H), 7.18 – 7.11 (m, 2H), 4.81 (d, J = 5.2 Hz, 1H), 3.93 (d, J = 6.4 Hz, 1H), 3.90 (s, 3H), 2.82 (s, 1H), 0.20 (s, 9H); **^{13}C NMR (100 MHz, $CDCl_3$) :** δ 166.9, 145.6, 136.8, 129.5, 129.1, 128.5, 128.4, 127.5, 126.7, 103.9, 91.2, 77.6, 52.0, 48.9, -0.1; **HRMS (ESI$^+$) [M+Na]$^+$** Calcd for $C_{21}H_{24}O_3NaSi$: 375.1387 m/z, Found: 375.1381 m/z; **Specific rotation**: $[\alpha]_D^{20}$ -26.34 (c 1.00, $CHCl_3$) for an enantiomerically enriched sample of 96:4 e.r.

Enantiomeric purity of **3d** was determined by HPLC analysis in comparison with authentic racemic material (96:4 e.r. shown; Chiralpak IF column, 99:1 n-hexane / i-PrOH, 0.8 mL/min, 220 nm).

<Sample Information>

Sample Name : wanglei-12-99-RAC-IF.lcd
Sample ID :
Data Filename : wanglei-12-99-RAC2X-IF.lcd
Method Filename : wanglei5hao-99-1-0.8ml-60minX.lcm
Batch Filename : WWLL1.lcb
Vial # : 1-91 Sample Type : Unknown
Injection Volume : 10 uL
Date Acquired : 10/2/2022 12:00:32 PM Acquired by : System Administrator
Date Processed : 10/3/2022 6:12:53 PM Processed by : System Administrator

<Chromatogram>

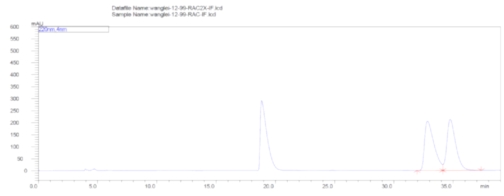

<Peak Table>

PDA Ch2 220nm

Peak#	Ret. Time	Area	Area%
1	32.835	11427792	50.740
2	34.751	11094532	49.260
Total		22522325	100.000

<Sample Information>

Sample Name : wanglei-12-99-RAC-IF.lcd
Sample ID :
Data Filename : wanglei-12-99-CHIRX-IF.lcd
Method Filename : wanglei5hao-99-1-0.8ml-60minX.lcm
Batch Filename : WWLL1.lcb
Vial # : 1-97 Sample Type : Unknown
Injection Volume : 5 uL
Date Acquired : 10/2/2022 4:26:43 PM Acquired by : System Administrator
Date Processed : 10/3/2022 6:14:01 PM Processed by : System Administrator

<Chromatogram>

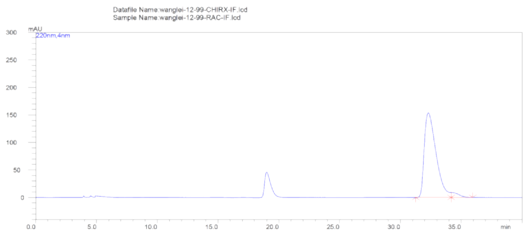

<Peak Table>

PDA Ch2 220nm

Peak#	Ret. Time	Area	Area%
1	32.296	10337877	96.451
2	34.193	380370	3.549
Total		10718247	100.000

1-(4-((1*R*,2*R*)-1-hydroxy-2-phenyl-4-(trimethylsilyl)but-3-yn-1-yl)phenyl)ethan-1-one (3e)

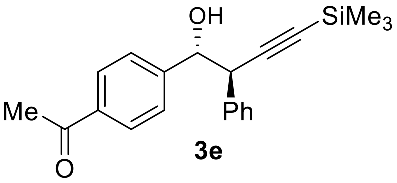

3e

White solid, m.p. 151–153 °C; **IR (neat):** 3492 (w), 2183 (w), 1665 (m), 1361 (m), 1248 (m), 842 (s), 760 (m), 711 (m) cm^{-1}; **^{1}H NMR (400 MHz, CDCl$_3$):** δ 7.83 (d, *J* = 8.0 Hz, 2H), 7.41 – 7.18 (m, 5H), 7.23 – 7.09 (m, 2H), 4.83 (dd, *J* = 6.4, 3.6 Hz, 1H), 3.94 (d, *J* = 6.4 Hz, 1H), 2.85 (d, *J* = 3.6 Hz, 1H), 2.58 (s, 3H), 0.20 (s, 9H); **^{13}C NMR (100 MHz, CDCl$_3$) :** δ 197.9, 145.9, 136.7, 136.4, 128.4, 128.3, 127.8, 127.4, 126.8, 103.8, 91.0, 77.4, 48.7, 26.6, -0.1; **HRMS (ESI$^+$) [M+Na]$^+$** Calcd for $C_{21}H_{24}O_2NaSi$: 359.1438 m/z, Found: 359.1437 m/z; **Specific rotation**: $[\alpha]_D^{20}$ -35.02 (*c* 1.00, CHCl$_3$) for an enantiomerically enriched sample of 99:1 e.r.

Enantiomeric purity of **3e** was determined by HPLC analysis in comparison with authentic racemic material (99:1 e.r. shown; Chiralpak IG column, 92:8 *n*-hexane / *i*-PrOH, 0.8 mL/min, 254 nm).

<Sample Information>

Sample Name : wanglei-13-20-rac-IG.lcd
Sample ID :
Data Filename : wanglei-13-20-rac-IG.lcd
Method Filename : wanglei6hao-92-8-0.8ml-25minX.lcm
Batch Filename : WWLL1.lcb
Vial # : 1-94 Sample Type : Unknown
Injection Volume : 3 uL
Date Acquired : 10/15/2022 10:28:41 PM Acquired by : System Administrator
Date Processed : 10/15/2022 10:53:45 PM Processed by : System Administrator

<Chromatogram>

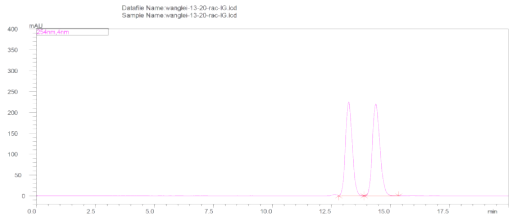

<Peak Table>

PDA Ch1 254nm

Peak#	Ret. Time	Area	Area%
1	13.233	4426932	49.452
2	14.381	4525099	50.548
Total		8952031	100.000

<Sample Information>

Sample Name : wanglei-13-20-chir-IG.lcd
Sample ID :
Data Filename : wanglei-13-20-chir-IG.lcd
Method Filename : wanglei6hao-92-8-0.8ml-25minX.lcm
Batch Filename : WWLL1.lcb
Vial # : 1-77 Sample Type : Unknown
Injection Volume : 2 uL
Date Acquired : 10/15/2022 10:54:13 PM Acquired by : System Administrator
Date Processed : 11/22/2022 1:12:57 AM Processed by : System Administrator

<Chromatogram>

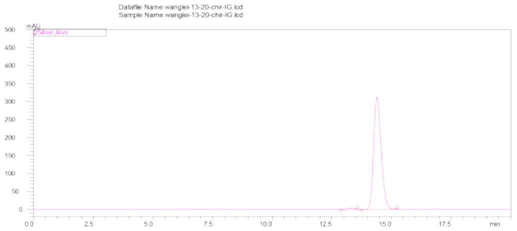

<Peak Table>

PDA Ch1 254nm

Peak#	Ret. Time	Area	Area%
1	13.262	59303	0.858
2	14.389	6855741	99.142
Total		6915044	100.000

(1*R*,2*R*)-2-phenyl-1-(3-(trifluoromethyl)phenyl)-4-(trimethylsilyl)but-3-yn-1-ol (3f)

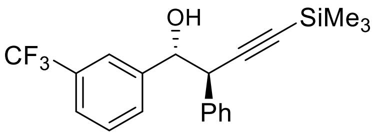

3f

Colorless oil. **IR (neat):** 3347 (w), 2175 (w), 1329 (s), 1146 (s), 1126 (m), 843 (s), 759 (m), 656 (s) cm^{-1}; **^{1}H NMR (400 MHz, $CDCl_3$):** δ 7.50 (d, *J* = 7.6 Hz, 1H), 7.44 (s, 1H), 7.40 – 7.29 (m, 2H), 7.30 – 7.22 (m, 3H), 7.22 – 7.12 (m, 2H), 4.83 (d, *J* = 6.0 Hz, 1H), 3.93 (d, *J* = 6.0 Hz, 1H), 2.79 (s, 1H), 0.19 (s, 9H); **^{13}C NMR (100 MHz, $CDCl_3$) :** δ 141.6, 136.7, 130.2 (q, *J* = 32.0 Hz), 130.0, 128.5, 128.4, 128.2, 127.6, 124.5 (d, *J* = 3.8 Hz), 124.1 (q, *J* = 271 Hz), 123.5 (q, *J* = 3.9 Hz), 103.5, 91.4, 77.3, 48.9, -0.1; **^{19}F NMR (376 MHz, $CDCl_3$):** δ -62.6; **HRMS (EI^+) $[M-H_2O]^+$** Calcd for $C_{20}H_{19}F_3Si$: 344.1203 m/z, Found: 344.1203 m/z; **Specific rotation**: $[\alpha]_D^{20}$ -12.33 (*c* 2.00, $CHCl_3$) for an enantiomerically enriched sample of 95:5 e.r.

Enantiomeric purity of **3f** was determined by HPLC analysis in comparison with authentic racemic material (95:5 e.r. shown; Chiralcel OJ-H column, 99.5:0.5 *n*-hexane / *i*-PrOH, 0.8 mL/min, 220 nm).

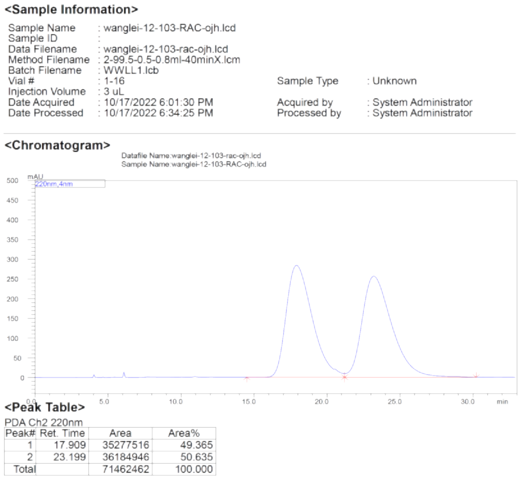

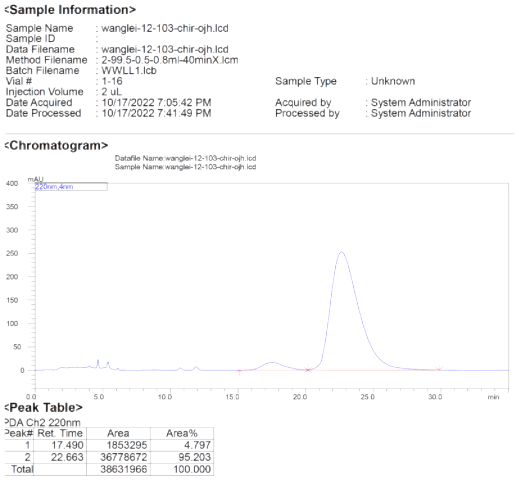

(1*R*,2*R*)-1-(4-methoxyphenyl)-2-phenyl-4-(trimethylsilyl)but-3-yn-1-ol (3g)

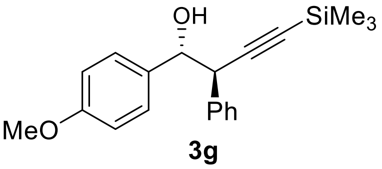

3g

Colorless oil. **IR (neat):** 3323 (w), 2173 (w), 1512 (m), 1247 (s), 1036 (m), 840 (s), 760 (m) cm^{-1}; **^{1}H NMR (400 MHz, $CDCl_3$):** δ 7.26 – 7.17 (m, 3H), 7.18 – 7.09 (m, 2H), 7.06 (d, *J* = 8.8 Hz, 2H), 6.76 (d, *J* = 8.8 Hz, 2H), 4.68 (dd, *J* = 6.8, 3.2 Hz, 1H), 3.89 (d, *J* = 6.8 Hz, 1H), 3.75 (s, 3H), 2.76 (d, *J* = 3.2 Hz, 1H), 0.20 (s, 9H); **^{13}C NMR (100 MHz, $CDCl_3$) :** δ 159.1, 137.3, 132.7, 128.5, 128.1, 127.8, 127.1, 113.2, 104.8, 90.5, 77.6, 55.1, 49.0, -0.0; **HRMS (ESI^+) $[M+Na]^+$** Calcd for $C_{20}H_{24}O_2NaSi$: 347.1438 m/z, Found: 347.1434 m/z; **Specific rotation**: $[\alpha]_D^{20}$ -29.07 (*c* 2.00, $CHCl_3$) for an enantiomerically enriched sample of 97:3 e.r.

Enantiomeric purity of **3g** was determined by SFC analysis in comparison with authentic racemic material (97:3 e.r. shown; Chiralcel OZ-H column, 94:6 CO_2 / *i*-PrOH, 0.8 mL/min, 220 nm).

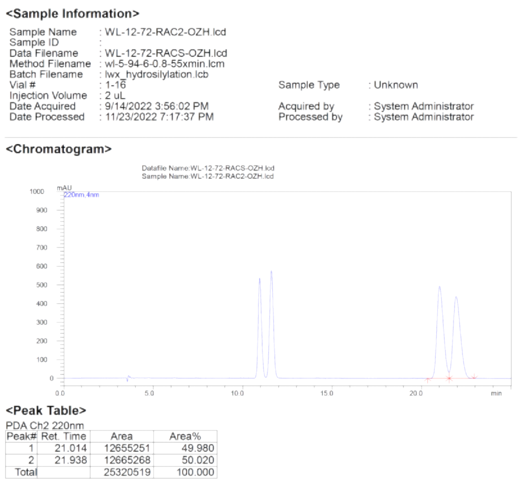

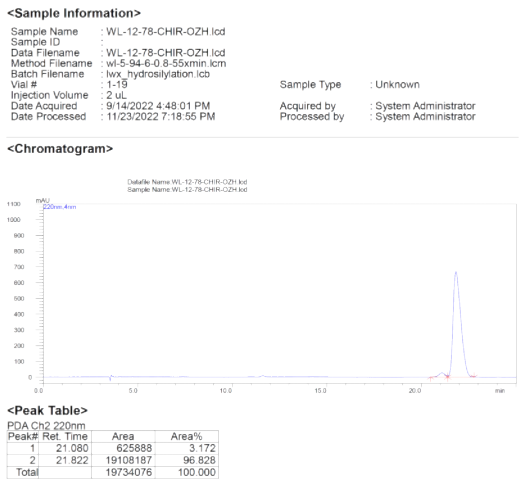

(1*R*,2*R*)-1-(3-methoxyphenyl)-2-phenyl-4-(trimethylsilyl)but-3-yn-1-ol (3h)

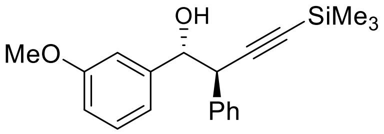

3h

Colorless oil. **IR (neat):** 3427 (w), 2174 (w), 1602 (m), 1250 (s), 1042 (m), 842 (s), 760 (m) cm^{-1}; **^{1}H NMR (400 MHz, $CDCl_3$):** δ 7.24 – 7.18 (m, 3H), 7.18 – 7.08 (m, 3H), 6.81 – 6.73 (m, 1H), 6.74 – 6.65 (m, 2H), 4.70 (dd, *J* = 6.4, 3.6 Hz, 1H), 3.91 (d, *J* = 6.4 Hz, 1H), 3.68 (s, 3H), 2.80 (d, *J* = 2.8 Hz, 1H), 0.19 (s, 9H); **^{13}C NMR (100 MHz, $CDCl_3$) :** δ 159.1, 142.1, 137.3, 128.7, 128.5, 128.2, 127.2, 119.0, 113.6, 111.8, 104.5, 90.6, 77.8, 55.0, 48.9, -0.0; **HRMS (ESI^{+}) [M+Na]$^{+}$** Calcd for $C_{20}H_{24}O_2NaSi$: 347.1438 m/z, Found: 347.1433 m/z; **Specific rotation**: $[\alpha]_D^{20}$ -26.89 (*c* 2.00, $CHCl_3$) for an enantiomerically enriched sample of 96:4 e.r.

Enantiomeric purity of **3h** was determined by HPLC analysis in comparison with authentic racemic material (96:4 e.r. shown; Chiralpak IB column, 99 : 1 *n*-hexane / *i*-PrOH, 0.8 mL/min, 220 nm).

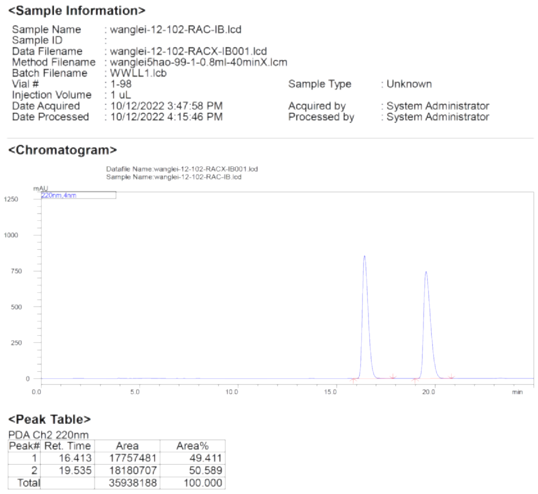

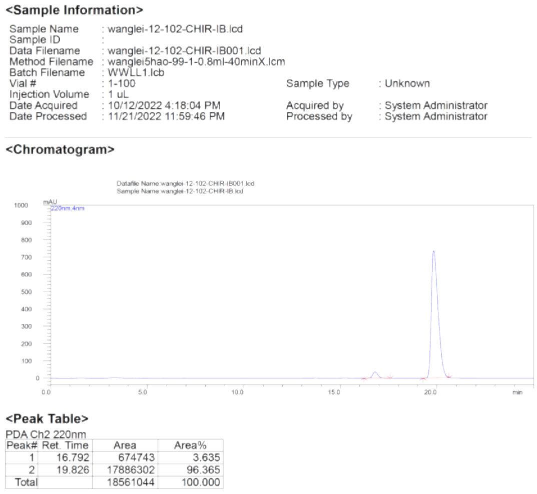

(1*R*,2*R*)-1-(4-fluorophenyl)-2-phenyl-4-(trimethylsilyl)but-3-yn-1-ol (3i)

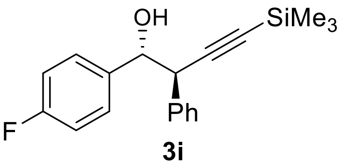

3i

Colorless oil. **IR (neat):** 3342 (w), 2176 (w), 1510 (m), 1250 (m), 1045 (m), 842 (s), 760 (m), 663 (m) cm^{-1};**^{1}H NMR (400 MHz, $CDCl_3$):** δ 7.25 – 7.17 (m, 3H), 7.15 – 7.02 (m, 4H), 6.98 – 6.76 (m, 2H), 4.70 (dd, *J* = 6.4, 3.2 Hz, 1H), 3.86 (d, *J* = 6.4 Hz, 1H), 2.83 (d, *J* = 3.2 Hz, 1H), 0.20 (s, 9H); **^{13}C NMR (100 MHz, $CDCl_3$) :** δ 162.2 (d, *J* = 245.7 Hz), 136.9, 136.2 (d, *J* = 3.2 Hz), 128.5, 128.3, 128.2, 127.3, 114.6 (d, *J* = 21.3 Hz), 104.3, 90.7, 77.3, 49.0, -0.1; **^{19}F NMR (376 MHz, $CDCl_3$):** δ -114.5; **HRMS (ESI$^+$) [M+Na]$^+$** Calcd for $C_{19}H_{21}OFNaSi$: 335.1238 m/z, Found: 335.1241 m/z; **Specific rotation**: $[\alpha]_D^{20}$ -139.71 (*c* 1.00, $CHCl_3$) for an enantiomerically enriched sample of 97:3 e.r.

Enantiomeric purity of **3i** was determined by SFC analysis in comparison with authentic racemic material (97:3 e.r. shown; Chiralpak ID column, 95:5 CO_2 / *i*-PrOH, 0.8 mL/min, 220 nm).

<Sample Information>

Sample Name : WL-12-89-RAC2-ID.lcd
Sample ID :
Data Filename : WL-12-91-RAC2-ID.lcd
Method Filename : wl-4-95-5-0.8-55xmin.lcm
Batch Filename : lwx_hydrosilylation.lcb
Vial # : 1-76 Sample Type : Unknown
Injection Volume : 2 uL
Date Acquired : 9/21/2022 12:47:27 PM Acquired by : System Administrator
Date Processed : 11/23/2022 9:27:12 PM Processed by : System Administrator

<Chromatogram>

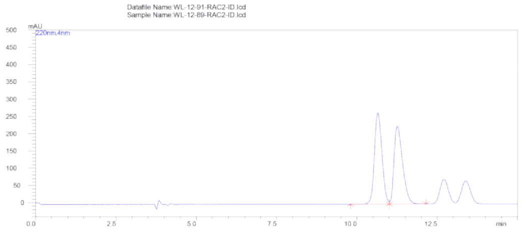

<Peak Table>

PDA Ch2 220nm

Peak#	Ret. Time	Area	Area%
1	10.651	4322587	50.954
2	11.257	4160715	49.046
Total		8483302	100.000

<Sample Information>

Sample Name : WL-12-91-CHIR-ID.lcd
Sample ID :
Data Filename : WL-12-91-CHIR-ID.lcd
Method Filename : wl-4-95-5-0.8-55xmin.lcm
Batch Filename : lwx_hydrosilylation.lcb
Vial # : 1-78 Sample Type : Unknown
Injection Volume : 2 uL
Date Acquired : 9/21/2022 3:34:22 PM Acquired by : System Administrator
Date Processed : 11/23/2022 9:28:35 PM Processed by : System Administrator

<Chromatogram>

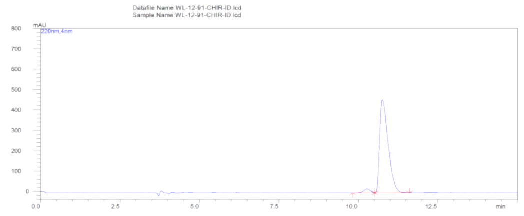

<Peak Table>

PDA Ch2 220nm

Peak#	Ret. Time	Area	Area%
1	10.264	288331	3.269
2	10.755	8532140	96.731
Total		8820471	100.000

(1*R*,2*R*)-1-(4-chlorophenyl)-2-phenyl-4-(trimethylsilyl)but-3-yn-1-ol (3j)

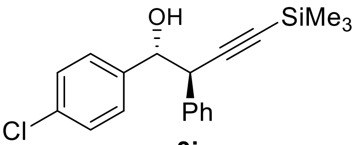

3j

Colorless oil. **IR (neat):** 3432 (w), 2174 (w), 1492 (m), 1249 (m), 1042 (m), 842 (s), 759 (m), 699 (s) cm^{-1};**^{1}H NMR (400 MHz, $CDCl_3$):** δ 7.29 – 7.18 (m, 5H), 7.18 – 7.10 (m, 2H), 7.07 (d, *J* = 8.4 Hz, 2H), 4.72 (dd, *J* = 6.8, 2.4 Hz, 1H), 3.88 (d, *J* = 6.4 Hz, 1H), 2.75 (d, *J* = 3.6 Hz, 1H), 0.20 (s, 9H); **^{13}C NMR (100 MHz, $CDCl_3$) :** δ 139.0, 136.8, 133.4, 128.5, 128.3, 128.0, 127.9, 127.4, 104.1, 91.0, 77.3, 48.9, -0.1; **HRMS (DART$^+$) [M+NH_4]$^+$** Calcd for $C_{19}H_{25}ONClSi$: 346.1388 m/z, Found: 346.1392 m/z; **Specific rotation**: $[\alpha]_D^{20}$ -29.76 (*c* 2.00, $CHCl_3$) for an enantiomerically enriched sample of 96:4 e.r.

Enantiomeric purity of **3j** was determined by HPLC analysis in comparison with authentic racemic material (96:4 e.r. shown; Chiralpak IE column, 99:1 *n*-hexane / *i*-PrOH, 0.6 mL/min, 220 nm).

<Sample Information>

Sample Name : wanglei-12-98-RACX-IE.lcd
Sample ID :
Data Filename : wanglei-12-98-RAC3X-IE.lcd
Method Filename : wanglei3hao-99-1-0.6ml-40minX.lcm
Batch Filename : WWLL1.lcb
Vial # : 1-93 Sample Type : Unknown
Injection Volume : 1 uL
Date Acquired : 10/2/2022 10:48:29 AM Acquired by : System Administrator
Date Processed : 11/21/2022 11:24:04 PM Processed by : System Administrator

<Chromatogram>

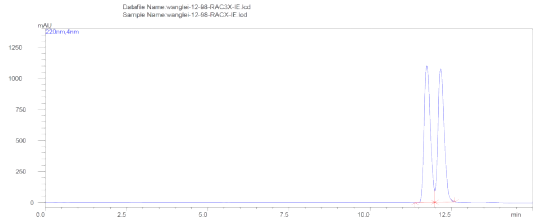

<Peak Table>

PDA Ch2 220nm

Peak#	Ret. Time	Area	Area%
1	11.745	14623402	50.581
2	12.170	14287237	49.419
Total		28910639	100.000

<Sample Information>

Sample Name : wanglei-12-98-CHIR-IE.lcd
Sample ID :
Data Filename : wanglei-12-98-CHIRX-IE001.lcd
Method Filename : wanglei1hao-99-1-0.6ml-40minX.lcm
Batch Filename : WWLL1.lcb
Vial # : 1-91 Sample Type : Unknown
Injection Volume : 0.3 uL
Date Acquired : 10/11/2022 7:12:57 PM Acquired by : System Administrator
Date Processed : 10/11/2022 7:52:59 PM Processed by : System Administrator

<Chromatogram>

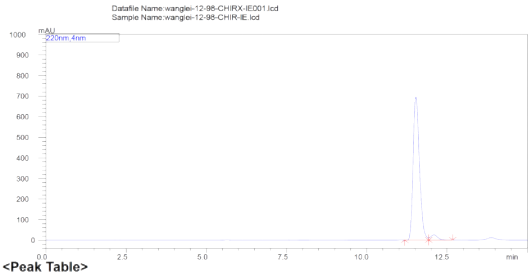

<Peak Table>

PDA Ch2 220nm

Peak#	Ret. Time	Area	Area%
1	11.527	9572377	96.202
2	12.091	377931	3.798
Total		9950308	100.000

(1*R*,2*R*)-1-(4-bromophenyl)-2-phenyl-4-(trimethylsilyl)but-3-yn-1-ol (3k)

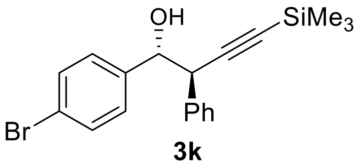

3k

White solid, m.p. 58–60 °C; **IR (neat):** 3541 (w), 2173 (w), 1251 (m), 1163 (w), 845 (s), 751 (m), 697 (s) cm^{-1};**^{1}H NMR (400 MHz, CDCl$_3$):** δ 7.35 (d, *J* = 8.4 Hz, 2H), 7.30 – 7.19 (m, 3H), 7.17 – 7.09 (m, 2H), 7.00 (d, *J* = 8.4 Hz, 2H), 4.69 (dd, *J* = 6.4, 3.6 Hz, 1H), 3.87 (d, *J* = 6.4 Hz, 1H), 2.77 (d, *J* = 3.6 Hz, 1H), 0.20 (s, 9H); **^{13}C NMR (100 MHz, CDCl$_3$) :** δ 139.5, 136.8, 130.8, 128.5, 128.4, 128.3, 127.4, 121.6, 104.0, 91.0, 77.3, 48.9, -0.0; **HRMS (ESI$^+$) [M+Na]$^+$** Calcd for $C_{19}H_{21}ONaSiBr$: 395.0437 m/z, Found: 395.0436 m/z; **Specific rotation**: $[\alpha]_D^{20}$ -45.77 (*c* 2.00, $CHCl_3$) for an enantiomerically enriched sample of 96:4 e.r.

Enantiomeric purity of **3k** was determined by SFC analysis in comparison with authentic racemic material (96:4 e.r. shown; Chiralpak ID column, 95:5 CO_2 / *i*-PrOH, 0.8 mL/min, 220 nm).

<Sample Information>

Sample Name : WL-12-92-RAC2-ID.lcd
Sample ID :
Data Filename : WL-12-92-RAC2-ID.lcd
Method Filename : wl-4-95-5-0.8-55xmin.lcm
Batch Filename : lwx_hydrosilylation.lcb
Vial # : 1-77 Sample Type : Unknown
Injection Volume : 2 uL
Date Acquired : 9/21/2022 1:38:17 PM Acquired by : System Administrator
Date Processed : 9/21/2022 2:13:20 PM Processed by : System Administrator

<Chromatogram>

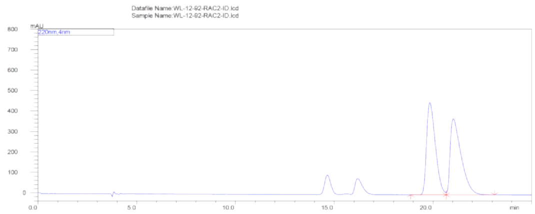

<Peak Table>

PDA Ch2 220nm

Peak#	Ret. Time	Area	Area%
1	19.844	13992913	49.366
2	21.035	14352151	50.634
Total		28345064	100.000

<Sample Information>

Sample Name : WL-12-92-CHIR-ID.lcd
Sample ID :
Data Filename : WL-12-92-CHIR-ID.lcd
Method Filename : wl-4-95-5-0.8-55xmin.lcm
Batch Filename : lwx_hydrosilylation.lcb
Vial # : 1-79 Sample Type : Unknown
Injection Volume : 2 uL
Date Acquired : 9/21/2022 2:43:35 PM Acquired by : System Administrator
Date Processed : 11/23/2022 9:38:44 PM Processed by : System Administrator

<Chromatogram>

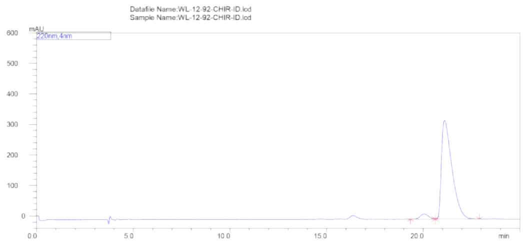

<Peak Table>

PDA Ch2 220nm

Peak#	Ret. Time	Area	Area%
1	20.045	480641	3.807
2	21.101	12144409	96.193
Total		12625051	100.000

(1*R*,2*R*)-1-(4-(methylthio)phenyl)-2-phenyl-4-(trimethylsilyl)but-3-yn-1-ol (3l)

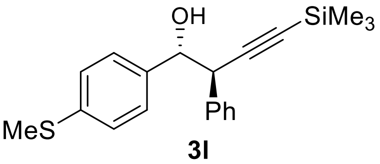

3l

White solid, m.p. 80–81 °C; **IR (neat):** 3482 (w), 2143 (w), 1494 (w), 1250 (w), 1066 (m), 842 (s), 738 (s), 701 (m) cm^{-1};**^{1}H NMR (400 MHz, CDCl$_3$):** δ 7.30 – 7.20 (m, 3H), 7.18 – 7.10 (m, 4H), 7.07 (d, *J* = 8.0 Hz, 2H), 4.71 (dd, *J* = 6.4, 3.2 Hz, 1H), 3.90 (d, *J* = 6.4 Hz,

1H), 2.73 (d, J = 3.6 Hz, 1H), 2.45 (s, 3H), 0.20 (s, 9H); **^{13}C NMR (100 MHz, CDCl$_3$) :** δ 137.8, 137.5, 137.1, 128.5, 128.2, 127.2, 127.1, 125.9, 104.4, 90.7, 77.6, 48.9, 15.8, -0.0; **HRMS (ESI$^+$) [M+Na]$^+$** Calcd for $C_{20}H_{24}ONaSiS$: 363.1209 m/z, Found: 363.1205 m/z; **Specific rotation**: $[\alpha]_D^{20}$ -63.29 (c 1.00, CHCl$_3$) for an enantiomerically enriched sample of 97:3 e.r.

Enantiomeric purity of **3l** was determined by SFC analysis in comparison with authentic racemic material (97:3 e.r. shown; Chiralpak ID column, 92:8 CO_2 / i-PrOH, 1.5 mL/min, 220 nm).

<Sample Information>

Sample Name : WL-13-18-rac-ID.lcd
Sample ID :
Data Filename : WL-13-18-rac2-ID.lcd
Method Filename : wl-2-92-8-1.5-35xmin.lcm
Batch Filename : lwx_hydrosilylation.lcb
Vial # : 1-23 Sample Type : Unknown
Injection Volume : 7 uL
Date Acquired : 10/8/2022 11:32:04 PM Acquired by : System Administrator
Date Processed : 10/10/2022 10:35:18 AM Processed by : System Administrator

<Chromatogram>

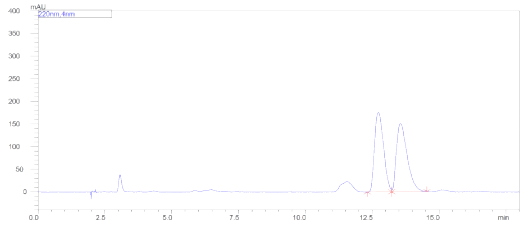

<Peak Table>

PDA Ch2 220nm

Peak#	Ret. Time	Area	Area%
1	12.733	4113992	49.314
2	13.556	4228472	50.686
Total		8342464	100.000

<Sample Information>

Sample Name : WL-13-18-CHIR-ID.lcd
Sample ID :
Data Filename : WL-13-18-CHIR-ID.lcd
Method Filename : wl-2-92-8-1.5-35xmin.lcm
Batch Filename : lwx_hydrosilylation.lcb
Vial # : 1-26 Sample Type : Unknown
Injection Volume : 1 uL
Date Acquired : 10/9/2022 2:09:03 AM Acquired by : System Administrator
Date Processed : 10/9/2022 8:51:51 AM Processed by : System Administrator

<Chromatogram>

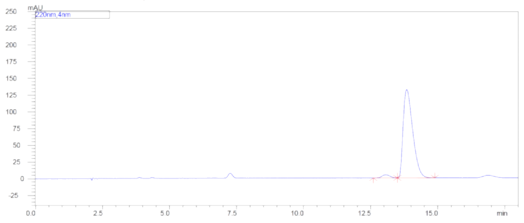

<Peak Table>

PDA Ch2 220nm

Peak#	Ret. Time	Area	Area%
1	13.052	97932	2.910
2	13.845	3266971	97.090
Total		3364903	100.000

(1*R*,2*R*)-2-phenyl-1-(4-(trifluoromethoxy)phenyl)-4-(trimethylsilyl)but-3-yn-1-ol (3m)

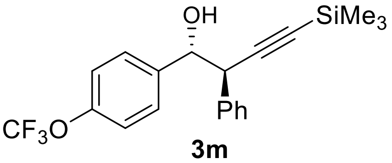

3m

White solid, m.p. 55–57 °C; **IR (neat):** 3335 (w), 2176 (w), 1509 (w), 1285 (s), 1251 (s)，894 (s), 761 (s), 699 (m) cm^{-1};**^{1}H NMR (400 MHz, CDCl$_3$):** δ 7.30 – 7.22 (m, 3H), 7.18 (d, J = 8.8 Hz, 2H), 7.16 – 7.11 (m, 2H), 7.09 (d, J = 8.4 Hz, 2H), 4.77 (dd, J = 6.8, 3.2 Hz, 1H), 3.90 (d, J = 6.4 Hz, 1H), 2.75 (d, J = 3.2 Hz, 1H), 0.20 (s, 9H); **^{13}C NMR (100 MHz, CDCl$_3$) :** δ 148.7, 139.2, 136.8, 128.5, 128.4, 128.0, 127.5, 120.4 (q, J = 256 Hz), 120.3, 103.9, 91.1, 77.3, 49.0, -0.1; **^{19}F NMR (376 MHz, CDCl$_3$):** δ -57.9; **HRMS (DART$^+$) [M+H]$^+$** Calcd for $C_{20}H_{22}O_2F_3Si$: 379.1336 m/z, Found: 379.1342 m/z; **Specific rotation**: $[\alpha]_D^{20}$ -16.28 (c 2.00, CHCl$_3$) for an enantiomerically enriched sample of 96:4 e.r.

Enantiomeric purity of **3m** was determined by SFC analysis in comparison with authentic racemic material (96:4 e.r. shown; Chiralpak IA column, 96:4 CO_2 / i-PrOH, 0.8 mL/min, 220 nm).

<Sample Information>

Sample Name : WL-13-17RACc-IA.lcd
Sample ID :
Data Filename : WL-13-17RACc-IA.lcd
Method Filename : wl-1-96-4-0.8-35xmin.lcm
Batch Filename : lwx_hydrosilylation.lcb
Vial # : 1-22
Injection Volume : 3 uL
Date Acquired : 10/9/2022 1:36:58 PM
Date Processed : 10/9/2022 2:12:01 PM
Sample Type : Unknown
Acquired by : System Administrator
Processed by : System Administrator

<Chromatogram>

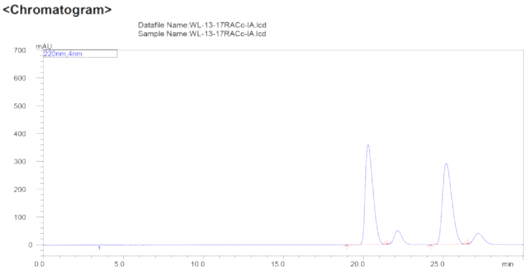

<Peak Table>

PDA Ch2 220nm

Peak#	Ret. Time	Area	Area%
1	20.300	12033628	50.106
2	25.184	11982879	49.894
Total		24016506	100.000

<Sample Information>

Sample Name : WL-13-17CHIR-IA.lcd
Sample ID :
Data Filename : WL-13-17CHIR-IA.lcd
Method Filename : wl-1-96-4-0.8-35xmin.lcm
Batch Filename : lwx_hydrosilylation.lcb
Vial # : 1-25
Injection Volume : 2 uL
Date Acquired : 10/9/2022 2:12:49 PM
Date Processed : 10/10/2022 10:35:30 AM
Sample Type : Unknown
Acquired by : System Administrator
Processed by : System Administrator

<Chromatogram>

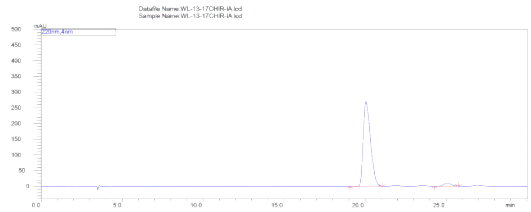

<Peak Table>

PDA Ch2 220nm

Peak#	Ret. Time	Area	Area%
1	20.055	8519844	96.101
2	25.055	345674	3.899
Total		8865517	100.000

(1*R*,2*R*)-2-phenyl-1-(4-(4,4,5,5-tetramethyl-1,3,2-dioxaborolan-2-yl)phenyl)-4-(trimethylsilyl)but-3-yn-1-ol (3n)

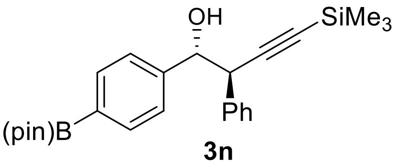

3n

White solid, m.p. 100–102 ºC; **IR (neat):** 2175 (w), 1509 (w), 1287 (m), 1252 (m), 840 (s), 760 (m) cm^{-1}; **^{1}H NMR (400 MHz, $CDCl_3$):** δ 7.69 (d, *J* = 8.0 Hz, 2H), 7.29 – 7.20 (m, 3H), 7.20 – 7.10 (m, 4H), 4.75 (dd, *J* = 6.4, 4.0 Hz, 1H), 3.94 (d, *J* = 6.4 Hz, 1H), 2.77 (d, *J* = 4.0 Hz, 1H), 1.33 (s, 12H), 0.20 (s, 9H); **^{13}C NMR (100 MHz, $CDCl_3$) :** δ 143.7, 137.2, 134.3, 128.5, 128.3, 127.3, 126.0, 104.2, 90.9, 83.7, 77.9, 48.9, 24.8, -0.0; **HRMS (ESI^{+}) [M+Na]$^{+}$** Calcd for $C_{25}H_{33}BO_3NaSi$: 443.2184 m/z, Found: 443.2190 m/z; **Specific rotation**: $[\alpha]_D^{20}$ -20.55 (*c* 1.00, $CHCl_3$) for an enantiomerically enriched sample of 98:2 e.r.

Enantiomeric purity of **3n** was determined by HPLC analysis in comparison with authentic racemic material (98:2 e.r. shown; Chiralpak IF column, 99:1 *n*-hexane / *i*-PrOH, 0.8 mL/min, 220 nm).

<Sample Information>

Sample Name : wanglei-12-101-RAC-IF.lcd
Sample ID :
Data Filename : wanglei-12-101-RAC2-IF.lcd
Method Filename : wanglei5hao-99-1-0.8ml-60minX.lcm
Batch Filename : WWLL1.lcb
Vial # : 1-94
Injection Volume : 1 uL
Date Acquired : 10/2/2022 2:01:39 PM
Date Processed : 11/21/2022 11:51:06 PM
Sample Type : Unknown
Acquired by : System Administrator
Processed by : System Administrator

<Chromatogram>

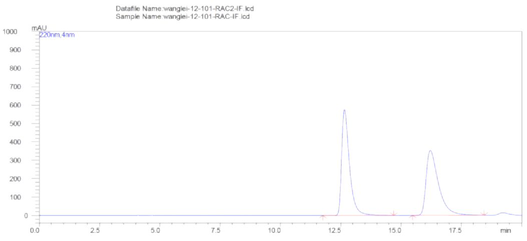

<Peak Table>

PDA Ch2 220nm

Peak#	Ret. Time	Area	Area%
1	12.655	13003265	50.747
2	16.213	12620558	49.253
Total		25623823	100.000

<Sample Information>

Sample Name : wanglei-12-101-CHIR-IF.lcd
Sample ID :
Data Filename : wanglei-12-101-CHIR-IF.lcd
Method Filename : wanglei5hao-99-1-0.8ml-60minX.lcm
Batch Filename : WWLL1.lcb
Vial # : 1-96
Injection Volume : 1 uL
Date Acquired : 10/2/2022 2:27:59 PM
Date Processed : 10/3/2022 6:10:21 PM
Sample Type : Unknown
Acquired by : System Administrator
Processed by : System Administrator

<Chromatogram>

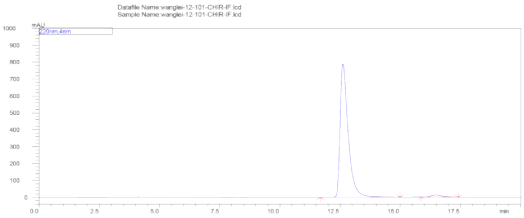

<Peak Table>

PDA Ch2 220nm

Peak#	Ret. Time	Area	Area%
1	12.620	18473250	97.992
2	16.469	378469	2.008
Total		18851719	100.000

(1*R*,2*R*)-2-phenyl-1-(*o*-tolyl)-4-(trimethylsilyl)but-3-yn-1-ol (3o)

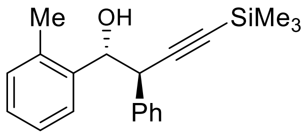

3o

White solid, m.p. 49–51 °C; **IR (neat):** 3335 (w), 2175 (w), 1493 (m), 1249 (s), 1031 (m), 842 (s), 760 (m) cm^{-1}; **^{1}H NMR (400 MHz, $CDCl_3$):** δ 7.55 (dd, *J* = 7.6, 1.6 Hz, 1H), 7.26 – 7.19 (m, 4H), 7.18 – 7.11 (m, 3H), 6.97 (d, *J* = 7.6 Hz, 1H), 4.94 (dd, *J* = 6.4, 3.6 Hz, 1H), 3.95 (d, *J* = 6.4 Hz, 1H), 2.78 (d, *J* = 4.0 Hz, 1H), 1.90 (s, 3H), 0.22 (s, 9H); **^{13}C NMR (100 MHz, $CDCl_3$) :** δ 138.9, 137.3, 135.2, 129.8, 128.3, 128.2, 127.5, 127.2, 126.3, 125.8, 104.4, 90.8, 73.7, 48.3, 18.8, -0.0; **HRMS (ESI$^+$) [M+Na]$^+$** Calcd for $C_{20}H_{24}ONaSi$: 331.1489 m/z, Found: 331.1485 m/z; **Specific rotation**: $[\alpha]_D^{20}$ -7.46 (*c* 2.00, $CHCl_3$) for an enantiomerically enriched sample of 95:5 e.r.

Enantiomeric purity of **3o** was determined by SFC analysis in comparison with authentic racemic material (95:5 e.r. shown; Chiralpak IB column, 97:3 CO_2 / *i*-PrOH, 0.8 mL/min, 220 nm).

<Sample Information>

Sample Name : WL-12-94-RAC2-IB.lcd
Sample ID :
Data Filename : WL-12-94-RAC-IB.lcd
Method Filename : wl-2-97-3-0.8-35xmin.lcm
Batch Filename : lwx_hydrosilylation.lcb
Vial # : 1-76 Sample Type : Unknown
Injection Volume : 2 uL
Date Acquired : 9/21/2022 8:12:27 PM Acquired by : System Administrator
Date Processed : 11/23/2022 9:51:27 PM Processed by : System Administrator

<Chromatogram>

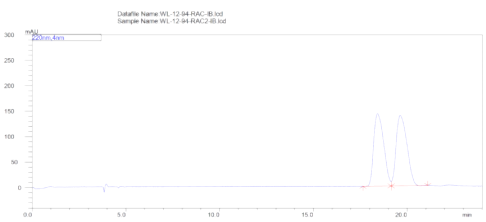

<Peak Table>

PDA Ch2 220nm

Peak#	Ret. Time	Area	Area%
1	18.418	5184288	49.334
2	19.627	5324226	50.666
Total		10508513	100.000

<Sample Information>

Sample Name : WL-12-94-CHIR-IB.lcd
Sample ID :
Data Filename : WL-12-94-CHIR-IB.lcd
Method Filename : wl-2-97-3-0.8-35xmin.lcm
Batch Filename : lwx_hydrosilylation.lcb
Vial # : 1-79 Sample Type : Unknown
Injection Volume : 2 uL
Date Acquired : 9/21/2022 8:55:30 PM Acquired by : System Administrator
Date Processed : 11/23/2022 9:53:06 PM Processed by : System Administrator

<Chromatogram>

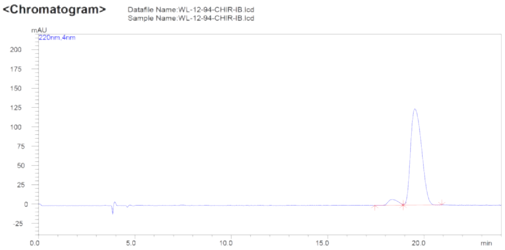

<Peak Table>

PDA Ch2 220nm

Peak#	Ret. Time	Area	Area%
1	18.357	263084	5.053
2	19.518	4943066	94.947
Total		5206150	100.000

(1*R*,2*R*)-1-(naphthalen-1-yl)-2-phenyl-4-(trimethylsilyl)but-3-yn-1-ol (3p)

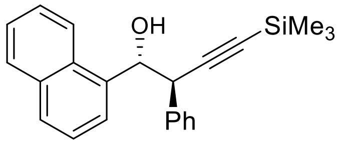

3p

White solid, m.p. 103–105 °C; **IR (neat):** 3567 (w), 2165 (w), 1454 (w), 1247 (m), 1026 (m), 841 (s), 760 (m), 700 (m) cm^{-1}; **^{1}H NMR (400 MHz, $CDCl_3$):** δ 8.09 – 7.97 (m, 1H), 7.92 – 7.82 (m, 1H), 7.78 (d, *J* = 8.0 Hz, 1H), 7.60 (d, *J* = 7.2 Hz, 1H), 7.51 – 7.39 (m, 3H), 7.37 – 7.28 (m, 2H), 7.31 – 7.18 (m, 3H), 5.53 (t, *J* = 4.4 Hz, 1H), 4.29 (d, *J* = 4.8 Hz, 1H), 2.82 (d, *J* = 4.4 Hz, 1H), 0.19 (s, 9H); **^{13}C NMR (100 MHz, $CDCl_3$) :** δ 138.1, 136.4, 133.6, 130.3, 128.9, 128.4, 128.3, 128.1, 127.3, 126.0, 125.2, 125.0, 124.7, 122.7, 103.8, 91.4, 74.6, 47.6, -0.0; **HRMS (ESI$^+$) [M+Na]$^+$** Calcd for $C_{23}H_{24}ONaSi$: 367.1489 m/z, Found: 367.1495 m/z; **Specific rotation**: $[\alpha]_D^{20}$ 10.75 (*c* 1.00, $CHCl_3$) for an enantiomerically enriched sample of 96:4 e.r.

Enantiomeric purity of **3p** was determined by HPLC analysis in comparison with authentic racemic material (96:4 e.r. shown; Chiralpak ID column, 99:1 *n*-hexane / *i*-PrOH, 0.6 mL/min, 220 nm).

<Sample Information>

Sample Name : wanglei-12-105-RAC-ID.lcd
Sample ID :
Data Filename : wanglei-12-105-RACX-ID.lcd
Method Filename : wanglei1hao-99-1-0.6ml-40minX.lcm
Batch Filename : WWLL1.lcb
Vial # : 1-79 Sample Type : Unknown
Injection Volume : 0.5 uL
Date Acquired : 10/3/2022 9:01:36 PM Acquired by : System Administrator
Date Processed : 11/22/2022 12:47:24 AM Processed by : System Administrator

<Chromatogram>

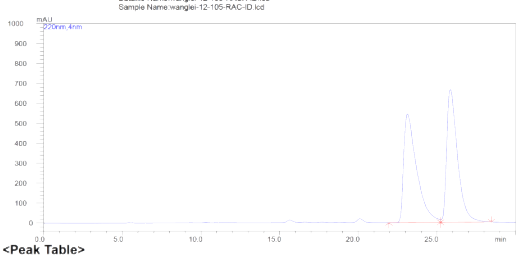

<Peak Table>

PDA Ch2 220nm

Peak#	Ret. Time	Area	Area%
1	23.125	31388002	49.629
2	25.849	31857217	50.371
Total		63245219	100.000

<Sample Information>

Sample Name : wanglei-12-105-CHIR-ID.lcd
Sample ID :
Data Filename : wanglei-12-105-CHIR-ID.lcd
Method Filename : wanglei1hao-99-1-0.6ml-40minX.lcm
Batch Filename : hw.lcb
Vial # : 1-81 Sample Type : Unknown
Injection Volume : 1 uL
Date Acquired : 10/4/2022 12:53:16 PM Acquired by : System Administrator
Date Processed : 11/22/2022 12:45:41 AM Processed by : System Administrator

<Chromatogram>

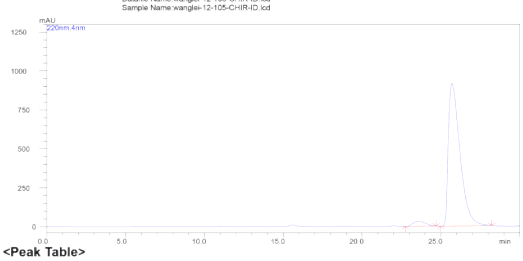

<Peak Table>

PDA Ch2 220nm

Peak#	Ret. Time	Area	Area%
1	23.580	1809239	3.722
2	25.701	46799014	96.278
Total		48608252	100.000

(1*R*,2*R*)-1-(naphthalen-2-yl)-2-phenyl-4-(trimethylsilyl)but-3-yn-1-ol (3q)

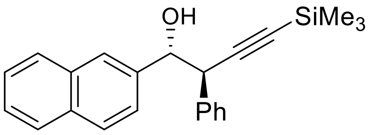

3q

White solid, m.p. 87–88 °C; **IR (neat):** 3541 (w), 2173 (w), 1252 (m), 1027 (m), 835 (s), 748 (m), 696 (m) cm^{-1}; **^{1}H NMR (400 MHz, $CDCl_3$):** δ 7.84 – 7.77 (m, 1H), 7.73 (d, *J* = 8.0 Hz, 2H), 7.61 (s, 1H), 7.51 – 7.40 (m, 2H), 7.31 (d, *J* = 8.4 Hz, 1H), 7.25 – 7.20 (m, 3H), 7.19 – 7.14 (m, 2H), 4.92 (d, *J* = 6.4 Hz, 1H), 4.05 (d, *J* = 6.4 Hz, 1H), 2.85 (s, 1H), 0.20 (s, 9H); **^{13}C NMR (100 MHz, $CDCl_3$) :** δ 138.0, 137.2, 133.0, 132.8, 128.5, 128.2, 128.0, 127.5, 127.4, 127.2, 125.8, 125.7, 124.6, 104.4, 90.8, 77.9, 48.8, -0.0; **HRMS (ESI^+) $[M+Na]^+$** Calcd for $C_{23}H_{24}ONaSi$: 367.1489 m/z, Found: 367.1488 m/z; **Specific rotation**: $[\alpha]_D^{20}$ -32.44 (*c* 2.00, $CHCl_3$) for an enantiomerically enriched sample of 97:3 e.r.

Enantiomeric purity of **3q** was determined by SFC analysis in comparison with authentic racemic material (97:3 e.r. shown; Chiralpak ID column, 88:12 CO_2 / *i*-PrOH, 0.8 mL/min, 220 nm).

<Sample Information>

Sample Name : WL-12-104rac-ID.lcd
Sample ID :
Data Filename : WL-12-104-rac3X-ID.lcd
Method Filename : wl-2-88-12-0.8-35xmin.lcm
Batch Filename : lwx_hydrosilylation.lcb
Vial # : 1-93 Sample Type : Unknown
Injection Volume : 1 uL
Date Acquired : 10/14/2022 4:52:12 AM Acquired by : System Administrator
Date Processed : 11/24/2022 12:14:14 AM Processed by : System Administrator

<Chromatogram>

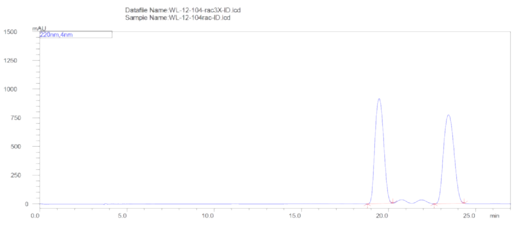

<Peak Table>

PDA Ch2 220nm

Peak#	Ret. Time	Area	Area%
1	19.462	31779154	49.482
2	23.445	32444145	50.518
Total		64223299	100.000

<Sample Information>

Sample Name : WL-12-104-CHIR-ID.lcd
Sample ID :
Data Filename : WL-12-104-CHIR-ID001.lcd
Method Filename : wl-2-88-12-0.8-35xmin.lcm
Batch Filename : lwx_hydrosilylation.lcb
Vial # : 1-92 Sample Type : Unknown
Injection Volume : 1 uL
Date Acquired : 10/14/2022 11:00:31 AM Acquired by : System Administrator
Date Processed : 10/14/2022 11:47:38 AM Processed by : System Administrator

<Chromatogram>

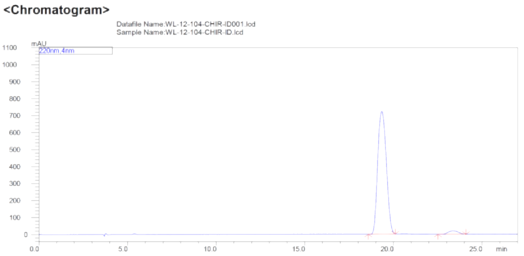

<Peak Table>

PDA Ch2 220nm

Peak#	Ret. Time	Area	Area%
1	19.344	24474769	96.821
2	23.360	803723	3.179
Total		25278492	100.000

(1*R*,2*R*)-1-(furan-2-yl)-2-phenyl-4-(trimethylsilyl)but-3-yn-1-ol (3r)

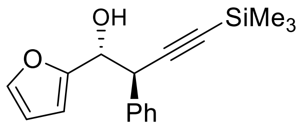

3r

Colorless oil. **IR (neat):** 3518 (w), 2179 (w), 1494 (w), 1252 (m), 1039 (m), 844 (s), 742 (m), 699 (m) cm^{-1}; **^{1}H NMR (400 MHz, CDCl$_3$):** δ 7.34 (s, 1H), 7.29 – 7.16 (m, 5H), 6.25 (dd, *J* = 3.2, 1.6 Hz, 1H), 6.16 (d, *J* = 3.2 Hz, 1H), 4.74 (t, *J* = 6.0 Hz, 1H), 4.22 (d, *J* = 6.4 Hz, 1H), 2.76 (d, *J* = 6.0 Hz, 1H), 0.19 (s, 9H); **^{13}C NMR (100 MHz, CDCl$_3$) :** δ 153.1, 141.8, 137.2, 128.3, 128.1, 127.3, 110.1, 107.8, 103.9, 90.7, 72.0, 45.9, -0.1; **HRMS (ESI$^+$) [M+Na]$^+$** Calcd for $C_{17}H_{20}O_2NaSi$: 307.1125 m/z, Found: 307.1132 m/z; **Specific rotation**: $[\alpha]_D^{20}$ -0.29 (*c* 1.00, CHCl$_3$) for an enantiomerically enriched sample of 94:6 e.r.

Enantiomeric purity of **3r** was determined by SFC analysis in comparison with authentic racemic material (94:6 e.r. shown; Chiralpak IB column, 95:5 CO_2 / *i*-PrOH, 0.8 mL/min, 220 nm).

<Sample Information>

Sample Name : WL-12-88-RAC2-IB.lcd
Sample ID :
Data Filename : WL-12-88-RA2-IB.lcd
Method Filename : wl-2-95-5-0.8-35xmin.lcm
Batch Filename : lwx_hydrosilylation.lcb
Vial # : 1-17 Sample Type : Unknown
Injection Volume : 2 uL
Date Acquired : 9/16/2022 7:52:46 PM Acquired by : System Administrator
Date Processed : 11/23/2022 9:06:36 PM Processed by : System Administrator

<Chromatogram>

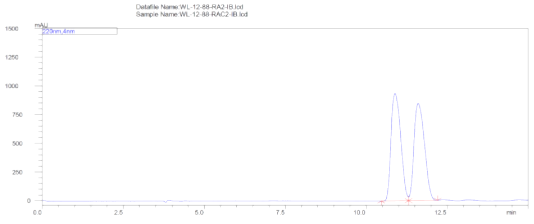

<Peak Table>

PDA Ch2 220nm

Peak#	Ret. Time	Area	Area%
1	10.887	19415185	50.573
2	11.603	18975498	49.427
Total		38390683	100.000

<Sample Information>

Sample Name : WL-12-88-CHIR-IB.lcd
Sample ID :
Data Filename : WL-12-88-CHIRX-IB.lcd
Method Filename : wl-2-95-5-0.8-35xmin.lcm
Batch Filename : lwx_hydrosilylation.lcb
Vial # : 1-76 Sample Type : Unknown
Injection Volume : 2 uL
Date Acquired : 9/17/2022 9:54:05 AM Acquired by : System Administrator
Date Processed : 11/23/2022 9:09:13 PM Processed by : System Administrator

<Chromatogram>

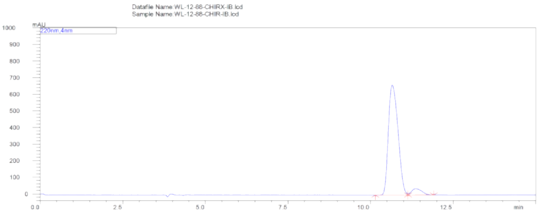

<Peak Table>

PDA Ch2 220nm

Peak#	Ret. Time	Area	Area%
1	10.658	13483304	94.231
2	11.368	825462	5.769
Total		14308766	100.000

(1*R*,2*R*)-1-(furan-3-yl)-2-phenyl-4-(trimethylsilyl)but-3-yn-1-ol (3s)

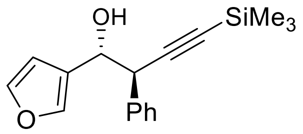

3s

Colorless oil. **IR (neat):** 3380 (w), 2174 (w), 1495 (m), 1250 (m), 1021 (m), 842 (s), 760 (m), 747 (m) cm^{-1}; **^{1}H NMR (400 MHz, CDCl$_3$):** δ 7.35 – 7.29 (m, 1H), 7.29 – 7.22 (m, 5H), 7.19 (s, 1H), 6.27 (s, 1H), 4.74 (d, *J* = 4.8 Hz, 1H), 3.92 (dd, *J* = 6.0, 1.6 Hz, 1H), 2.62 (s, 1H), 0.20 (s, 9H); **^{13}C NMR (100 MHz, CDCl$_3$) :** δ 142.7, 139.9, 137.3, 128.5, 128.3, 127.4, 125.6, 108.8, 104.5, 90.5, 71.1, 47.7, -0.1; **HRMS (ESI$^+$) [M+Na]$^+$** Calcd for $C_{17}H_{20}O_2NaSi$: 307.1125 m/z, Found: 307.1133 m/z; **Specific rotation**: $[\alpha]_D^{20}$ 4.57 (*c* 2.00, CHCl$_3$) for an enantiomerically enriched sample of 95:5 e.r.

Enantiomeric purity of **3s** was determined by SFC analysis in comparison with authentic racemic material (95:5 e.r. shown; Chiralcel OZ-H column, 98:2 CO_2 / *i*-PrOH, 0.8 mL/min, 220 nm).

<Sample Information>

Sample Name : WL-12-89-RAC2-OZH.lcd
Sample ID :
Data Filename : WL-12-89-RAC-OZH.lcd
Method Filename : wl-5-98-2-0.8-55xmin.lcm
Batch Filename : lwx_hydrosilylation.lcb
Vial # : 1-18 Sample Type : Unknown
Injection Volume : 2 uL
Date Acquired : 9/16/2022 11:27:01 PM Acquired by : System Administrator
Date Processed : 9/17/2022 12:02:05 AM Processed by : System Administrator

<Chromatogram>

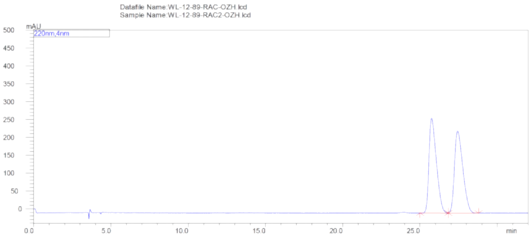

<Peak Table>

PDA Ch2 220nm

Peak#	Ret. Time	Area	Area%
1	25.750	8840599	50.471
2	27.431	8675423	49.529
Total		17516022	100.000

<Sample Information>

Sample Name : WL-12-89-CHIR-OZH.lcd
Sample ID :
Data Filename : WL-12-89-CHIRX-OZH.lcd
Method Filename : wl-5-98-2-0.8-55xmin.lcm
Batch Filename : lwx_hydrosilylation.lcb
Vial # : 1-77 Sample Type : Unknown
Injection Volume : 14 uL
Date Acquired : 9/17/2022 1:41:22 AM Acquired by : System Administrator
Date Processed : 11/23/2022 9:20:47 PM Processed by : System Administrator

<Chromatogram>

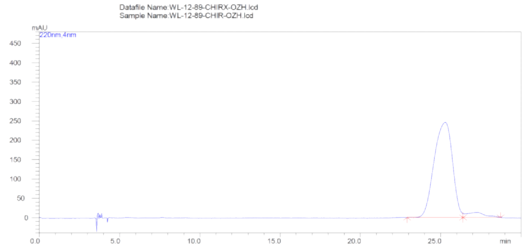

<Peak Table>

PDA Ch2 220nm

Peak#	Ret. Time	Area	Area%
1	25.301	19578696	94.848
2	27.264	1063563	5.152
Total		20642259	100.000

(1*R*,2*R*)-2-phenyl-1-(thiophen-2-yl)-4-(trimethylsilyl)but-3-yn-1-ol (3t)

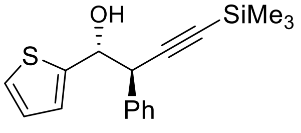

3t

Colorless oil. **IR (neat):** 3442 (w), 2175 (w), 1494 (m), 1250 (m), 1037 (m), 844 (s), 761 (m), 700 (m) cm^{-1}; **^{1}H NMR (400 MHz, $CDCl_3$):** δ 7.37 – 7.07 (m, 6H), 7.00 – 6.79 (m, 1H), 6.68 (d, *J* = 2.0 Hz, 1H), 4.99 (t, *J* = 5.6 Hz, 1H), 4.00 (d, *J* = 6.0 Hz, 1H), 2.92 (d, *J* = 4.4 Hz, 1H), 0.20 (s, 9H); **^{13}C NMR (100 MHz, $CDCl_3$) :** δ 144.2, 137.1, 128.4, 128.3, 127.4, 126.1, 125.0, 124.7, 104.0, 91.2, 74.4, 49.1, -0.1; **HRMS (ESI^{+}) [M+Na]$^{+}$** Calcd for $C_{17}H_{20}ONaSiS$: 323.0896 m/z, Found: 323.0902 m/z; **Specific rotation**: $[\alpha]_D^{20}$ -21.04 (*c* 2.00, $CHCl_3$) for an enantiomerically enriched sample of 95:5 e.r.

Enantiomeric purity of **3t** was determined by SFC analysis in comparison with authentic racemic material (95:5 e.r. shown; Chiralcel OZ-H column, 97:3 CO_2 / *i*-PrOH, 0.8 mL/min, 220 nm).

<Sample Information>

Sample Name : WL-12-85-RAC-OZH.lcd
Sample ID :
Data Filename : WL-12-85-RAC-OZH.lcd
Method Filename : wl-5-97-3-0.8-55xmin.lcm
Batch Filename : lwx_hydrosilylation.lcb
Vial # : 1-19 Sample Type : Unknown
Injection Volume : 2 uL
Date Acquired : 9/15/2022 11:27:46 PM Acquired by : System Administrator
Date Processed : 11/23/2022 8:34:15 PM Processed by : System Administrator

<Chromatogram>

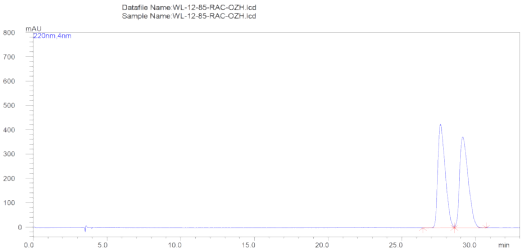

<Peak Table>

PDA Ch2 220nm

Peak#	Ret. Time	Area	Area%
1	27.620	14817762	49.576
2	29.131	15071409	50.424
Total		29889172	100.000

<Sample Information>

Sample Name : WL-12-85-CHIR-OZH.lcd
Sample ID :
Data Filename : WL-12-85-CHIR-OZH.lcd
Method Filename : wl-5-97-3-0.8-55xmin.lcm
Batch Filename : lwx_hydrosilylation.lcb
Vial # : 1-16 Sample Type : Unknown
Injection Volume : 1 uL
Date Acquired : 9/16/2022 12:34:13 AM Acquired by : System Administrator
Date Processed : 11/23/2022 8:35:45 PM Processed by : System Administrator

<Chromatogram>

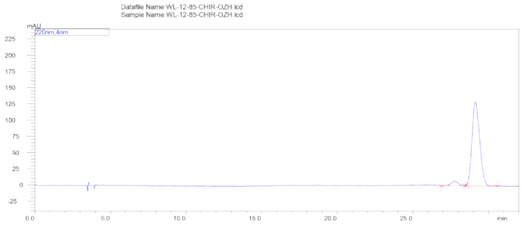

<Peak Table>

PDA Ch2 220nm

Peak#	Ret. Time	Area	Area%
1	27.735	225039	4.683
2	29.129	4580368	95.317
Total		4805407	100.000

(1*R*,2*R*)-2-phenyl-1-(thiophen-2-yl)-4-(trimethylsilyl)but-3-yn-1-ol (3u)

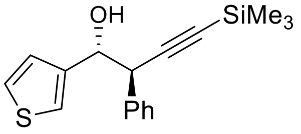

3u

Colorless oil. **IR (neat):** 3491 (w), 2174 (w), 1664 (m), 1250 (m), 1040 (m), 842 (s), 759 (m), 698 (m) cm^{-1}; **^{1}H NMR (400 MHz, $CDCl_3$):** δ 7.30 – 7.20 (m, 3H), 7.23 – 7.14 (m, 3H), 7.01 – 6.97 (m, 1H), 6.94 – 6.88 (m, 1H), 4.84 (dd, *J* = 6.4, 3.2 Hz, 1H), 3.95 (dd, *J* = 6.4, 1.6 Hz, 1H), 2.73 (d, *J* = 2.8

Hz, 1H), 0.20 (d, *J* = 1.6 Hz, 9H); **^{13}C NMR (100 MHz, $CDCl_3$) :** δ 142.1, 137.3, 128.4, 128.2, 127.3, 126.0, 125.1, 122.0, 104.5, 90.6, 74.4, 48.3, -0.0; **HRMS (ESI$^+$) [M+Na]$^+$** Calcd for $C_{17}H_{20}ONaSiS$: 323.0896 m/z, Found: 323.0901 m/z; **Specific rotation**: $[\alpha]_D^{20}$ -8.24 (*c* 2.00, $CHCl_3$) for an enantiomerically enriched sample of 96:4 e.r.

Enantiomeric purity of **3u** was determined by SFC analysis in comparison with authentic racemic material (96:4 e.r. shown; Chiralpak IF column, 95:5 CO_2 / *i*-PrOH, 0.8 mL/min, 220 nm).

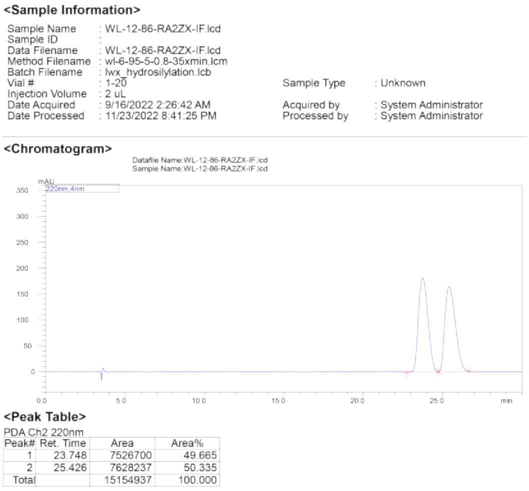

Peak#	Ret. Time	Area	Area%
1	23.748	7526700	49.665
2	25.426	7628237	50.335
Total		15154937	100.000

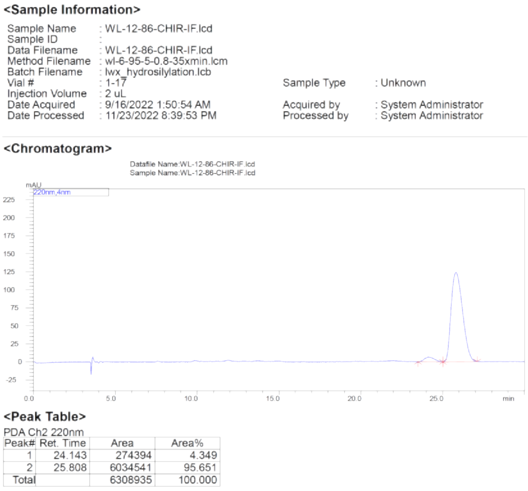

Peak#	Ret. Time	Area	Area%
1	24.143	274394	4.349
2	25.808	6034541	95.651
Total		6308935	100.000

(1*R*,2*R*)-2-phenyl-1-(pyridin-3-yl)-4-(trimethylsilyl)but-3-yn-1-ol (3v)

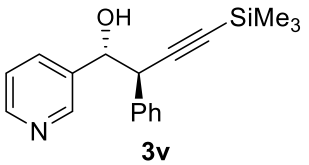

3v

Colorless oil. **IR (neat):** 3425 (w), 2175 (w), 1427 (m), 1249 (m), 1028 (m), 840 (s), 759 (m), 699 (m) cm^{-1}; **^{1}H NMR (400 MHz, $CDCl_3$):** δ 8.43 (dd, *J* = 4.8, 1.6 Hz, 1H), 8.29 (d, *J* = 2.0 Hz, 1H), 7.56 – 7.49 (m, 1H), 7.30 – 7.23 (m, 3H), 7.21 – 7.11 (m, 3H), 4.83 (d, *J* = 6.4 Hz, 1H), 3.94 (d, *J* = 6.4 Hz, 1H), 3.32 (s, 1H), 0.20 (s, 9H); **^{13}C NMR (100 MHz, $CDCl_3$) :** δ 148.5, 148.0, 136.5, 134.6, 128.6, 128.2, 127.4, 122.7, 104.0, 90.8, 75.3, 48.4, -0.1; **HRMS (ESI$^+$) [M+H]$^+$** Calcd for $C_{18}H_{22}NOSi$: 296.1465 m/z, Found: 296.1462 m/z; **Specific rotation**: $[\alpha]_D^{20}$ -8.68 (*c* 1.00, $CHCl_3$) for an enantiomerically enriched sample of 96:4 e.r.

Enantiomeric purity of **3v** was determined by HPLC analysis in comparison with authentic racemic material (96:4 e.r. shown; Chiralpak IG column, 93:7 *n*-hexane / *i*-PrOH, 1.0 mL/min, 220 nm).

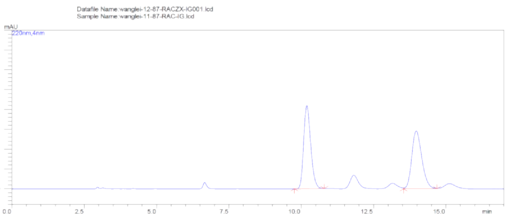

<Sample Information>

Sample Name : wanglei-11-87-RAC-IG.lcd
Sample ID :
Data Filename : wanglei-12-87-RACZX-IG001.lcd
Method Filename : wanglei6hao-93-7-1.0ml-40minX.lcm
Batch Filename : WWLL1.lcb
Vial # : 1-95 Sample Type : Unknown
Injection Volume : 2 uL
Date Acquired : 10/12/2022 1:47:59 AM Acquired by : System Administrator
Date Processed : 11/21/2022 11:14:53 PM Processed by : System Administrator

<Chromatogram>

<Peak Table>

PDA Ch2 220nm

Peak#	Ret. Time	Area	Area%
1	10.192	3572290	50.295
2	13.974	3530408	49.705
Total		7102698	100.000

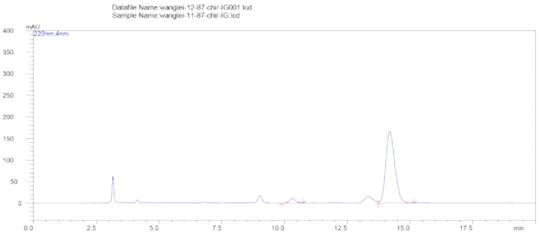

<Sample Information>

Sample Name : wanglei-11-87-chir-IG.lcd
Sample ID :
Data Filename : wanglei-12-87-chir-IG001.lcd
Method Filename : wanglei6hao-93-7-1.0ml-40minX.lcm
Batch Filename : WWLL1.lcb
Vial # : 1-94 Sample Type : Unknown
Injection Volume : 2 uL
Date Acquired : 10/10/2022 6:10:19 PM Acquired by : System Administrator
Date Processed : 11/21/2022 11:19:13 PM Processed by : System Administrator

<Chromatogram>

<Peak Table>

PDA Ch2 220nm

Peak#	Ret. Time	Area	Area%
1	10.307	180609	4.168
2	14.190	4152326	95.832
Total		4332935	100.000

***tert*-butyl 2-((1*R*,2*R*)-1-hydroxy-2-phenyl-4-(trimethylsilyl)but-3-yn-1-yl)-1H-indole-1-carboxylate (3w)**

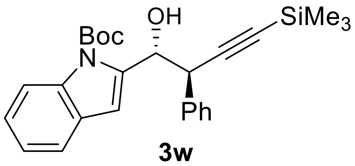

3w

Colorless oil. **IR (neat):** 3468 (w), 2175 (w), 1724 (s), 1453 (m), 1328 (s), 1157 (m), 840 (s), 761 (m), 698 (m) cm^{-1}; **^{1}H NMR (400 MHz, $CDCl_3$):** δ 7.98 (d, *J* = 8.4 Hz, 1H), 7.55 (d, *J* = 6.4 Hz, 2H), 7.51 (d, *J* = 7.2 Hz, 1H), 7.34 (t, *J* = 7.6 Hz, 2H), 7.31 – 7.16 (m, 3H), 6.77 (s, 1H), 5.55 – 5.51 (m, 1H), 4.44 (d, *J* = 3.6 Hz, 1H), 3.16 (d, *J* = 8.4 Hz, 1H), 1.72 (s, 9H), 0.16 (s, 9H); **^{13}C NMR (100 MHz, $CDCl_3$) :** δ 151.0, 141.9, 138.6, 136.2, 129.0, 128.4, 128.1, 127.2, 124.0, 122.9, 120.7, 115.7, 109.2, 103.5, 91.0, 84.7, 72.2, 45.6, 28.2, 0.0; **HRMS (ESI$^+$) [M+Na]$^+$** Calcd for $C_{26}H_{31}NO_3NaSi$: 456.1965 m/z, Found: 456.1964 m/z; **Specific rotation**: $[\alpha]_D^{20}$ 50.05 (*c* 2.00, $CHCl_3$) for an enantiomerically enriched sample of 98:2 e.r.

Enantiomeric purity of **3w** was determined by HPLC analysis in comparison with authentic racemic material (98:2 e.r. shown; Chiralpak IF column, 95:5 *n*-hexane / *i*-PrOH, 0.8 mL/min, 220 nm).

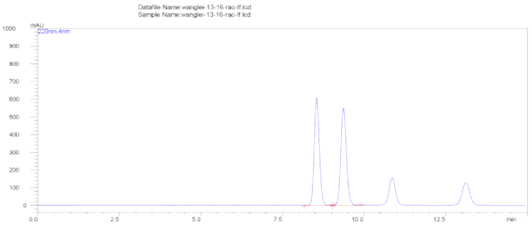

<Sample Information>

Sample Name : wanglei-13-16-rac-If.lcd
Sample ID :
Data Filename : wanglei-13-16-rac-If.lcd
Method Filename : wanglei3hao-95-5-0.8ml-40minX.lcm
Batch Filename : WWLL1.lcb
Vial # : 1-16 Sample Type : Unknown
Injection Volume : 1 uL
Date Acquired : 10/17/2022 10:15:54 PM Acquired by : System Administrator
Date Processed : 11/22/2022 1:07:48 AM Processed by : System Administrator

<Chromatogram>

<Peak Table>

PDA Ch2 220nm

Peak#	Ret. Time	Area	Area%
1	8.549	6642301	49.925
2	9.369	6662388	50.075
Total		13304689	100.000

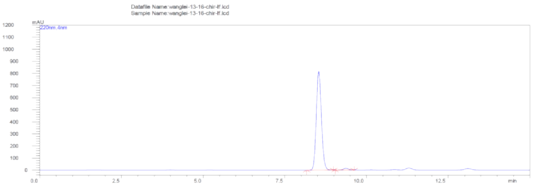

<Sample Information>

Sample Name : wanglei-13-16-chir-If.lcd
Sample ID :
Data Filename : wanglei-13-16-chir-If.lcd
Method Filename : wanglei3hao-95-5-0.8ml-40minX.lcm
Batch Filename : WWLL1.lcb
Vial # : 1-17 Sample Type : Unknown
Injection Volume : 1 uL
Date Acquired : 10/17/2022 10:31:57 PM Acquired by : System Administrator
Date Processed : 10/17/2022 11:12:01 PM Processed by : System Administrator

<Chromatogram>

<Peak Table>

PDA Ch2 220nm

Peak#	Ret. Time	Area	Area%
1	8.536	8937404	98.076
2	9.368	175328	1.924
Total		9112732	100.000

***tert*-butyl 3-((1*R*,2*R*)-1-hydroxy-2-phenyl-4-(trimethylsilyl)but-3-yn-1-yl)-1H-indole-1-carboxylate (3x)**

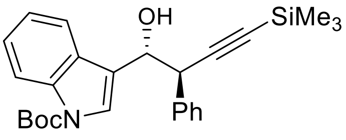

3x

Colorless oil. **IR (neat):** 3535 (w), 2175 (w), 1735 (s), 1453 (m), 1327 (m), 1252 (m), 1156 (m), 842 (s), 746 (m), 698 (m) cm^{-1}; **^{1}H NMR (400 MHz, CDCl$_3$):** δ 8.15 (d, *J* = 8.4 Hz, 1H), 7.47 (d, *J* = 6.8 Hz, 2H), 7.38 – 7.21 (m, 6H), 7.17 (t, *J* = 7.6 Hz, 1H), 5.03 (t, *J* = 4.8 Hz, 1H), 4.22 (d, *J* = 5.2 Hz, 1H), 2.68 (d, *J* = 4.8 Hz, 1H), 1.64 (s, 9H), 0.20 (s, 9H); **^{13}C NMR (100 MHz, CDCl$_3$) :** δ 149.5, 137.7, 135.4, 128.6, 128.4, 128.3, 127.4, 124.3, 123.6, 122.5, 120.7, 119.5, 115.2, 104.0, 90.9, 83.5, 72.0, 47.1, 28.1, -0.0; **HRMS (ESI$^+$) [M+Na]$^+$** Calcd for $C_{26}H_{31}NO_3NaSi$: 456.1965 m/z, Found: 456.1969 m/z; **Specific rotation**: $[\alpha]_D^{20}$ 0.95 (*c* 1.00, $CHCl_3$) for an enantiomerically enriched sample of 98:2 e.r.

Enantiomeric purity of **3x** was determined by HPLC analysis in comparison with authentic racemic material (98:2 e.r. shown; Chiralpak IG column, 92:8 *n*-hexane / *i*-PrOH, 0.8 mL/min, 254 nm).

<Sample Information>

Sample Name : wanglei-13-23-RAC-IG.lcd
Sample ID :
Data Filename : wanglei-13-23-RACX-IG.lcd
Method Filename : wanglei6hao-92-8-0.8ml-25minX.lcm
Batch Filename : WWLL1.lcb
Vial # : 1-93 Sample Type : Unknown
Injection Volume : 1 uL
Date Acquired : 10/15/2022 4:59:59 PM Acquired by : System Administrator
Date Processed : 10/15/2022 11:25:58 PM Processed by : System Administrator

<Chromatogram>

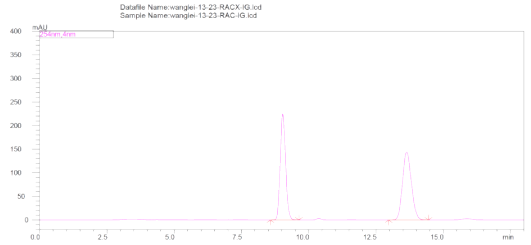

<Peak Table>

PDA Ch1 254nm

Peak#	Ret. Time	Area	Area%
1	9.033	3146792	49.304
2	13.641	3235593	50.696
Total		6382385	100.000

<Sample Information>

Sample Name : wanglei-13-23-chirx-IG.lcd
Sample ID :
Data Filename : wanglei-13-23-chirx-IG.lcd
Method Filename : wanglei6hao-92-8-0.8ml-25minX.lcm
Batch Filename : WWLL1.lcb
Vial # : 1-76 Sample Type : Unknown
Injection Volume : 1 uL
Date Acquired : 10/15/2022 10:03:09 PM Acquired by : System Administrator
Date Processed : 10/15/2022 10:28:12 PM Processed by : System Administrator

<Chromatogram>

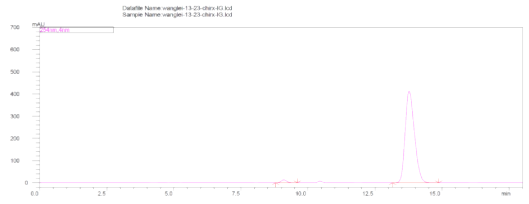

<Peak Table>

PDA Ch1 254nm

Peak#	Ret. Time	Area	Area%
1	9.097	187282	1.852
2	13.770	9924683	98.148
Total		10111966	100.000

(3*S*,4*R*)-1,4-diphenyl-6-(trimethylsilyl)hex-5-yn-3-ol (3y)

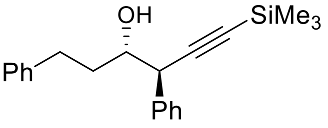

3y

Colorless oil. **IR (neat):** 3440 (w), 2174 (w), 1454 (m), 1249 (m), 1039 (m), 845 (s), 761 (m), 701 (m) cm^{-1}; **^{1}H NMR (400 MHz, CDCl$_3$):** δ 7.32 (d, *J* = 4.4 Hz, 4H), 7.29 – 7.22 (m, 3H), 7.20 – 7.12 (m, 3H), 3.75 (d, *J* = 5.6 Hz, 1H), 3.74 – 3.63 (m, 1H), 2.91 – 2.77 (m, 1H), 2.72 – 2.60 (m, 1H), 2.05 (d, *J* = 5.2 Hz, 1H), 1.99 – 1.73 (m, 2H), 0.20 (s, 9H); **^{13}C NMR (100 MHz, CDCl$_3$) :** δ 141.7, 138.0, 128.5, 128.4, 128.3, 128.3, 127.2, 125.8, 104.5, 90.0, 74.2, 46.4, 36.0, 32.0, 0.0; **HRMS (ESI$^+$) [M+Na]$^+$** Calcd for $C_{21}H_{26}ONaSi$: 345.1645 m/z, Found: 345.1644 m/z; **Specific rotation**: $[\alpha]_D^{20}$ -12.80 (*c* 2.00, $CHCl_3$) for an enantiomerically enriched sample of 92.5:7.5 e.r.

Enantiomeric purity of **3y** was determined by SFC analysis in comparison with authentic racemic material (92.5:7.5 e.r. shown; Chiralpak IC column, 95:5 CO_2 / *i*-PrOH, 0.8 mL/min, 220 nm).

<Sample Information>

Sample Name : WL-12-59-RAC-IC.lcd
Sample ID :
Data Filename : WL-12-75-RACZX-IC.lcd
Method Filename : wl-1-95-5-0.8-35xmin.lcm
Batch Filename : lwx_hydrosilylation.lcb
Vial # : 1-18 Sample Type : Unknown
Injection Volume : 6 uL
Date Acquired : 9/14/2022 2:05:46 PM Acquired by : System Administrator
Date Processed : 11/23/2022 7:36:44 PM Processed by : System Administrator

<Chromatogram>

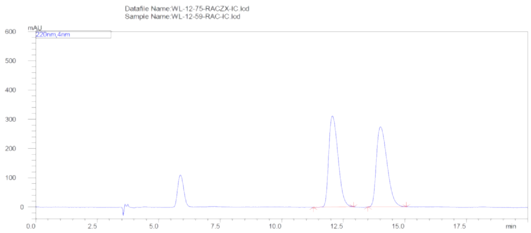

<Peak Table>

PDA Ch2 220nm

Peak#	Ret. Time	Area	Area%
1	12.062	8212366	49.918
2	14.013	8239369	50.082
Total		16451735	100.000

<Sample Information>

Sample Name : WL-12-79-CHIR-IC.lcd
Sample ID :
Data Filename : WL-12-79-CHIR-IC.lcd
Method Filename : wl-1-95-5-0.8-35xmin.lcm
Batch Filename : lwx_hydrosilylation.lcb
Vial # : 1-21 Sample Type : Unknown
Injection Volume : 10 uL
Date Acquired : 9/14/2022 2:59:24 PM Acquired by : System Administrator
Date Processed : 11/23/2022 7:38:27 PM Processed by : System Administrator

<Chromatogram>

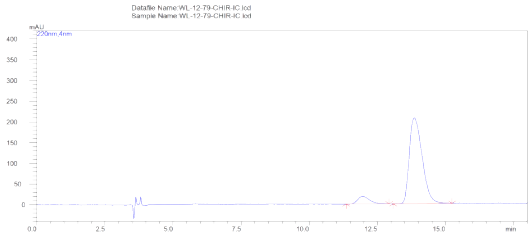

<Peak Table>

PDA Ch2 220nm

Peak#	Ret. Time	Area	Area%
1	11.947	547176	7.472
2	13.853	6775770	92.528
Total		7322946	100.000

(1*S*,2*R*)-1-cyclohexyl-2-phenyl-4-(trimethylsilyl)but-3-yn-1-ol (3z)

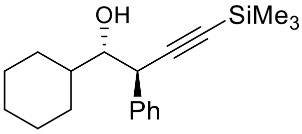

3z

Colorless oil. **IR (neat):** 3482 (w), 2172 (w), 1452 (w), 1250 (m), 841 (s), 759 (m), 698 (m) cm^{-1}; **^{1}H NMR (400 MHz, $CDCl_3$):** δ 7.38 – 7.31 (m, 4H), 7.30 – 7.21 (m, 1H), 3.94 (dd, *J* = 4.8, 1.6 Hz, 1H), 3.64 – 3.24 (m, 1H), 1.99 (d, *J* = 12.8 Hz, 1H), 1.90 (dd, *J* = 5.6, 2.0 Hz, 1H), 1.83 – 1.70 (m, 3H), 1.70 – 1.62 (m, 1H), 1.53 – 1.40 (m, 1H), 1.37 – 1.03 (m, 5H), 0.20 (s, 9H); **^{13}C NMR (100 MHz, $CDCl_3$) :** δ 138.9, 128.5, 128.1, 127.1, 104.5, 90.0, 79.4, 43.2, 41.0, 29.8, 28.0, 26.4, 26.2, 25.9, 0.1; **HRMS (ESI^+) $[M+Na]^+$** Calcd for $C_{19}H_{28}ONaSi$: 323.1802 m/z, Found: 323.1802 m/z; **Specific rotation**: $[\alpha]_D^{20}$ -7.58 (*c* 1.00, $CHCl_3$) for an enantiomerically enriched sample of 95.5:4.5 e.r.

Enantiomeric purity of **3z** was determined by SFC analysis in comparison with authentic racemic material (95.5:4.5 e.r. shown; Chiralpak IC column, 96:4 CO_2 / *i*-PrOH, 0.8 mL/min, 220 nm).

<Sample Information>

Sample Name : WL-12-84-RAC-IC.lcd
Sample ID :
Data Filename : WL-12-84-RAC-IC.lcd
Method Filename : wl-1-96-4-0.8-35xmin.lcm
Batch Filename : lwx_hydrosilylation.lcb
Vial # : 1-23 Sample Type : Unknown
Injection Volume : 2 uL
Date Acquired : 9/16/2022 9:17:35 AM Acquired by : System Administrator
Date Processed : 11/23/2022 8:26:19 PM Processed by : System Administrator

<Chromatogram>

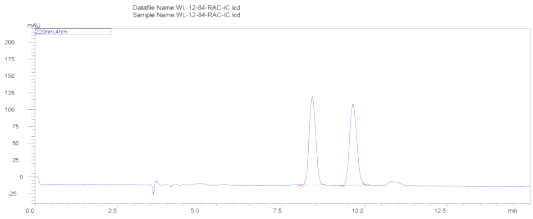

<Peak Table>

PDA Ch2 220nm

Peak#	Ret. Time	Area	Area%
1	8.406	1795798	49.255
2	9.639	1850138	50.745
Total		3645936	100.000

<Sample Information>

Sample Name : WL-12-84-CHIR-IC.lcd
Sample ID :
Data Filename : WL-12-84-CHIR-IC.lcd
Method Filename : wl-1-96-4-0.8-35xmin.lcm
Batch Filename : lwx_hydrosilylation.lcb
Vial # : 1-24 Sample Type : Unknown
Injection Volume : 2 uL
Date Acquired : 9/16/2022 9:45:45 AM Acquired by : System Administrator
Date Processed : 11/23/2022 8:27:43 PM Processed by : System Administrator

<Chromatogram> Datafile Name:WL-12-84-CHIR-IC.lcd Sample Name:WL-12-84-CHIR-IC.lcd

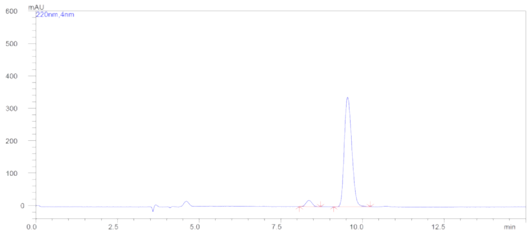

<Peak Table>

PDA Ch2 220nm

Peak#	Ret. Time	Area	Area%
1	8.357	251380	4.474
2	9.544	5366691	95.526
Total		5618072	100.000

(3*R*,4*S*)-3-phenyl-1-(trimethylsilyl)oct-1-yn-4-ol (3aa)

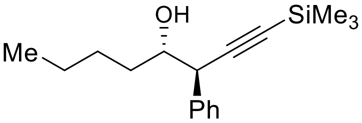

3aa

Colorless oil. **IR (neat):** 3445 (w), 2172 (w), 1250 (m), 841 (s), 760 (m), 699 (m) cm^{-1}; **^{1}H NMR (400 MHz, $CDCl_3$):** δ 7.37 – 7.30 (m, 4H), 7.29 – 7.22 (m, 1H), 3.73 (d, *J* = 5.2 Hz, 1H), 3.71 – 3.62 (m, 1H), 1.99 (d, *J* = 5.2 Hz, 1H), 1.62 – 1.42 (m, 3H), 1.39 – 1.17 (m, 3H), 0.88

(t, J = 7.2 Hz, 3H), 0.19 (s, 9H); **^{13}C NMR (100 MHz, $CDCl_3$) :** δ 138.3, 128.5, 128.3, 127.2, 104.7, 89.8, 75.1, 46.4, 34.2, 27.9, 22.5, 14.0, 0.0; **HRMS (EI$^+$) [M-H_2O]$^+$**Calcd for $C_{17}H_{24}Si$: 256.1642 m/z, Found: 256.1637 m/z; **Specific rotation**: $[\alpha]_D^{20}$ -1.33 (*c* 2.00, $CHCl_3$) for an enantiomerically enriched sample of 93.5:6.5 e.r.

Enantiomeric purity of **3aa** was determined by HPLC analysis in comparison with authentic racemic material (93.5:6.5 e.r. shown; Chiralcel OZ-H column, 99.5:0.5 *n*-hexane / *i*-PrOH, 0.8 mL/min, 220 nm).

<Sample Information>

Sample Name : wanglei-12-28-RAC-ozh.lcd
Sample ID :
Data Filename : wanglei-12-28-RACX-ozh.lcd
Method Filename : 1-99.5-0.5-0.8ml-40minX.lcm
Batch Filename : WWLL1.lcb
Vial # : 1-21 Sample Type : Unknown
Injection Volume : 1 uL
Date Acquired : 10/17/2022 2:16:54 AM Acquired by : System Administrator
Date Processed : 10/17/2022 2:56:57 AM Processed by : System Administrator

<Chromatogram>

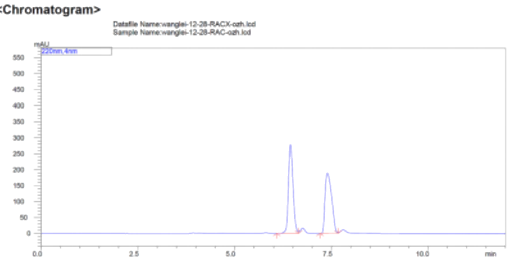

<Peak Table>

PDA Ch2 220nm

Peak#	Ret. Time	Area	Area%
1	6.436	2319765	50.728
2	7.393	2253179	49.272
Total		4572944	100.000

<Sample Information>

Sample Name : wanglei-12-28-chir-ozh.lcd
Sample ID :
Data Filename : wanglei-12-28-chirx-ozh.lcd
Method Filename : 1-99.5-0.5-0.8ml-40minX.lcm
Batch Filename : WWLL1.lcb
Vial # : 1-22 Sample Type : Unknown
Injection Volume : 0.3 uL
Date Acquired : 10/17/2022 11:34:49 AM Acquired by : System Administrator
Date Processed : 11/24/2022 4:25:52 PM Processed by : System Administrator

<Chromatogram>

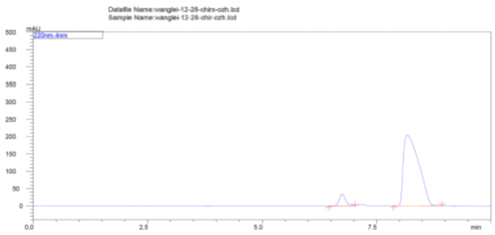

<Peak Table>

PDA Ch2 220nm

Peak#	Ret. Time	Area	Area%
1	6.747	329828	6.484
2	8.171	4756854	93.516
Total		5086682	100.000

(1*S*,2*R*)-1-cyclopropyl-2-phenyl-4-(trimethylsilyl)but-3-yn-1-ol (3ab)

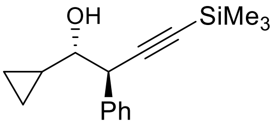

3ab

Colorless oil. **IR (neat):** 3449 (w), 2172 (w), 1249 (m), 1031 (m), 840 (s), 760 (m), 699 (m) cm^{-1}; **^{1}H NMR (400 MHz, $CDCl_3$):** δ 7.39 (d, J = 8.0 Hz, 2H), 7.33 (t, J = 7.6 Hz, 2H), 7.29 – 7.21 (m, 1H), 3.91 (d, J = 4.8 Hz, 1H), 3.05 (dt, J = 9.2, 4.8 Hz, 1H), 2.10 (d, J = 4.8 Hz, 1H), 1.15 – 0.88 (m, 1H), 0.79 – 0.41 (m, 2H), 0.40 – 0.30 (m, 1H), 0.20 (s, 9H), 0.18 – 0.09 (m, 1H); **^{13}C NMR (100 MHz, $CDCl_3$) :** δ 138.2, 128.4, 128.3, 127.1, 104.8, 89.8, 79.4, 46.8, 15.3, 3.1, 2.6, 0.0; **HRMS (EI$^+$) [M-H_2O]$^+$**Calcd for $C_{16}H_{20}Si$: 240.1329 m/z, Found: 240.1324 m/z; **Specific rotation**: $[\alpha]_D^{20}$ 0.76 (*c* 2.00, $CHCl_3$) for an enantiomerically enriched sample of 94:6 e.r.

Enantiomeric purity of **3ab** was determined by HPLC analysis in comparison with authentic racemic material (94:6 e.r. shown; Chiralpak IF column, 99.5:0.5 *n*-hexane / *i*-PrOH, 1.0 mL/min, 220 nm).

<Sample Information>

Sample Name : wanglei-13-32RAC-chir-lf.lcd
Sample ID :
Data Filename : wanglei-13-32-RAC-lf.lcd
Method Filename : wanglei3hao-99.5-0.5-1.0ml-50minX.lcm
Batch Filename : WWLL1.lcb
Vial # : 1-92 Sample Type : Unknown
Injection Volume : 1 uL
Date Acquired : 10/19/2022 10:52:55 PM Acquired by : System Administrator
Date Processed : 10/19/2022 11:46:49 PM Processed by : System Administrator

<Chromatogram>

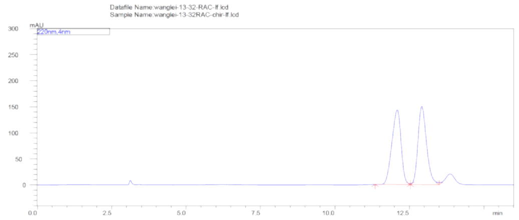

<Peak Table>

PDA Ch2 220nm

Peak#	Ret. Time	Area	Area%
1	12.095	3068731	50.203
2	12.918	3043959	49.797
Total		6112690	100.000

<Sample Information>

Sample Name : wanglei-13-32-chir-lf.lcd
Sample ID :
Data Filename : wanglei-13-32-CHIR-lf.lcd
Method Filename : wanglei3hao-99.5-0.5-1.0ml-50minX.lcm
Batch Filename : WWLL1.lcb
Vial # : 1-93 Sample Type : Unknown
Injection Volume : 1 uL
Date Acquired : 10/19/2022 11:23:40 PM Acquired by : System Administrator
Date Processed : 11/22/2022 9:23:49 AM Processed by : System Administrator

<Chromatogram>

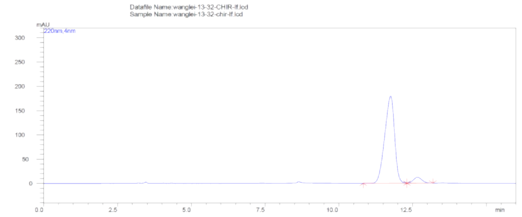

<Peak Table>

PDA Ch2 220nm

Peak#	Ret. Time	Area	Area%
1	11.753	4155198	94.356
2	12.665	248538	5.644
Total		4403736	100.000

(2*S*,3*R*,4*R*)-2-((*tert*-butyldimethylsilyl)oxy)-4-phenyl-6-(trimethylsilyl)hex-5-yn-3-ol (3ac)

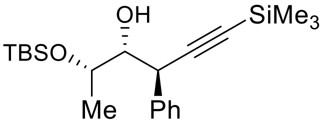

3ac

Colorless oil. **IR (neat):** 2957 (w), 2173 (w), 1453 (w), 1250 (m), 1088 (m), 837 (s), 775 (m), 698 (m) cm^{-1}; **^{1}H NMR (400 MHz, $CDCl_3$):** δ 7.40 (d, *J* = 8.0 Hz, 2H), 7.34 (t, *J* = 7.2 Hz, 2H), 7.30 – 7.22 (m, 1H), 4.28 (d, *J* = 2.8 Hz, 1H), 3.82 (p, *J* = 6.4 Hz, 1H), 3.37 (td, *J* = 8.0, 2.8 Hz, 1H), 1.80 (d, *J* = 8.0 Hz, 1H), 1.25 (d, *J* = 6.0 Hz, 3H), 0.93 (s, 9H), 0.20 (s, 9H), 0.15 (s, 3H), 0.11 (s, 3H); **^{13}C NMR (100 MHz, $CDCl_3$) :** δ 139.1, 128.5, 128.0, 127.0, 103.4, 90.7, 80.1, 70.0, 41.8, 25.9, 20.4, 18.0, 0.1, -4.1, -5.0; **HRMS (ESI^{+}) [M+Na]$^{+}$** Calcd for $C_{21}H_{36}O_2NaSi_2$: 399.2146 m/z, Found: 399.2140 m/z; **Specific rotation**: $[\alpha]_D^{20}$ -22.84 (*c* 2.00, $CHCl_3$) for an enantiomerically enriched sample.

(2*S*,3*S*,4*S*)-2-((tert-butyldimethylsilyl)oxy)-4-phenyl-6-(trimethylsilyl)hex-5-yn-3-ol (3ad)

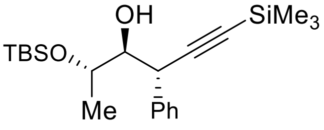

3ad

Colorless oil. **IR (neat):** 2958 (w), 2173 (w), 1452 (w), 1259 (m), 1090 (m), 840 (s), 776 (m), 698 (m) cm^{-1}; **^{1}H NMR (400 MHz, $CDCl_3$):** δ 7.40 (d, *J* = 6.8 Hz, 2H), 7.33 (t, *J* = 7.6 Hz, 2H), 7.29 – 7.21 (m, 1H), 4.16 – 3.80 (m, 2H), 3.48 (q, *J* = 5.2 Hz, 1H), 2.60 (d, *J* = 5.2 Hz, 1H), 1.24 (d, *J* = 6.4 Hz, 3H), 0.91 (s, 9H), 0.19 (s, 9H), 0.09 (s, 3H), 0.08 (s, 3H); **^{13}C NMR (100 MHz, $CDCl_3$) :** δ 138.8, 128.4, 128.2, 127.0, 104.6, 89.4, 78.9, 69.8, 41.8, 25.8, 20.1, 18.0, 0.1, -4.1, -4.8; **HRMS (ESI^{+}) [M+Na]$^{+}$** Calcd for $C_{21}H_{36}O_2NaSi_2$: 399.2146 m/z, Found: 399.2145 m/z; **Specific rotation**: $[\alpha]_D^{20}$ -19.28 (*c* 1.00, $CHCl_3$) for an enantiomerically enriched sample.

(1*R*,2*R*)-1-((*S*)-2,2-dimethyl-1,3-dioxolan-4-yl)-2-phenyl-4-(trimethylsilyl)but-3-yn-1-ol (3ae)

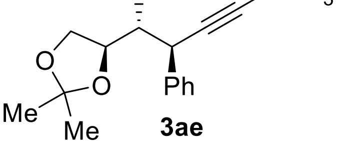

3ae

Colorless oil. **IR (neat):** 3556 (w), 2173 (w), 1453 (m), 1205 (m), 1072 (m), 845 (s), 760 (m), 733 (m) cm^{-1}; **^{1}H NMR (400 MHz, $CDCl_3$):** δ 7.40 (d, *J* = 6.8 Hz, 2H), 7.34 (t, *J* = 7.6 Hz, 2H), 7.32 – 7.23 (m, 1H), 3.99 (q, *J* = 6.4 Hz, 1H), 3.96 – 3.86 (m, 2H), 3.86 – 3.77 (m, 1H), 3.67 (q, *J* = 5.6 Hz, 1H), 2.54 (d, *J* = 5.6 Hz, 1H), 1.44 (s, 3H), 1.33 (s, 3H), 0.19 (s, 9H); **^{13}C NMR (100 MHz, $CDCl_3$) :** δ 137.3, 128.5, 127.4, 109.3,

104.5, 89.6, 76.0, 74.9, 66.0, 43.3, 26.4, 25.4, -0.0; **HRMS (ESI$^+$) [M+Na]$^+$** Calcd for $C_{18}H_{26}O_3NaSi$: 341.1543 m/z, Found: 341.1544 m/z; **Specific rotation**: $[\alpha]_D^{20}$ 4.09 (*c* 1.00, $CHCl_3$) for an enantiomerically enriched sample.

***tert*-butyl ((2*S*,3*S*,4*S*)-3-hydroxy-4-phenyl-6-(trimethylsilyl)hex-5-yn-2-yl)carbamate (3af)**

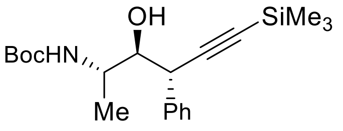

3af

Colorless oil. **IR (neat):** 3438 (w), 2173 (w), 1692 (s), 1506 (m), 1249 (m), 1052 (m), 842 (s), 760 (m), 699 (m) cm^{-1}; **^{1}H NMR (400 MHz, $CDCl_3$):** δ 7.34 (d, *J* = 4.4 Hz, 4H), 7.30 – 7.24 (m, 1H), 4.93 (d, *J* = 9.2 Hz, 1H), 3.81 (d, *J* = 8.4 Hz, 1H), 3.61 (s, 1H), 3.51 (d, *J* = 8.4 Hz, 1H), 2.89 (s, 1H), 1.47 (s, 9H), 1.14 (d, *J* = 6.8 Hz, 3H), 0.19 (s, 9H); **^{13}C NMR (100 MHz, $CDCl_3$) :** δ 155.5, 137.3, 128.6, 128.5, 127.5, 105.2, 89.9, 79.2, 78.0, 46.0, 43.9, 28.4, 19.2, -0.0; **HRMS (ESI$^+$) [M+Na]$^+$** Calcd for $C_{20}H_{31}NO_3NaSi$: 384.1965 m/z, Found: 384.1966 m/z; **Specific rotation**: $[\alpha]_D^{20}$ -24.40 (*c* 2.00, $CHCl_3$) for an enantiomerically enriched sample.

(*syn*)-1,2-diphenyl-4-(trimethylsilyl)but-3-yn-1-ol ((±)-4a)

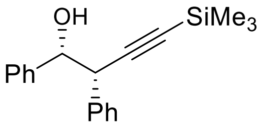

(±)-**4a**

Colorless oil. **^{1}H NMR (400 MHz, $CDCl_3$):** δ 7.87 – 6.87 (m, 10H), 4.85 (dd, *J* = 7.2, 3.6 Hz, 1H), 3.99 (d, *J* = 7.2 Hz, 1H), 2.25 (d, *J* = 3.6 Hz, 1H), 0.15 (s, 9H); **^{13}C NMR (100 MHz, $CDCl_3$):** δ 140.6, 137.3, 128.8, 128.4, 127.8, 127.7, 127.5, 127.0, 104.8, 90.1, 78.0, 48.2, -0.1.

(1*R*,2*R*)-1-phenyl-2-(4-(trifluoromethyl)phenyl)-4-(trimethylsilyl)but-3-yn-1-ol (6a)

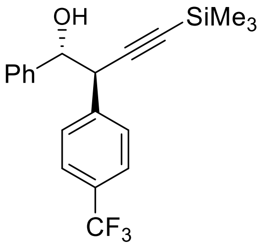

6a

Colorless oil. **IR (neat):** 3542 (w), 2176 (w), 1326 (s), 1165 (m), 1068 (m), 842 (s), 761 (m), 701 (m) cm^{-1}; **^{1}H NMR (400 MHz, $CDCl_3$):** δ 7.47 (d, *J* = 8.0 Hz, 2H), 7.44 – 7.08 (m, 5H), 7.20 – 7.04 (m, 2H), 4.73 (dd, *J* = 6.4, 3.6 Hz, 1H), 3.98 (d, *J* = 6.4 Hz, 1H), 2.80 (d, *J* = 3.6 Hz, 1H), 0.20 (s, 9H); **^{13}C NMR (100 MHz, $CDCl_3$) :** δ 141.4, 140.1, 129.4 (q, *J* = 33 Hz), 129.0, 128.0, 127.9, 126.6, 125.0 (q, *J* = 3.8 Hz), 124.1 (q, *J* = 271 Hz), 103.6, 91.4, 77.8, 48.6, -0.1; **^{19}F NMR (376 MHz, $CDCl_3$):** δ -62.4; **HRMS (ESI$^+$) [M+Na]$^+$** Calcd for $C_{20}H_{21}OF_3NaSi$: 385.1206 m/z, Found: 385.1204 m/z; **Specific rotation**: $[\alpha]_D^{20}$ -21.00 (*c* 2.00, $CHCl_3$) for an enantiomerically enriched sample of 96:4 e.r.

Enantiomeric purity of **6a** was determined by SFC analysis in comparison with authentic racemic material (96:4 e.r. shown; Chiralpak IF column, 97:3 CO_2 / *i*-PrOH, 0.8 mL/min, 220 nm).

<Sample Information>

Sample Name : WL-12-81-RAC-IF.lcd
Sample ID :
Data Filename : WL-12-81-RAC-IF.lcd
Method Filename : wl-6-97-3-0.8-35xmin.lcm
Batch Filename : lwx_hydrosilylation.lcb
Vial # : 1-19 Sample Type : Unknown
Injection Volume : 2 uL
Date Acquired : 9/17/2022 8:16:48 AM Acquired by : System Administrator
Date Processed : 11/23/2022 7:47:00 PM Processed by : System Administrator

<Chromatogram>

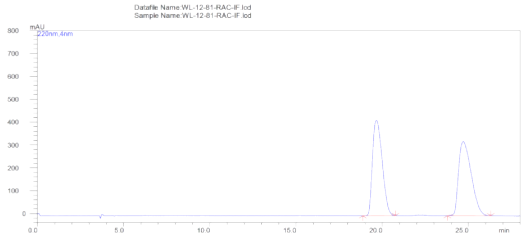

<Peak Table>

PDA Ch2 220nm

Peak#	Ret. Time	Area	Area%
1	19.667	16321668	49.671
2	24.701	16537777	50.329
Total		32859445	100.000

<Sample Information>

Sample Name : WL-12-81-CHIR-IF.lcd
Sample ID :
Data Filename : WL-12-81-CHIR-IF.lcd
Method Filename : wl-6-97-3-0.8-35xmin.lcm
Batch Filename : lwx_hydrosilylation.lcb
Vial # : 1-21 Sample Type : Unknown
Injection Volume : 2 uL
Date Acquired : 9/17/2022 10:48:16 AM Acquired by : System Administrator
Date Processed : 11/23/2022 7:45:26 PM Processed by : System Administrator

<Chromatogram>

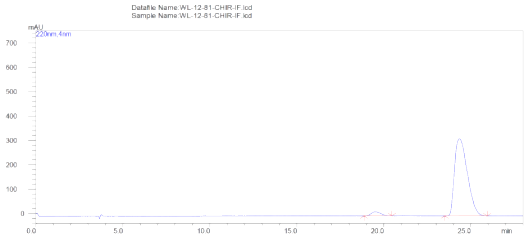

<Peak Table>

PDA Ch2 220nm

Peak#	Ret. Time	Area	Area%
1	19.529	624204	3.718
2	24.341	16163632	96.282
Total		16787836	100.000

4-((1*R*,2*R*)-1-hydroxy-1-phenyl-4-(trimethylsilyl)but-3-yn-2-yl)benzonitrile (6b)

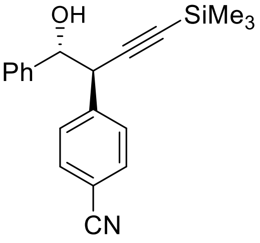

6b

Colorless oil. **IR (neat):** 3478 (w), 2229 (w), 1607 (m), 1249 (m), 842 (s), 760 (m), 700 (m) cm^{-1}; **^{1}H NMR (400 MHz, CDCl$_3$):** δ 7.50 (d, *J* = 8.4 Hz, 2H), 7.33 – 7.18 (m, 5H), 7.15 – 7.07 (m, 2H), 4.77 (dd, *J* = 6.4, 3.6 Hz, 1H), 4.01 (d, *J* = 6.4 Hz, 1H), 2.74 (d, *J* = 3.6 Hz, 1H), 0.21 (s, 9H); **^{13}C NMR (100 MHz, CDCl$_3$):** δ 142.7, 139.8, 131.8, 129.5, 128.2, 128.0, 126.6, 118.7, 111.0, 103.1, 91.7, 77.7, 48.7, -0.2; **HRMS (ESI$^+$) [M+Na]$^+$** Calcd for $C_{20}H_{21}NONaSi$: 342.1285 m/z, Found: 342.1276 m/z; **Specific rotation**: $[\alpha]_D^{20}$ -55.23 (*c* 1.00, $CHCl_3$) for an enantiomerically enriched sample of 96:4 e.r.

Enantiomeric purity of **6b** was determined by SFC analysis in comparison with authentic racemic material (96:4 e.r. shown; Chiralpak IF column, 88:12 CO_2 / *i*-PrOH, 1.5 mL/min, 220 nm).

<Sample Information>

Sample Name : WL-13-12rac-IF.lcd
Sample ID :
Data Filename : WL-13-12racZX-IF.lcd
Method Filename : wl-3-88-12-1.5-15xmin.lcm
Batch Filename : lwx_hydrosilylation.lcb
Vial # : 1-16 Sample Type : Unknown
Injection Volume : 3 uL
Date Acquired : 10/7/2022 8:47:49 PM Acquired by : System Administrator
Date Processed : 11/24/2022 3:03:50 PM Processed by : System Administrator

<Chromatogram>

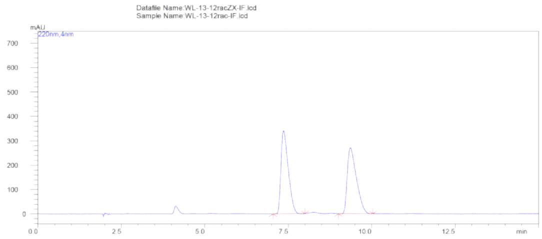

<Peak Table>

PDA Ch2 220nm

Peak#	Ret. Time	Area	Area%
1	7.359	5184655	50.066
2	9.363	5171061	49.934
Total		10355715	100.000

<Sample Information>

Sample Name : WL-13-12CHIR-IF.lcd
Sample ID :
Data Filename : WL-13-12CHIR-IF.lcd
Method Filename : wl-3-88-12-1.5-15xmin.lcm
Batch Filename : lwx_hydrosilylation.lcb
Vial # : 1-16 Sample Type : Unknown
Injection Volume : 2 uL
Date Acquired : 10/8/2022 11:04:45 AM Acquired by : System Administrator
Date Processed : 10/11/2022 9:07:14 AM Processed by : System Administrator

<Chromatogram>

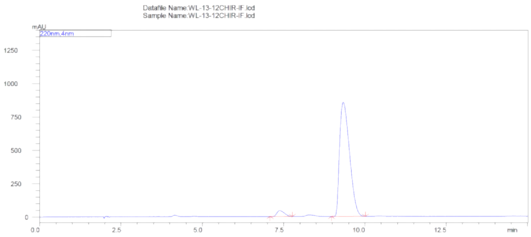

<Peak Table>

PDA Ch2 220nm

Peak#	Ret. Time	Area	Area%
1	7.383	686974	3.648
2	9.333	18143449	96.352
Total		18830423	100.000

Methyl 4-((1*R*,2*R*)-1-hydroxy-1-phenyl-4-(trimethylsilyl)but-3-yn-2-yl)benzoate (6c)

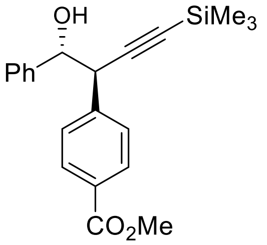

6c

White solid, m.p. 68–70 ºC; **IR (neat):** 3509 (w), 2177 (w), 1718 (m), 1282 (m), 844 (s), 758 (m), 701 (m) cm^{-1}; **^{1}H NMR (400 MHz, $CDCl_3$):** δ 7.89 (d, *J* = 8.4 Hz, 2H), 7.28 – 7.22 (m, 3H), 7.19 (d, *J* = 8.0 Hz, 2H), 7.15 – 7.07 (m, 2H), 4.76 (d, *J* = 6.4 Hz, 1H), 3.99 (d, *J* = 6.8 Hz, 1H), 3.89 (s, 3H), 2.80 (s, 1H), 0.21 (s, 9H); **^{13}C NMR (100 MHz, $CDCl_3$):** δ 166.8, 142.5, 140.1, 129.4, 129.0, 128.7, 128.0, 127.9, 126.6, 103.8, 91.3, 77.9, 52.1, 48.9, -0.1; **HRMS (ESI^{+}) [M+Na]$^{+}$** Calcd for $C_{21}H_{24}O_3NaSi$: 375.1387 m/z, Found: 375.1387 m/z; **Specific rotation**: $[\alpha]_D^{20}$ -37.79 (*c* 1.00, $CHCl_3$) for an enantiomerically enriched sample of 96:4 e.r.

Enantiomeric purity of **6c** was determined by HPLC analysis in comparison with authentic racemic material (96:4 e.r. shown; Chiralpak IF column, 92:8 *n*-hexane / *i*-PrOH, 0.8 mL/min, 220 nm).

<Sample Information>

Sample Name : wanglei-13-13-RAC-If.lcd
Sample ID :
Data Filename : wanglei-13-13-RACX-If.lcd
Method Filename : wanglei3hao-92-8-0.8ml-40minX.lcm
Batch Filename : WWLL1.lcb
Vial # : 1-93 Sample Type : Unknown
Injection Volume : 1 uL
Date Acquired : 10/17/2022 8:46:28 PM Acquired by : System Administrator
Date Processed : 10/17/2022 9:18:07 PM Processed by : System Administrator

<Chromatogram>

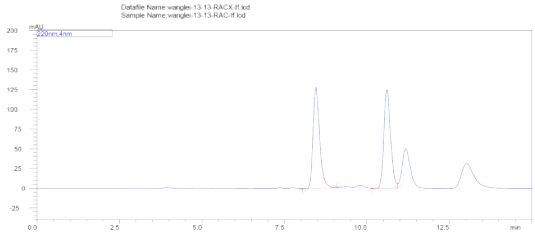

<Peak Table>

PDA Ch2 220nm

Peak#	Ret. Time	Area	Area%
1	8.453	1775658	50.095
2	10.605	1768912	49.905
Total		3544570	100.000

<Sample Information>

Sample Name : wanglei-13-13-chir-If.lcd
Sample ID :
Data Filename : wanglei-13-13-chir-If.lcd
Method Filename : wanglei3hao-92-8-0.8ml-40minX.lcm
Batch Filename : WWLL1.lcb
Vial # : 1-17 Sample Type : Unknown
Injection Volume : 1 uL
Date Acquired : 10/17/2022 9:20:12 PM Acquired by : System Administrator
Date Processed : 10/17/2022 9:38:14 PM Processed by : System Administrator

<Chromatogram>

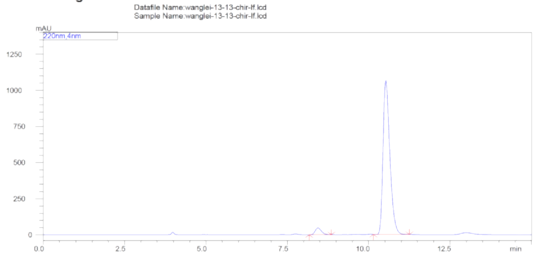

<Peak Table>

PDA Ch2 220nm

Peak#	Ret. Time	Area	Area%
1	8.441	640686	3.958
2	10.526	15545646	96.042
Total		16186332	100.000

(1*R*,2*R*)-2-(4-methoxyphenyl)-1-phenyl-4-(trimethylsilyl)but-3-yn-1-ol (6d)

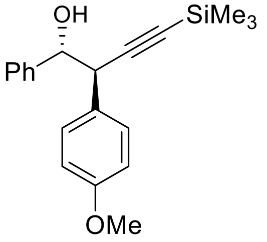

6d

Colorless oil. **IR (neat):** 3460 (w), 2173 (w), 1610 (s), 1248 (s), 842 (s), 761 (m), 699 (m) cm^{-1}; **^{1}H NMR (400 MHz, $CDCl_3$):** δ 7.28 – 7.21 (m, 3H), 7.19 – 7.10 (m, 2H), 7.04 (d, *J* = 8.4 Hz, 2H), 6.77 (d, *J* = 8.4 Hz, 2H), 4.71 (d, *J* = 6.8 Hz, 1H), 3.89 (d, *J* = 6.4 Hz, 1H), 3.76 (s, 3H), 2.74 (s, 1H), 0.20 (s, 9H); **^{13}C NMR (100 MHz, $CDCl_3$) :** δ 158.8, 140.6, 129.5, 129.3, 127.8, 127.7, 126.7, 113.6, 104.8, 90.5, 78.1, 55.2, 48.2, -0.0; **HRMS (ESI^{+}) [M+Na]$^{+}$** Calcd for $C_{20}H_{24}O_2NaSi$: 347.1438 m/z, Found: 347.1438 m/z; **Specific rotation**: $[\alpha]_D^{20}$ -43.06 (*c* 1.00, $CHCl_3$) for an enantiomerically enriched sample of 97:3 e.r.

Enantiomeric purity of **6d** was determined by SFC analysis in comparison with authentic racemic material (97:3 e.r. shown; Chiralpak IC column, 94:6 CO_2 / *i*-PrOH, 0.6 mL/min, 220 nm).

<Sample Information>

Sample Name : WL-12-83-RAC-IC.lcd
Sample ID :
Data Filename : WL-12-83-RACX-IC.lcd
Method Filename : wl-1-94-6-0.6-45xmin.lcm
Batch Filename : lwx_hydrosilylation.lcb
Vial # : 1-16 Sample Type : Unknown
Injection Volume : 19 uL
Date Acquired : 9/17/2022 3:24:52 PM Acquired by : System Administrator
Date Processed : 11/23/2022 8:16:36 PM Processed by : System Administrator

<Chromatogram>

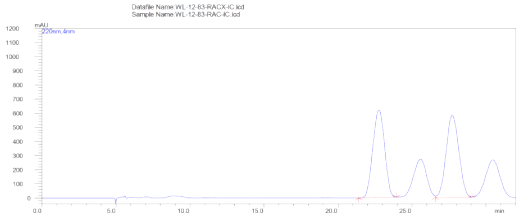

<Peak Table>

PDA Ch2 220nm

Peak#	Ret. Time	Area	Area%
1	22.769	34215933	49.703
2	27.715	34625045	50.297
Total		68840977	100.000

<Sample Information>

Sample Name : WL-12-83-RAC-IC.lcd
Sample ID :
Data Filename : WL-12-83-CHIRZX-IC.lcd
Method Filename : wl-1-94-6-0.6-45xmin.lcm
Batch Filename : lwx_hydrosilylation.lcb
Vial # : 1-17 Sample Type : Unknown
Injection Volume : 4 uL
Date Acquired : 9/17/2022 2:38:42 PM Acquired by : System Administrator
Date Processed : 11/23/2022 8:18:08 PM Processed by : System Administrator

<Chromatogram>

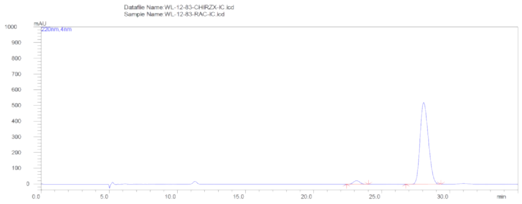

<Peak Table>

PDA Ch2 220nm

Peak#	Ret. Time	Area	Area%
1	23.147	771573	3.400
2	28.081	21919833	96.600
Total		22691406	100.000

(1*R*,2*R*)-2-(4-fluorophenyl)-1-phenyl-4-(trimethylsilyl)but-3-yn-1-ol (6e)

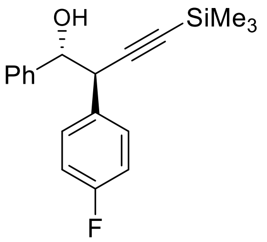

6e

Colorless oil. **IR (neat):** 3397 (w), 2175 (w), 1508 (m), 1250 (m), 841 (s), 761 (m), 699 (m) cm^{-1}; **^{1}H NMR (400 MHz, $CDCl_3$):** δ 7.38 – 7.17 (m, 3H), 7.14 – 7.08 (m, 2H), 7.08 – 7.00 (m, 2H), 6.89 (t, J = 8.8 Hz, 2H), 4.68 (dd, J = 6.8, 3.6 Hz, 1H), 3.90 (d, J = 6.8 Hz, 1H), 2.79 (d, J = 3.6 Hz, 1H), 0.20 (s, 9H); **^{13}C NMR (100 MHz, $CDCl_3$) :** δ 162.0 (d, J = 245.7 Hz), 140.2, 132.9 (d, J = 3.2 Hz), 130.1 (d, J = 8.1 Hz), 127.9, 127.8, 126.7, 114.9 (d, J = 21.4 Hz), 104.4, 90.8, 78.0, 48.1, -0.1; **^{19}F NMR (376 MHz, $CDCl_3$):** δ -115.3; **HRMS (EI$^+$) [M-H_2O]$^+$**Calcd for $C_{19}H_{19}FSi$: 294.1235 m/z, Found: 294.1241 m/z; **Specific rotation**: $[\alpha]_D^{20}$ -18.87 (*c* 2.00, $CHCl_3$) for an enantiomerically enriched sample of 97:3 e.r.

Enantiomeric purity of **6e** was determined by SFC analysis in comparison with authentic racemic material (97:3 e.r. shown; Chiralpak IF column, 89:11 CO_2 / *i*-PrOH, 0.8 mL/min, 220 nm).

<Sample Information>

Sample Name : WL-13-1-RAC-IF.lcd
Sample ID :
Data Filename : WL-13-1-RAC-IF.lcd
Method Filename : wl-3-89-11-0.8-35xmin.lcm
Batch Filename : lwx_hydrosilylation.lcb
Vial # : 1-77 Sample Type : Unknown
Injection Volume : 6 uL
Date Acquired : 10/4/2022 7:03:54 PM Acquired by : System Administrator
Date Processed : 11/24/2022 12:27:28 AM Processed by : System Administrator

<Chromatogram>

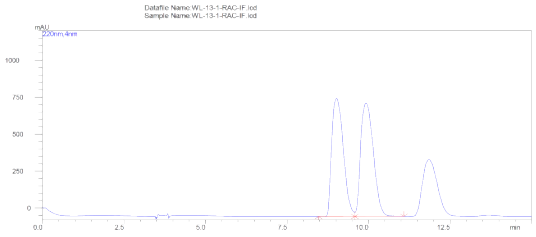

<Peak Table>

PDA Ch2 220nm

Peak#	Ret. Time	Area	Area%
1	9.018	19703494	49.340
2	9.922	20230906	50.660
Total		39934401	100.000

<Sample Information>

Sample Name : WL-13-1-chir-IF.lcd
Sample ID :
Data Filename : WL-13-1-chir-IF.lcd
Method Filename : wl-3-89-11-0.8-20xmin.lcm
Batch Filename : lwx_hydrosilylation.lcb
Vial # : 1-16 Sample Type : Unknown
Injection Volume : 2 uL
Date Acquired : 10/5/2022 10:00:12 AM Acquired by : System Administrator
Date Processed : 11/24/2022 12:28:47 AM Processed by : System Administrator

<Chromatogram>

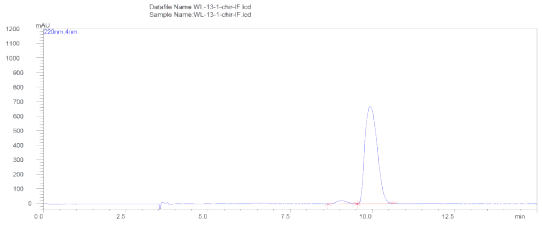

<Peak Table>

PDA Ch2 220nm

Peak#	Ret. Time	Area	Area%
1	9.057	517061	2.892
2	9.931	17364111	97.108
Total		17881173	100.000

(1*R*,2*S*)-2-(4-chlorophenyl)-1-phenyl-4-(trimethylsilyl)but-3-yn-1-ol (6f)

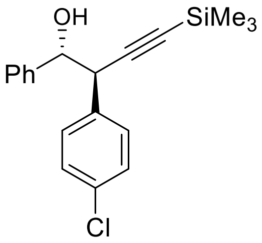

6f

Colorless oil. **IR (neat):** 3427 (w), 2175 (w), 1491 (m), 1246 (m), 842 (s), 760 (m), 665 (m) cm^{-1}; **^{1}H NMR (400 MHz, CDCl$_3$):** δ 7.27 – 7.21 (m, 3H), 7.18 (d, J = 8.4 Hz, 2H), 7.13 – 7.08 (m, 2H), 7.03 (d, J = 8.4 Hz, 2H), 4.69 (dd, J = 6.4, 3.6 Hz, 1H), 3.89 (d, J = 6.4 Hz, 1H), 2.77 (d, J = 3.6 Hz, 1H), 0.20 (s, 9H); **^{13}C NMR (100 MHz, CDCl$_3$) :** δ 140.1, 135.7, 133.0, 129.9, 128.2, 127.9, 127.9, 126.6, 104.1, 91.0, 77.8, 48.2, -0.1; **HRMS (ESI$^+$) [M+Na]$^+$** Calcd for $C_{19}H_{21}ONaSiCl$: 351.0942 m/z, Found: 351.0947 m/z; **Specific rotation**: $[\alpha]_D^{20}$ -37.45 (c 2.00, $CHCl_3$) for an enantiomerically enriched sample of 97:3 e.r.

Enantiomeric purity of **6f** was determined by SFC analysis in comparison with authentic racemic material (97:3 e.r. shown; Chiralpak ID column, 95:5 CO_2 / i-PrOH, 0.8 mL/min, 220 nm).

<Sample Information>

Sample Name : WL-13-2-rac-ID.lcd
Sample ID :
Data Filename : WL-13-2-racC2-ID.lcd
Method Filename : wl-2-95-5-0.8-55xmin.lcm
Batch Filename : lwx_hydrosilylation.lcb
Vial # : 1-94
Sample Type : Unknown
Injection Volume : 3 uL
Date Acquired : 10/7/2022 10:12:45 AM
Acquired by : System Administrator
Date Processed : 11/24/2022 12:38:31 AM
Processed by : System Administrator

<Chromatogram>

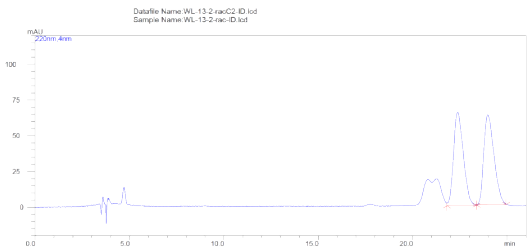

<Peak Table>

PDA Ch2 220nm

Peak#	Ret. Time	Area	Area%
1	22.382	2358475	49.762
2	23.980	2381012	50.238
Total		4739488	100.000

<Sample Information>

Sample Name : WL-13-2-CHIR-ID.lcd
Sample ID :
Data Filename : WL-13-2-CHIR-ID.lcd
Method Filename : wl-2-95-5-0.8-35xmin.lcm
Batch Filename : lwx_hydrosilylation.lcb
Vial # : 1-18
Sample Type : Unknown
Injection Volume : 1 uL
Date Acquired : 10/7/2022 5:39:22 PM
Acquired by : System Administrator
Date Processed : 10/7/2022 6:10:10 PM
Processed by : System Administrator

<Chromatogram>

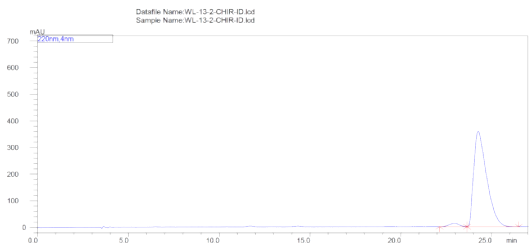

<Peak Table>

PDA Ch2 220nm

Peak#	Ret. Time	Area	Area%
1	22.935	504690	2.766
2	24.264	17743107	97.234
Total		18247797	100.000

(1*R*,2*R*)-2-(4-bromophenyl)-1-phenyl-4-(trimethylsilyl)but-3-yn-1-ol (6g)

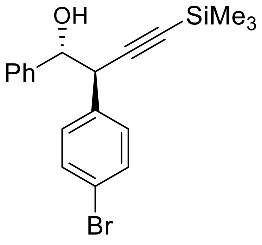

6g

Colorless oil. **IR (neat):** 3444 (w), 2175 (w), 1486 (m), 1249 (m), 842 (s), 760 (m), 700 (m) cm^{-1}; **^{1}H NMR (400 MHz, CDCl$_3$):** δ 7.34 (d, J = 8.4 Hz, 2H), 7.28 – 7.19 (m, 3H), 7.18 – 7.09 (m, 2H), 6.98 (d, J = 8.4 Hz, 2H), 4.71 (dd, J = 6.8, 2.8 Hz, 1H), 3.89 (d, J = 6.8 Hz, 1H), 2.73 (d, J = 3.2 Hz, 1H), 0.20 (s, 9H); **^{13}C NMR (100 MHz, CDCl$_3$):** δ 140.1, 136.3, 131.2, 130.3, 128.0, 127.9, 126.6, 121.2, 104.0, 91.0, 77.8, 48.3, -0.1; **HRMS (ESI$^+$) [M+Na]$^+$** Calcd for $C_{19}H_{21}ONaSiBr$: 395.0437 m/z, Found: 395.0434 m/z; **Specific rotation**: $[\alpha]_D^{20}$ -37.37 (c 2.00, $CHCl_3$) for an enantiomerically enriched sample of 97:3 e.r.

Enantiomeric purity of **6g** was determined by SFC analysis in comparison with authentic racemic material (97:3 e.r. shown; Chiralpak IF column, 89:11 CO_2 / i-PrOH, 0.8 mL/min, 220 nm).

<Sample Information>

Sample Name : WL-13-3-RAC-IF.lcd
Sample ID :
Data Filename : WL-13-3-RAC-IF.lcd
Method Filename : wl-3-89-11-0.8-35xmin.lcm
Batch Filename : lwx_hydrosilylation.lcb
Vial # : 1-79 Sample Type : Unknown
Injection Volume : 6 uL
Date Acquired : 10/4/2022 8:15:44 PM Acquired by : System Administrator
Date Processed : 11/24/2022 12:48:11 AM Processed by : System Administrator

<Chromatogram>

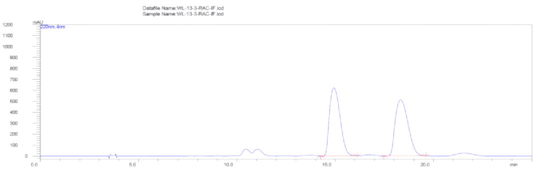

<Peak Table>

PDA Ch2 220nm

Peak#	Ret. Time	Area	Area%
1	14.937	23261479	50.245
2	18.329	23034795	49.755
Total		46296273	100.000

<Sample Information>

Sample Name : WL-13-3-chir-IF.lcd
Sample ID :
Data Filename : WL-13-3-chir-IF.lcd
Method Filename : wl-3-89-11-0.8-35xmin.lcm
Batch Filename : lwx_hydrosilylation.lcb
Vial # : 1-17 Sample Type : Unknown
Injection Volume : 2 uL
Date Acquired : 10/5/2022 10:21:03 AM Acquired by : System Administrator
Date Processed : 11/24/2022 12:46:54 AM Processed by : System Administrator

<Chromatogram>

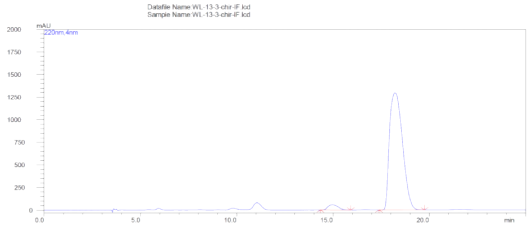

<Peak Table>

PDA Ch2 220nm

Peak#	Ret. Time	Area	Area%
1	14.966	2148881	3.405
2	18.211	60968489	96.595
Total		63117370	100.000

(1*R*,2*R*)-1-phenyl-2-(3-(trifluoromethyl)phenyl)-4-(trimethylsilyl)but-3-yn-1-ol (6h)

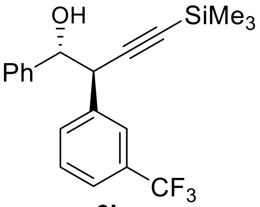

6h

White solid, m.p. 39–40 °C; **IR (neat):** 3328 (w), 2179 (w), 1332 (m), 1251 (m), 1125 (m), 842 (s), 760 (m), 700 (m) cm^{-1}; **^{1}H NMR (400 MHz, $CDCl_3$):** δ 7.46 (d, *J* = 7.2 Hz, 1H), 7.38 – 7.27 (m, 3H), 7.27 – 7.20 (m, 3H), 7.14 – 7.07 (m, 2H), 4.74 (dd, *J* = 6.4, 2.4 Hz, 1H), 3.99 (d, *J* = 6.4 Hz, 1H), 2.75 (s, 1H), 0.21 (s, 9H); **^{13}C NMR (100 MHz, $CDCl_3$):** δ 140.0, 138.3, 132.0, 130.4 (q, *J* = 32.2 Hz), 128.5, 128.1, 128.0, 126.6, 125.6 (q, *J* = 3.9 Hz), 124.1 (q, *J* = 3.8 Hz), 124.0 (q, *J* = 272.4 Hz), 103.7, 91.6, 77.9, 48.6, -0.1; **^{19}F NMR (376 MHz, $CDCl_3$):** δ -62.7; **HRMS (EI^+) $[M-H_2O]^+$**Calcd for $C_{20}H_{19}F_3Si$: 344.1203 m/z, Found: 344.1213 m/z; **Specific rotation**: $[\alpha]_D^{20}$ -13.13 (*c* 2.00, $CHCl_3$) for an enantiomerically enriched sample of 97:3 e.r.,

Enantiomeric purity of **6h** was determined by SFC analysis in comparison with authentic racemic material (97:3 e.r. shown; Chiralpak ID column, 95:5 CO_2 / *i*-PrOH, 0.8 mL/min, 220 nm).

<Sample Information>

Sample Name : WL-13-7-rac-ID.lcd
Sample ID :
Data Filename : WL-13-7-rac2-ID.lcd
Method Filename : wl-2-95-5-0.8-55xmin.lcm
Batch Filename : lwx_hydrosilylation.lcb
Vial # : 1-22 Sample Type : Unknown
Injection Volume : 3 uL
Date Acquired : 10/7/2022 8:36:29 AM Acquired by : System Administrator
Date Processed : 11/24/2022 2:42:22 PM Processed by : System Administrator

<Chromatogram>

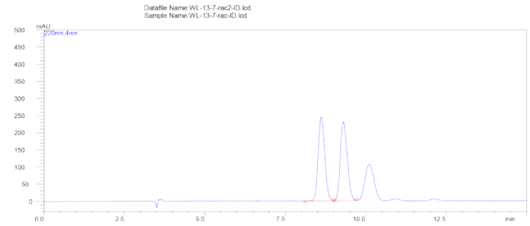

<Peak Table>

PDA Ch2 220nm

Peak#	Ret. Time	Area	Area%
1	8.616	3479900	49.873
2	9.306	3497662	50.127
Total		6977561	100.000

<Sample Information>

Sample Name : WL-13-7-CHIR-ID.lcd
Sample ID :
Data Filename : WL-13-7-CHIRX-ID.lcd
Method Filename : wl-2-95-5-0.8-20xmin.lcm
Batch Filename : lwx_hydrosilylation.lcb
Vial # : 1-77 Sample Type : Unknown
Injection Volume : 2 uL
Date Acquired : 10/7/2022 11:14:50 AM Acquired by : System Administrator
Date Processed : 11/24/2022 2:40:35 PM Processed by : System Administrator

<Chromatogram>

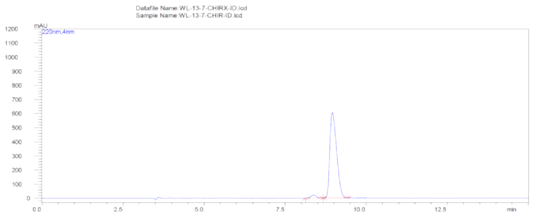

<Peak Table>

PDA Ch2 220nm

Peak#	Ret. Time	Area	Area%
1	8.374	267632	2.955
2	8.955	8788789	97.045
Total		9056421	100.000

(1*R*,2*R*)-2-(3-methoxyphenyl)-1-phenyl-4-(trimethylsilyl)but-3-yn-1-ol (6i)

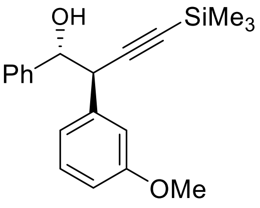

6i

White solid, m.p. 49–51 ºC; **IR (neat):** 3339 (w), 2178 (w), 1610 (m), 1315 (m),1043 (s), 897 (s), 781 (m), 700 (m) cm^{-1}; **^{1}H NMR (400 MHz, CDCl$_3$):** δ 7.28 – 7.21 (m, 3H), 7.20 – 7.15 (m, 2H), 7.13 (d, *J* = 8.0 Hz, 1H), 6.75 (d, *J* = 8.0 Hz, 2H), 6.72 – 6.66 (m, 1H), 4.74 (dd, *J* = 6.4, 2.4 Hz, 1H), 3.90 (d, *J* = 6.4 Hz, 1H), 3.68 (s, 3H), 2.75 (d, *J* = 3.2 Hz, 1H), 0.20 (s, 9H); **^{13}C NMR (100 MHz, CDCl$_3$):** δ 159.4, 140.6, 138.8, 129.2, 127.8, 127.7, 126.6, 120.8, 114.0, 113.0, 104.4, 90.8, 77.9, 55.0, 49.0, -0.0; **HRMS (ESI$^+$) [M+Na]$^+$** Calcd for $C_{20}H_{24}O_2NaSi$: 347.1438 m/z, Found: 347.1435 m/z; **Specific rotation**: $[\alpha]_D^{20}$ -14.81 (*c* 2.00, $CHCl_3$) for an enantiomerically enriched sample of 98:2 e.r.

Enantiomeric purity of **6i** was determined by SFC analysis in comparison with authentic racemic material (98:2 e.r. shown; Chiralpak IC column, 92:8 CO_2 / *i*-PrOH, 0.8 mL/min, 220 nm).

<Sample Information>

Sample Name : WL-13-6-rac-IC.lcd
Sample ID :
Data Filename : WL-13-6-rac-IC.lcd
Method Filename : wl-1-92-8-0.8-35xmin.lcm
Batch Filename : lwx_hydrosilylation.lcb
Vial # : 1-21
Injection Volume : 1 uL
Date Acquired : 10/6/2022 8:14:44 PM
Date Processed : 11/24/2022 2:29:53 PM
Sample Type : Unknown
Acquired by : System Administrator
Processed by : System Administrator

<Chromatogram>

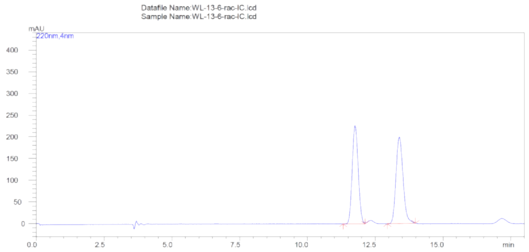

<Peak Table>

PDA Ch2 220nm

Peak#	Ret. Time	Area	Area%
1	11.758	3513962	49.362
2	13.389	3604859	50.638
Total		7118821	100.000

<Sample Information>

Sample Name : WL-13-6-CHIR-IC.lcd
Sample ID :
Data Filename : WL-13-6-CHIR-IC.lcd
Method Filename : wl-1-92-8-0.8-35xmin.lcm
Batch Filename : lwx_hydrosilylation.lcb
Vial # : 1-76
Injection Volume : 1 uL
Date Acquired : 10/6/2022 8:58:11 PM
Date Processed : 11/24/2022 2:28:23 PM
Sample Type : Unknown
Acquired by : System Administrator
Processed by : System Administrator

<Chromatogram>

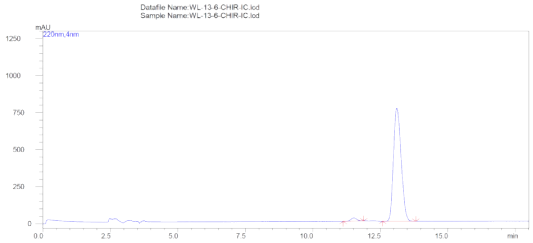

<Peak Table>

PDA Ch2 220nm

Peak#	Ret. Time	Area	Area%
1	11.527	322274	2.091
2	13.112	15093627	97.909
Total		15415901	100.000

(1*R*,2*R*)-2-(naphthalen-2-yl)-1-phenyl-4-(trimethylsilyl)but-3-yn-1-ol (6j)

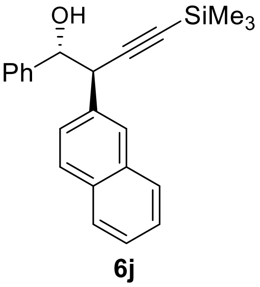

6j

White solid, m.p. 91–93 ºC; **IR (neat):** 3448 (w), 2173 (w), 1249 (m), 1037 (m), 844 (s), 760 (m), 700 (m) cm^{-1}; **^{1}H NMR (400 MHz, CDCl$_3$):** δ 7.81 – 7.74 (m, 1H), 7.74 – 7.66 (m, 2H), 7.63 (s, 1H), 7.53 – 7.35 (m, 2H), 7.33 – 7.09 (m, 6H), 4.84 (d, *J* = 5.6 Hz, 1H), 4.10 (d, *J* = 6.4 Hz, 1H), 2.78 (s, 1H), 0.22 (s, 9H); **^{13}C NMR (100 MHz, CDCl$_3$):** δ 140.5, 134.7, 133.1, 132.6, 127.8, 127.8, 127.8, 127.5, 126.6, 126.4, 126.0, 125.8, 104.4, 91.0, 77.8, 49.1, -0.0; **HRMS (ESI$^+$) [M+Na]$^+$** Calcd for $C_{23}H_{24}ONaSi$: 367.1489 m/z, Found: 367.1483 m/z; **Specific rotation**: $[\alpha]_D^{20}$ -37.13 (*c* 2.00, $CHCl_3$) for an enantiomerically enriched sample of 97:3 e.r.

Enantiomeric purity of **6j** was determined by SFC analysis in comparison with authentic racemic material (97:3 e.r. shown; Chiralpak IJ-3 column, 92:8 CO_2 / *i*-PrOH, 0.8 mL/min, 220 nm).

<Sample Information>

Sample Name : WL-13-8-rac-ash.lcd
Sample ID :
Data Filename : WL-13-8-rac-IJ3.lcd
Method Filename : wl-6-92-8-0.8-55xmin.lcm
Batch Filename : lwx_hydrosilylation.lcb
Vial # : 1-23 Sample Type : Unknown
Injection Volume : 1 uL
Date Acquired : 10/7/2022 1:54:03 PM Acquired by : System Administrator
Date Processed : 11/24/2022 2:47:54 PM Processed by : System Administrator

<Chromatogram>

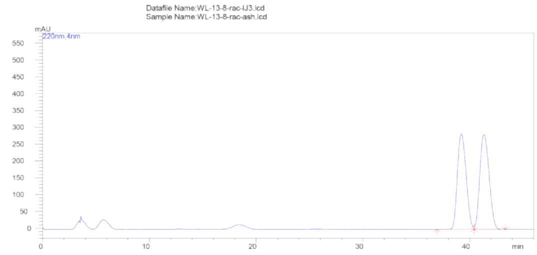

<Peak Table>

PDA Ch2 220nm

Peak#	Ret. Time	Area	Area%
1	39.229	15815886	49.252
2	41.350	16296477	50.748
Total		32112362	100.000

<Sample Information>

Sample Name : WL-13-8-CHIR-IJ3.lcd
Sample ID :
Data Filename : WL-13-8-CHIR-IJ3.lcd
Method Filename : wl-6-92-8-0.8-55xmin.lcm
Batch Filename : lwx_hydrosilylation.lcb
Vial # : 1-78 Sample Type : Unknown
Injection Volume : 1 uL
Date Acquired : 10/7/2022 7:13:13 PM Acquired by : System Administrator
Date Processed : 11/24/2022 2:49:13 PM Processed by : System Administrator

<Chromatogram>

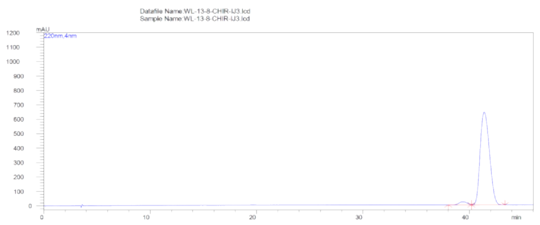

<Peak Table>

PDA Ch2 220nm

Peak#	Ret. Time	Area	Area%
1	39.383	1167664	2.906
2	41.396	39015250	97.094
Total		40182914	100.000

(1*R*,2*R*)-1-phenyl-2-(*o*-tolyl)-4-(trimethylsilyl)but-3-yn-1-ol (6k)

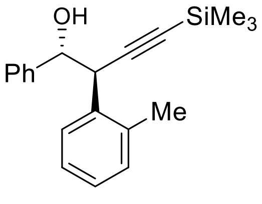

6k

Colorless oil. **IR (neat):** 3514 (w), 2172 (w), 1492 (w), 1249 (m), 842 (s), 762 (m), 699 (m) cm^{-1}; **^{1}H NMR (400 MHz, $CDCl_3$):** δ 7.54 (d, *J* = 7.6 Hz, 1H), 7.26 – 7.18 (m, 4H), 7.18 – 7.09 (m, 3H), 7.00 (d, *J* = 7.6 Hz, 1H), 4.71 (dd, *J* = 6.4, 3.6 Hz, 1H), 4.11 (d, *J* = 6.4 Hz, 1H), 2.90 (d, *J* = 3.6 Hz, 1H), 1.91 (s, 3H), 0.19 (s, 9H); **^{13}C NMR (100 MHz, $CDCl_3$) :** δ 140.6, 136.0, 135.7, 130.3, 128.8, 127.8, 127.7, 127.2, 126.3, 126.1, 105.1, 89.9, 76.9, 45.2, 19.0, -0.0; **HRMS (ESI$^+$) [M+Na]$^+$** Calcd for $C_{20}H_{24}ONaSi$: 331.1489 m/z, Found: 331.1490 m/z; **Specific rotation**: $[\alpha]_D^{20}$ -5.16 (*c* 2.00, $CHCl_3$) for an enantiomerically enriched sample of 96:4 e.r.

Enantiomeric purity of **6k** was determined by SFC analysis in comparison with authentic racemic material (96:4 e.r. shown; Chiralpak IC column, 97:3 CO_2 / *i*-PrOH, 0.8 mL/min, 220 nm).

<Sample Information>

Sample Name : WL-12-82-RAC-IC.lcd
Sample ID :
Data Filename : WL-12-82-RAC-IC.lcd
Method Filename : wl-1-97-3-0.8-35xmin.lcm
Batch Filename : lwx_hydrosilylation.lcb
Vial # : 1-20 Sample Type : Unknown
Injection Volume : 2 uL
Date Acquired : 9/17/2022 5:47:40 AM Acquired by : System Administrator
Date Processed : 12/23/2022 10:47:28 AM Processed by : System Administrator

<Chromatogram>

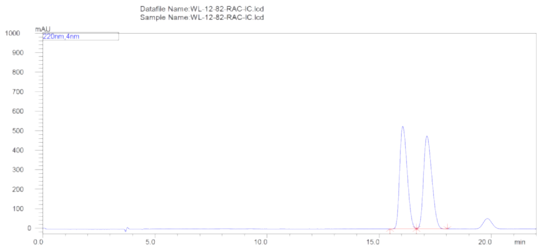

<Peak Table>

PDA Ch2 220nm

Peak#	Ret. Time	Area	Area%
1	16.048	12292327	49.821
2	17.127	12380496	50.179
Total		24672823	100.000

<Sample Information>

Sample Name : WL-12-82-CHIR-IC.lcd
Sample ID :
Data Filename : WL-12-82-CHIR-IC.lcd
Method Filename : wl-1-97-3-0.8-35xmin.lcm
Batch Filename : lwx_hydrosilylation.lcb
Vial # : 1-22 Sample Type : Unknown
Injection Volume : 2 uL
Date Acquired : 9/17/2022 11:44:52 AM Acquired by : System Administrator
Date Processed : 12/23/2022 10:45:39 AM Processed by : System Administrator

<Chromatogram>

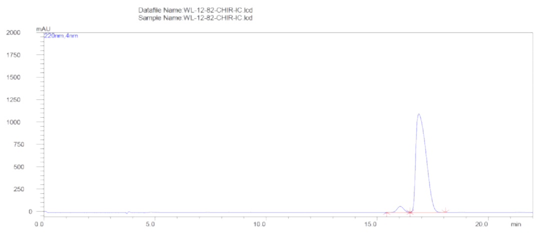

<Peak Table>

PDA Ch2 220nm

Peak#	Ret. Time	Area	Area%
1	16.038	1397456	3.821
2	16.871	35175776	96.179
Total		36573232	100.000

(1*R*,2*R*)-2-(naphthalen-1-yl)-1-phenyl-4-(trimethylsilyl)but-3-yn-1-ol (6l)

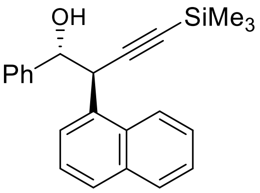

6l

White solid, m.p. 64–66 ºC; **IR (neat):** 3334 (w), 2176 (w), 1250 (m), 1047 (m), 841 (s), 758 (m), 698 (m) cm^{-1}; **^{1}H NMR (400 MHz, $CDCl_3$):** δ 8.10 – 7.97 (m, 1H), 7.93 – 7.83 (m, 1H), 7.81 – 7.72 (m, 2H), 7.56 – 7.44 (m, 3H), 7.43 – 7.31 (m, 2H), 7.31 – 7.19 (m, 3H), 5.03 (t, *J* = 4.4 Hz, 1H), 4.80 (d, *J* = 4.8 Hz, 1H), 2.71 (d, *J* = 4.4 Hz, 1H), 0.25 (s, 9H); **^{13}C NMR (100 MHz, $CDCl_3$):** δ 141.3, 133.9, 133.6, 130.7, 129.0, 128.2, 127.9, 127.7, 127.0, 126.2, 126.0, 125.5, 125.3, 122.7, 104.2, 91.2, 75.9, 45.2, -0.0; **HRMS (ESI$^+$) [M+Na]$^+$** Calcd for $C_{23}H_{24}ONaSi$: 367.1489 m/z, Found: 367.1487 m/z; **Specific rotation**: $[\alpha]_D^{20}$ -7.34 (*c* 2.00, $CHCl_3$) for an enantiomerically enriched sample of 96:4 e.r.,

Enantiomeric purity of **6l** was determined by SFC analysis in comparison with authentic racemic material (96:4 e.r. shown; Chiralpak ID column, 92:8 CO_2 / *i*-PrOH, 0.8 mL/min, 220 nm).

<Sample Information>

Sample Name : WL-13-9-rac-ID.lcd
Sample ID :
Data Filename : WL-13-9-rac2-ID.lcd
Method Filename : wl-2-92-8-0.8-55xmin.lcm
Batch Filename : lwx_hydrosilylation.lcb
Vial # : 1-24
Injection Volume : 1 uL
Date Acquired : 10/6/2022 3:54:20 PM
Date Processed : 11/24/2022 2:54:26 PM
Sample Type : Unknown
Acquired by : System Administrator
Processed by : System Administrator

<Chromatogram>

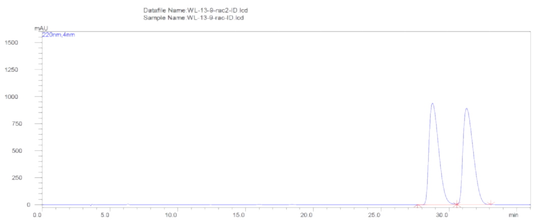

<Peak Table>

PDA Ch2 220nm

Peak#	Ret. Time	Area	Area%
1	28.782	46503220	49.972
2	31.295	46556225	50.028
Total		93059445	100.000

<Sample Information>

Sample Name : WL-13-9-chir-ID.lcd
Sample ID :
Data Filename : WL-13-9-chir-ID.lcd
Method Filename : wl-2-92-8-0.8-55xmin.lcm
Batch Filename : lwx_hydrosilylation.lcb
Vial # : 1-79
Injection Volume : 1 uL
Date Acquired : 10/6/2022 4:50:01 PM
Date Processed : 11/24/2022 2:57:29 PM
Sample Type : Unknown
Acquired by : System Administrator
Processed by : System Administrator

<Chromatogram>

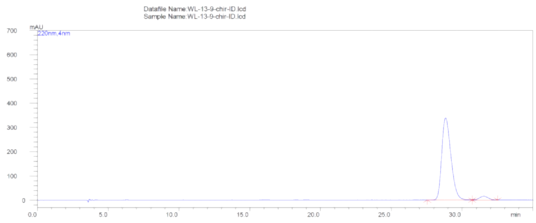

<Peak Table>

PDA Ch2 220nm

Peak#	Ret. Time	Area	Area%
1	28.833	15385822	95.654
2	31.538	699115	4.346
Total		16084936	100.000

(1*R*,2*S*)-2-(furan-3-yl)-1-phenyl-4-(trimethylsilyl)but-3-yn-1-ol (6m)

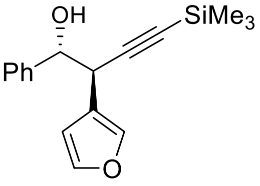

6m

Colorless oil. **IR (neat):** 3416 (w), 2177 (w), 1250 (m), 1022 (m), 842 (s), 760 (m), 688 (m) cm^{-1}; **^{1}H NMR (400 MHz, $CDCl_3$):** δ 7.51 – 7.23 (m, 6H), 7.19 (s, 1H), 6.07 (d, *J* = 2.0 Hz, 1H), 4.71 (dd, *J* = 6.4, 2.8 Hz, 1H), 3.88 (d, *J* = 6.4 Hz, 1H), 2.72 (d, *J* = 3.2 Hz, 1H), 0.19 (s, 9H); **^{13}C NMR (100 MHz, $CDCl_3$):** δ 142.8, 140.8, 140.5, 128.0, 127.9, 126.8, 121.6, 110.3, 103.8, 89.5, 76.8, 39.4, -0.1; **HRMS (EI$^+$) [M-H_2O]$^+$**Calcd for $C_{17}H_{18}OSi$: 266.1121 m/z, Found: 266.1125 m/z; **Specific rotation**: $[\alpha]_D^{20}$ 10.34 (*c* 2.00, $CHCl_3$) for an enantiomerically enriched sample of 98:2 e.r.,

Enantiomeric purity of **6m** was determined by SFC analysis in comparison with authentic racemic material (98:2 e.r. shown; Chiralpak ID column, 89:11 CO_2 / *i*-PrOH, 0.8 mL/min, 220 nm).

<Sample Information>

Sample Name : WL-13-4-RAC-ID.lcd
Sample ID :
Data Filename : WL-13-4-RACzx-ID.lcd
Method Filename : wl-1-91-9-0.8-35xmin.lcm
Batch Filename : lwx_hydrosilylation.lcb
Vial # : 1-80 Sample Type : Unknown
Injection Volume : 6 uL
Date Acquired : 10/5/2022 11:05:26 AM Acquired by : System Administrator
Date Processed : 10/5/2022 11:22:22 AM Processed by : System Administrator

<Chromatogram>

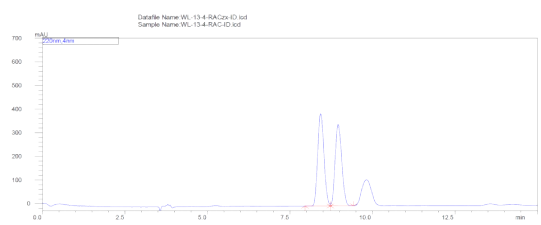

<Peak Table>

PDA Ch2 220nm

Peak#	Ret. Time	Area	Area%
1	8.429	4974216	50.910
2	8.960	4796362	49.090
Total		9770578	100.000

<Sample Information>

Sample Name : WL-13-4-chirzx-ID.lcd
Sample ID :
Data Filename : WL-13-4-chirzx-ID.lcd
Method Filename : wl-3-89-11-0.8-20xmin.lcm
Batch Filename : lwx_hydrosilylation.lcb
Vial # : 1-19 Sample Type : Unknown
Injection Volume : 1 uL
Date Acquired : 10/5/2022 12:58:01 PM Acquired by : System Administrator
Date Processed : 11/24/2022 9:17:29 AM Processed by : System Administrator

<Chromatogram>

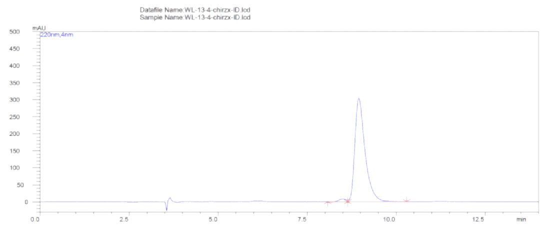

<Peak Table>

PDA Ch2 220nm

Peak#	Ret. Time	Area	Area%
1	8.502	114429	1.858
2	8.953	6043020	98.142
Total		6157449	100.000

(1*R*,2*R*)-1-phenyl-2-(thiophen-3-yl)-4-(trimethylsilyl)but-3-yn-1-ol (6n)

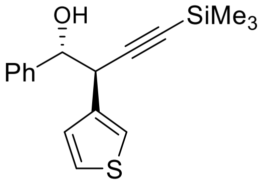

6n

Colorless oil. **IR (neat):** 3340 (w), 2177 (w), 1249 (m), 1043 (m), 841 (s), 760 (m), 699 (m) cm^{-1}; **^{1}H NMR (400 MHz, $CDCl_3$):** δ 7.33 – 7.23 (m, 3H), 7.23 – 7.15 (m, 3H), 7.01 (s, 1H), 6.81 (d, *J* = 4.4 Hz, 1H), 4.77 (d, *J* = 6.4 Hz, 1H), 4.05 (d, *J* = 6.4 Hz, 1H), 2.70 (s, 1H), 0.20 (s, 9H); **^{13}C NMR (100 MHz, $CDCl_3$):** δ 140.6, 137.6, 127.8, 127.5, 126.6, 125.4, 123.0, 104.2, 90.1, 77.2, 44.1, -0.1; **HRMS (ESI$^+$) [M+Na]$^+$** Calcd for $C_{17}H_{20}ONaSiS$: 323.0896 m/z, Found: 323.0896 m/z; **Specific rotation**: $[\alpha]_D^{20}$ -11.31 (*c* 2.00, $CHCl_3$) for an enantiomerically enriched sample of 97:3 e.r.,

Enantiomeric purity of **6n** was determined by SFC analysis in comparison with authentic racemic material (97:3 e.r. shown; Chiralpak IF column, 95:5 CO_2 / *i*-PrOH, 0.8 mL/min, 220 nm).

<Sample Information>

Sample Name : WL-12-95-RA2C-IF.lcd
Sample ID :
Data Filename : WL-12-95-RA-IF.lcd
Method Filename : wl-6-95-5-0.8-35xmin.lcm
Batch Filename : lwx_hydrosilylation.lcb
Vial # : 1-77 Sample Type : Unknown
Injection Volume : 2 uL
Date Acquired : 9/21/2022 4:54:45 PM Acquired by : System Administrator
Date Processed : 11/23/2022 9:57:41 PM Processed by : System Administrator

<Chromatogram>

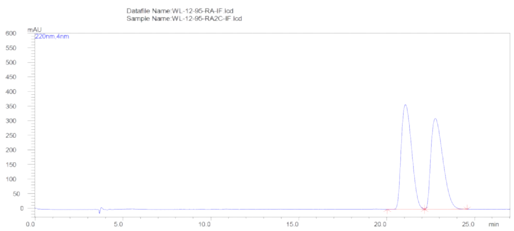

<Peak Table>

PDA Ch2 220nm

Peak#	Ret. Time	Area	Area%
1	21.061	14792072	49.962
2	22.763	14814832	50.038
Total		29606904	100.000

<Sample Information>

Sample Name : WL-12-95-CHIR-IF.lcd
Sample ID :
Data Filename : WL-12-95-CHIRX-IF001.lcd
Method Filename : wl-6-95-5-0.8-35xmin.lcm
Batch Filename : lwx_hydrosilylation.lcb
Vial # : 1-80 Sample Type : Unknown
Injection Volume : 2 uL
Date Acquired : 9/21/2022 10:33:03 PM Acquired by : System Administrator
Date Processed : 11/23/2022 9:59:07 PM Processed by : System Administrator

<Chromatogram>

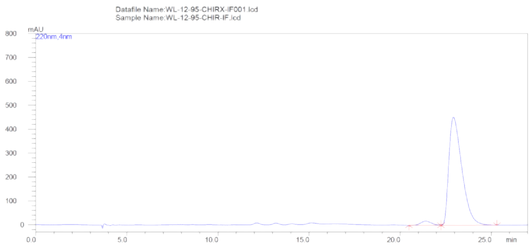

<Peak Table>

PDA Ch2 220nm

Peak#	Ret. Time	Area	Area%
1	21.421	699837	3.014
2	22.927	22521474	96.986
Total		23221310	100.000

(1*R*,2*R*)-1-phenyl-2-(pyridin-3-yl)-4-(trimethylsilyl)but-3-yn-1-ol (6o)

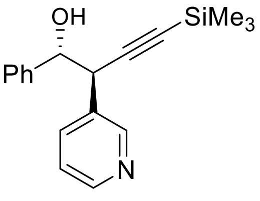

6o

White solid, m.p. 97–99 ºC; **IR (neat):** 3229 (w), 2182 (w), 1322 (m), 1062 (m), 844 (s), 760 (m), 699 (m) cm^{-1}; **^{1}H NMR (400 MHz, $CDCl_3$):** δ 8.35 (d, *J* = 4.8 Hz, 1H), 8.27 (s, 1H), 7.43 (d, *J* = 8.0 Hz, 1H), 7.30 – 7.15 (m, 3H), 7.19 – 7.02 (m, 3H), 4.80 (d, *J* = 6.0 Hz, 1H), 3.97 (d, *J* = 6.0 Hz, 1H), 3.72 (s, 1H), 0.19 (s, 9H); **^{13}C NMR (100 MHz, $CDCl_3$):** δ 149.8, 148.1, 140.2, 136.4, 133.2, 128.0, 127.8, 126.7, 122.9, 103.5, 91.1, 77.4, 46.0, -0.1; **HRMS (ESI$^+$) [M+H]$^+$** Calcd for $C_{18}H_{22}NOSi$: 296.1465 m/z, Found: 296.1468 m/z; **Specific rotation**: $[\alpha]_D^{20}$ -12.82 (*c* 1.00, $CHCl_3$) for an enantiomerically enriched sample of 95:5 e.r.

Enantiomeric purity of **6o** was determined by HPLC analysis in comparison with authentic racemic material (95:5 e.r. shown; Chiralpak IG column, 92:8 *n*-hexane / *i*-PrOH, 0.8 mL/min, 220 nm).

<Sample Information>

Sample Name : wanglei-13-14-RAC-IG.lcd
Sample ID :
Data Filename : wanglei-13-14-RACzX-IG.lcd
Method Filename : wanglei6hao-92-8-0.8ml-25minX.lcm
Batch Filename : WWLL1.lcb
Vial # : 1-91 Sample Type : Unknown
Injection Volume : 2 uL
Date Acquired : 10/15/2022 3:43:21 PM Acquired by : System Administrator
Date Processed : 10/15/2022 4:08:26 PM Processed by : System Administrator

<Chromatogram>

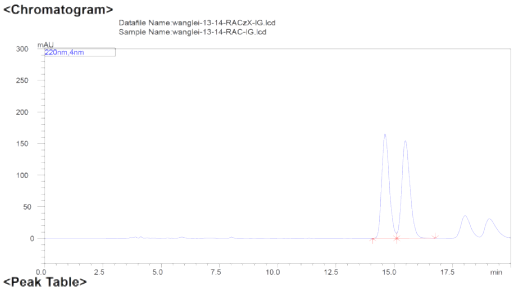

<Peak Table>

PDA Ch2 220nm

Peak#	Ret. Time	Area	Area%
1	14.607	3730577	49.572
2	15.479	3795023	50.428
Total		7525600	100.000

<Sample Information>

Sample Name : wanglei-13-14-chir-IG.lcd
Sample ID :
Data Filename : wanglei-13-14-chir-IG.lcd
Method Filename : wanglei6hao-92-8-0.8ml-25minX.lcm
Batch Filename : WWLL1.lcb
Vial # : 1-95 Sample Type : Unknown
Injection Volume : 1 uL
Date Acquired : 10/15/2022 4:08:54 PM Acquired by : System Administrator
Date Processed : 10/15/2022 4:33:58 PM Processed by : System Administrator

<Chromatogram>

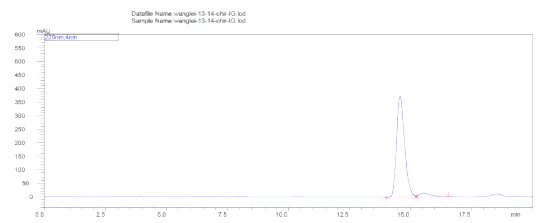

<Peak Table>

PDA Ch2 220nm

Peak#	Ret. Time	Area	Area%
1	14.580	8549822	95.062
2	15.545	444078	4.938
Total		8993900	100.000

(1*R*,2*S*)-2-(benzofuran-2-yl)-1-phenyl-4-(trimethylsilyl)but-3-yn-1-ol (6p)

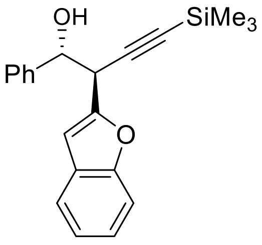

6p

White solid, m.p. 56–58 ºC; **IR (neat):** 3423 (w), 2177 (w), 1454 (m), 1250 (m), 845 (s), 762 (m), 702 (m) cm^{-1}; **^{1}H NMR (400 MHz, $CDCl_3$):** δ7.50 (d, *J* = 7.6 Hz, 1H), 7.44 (d, *J* = 7.6 Hz, 1H), 7.40 – 7.33 (m, 2H), 7.34 – 7.27 (m, 3H), 7.26 – 7.15 (m, 2H), 6.63 (s, 1H), 5.17 (t, *J* = 4.8 Hz, 1H), 4.20 (d, *J* = 4.0 Hz, 1H), 2.68 (d, *J* = 5.2 Hz, 1H), 0.20 (s, 9H); **^{13}C NMR (100 MHz, $CDCl_3$):** δ 155.0, 154.0, 140.6, 128.2, 128.0, 127.9, 126.2, 124.0, 122.8, 120.9, 111.1, 105.2, 100.3, 91.1, 74.6, 43.1, -0.1; **HRMS (ESI$^+$) [M+Na]$^+$** Calcd for $C_{21}H_{22}O_2NaSi$: 357.1281 m/z, Found: 357.1275 m/z; **Specific rotation**: $[\alpha]_D^{20}$ -9.46 (*c* 1.00, $CHCl_3$) for an enantiomerically enriched sample of 96.5:3.5 e.r.,

Enantiomeric purity of **6p** was determined by SFC analysis in comparison with authentic racemic material (96.5:3.5 e.r. shown; Chiralpak IC column, 91:9 CO_2 / *i*-PrOH, 1.0 mL/min, 220 nm).

<Sample Information>

Sample Name : WL-13-62-RAC-IC.lcd
Sample ID :
Data Filename : WL-13-62-RAC2-IC.lcd
Method Filename : wl-1-91-9-1.0-35xmin.lcm
Batch Filename : lwx_hydrosilylation.lcb
Vial # : 1-91 Sample Type : Unknown
Injection Volume : 3 uL
Date Acquired : 11/2/2022 12:35:12 PM Acquired by : System Administrator
Date Processed : 11/2/2022 1:31:01 PM Processed by : System Administrator

<Chromatogram>

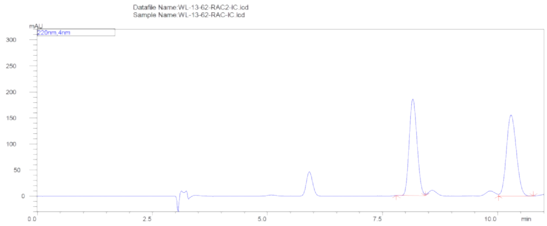

<Peak Table>

PDA Ch2 220nm

Peak#	Ret. Time	Area	Area%
1	8.156	2140491	49.229
2	10.288	2207578	50.771
Total		4348069	100.000

<Sample Information>

Sample Name : WL-13-62-CHIR-IC.lcd
Sample ID :
Data Filename : WL-13-62-CHIR-IC.lcd
Method Filename : wl-1-91-9-1.0-35xmin.lcm
Batch Filename : lwx_hydrosilylation.lcb
Vial # : 1-94 Sample Type : Unknown
Injection Volume : 2 uL
Date Acquired : 11/2/2022 10:32:50 AM Acquired by : System Administrator
Date Processed : 11/24/2022 4:36:19 PM Processed by : System Administrator

<Chromatogram>

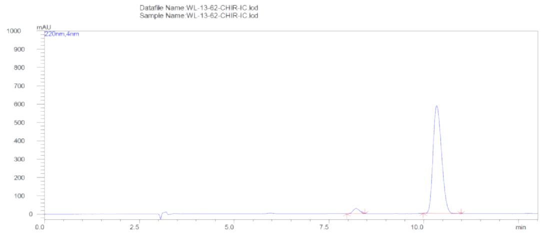

<Peak Table>

PDA Ch2 220nm

Peak#	Ret. Time	Area	Area%
1	8.214	334636	3.515
2	10.340	9185701	96.485
Total		9520337	100.000

(1*R*,2*S*)-2-(benzo[*b*]thiophen-2-yl)-1-phenyl-4-(trimethylsilyl)but-3-yn-1-ol (6q)

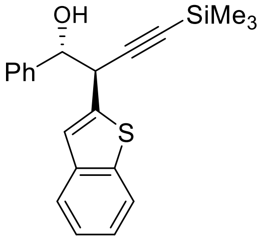

6q

White solid, m.p. 91–93 °C; **IR (neat):** 3446 (w), 2175 (w), 1249 (m), 1024 (m), 843 (s), 761 (m), 701 (m) cm^{-1}; **^{1}H NMR (400 MHz, $CDCl_3$):** δ 7.75 (d, *J* = 7.2 Hz, 1H), 7.66 – 7.60 (m, 1H), 7.38 – 7.20 (m, 7H), 7.03 (s, 1H), 4.94 (d, *J* = 6.0 Hz, 1H), 4.29 (d, *J* = 5.6 Hz, 1H), 2.76 (s, 1H), 0.21 (s, 9H); **^{13}C NMR (100 MHz, $CDCl_3$):** δ 141.1, 140.2, 139.7, 139.3, 128.1, 128.0, 126.6, 124.2, 124.1, 123.3, 123.1, 122.1, 102.8, 91.3, 77.3, 44.7, -0.1; **HRMS (ESI$^+$) [M+Na]$^+$** Calcd for $C_{21}H_{22}ONaSiS$: 373.1053 m/z, Found: 373.1051 m/z; **Specific rotation**: $[\alpha]_D^{20}$ -14.86 (*c* 2.00, $CHCl_3$) for an enantiomerically enriched sample of 96:4 e.r.,

Enantiomeric purity of **6q** was determined by SFC analysis in comparison with authentic racemic material (96:4 e.r. shown; Chiralpak IC column, 91:9 CO_2 / *i*-PrOH, 1.0 mL/min, 220 nm).

<Sample Information>

Sample Name : WL-13-63-RAC-IC.lcd
Sample ID :
Data Filename : WL-13-63-RAC-IC.lcd
Method Filename : wl-1-91-9-1.0-35xmin.lcm
Batch Filename : lwx_hydrosilylation.lcb
Vial # : 1-92 Sample Type : Unknown
Injection Volume : 1 uL
Date Acquired : 11/2/2022 3:48:43 AM Acquired by : System Administrator
Date Processed : 11/4/2022 7:28:18 PM Processed by : System Administrator

<Chromatogram>

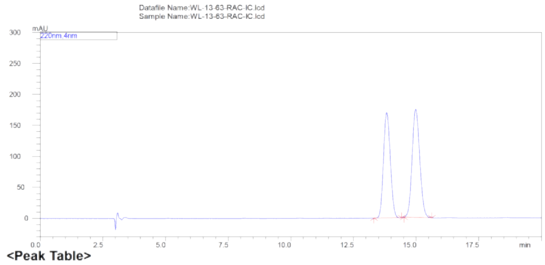

<Peak Table>

PDA Ch2 220nm

Peak#	Ret. Time	Area	Area%
1	13.826	3218551	49.138
2	14.983	3331516	50.862
Total		6550066	100.000

<Sample Information>

Sample Name : WL-13-63-CHIR-IC.lcd
Sample ID :
Data Filename : WL-13-63-CHIR-IC.lcd
Method Filename : wl-1-91-9-1.0-35xmin.lcm
Batch Filename : lwx_hydrosilylation.lcb
Vial # : 1-95 Sample Type : Unknown
Injection Volume : 2 uL
Date Acquired : 11/2/2022 11:08:37 AM Acquired by : System Administrator
Date Processed : 11/2/2022 1:32:43 PM Processed by : System Administrator

<Chromatogram>

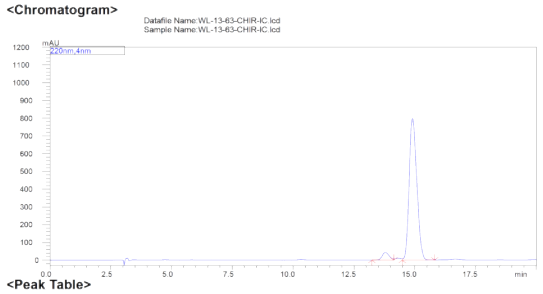

<Peak Table>

PDA Ch2 220nm

Peak#	Ret. Time	Area	Area%
1	13.797	869653	4.723
2	14.909	17545046	95.277
Total		18414699	100.000

***tert*-butyl 6-((1*R*,2*S*)-1-hydroxy-1-phenyl-4-(trimethylsilyl)but-3-yn-2-yl)-1H-indole-1-carboxylate (6r)**

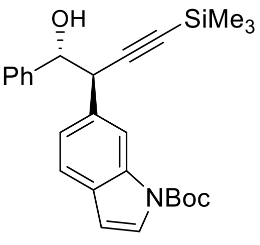

6r

Colorless oil. **IR (neat):** 3349 (w), 2172 (w), 1773 (s), 1337 (m), 1146 (m), 842 (s), 761 (m), 700 (m) cm^{-1}; **^{1}H NMR (400 MHz, $CDCl_3$):** δ 8.01 (s, 1H), 7.56 (d, *J* = 3.6 Hz, 1H), 7.41 (d, *J* = 8.0 Hz, 1H), 7.30 – 7.14 (m, 5H), 7.02 (dd, *J* = 8.0, 1.6 Hz, 1H), 6.51 (d, *J* = 3.6 Hz, 1H), 4.82 (d, *J* = 6.4 Hz, 1H), 4.07 (d, *J* = 6.4 Hz, 1H), 2.79 (s, 1H), 1.64 (s, 9H), 0.21 (s, 9H); **^{13}C NMR (100 MHz, $CDCl_3$):** δ 149.6, 140.7, 135.0, 133.5, 129.8, 127.8, 127.6, 126.6, 126.1, 123.2, 120.5, 115.4, 107.0, 105.0, 90.4, 83.5, 78.1, 49.4, 28.1, -0.0; **HRMS (ESI^+) $[M+Na]^+$** Calcd for $C_{26}H_{31}NO_3NaSi$: 456.1965 m/z, Found: 456.1962 m/z; **Specific rotation**: $[\alpha]_D^{20}$ -0.38 (*c* 2.00, $CHCl_3$) for an enantiomerically enriched sample of 97:3 e.r.,

Enantiomeric purity of **6r** was determined by SFC analysis in comparison with authentic racemic material (97:3 e.r. shown; Chiralpak IC column, 91:9 CO_2 / *i*-PrOH, 1.0 mL/min, 220 nm).

<Sample Information>

Sample Name : WL-13-64-RAC2-IC.lcd
Sample ID :
Data Filename : WL-13-64-RACzx-IC001.lcd
Method Filename : wl-1-91-9-1.0-35xmin.lcm
Batch Filename : lwx_hydrosilylation.lcb
Vial # : 1-95 Sample Type : Unknown
Injection Volume : 2 uL
Date Acquired : 11/4/2022 10:12:12 PM Acquired by : System Administrator
Date Processed : 11/5/2022 9:08:49 AM Processed by : System Administrator

<Chromatogram>

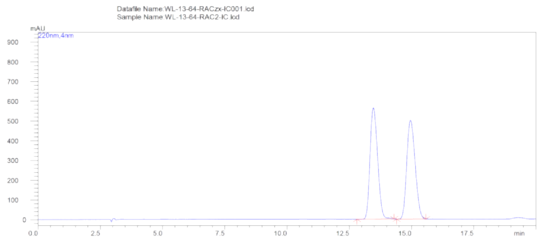

<Peak Table>

PDA Ch2 220nm

Peak#	Ret. Time	Area	Area%
1	13.469	11960847	50.440
2	14.960	11752403	49.560
Total		23713250	100.000

<Sample Information>

Sample Name : WL-13-64-chir-IC.lcd
Sample ID :
Data Filename : WL-13-64-chirzx-IC001.lcd
Method Filename : wl-1-91-9-1.0-35xmin.lcm
Batch Filename : lwx_hydrosilylation.lcb
Vial # : 1-93 Sample Type : Unknown
Injection Volume : 1 uL
Date Acquired : 11/5/2022 1:21:35 AM Acquired by : System Administrator
Date Processed : 11/5/2022 9:05:40 AM Processed by : System Administrator

<Chromatogram>

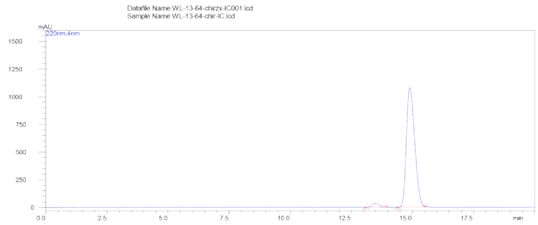

<Peak Table>

PDA Ch2 220nm

Peak#	Ret. Time	Area	Area%
1	13.484	693525	2.661
2	14.894	25371907	97.339
Total		26065432	100.000

(1*R*,2*R*)-4-(*tert*-butyldimethylsilyl)-1,2-diphenylbut-3-yn-1-ol (6s)

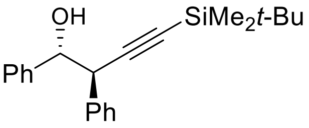

6s

White solid, m.p. 67–68 °C; **IR (neat):** 3376 (w), 2177 (w), 1249 (m), 1040 (m), 837 (s), 733 (m), 697 (m) cm^{-1}; **^{1}H NMR (400 MHz, $CDCl_3$):** δ 7.31 – 7.21 (m, 6H), 7.21 – 7.15 (m, 4H), 4.77 (dd, *J* = 6.4, 3.6 Hz, 1H), 3.99 (d, *J* = 6.4 Hz, 1H), 2.75 (d, *J* = 3.6 Hz, 1H), 0.97 (s, 9H), 0.16 (s, 6H); **^{13}C NMR (100 MHz, $CDCl_3$):** δ140.6, 137.4, 128.5, 128.2, 127.8, 127.7, 127.2, 126.6, 105.0, 89.0, 78.0, 49.0, 26.1, 16.5, -4.6; **HRMS (ESI^+) $[M+Na]^+$** Calcd for $C_{22}H_{28}ONaSi$: 359.1802 m/z, Found: 359.1796 m/z; **Specific rotation**: $[\alpha]_D^{20}$ -13.43 (*c* 2.00, $CHCl_3$) for an enantiomerically enriched sample of 99:1 e.r.

Enantiomeric purity of **6s** was determined by HPLC analysis in comparison with authentic racemic material (99:1 e.r. shown; Chiralpak IF column, 99.5:0.5 *n*-hexane / *i*-PrOH, 0.8 mL/min, 220 nm).

<Sample Information>

Sample Name : wanglei-13-54-rac-IF.lcd
Sample ID :
Data Filename : wanglei-13-54-razcx-IF.lcd
Method Filename : wanglei3hao-99.5-0.5-0.8ml-40minX.lcm
Batch Filename : WWLL1.lcb
Vial # : 1-33 Sample Type : Unknown
Injection Volume : 1 uL
Date Acquired : 10/29/2022 12:02:35 AM Acquired by : System Administrator
Date Processed : 11/22/2022 9:45:25 AM Processed by : System Administrator

<Chromatogram>

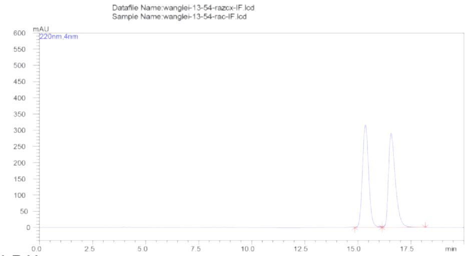

<Peak Table>

PDA Ch2 220nm

Peak#	Ret. Time	Area	Area%
1	15.358	6475563	49.676
2	16.567	6559979	50.324
Total		13035541	100.000

<Sample Information>

Sample Name : wanglei-13-54-chir-IF.lcd
Sample ID :
Data Filename : wanglei-13-54-chix-IF.lcd
Method Filename : wanglei3hao-99.5-0.5-0.8ml-40minX.lcm
Batch Filename : WWLL1.lcb
Vial # : 1-34 Sample Type : Unknown
Injection Volume : 1 uL
Date Acquired : 10/28/2022 11:30:38 PM Acquired by : System Administrator
Date Processed : 10/29/2022 12:01:40 AM Processed by : System Administrator

<Chromatogram>

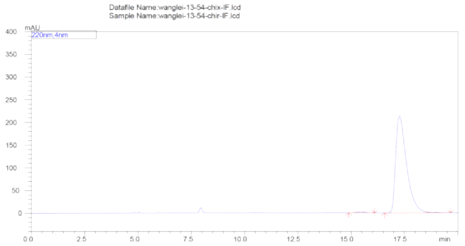

<Peak Table>

PDA Ch2 220nm

Peak#	Ret. Time	Area	Area%
1	15.446	55859	0.731
2	17.269	7586874	99.269
Total		7642733	100.000

(1*R*,2*R*)-4-cyclohexyl-1,2-diphenylbut-3-yn-1-ol (6t)

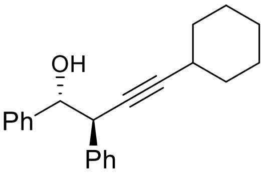

6t

Colorless oil. **IR (neat):** 3027 (w), 1540 (m), 1030 (w), 844 (s), 750 (m), 699 (s) cm^{-1}; **^{1}H NMR (400 MHz, $CDCl_3$):** δ 7.27 – 7.20 (m, 6H), 7.20 – 7.11 (m, 4H), 4.70 (dd, *J* = 6.4, 3.6 Hz, 1H), 3.91 (dd, *J* = 6.4, 2.0 Hz, 1H), 2.78 (d, *J* = 3.6 Hz, 1H), 2.59 – 2.36 (m, 1H), 2.05 – 1.77 (m, 2H), 1.76 – 1.64 (m, 2H), 1.54 – 1.41 (m, 3H), 1.39 – 1.24 (m, 3H); **^{13}C NMR (100 MHz, $CDCl_3$):** δ 140.9, 138.4, 128.5, 128.1, 127.8, 127.6, 127.1, 126.6, 90.9, 78.3, 77.9, 48.2, 32.8, 29.1, 25.8, 24.8; **HRMS (DART$^+$) [M+H]$^+$** Calcd for $C_{22}H_{25}O$: 305.1900 m/z, Found: 305.1905 m/z; **Specific rotation**: $[\alpha]_D^{20}$ 4.31 (*c* 1.00, $CHCl_3$) for an enantiomerically enriched sample of 89:11 e.r.

Enantiomeric purity of **6t** was determined by HPLC analysis in comparison with authentic racemic material (89:11 e.r. shown; Chiralpak IB column, 99.5:0.5 *n*-hexane / *i*-PrOH, 0.8 mL/min, 220 nm).

<Sample Information>

Sample Name : wanglei-13-46-RAC-iB.lcd
Sample ID :
Data Filename : wanglei-13-46-RAC-iB.lcd
Method Filename : 5-99.5-0.5-0.8ml-60minX.lcm
Batch Filename : WWLL1.lcb
Vial # : 1-18 Sample Type : Unknown
Injection Volume : 1 uL
Date Acquired : 10/25/2022 6:28:28 PM Acquired by : System Administrator
Date Processed : 11/22/2022 9:39:33 AM Processed by : System Administrator

<Chromatogram>

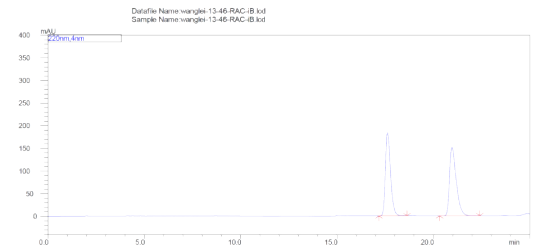

<Peak Table>

PDA Ch2 220nm

Peak#	Ret. Time	Area	Area%
1	17.617	3440967	49.489
2	20.962	3512068	50.511
Total		6953035	100.000

<Sample Information>

Sample Name : wanglei-13-46-CHIR-iB.lcd
Sample ID :
Data Filename : wanglei-13-46-CHIR-10MOLCAT-iB.lcd
Method Filename : 5-99.5-0.5-0.8ml-40minX.lcm
Batch Filename : WWLL1.lcb
Vial # : 1-32 Sample Type : Unknown
Injection Volume : 2 uL
Date Acquired : 10/26/2022 7:29:04 PM Acquired by : System Administrator
Date Processed : 10/26/2022 8:09:08 PM Processed by : System Administrator

<Chromatogram>

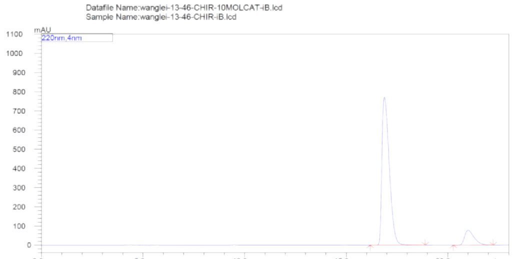

<Peak Table>

PDA Ch2 220nm

Peak#	Ret. Time	Area	Area%
1	16.880	19355993	89.299
2	21.004	2319449	10.701
Total		21675442	100.000

(1*R*,2*R*)-4-cyclopentyl-1,2-diphenylbut-3-yn-1-ol (6u).

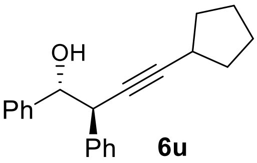

6u

Colorless oil. **IR (neat):** 3087 (w), 1535 (m), 1037 (w), 841 (s), 748 (m), 696 (s) cm^{-1}; **^{1}H NMR (400 MHz, $CDCl_3$):** δ 7.26 – 7.19 (m, 6H), 7.18 – 7.12 (m, 4H), 4.69 (dd, *J* = 6.0, 2.8 Hz, 1H), 3.89 (dd, *J* = 6.4, 2.0 Hz, 1H), 2.76 (d, *J* = 2.8 Hz, 1H), 2.69 (td, *J* = 7.2, 2.0 Hz, 1H), 1.98 – 1.87 (m, 2H), 1.77 – 1.68 (m, 2H), 1.67 – 1.51 (m, 4H); **^{13}C NMR (100 MHz, $CDCl_3$):** δ 140.9, 138.4, 128.4, 128.1, 127.8,

127.6, 127.0, 126.6, 91.0, 78.2, 77.4, 48.2, 34.0, 34.0, 30.3, 24.9; **HRMS (ESI$^+$) [M+Na]$^+$** Calcd for $C_{19}H_{20}ONa$: 287.1406 m/z, Found: 287.1418 m/z; **Specific rotation**: $[\alpha]_D^{20}$ -23.40 (*c* 1.00, $CHCl_3$) for an enantiomerically enriched sample of 88:12 e.r.

Enantiomeric purity of **6u** was determined by HPLC analysis in comparison with authentic racemic material (88:12 e.r. shown; Chiralpak IF column, 97:3 *n*-hexane / *i*-PrOH, 1.0 mL/min, 220 nm).

<Sample Information>

Sample Name : WL-15-7.lcd
Sample ID :
Data Filename : WL-15-7-rac-IF1.lcd
Method Filename : wanglei1hao-97-3-1.0ml-45minX.lcm
Batch Filename : WWLL1.lcb
Vial # : 1-16 Sample Type : Unknown
Injection Volume : 2 uL
Date Acquired : 5/2/2023 2:16:00 PM Acquired by : System Administrator
Date Processed : 5/2/2023 3:01:03 PM Processed by : System Administrator

<Chromatogram>

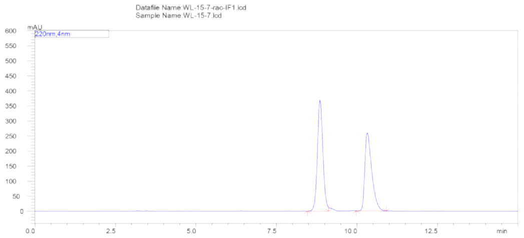

<Peak Table>

PDA Ch2 220nm

Peak#	Ret. Time	Area	Area%
1	8.854	4016413	50.717
2	10.324	3902881	49.283
Total		7919294	100.000

<Sample Information>

Sample Name : WL-15-7.lcd
Sample ID :
Data Filename : WL-15-7-chir-IF1.lcd
Method Filename : wanglei1hao-97-3-1.0ml-45minX.lcm
Batch Filename : WWLL1.lcb
Vial # : 1-17 Sample Type : Unknown
Injection Volume : 1 uL
Date Acquired : 5/2/2023 3:50:50 PM Acquired by : System Administrator
Date Processed : 5/2/2023 4:35:53 PM Processed by : System Administrator

<Chromatogram>

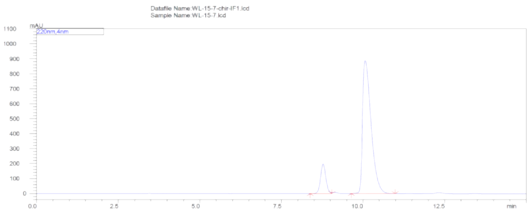

<Peak Table>

PDA Ch2 220nm

Peak#	Ret. Time	Area	Area%
1	8.777	2257200	12.250
2	10.067	16169657	87.750
Total		18426858	100.000

(1*R*,2*R*)-5-methyl-1,2-diphenylhex-3-yn-1-ol (6v)

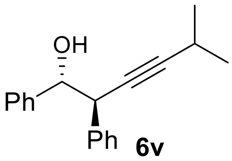

6v

Colorless oil. **IR (neat):** 3043 (w), 1543 (m), 1043 (w), 844 (s), 762 (m), 701 (s) cm^{-1}; **^{1}H NMR (400 MHz, $CDCl_3$):** δ 7.26 – 7.19 (m, 6H), 7.19 – 7.13 (m, 4H), 4.70 (dd, *J* = 6.0, 3.6 Hz, 1H), 3.88 (dd, *J* = 6.0, 1.6 Hz, 1H), 2.76 (d, *J* = 3.6 Hz, 1H), 2.74 – 2.44 (m, 1H), 1.21 (s, 3H), 1.19 (s, 3H); **^{13}C NMR (100 MHz, $CDCl_3$):** δ 140.9, 138.3, 128.4, 128.1, 127.8, 127.6, 127.1, 126.6, 92.2, 78.2, 77.2, 48.1, 23.2, 20.6; **HRMS (DART$^+$) [M+NH_4]$^+$** Calcd for $C_{19}H_{24}ON$: 282.1852 m/z, Found: 282.1850 m/z; **Specific rotation**: $[\alpha]_D^{20}$ -17.42 (*c* 1.00, $CHCl_3$) for an enantiomerically enriched sample of 90:10 e.r.

Enantiomeric purity of **6v** was determined by HPLC analysis in comparison with authentic racemic material (90:10 e.r. shown; Chiralpak IBN5 column, 99:1 *n*-hexane / *i*-PrOH, 1.0 mL/min, 220 nm).

<Sample Information>

Sample Name : WL-15-11.lcd
Sample ID :
Data Filename : WL-15-11-ibn5.lcd
Method Filename : wanglei4hao-99-1-1.0ml-45minX.lcm
Batch Filename : WWLL1.lcb
Vial # : 1-2 Sample Type : Unknown
Injection Volume : 1 uL
Date Acquired : 5/9/2023 5:20:10 PM Acquired by : System Administrator
Date Processed : 5/9/2023 5:47:39 PM Processed by : System Administrator

<Chromatogram>

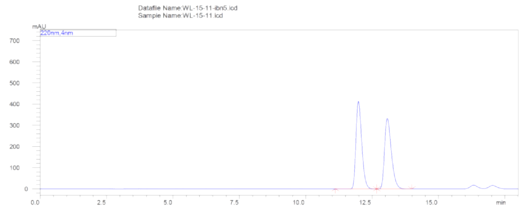

<Peak Table>

PDA Ch2 220nm

Peak#	Ret. Time	Area	Area%
1	11.983	6179807	50.960
2	13.072	5946941	49.040
Total		12126748	100.000

<Sample Information>

Sample Name : WL-15-11.lcd
Sample ID :
Data Filename : WL-15-11chir-ibn5.lcd
Method Filename : wanglei4hao-99-1-1.0ml-45minX.lcm
Batch Filename : WWLL1.lcb
Vial # : 1-3 Sample Type : Unknown
Injection Volume : 1 uL
Date Acquired : 5/9/2023 6:14:25 PM Acquired by : System Administrator
Date Processed : 5/9/2023 7:11:49 PM Processed by : System Administrator

<Chromatogram>

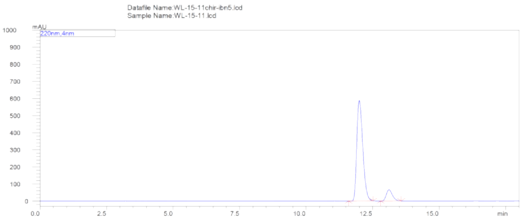

<Peak Table>

PDA Ch2 220nm

Peak#	Ret. Time	Area	Area%
1	11.996	9217295	89.810
2	13.113	1045845	10.190
Total		10263140	100.000

(1*R*,2*R*)-1,2-diphenylhept-3-yn-1-ol (6w)

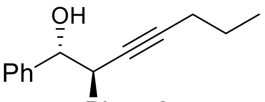

6w

Colorless oil. **IR (neat):** 3030 (w), 1549 (m), 1038 (w), 841 (s), 765 (m), 689 (s) cm^{-1}; **^{1}H NMR (400 MHz, $CDCl_3$):** δ 7.26 – 7.18 (m, 6H), 7.19 – 7.11 (m, 4H), 4.71 (d, J = 6.0 Hz, 1H), 3.90 (d, J = 6.4 Hz, 1H), 2.78 (s, 1H), 2.25 (t, J = 7.2 Hz, 2H), 1.56 (h, J = 7.2 Hz, 2H), 0.99 (t, J = 7.2 Hz, 3H); **^{13}C NMR (100 MHz, $CDCl_3$):** δ 140.9, 138.4, 128.4, 128.1, 127.8, 127.6, 127.1, 126.6, 86.3, 78.3, 78.2, 48.2, 22.3, 20.8, 13.5; **HRMS (ESI^{+}) [M+Na]$^{+}$** Calcd for $C_{19}H_{20}ONa$: 287.1406 m/z, Found: 287.1407 m/z; **Specific rotation**: $[\alpha]_D^{20}$ -1.74 (*c* 1.00, $CHCl_3$) for an enantiomerically enriched sample of 83:17 e.r.

Enantiomeric purity of **6w** was determined by HPLC analysis in comparison with authentic racemic material (83:17 e.r. shown; Chiralpak ODH column, 99:1 *n*-hexane / *i*-PrOH, 0.8 mL/min, 220 nm).

<Sample Information>

Sample Name : wanglei-13-25-CHIR2-ODH.lcd
Sample ID :
Data Filename : wanglei-13-25-CHIR3-ODH.lcd
Method Filename : 2-99-1-0.8ml-60minX.lcm
Batch Filename : WWLL1.lcb
Vial # : 1-95 Sample Type : Unknown
Injection Volume : 1 uL
Date Acquired : 10/20/2022 10:39:59 AM Acquired by : System Administrator
Date Processed : 10/20/2022 11:40:03 AM Processed by : System Administrator

<Chromatogram>

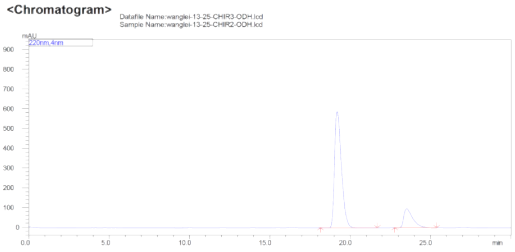

<Peak Table>

PDA Ch2 220nm

Peak#	Ret. Time	Area	Area%
1	19.194	17546871	82.737
2	23.521	3661132	17.263
Total		21208002	100.000

<Sample Information>

Sample Name : wanglei-13-25-RAC-ODH.lcd
Sample ID :
Data Filename : wanglei-13-25-RACX-ODH.lcd
Method Filename : 2-99-1-0.8ml-60minX.lcm
Batch Filename : WWLL1.lcb
Vial # : 1-91 Sample Type : Unknown
Injection Volume : 4 uL
Date Acquired : 10/20/2022 2:38:12 AM Acquired by : System Administrator
Date Processed : 10/20/2022 3:38:17 AM Processed by : System Administrator

<Chromatogram>

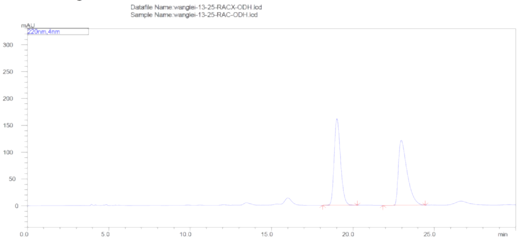

<Peak Table>

PDA Ch2 220nm

Peak#	Ret. Time	Area	Area%
1	19.020	4404084	49.281
2	22.972	4532605	50.719
Total		8936689	100.000

(1*R*,2*R*)-2-methyl-1-phenyl-4-(trimethylsilyl)but-3-yn-1-ol (6x)

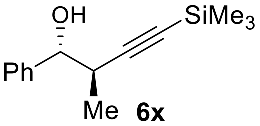

6x

Colorless oil. **^{1}H NMR (400 MHz, $CDCl_3$):** δ 7.45 – 7.37 (m, 4H), 7.38 – 7.33 (m, 1H), 4.52 (dd, *J* = 7.6, 3.6 Hz, 1H), 2.84 (p, *J* = 7.2 Hz, 1H), 2.68 (d, J = 3.6 Hz, 1H), 1.12 (d, *J* = 6.8 Hz, 3H), 0.23 (s, 9H); **^{13}C NMR (100 MHz, $CDCl_3$):** δ 141.2, 128.2, 127.9, 126.7, 107.6, 88.1, 77.3, 36.5, 17.2, 0.1; **Specific rotation**: $[\alpha]_D^{20}$ 57.16 (*c* 0.50, $CHCl_3$) for an enantiomerically enriched sample of 95.5:4.5 e.r.,

Enantiomeric purity of **6x** was determined by SFC analysis in comparison with authentic racemic material (95.5:4.5 e.r. shown; Chiralcel OZ-H column, 98:2 CO_2 / *i*-PrOH, 1.0 mL/min, 220 nm).

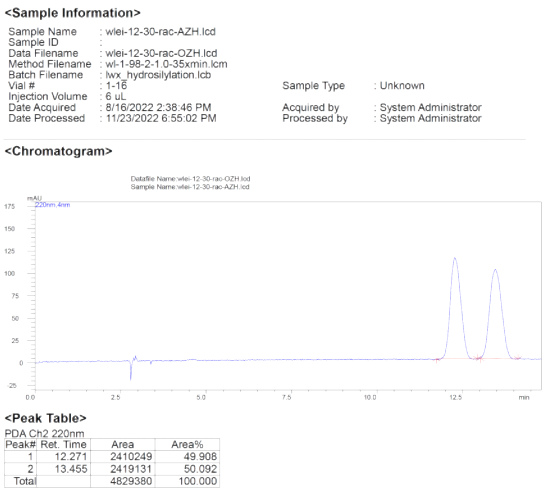

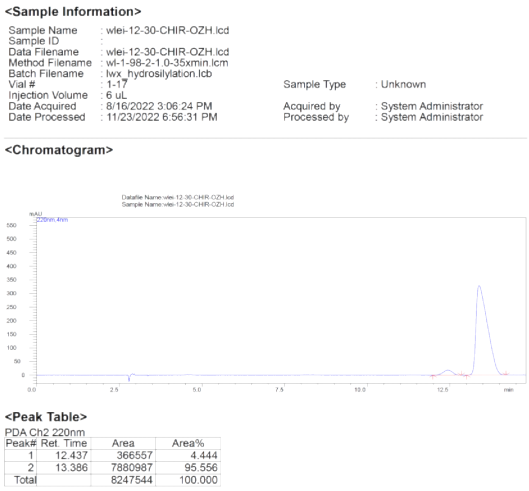

(*R*)-phenyl(1-((trimethylsilyl)ethynyl)cyclopentyl)methanol (6y)

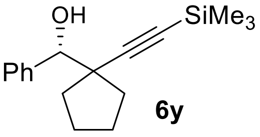

6y

Colorless oil. **IR (neat):** 3245 (w), 2161 (w), 1452 (m), 1249 (m), 840 (s), 759 (m), 700 (m) cm^{-1}; **^{1}H NMR (400 MHz, $CDCl_3$):** δ 7.44 (d, *J* = 7.6 Hz, 2H), 7.37 – 7.13 (m, 3H), 4.43 (d, *J* = 6.0 Hz, 1H), 2.54 (d, *J* = 6.0 Hz, 1H), 2.05 – 1.95 (m, 1H), 1.95 – 1.72 (m, 3H), 1.71 – 1.50 (m, 4H), 0.15 (s, 9H); **^{13}C NMR (100 MHz, $CDCl_3$):** δ 141.4, 127.7, 127.6, 127.4, 110.8, 87.9, 78.9, 50.7, 38.2, 36.8, 24.2, 24.2, 0.1; **HRMS (EI^{+}) [M-H_2O]$^{+}$**Calcd for $C_{17}H_{22}Si$: 254.1485 m/z, Found: 254.1483 m/z; **Specific rotation**: $[\alpha]_D^{20}$ 49.98 (*c* 1.00, $CHCl_3$) for an enantiomerically enriched sample of 90:10 e.r.

Enantiomeric purity of **6y** was determined by HPLC analysis in comparison with authentic racemic material (90:10 e.r. shown; Chiralpak IF column, 99.5:0.5 *n*-hexane / *i*-PrOH, 0.8 mL/min, 220 nm).

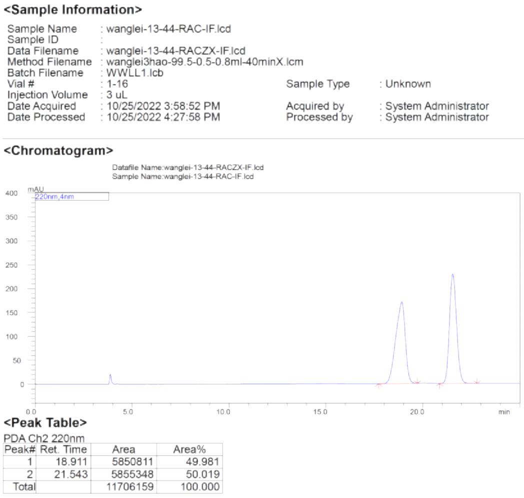

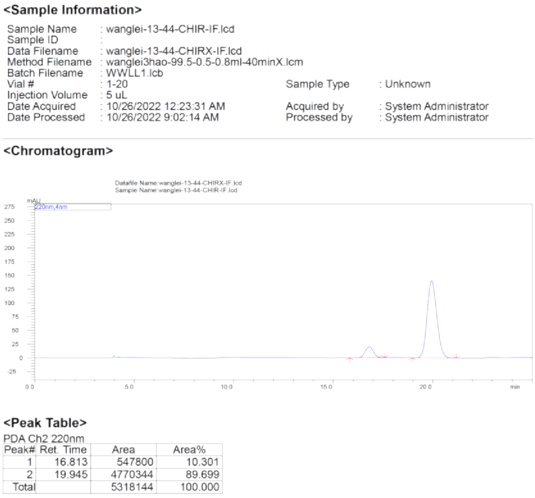

(*R*)-phenyl(1-((trimethylsilyl)ethynyl)cyclohexyl)methanol (6z)

Colorless oil. **IR (neat):** 3034 (w), 2163 (w), 1450 (w), 1249 (m), 867 (m), 840 (s), 759

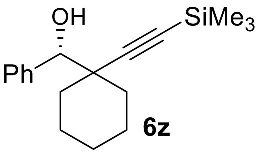

6z

(m), 700 (m) cm^{-1}; **^{1}H NMR (400 MHz, $CDCl_3$):** δ 7.42 – 7.35 (m, 2H), 7.35 – 7.24 (m, 3H), 4.37 (d, *J* = 4.8 Hz, 1H), 2.56 (d, *J* = 5.2 Hz, 1H), 1.98 (d, *J* = 12.4 Hz, 1H), 1.70 – 1.57 (m, 3H), 1.58 – 1.52 (m, 2H), 1.51 – 1.37 (m, 1H), 1.32 – 1.18 (m, 1H), 1.15 – 0.96 (m, 2H), 0.19 (s, 9H); **^{13}C NMR (100 MHz, $CDCl_3$):** δ 140.1, 127.8, 127.6, 127.4, 109.1, 90.4, 80.5, 44.9, 34.8, 33.0, 25.9, 22.8, 22.7, 0.2;

HRMS (ESI$^+$) [M+Na]$^+$ Calcd for $C_{18}H_{26}ONaSi$: 309.1645 m/z, Found: 309.1648 m/z; **Specific rotation**: $[\alpha]_D^{20}$ 47.08 (*c* 2.00, $CHCl_3$) for an enantiomerically enriched sample of 90:10 e.r.

Enantiomeric purity of **6z** was determined by HPLC analysis in comparison with authentic racemic material (90:10 e.r. shown; Chiralcel OZ-H column, 99.5:0.5 *n*-hexane / *i*-PrOH, 0.8 mL/min, 220 nm).

<Sample Information>

Sample Name : wanglei-13-27-RAC-ozh.lcd
Sample ID :
Data Filename : wanglei-13-27-RACzX-ozh.lcd
Method Filename : 1-99.5-0.5-0.8ml-40minX.lcm
Batch Filename : WWLL1.lcb
Vial # : 1-17 Sample Type : Unknown
Injection Volume : 7 uL
Date Acquired : 10/17/2022 1:36:21 AM Acquired by : System Administrator
Date Processed : 10/17/2022 11:21:06 AM Processed by : System Administrator

<Chromatogram>

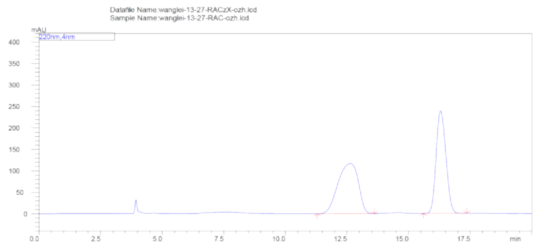

<Peak Table>

PDA Ch2 220nm

Peak#	Ret. Time	Area	Area%
1	12.635	6721953	50.497
2	16.301	6589640	49.503
Total		13311593	100.000

<Sample Information>

Sample Name : wanglei-13-27-chir-ozh.lcd
Sample ID :
Data Filename : wanglei-13-27-chirx-ozh.lcd
Method Filename : 1-99.5-0.5-0.8ml-40minX.lcm
Batch Filename : WWLL1.lcb
Vial # : 1-19 Sample Type : Unknown
Injection Volume : 0.8 uL
Date Acquired : 10/17/2022 10:56:45 AM Acquired by : System Administrator
Date Processed : 10/17/2022 11:21:17 AM Processed by : System Administrator

<Chromatogram>

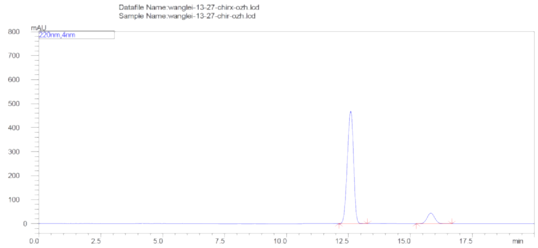

<Peak Table>

PDA Ch2 220nm

Peak#	Ret. Time	Area	Area%
1	12.594	8196097	89.635
2	15.828	947764	10.365
Total		9143861	100.000

(*R*)-2,2-dimethyl-1-phenyl-4-(trimethylsilyl)but-3-yn-1-ol (6aa)

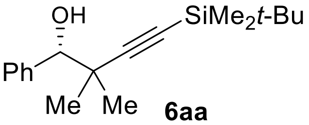

6aa

Colorless oil. **^{1}H NMR (400 MHz, $CDCl_3$):** δ 7.55 – 7.42 (m, 2H), 7.41 – 7.33 (m, 3H), 4.53 (s, 1H), 2.60 (s, 1H), 1.31 (s, 3H), 1.14 (s, 3H), 0.24 (s, 9H); **^{13}C NMR (100 MHz, $CDCl_3$):** δ 139.8, 127.7, 127.5, 111.5, 87.0, 80.1, 38.7, 26.1, 24.5, 0.1; **Specific rotation**: $[\alpha]_D^{20}$ 46.57 (*c* 1.00, $CHCl_3$) for an enantiomerically enriched sample of 88:12 e.r. The absolute configuration of **6** was assigned as *R* by comparing the optical rotation of **6aa** to literature precedent[2].

Enantiomeric purity of **6aa** was determined by SFC analysis in comparison with authentic racemic material (88:12 e.r. shown; Chiralpak IC column, 96:4 CO_2 / *i*-PrOH, 1.0 mL/min, 220 nm).

<Sample Information>

Sample Name : WL-12--51X-RAC-IC.lcd
Sample ID :
Data Filename : WL-12--51X-RAC-IC.lcd
Method Filename : wl-1-96-4-1.0-15min.lcm
Batch Filename : lwx_hydrosilylation.lcb
Vial # : 1-76 Sample Type : Unknown
Injection Volume : 4 uL
Date Acquired : 12/13/2022 4:57:28 PM Acquired by : System Administrator
Date Processed : 12/13/2022 6:07:45 PM Processed by : System Administrator

<Chromatogram>

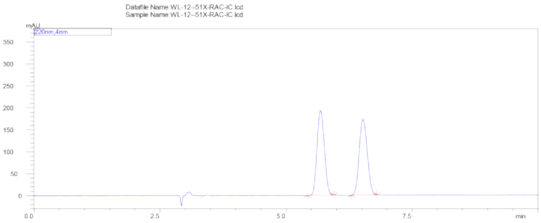

<Peak Table>

PDA Ch2 220nm

Peak#	Ret. Time	Area	Area%
1	5.684	1954126	50.225
2	6.527	1936634	49.775
Total		3890760	100.000

<Sample Information>

Sample Name : WL-12--51X-CHIR-IC.lcd
Sample ID :
Data Filename : WL-12--51X-CHIR-IC.lcd
Method Filename : wl-1-96-4-1.0-15min.lcm
Batch Filename : lwx_hydrosilylation.lcb
Vial # : 1-77 Sample Type : Unknown
Injection Volume : 2 uL
Date Acquired : 12/13/2022 4:41:35 PM Acquired by : System Administrator
Date Processed : 12/13/2022 6:09:55 PM Processed by : System Administrator

<Chromatogram>

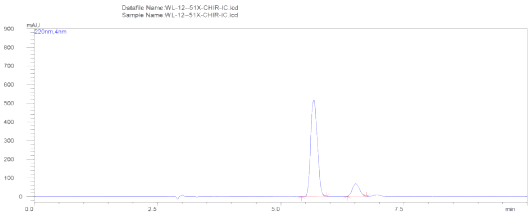

<Peak Table>

PDA Ch2 220nm

Peak#	Ret. Time	Area	Area%
1	5.668	4845815	87.759
2	6.522	675899	12.241
Total		5521713	100.000

(*R*)-4-(*tert*-butyldimethylsilyl)-2,2-dimethyl-1-phenylbut-3-yn-1-ol (6ab)

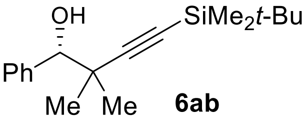

6ab

Colorless oil. **IR (neat):** 3482 (w), 2164 (w), 1467 (m), 1252 (m), 837 (s), 738 (m), 701 (m) cm^{-1}; **^{1}H NMR (400 MHz, $CDCl_3$):** δ 7.42 – 7.37 (m, 2H), 7.35 – 7.27 (m, 3H), 4.47 (d, *J* = 3.6 Hz, 1H), 2.51 (d, *J* = 4.0 Hz, 1H), 1.25 (s, 3H), 1.08 (s, 3H), 0.93 (s, 9H), 0.11 (s, 6H); **^{13}C NMR (100 MHz, $CDCl_3$):** δ 139.8, 127.7, 127.5, 112.1, 85.3, 80.1, 38.8, 26.3, 26.1, 24.4, 16.5, -4.5; **HRMS (ESI$^+$) [M+Na]$^+$** Calcd for $C_{18}H_{28}ONaSi$: 311.1802 m/z, Found: 311.1808 m/z; **Specific rotation**: $[\alpha]_D^{20}$ 51.21 (*c* 1.00, $CHCl_3$) for an enantiomerically enriched sample of 95:5 e.r.,

Enantiomeric purity of **6ab** was determined by SFC analysis in comparison with authentic racemic material (95:5 e.r. shown; Chiralpak IC column, 97:3 CO_2 / *i*-PrOH, 1.0 mL/min, 220 nm).

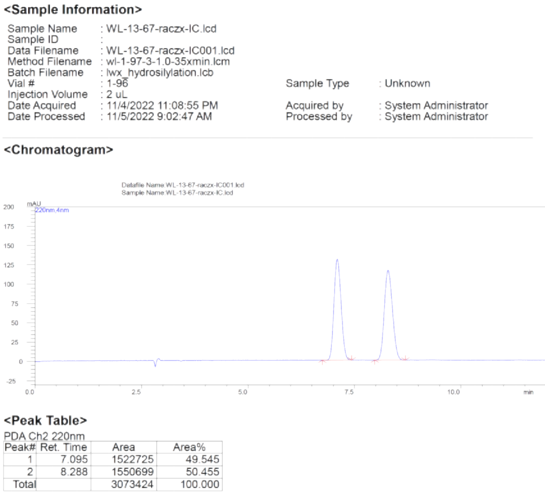

<Sample Information>

Sample Name : WL-13-67-raczx-IC.lcd
Sample ID :
Data Filename : WL-13-67-raczx-IC001.lcd
Method Filename : wl-1-97-3-1.0-35xmin.lcm
Batch Filename : lwx_hydrosilylation.lcb
Vial # : 1-96
Injection Volume : 2 uL
Date Acquired : 11/4/2022 11:08:55 PM
Date Processed : 11/5/2022 9:02:47 AM
Sample Type : Unknown
Acquired by : System Administrator
Processed by : System Administrator

<Chromatogram>

<Peak Table>

PDA Ch2 220nm

Peak#	Ret. Time	Area	Area%
1	7.095	1522725	49.545
2	8.288	1550699	50.455
Total		3073424	100.000

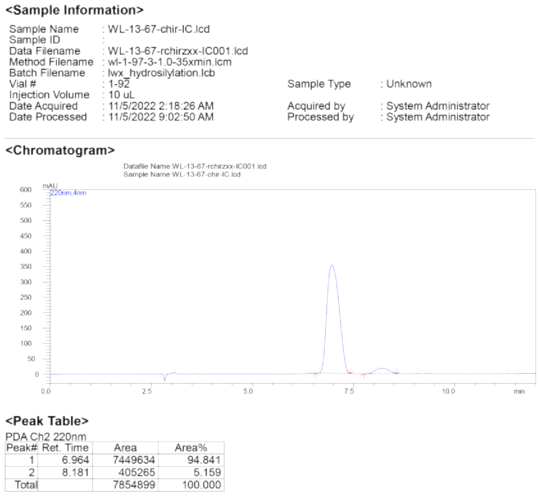

<Sample Information>

Sample Name : WL-13-67-chir-IC.lcd
Sample ID :
Data Filename : WL-13-67-rchirzxx-IC001.lcd
Method Filename : wl-1-97-3-1.0-35xmin.lcm
Batch Filename : lwx_hydrosilylation.lcb
Vial # : 1-92
Injection Volume : 10 uL
Date Acquired : 11/5/2022 2:18:26 AM
Date Processed : 11/5/2022 9:02:50 AM
Sample Type : Unknown
Acquired by : System Administrator
Processed by : System Administrator

<Chromatogram>

<Peak Table>

PDA Ch2 220nm

Peak#	Ret. Time	Area	Area%
1	6.964	7449634	94.841
2	8.181	405265	5.159
Total		7854899	100.000

(*R*)-1-phenyl-4-(trimethylsilyl)but-3-yn-1-ol (6ac)

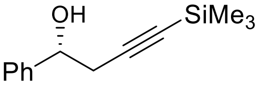

6ac

Colorless oil. **^{1}H NMR (400 MHz, $CDCl_3$):** δ 7.44 – 7.33 (m, 4H), 7.35 – 7.29 (m, 1H), 4.87 (td, *J* = 6.4, 2.8 Hz, 1H), 2.68 (d, *J* = 6.0 Hz, 2H), 2.56 (s, 1H), 0.18 (s, 9H); **^{13}C NMR (100 MHz, $CDCl_3$):** δ 142.5, 128.4, 127.8, 125.7, 102.9, 87.9, 72.3, 31.2, -0.0; **Specific rotation**: $[\alpha]_D^{20}$ 47.84 (*c* 1.00, $CHCl_3$) for an enantiomerically enriched sample of 96:4 e.r. The absolute configuration of **6ac** was assigned as *R* by comparing the optical rotation of **6ac** to literature precedent[3].

Enantiomeric purity of **6ac** was determined by HPLC analysis in comparison with authentic racemic material (96:4 e.r. shown; Chiralpak IF column, 99:1 *n*-hexane / *i*-PrOH, 0.6 mL/min, 220 nm).

<Sample Information>

Sample Name : WL-14-9-2RAC-IF.lcd
Sample ID :
Data Filename : WL-14-9-2RAC-IF.lcd
Method Filename : wanglei6hao-99-1--0.6ml-30minX.lcm
Batch Filename : WWLL1.lcb
Vial # : 1-92 Sample Type : Unknown
Injection Volume : 3 uL
Date Acquired : 12/15/2022 10:08:15 AM Acquired by : System Administrator
Date Processed : 12/15/2022 12:20:25 PM Processed by : System Administrator

<Chromatogram>

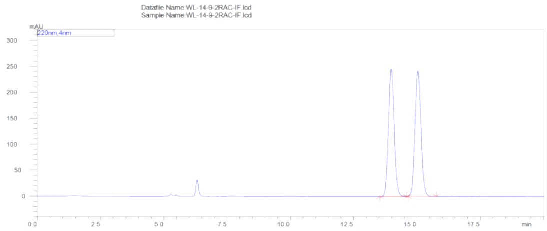

<Peak Table>

PDA Ch2 220nm

Peak#	Ret. Time	Area	Area%
1	13.979	3916904	49.481
2	15.033	3999012	50.519
Total		7915915	100.000

<Sample Information>

Sample Name : WL-14-9-2CHIR-IF.lcd
Sample ID :
Data Filename : WL-14-9-2CHIR-IF.lcd
Method Filename : wanglei6hao-99-1--0.6ml-30minX.lcm
Batch Filename : WWLL1.lcb
Vial # : 1-94 Sample Type : Unknown
Injection Volume : 3 uL
Date Acquired : 12/15/2022 11:30:49 AM Acquired by : System Administrator
Date Processed : 12/15/2022 12:22:02 PM Processed by : System Administrator

<Chromatogram>

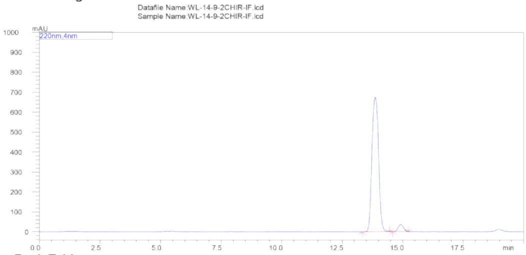

<Peak Table>

PDA Ch2 220nm

Peak#	Ret. Time	Area	Area%
1	13.869	12830214	95.845
2	14.921	556212	4.155
Total		13386426	100.000

(*R*)-1-phenyl-2-(trimethylsilyl)buta-2,3-dien-1-ol (S31)

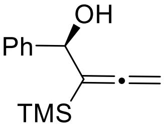

S31

25% yield of pure allenyl addition product was isolated. Colorless oil. **^{1}H NMR (400 MHz, $CDCl_3$):** δ 7.63 – 7.49 (m, 4H), 7.51 – 7.39 (m, 1H), 5.43 (s, 1H), 4.98 – 4.72 (m, 2H), 2.53 (d, *J* = 4.8 Hz, 1H), 0.17 (s, 9H); **^{13}C NMR (100 MHz, $CDCl_3$):** δ 207.0, 143.0, 128.2, 127.8, 126.9, 101.3, 72.9, 72.0, -1.2; **Specific rotation**: $[\alpha]_D^{20}$ -92.99 (*c* 0.50, $CHCl_3$) for an enantiomerically enriched sample of >99.5:0.5 e.r. The absolute configuration of **S31** was assigned as *R* by comparing the optical rotation of **S26** to literature precedent[4].

Enantiomeric purity of **S31** was determined by HPLC analysis in comparison with authentic racemic material (>99.5:0.5 e.r. shown; Chiralpak IF column, 99:1 *n*-hexane / *i*-PrOH, 0.6 mL/min, 220 nm).

<Sample Information>

Sample Name : WL-14-9-1RAC-IF.lcd
Sample ID :
Data Filename : WL-14-9-1RAC-IF001.lcd
Method Filename : wanglei6hao-99-1--0.6ml-30minX.lcm
Batch Filename : WWLL1.lcb
Vial # : 1-91 Sample Type : Unknown
Injection Volume : 3 uL
Date Acquired : 12/15/2022 5:53:15 PM Acquired by : System Administrator
Date Processed : 12/15/2022 6:18:19 PM Processed by : System Administrator

<Chromatogram>

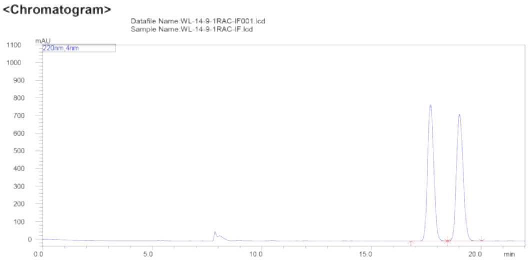

<Peak Table>

PDA Ch2 220nm

Peak#	Ret. Time	Area	Area%
1	17.693	15186943	49.660
2	19.015	15394629	50.340
Total		30581572	100.000

<Sample Information>

Sample Name : WL-14-9-1CHIR-IF.lcd
Sample ID :
Data Filename : WL-14-9-1CHIRXZ-IF.lcd
Method Filename : wanglei6hao-99-1--0.6ml-30minX.lcm
Batch Filename : WWLL1.lcb
Vial # : 1-93 Sample Type : Unknown
Injection Volume : 3 uL
Date Acquired : 12/15/2022 5:03:27 PM Acquired by : System Administrator
Date Processed : 12/15/2022 6:29:20 PM Processed by : System Administrator

<Chromatogram>

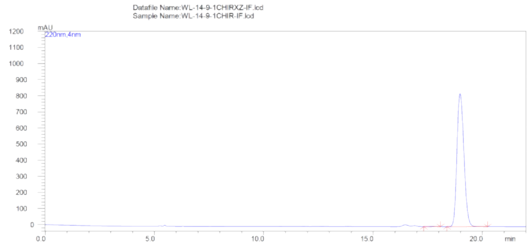

<Peak Table>

PDA Ch2 220nm

Peak#	Ret. Time	Area	Area%
1	17.699	37123	0.202
2	18.986	18330609	99.798
Total		18367731	100.000

(*R*)-1,4-diphenylbut-3-yn-1-ol (6ad)

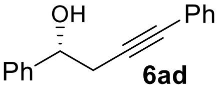

6ad

Colorless oil. **^{1}H NMR (400 MHz, $CDCl_3$):** δ 7.44 (d, *J* = 7.2 Hz, 2H), 7.42 – 7.34 (m, 4H), 7.34 – 7.26 (m, 4H), 4.96 (td, *J* = 6.4, 2.8 Hz, 1H), 2.87 (d, *J* = 6.4 Hz, 2H), 2.45 (d, *J* = 3.2 Hz, 1H); **^{13}C NMR (100 MHz, $CDCl_3$):** δ 142.6, 131.6, 128.4, 128.2, 128.0, 127.9, 125.8, 123.2, 85.9, 83.2, 72.6, 30.6; **Specific rotation**: $[\alpha]_D^{20}$ 23.28 (*c* 1.00, $CHCl_3$) for an enantiomerically enriched sample of 92.5:7.5 e.r.

Enantiomeric purity of **6ad** was determined by HPLC analysis in comparison with authentic racemic material (92.5:7.5 e.r. shown; Chiralpak IF column, 98:2 *n*-hexane / *i*-PrOH, 0.6 mL/min, 254 nm).

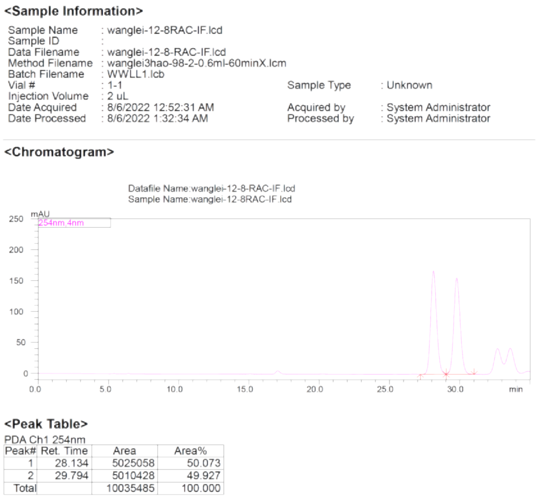
<Sample Information>
Sample Name : wanglei-12-8RAC-IF.lcd
Sample ID :
Data Filename : wanglei-12-8-RAC-IF.lcd
Method Filename : wanglei3hao-98-2-0.6ml-60minX.lcm
Batch Filename : WWLL1.lcb
Vial # : 1-1 Sample Type : Unknown
Injection Volume : 2 uL
Date Acquired : 8/6/2022 12:52:31 AM Acquired by : System Administrator
Date Processed : 8/6/2022 1:32:34 AM Processed by : System Administrator

<Chromatogram>

<Peak Table>
PDA Ch1 254nm

Peak#	Ret. Time	Area	Area%
1	28.134	5025058	50.073
2	29.794	5010428	49.927
Total		10035485	100.000

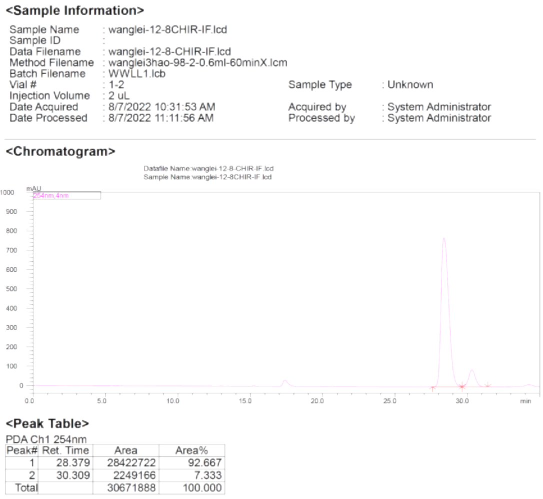
<Sample Information>
Sample Name : wanglei-12-8CHIR-IF.lcd
Sample ID :
Data Filename : wanglei-12-8-CHIR-IF.lcd
Method Filename : wanglei3hao-98-2-0.6ml-60minX.lcm
Batch Filename : WWLL1.lcb
Vial # : 1-2 Sample Type : Unknown
Injection Volume : 2 uL
Date Acquired : 8/7/2022 10:31:53 AM Acquired by : System Administrator
Date Processed : 8/7/2022 11:11:56 AM Processed by : System Administrator

<Chromatogram>

<Peak Table>
PDA Ch1 254nm

Peak#	Ret. Time	Area	Area%
1	28.379	28422722	92.667
2	30.309	2249166	7.333
Total		30671888	100.000

(*R*)-4-(4-methoxyphenyl)-1-phenylbut-3-yn-1-ol (6ae)

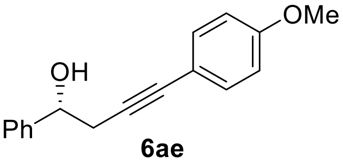

6ae

Colorless oil. **^{1}H NMR (400 MHz, CDCl$_3$):** δ 7.43 (d, *J* = 6.8 Hz, 2H), 7.42 – 7.28 (m, 5H), 6.82 (d, *J* = 6.8 Hz, 2H), 4.93 (t, *J* = 6.4 Hz, 1H), 3.80 (s, 3H), 2.84 (d, *J* = 6.4 Hz, 2H), 2.48 (s, 1H); **^{13}C NMR (100 MHz, CDCl$_3$):** δ 159.3, 142.7, 133.0, 128.4, 127.8, 125.8, 115.3, 113.8, 84.3, 83.0, 72.6, 55.2, 30.7. **Specific rotation**: $[\alpha]_D^{20}$ 14.46 (*c* 1.00, CHCl$_3$) for an enantiomerically enriched sample of 87:13 e.r.

Enantiomeric purity of **6ae** was determined by HPLC analysis in comparison with authentic racemic material (87:13 e.r. shown; Chiralpak IF column, 92:8 *n*-hexane / *i*-PrOH, 1.0 mL/min, 254 nm).

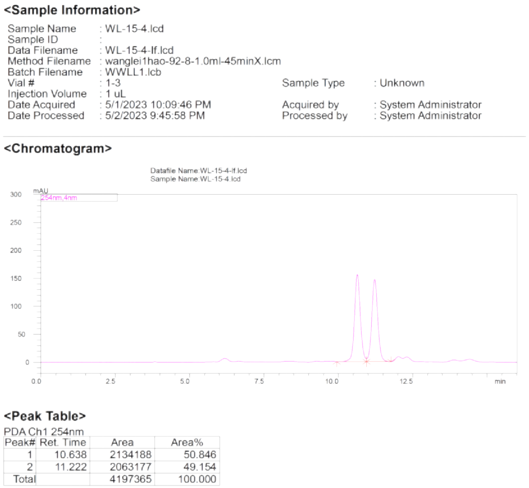
<Sample Information>
Sample Name : WL-15-4.lcd
Sample ID :
Data Filename : WL-15-4-If.lcd
Method Filename : wanglei1hao-92-8-1.0ml-45minX.lcm
Batch Filename : WWLL1.lcb
Vial # : 1-3 Sample Type : Unknown
Injection Volume : 1 uL
Date Acquired : 5/1/2023 10:09:46 PM Acquired by : System Administrator
Date Processed : 5/2/2023 9:45:58 PM Processed by : System Administrator

<Chromatogram>

<Peak Table>
PDA Ch1 254nm

Peak#	Ret. Time	Area	Area%
1	10.638	2134188	50.846
2	11.222	2063177	49.154
Total		4197365	100.000

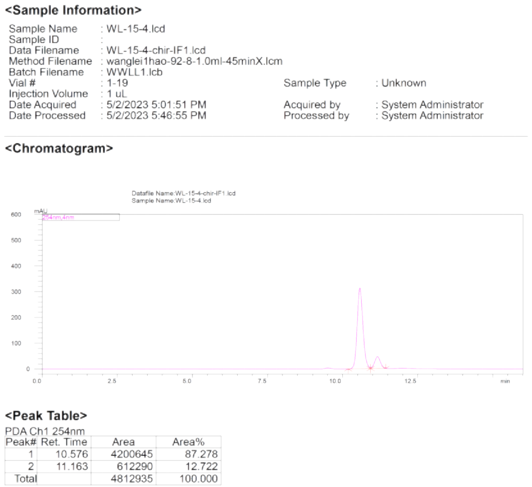
<Sample Information>
Sample Name : WL-15-4.lcd
Sample ID :
Data Filename : WL-15-4-chir-IF1.lcd
Method Filename : wanglei1hao-92-8-1.0ml-45minX.lcm
Batch Filename : WWLL1.lcb
Vial # : 1-19 Sample Type : Unknown
Injection Volume : 1 uL
Date Acquired : 5/2/2023 5:01:51 PM Acquired by : System Administrator
Date Processed : 5/2/2023 5:46:55 PM Processed by : System Administrator

<Chromatogram>

<Peak Table>
PDA Ch1 254nm

Peak#	Ret. Time	Area	Area%
1	10.576	4200645	87.278
2	11.163	612290	12.722
Total		4812935	100.000

(*R*)-4-(3-methoxyphenyl)-1-phenylbut-3-yn-1-ol (6af)

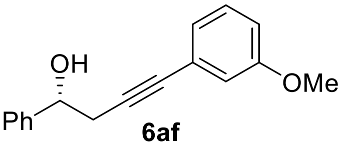

6af

Colorless oil. **^{1}H NMR (400 MHz, CDCl$_3$):** δ 7.49 – 7.42 (m, 2H), 7.41 – 7.36 (m, 2H), 7.35 – 7.28 (m, 1H), 7.20 (t, *J* = 8.0 Hz, 1H), 6.99 (d, *J* = 7.6 Hz, 1H), 6.95 – 6.90 (m, 1H), 6.90 – 6.80 (m, 1H), 4.96 (t, *J* = 6.4 Hz, 1H), 3.79 (s, 3H), 2.86 (d, *J* = 6.4 Hz, 2H), 2.44 (s, 1H); **^{13}C NMR (100**

MHz, $CDCl_3$): δ 159.2, 142.6, 129.3, 128.4, 127.9, 125.8, 124.2, 116.5, 114.6, 85.8, 83.1, 72.6, 55.2, 30.6. **Specific rotation**: $[\alpha]_D^{20}$ 8.35 (*c* 1.00, $CHCl_3$) for an enantiomerically enriched sample of 94:6 e.r.

Enantiomeric purity of **6af** was determined by HPLC analysis in comparison with authentic racemic material (94:6 e.r. shown; Chiralpak OJH column, 92:8 *n*-hexane / *i*-PrOH, 0.6 mL/min, 254 nm).

<Sample Information>

Sample Name : WL-15-3chir.lcd
Sample ID :
Data Filename : WL-15-3-rac2-ojh003.lcd
Method Filename : wanglei6hao-92-8-0.6ml-75minX.lcm
Batch Filename : WWLL1.lcb
Vial # : 1-1 Sample Type : Unknown
Injection Volume : 15 uL
Date Acquired : 5/9/2023 3:39:08 PM Acquired by : System Administrator
Date Processed : 5/9/2023 4:54:12 PM Processed by : System Administrator

<Chromatogram>

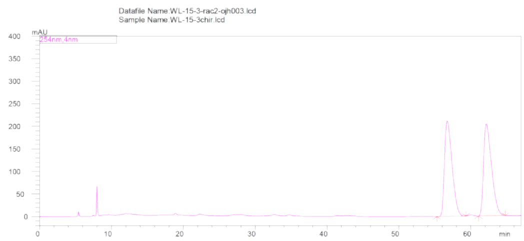

<Peak Table>

PDA Ch1 254nm

Peak#	Ret. Time	Area	Area%
1	56.717	15464417	49.563
2	62.154	15737282	50.437
Total		31201699	100.000

<Sample Information>

Sample Name : WL-15-3chir.lcd
Sample ID :
Data Filename : WL-15-3-chir2-ojh002.lcd
Method Filename : wanglei6hao-92-8-0.6ml-75minX.lcm
Batch Filename : WWLL1.lcb
Vial # : 1-2 Sample Type : Unknown
Injection Volume : 4 uL
Date Acquired : 5/4/2023 3:47:29 PM Acquired by : System Administrator
Date Processed : 5/4/2023 5:02:32 PM Processed by : System Administrator

<Chromatogram>

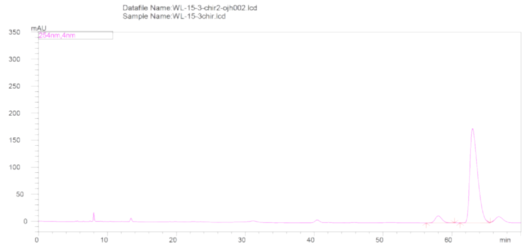

<Peak Table>

PDA Ch1 254nm

Peak#	Ret. Time	Area	Area%
1	58.023	898909	6.210
2	62.989	13576283	93.790
Total		14475192	100.000

(*R*)-4-(3-fluorophenyl)-1-phenylbut-3-yn-1-ol (6ag)

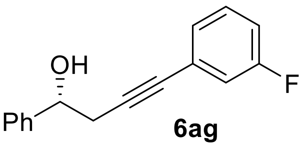

6ag

Colorless oil. **^{1}H NMR (400 MHz, $CDCl_3$):** δ 7.47 – 7.41 (m, 2H), 7.41 – 7.36 (m, 2H), 7.36 – 7.29 (m, 1H), 7.27 – 7.22 (m, 1H), 7.20 – 7.12 (m, 1H), 7.12 – 7.04 (m, 1H), 7.03 – 6.93 (m, 1H), 4.96 (td, *J* = 6.4, 2.8 Hz, 1H), 2.86 (d, *J* = 6.4 Hz, 2H), 2.39 (d, *J* = 3.2 Hz, 1H); **^{13}C NMR (100 MHz, $CDCl_3$):** δ 162.3 (d, *J* = 246.2 Hz), 129.8 (d, *J* = 8.7 Hz), 128.5, 128.0, 127.5 (d, *J* = 3.0 Hz), 125.8, 118.4 (d, *J* = 22.7 Hz), 115.3 (d, *J* = 21.2 Hz), 87.1, 81.9, 72.6, 30.4; **^{19}F NMR (376 MHz, $CDCl_3$):** δ -113.2. **Specific rotation**: $[\alpha]_D^{20}$ 10.11 (*c* 1.00, $CHCl_3$) for an enantiomerically enriched sample of 89:11 e.r.

Enantiomeric purity of **6ag** was determined by HPLC analysis in comparison with authentic racemic material (89:11 e.r. shown; Chiralpak OJH column, 95:5 *n*-hexane / *i*-PrOH, 1.0 mL/min, 254 nm).

<Sample Information>

Sample Name : WL-15-12.lcd
Sample ID :
Data Filename : WL-15-12-ojh.lcd
Method Filename : wanglei6hao-95-5-1.0ml-45minX.lcm
Batch Filename : WWLL1.lcb
Vial # : 1-6 Sample Type : Unknown
Injection Volume : 1 uL
Date Acquired : 5/10/2023 3:10:24 AM Acquired by : System Administrator
Date Processed : 5/10/2023 9:38:12 PM Processed by : System Administrator

<Chromatogram>

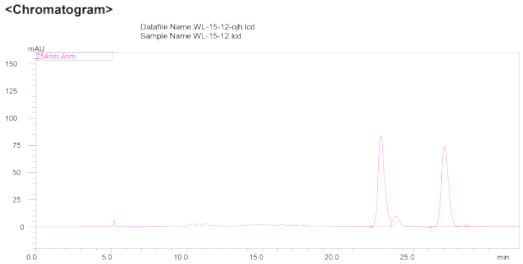

<Peak Table>

PDA Ch1 254nm

Peak#	Ret. Time	Area	Area%
1	22.844	2474000	50.407
2	27.065	2434008	49.593
Total		4908008	100.000

<Sample Information>

Sample Name : WL-15-12.lcd
Sample ID :
Data Filename : WL-15-12chir0509-ojh.lcd
Method Filename : wanglei6hao-95-5-1.0ml-45minX.lcm
Batch Filename : WWLL1.lcb
Vial # : 1-1 Sample Type : Unknown
Injection Volume : 1 uL
Date Acquired : 5/10/2023 9:14:32 PM Acquired by : System Administrator
Date Processed : 5/10/2023 10:14:36 PM Processed by : System Administrator

<Chromatogram>

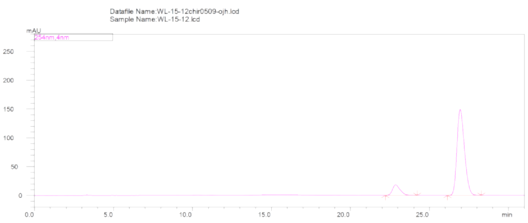

<Peak Table>

PDA Ch1 254nm

Peak#	Ret. Time	Area	Area%
1	22.858	615770	11.093
2	26.939	4935373	88.907
Total		5551143	100.000

(*R*)-1-phenyl-4-(*m*-tolyl)but-3-yn-1-ol (6ah)

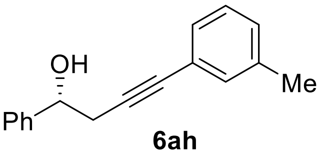

6ah

Colorless oil. **^{1}H NMR (400 MHz, $CDCl_3$):** 7.50 – 7.41 (m, 2H), 7.41 – 7.35 (m, 2H), 7.34 – 7.28 (m, 1H), 7.27 – 7.15 (m, 3H), 7.16 – 7.04 (m, 1H), 4.95 (td, *J* = 6.4, 2.8 Hz, 1H), 2.85 (d, *J* = 6.8 Hz, 2H), 2.47 (d, *J* = 2.4 Hz, 1H), 2.31 (s, 3H); **^{13}C NMR (100 MHz, $CDCl_3$):** δ 159.2, 142.6, 129.3, 128.4, 127.9, 125.8, 124.2, 116.5, 114.6, 85.8, 83.1, 72.6, 55.2, 30.6. **Specific rotation**: $[\alpha]_D^{20}$ 17.92 (*c* 1.00, $CHCl_3$) for an enantiomerically enriched sample of 92:8 e.r.

Enantiomeric purity of **6ah** was determined by HPLC analysis in comparison with authentic racemic material (92:8 e.r. shown; Chiralpak IBN5 column, 92:8 *n*-hexane / *i*-PrOH, 1.0 mL/min, 254 nm).

<Sample Information>

Sample Name : WL-15-13.lcd
Sample ID :
Data Filename : WL-15-13-ibn5.lcd
Method Filename : wanglei4hao-92-8-1.0ml-45minX.lcm
Batch Filename : WWLL1.lcb
Vial # : 1-7 Sample Type : Unknown
Injection Volume : 1 uL
Date Acquired : 5/9/2023 9:54:00 PM Acquired by : System Administrator
Date Processed : 5/10/2023 9:04:16 PM Processed by : System Administrator

<Chromatogram>

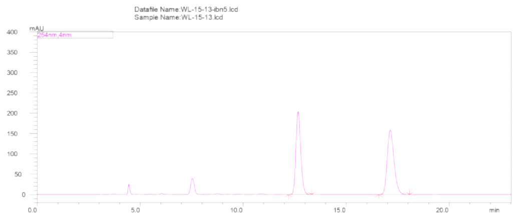

<Peak Table>

PDA Ch1 254nm

Peak#	Ret. Time	Area	Area%
1	12.663	3331865	49.221
2	17.137	3437278	50.779
Total		6769143	100.000

<Sample Information>

Sample Name : WL-15-13.lcd
Sample ID :
Data Filename : WL-15-13-chirx0509-ibn5.lcd
Method Filename : wanglei4hao-92-8-1.0ml-45minX.lcm
Batch Filename : WWLL1.lcb
Vial # : 1-2 Sample Type : Unknown
Injection Volume : 4 uL
Date Acquired : 5/10/2023 8:03:31 PM Acquired by : System Administrator
Date Processed : 5/10/2023 8:48:35 PM Processed by : System Administrator

<Chromatogram>

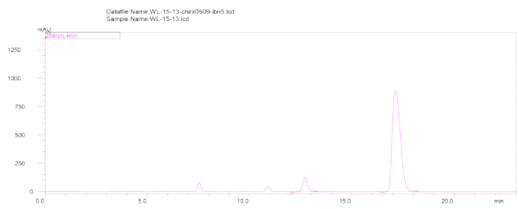

<Peak Table>

PDA Ch1 254nm

Peak#	Ret. Time	Area	Area%
1	12.699	2109938	7.934
2	17.130	24484695	92.066
Total		26594633	100.000

(*R*)-4-(benzo[*d*][1,3]dioxol-5-yl)-1-phenylbut-3-yn-1-ol (6ai)

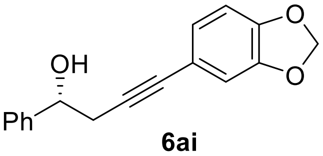

6ai

Colorless oil. **IR (neat):** 3490 (w), 2165 (w), 1469 (m), 1250 (m), 840 (s), 735 (m), 698 (m) cm^{-1}; **^{1}H NMR (400 MHz, $CDCl_3$):** δ 7.47 – 7.41 (m, 2H), 7.41 – 7.34 (m, 2H), 7.34 – 7.28 (m, 1H), 6.91 (dd, *J* = 8.0, 1.6 Hz, 1H), 6.83 (d, *J* = 1.6 Hz, 1H), 6.72 (d, *J* = 8.0 Hz, 1H), 5.95 (s, 2H), 4.93 (td, *J* = 6.4, 3.2 Hz, 1H), 2.83 (d, *J* = 6.0 Hz, 2H), 2.47 (d, *J* = 2.8 Hz, 1H); **^{13}C NMR (100 MHz, $CDCl_3$):** δ 147.6, 147.3, 142.7, 128.4, 127.9, 126.1, 125.8, 116.4, 111.6, 108.3, 101.2, 84.2, 83.0, 72.6, 30.6. **HRMS (ESI$^+$) $[M+Na]^+$** Calcd for $C_{17}H_{14}O_3NaSi$: 289.0835 m/z, Found: 289.0838 m/z; **Specific rotation**: $[\alpha]_D^{20}$ 15.97 (*c* 1.00, $CHCl_3$) for an enantiomerically enriched sample of 92:8 e.r.

Enantiomeric purity of **6ai** was determined by HPLC analysis in comparison with authentic racemic material (92:8 e.r. shown; Chiralpak IBN5 column, 92:8 *n*-hexane / *i*-PrOH, 1.0 mL/min, 254 nm).

<Sample Information>

Sample Name : WL-15-10.lcd
Sample ID :
Data Filename : WL-15-10rac0507-ibn5.lcd
Method Filename : wanglei4hao-92-8-1.0ml-45minX.lcm
Batch Filename : WWLL1.lcb
Vial # : 1-18 Sample Type : Unknown
Injection Volume : 2 uL
Date Acquired : 5/8/2023 1:49:30 AM Acquired by : System Administrator
Date Processed : 5/8/2023 10:06:53 AM Processed by : System Administrator

<Chromatogram>

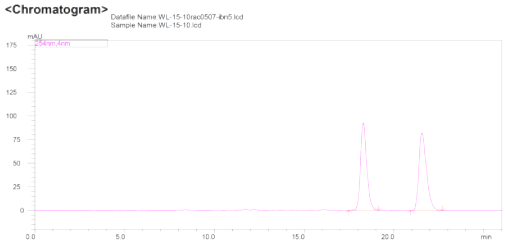

<Peak Table>

PDA Ch1 254nm

Peak#	Ret. Time	Area	Area%
1	18.295	2249323	49.463
2	21.566	2298150	50.537
Total		4547472	100.000

<Sample Information>

Sample Name : WL-15-10.lcd
Sample ID :
Data Filename : WL-15-10-chir0507-ibn5.lcd
Method Filename : wanglei4hao-92-8-1.0ml-45minX.lcm
Batch Filename : WWLL1.lcb
Vial # : 1-19 Sample Type : Unknown
Injection Volume : 2 uL
Date Acquired : 5/8/2023 9:33:05 AM Acquired by : System Administrator
Date Processed : 5/8/2023 10:04:16 AM Processed by : System Administrator

<Chromatogram>

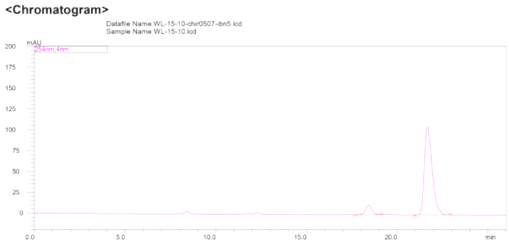

<Peak Table>

PDA Ch1 254nm

Peak#	Ret. Time	Area	Area%
1	18.413	274659	8.008
2	21.666	3154976	91.992
Total		3429635	100.000

(*R*)-4-(naphthalen-2-yl)-1-phenylbut-3-yn-1-ol (6aj)

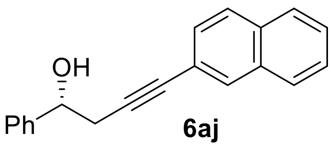

6aj

Colorless oil. **^{1}H NMR (400 MHz, $CDCl_3$):** δ 7.91 (s, 1H), 7.85 – 7.72 (m, 3H), 7.51 – 7.43 (m, 4H), 7.43 – 7.37 (m, 3H), 7.37 – 7.29 (m, 1H), 5.00 (t, *J* = 5.6 Hz, 1H), 2.92 (d, *J* = 6.4 Hz, 2H), 2.51 (s, 1H).; **^{13}C NMR (100 MHz, $CDCl_3$):** δ 142.7, 132.9, 132.6, 131.4, 128.5, 128.5, 127.9, 127.9, 127.7, 127.6, 126.5, 126.4, 125.8, 120.5, 86.3, 83.5, 72.6, 30.7. **Specific rotation**: $[\alpha]_D^{20}$ 10.38 (*c* 0.40, $CHCl_3$) for an enantiomerically enriched sample of 94:6 e.r.

Enantiomeric purity of **6aj** was determined by HPLC analysis in comparison with authentic racemic material (94:6 e.r. shown; Chiralpak IBN5 column, 92:8 *n*-hexane / *i*-PrOH, 1.0 mL/min, 254 nm).

<Sample Information>

Sample Name : WL-15-1.lcd
Sample ID :
Data Filename : WL-15-1-rac-ibn5.lcd
Method Filename : wanglei4hao-92-8-1.0ml-45minX.lcm
Batch Filename : WWLL1.lcb
Vial # : 1-1 Sample Type : Unknown
Injection Volume : 3 uL
Date Acquired : 5/2/2023 10:48:36 PM Acquired by : System Administrator
Date Processed : 5/2/2023 11:33:40 PM Processed by : System Administrator

<Chromatogram>

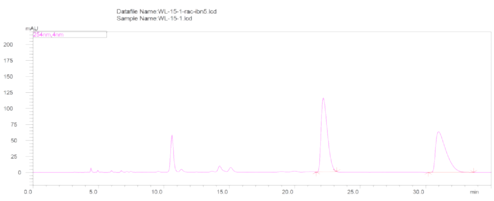

<Peak Table>

PDA Ch1 254nm

Peak#	Ret. Time	Area	Area%
1	22.180	3638491	50.684
2	30.985	3540310	49.316
Total		7178802	100.000

<Sample Information>

Sample Name : WL-15-1.lcd
Sample ID :
Data Filename : WL-15-1-chir0508-ibn5.lcd
Method Filename : wanglei4hao-92-8-1.0ml-45minX.lcm
Batch Filename : WWLL1.lcb
Vial # : 1-16 Sample Type : Unknown
Injection Volume : 2 uL
Date Acquired : 5/9/2023 2:23:37 AM Acquired by : System Administrator
Date Processed : 5/9/2023 3:08:40 AM Processed by : System Administrator

<Chromatogram>

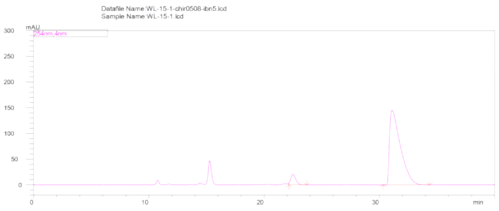

<Peak Table>

PDA Ch1 254nm

Peak#	Ret. Time	Area	Area%
1	22.497	626071	6.163
2	31.112	9532803	93.837
Total		10158875	100.000

■ Experimental Procedure of Gram-Scale Synthesis of 3a

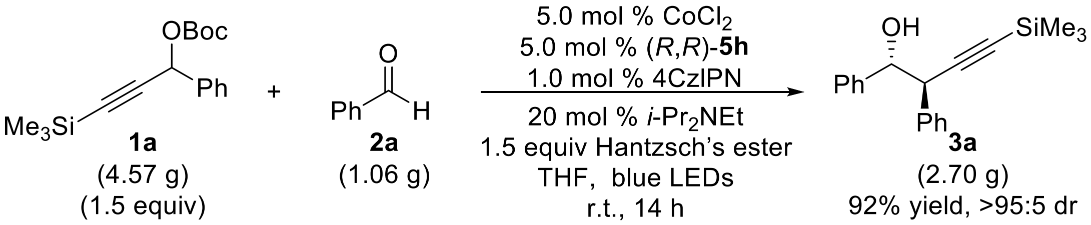

In a N_2-filled glove-box, an oven-dried 100-mL round-bottomed flask equipped with a magnetic stir bar was charged with $CoCl_2$ (64.9 mg, 0.5 mmol, 5.0 mol %), (*R*, *R*)-**5h** (153.1 mg, 0.5 mmol, 5.0 mol %). THF (70.0 mL) was added, then the mixture was allowed to stir at room temperature for 20 min. **2a** (1.061 g, 10 mmol, 1.0 equiv), **1a** (4.566 g, 15 mmol, 1.5 equiv), *i*-Pr_2NEt (258.4 mg, 2.0 mmol, 0.2 equiv), 4CzIPN (78.9 mg, 0.1 mmol, 1.0 mol %) and Hantzsch's ester (3.798 g, 15 mmol, 1.5 equiv) were added to the solution. Then the flask was sealed with a rubber cap and taken out of the glove box. It was irradiated by 2× 25 W blue LEDs (450-460 nm) and allowed to stir at room temperature (about 22 °C) for 14 h with cooling fans.

The blue LEDs (**Supplementary Figure 4**) were purchased online from XuZhou Aijia Electronic Technology Co., Ltd.

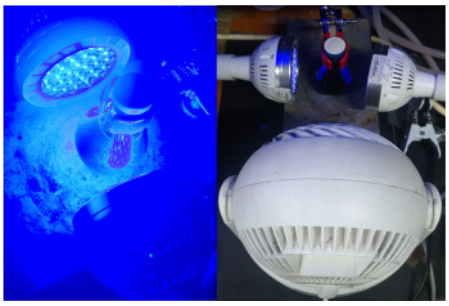

Supplementary Figure 4. Photochemical setup of gram-scale synthesis

Workup: The mixture is filtered through a short plug of 100-200 mesh silica gel eluting with diethyl ether (3×60 mL). The filtrate is concentrated under reduced pressure and the residue was purified by silica-gel column chromatography (eluent: Petroleum ether/ diethyl ether = 12:1) to afford the **3a** as white solid (2.708 g, 9.20 mmol, 92%).

■ Experimental Procedure and Characterization for Functionalization

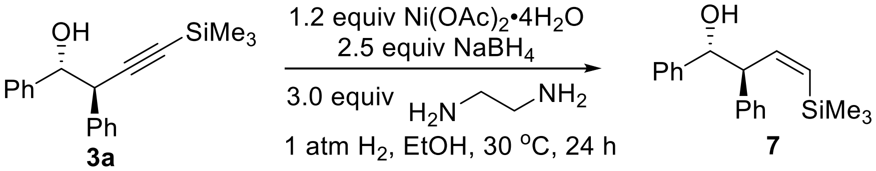

To a solution of **3a** (29.4 mg, 0.1 mmol, 1.0 equiv) in EtOH (1.5 mL) was added nickel(II) acetate tetrahydrate (29.8 mg, 0.12 mmol, 1.2 equiv) and ethylenediamine (18.0 mg, 0.3 mmol, 3.0 equiv) under N_2 atmosphere. The resulting mixture was cooled to 0 °C and $NaBH_4$ (9.5 mg, 0.25 mmol, 2.5 equiv) was added in portions. Then the reaction flask was purged three times with H_2, and the mixture was stirred under H_2 atmosphere at 30 °C for 24 h. Upon completion, the mixture is filtered through a short plug of 100-200 mesh silica gel eluting with diethyl ether (3×20 mL). The filtrate is concentrated under reduced pressure and the residue was purified by silica-gel column chromatography (eluent: Petroleum ether/ diethyl ether = 12:1) to afford the **7** as colorless oil (23.1 mg, 74%).

(1*R*,2*S*, *Z*)-1,2-diphenyl-4-(trimethylsilyl)but-3-en-1-ol (7)

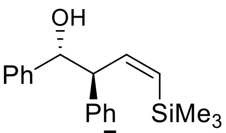

7

Colorless oil. **IR (neat):** 3473 (w), 1600 (w), 1247 (m), 836 (s), 761 (m), 698 (m) cm^{-1}; **^{1}H NMR (400 MHz, $CDCl_3$):** δ 7.36 – 7.22 (m, 5H), 7.22 – 7.15 (m, 3H), 7.10 (d, *J* = 8.0 Hz, 2H), 6.85 (dd, *J* = 14.0, 10.8 Hz, 1H), 5.92 (d, *J* = 14.0 Hz, 1H), 4.88 (d, *J* = 8.0 Hz, 1H), 3.78 (dd, *J* = 10.8, 8.0 Hz, 1H), 2.38 (s, 1H), 0.21 (s, 9H); **^{13}C NMR (100 MHz, $CDCl_3$):** δ 146.7, 141.6, 140.8, 134.0, 128.4, 128.1, 127.9, 127.4, 126.7, 126.5, 77.8, 58.2, 0.3; **HRMS (DART$^+$) [M+NH_4]$^+$**Calcd for $C_{19}H_{28}ONSi$: 314.1935 m/z, Found: 314.1940 m/z; **Specific rotation**: $[\alpha]_D^{20}$ 51.18 (*c* 1.00, $CHCl_3$) for an enantiomerically enriched sample of 97:3 e.r.

Enantiomeric purity of **7** was determined by HPLC analysis in comparison with authentic racemic material (97:3 e.r. shown; Chiralpak IF column, 99:1 *n*-hexane / *i*-PrOH, 0.6 mL/min, 220 nm).

<Sample Information>

Sample Name : WL-13-98RAC-IF002.lcd
Sample ID :
Data Filename : WL-13-98RAC-IF002.lcd
Method Filename : wanglei1hao-99-1-0.6ml-55minX.lcm
Batch Filename : WWLL1.lcb
Vial # : 1-92 Sample Type : Unknown
Injection Volume : 1 uL
Date Acquired : 11/26/2022 2:35:06 PM Acquired by : System Administrator
Date Processed : 11/26/2022 3:05:10 PM Processed by : System Administrator

<Chromatogram>

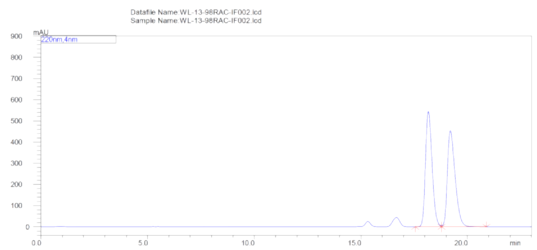

<Peak Table>

PDA Ch2 220nm

Peak#	Ret. Time	Area	Area%
1	18.166	11778190	50.640
2	19.197	11480535	49.360
Total		23258725	100.000

<Sample Information>

Sample Name : WL-13-98CHIR-IF002.lcd
Sample ID :
Data Filename : WL-13-98CHIR-IF002.lcd
Method Filename : wanglei1hao-99-1-0.6ml-55minX.lcm
Batch Filename : WWLL1.lcb
Vial # : 1-91 Sample Type : Unknown
Injection Volume : 1 uL
Date Acquired : 11/26/2022 2:04:32 PM Acquired by : System Administrator
Date Processed : 11/26/2022 2:34:35 PM Processed by : System Administrator

<Chromatogram>

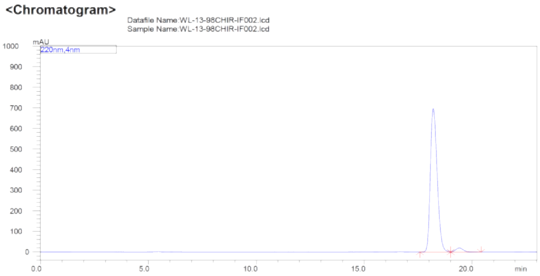

<Peak Table>

PDA Ch2 220nm

Peak#	Ret. Time	Area	Area%
1	18.202	15395070	96.854
2	19.407	500076	3.146
Total		15895146	100.000

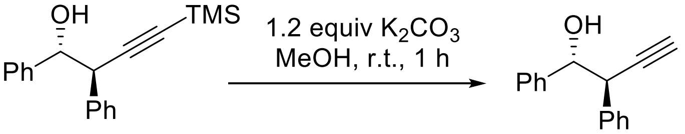

3a **8**

To a solution of **3a** (117.8 mg, 0.4 mmol, 1.0 equiv) in MeOH (1.5 mL) was added K_2CO_3 (66.3 mg, 0.48 mmol, 1.2 equiv) in portions under N_2 atmosphere. The reaction mixture was vigorously stirred at room temperature (about 22 °C) for 1 h. The reaction mixture was quenched with H_2O (4.0 mL) and the resulting aqueous phase was extracted with EA (10 mL×6). The combined organic phase was dried over Na_2SO_4. After removal of the solvent, the residue was purified by silica gel column chromatography (hexanes/EA = 10:1) to afford the **8** (85.3 mg, 96% yield) as white solid.

(1*R*,2*S*)-1,2-diphenylbut-3-yn-1-ol (8)

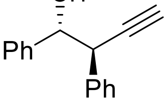

8

White solid, m.p. 62–64 °C; **IR (neat):** 3462 (w), 1494 (w), 1191 (m), 1065 (m), 768 (s), 713 (m), 636 (m) cm^{-1}; **^{1}H NMR (400 MHz, $CDCl_3$):** δ 7.27 – 7.19 (m, 6H), 7.18 – 7.13 (m, 4H), 4.77 (d, *J* = 6.4 Hz, 1H), 3.92 (dd, *J* = 6.4, 2.4 Hz, 1H), 2.70 (s, 1H), 2.37 (d, *J* = 2.4 Hz, 1H); **^{13}C NMR (100 MHz, $CDCl_3$):** δ 140.5, 137.1, 128.4, 128.2, 127.9, 127.8, 127.3, 126.5, 82.6, 77.9, 73.6, 47.5; **HRMS (EI$^+$) [M-H_2O]$^+$**Calcd for $C_{16}H_{12}$:

204.0934 m/z, Found: 204.0925 m/z; **Specific rotation**: $[\alpha]_D^{20}$ -33.24 (*c* 2.00, $CHCl_3$) for an enantiomerically enriched sample of 97:3 e.r.,

Enantiomeric purity of **8** was determined by SFC analysis in comparison with authentic racemic material (97:3 e.r. shown; Chiralpak IA column, 85:15 CO_2 / *i*-PrOH, 1.0 mL/min, 220 nm).

<Sample Information>

Sample Name : WL-13-72-ra-IA.lcd
Sample ID :
Data Filename : WL-13-72-RACX-IA.lcd
Method Filename : wl-4-85-15-1.0-35xmin.lcm
Batch Filename : lwx_hydrosilylation.lcb
Vial # : 1-16 Sample Type : Unknown
Injection Volume : 4 uL
Date Acquired : 11/9/2022 8:58:18 AM Acquired by : System Administrator
Date Processed : 11/24/2022 4:47:52 PM Processed by : System Administrator

<Chromatogram>

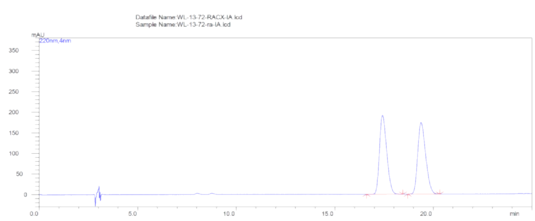

<Peak Table>

PDA Ch2 220nm

Peak#	Ret. Time	Area	Area%
1	17.422	4887505	49.927
2	19.379	4901749	50.073
Total		9789255	100.000

<Sample Information>

Sample Name : WL-13-72-CHIRX-IA.lcd
Sample ID :
Data Filename : WL-13-72-CHIRZX-IA.lcd
Method Filename : wl-4-85-15-1.0-35xmin.lcm
Batch Filename : lwx_hydrosilylation.lcb
Vial # : 1-17 Sample Type : Unknown
Injection Volume : 2 uL
Date Acquired : 11/9/2022 10:21:54 AM Acquired by : System Administrator
Date Processed : 11/24/2022 4:49:16 PM Processed by : System Administrator

<Chromatogram>

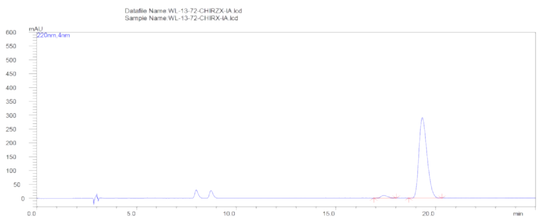

<Peak Table>

PDA Ch2 220nm

Peak#	Ret. Time	Area	Area%
1	17.398	230216	2.726
2	19.331	8216008	97.274
Total		8446224	100.000

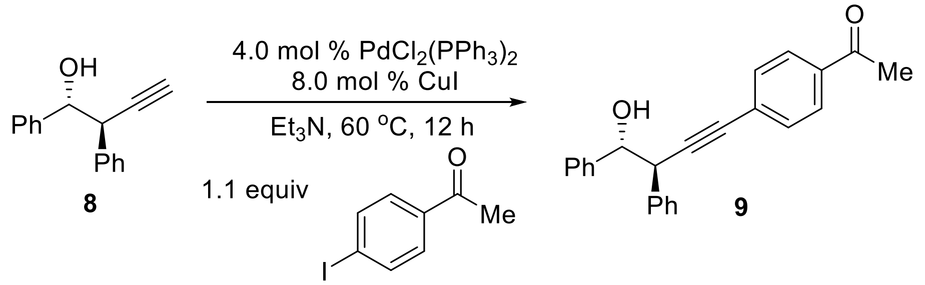

In a N_2-filled glove-box, an oven-dried 8 mL vial equipped with a magnetic stir bar was charged with **8** (44.4 mg, 0.2 mmol, 1.0 equiv), 1-(4-iodophenyl)ethan-1-one (54.1 mg, 0.22 mmol, 1.1 equiv), $PdCl_2(PPh_3)_2$ (5.6 mg, 0.008 mmol, 4.0 mol %), CuI (3.0 mg, 0.016 mmol, 8.0 mol %) and Et_3N (1.0 mL). The vial was sealed with a cap with a septum, and the mixture was allowed to stir at 60 ºC for 12 h. Upon completion, the mixture is filtered through a short plug of 100-200 mesh silica gel eluting with EA (3×20 mL). The filtrate is concentrated under reduced pressure and the residue was purified by silica-gel column chromatography (eluent: Petroleum ether/EA = 6:1) to afford the **9** as white solid (62.9 mg, 92%).

1-(4-((3*R*,4*R*)-4-hydroxy-3,4-diphenylbut-1-yn-1-yl)phenyl)ethan-1-one (9)

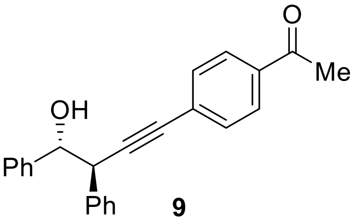

White solid, m.p. 112–114 ºC; **IR (neat):** 3468 (w), 1670 (s), 1600 (m), 1267 (m), 834 (s), 758 (m), 699 (m) cm^{-1}; **^{1}H NMR (400 MHz, $CDCl_3$):** δ 7.89 (d, *J* = 8.0 Hz, 2H), 7.51 (d, *J* = 8.0 Hz, 2H), 7.33 – 7.14 (m, 10H), 4.93 (d, *J* = 6.0 Hz, 1H), 4.20 (d, *J* = 6.0 Hz, 1H), 2.63 (s, 1H), 2.59 (s, 3H); **^{13}C NMR (100 MHz, $CDCl_3$):** δ 197.3, 140.8, 137.4, 136.1, 131.8, 128.5, 128.4, 128.1, 128.0, 127.9, 127.9, 127.4, 126.5, 91.7, 84.9, 78.0, 48.4, 26.5; **HRMS (DART$^+$) $[M+H]^+$** Calcd for $C_{24}H_{21}O_2$: 341.1536 m/z, Found: 341.1540 m/z; **Specific rotation**: $[\alpha]_D^{20}$ 4.40 (*c* 1.00, $CHCl_3$) for an enantiomerically enriched sample of 98:2 e.r.

Enantiomeric purity of **9** was determined by HPLC analysis in comparison with authentic racemic material (98:2 e.r. shown; Chiralpak IA column, 90:10 *n*-hexane / *i*-PrOH, 1.5 mL/min, 220 nm).

<Sample Information>

Sample Name : WL-13-76-RAC-IA.lcd
Sample ID :
Data Filename : WL-13-76-RAC2-IA.lcd
Method Filename : wanglei2hao-90-10-1.5ml-45minX.lcm
Batch Filename : WWLL1.lcb
Vial # : 1-91 Sample Type : Unknown
Injection Volume : 20 uL
Date Acquired : 11/15/2022 10:19:51 AM Acquired by : System Administrator
Date Processed : 11/15/2022 11:46:10 AM Processed by : System Administrator

<Chromatogram>

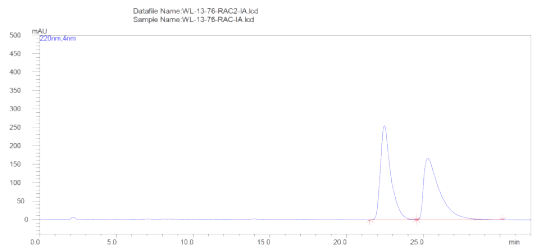

<Peak Table>

PDA Ch2 220nm

Peak#	Ret. Time	Area	Area%
1	22.466	12013440	50.045
2	25.292	11992024	49.955
Total		24005464	100.000

<Sample Information>

Sample Name : WL-13-76-CHIR-IA.lcd
Sample ID :
Data Filename : WL-13-76-CHIR-IA.lcd
Method Filename : wanglei2hao-90-10-1.5ml-45minX.lcm
Batch Filename : WWLL1.lcb
Vial # : 1-92 Sample Type : Unknown
Injection Volume : 3 uL
Date Acquired : 11/15/2022 11:05:08 AM Acquired by : System Administrator
Date Processed : 11/15/2022 11:44:43 AM Processed by : System Administrator

<Chromatogram>

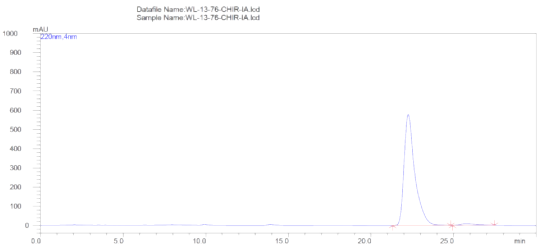

<Peak Table>

PDA Ch2 220nm

Peak#	Ret. Time	Area	Area%
1	22.256	26280725	97.841
2	25.771	579827	2.159
Total		26860552	100.000

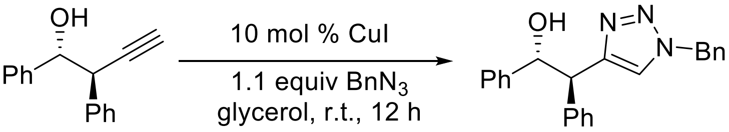

8 **10**

In a N_2-filled glove-box, an oven-dried 8 mL vial equipped with a magnetic stir bar was charged with **8** (44.4 mg, 0.2 mmol, 1.0 equiv), CuI (3.8 mg, 0.02 mmol, 10 mol %), BnN_3 (29.2 mg, 0.22 mmol, 1.1 equiv) and glycerol (2.0 mL). The vial was sealed with a cap with a septum, and the reaction mixture was vigorously stirred at room temperature (about 22 °C) for 12 h. After addition of 1.0 ml H_2O, the mixture is filtered through a short plug of 100-200 mesh silica gel eluting with EA (3×20 mL). The filtrate is concentrated under reduced pressure and the residue was purified by silica-gel column chromatography (eluent: Petroleum ether/EA = 2:1) to afford the **10** as white solid (68.4 mg, 96%).

(1*R*,2*R*)-2-(1-benzyl-1*H*-1,2,3-triazol-4-yl)-1,2-diphenylethan-1-ol (10)

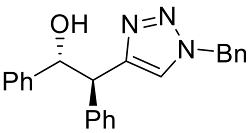

10

White solid, m.p. 131–133 °C; **IR (neat):** 3300 (w), 1494 (w), 1132 (m), 1069 (m), 719 (s), 696 (s) cm^{-1}; **^{1}H NMR (400 MHz, CDCl$_3$):** δ 7.38 – 7.29 (m, 3H), 7.22 – 7.09 (m, 11H), 7.06 (d, *J* = 7.2 Hz, 2H), 5.49 (d, *J* = 14.8 Hz, 1H), 5.40 (d, *J* = 14.8 Hz, 1H), 5.28 (d, *J* = 7.6 Hz, 1H), 4.72 (s, 1H), 4.29 (d, *J* = 7.6 Hz, 1H); **^{13}C NMR (100 MHz, CDCl$_3$):** δ 149.2, 141.8, 139.9, 134.4, 129.0, 128.7, 128.6, 128.2, 127.8, 127.7, 127.1, 126.7, 126.5, 122.7, 78.0, 54.0, 51.5; **HRMS (ESI$^+$) [M+Na]$^+$** Calcd for $C_{23}H_{21}N_3ONa$: 378.1577 m/z, Found: 378.1570 m/z; **Specific rotation**: $[\alpha]_D^{20}$ -2.78 (*c* 1.00, $CHCl_3$) for an enantiomerically enriched sample of 97.5:2.5 e.r.,

Enantiomeric purity of **10** was determined by SFC analysis in comparison with authentic racemic material (97.5:2.5 e.r. shown; Chiralpak IA column, 70:30 CO_2 / *i*-PrOH, 2.0 mL/min, 220 nm).

<Sample Information>

Sample Name : WL-13-80-ra-IA.lcd
Sample ID :
Data Filename : WL-13-80-RAC2-IA.lcd
Method Filename : wl-4-70-30-2.0-60xmin.lcm
Batch Filename : lwx_hydrosilylation.lcb
Vial # : 1-20 Sample Type : Unknown
Injection Volume : 20 uL
Date Acquired : 11/12/2022 5:00:43 AM Acquired by : System Administrator
Date Processed : 11/12/2022 6:00:46 AM Processed by : System Administrator

<Chromatogram>

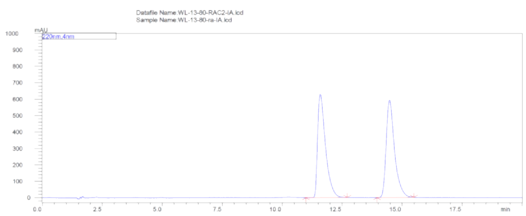

<Peak Table>

PDA Ch2 220nm

Peak#	Ret. Time	Area	Area%
1	11.589	14361395	49.948
2	14.476	14391391	50.052
Total		28752786	100.000

<Sample Information>

Sample Name : WL-13-80-chir-IA.lcd
Sample ID :
Data Filename : WL-13-80-chirx-IA.lcd
Method Filename : wl-4-70-30-2.0-60xmin.lcm
Batch Filename : lwx_hydrosilylation.lcb
Vial # : 1-19 Sample Type : Unknown
Injection Volume : 20 uL
Date Acquired : 11/14/2022 10:54:05 PM Acquired by : System Administrator
Date Processed : 11/14/2022 11:20:13 PM Processed by : System Administrator

<Chromatogram>

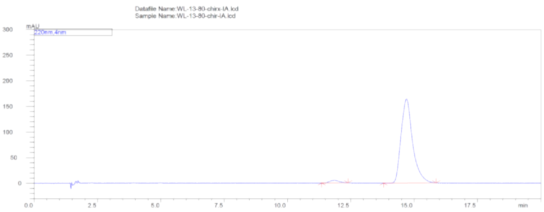

<Peak Table>

PDA Ch2 220nm

Peak#	Ret. Time	Area	Area%
1	11.851	133202	2.497
2	14.697	5202159	97.503
Total		5335361	100.000

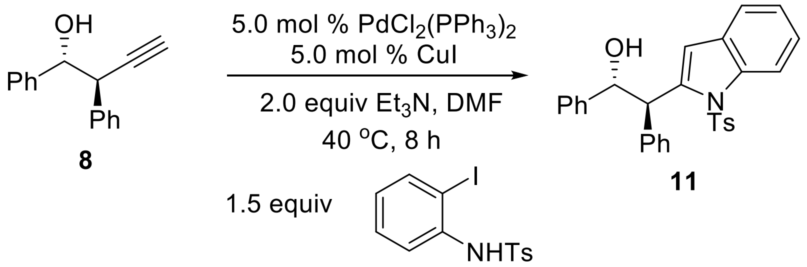

In a N_2-filled glove-box, an oven-dried 8 mL vial equipped with a magnetic stir bar was charged with **8** (44.4 mg, 0.2 mmol, 1.0 equiv), *N*-(2-iodophenyl)-4-methylbenzenesulfonamide (111.9 mg, 0.3 mmol, 1.5 equiv), $PdCl_2(PPh_3)_2$ (7.0 mg, 0.01 mmol, 5.0 mol %), CuI (1.9 mg, 0.01 mmol, 5.0 mol %), Et_3N (40.5 mg, 0.4 mmol, 2.0 equiv) and DMF (1.0 mL). The vial was sealed with a cap with a septum, and the mixture was allowed to stir at 40 °C for 8 h. The reaction mixture was quenched with H_2O (2.0 mL) and the resulting aqueous phase was extracted with EA (10 mL × 3). The combined organic phase was dried over Na_2SO_4. After removal of the solvent, the residue was purified by silica gel column chromatography (hexanes/EA = 10:1) to afford the **11** (86.2 mg, 92% yield) as white solid.

(1*R*,2*R*)-1,2-diphenyl-2-(1-tosyl-1*H*-indol-2-yl)ethan-1-ol (11)

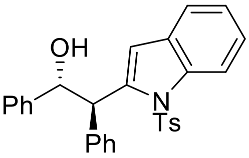

11

White solid, m.p. 140–142 °C; **IR (neat):** 3398 (w), 2179 (w), 1602 (w), 1050 (m), 842 (s), 760 (s), 698 (s) cm^{-1}; **^{1}H NMR (400 MHz, $CDCl_3$):** δ 8.06 (d, *J* = 7.6 Hz, 1H), 7.53 (d, *J* = 6.4 Hz, 1H), 7.29 – 7.15 (m, 9H), 7.16 – 7.05 (m, 4H), 7.05 – 6.98 (m, 2H), 6.91 (d, *J* = 8.0 Hz, 2H), 5.37 (d, *J* = 8.4 Hz, 1H), 5.16 (d, *J* = 8.4 Hz, 1H), 2.52 (s, 1H), 2.21 (s, 3H); **^{13}C NMR (100 MHz, $CDCl_3$):** δ 144.3, 141.2, 141.1, 138.8, 136.8, 135.5, 129.4, 129.4, 129.3, 128.0, 128.0, 127.6, 126.8, 126.7, 126.4, 124.3, 123.5, 120.6, 115.0, 110.1, 78.2, 52.4, 21.3; **HRMS (ESI$^+$) [M+Na]$^+$** Calcd for $C_{29}H_{25}NO_3NaS$: 490.1447 m/z, Found: 490.1444 m/z; **Specific rotation**: $[\alpha]_D^{20}$ -7.79 (*c* 1.00, $CHCl_3$) for an enantiomerically enriched sample of 97:3 e.r.,

Enantiomeric purity of **11** was determined by SFC analysis in comparison with authentic racemic material (97:3 e.r. shown; Chiralpak IC column, 80:20 CO_2 / *i*-PrOH, 2.0 mL/min, 220 nm).

<Sample Information>

Sample Name	: WL-13-82-rac-IC.lcd		
Sample ID	:		
Data Filename	: WL-13-82-rac-IC002.lcd		
Method Filename	: wl-1-80-20-2.0-55xmin.lcm		
Batch Filename	: lwx_hydrosilylation.lcb		
Vial #	: 1-21	Sample Type	: Unknown
Injection Volume	: 20 uL		
Date Acquired	: 11/14/2022 11:37:47 PM	Acquired by	: System Administrator
Date Processed	: 11/15/2022 12:27:37 AM	Processed by	: System Administrator

<Chromatogram>

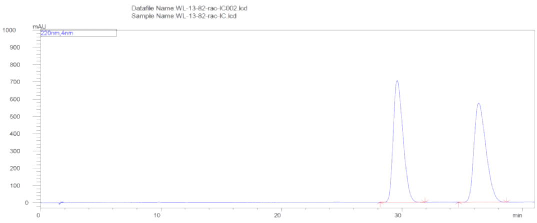

<Peak Table>

PDA Ch2 220nm

Peak#	Ret. Time	Area	Area%
1	29.597	39575207	50.003
2	36.363	39569701	49.997
Total		79144908	100.000

<Sample Information>

Sample Name	: WL-13-82-chir-IC.lcd		
Sample ID	:		
Data Filename	: WL-13-82-chir-IC.lcd		
Method Filename	: wl-1-80-20-2.0-55xmin.lcm		
Batch Filename	: lwx_hydrosilylation.lcb		
Vial #	: 1-18	Sample Type	: Unknown
Injection Volume	: 15 uL		
Date Acquired	: 11/14/2022 9:13:50 PM	Acquired by	: System Administrator
Date Processed	: 11/15/2022 12:11:47 AM	Processed by	: System Administrator

<Chromatogram>

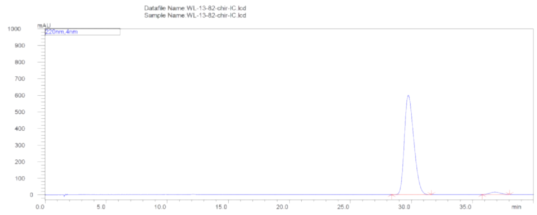

<Peak Table>

PDA Ch2 220nm

Peak#	Ret. Time	Area	Area%
1	29.746	32174492	97.402
2	36.820	858185	2.598
Total		33032677	100.000

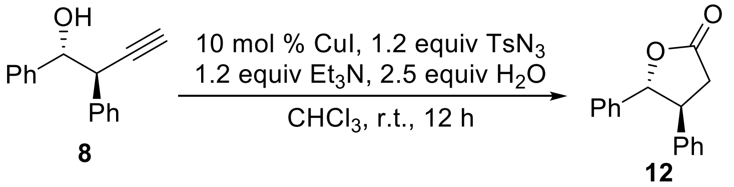

In a N_2-filled glove-box, an oven-dried 8 mL vial equipped with a magnetic stir bar was charged with **8** (44.4 mg, 0.2 mmol, 1.0 equiv), TsN_3 (47.3 mg, 0.24 mmol, 1.2 equiv), CuI (3.8 mg, 0.02 mmol, 10 mol %), Et_3N (24.3 mg, 0.24 mmol, 1.2 equiv), H_2O (9.0 mg, 0.5 mmol, 2.5 equiv) and $CHCl_3$ (0.4 mL). The vial was sealed with a cap with a septum, and the mixture was allowed to stir at room temperature (about 22 °C) for 12 h. The reaction mixture was quenched with aqueous saturated NH_4Cl (2.0 mL) and the resulting aqueous phase was extracted with DCM (10 mL×3). The combined organic phase was dried over Na_2SO_4. After removal of the solvent, the residue was purified by silica gel column chromatography (hexanes/EA = 6:1) to afford the **12** (34.7 mg, 73% yield) as white solid.

(4*S*,5*R*)-4,5-diphenyldihydrofuran-2(3*H*)-one (12)

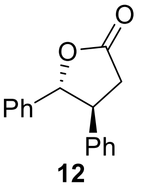

White solid, m.p. 97–99 °C; **IR (neat):** 2926 (w),1779 (s), 1512 (w), 1270 (m), 1032 (m), 842 (m), 763 (m) cm^{-1}; **^{1}H NMR (400 MHz, $CDCl_3$):** δ 7.45 – 7.27 (m, 6H), 7.24 – 7.06 (m, 4H), 5.43 (d, J = 8.4 Hz, 1H), 3.60 (q, J = 9.2 Hz, 1H), 3.06 (dd, J = 17.6, 8.4 Hz, 1H), 2.92 (dd, J = 17.6, 10.8 Hz, 1H); **^{13}C NMR (100 MHz, $CDCl_3$):** δ 175.3, 137.9, 137.7, 129.1, 128.6, 127.8, 127.3, 125.6, 87.4, 50.5, 37.1; **HRMS (EI$^+$) [M]$^+$** Calcd for $C_{16}H_{14}O_2$: 238.0988 m/z, Found: 238.0986 m/z; **Specific rotation**: $[\alpha]_D^{20}$ -73.98 (*c* 1.00, $CHCl_3$) for an enantiomerically enriched sample of 97:3 e.r.,

Enantiomeric purity of **12** was determined by SFC analysis in comparison with authentic racemic material (97:3 e.r. shown; Chiralpak IJ-3 column, 90:10 CO_2 / *i*-PrOH, 1.5 mL/min, 220 nm).

<Sample Information>

Sample Name : WL-13-77-ra-IJ3.lcd
Sample ID :
Data Filename : WL-13-77-RACX-IJ3.lcd
Method Filename : wl-6-90-10-1.5-35xmin.lcm
Batch Filename : lwx_hydrosilylation.lcb
Vial # : 1-16
Injection Volume : 15 uL
Date Acquired : 11/11/2022 9:24:20 AM
Date Processed : 11/14/2022 6:21:48 PM
Sample Type : Unknown
Acquired by : System Administrator
Processed by : System Administrator

<Chromatogram>

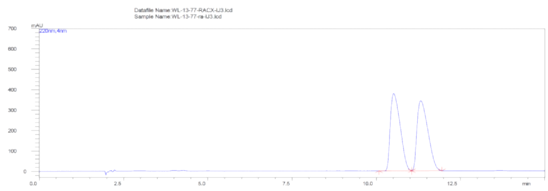

<Peak Table>

PDA Ch2 220nm

Peak#	Ret. Time	Area	Area%
1	10.519	7729095	49.999
2	11.323	7729451	50.001
Total		15458546	100.000

<Sample Information>

Sample Name : WL-13-77-chir-IJ3.lcd
Sample ID :
Data Filename : WL-13-77-chir-IJ4.lcd
Method Filename : wl-6-90-10-1.5-35xmin.lcm
Batch Filename : lwx_hydrosilylation.lcb
Vial # : 1-16
Injection Volume : 15 uL
Date Acquired : 11/14/2022 5:50:57 PM
Date Processed : 11/14/2022 6:25:13 PM
Sample Type : Unknown
Acquired by : System Administrator
Processed by : System Administrator

<Chromatogram>

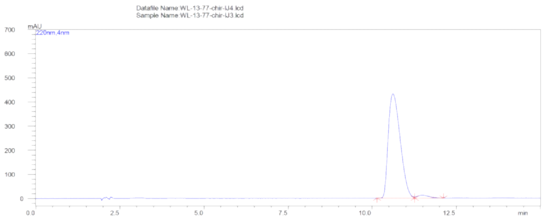

<Peak Table>

PDA Ch2 220nm

Peak#	Ret. Time	Area	Area%
1	10.618	10672519	97.328
2	11.477	293026	2.672
Total		10965545	100.000

■ Experimental Procedure and Characterization for Mechanistic Studies

Secondary isotope effect

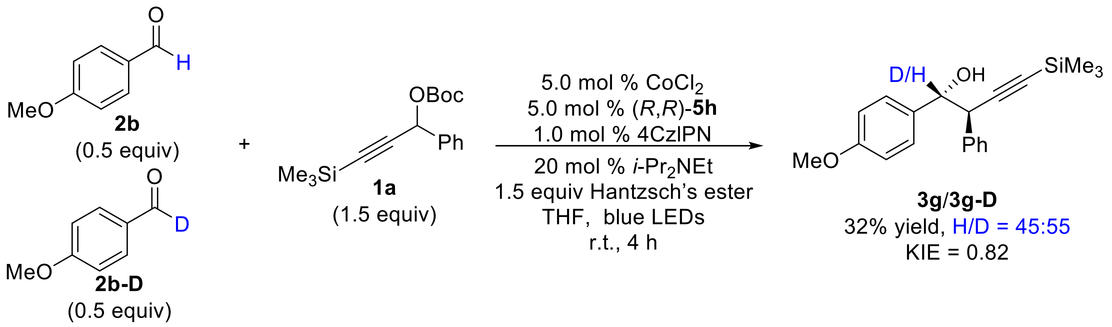

In a N_2-filled glove-box, an oven-dried vial (8 mL) equipped with a magnetic stir bar was charged with $CoCl_2$ (2.6 mg, 0.02 mmol, 5.0 mol %), (*R*,*R*)-**5h** (6.2 mg, 0.02 mmol, 5.0 mol %). THF (3.0 mL) was added, then the mixture was allowed to stir at room temperature for 20 min. **2b/2b-D (**1:1, 0.4 mmol), **1a** (182.6 mg, 0.6 mmol, 1.5 equiv), *i*-Pr_2NEt (10.3 mg, 0.08 mmol, 0.2 equiv), 4CzIPN (3.1 mg, 0.004 mmol, 1.0 mol %) and Hantzsch's ester (151.9 mg, 0.6 mmol, 1.5 equiv) were added to the solution. The vial was sealed with a cap (phenolic open top cap with red PTFE/white silicone septum) and taken out of the glove box. It was irradiated by 40 W blue LEDs (450-455 nm) and allowed to stir at room temperature (about 22 ºC) for 4 h with cooling fans.

Workup: The mixture is filtered through a short plug of 100-200 mesh silica gel eluting with diethyl ether (3×30 mL). The filtrate is concentrated under reduced pressure and the residue was purified by silica-gel column chromatography (eluent: Petroleum ether/ diethyl ether = 10:1) to afford the **3g/3g-D** as colorless oil (41.6 mg, 32%).

EPR studies

Electron paramagnetic resonance (EPR) spectra were recorded at room temperature on a Bruker E500-10/12.

(1) Background

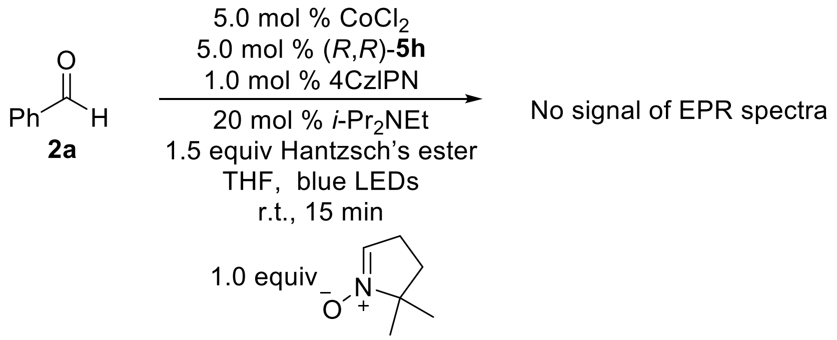

In a N_2-filled glove-box, an oven-dried vial (8 mL) equipped with a magnetic stir bar was charged with $CoCl_2$ (2.6 mg, 0.02 mmol, 5.0 mol %), (*R*, *R*)-**5h** (6.2 mg, 0.02 mmol, 5.0 mol %). THF (3.0 mL) was added, then the mixture was allowed to stir at room temperature for 20 min. **2a** (42.3 mg, 0.4 mmol, 1.0 equiv), *i*-Pr_2NEt (10.3 mg, 0.08 mmol, 0.2 equiv), DMPO (45.2 mg, 0.4 mmol, 1.0 equiv), 4CzIPN (3.1 mg, 0.004 mmol, 1.0 mol %) and Hantzsch's ester (151.9 mg, 0.6 mmol, 1.5 equiv) were added to the solution. The vial was sealed with a cap (phenolic open top cap with red PTFE/white silicone septum) and taken out of the glove box. It was irradiated by 40 W blue LEDs (450-455 nm) and allowed to stir at room temperature (about 22 ^{o}C) for 15 min with cooling fans. The reaction mixture was transferred to quartz EPR tube and the spectra was measured at room temperature (**Supplementary Figure 5**). No signal was observed in the EPR spectra, which indicated that no radical species in the reaction in the absence of propargylic carbonate.

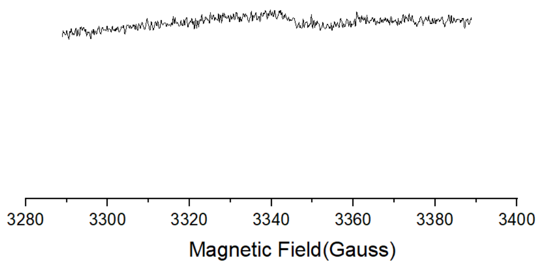

Supplementary Figure 5. EPR spectra in the absence of propargylic carbonate

(2) EPR studies of standard reaction

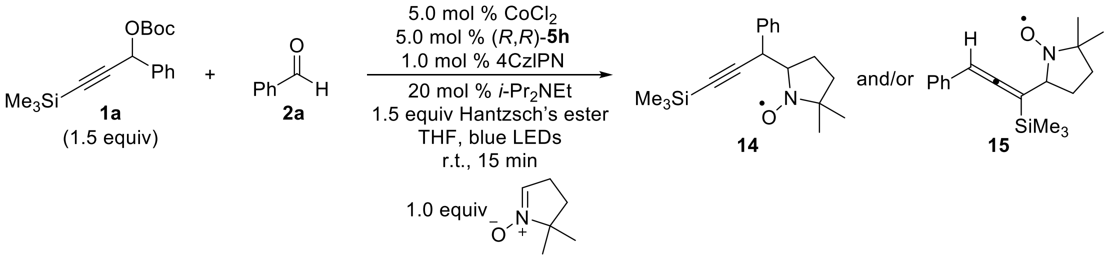

In a N_2-filled glove-box, an oven-dried vial (8 mL) equipped with a magnetic stir bar was charged with $CoCl_2$ (2.6 mg, 0.02 mmol, 5.0 mol %), (*R*, *R*)-**5h** (6.2 mg, 0.02 mmol,

5.0 mol %). THF (3.0 mL) was added, then the mixture was allowed to stir at room temperature for 20 min. **2a** (42.3 mg, 0.4 mmol, 1.0 equiv), **1a** (182.6 mg, 0.6 mmol, 1.5 equiv), *i*-Pr_2NEt (10.3 mg, 0.08 mmol, 0.2 equiv), DMPO (45.2 mg, 0.4 mmol, 1.0 equiv), 4CzIPN (3.1 mg, 0.004 mmol, 1.0 mol %) and Hantzsch's ester (151.9 mg, 0.6 mmol, 1.5 equiv) were added to the solution. The vial was sealed with a cap (phenolic open top cap with red PTFE/white silicone septum) and taken out of the glove box. It was irradiated by 40 W blue LEDs (450-455 nm) and allowed to stir at room temperature (about 22 ^{o}C) for 15 min with cooling fans. The reaction mixture was transferred to quartz EPR tube and the spectra was measured at room temperature (**Supplementary Figure 6**).

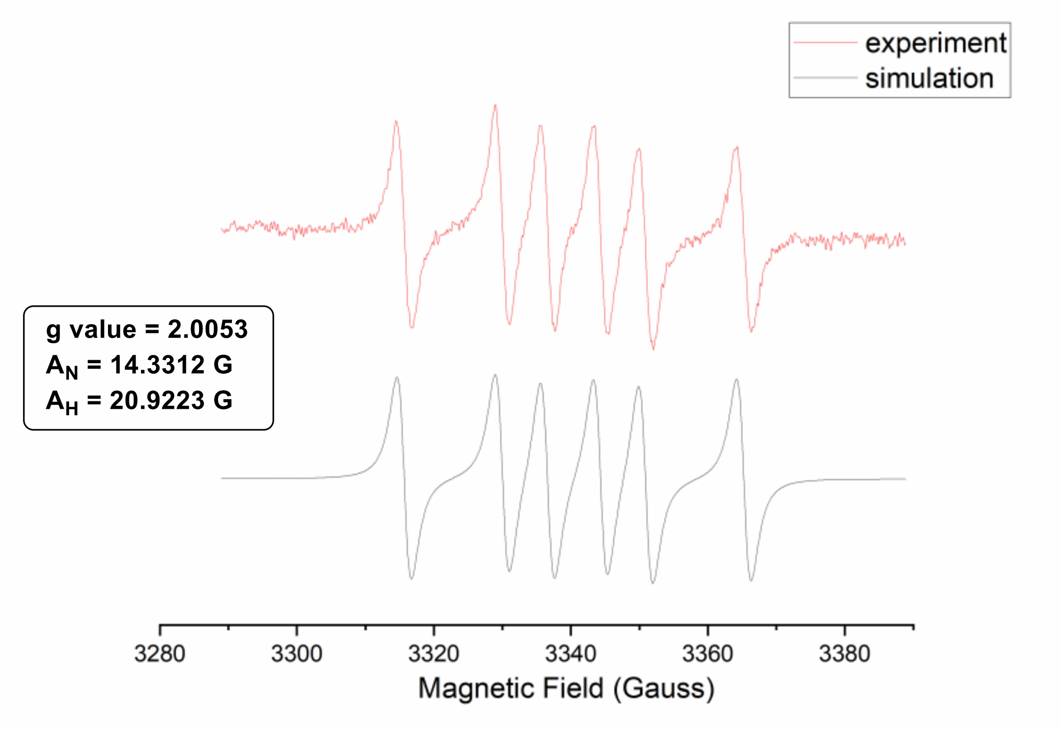

Supplementary Figure 6. EPR spectra of standard reaction

The existence of **14** and/or **15** was further detected by HRMS analysis of reaction mixture **(Supplementary Figure 7)**. **HRMS (ESI$^+$) [M+Na]$^+$** Calcd for $C_{18}H_{26}NONaSi$: 323.16759 m/z, Found: 323.16724 m/z.

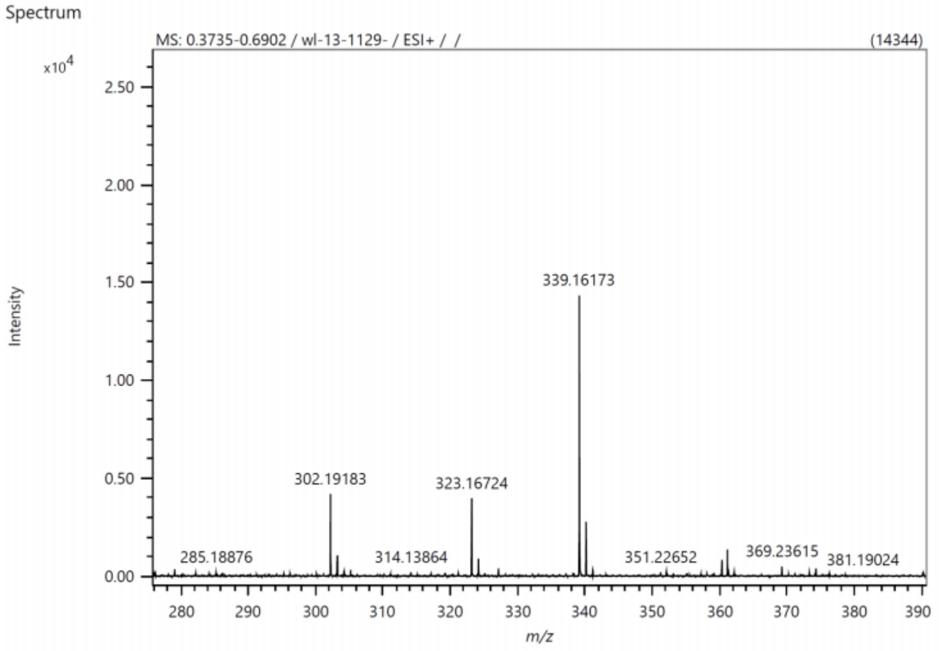

Elemental Composition

Parameters	
Tolerance:	±5.00 ppm
Electron:	Odd/Even
Charge:	+1
DBE:	-1.5 - 200.0

Elements Set 1:

Symbol	C	H	N	O	Na	S	Cl	Br
Min	0	0	0	0	1	0	0	0
Max	200	200	3	8	1	0	0	0

Symbol	Si	F	P
Min	1	0	0
Max	1	0	0

Results

Mass	Intensity	Intensity [%]	Formula	Calculated Mass	Mass Difference [mDa]	Mass Difference [ppm]	DBE
323.16724	3982.22	1.63	C18 H26 N O Na Si	323.16759	-0.35	-1.07	7.0

Supplementary Figure 7. HRMS analysis

The synthesis of 13 and 13-D

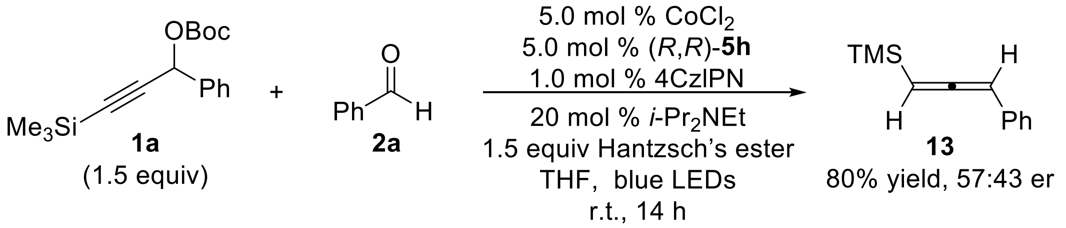

In a N_2-filled glove-box, an oven-dried vial (8 mL) equipped with a magnetic stir bar was charged with $CoCl_2$ (2.6 mg, 0.02 mmol, 5.0 mol %), (*R*, *R*)-**5h** (6.2 mg, 0.02 mmol, 5.0 mol %). THF (3.0 mL) was added, then the mixture was allowed to stir at room temperature for 20 min. **2a** (42.3 mg, 0.4 mmol, 1.0 equiv), **1a** (182.6 mg, 0.6 mmol, 1.5 equiv), *i*-Pr_2NEt (10.3 mg, 0.08 mmol, 0.2 equiv), 4CzIPN (3.1 mg, 0.004 mmol, 1.0 mol %) and Hantzsch's ester (151.9 mg, 0.6 mmol, 1.5 equiv) were added to the solution. The vial was sealed with a cap (phenolic open top cap with red PTFE/white silicone septum) and taken out of the glove box. It was irradiated by 40 W blue LEDs

(450-455 nm) and allowed to stir at room temperature(about 22 °C) for 14 h with cooling fans.

Workup: The mixture is filtered through a short plug of 100-200 mesh silica gel eluting with diethyl ether (3×30 mL). The filtrate is concentrated under reduced pressure and the residue was purified by silica-gel column chromatography (eluent: Petroleum ether) to afford the **13** as colorless oil (30.2 mg, 0.16 mmol).

Trimethyl(3-phenylpropa-1,2-dien-1-yl) silane (13)

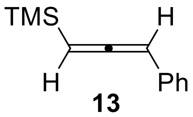

13

Colorless oil. **^{1}H NMR (400 MHz, $CDCl_3$):** δ 7.38 – 7.18 (m, 4H), 7.18 – 7.09 (m, 1H), 5.86 (d, J = 6.8 Hz, 1H), 5.42 (d, J = 6.8 Hz, 1H), 0.17 (s, 9H); **^{13}C NMR (100 MHz, $CDCl_3$):** δ 210.2, 135.1, 128.6, 125.9, 87.9, 87.0, -0.8; **Specific rotation**: $[\alpha]_D^{20}$ -7.87 (*c* 1.00, $CHCl_3$) for an enantiomerically enriched sample of 57:43 e.r.,

Enantiomeric purity of **13** was determined by SFC analysis in comparison with authentic racemic material (57:43 e.r. shown; Chiralcel OJ-H column, 99.9:0.1 CO_2 / *i*-PrOH, 0.8 mL/min, 254 nm).

<Sample Information>

Sample Name : WL-13-87-RAC-OJH.lcd
Sample ID :
Data Filename : WL-13-87-RAC-OJH.lcd
Method Filename : wl-5-99.9-0.1-0.8-30min.lcm
Batch Filename : lwx_hydrosilylation.lcb
Vial # : 1-49
Injection Volume : 0.4 uL
Date Acquired : 11/18/2022 4:29:11 AM
Date Processed : 11/18/2022 10:27:40 AM
Sample Type : Unknown
Acquired by : System Administrator
Processed by : System Administrator

<Chromatogram>

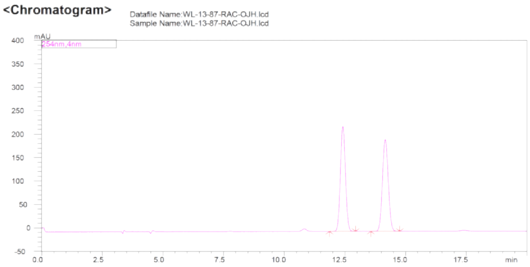

<Peak Table>

PDA Ch1 254nm

Peak#	Ret. Time	Area	Area%
1	12.414	3274325	50.017
2	14.164	3272048	49.983
Total		6546373	100.000

<Sample Information>

Sample Name : WL-13-87-CHIR-OJH.lcd
Sample ID :
Data Filename : WL-13-87-CHIR-OJH.lcd
Method Filename : wl-5-99.9-0.1-0.8-30min.lcm
Batch Filename : lwx_hydrosilylation.lcb
Vial # : 1-48
Injection Volume : 0.4 uL
Date Acquired : 11/18/2022 9:24:00 AM
Date Processed : 12/12/2022 3:58:55 AM
Sample Type : Unknown
Acquired by : System Administrator
Processed by : System Administrator

<Chromatogram>

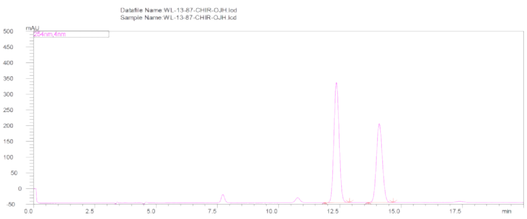

<Peak Table>

PDA Ch1 254nm

Peak#	Ret. Time	Area	Area%
1	12.359	5627008	57.181
2	14.109	4213619	42.819
Total		9840627	100.000

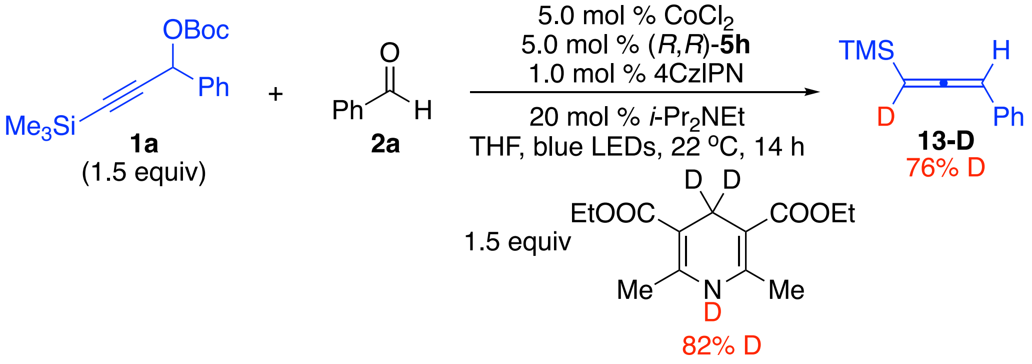

In a N_2-filled glove-box, an oven-dried vial (8 mL) equipped with a magnetic stir bar was charged with $CoCl_2$ (2.6 mg, 0.02 mmol, 5.0 mol %), (*R*, *R*)-**5h** (6.2 mg, 0.02 mmol, 5.0 mol %). THF (3.0 mL) was added, then the mixture was allowed to stir at room temperature for 20 min. **2a** (42.3 mg, 0.4 mmol, 1.0 equiv), **1a** (182.6 mg, 0.6 mmol, 1.5 equiv), *i*-Pr_2NEt (10.3 mg, 0.08 mmol, 0.2 equiv), 4CzIPN (3.1 mg, 0.004 mmol, 1.0 mol %) and ***d*3-HE** (153.7 mg, 0.6 mmol, 1.5 equiv) were added to the solution. The

vial was sealed with a cap (phenolic open top cap with red PTFE/white silicone septum) and taken out of the glove box. It was irradiated by 40 W blue LEDs (450-455 nm) and allowed to stir at room temperature (about 22 °C) for 14 h with cooling fans.

Workup: The mixture is filtered through a short plug of 100-200 mesh silica gel eluting with diethyl ether (3×30 mL). The filtrate is concentrated under reduced pressure and the residue was purified by silica-gel column chromatography (eluent: Petroleum ether) to afford the **13-D** as colorless oil (76% D, 23.8 mg, 0.13 mmol). Based on ^{1}H NMR and ^{2}H NMR analysis of **13-D**, deuterium scrambling existed in this reaction.

Trimethyl(3-phenylpropa-1,2-dien-1-yl-1-*d*)silane (13-D)

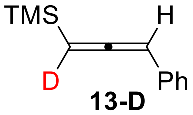

13-D
76% D

Colorless oil. **^{1}H NMR (400 MHz, CDCl$_3$):** δ 7.32 – 7.20 (m, 4H), 7.18 – 7.09 (m, 1H), 6.38 – 5.56 (m, 1H), 5.42 (d, *J* = 6.8 Hz, 0.24H), 0.17 (s, 9H); **^{2}H NMR (61 MHz, CHCl$_3$)** δ 5.49.

The reaction of (*rac*)-1a with (*R*,*R*)-5h monitored over different reaction time

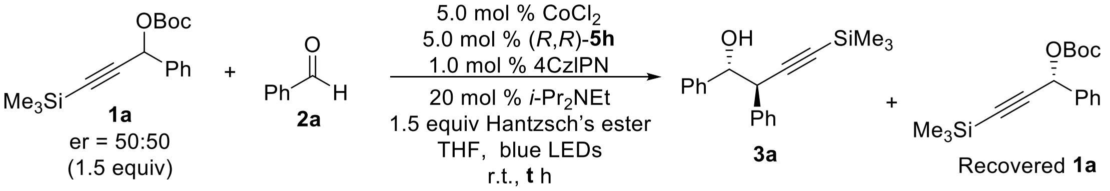

Time (h)	Yield (%) of **3a**	ee (%) of **3a**	ee (%) of recovered **1a**
2	14	94	2 (*R*)
3.5	26	94	4 (*R*)
5	40	94	6 (*R*)
6.5	56	92	8 (*R*)
8	76	94	14 (*R*)

The reaction of (*R*)-1a with (*R*,*R*)-5h monitored over different reaction time

(*R*)-**1a** was prepared according to a previous reported procedure[5]

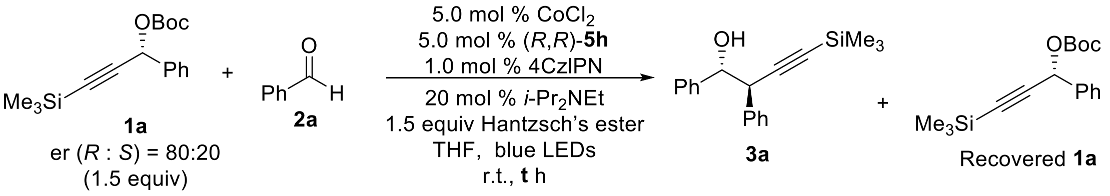

Time (h)	Yield (%) of **3a**	ee (%) of **3a**	ee (%) of recovered **1a**

2	14	94	62 (*R*)
3.5	26	94	64 (*R*)
5	40	94	66 (*R*)
6.5	56	92	68 (*R*)

The reaction of (*R*)-1a with (*S*,*S*)-5h monitored over different reaction time

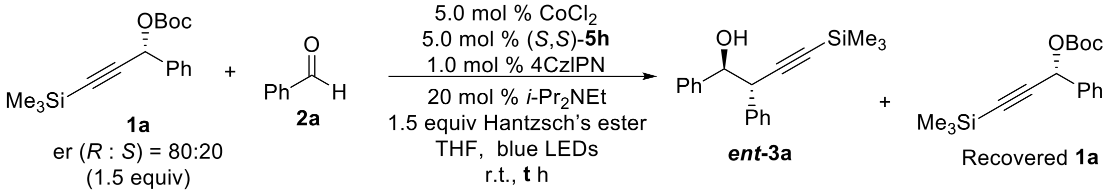

Time (h)	Yield (%) of **3a**	ee (%) of ***ent*-3a**	ee (%) of recovered **1a**
2	15	92	58 (*R*)
3.5	27	91	57 (*R*)
5	38	92	55 (*R*)
6.5	58	93	54 (*R*)

■ Computational Details

All of the calculations were performed using the Gaussian 16 program.[6] Structures were optimized at the (U)B3LYP level of density functional theory[7] with Grimme's D3(BJ) dispersion correction[8] in gas phase. For optimizations, Ahlrichs's def2SVP basis set was used for all atoms.[9] Frequency calculations have been performed to verify the optimized structures as local minima or transition state and to obtain Gibbs free energy at 298 K. To reduce error caused by the breakdown of the harmonic oscillator approximation, Truhlar's quasi-harmonic correction was used to compute molecular entropies by setting all positive frequencies that are less than 100 cm^{-1} to 100 cm^{-1}.[10] A correction of -1.9 (or 1.9) kcal/mol to free energies at T = 298.15 K was made for two-to-one (or one-to-two) molecularity transformations on the basis of theory of free volume. Intrinsic reaction coordinate (IRC) calculations were carried out to make sure that every transition state links relevant intermediates.[11] The electronic energies were further refined by carrying out single-point energy calculations using (U)B3LYP functional with Grimme's D3(BJ) dispersion correction. The def2TZVP basis set was

applied for all atoms.[9] The SMD solvation model with THF as the solvent was employed to account for solvation effect.[12] The three-dimensional (3D) structures were depicted using CYLview software.

Additional Computational Results

Supplementary Table 4. Distortion/interaction analysis for stereo-determining transition states

	^{2}**TS2**$_{top\text{-}RR}$	^{2}**TS2**$_{btm\text{-}SS}$	^{2}**TS2**$_{btm\text{-}RS}$	^{2}**TS2**$_{top\text{-}SR}$
$\Delta\Delta E_{dist}$(cat)	**0.0**	**3.8**	**3.8**	**4.2**
$\Delta\Delta E_{dist}$(sub)[a]	0.0	-0.8	2.8	4.1
$\Delta\Delta E_{dist}$(total)	0.0	3.0	6.6	8.3
ΔE_{int}	-34.0	-34.2	-36.3	-36.9
$\Delta\Delta E_{int}$	0.0	-0.2	-2.3	-3.0
$\boldsymbol{E^{\ddagger}}$	**0.0**	**2.8**	**4.3**	**5.4**

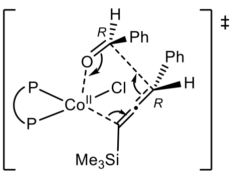

^{2}**TS2**$_{top\text{-}RR}$

$\Delta\Delta G^{\ddagger}$ = 0.0
$\Delta\Delta E^{\ddagger}$ = 0.0

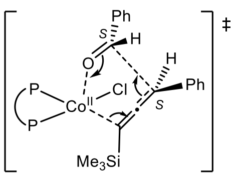

^{2}**TS2**$_{top\text{-}SS}$

$\Delta\Delta G^{\ddagger}$ = 6.2
$\Delta\Delta E^{\ddagger}$ = 6.5

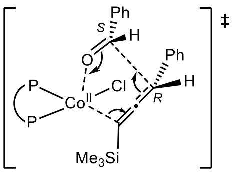

^{2}**TS2**$_{top\text{-}RS}$

$\Delta\Delta G^{\ddagger}$ = 5.9
$\Delta\Delta E^{\ddagger}$ = 6.0

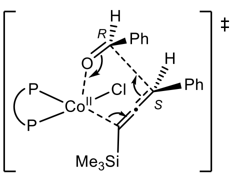

^{2}**TS2**$_{top\text{-}SR}$

$\Delta\Delta G^{\ddagger}$ = 5.5
$\Delta\Delta E^{\ddagger}$ = 5.4

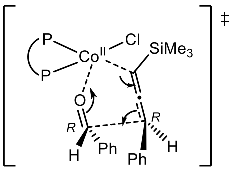

^{2}**TS2**$_{btm\text{-}RR}$

$\Delta\Delta G^{\ddagger}$ = 6.8
$\Delta\Delta E^{\ddagger}$ = 6.9

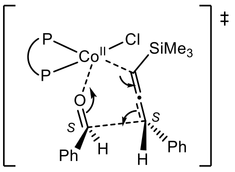

^{2}**TS2**$_{btm\text{-}SS}$

$\Delta\Delta G^{\ddagger}$ = 3.3
$\Delta\Delta E^{\ddagger}$ = 2.8

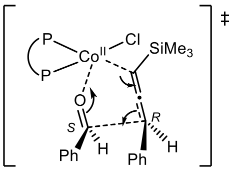

^{2}**TS2**$_{btm\text{-}RS}$

$\Delta\Delta G^{\ddagger}$ = 4.9
$\Delta\Delta E^{\ddagger}$ = 4.3

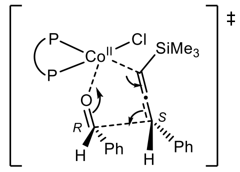

^{2}**TS2**$_{btm\text{-}SR}$

$\Delta\Delta G^{\ddagger}$ = 7.4
$\Delta\Delta E^{\ddagger}$ = 7.4

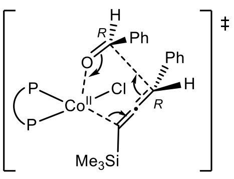

^{4}**TS2**$_{top\text{-}RR}$

$\Delta\Delta G^{\ddagger}$ = 2.3
$\Delta\Delta E^{\ddagger}$ = 4.5

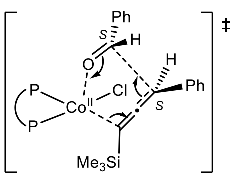

^{4}**TS2**$_{top\text{-}SS}$

$\Delta\Delta G^{\ddagger}$ = 4.8
$\Delta\Delta E^{\ddagger}$ = 7.0

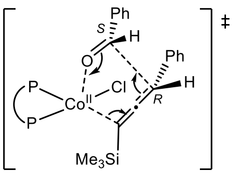

^{4}**TS2**$_{top\text{-}RS}$

$\Delta\Delta G^{\ddagger}$ = 3.7
$\Delta\Delta E^{\ddagger}$ = 6.0

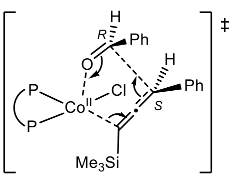

^{4}**TS2**$_{top\text{-}SR}$

$\Delta\Delta G^{\ddagger}$ = 3.4
$\Delta\Delta E^{\ddagger}$ = 5.6

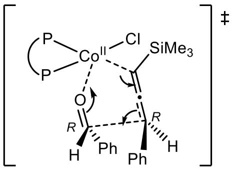

^{4}**TS2**$_{btm\text{-}RR}$

$\Delta\Delta G^{\ddagger}$ = 5.6
$\Delta\Delta E^{\ddagger}$ = 7.4

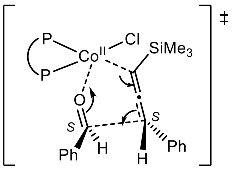

^{4}**TS2**$_{btm\text{-}SS}$

$\Delta\Delta G^{\ddagger}$ =6.0
$\Delta\Delta E^{\ddagger}$ = 7.6

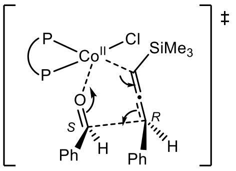

^{4}**TS2**$_{btm\text{-}RS}$

$\Delta\Delta G^{\ddagger}$ = 3.1
$\Delta\Delta E^{\ddagger}$ = 5.1

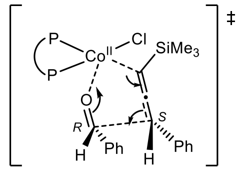

^{4}**TS2**$_{btm\text{-}SR}$

$\Delta\Delta G^{\ddagger}$ = 6.1
$\Delta\Delta E^{\ddagger}$ = 8.1

Supplementary Figure 8. Calculated energy difference for all possible nucleophilic addition transition states for both doublet state and quartet state. All energies are given in kcal/mol. The configurations with aldehyde approaching from bottom is the same with exchanging Cl and allenyl group due to C2 symmetry of the ligand.

Supplementary Table 5. Benchmark of different density functional for calculating the spin-splitting energy of **IM3**. Based on the optimized geometry at the level of B3LYP-D3BJ/def2SVP theory, single point energy calculations were performed using def2TZVP basis set together with SMD(THF) accounting for solvation effect.

	B3LYP-D3BJ	MN15L	M06L	TPSSh
ΔG(^{4}**IM3**)-ΔG(^{2}**IM3**)	-0.2	-2.5	3.0	4.5

Remark on **Supplementary Table 5**: We examined several commonly used density functionals to calculate the spin-splitting energy in our system. While there were slight differences in the calculated spin-splitting energies across the different

functionals, both doublet and quartet states are close in energy. The results presented in the manuscript were obtained using B3LYP-D3BJ, which closely aligned with the results obtained using MN15L. MN15L is recognized as one of the most suitable functionals for calculations involving multi-spin states.

Supplementary Table 6. Comparison of calculated free energy difference among different configurations of stereo-determining transition states using B3LYP-D3BJ and MN15L.

	B3LYP-D3BJ	MN15L
$^{2}\mathbf{TS}_{\mathbf{top}\text{-}\boldsymbol{RR}}$	0.0	0.0
$^{2}\mathbf{TS}_{\mathbf{top}\text{-}\boldsymbol{SS}}$	6.2	7.3
$^{2}\mathbf{TS}_{\mathbf{top}\text{-}\boldsymbol{RS}}$	5.9	9.4
$^{2}\mathbf{TS}_{\mathbf{top}\text{-}\boldsymbol{SR}}$	5.5	6.0
$^{2}\mathbf{TS}_{\mathbf{btm}\text{-}\boldsymbol{RR}}$	6.8	8.7
$^{2}\mathbf{TS}_{\mathbf{btm}\text{-}\boldsymbol{SS}}$	3.3	2.0
$^{2}\mathbf{TS}_{\mathbf{btm}\text{-}\boldsymbol{RS}}$	4.9	5.2
$^{2}\mathbf{TS}_{\mathbf{btm}\text{-}\boldsymbol{SR}}$	7.4	9.7
$^{4}\mathbf{TS}_{\mathbf{top}\text{-}\boldsymbol{RR}}$	2.3	5.6
$^{4}\mathbf{TS}_{\mathbf{top}\text{-}\boldsymbol{SS}}$	4.8	11.3
$^{4}\mathbf{TS}_{\mathbf{top}\text{-}\boldsymbol{RS}}$	3.7	9.7
$^{4}\mathbf{TS}_{\mathbf{top}\text{-}\boldsymbol{SR}}$	3.4	5.5
$^{4}\mathbf{TS}_{\mathbf{btm}\text{-}\boldsymbol{RR}}$	5.6	8.4
$^{4}\mathbf{TS}_{\mathbf{btm}\text{-}\boldsymbol{SS}}$	6.0	8.0
$^{4}\mathbf{TS}_{\mathbf{btm}\text{-}\boldsymbol{RS}}$	3.1	1.5
$^{4}\mathbf{TS}_{\mathbf{btm}\text{-}\boldsymbol{SR}}$	6.1	7.3

Remark on **Supplementary Table 6**: The relative stabilities of different configurations of stereo-determining transition states exhibit similar trend when using either B3LYP-D3BJ or MN15L.

XYZ coordinate

2a

E = -345.743233767 G = -345.663662

6	2.21806000	-0.25218200	-0.00004400
6	1.32552500	-1.33293800	0.00012300
6	-0.04736400	-1.10100000	0.00001100
6	-0.53509000	0.21645100	-0.00003000
6	0.36156400	1.29431200	-0.00002800
6	1.73788300	1.06103800	-0.00009700
1	3.29516000	-0.43682600	-0.00002700
1	1.70878600	-2.35604400	0.00024400
1	-0.76890300	-1.92066700	0.00008100
1	-0.02653700	2.31714300	0.00008400
1	2.43739400	1.90002800	-0.00023700
6	-1.99670700	0.46677800	0.00021000
8	-2.84373200	-0.39637500	-0.00024500
1	-2.27927600	1.55260600	0.00094700

2INT1

E = -3462.33560238 G = -3461.842194

27	0.60556100	-0.84528600	-0.05078500
17	0.35849900	-3.09874800	-0.12627900
6	-2.82776500	3.74495000	0.05914600
6	-1.49189900	3.34467300	0.00737700
6	-1.15959900	1.98112300	0.00107600
6	-2.18619000	1.01476200	0.04093100
6	-3.52666900	1.42710300	0.09597000
6	-3.84593000	2.78551700	0.10480500
1	-3.07793300	4.80837800	0.06612600
1	-0.70391400	4.10018300	-0.02621700
1	-4.32758700	0.68523900	0.13014900
1	-4.89163900	3.09910400	0.14520000
6	1.42366300	2.24693500	-1.42890700
6	1.53633000	2.14397500	1.32443900
6	2.66947000	2.85070300	-0.75430800
1	0.74015600	3.06503800	-1.71367800
6	2.27288500	3.31374700	0.65170800
1	2.27169800	1.34870500	1.52728600
1	3.43661500	2.06555400	-0.67454000
1	3.08568400	3.66982100	-1.36306300
1	3.14961000	3.61766700	1.24717400
1	1.60855400	4.19533100	0.59215300
6	-2.56419900	-1.58539500	1.40538500
6	-2.57061000	-1.62626500	-1.35642100
6	-3.23929400	-2.78992200	0.72352200
1	-3.34486200	-0.86759000	1.70990900
6	-3.74238100	-2.32964800	-0.64869700
1	-1.83612600	-2.39564400	-1.64406100
1	-2.48821200	-3.58491100	0.59156800
1	-4.04971700	-3.18975100	1.35454000
1	-4.11257400	-3.17167900	-1.25615700
1	-4.58738400	-1.62702000	-0.52958900
15	0.56064100	1.33096600	-0.04192900
15	-1.64346000	-0.74295300	0.00752900
6	1.71228600	1.36502500	-2.63930900
1	0.78141000	1.00635800	-3.10658700
1	2.27783600	1.92616900	-3.40125400
1	2.29610000	0.48852800	-2.32535300
6	-1.68382500	-1.92352700	2.60461100
1	-1.25930700	-1.01405200	3.05904100
1	-2.27270200	-2.44209700	3.37878400
1	-0.85198200	-2.57335500	2.29666200
6	0.75903200	2.46885400	2.59221300
1	1.43962000	2.84583400	3.37270900
1	-0.01083300	3.23649700	2.41811900
1	0.25625900	1.57430100	2.99279300
6	-2.92387400	-0.77859700	-2.57100200
1	-3.38247200	-1.40693600	-3.35151000
1	-3.63604600	0.02298000	-2.32243700
1	-2.02747200	-0.30882000	-3.00549100
8	2.41570400	-0.52781200	-0.07105100
6	3.52303500	-1.39520800	0.05503900
6	3.61628400	-2.32958800	-1.16086200
6	4.77061000	-0.49559500	0.10656000
6	3.42076300	-2.20251900	1.35820500
1	2.71863600	-2.95827600	-1.22442500
1	3.69227200	-1.73353100	-2.08489500
1	4.50330700	-2.98170100	-1.09898300
1	4.70285900	0.20065300	0.95795600
1	5.69374400	-1.08760500	0.21588300
1	4.85008200	0.09824400	-0.81789900
1	4.31319200	-2.83071100	1.51472800
1	3.32825200	-1.51389800	2.21417300
1	2.53414800	-2.84989900	1.33467300

2TSbtm-RR

E = -4330.96228270 G = -4330.266644

27	-0.57588100	0.21634400	0.59754800
17	-0.86910200	2.02138000	2.00762500

6	0.89377700	-0.53199800	1.95885100	1	0.46343900	-5.08517900	-0.07881700
14	0.42989700	-0.82734800	3.74650200	1	2.02533000	-3.80132700	-1.74877200
6	-1.43133300	-0.82566500	4.02987700	1	0.37858600	-4.06906400	-2.35079100
1	-1.64378400	-1.09370900	5.07838800	6	-2.81710800	2.53677500	-0.93292900
1	-1.82063100	0.18423300	3.83725900	6	-3.96611400	0.84888300	0.93026300
1	-1.96888300	-1.53409400	3.38463300	6	-3.83683600	3.15326500	0.04461400
6	1.21546000	0.56456000	4.73404200	1	-3.31764300	2.35938500	-1.89947200
1	0.83477900	1.52526400	4.35593700	6	-4.81009100	2.05537200	0.48686400
1	0.97336600	0.47974400	5.80653700	1	-3.47008700	1.11051600	1.87825400
1	2.31119200	0.55507200	4.62114300	1	-3.29055400	3.53261400	0.92288200
6	1.18112000	-2.48777700	4.24818100	1	-4.35414800	4.00704300	-0.42244200
1	2.27046800	-2.47775900	4.08278500	1	-5.46823900	2.39222200	1.30475900
1	0.99948600	-2.68425100	5.31815400	1	-5.46967100	1.76507500	-0.35092700
1	0.76094300	-3.32919100	3.67606000	15	-0.73591200	-1.63137300	-0.63446600
6	1.87817600	-0.18645400	1.27484500	15	-2.51883300	0.81541900	-0.25421600
6	2.85993700	0.31775400	0.40349400	6	-1.44756400	-3.48868500	1.44353300
1	3.32125000	1.22442300	0.81335100	1	-2.44740600	-3.03657900	1.52998000
6	3.87085100	-0.62603700	-0.15872200	1	-1.52871000	-4.54369400	1.75158000
6	4.92444200	-0.12326100	-0.94391900	1	-0.78508200	-2.98006600	2.15334300
6	3.79446000	-2.01599900	0.03171400	6	-1.56363500	3.37174100	-1.16848100
6	5.84891500	-0.98273200	-1.53905600	1	-0.82448600	2.83044200	-1.77373200
1	5.01397000	0.95589900	-1.08614500	1	-1.83061600	4.31222000	-1.67779300
6	4.71721800	-2.87719100	-0.56536200	1	-1.08116400	3.61286200	-0.21322200
1	3.00634800	-2.41888600	0.67008600	6	0.75481600	-1.45601700	-3.09675500
6	5.74689200	-2.36618000	-1.36062300	1	1.73990000	-1.61231100	-3.56493400
1	6.65813200	-0.56840500	-2.14590600	1	-0.00178500	-1.93622600	-3.73504200
1	4.63724800	-3.95457400	-0.39738800	1	0.54957600	-0.37742000	-3.06463400
1	6.47097200	-3.03832800	-1.82695100	6	-4.70953000	-0.47003100	1.09171100
6	-3.60898400	-2.24189500	-3.57458200	1	-5.48868000	-0.37870800	1.86551700
6	-2.49986900	-2.42392000	-2.74894300	1	-5.19636700	-0.78356300	0.15540600
6	-2.18234000	-1.47425600	-1.76461400	1	-4.02707700	-1.27671000	1.40244400
6	-2.99541500	-0.33684800	-1.60821700	6	3.17899700	5.23829700	-0.90877800
6	-4.11140100	-0.16325200	-2.44223600	6	2.24564400	4.76574500	0.02288000
6	-4.41622400	-1.10898600	-3.42108500	6	1.81670400	3.43949800	-0.01653500
1	-3.84429600	-2.98281200	-4.34210600	6	2.32473700	2.56241900	-0.98776300
1	-1.87613400	-3.31078200	-2.87747100	6	3.25661100	3.04001900	-1.91794100
1	-4.75132100	0.71364900	-2.32681000	6	3.68297600	4.37210900	-1.88133000
1	-5.28665800	-0.96518400	-4.06541700	1	3.50972100	6.27953000	-0.87625800
6	-0.90456300	-3.38211200	0.02825900	1	1.84742600	5.43916300	0.78645400
6	0.76001400	-2.02929600	-1.68573200	1	1.09017400	3.06860200	0.70918300
6	0.49046900	-3.98857800	-0.18735900	1	3.64183900	2.36310800	-2.68585200
1	-1.60376600	-3.88198600	-0.66212500	1	4.40563000	4.73397800	-2.61744400
6	0.96747200	-3.54878900	-1.57450900	6	1.85841200	1.12887900	-1.04345200
1	1.56438700	-1.53979100	-1.12429700	8	0.58396900	0.91252300	-0.93770100
1	1.17649500	-3.60395900	0.58626100	1	2.37835300	0.58061400	-1.85947900

2TSbtm-RS

E = -4330.96642268 G =

27	-0.71871100	0.32530800	-0.62616600
17	-1.02985300	0.19005400	-2.91213300
6	0.37295200	2.14938600	-0.97260700
14	-0.52421400	3.60109100	-1.73206600
6	-2.38830700	3.33234800	-1.75679500
1	-2.89118700	4.22697100	-2.16030400
1	-2.60942700	2.47823500	-2.41351100
1	-2.81004300	3.12482000	-0.76386900
6	0.09995000	3.72983200	-3.50054300
1	-0.09488500	2.77655600	-4.01479400
1	-0.40727300	4.54266200	-4.04657700
1	1.18482300	3.92052100	-3.51982400
6	-0.06602600	5.16708300	-0.77883700
1	1.02670800	5.30847200	-0.79048000
1	-0.52878300	6.04923300	-1.25240800
1	-0.39063300	5.14083400	0.27210200
6	1.49040500	1.59503600	-1.03028800
6	2.62174300	0.76103400	-1.12021900
1	3.04319500	0.74855000	-2.13421100
6	3.66843200	0.87864400	-0.06273800
6	4.90313700	0.21987800	-0.20798300
6	3.45925100	1.62859300	1.10754000
6	5.87191400	0.28010900	0.79389400
1	5.10269200	-0.35442600	-1.11350200
6	4.42755100	1.69053200	2.11109100
1	2.53063700	2.19004000	1.21350700
6	5.63811000	1.00733800	1.96471000
1	6.81790300	-0.24970600	0.65749500
1	4.24070200	2.29006100	3.00617800
1	6.39780800	1.05337200	2.74865200
6	-3.36819400	-1.88911000	3.93668100
6	-2.42704100	-0.93438800	3.55221200
6	-2.12864900	-0.73546500	2.19473300
6	-2.78903000	-1.50432000	1.21934400
6	-3.73253100	-2.46555300	1.61519200
6	-4.02099500	-2.65769200	2.96624700
1	-3.59014600	-2.03966000	4.99570100
1	-1.91675700	-0.34783600	4.31899500
1	-4.25188400	-3.06590000	0.86580300
1	-4.75811000	-3.40660700	3.26507800
6	-1.18790200	2.00247900	2.61311700
6	0.73428200	0.04858300	2.48529900
6	0.21116300	2.40103300	3.10567600
1	-1.76424300	1.65264100	3.48530000
6	0.89826100	1.11625700	3.58020300
1	1.47691100	0.22060200	1.69729300
1	0.77628200	2.85224000	2.27200900
1	0.14678700	3.16199000	3.90058500
1	1.96525700	1.27239400	3.80368800
1	0.42087300	0.77149600	4.51478600
6	-2.31996500	-2.76212500	-1.41872300
6	-3.98089900	-0.55479600	-1.31281900
6	-3.38913600	-2.62899300	-2.52088000
1	-2.67825500	-3.48561200	-0.66728100
6	-4.56462600	-1.82169200	-1.95941500
1	-3.61676700	0.09986100	-2.11959400
1	-2.94655300	-2.08659600	-3.37133800
1	-3.69957400	-3.62333400	-2.88102200
1	-5.29476300	-1.55817800	-2.74239200
1	-5.11161400	-2.41339500	-1.20321100
15	-0.86274600	0.46242900	1.59443000
15	-2.37434000	-1.11239800	-0.53013800
6	-1.97680600	3.09385200	1.91036000
1	-2.96806800	2.73548900	1.59274900
1	-2.12982500	3.95640900	2.57907100
1	-1.44452900	3.44722600	1.02050400
6	-0.94176600	-3.19527100	-1.90638000
1	-0.21377300	-3.21992800	-1.08477200
1	-1.00546000	-4.19833400	-2.35945400
1	-0.56566300	-2.49007900	-2.65961100
6	0.87993300	-1.39657700	2.94219600
1	1.90186600	-1.56271700	3.32040700
1	0.17704300	-1.65357400	3.74865600
1	0.71171800	-2.07926700	2.09867600
6	-4.91416000	0.23114600	-0.40218800
1	-5.78494100	0.59637300	-0.97025300
1	-5.28704100	-0.38206200	0.43245400
1	-4.40582400	1.10962900	0.02566500
6	5.01518500	-3.73793300	-0.93997000
6	4.30367200	-3.38269200	0.21047600
6	3.23251300	-2.49242600	0.12702500
6	2.85826400	-1.94485500	-1.10680900
6	3.56754000	-2.31523600	-2.25815000
6	4.64161200	-3.20369600	-2.17799500
1	5.85599100	-4.43298100	-0.87387700
1	4.59207500	-3.79705900	1.17990900

1	2.67712500	-2.19998700	1.01667300
1	3.27209900	-1.89649900	-3.22478500
1	5.18632200	-3.48551000	-3.08274200
6	1.74124200	-0.95417400	-1.20211800
8	0.80153500	-1.01225300	-0.30480800
1	1.41041800	-0.81637200	-2.24960200

2TSbtm-SR

E = -4330.96157780 G = -4330.265556

27	0.49879500	0.03325200	0.45470900
17	-0.24175000	-1.10569700	2.32275300
6	-0.82178200	1.64339400	0.97450800
14	-0.78282400	2.40597500	2.68065700
6	0.80017900	2.02240300	3.62385200
1	0.77869500	2.54053100	4.59720400
1	0.85961000	0.93942500	3.80280600
1	1.70813800	2.34042000	3.09305000
6	-2.25898900	1.69861400	3.60272700
1	-2.15126400	0.60565500	3.66736700
1	-2.32027500	2.11352600	4.62263500
1	-3.19742300	1.92736600	3.07404600
6	-0.99055100	4.27196000	2.46602000
1	-1.92073700	4.49040900	1.91740400
1	-1.05008200	4.76576700	3.45048600
1	-0.15577400	4.72731500	1.91132600
6	-1.56639300	1.41640200	0.00219500
6	-2.39058300	1.01056100	-1.06974300
1	-2.24922500	1.62641700	-1.96832800
6	-3.83822100	0.86060800	-0.74521600
6	-4.80779200	1.15606700	-1.71611900
6	-4.26336900	0.35184900	0.49443100
6	-6.16403000	0.94033900	-1.46361900
1	-4.49088500	1.55268200	-2.68492100
6	-5.61663000	0.13205200	0.74383800
1	-3.51916100	0.10758700	1.25348200
6	-6.57513300	0.42291500	-0.23302000
1	-6.90280400	1.17461900	-2.23452000
1	-5.92540400	-0.27895400	1.70816600
1	-7.63515600	0.24718000	-0.03494700
6	5.23171800	-0.09059900	-2.76227700
6	4.09946400	0.67204900	-2.47725000
6	3.19334200	0.25770900	-1.48786300
6	3.43771900	-0.92966000	-0.77424600
6	4.58070200	-1.69042000	-1.06740900
6	5.47175800	-1.27522000	-2.05692700
1	5.92767900	0.23619500	-3.53830200
1	3.92032900	1.59324800	-3.03515200
1	4.78260300	-2.61175800	-0.51799200
1	6.35831300	-1.87416600	-2.27772500
6	2.27763200	2.95286900	-0.83916200
6	0.77604700	1.56032300	-2.66564300
6	1.32839800	3.78011000	-1.72050100
1	3.27515900	2.96710200	-1.30857200
6	1.15040300	3.00660000	-3.02992400
1	-0.27218400	1.55434500	-2.34538700
1	0.35472600	3.88571300	-1.21081800
1	1.72541900	4.79589900	-1.88021600
1	0.38069600	3.45575200	-3.67840600
1	2.09483500	3.02166800	-3.60245300
6	2.04068500	-3.20114900	0.50514400
6	3.20492000	-1.30678500	2.14711100
6	2.49106000	-3.66030200	1.90604400
1	2.79180700	-3.52662900	-0.23356100
6	3.64565700	-2.76353400	2.36495800
1	2.41990100	-1.07482300	2.88381000
1	1.64200900	-3.54349700	2.59843900
1	2.76852000	-4.72682500	1.89445300
1	3.90746100	-2.93718000	3.42178100
1	4.55594700	-2.97395600	1.77502800
15	1.66432300	1.18889000	-1.05664400
15	2.22762900	-1.33766700	0.55182400
6	2.40474400	3.42158400	0.60051800
1	3.09419000	2.78421700	1.17470300
1	2.78920300	4.45355400	0.64116000
1	1.43104100	3.40426500	1.10296500
6	0.66849800	-3.70513700	0.07330100
1	0.33390900	-3.22504600	-0.85625800
1	0.69758400	-4.79731600	-0.07197800
1	-0.07907000	-3.47509400	0.84233300
6	0.93988700	0.51778300	-3.76407800
1	0.27641900	0.75552700	-4.61149900
1	1.97055300	0.48013200	-4.14678300
1	0.67304300	-0.47509300	-3.37664100
6	4.30126800	-0.25311100	2.22262800
1	4.76684700	-0.25614100	3.22131000
1	5.09312300	-0.43030800	1.47862800
1	3.89597100	0.75621900	2.04978900
6	-4.66890900	-3.51857600	-0.50333300
6	-3.60226900	-3.25041300	0.35999800

6	-2.60494000	-2.34783200	-0.01029900
6	-2.65586500	-1.70568600	-1.25435000
6	-3.72482900	-1.98424400	-2.11751200
6	-4.72784100	-2.88040200	-1.74590300
1	-5.45392500	-4.21998000	-0.20915200
1	-3.55203500	-3.73767900	1.33738400
1	-1.79419000	-2.10772000	0.67468500
1	-3.77536600	-1.48234800	-3.08754700
1	-5.55978900	-3.07989100	-2.42602200
6	-1.61733800	-0.69735000	-1.64395900
8	-0.40534600	-0.82061600	-1.18504000
1	-1.69534200	-0.47097700	-2.73130800

2TSbtm-SS

E = -4330.96882576 G = -4330.27219

27	-0.50514000	0.49524400	-0.31382800
17	0.31022000	1.20486000	-2.34680100
6	0.64216600	2.02027100	0.63171200
14	0.29458700	3.81453800	0.25722800
6	-1.26818500	4.02612100	-0.77496800
1	-1.46531300	5.09922400	-0.93581100
1	-1.10509500	3.54647400	-1.75130300
1	-2.16499900	3.58161300	-0.32196500
6	1.75417300	4.43201900	-0.75427200
1	1.79193000	3.87633400	-1.70353400
1	1.65082000	5.50694000	-0.97827200
1	2.70564700	4.27249600	-0.22389900
6	0.19035000	4.77410400	1.88179400
1	1.12585400	4.65137400	2.45100400
1	0.05281200	5.84991300	1.68115000
1	-0.63990900	4.44233200	2.52282900
6	1.58607700	1.23641800	0.87171800
6	2.60165700	0.26815700	0.92688100
1	2.69770100	-0.24733900	1.89125300
6	3.90581600	0.61842800	0.30673700
6	5.10369900	0.13700000	0.85961800
6	3.96406200	1.34240300	-0.89944800
6	6.32813300	0.37848200	0.23375700
1	5.06901400	-0.44576700	1.78333600
6	5.18894900	1.58170500	-1.52237900
1	3.03725800	1.69512500	-1.35650000
6	6.37807000	1.10305200	-0.96012700
1	7.24834700	-0.00745300	0.67990200
1	5.21428000	2.14300200	-2.46004800
1	7.33573700	1.29167900	-1.45134200
6	-4.94520700	-2.56057800	1.56989400
6	-3.91221000	-1.72403400	1.99278900
6	-3.03910300	-1.14386800	1.05933100
6	-3.21832000	-1.40085500	-0.31378200
6	-4.26046500	-2.24384000	-0.72953600
6	-5.11807000	-2.82276200	0.20634500
1	-5.61600600	-3.01223600	2.30436100
1	-3.78589700	-1.52731600	3.05952700
1	-4.41076300	-2.44707400	-1.79164000
1	-5.92685100	-3.47699500	-0.12735700
6	-2.39205200	1.15719800	2.74917200
6	-0.58899700	-0.90031800	2.81296900
6	-1.29709000	1.26847500	3.82034300
1	-3.24024500	0.61040300	3.19506800
6	-0.86345900	-0.16631900	4.14012300
1	0.42526200	-0.66276500	2.46683500
1	-0.44490700	1.84442600	3.42085500
1	-1.66761500	1.80247200	4.71067600
1	0.02318500	-0.19727300	4.79322600
1	-1.67826600	-0.67789300	4.68274900
6	-1.69121500	-1.73910300	-2.83556100
6	-3.14316500	0.58864800	-2.51263400
6	-2.13903400	-1.00288200	-4.11302300
1	-2.38169500	-2.58045200	-2.65651700
6	-3.41523900	-0.21798000	-3.79252800
1	-2.43776800	1.39667500	-2.76375900
1	-1.34212700	-0.30231800	-4.40978200
1	-2.28351500	-1.71681000	-4.94011400
1	-3.70898700	0.44916100	-4.61963500
1	-4.26056600	-0.91064500	-3.62780100
15	-1.64195000	-0.03525900	1.51766000
15	-2.07095700	-0.52604300	-1.45805700
6	-2.90442700	2.46464100	2.17057200
1	-3.68272700	2.29240100	1.41109500
1	-3.34098800	3.09613000	2.96117400
1	-2.09353000	3.02861100	1.69900200
6	-0.26161500	-2.26798600	-2.86486300
1	0.00479000	-2.75456200	-1.91753900
1	-0.15190600	-2.99660600	-3.68492000
1	0.44656200	-1.44422900	-3.02698300
6	-0.72820700	-2.41664000	2.86076400
1	0.03499800	-2.84359000	3.53162800
1	-1.71371800	-2.72977100	3.23555100
1	-0.58543300	-2.84437200	1.85970000

6	-4.36095000	1.17752200	-1.81406100
1	-4.87821700	1.88808700	-2.47868400
1	-5.08252200	0.39904800	-1.52210500
1	-4.07096000	1.72579500	-0.90401800
6	4.47594800	-4.43806800	0.48075400
6	3.28044300	-4.32867400	1.20077200
6	2.41221000	-3.26379100	0.95989200
6	2.72461200	-2.29138500	-0.00207500
6	3.92218100	-2.41202000	-0.72435300
6	4.79248000	-3.47717400	-0.48345200
1	5.15639600	-5.27239600	0.66916900
1	3.02485400	-5.08087300	1.95193200
1	1.47772100	-3.17123100	1.51087300
1	4.17115300	-1.66135800	-1.47696800
1	5.72273600	-3.55566200	-1.05150800
6	1.81686300	-1.12906100	-0.24399100
8	0.55996700	-1.24283200	0.04796300
1	2.07291500	-0.58210600	-1.17014300

2TStop-RR

E = -4330.97931975 G = -4330.281267

27	-0.40508800	0.46547900	0.43962000
17	0.50377900	0.80680200	2.53510400
6	0.61648100	2.26301900	-0.27705000
14	0.05206600	3.94064700	0.27995100
6	-1.39330100	3.78615100	1.47113400
1	-1.72514500	4.78608000	1.79616100
1	-2.25738200	3.26835200	1.03011400
1	-1.07026700	3.21248700	2.35183100
6	-0.43511700	4.94759800	-1.24039700
1	-1.31296700	4.52106000	-1.75057000
1	-0.68584200	5.98204000	-0.95237100
1	0.39229600	4.98685300	-1.96668500
6	1.52701500	4.70362600	1.15891600
1	2.39761300	4.77102200	0.48788700
1	1.29431100	5.71347900	1.53520300
1	1.80454700	4.06463500	2.01188000
6	1.42630300	1.35993900	-0.51925800
6	2.43550100	0.33728500	-0.76508000
1	2.50699200	0.17235000	-1.85014300
6	3.79177600	0.73724700	-0.22937000
6	4.93899900	0.56684700	-1.01444600
6	3.92816400	1.20415800	1.08750400
6	6.20407700	0.85948600	-0.49880000
1	4.83918600	0.18666000	-2.03411200
6	5.19272200	1.49685600	1.60175400
1	3.03652900	1.32517800	1.70782600
6	6.33468400	1.32614000	0.81176500
1	7.09042100	0.71892200	-1.12255700
1	5.28705600	1.85867200	2.62880000
1	7.32331800	1.55537100	1.21726300
6	-4.59219300	-2.61365600	-1.85919700
6	-3.77189100	-1.50897100	-2.09170600
6	-2.88851000	-1.05528400	-1.09984600
6	-2.83649500	-1.72653400	0.13661800
6	-3.67280700	-2.82939200	0.36665100
6	-4.54499800	-3.27402500	-0.62692700
1	-5.27422700	-2.95818300	-2.63999000
1	-3.83072500	-0.99826300	-3.05447800
1	-3.64137500	-3.34801400	1.32691500
1	-5.18736400	-4.13819200	-0.44257900
6	-1.32752200	0.48799700	-3.07765400
6	-3.04796500	1.86482000	-1.43430400
6	-1.89675600	1.81366000	-3.61324500
1	-1.89606800	-0.34098600	-3.52775200
6	-3.24922400	2.06238100	-2.94338200
1	-2.46929700	2.72291900	-1.05899800
1	-1.20180400	2.63152100	-3.35589700
1	-1.96982200	1.79022800	-4.71265300
1	-3.64208800	3.06981700	-3.15979400
1	-3.99888600	1.34334700	-3.31740200
6	-2.68445200	-0.78075100	2.92027100
6	-0.68214800	-2.49941700	2.09737200
6	-1.89996900	-1.51080800	4.02575400
1	-3.62665600	-1.32667700	2.74114100
6	-1.37468700	-2.82113200	3.43378900
1	0.28044600	-2.01151400	2.31339300
1	-1.05266700	-0.87750400	4.33274900
1	-2.53735200	-1.67599600	4.91000000
1	-0.67552400	-3.33147800	4.11593200
1	-2.21334100	-3.51994800	3.26003700
15	-1.82653300	0.44447500	-1.26207500
15	-1.65292500	-1.07135800	1.37882500
6	0.14978700	0.27018700	-3.37482200
1	0.52568200	-0.61817200	-2.85236200
1	0.30145500	0.14869600	-4.45976100
1	0.74223500	1.13353100	-3.04237600
6	-2.99356300	0.68057200	3.22169300
1	-3.57363300	1.14963200	2.41281900

1	-3.58750200	0.76298500	4.14658700
1	-2.06199300	1.24780600	3.35009200
6	-4.32159100	1.73518200	-0.61022200
1	-4.92980900	2.65025700	-0.69361800
1	-4.93462000	0.88617400	-0.94794000
1	-4.09645000	1.57952800	0.45526800
6	-0.44150800	-3.68496000	1.17219800
1	0.24342100	-4.40022200	1.65597500
1	-1.37358100	-4.22185400	0.93790800
1	0.01729500	-3.33951200	0.23727700
6	4.18491600	-4.33389800	-1.74631400
6	2.92237900	-4.03613400	-2.26823400
6	2.17578600	-2.98006200	-1.73980900
6	2.67856000	-2.20642800	-0.68582000
6	3.93936100	-2.52053600	-0.15828400
6	4.68966000	-3.57390300	-0.68652700
1	4.76997800	-5.16059300	-2.15750900
1	2.51542700	-4.63480100	-3.08781800
1	1.17928200	-2.74678600	-2.11841900
1	4.33424300	-1.93422500	0.67434400
1	5.67146200	-3.80565800	-0.26541000
6	1.86980900	-1.03873200	-0.14760300
8	0.53975600	-1.12516200	-0.42825100
1	2.08351900	-0.95028700	0.94000500

2TStop-RS

E = -4330.96377209 G = -4330.267947

27	-0.40677100	0.22181500	0.37163900
17	0.77516300	-0.20706400	2.30419400
6	0.52669000	2.13812300	0.23863400
14	0.16834200	3.43965000	1.52208000
6	-1.14066000	2.87880700	2.75204900
1	-1.30116700	3.66947400	3.50385300
1	-2.11312200	2.65338300	2.29153700
1	-0.78699200	1.97274000	3.26433200
6	-0.40088400	5.00478500	0.62940800
1	-1.35777300	4.85376000	0.10513800
1	-0.53977400	5.83005000	1.34756800
1	0.34624900	5.32133000	-0.11572000
6	1.78384100	3.72713600	2.43685300
1	2.58698400	4.01701700	1.74160700
1	1.67929300	4.51192200	3.20426900
1	2.08503700	2.78974500	2.92983500
6	1.42488200	1.73175600	-0.52459300
6	2.40974400	1.11720300	-1.32274700
1	2.44119400	1.52716800	-2.34065000
6	3.77397500	1.03727900	-0.71954600
6	3.95935500	0.84946400	0.66088700
6	4.90564500	1.08884500	-1.54833500
6	5.24318900	0.72217300	1.19020300
1	3.08850700	0.78023900	1.31533500
6	6.18981700	0.95843400	-1.01691600
1	4.77470000	1.22828700	-2.62509700
6	6.36518500	0.77479100	0.35700600
1	5.36689500	0.57037900	2.26557100
1	7.05766300	0.99913300	-1.68031900
1	7.36931600	0.67118000	0.77542400
6	-5.11804200	-1.71622500	-2.19513800
6	-4.25286800	-0.62298700	-2.13837400
6	-3.19731600	-0.59675000	-1.21356400
6	-3.02265500	-1.68199800	-0.33374800
6	-3.90185900	-2.77419000	-0.39260700
6	-4.94211100	-2.79469200	-1.32155100
1	-5.93479000	-1.72609900	-2.92060000
1	-4.41247000	0.21545900	-2.81892100
1	-3.77169000	-3.61806600	0.28775500
1	-5.61699200	-3.65256400	-1.36631900
6	-1.83576600	1.55556800	-2.71421000
6	-3.12314600	2.23515400	-0.38650700
6	-2.25176500	3.02694200	-2.55307500
1	-2.60982300	1.04980900	-3.31296200
6	-3.47163500	3.06512500	-1.63054800
1	-2.41941500	2.82380900	0.21866900
1	-1.42005500	3.59127100	-2.09723800
1	-2.45273200	3.48383800	-3.53562100
1	-3.75225500	4.09384700	-1.35004700
1	-4.34580200	2.62464600	-2.14188200
6	-2.38648000	-1.91787400	2.52643100
6	-0.65066800	-3.14989100	0.76999200
6	-1.51910400	-3.06183500	3.08491000
1	-3.38723000	-2.32084700	2.29571700
6	-1.19005400	-4.00813000	1.92588900
1	0.35461400	-2.80289200	1.04558500
1	-0.58667600	-2.63263400	3.48574000
1	-2.03749700	-3.57348100	3.91232500
1	-0.45114000	-4.77325900	2.21465500
1	-2.09863900	-4.55015200	1.60654500
15	-2.03939100	0.82314900	-0.99594500
15	-1.62153800	-1.55624900	0.85110600

6	-0.49243600	1.33885400	-3.39276800
1	-0.23237400	0.27392800	-3.42232100
1	-0.52756900	1.73054700	-4.42260600
1	0.30389700	1.86653800	-2.85407400
6	-2.52946300	-0.71347200	3.44933300
1	-3.16422700	0.06763700	3.00366600
1	-2.99442500	-1.01659700	4.40163700
1	-1.54519000	-0.27481700	3.66019600
6	-4.29894800	1.82646500	0.49022200
1	-4.80884300	2.71777300	0.89003500
1	-5.03635300	1.23536300	-0.07320900
1	-3.97001000	1.21801200	1.34618200
6	-0.57384200	-3.80301100	-0.60247500
1	0.12733700	-4.65235000	-0.57300700
1	-1.55275200	-4.18102900	-0.93599500
1	-0.20225600	-3.07528300	-1.33721900
6	4.73928100	-3.40986600	-0.50465000
6	3.78364800	-2.97734400	0.42103700
6	2.78777000	-2.07906300	0.03926200
6	2.72967100	-1.60064400	-1.27723000
6	3.69537600	-2.03200900	-2.19610300
6	4.69368600	-2.93186100	-1.81684800
1	5.52052000	-4.11210100	-0.20275100
1	3.82331800	-3.33140300	1.45455000
1	2.07126900	-1.70733300	0.77014900
1	3.66036500	-1.65746900	-3.22340000
1	5.43832800	-3.26036000	-2.54646800
6	1.66456200	-0.63741100	-1.71235400
8	0.46644800	-0.74835000	-1.21446500
1	1.70547000	-0.51615300	-2.81716900

2TStop-SR

E = -4330.96476211 G = -4330.268648

27	0.67458000	0.20379800	-0.69620300
17	0.70801300	-0.25875600	-2.95988300
6	-0.42148700	2.00104000	-1.14304400
14	0.44613500	3.42808000	-1.96553900
6	2.26995100	3.05112500	-2.23846800
1	2.75516500	3.90078700	-2.74700300
1	2.82627600	2.85306700	-1.31111500
1	2.35368100	2.16261100	-2.88079600
6	0.22720600	4.96949500	-0.89555400
1	0.70458400	4.86285100	0.09059500
1	0.67133500	5.84929000	-1.39030300
1	-0.84289700	5.17374800	-0.73100100
6	-0.38080100	3.65132500	-3.63866000
1	-1.44933000	3.89163200	-3.52087600
1	0.09627700	4.45355000	-4.22552400
1	-0.30078200	2.70642600	-4.19894400
6	-1.52734000	1.42313600	-1.13963200
6	-2.65878000	0.58371600	-1.11311100
1	-3.04935500	0.40063100	-2.12351900
6	-3.74420100	0.91926400	-0.14034300
6	-4.99222400	0.27641500	-0.22609000
6	-3.56251700	1.86685300	0.88078400
6	-6.00395300	0.54460300	0.69554600
1	-5.16806200	-0.45143400	-1.01903700
6	-4.57314200	2.13645000	1.80488400
1	-2.61634600	2.40581600	0.93924200
6	-5.79882100	1.47040300	1.72290600
1	-6.96023800	0.02257900	0.61038600
1	-4.40303300	2.87839200	2.58964500
1	-6.59128200	1.67909500	2.44570500
6	3.74752800	-1.40288600	3.84279800
6	2.90546300	-0.39464000	3.37376400
6	2.40821300	-0.43606800	2.06131300
6	2.77316200	-1.50003000	1.21570700
6	3.62087400	-2.51062500	1.69563600
6	4.10204900	-2.46562300	3.00393200
1	4.12905600	-1.36148400	4.86563000
1	2.63734900	0.42869000	4.03919000
1	3.90724700	-3.33937900	1.04502800
1	4.75627800	-3.25934100	3.37201500
6	0.07516600	1.18482500	2.74575500
6	2.09678500	2.50577400	1.46897400
6	-0.06384000	2.71257300	2.67965700
1	0.65761800	0.96574800	3.65663600
6	1.36608600	3.26021500	2.59603400
1	1.80619300	2.96768700	0.51743800
1	-0.63634100	3.00493200	1.78323900
1	-0.60865300	3.09676500	3.55707800
1	1.39057000	4.34750100	2.41734100
1	1.88134600	3.08253900	3.55641200
6	3.65863100	-1.62407100	-1.56106600
6	1.40347800	-3.09462100	-0.94924700
6	3.30511600	-2.78282800	-2.51134600
1	4.43345900	-1.97625900	-0.85939900
6	2.52850100	-3.82731300	-1.70104700
1	0.63652500	-2.80059600	-1.68218400

1	2.66643100	-2.39314500	-3.31963300
1	4.21611300	-3.20154500	-2.96975600
1	2.11134900	-4.62013200	-2.34315200
1	3.20151900	-4.32601400	-0.97988100
15	1.26292500	0.82012300	1.34847700
15	2.12128800	-1.42995000	-0.50347900
6	-1.19486700	0.35109900	2.78074200
1	-0.95431700	-0.70552300	2.96725900
1	-1.86120100	0.70415100	3.58451700
1	-1.73421000	0.39131400	1.83179600
6	4.14010100	-0.34628800	-2.23692800
1	4.35930500	0.44237400	-1.49964000
1	5.06442100	-0.53883000	-2.80583000
1	3.37237300	0.03157500	-2.92492100
6	3.61763700	2.49516600	1.55014000
1	4.01091000	3.52384100	1.50512400
1	3.97431300	2.03661800	2.48443300
1	4.06078600	1.93043500	0.71479700
6	0.73392400	-3.84809700	0.19196900
1	0.26055700	-4.76681600	-0.19093800
1	1.45337300	-4.14093200	0.97248000
1	-0.04851300	-3.22195400	0.64337600
6	-5.06352700	-3.73830500	0.21571700
6	-4.30202400	-3.12713800	1.21738800
6	-3.24056300	-2.28736300	0.87913300
6	-2.92901800	-2.04311500	-0.46467900
6	-3.68744100	-2.66938200	-1.46330400
6	-4.75030400	-3.51106000	-1.12838400
1	-5.89617800	-4.39444000	0.48148500
1	-4.54275900	-3.30303700	2.26895100
1	-2.64111800	-1.80200100	1.64858900
1	-3.44041100	-2.48792800	-2.51366800
1	-5.33396700	-3.99443400	-1.91608300
6	-1.81761400	-1.11213600	-0.83956900
8	-0.83028100	-0.98993100	0.00407500
1	-1.53658500	-1.23070000	-1.90355200

2TStop-SS

E = -4330.96291048 G = -4330.267525

27	0.50746700	-0.10591600	0.59680100
17	0.05731800	0.89368600	2.61739100
6	-0.78327100	-1.75408700	1.11002100
14	-0.27001400	-2.95686900	2.43582300
6	1.43334700	-2.52586300	3.11093600
1	1.71293300	-3.23878100	3.90433600
1	2.22653500	-2.54265700	2.34964400
1	1.39815800	-1.51502700	3.54260500
6	-0.28132100	-4.70266600	1.71252500
1	0.47286000	-4.83273600	0.92105900
1	-0.07070600	-5.44469400	2.50063400
1	-1.26784100	-4.93572000	1.28063200
6	-1.53218000	-2.80579200	3.82043100
1	-2.54253500	-3.05706800	3.46052300
1	-1.28887300	-3.46497200	4.67007300
1	-1.54517500	-1.76272000	4.17422900
6	-1.80871500	-1.12232500	0.77894400
6	-2.83527400	-0.23541900	0.42642300
1	-3.04799500	0.45069300	1.25588400
6	-4.05521000	-0.74328700	-0.25130600
6	-5.21765600	0.04930200	-0.26891100
6	-4.08401100	-1.97640500	-0.92753900
6	-6.35924400	-0.36781500	-0.95497100
1	-5.21962000	1.00308200	0.26204200
6	-5.22557800	-2.39256400	-1.61362800
1	-3.20307800	-2.62111600	-0.89748100
6	-6.36948700	-1.58866200	-1.63622600
1	-7.25004800	0.26572200	-0.95375700
1	-5.22444800	-3.35688000	-2.12870600
1	-7.26409000	-1.91555600	-2.17153600
6	4.44240100	0.27020200	-3.51429500
6	3.42954000	-0.54938200	-3.01496200
6	2.74432500	-0.19880600	-1.84139100
6	3.09222700	0.98577000	-1.16434400
6	4.11655400	1.80083700	-1.66982100
6	4.78634300	1.44781700	-2.84138600
1	4.96828300	-0.01131300	-4.42953800
1	3.18066500	-1.47096200	-3.54491800
1	4.39031800	2.72054900	-1.14893400
1	5.57742700	2.09159000	-3.23277400
6	0.51208800	-2.03315300	-2.47240200
6	2.30398900	-2.82593600	-0.54769500
6	0.54997400	-3.53308900	-2.13844800
1	1.15845500	-1.86946400	-3.35003500
6	1.96196500	-3.85182600	-1.63974800
1	1.75412700	-3.11023600	0.35919800
1	-0.18691600	-3.74941000	-1.34602200
1	0.26899000	-4.13526000	-3.01755400
1	2.04728700	-4.88039800	-1.25194800
1	2.68165200	-3.76457900	-2.47299000

6	3.44072100	1.72163200	1.66404200
6	1.52501200	3.11851100	0.24887300
6	3.03135600	3.10142700	2.21366200
1	4.38219400	1.83499600	1.10011100
6	2.55249700	3.95198000	1.03242200
1	0.60435900	3.05781000	0.84528100
1	2.20264600	2.96326100	2.92657800
1	3.86997100	3.56808600	2.75596200
1	2.10470200	4.90306000	1.36389100
1	3.40586900	4.21416600	0.38076500
15	1.43744600	-1.23424000	-1.05297900
15	2.13985000	1.35876400	0.36433100
6	-0.86398200	-1.46091400	-2.76968900
1	-0.80771200	-0.38677200	-2.98655000
1	-1.30920900	-1.97770300	-3.63546600
1	-1.53595300	-1.58878300	-1.91452600
6	3.60955200	0.63378400	2.71793900
1	3.90744700	-0.32567600	2.26706500
1	4.39075800	0.91919600	3.44125400
1	2.66608400	0.47974600	3.25854100
6	3.77986900	-2.68990800	-0.19825600
1	4.17173600	-3.64346600	0.19105400
1	4.38020600	-2.40737100	-1.07588100
1	3.94121700	-1.92212600	0.57422500
6	1.16435900	3.60112300	-1.14842800
1	0.69934800	4.59838700	-1.09005700
1	2.04706000	3.67543300	-1.80247600
1	0.43851100	2.91198800	-1.60181200
6	-3.81001800	4.77223100	0.18339200
6	-2.79350000	4.25023900	0.99438000
6	-2.19444400	3.03202900	0.67715700
6	-2.60793800	2.31341000	-0.45690300
6	-3.63001900	2.83683800	-1.25925900
6	-4.22831300	4.06117700	-0.94342000
1	-4.27563100	5.72900600	0.43322900
1	-2.47095700	4.79680700	1.88458800
1	-1.42565900	2.60390500	1.32352700
1	-3.95623600	2.27783100	-2.13997100
1	-5.02145700	4.46047100	-1.58088400
6	-1.95333800	1.01053000	-0.82426300
8	-0.66615800	0.92683000	-0.73252000
1	-2.41736700	0.58073400	-1.73717400

E = -1102.42600708 G = -1102.119515

6	2.49451000	-0.23851800	0.24740800
14	4.32825800	-0.28186800	0.02908200
6	4.77553600	-1.86242300	-0.89390200
1	5.86501500	-1.92490600	-1.05184500
1	4.46326000	-2.75422700	-0.32707200
1	4.28633200	-1.89699200	-1.88053700
6	5.13673600	-0.25894100	1.72905800
1	4.82527800	-1.13094300	2.32586900
1	6.23536100	-0.28131900	1.63753800
1	4.85777100	0.65010200	2.28522200
6	4.83775400	1.23170800	-0.96907000
1	4.54914200	2.15975700	-0.45038200
1	5.92980000	1.24928400	-1.12057800
1	4.35537200	1.23148100	-1.95956100
6	1.28542300	-0.17397500	0.40575300
6	-0.17227700	-0.16331500	0.51766800
1	-0.44788200	-0.57513400	1.50302400
6	-0.77521900	1.22256600	0.37812000
6	-1.89408400	1.57775700	1.14221900
6	-0.28543500	2.12995300	-0.57044000
6	-2.51379400	2.81577900	0.96266100
1	-2.29258600	0.86929800	1.87207400
6	-0.90643000	3.36743400	-0.75516500
1	0.59455300	1.86466300	-1.16109200
6	-2.02332100	3.71379500	0.01079400
1	-3.38641200	3.07751300	1.56578300
1	-0.51369500	4.06615200	-1.49797900
1	-2.50798200	4.68267200	-0.13161500
6	-5.02291600	-1.49968000	0.03565700
6	-4.15195500	-2.35947900	0.71195400
6	-2.77380200	-2.26998200	0.50349600
6	-2.25240500	-1.31893800	-0.38326100
6	-3.13042500	-0.46702500	-1.06476600
6	-4.50826300	-0.55436600	-0.85584400
1	-6.10116400	-1.57045200	0.19871000
1	-4.54913100	-3.10743200	1.40286100
1	-2.08596000	-2.94522300	1.01475600
1	-2.72998500	0.27913600	-1.75492100
1	-5.18198300	0.11870100	-1.39150700
6	-0.75881600	-1.17094600	-0.54597800
8	-0.14854800	-2.42865900	-0.40232700
1	-0.54623700	-0.73226200	-1.54054500
1	0.80901400	-2.28286600	-0.36491800

3a

3INT2

E = -4218.39669277 G = -4217.688396

27	0.41581100	-0.47722000	0.61235900
17	-1.12521600	-0.94431300	2.24655300
6	-0.46932100	-1.73149500	-0.67408600
14	-0.01929900	-3.56403900	-0.63044400
6	1.85103600	-3.77231400	-0.64280800
1	2.13957100	-4.78635900	-0.32252300
1	2.25970800	-3.61138000	-1.65330600
1	2.29516800	-3.03509300	0.04167100
6	-0.75225100	-4.34477100	-2.18831600
1	-0.30192200	-3.91982900	-3.10024200
1	-0.57363600	-5.43301600	-2.19906400
1	-1.83941800	-4.17358600	-2.23665500
6	-0.81523900	-4.33697300	0.88553600
1	-1.91163900	-4.29567400	0.78607300
1	-0.51435700	-5.39222500	0.99483000
1	-0.54594800	-3.79029500	1.79797500
6	-1.52161500	-1.29971300	-1.27165500
6	-2.62464100	-0.85950400	-1.88340000
1	-2.55952200	-0.57422800	-2.94045400
6	-3.92015000	-0.66937400	-1.23638900
6	-4.99887100	-0.13545400	-1.96994700
6	-4.11506500	-0.95147200	0.13411900
6	-6.22820600	0.11337400	-1.35904600
1	-4.86165100	0.08811200	-3.03163300
6	-5.34328000	-0.69748600	0.73938100
1	-3.28505800	-1.34716200	0.72361300
6	-6.40698700	-0.16400200	-0.00017500
1	-7.05171600	0.52788600	-1.94619000
1	-5.47108100	-0.91627300	1.80256400
1	-7.36846700	0.03294900	0.47999100
6	2.42088300	4.42231900	-1.85219700
6	2.44799300	3.03021300	-1.92567800
6	1.64072400	2.24604200	-1.08354800
6	0.79224900	2.88739800	-0.15738200
6	0.76490500	4.28988700	-0.09905800
6	1.57495600	5.05528500	-0.93659700
1	3.06337100	5.01375200	-2.50845900
1	3.11946100	2.55397900	-2.64164000
1	0.09735400	4.79446900	0.60182000
1	1.54395900	6.14575100	-0.87875800
6	1.69481500	0.03806500	-3.00112600
6	3.50989100	-0.06139000	-0.94302400
6	2.90777200	-0.88404000	-3.19790100
1	1.95081800	1.01666200	-3.43731100
6	4.05268900	-0.32077500	-2.35541600
1	3.41474600	-1.00776900	-0.39164100
1	2.65421000	-1.90122900	-2.85651500
1	3.16521300	-0.95672900	-4.26721500
1	4.91436000	-1.00676600	-2.31562400
1	4.41763900	0.61969000	-2.80570700
6	-0.72194100	2.71662000	2.42848800
6	-1.99837900	1.97440100	0.10024300
6	-2.26018400	2.65896200	2.46051000
1	-0.42034800	3.76140300	2.24708900
6	-2.76334100	2.91791500	1.04145000
1	-2.42001000	0.96751300	0.21768100
1	-2.56972400	1.65326800	2.78811400
1	-2.65576600	3.38612000	3.18838200
1	-3.84822100	2.74973200	0.94631200
1	-2.56946900	3.96811600	0.75726700
15	1.70202900	0.39304200	-1.14243900
15	-0.28273700	1.79614000	0.85277500
6	0.41724600	-0.44576100	-3.67067200
1	-0.42596000	0.23159900	-3.47672000
1	0.57303900	-0.48397300	-4.76136500
1	0.12964900	-1.44619700	-3.32730500
6	-0.04976400	2.21808200	3.70035900
1	1.04575600	2.28021500	3.63684300
1	-0.37510700	2.83008500	4.55752800
1	-0.32608000	1.17287300	3.89250000
6	4.32277200	0.89257600	-0.07901200
1	5.32558400	0.47198200	0.09926600
1	4.44951200	1.87905700	-0.54948800
1	3.83854700	1.03117600	0.89724100
6	-2.02898600	2.36778100	-1.36877600
1	-3.06096200	2.30367200	-1.74762200
1	-1.66453600	3.39545000	-1.52016600
1	-1.41308100	1.69423600	-1.98058600
8	2.07479300	-1.10291500	1.28008800
6	2.54357500	-1.51937800	2.54119300
6	1.80436400	-2.77419600	3.03545300
6	4.03995300	-1.85046800	2.38149800
6	2.37131100	-0.39094200	3.56615800
1	0.73599000	-2.55802200	3.16193300
1	1.91410500	-3.59450600	2.31104800
1	2.21102000	-3.11561800	4.00137400
1	4.60166700	-0.95999700	2.06329200
1	4.47610200	-2.21331400	3.32630500

1	4.17412000	-2.63297900	1.61813300
1	2.81549700	-0.65237200	4.54033000
1	2.85469900	0.52979900	3.20080700
1	1.30356800	-0.19101500	3.71252900

4INT1+radical

E = -4218.39247387 G = -4217.687813

27	-0.65655500	0.30513800	0.89234000
17	1.14422000	1.22514000	1.99702900
6	1.04051600	2.88202200	-1.30752600
14	-0.18157700	4.16789900	-0.78049500
6	-1.93093800	3.48887500	-0.89962800
1	-2.64552400	4.28610300	-0.63250100
1	-2.15731700	3.17238800	-1.93057900
1	-2.09917300	2.64361100	-0.21182100
6	0.00533600	5.60949400	-1.98786000
1	-0.22000000	5.29555000	-3.01972800
1	-0.68752000	6.42499900	-1.71996500
1	1.03063400	6.01183900	-1.96975800
6	0.25305100	4.70407500	0.96433200
1	1.21387400	5.24341300	0.97112400
1	-0.52215300	5.37035200	1.37661000
1	0.35301400	3.82358000	1.61531100
6	1.96749200	2.15720300	-1.68853700
6	3.01275600	1.35918700	-2.12034900
1	3.02888900	1.06188200	-3.17482800
6	4.08763500	0.89765200	-1.28536000
6	5.11606100	0.09021700	-1.83371700
6	4.13716500	1.19631500	0.10079900
6	6.14189600	-0.40154600	-1.03281500
1	5.09350100	-0.14569900	-2.90110200
6	5.16216200	0.69437300	0.89424200
1	3.33709600	1.78841900	0.54736100
6	6.17081100	-0.10528100	0.33701100
1	6.92551700	-1.02235300	-1.47416600
1	5.17102200	0.92299500	1.96262100
1	6.97404800	-0.49594700	0.96608700
6	-1.91725800	-4.83321500	-1.76675700
6	-2.14804000	-3.46030200	-1.85346500
6	-1.45230500	-2.55248500	-1.03749300
6	-0.49370400	-3.05263500	-0.12295600
6	-0.26606300	-4.43563800	-0.04918200
6	-0.97463100	-5.32327800	-0.85885400
1	-2.47464700	-5.52071400	-2.40718400
1	-2.88883200	-3.09314200	-2.56553500
1	0.48131500	-4.82892200	0.64248400
1	-0.78577900	-6.39693500	-0.78660800
6	-1.85290700	-0.33710000	-2.94970400
6	-3.64877600	-0.56309100	-0.87245100
6	-3.24876700	0.28264900	-3.15565800
1	-1.82112500	-1.31018800	-3.46635100
6	-4.24756300	-0.47973200	-2.28297900
1	-3.69798200	0.42337500	-0.38437000
1	-3.22249200	1.33977800	-2.84158900
1	-3.52259400	0.27074100	-4.22338300
1	-5.23367400	0.01325500	-2.26062700
1	-4.41193200	-1.49257500	-2.69192300
6	1.21483800	-2.64108100	2.34564100
6	2.09501700	-1.67766900	-0.09159800
6	2.72517800	-2.36825300	2.19185800
1	1.03863900	-3.72483800	2.24895200
6	3.08778300	-2.52783800	0.71559900
1	2.35853400	-0.62074200	0.04771300
1	2.93040100	-1.33143500	2.50582700
1	3.30788400	-3.03678400	2.84635200
1	4.12041500	-2.20806700	0.50564700
1	3.00230800	-3.58790500	0.41405600
15	-1.79429600	-0.73149400	-1.10299200
15	0.46861100	-1.81794700	0.83851200
6	-0.70627000	0.53434200	-3.44794600
1	0.26889700	0.04187700	-3.32086300
1	-0.83246200	0.75640900	-4.52052100
1	-0.65677700	1.48687600	-2.90343300
6	0.62025800	-2.14075100	3.65977800
1	-0.45740300	-2.35352400	3.72824400
1	1.11605200	-2.62994200	4.51414400
1	0.76290300	-1.05381500	3.75158900
6	-4.28286200	-1.57549700	0.07113500
1	-5.34070900	-1.31922200	0.24469600
1	-4.24399700	-2.60054500	-0.32772400
1	-3.77426300	-1.56585700	1.04598000
6	2.03247000	-1.99197400	-1.57811400
1	3.02060000	-1.82347200	-2.03238500
1	1.74042300	-3.03704600	-1.76318400
1	1.31366600	-1.34196100	-2.09727300
8	-2.33310800	1.04162600	1.27546500
6	-2.80471000	1.35614900	2.56660700
6	-2.20902800	2.69821900	3.02148000
6	-4.33568700	1.46104300	2.48879900
6	-2.40214200	0.25165900	3.55867500

1	-1.11367000	2.62251000	3.07560800
1	-2.46404400	3.48691300	2.29728500
1	-2.59105100	2.99797600	4.01119600
1	-4.76920500	0.50139000	2.16769900
1	-4.77735200	1.73321400	3.46129800
1	-4.61854500	2.22777300	1.75076700
1	-2.77992000	0.45012800	4.57473900
1	-2.79978400	-0.72215400	3.22795000
1	-1.30489200	0.18172800	3.61573700

3TS1

E = -4218.39042701 G = -4217.685184

27	0.48565200	-0.40361500	0.85662600
17	-1.18412200	-1.36500400	2.10903500
6	-0.55503600	-2.49166200	-1.16600200
14	0.61313500	-3.88902300	-0.79707800
6	2.39013700	-3.28969600	-0.80964000
1	3.06668400	-4.12208600	-0.55359300
1	2.67086600	-2.92417400	-1.81044400
1	2.52506800	-2.48007700	-0.07357000
6	0.35663100	-5.13341500	-2.19694500
1	0.61167500	-4.69068900	-3.17313100
1	0.99971800	-6.01682500	-2.04462200
1	-0.69015000	-5.47365500	-2.23817900
6	0.10452500	-4.63583800	0.84703700
1	-0.86235300	-5.15364400	0.74193900
1	0.85255100	-5.36470400	1.19921300
1	-0.01730600	-3.84995300	1.60489300
6	-1.56588700	-1.91643700	-1.59756000
6	-2.69010700	-1.29541700	-2.10430300
1	-2.65789500	-0.95397400	-3.14494600
6	-3.90077600	-1.05797700	-1.36662500
6	-4.98644900	-0.38955600	-1.98557700
6	-4.02750400	-1.42905900	-0.00327400
6	-6.14162100	-0.09157200	-1.26990300
1	-4.90343000	-0.10213200	-3.03715700
6	-5.18310300	-1.12064100	0.70503600
1	-3.19061700	-1.91775600	0.49873200
6	-6.24577300	-0.45110500	0.08094000
1	-6.96787700	0.42667600	-1.76268100
1	-5.25354900	-1.39923400	1.75913500
1	-7.15116000	-0.21164400	0.64354400
6	1.75500200	4.86801700	-1.61566200
6	1.99462500	3.50273300	-1.76811700
6	1.31063600	2.54834400	-0.99555100
6	0.35616700	2.99832800	-0.05193800
6	0.11855200	4.37497800	0.08818800
6	0.81344700	5.30703900	-0.68155200
1	2.30561300	5.58881800	-2.22460900
1	2.73755800	3.17917300	-2.49839700
1	-0.62678600	4.72938900	0.80218300
1	0.61581000	6.37422000	-0.55648600
6	1.83360500	0.48628900	-3.02644200
6	3.53627800	0.61478700	-0.86400100
6	3.24503600	-0.09879600	-3.21475000
1	1.81688000	1.49849700	-3.46073300
6	4.19191400	0.61912800	-2.25241000
1	3.61303700	-0.38591500	-0.41271600
1	3.22419100	-1.17533900	-2.97534500
1	3.56323900	-0.01102900	-4.26669100
1	5.18425700	0.13967700	-2.21670000
1	4.35751400	1.65682300	-2.59232700
6	-1.34205600	2.49958300	2.40128200
6	-2.22879600	1.61512000	-0.05767300
6	-2.84473300	2.18512800	2.26201600
1	-1.20424300	3.58938900	2.31390800
6	-3.23445500	2.39830300	0.79947900
1	-2.47886800	0.55003800	0.02526000
1	-3.01267200	1.13054200	2.53696800
1	-3.43506900	2.80853300	2.95321500
1	-4.26082500	2.05720800	0.58846900
1	-3.18520900	3.47283300	0.54557600
15	1.68085700	0.73299700	-1.15454900
15	-0.59553400	1.72415200	0.86772500
6	0.72901300	-0.34139000	-3.67144500
1	-0.26154200	0.11328700	-3.52098400
1	0.90088600	-0.42108900	-4.75770600
1	0.68824600	-1.35529800	-3.25161100
6	-0.71997500	2.01240700	3.70610400
1	0.34868000	2.26567100	3.76804600
1	-1.22844100	2.47871300	4.56585000
1	-0.82149100	0.92077600	3.79250600
6	4.10044800	1.61393400	0.13669600
1	5.15843100	1.38385600	0.34299200
1	4.04593800	2.64893100	-0.23276900
1	3.55325200	1.55414400	1.08733300
6	-2.17881200	2.01058300	-1.52538600
1	-3.16046700	1.83392400	-1.99115500
1	-1.92257400	3.07361900	-1.65110900

1	-1.43592200	1.41741400	-2.07836000
8	2.22426100	-0.91607600	1.34331600
6	2.74071300	-1.21676500	2.61869400
6	2.18366600	-2.55844800	3.12219300
6	4.27007700	-1.31411200	2.48554600
6	2.37122100	-0.10443400	3.61285700
1	1.08967200	-2.50275700	3.21152100
1	2.43226500	-3.36075000	2.41118900
1	2.60394700	-2.82651200	4.10554100
1	4.68973000	-0.35459600	2.14890500
1	4.74520800	-1.58294100	3.44301900
1	4.53151000	-2.08220700	1.74101800
1	2.80798900	-0.27995500	4.60946200
1	2.72940300	0.87132400	3.24598300
1	1.27861000	-0.05445900	3.72415600

4CzIPN-

E = -2483.10304579 G = -2482.447486

6	-0.17588900	1.15999500	0.39577700
6	0.51353000	0.00055700	-0.00049300
6	-0.17411400	-1.15985100	-0.39701000
6	-1.63230100	-1.14134300	-0.45701500
6	-2.31555500	-0.00154700	-0.00061400
6	-1.63402300	1.13926800	0.45568000
6	-2.34497800	2.22224800	1.02706500
7	-2.92165800	3.12629300	1.48769300
6	-2.34160000	-2.22534500	-1.02846700
7	-2.91699600	-3.13029300	-1.48894400
7	-3.73324900	-0.00250500	-0.00058600
6	-4.54540900	0.81203500	-0.78838600
6	-4.54430200	-0.81810100	0.78724800
6	-4.18254200	1.76913700	-1.73954900
6	-5.90720000	0.51613600	-0.50614900
6	-5.90649100	-0.52402700	0.50503800
6	-4.18012700	-1.77463400	1.73848800
6	-5.20658700	2.44019200	-2.40666300
1	-3.13346900	1.98318400	-1.94389700
6	-6.91670500	1.20517700	-1.19096600
6	-6.91505600	-1.21439200	1.18989600
6	-5.20325700	-2.44709300	2.40558700
1	-3.13074000	-1.98709200	1.94291200
6	-6.56113000	2.16572100	-2.13730500
1	-4.94936400	3.19783200	-3.15110500
1	-7.96815600	0.98911300	-0.98517900
6	-6.55817400	-2.17446100	2.13622800
1	-7.96679800	-0.99972000	0.98413900
1	-4.94500400	-3.20434500	3.15006700
7	0.51462600	-2.34244100	-0.75354000
6	1.47757700	-2.47078400	-1.75599900
6	0.46068200	-3.54302900	-0.04214300
6	1.86135600	-1.55162500	-2.73558100
6	2.07565800	-3.75842900	-1.66915300
6	1.41987000	-4.44700600	-0.57431100
6	-0.33914300	-3.88358100	1.05276800
6	2.87988800	-1.92108800	-3.61252500
1	1.38382800	-0.57599900	-2.80629600
6	3.09374500	-4.10831900	-2.56559000
6	1.57315600	-5.71111200	0.00860000
6	-0.16688300	-5.14810100	1.61546400
1	-1.07502500	-3.18273000	1.44640200
6	3.49721300	-3.18401700	-3.52894000
1	3.20577500	-1.20967300	-4.37521300
1	3.56498500	-5.09280300	-2.50667000
6	0.77897600	-6.05620300	1.10235200
1	2.31135700	-6.41381100	-0.38639400
1	-0.78483000	-5.43955900	2.46852400
7	1.93413800	0.00152300	0.00014300
6	2.74855100	0.82602400	-0.77776800
6	2.74866600	-0.82310100	0.77779000
6	2.39127400	1.77899500	-1.73414400
6	4.10976600	0.52997000	-0.49620200
6	4.10984400	-0.52747800	0.49565800
6	2.39147400	-1.77597800	1.73428400
6	3.41578100	2.46359100	-2.38691700
1	1.34756300	1.98525400	-1.96146600
6	5.12101200	1.22718600	-1.16877900
6	5.12112800	-1.22518400	1.16770000
6	3.41597900	-2.46118300	2.38639300
1	1.34780900	-1.98183900	1.96214100
6	4.76905700	2.19731300	-2.10701100
1	3.15487700	3.22593900	-3.12478900
1	6.17141800	1.00947400	-0.95984600
6	4.76921800	-2.19542300	2.10584000
1	6.17152800	-1.00787400	0.95832200
1	3.15505200	-3.22368200	3.12409300
7	0.51112400	2.34342500	0.75274600
6	0.45509800	3.54433300	0.04209700
6	1.47256500	2.47339800	1.75641000
6	-0.34404600	3.88356700	-1.05374100

6	1.41132500	4.45036700	0.57610500
6	2.06747200	3.76264700	1.67128000
6	1.85755800	1.55446200	2.73570700
6	-0.17416900	5.14885700	-1.61542000
1	-1.07761800	3.18111800	-1.44884900
6	1.56215200	5.71526300	-0.00575500
6	3.08366600	4.11437800	2.56914100
6	2.87419700	1.92576900	3.61405700
1	1.38244400	0.57758800	2.80516800
6	0.76861300	6.05906600	-1.10037600
1	-0.79162900	5.43927700	-2.46918700
1	2.29797900	6.41960000	0.39074800
6	3.48840000	3.19033200	3.53221800
1	3.55247300	5.10010300	2.51154700
1	3.20103600	1.21450800	4.37648800
1	0.88771200	-7.04014100	1.56515900
1	-7.33513000	-2.72201100	2.67528600
1	-7.33882900	2.71223400	-2.67634400
1	0.87546400	7.04357900	-1.56239400
1	4.28669300	3.44827700	4.23247400
1	5.54848700	2.75398500	-2.63298500
1	5.54867900	-2.75258600	2.63124800
1	4.29693400	-3.44050500	-4.22810000

4CzIPN

E = -2482.99925633 G = -2482.338773

6	-0.21235600	-1.15444300	-0.40643600
6	0.50237300	0.00020900	-0.00006600
6	-0.21113100	1.15566800	0.40600200
6	-1.62553700	1.13658400	0.43537700
6	-2.33699900	0.00165000	0.00009800
6	-1.62671100	-1.13390000	-0.43562900
6	-2.34162700	-2.23188200	-1.01103700
7	-2.92483900	-3.11077300	-1.49296800
6	-2.33926500	2.23538100	1.01070200
7	-2.92159900	3.11491400	1.49252600
7	-3.73717600	0.00200600	0.00074200
6	-4.55455100	-0.84813400	0.76369800
6	-4.55518200	0.85101300	-0.76282000
6	-4.19244500	-1.82602600	1.69060200
6	-5.91063500	-0.53922100	0.48642300
6	-5.91103700	0.54021900	-0.48643400
6	-4.19385500	1.82956900	-1.68932200
6	-5.21962900	-2.52012200	2.33205100
1	-3.14631400	-2.04141700	1.91179100
6	-6.92291000	-1.24814500	1.14494400
6	-6.92387700	1.24780100	-1.14551100
6	-5.22160700	2.52232000	-2.33131900
1	-3.14789600	2.04656300	-1.90971300
6	-6.57044600	-2.23891800	2.06125400
1	-4.96603300	-3.29555300	3.05826500
1	-7.97299600	-1.02401700	0.94488200
6	-6.57220800	2.23917700	-2.06147300
1	-7.97377200	1.02218200	-0.94612400
1	-4.96861200	3.29824900	-3.05720900
7	0.46170700	2.31528500	0.81182100
6	1.50949500	2.37551900	1.74968000
6	0.39438100	3.55436800	0.13933700
6	1.91180400	1.42296500	2.68453200
6	2.12998200	3.64583900	1.66041700
6	1.42291100	4.39394900	0.63552600
6	-0.44593200	3.95795600	-0.89871700
6	2.99778200	1.73645300	3.50263700
1	1.40600500	0.46344400	2.77676900
6	3.21074100	3.94385500	2.49868100
6	1.59792500	5.66851600	0.08508000
6	-0.25141600	5.23343000	-1.43366100
1	-1.23193400	3.31048300	-1.28594000
6	3.64723400	2.97956900	3.40730800
1	3.34523800	0.99748400	4.22766200
1	3.70241100	4.91732600	2.44044900
6	0.75687200	6.08299900	-0.94784100
1	2.38959600	6.32367800	0.45506100
1	-0.90247500	5.57512700	-2.24124900
7	1.90471300	-0.00059500	0.00008200
6	2.72412000	-0.87991300	0.72818200
6	2.72538200	0.87750500	-0.72806900
6	2.36863400	-1.87900900	1.63378100
6	4.07983600	-0.56346400	0.46048000
6	4.08064400	0.55880600	-0.46072900
6	2.37134100	1.87717800	-1.63358400
6	3.39661400	-2.60267600	2.24037600
1	1.32876000	-2.09652300	1.86955800
6	5.09441600	-1.29827300	1.08457100
6	5.09628100	1.29174700	-1.08529600
6	3.40034500	2.59910000	-2.24051900
1	1.33179100	2.09641600	-1.86914600
6	4.74624700	-2.32312800	1.96490000
1	3.13961500	-3.40232200	2.93829500

1	6.14337400	-1.06731100	0.88631600
6	4.74958800	2.31719500	-1.96552200
1	6.14490300	1.05890900	-0.88745700
1	3.14445800	3.39917500	-2.93835300
7	0.45934700	-2.31464500	-0.81248500
6	0.39099700	-3.55373800	-0.14007000
6	1.50758600	-2.37547100	-1.74980600
6	-0.44993200	-3.95688500	0.89765000
6	1.41924900	-4.39391600	-0.63584700
6	2.12731800	-3.64612900	-1.66029200
6	1.91106200	-1.42307300	-2.68429600
6	-0.25626900	-5.23242900	1.43274500
1	-1.23584600	-3.30909400	1.28449700
6	1.59336400	-5.66856900	-0.08532300
6	3.20860500	-3.94455400	-2.49775000
6	2.99778100	-1.73676900	-3.50130200
1	1.40560300	-0.46345600	-2.77711500
6	0.75173900	-6.08255100	0.94733800
1	-0.90782900	-5.57373900	2.24009300
1	2.38476800	-6.32420800	-0.45503300
6	3.64651900	-2.98023800	-3.40567100
1	3.69971400	-4.91829500	-2.43931100
1	3.34631300	-0.99763800	-4.22564100
1	0.88135900	7.07588900	-1.38496700
1	-7.35259900	2.80005600	-2.57992500
1	-7.35039200	-2.80082500	2.57926400
1	0.87553500	-7.07549600	1.38453600
1	4.49497600	-3.19567400	-4.05853700
1	5.52811900	-2.90903200	2.45271500
1	5.53230700	2.90172400	-2.45363200
1	4.49511900	3.19476700	4.06099600

4INT1

E = -3462.34679840 G = -3461.857326

27	-1.05205900	-0.34142200	0.53850500
17	-1.21599100	-0.86683700	2.71956600
6	4.07754300	2.15158100	-0.50943300
6	2.73018400	2.42161200	-0.27067500
6	1.79185500	1.38381400	-0.14273800
6	2.23177600	0.04174900	-0.26119100
6	3.59242100	-0.21352800	-0.50138600
6	4.51130400	0.82810600	-0.62436100
1	4.78941200	2.97465300	-0.60617200
1	2.40693300	3.46012400	-0.18282100
1	3.94408000	-1.24332300	-0.58913200
1	5.56549100	0.60712700	-0.80670700
6	-0.12687300	3.27282000	1.15790700
6	-0.65913100	2.46739500	-1.42080700
6	-0.79094900	4.30527700	0.21364500
1	0.90083300	3.59254000	1.39192100
6	-0.43463700	3.97382900	-1.24112000
1	-1.73626100	2.24102400	-1.33412000
1	-1.88542600	4.23602800	0.33457600
1	-0.50454600	5.33199000	0.49258300
1	-1.04484900	4.55550800	-1.95245200
1	0.62019300	4.22457200	-1.45143900
6	1.40468800	-2.51006300	-1.50337800
6	1.65654300	-2.50015300	1.24630200
6	1.58531200	-3.87368800	-0.81023800
1	2.37991000	-2.19399100	-1.90835500
6	2.35911200	-3.63959900	0.49157800
1	0.71168100	-2.87784700	1.67249000
1	0.59291700	-4.29924800	-0.57849700
1	2.09148900	-4.58714600	-1.48067900
1	2.40910300	-4.54902300	1.11253100
1	3.40154200	-3.35456700	0.26265000
15	-0.00412600	1.69946600	0.14988600
15	1.01619400	-1.34180400	-0.08555600
6	-0.89144600	3.03503100	2.46124000
1	-0.39983400	2.27829100	3.08980400
1	-0.97846000	3.97138300	3.03664600
1	-1.90672200	2.66080500	2.25568300
6	0.36462100	-2.47698300	-2.61835500
1	0.31011300	-1.48246100	-3.08660600
1	0.61429400	-3.20778300	-3.40480800
1	-0.63822700	-2.71431200	-2.23385800
6	-0.14326800	1.83037100	-2.70307900
1	-0.61112800	2.30034600	-3.58319600
1	0.94925100	1.93121000	-2.80263100
1	-0.40304500	0.76092200	-2.71868700
6	2.43344500	-1.83601200	2.37502500
1	2.70698800	-2.58517200	3.13575500
1	3.35967500	-1.36045500	2.01791800
1	1.80767800	-1.07518800	2.86419300
8	-2.47627900	-0.14135300	-0.60322400
6	-3.58484000	-1.00471400	-0.64847900
6	-4.58931100	-0.55696500	0.42755700
6	-4.20741600	-0.89435600	-2.04757200
6	-3.15470800	-2.45845100	-0.37596700

1	-4.11371900	-0.61607800	1.41885200
1	-4.88027300	0.49000700	0.24879700
1	-5.49926500	-1.17966500	0.43179800
1	-3.47066700	-1.19409400	-2.80994000
1	-5.10075800	-1.53154800	-2.15186900
1	-4.49562300	0.14962200	-2.24625500
1	-4.01202900	-3.15064500	-0.39536500
1	-2.43164900	-2.79105700	-1.13748700
1	-2.67980100	-2.53445600	0.61662000

4TSbtm-RR

E = -4330.96159667 G = -4330.268493

27	-0.38388700	0.45985800	0.66583800
17	-0.94305000	2.25271700	2.08808400
6	1.24765600	-0.19021300	1.99409600
14	0.70364600	-0.56496000	3.73712200
6	-1.17252300	-0.68455200	3.89406400
1	-1.44445000	-1.24884600	4.80156600
1	-1.59898200	0.32616200	3.95513400
1	-1.63615800	-1.18752800	3.03369100
6	1.33815000	0.82691500	4.82656400
1	0.91709900	1.77602100	4.46066200
1	1.03038400	0.68415600	5.87586900
1	2.43688000	0.89223400	4.78876300
6	1.50597200	-2.21277800	4.20012400
1	2.60294700	-2.13480800	4.13427100
1	1.24207900	-2.50530200	5.23023600
1	1.18786500	-3.02424400	3.52636000
6	2.23524000	0.06624200	1.27510700
6	3.16771800	0.39865900	0.29396500
1	3.70493900	1.33543800	0.47124200
6	3.98266800	-0.66209900	-0.34192100
6	4.96252200	-0.31185100	-1.29146800
6	3.79063800	-2.02910700	-0.06672200
6	5.70019900	-1.29033900	-1.95838600
1	5.14210200	0.74517700	-1.50325300
6	4.53054500	-3.00743100	-0.73249100
1	3.06236100	-2.31713100	0.69400200
6	5.48504400	-2.64584500	-1.68837600
1	6.45335500	-0.99251100	-2.69248600
1	4.36938000	-4.06185600	-0.49246700
1	6.06502900	-3.41183200	-2.20822000
6	-3.74375600	-2.57058200	-3.23868600
6	-2.68868700	-2.71778700	-2.33925400
6	-2.31110200	-1.66722100	-1.48569800
6	-3.01671200	-0.44325500	-1.54555200
6	-4.07731300	-0.30924400	-2.45703700
6	-4.44056600	-1.36028200	-3.29837400
1	-4.01866200	-3.39807900	-3.89686000
1	-2.14588100	-3.66446500	-2.31234200
1	-4.63372600	0.62803900	-2.51037900
1	-5.26897900	-1.23610100	-3.99981000
6	-1.00194000	-3.57113700	0.35665900
6	0.60036900	-2.23162000	-1.43631500
6	0.39964200	-4.15494400	0.11198800
1	-1.71283300	-4.10288100	-0.29599900
6	0.82445300	-3.74523000	-1.29852900
1	1.42197000	-1.71512000	-0.92259600
1	1.10411200	-3.73512900	0.85178500
1	0.39811700	-5.24813100	0.25444900
1	1.87673000	-3.99496300	-1.50716300
1	0.21115200	-4.28511600	-2.04163600
6	-2.92919300	2.52043300	-1.17913500
6	-3.96904500	0.95539400	0.84599800
6	-4.00239200	3.15417900	-0.26852800
1	-3.37864100	2.28166400	-2.15651000
6	-4.89835100	2.04054900	0.28148200
1	-3.48263200	1.35437900	1.75062900
1	-3.49291900	3.65328000	0.57211400
1	-4.57293300	3.92364900	-0.81365900
1	-5.58682000	2.41232900	1.05888000
1	-5.52666900	1.61670200	-0.52260500
15	-0.87418400	-1.82184600	-0.33455900
15	-2.53253500	0.88163600	-0.35951400
6	-1.49125800	-3.67151000	1.79517300
1	-2.48748300	-3.22068700	1.92095300
1	-1.56153700	-4.72769800	2.10297300
1	-0.80328300	-3.16700700	2.48620500
6	-1.69226700	3.39081400	-1.38997200
1	-0.93579500	2.88141700	-2.00343900
1	-1.96839100	4.33532600	-1.88700300
1	-1.22584800	3.62829000	-0.42405500
6	0.53716700	-1.70938200	-2.86519000
1	1.50555700	-1.88102300	-3.36286200
1	-0.24131800	-2.21696300	-3.45421700
1	0.33115100	-0.63075300	-2.87210500
6	-4.62081900	-0.38238100	1.16919600
1	-5.39731800	-0.25381400	1.94070200
1	-5.09417300	-0.83234900	0.28277700

1	-3.88393900	-1.10219700	1.55959600
6	3.07113800	5.28875300	-1.07574000
6	2.11329300	4.81057200	-0.17359900
6	1.71329600	3.47506800	-0.20832700
6	2.27668400	2.59677200	-1.14858500
6	3.23898400	3.07987000	-2.04868500
6	3.63343800	4.41935800	-2.01489800
1	3.37844100	6.33707000	-1.04638200
1	1.67274300	5.48395000	0.56584400
1	0.97485300	3.10668600	0.50606700
1	3.67109200	2.39937500	-2.78767800
1	4.37745900	4.78672500	-2.72622700
6	1.87411400	1.16265300	-1.19014300
8	0.64822600	0.84082000	-0.95104100
1	2.38427300	0.57746400	-1.97697900

4TSbtm-RS

E = -4330.96522059 G = -4330.272472

27	-0.55229200	0.62022600	-0.70515000
17	-1.07641000	1.15693200	-2.93874600
6	0.58797700	2.52592600	-0.45599400
14	-0.58192200	3.96954000	-0.45944800
6	-2.33430600	3.35502900	-0.11059800
1	-3.03795100	4.20050300	-0.03759600
1	-2.65896100	2.71458800	-0.94516000
1	-2.40614600	2.77410700	0.82179100
6	-0.52211200	4.72029100	-2.17964000
1	-0.77812000	3.94654900	-2.91887800
1	-1.23481800	5.55729900	-2.26926900
1	0.48788800	5.09828100	-2.40455200
6	-0.04939700	5.25185700	0.81851900
1	0.98113200	5.58353300	0.61357600
1	-0.70812900	6.13492600	0.76206700
1	-0.08303700	4.86835600	1.84827300
6	1.73053100	2.07828900	-0.68406300
6	2.89279800	1.35607500	-0.94509700
1	3.38735600	1.57586100	-1.89706700
6	3.77332100	0.94823200	0.15807300
6	5.09554800	0.53502000	-0.09464500
6	3.29605900	0.85688000	1.47994600
6	5.89856400	0.03143600	0.92708800
1	5.48556900	0.59193300	-1.11291400
6	4.09980500	0.35174300	2.50180600
1	2.28220700	1.19844800	1.69179400
6	5.40574500	-0.06996400	2.23205900
1	6.91739000	-0.29257200	0.70060600
1	3.70663500	0.28973700	3.52034800
1	6.03487600	-0.46720800	3.03202000
6	-3.98374300	-2.30515400	3.27977500
6	-2.91672100	-1.41240000	3.17616900
6	-2.41761300	-1.02140800	1.92226800
6	-3.02827700	-1.53215500	0.75228700
6	-4.08950300	-2.44424700	0.87297900
6	-4.56511300	-2.83424100	2.12437400
1	-4.35360700	-2.59717000	4.26547000
1	-2.46388800	-1.03047800	4.09177300
1	-4.55859600	-2.85033300	-0.02500000
1	-5.39263900	-3.54360800	2.19845900
6	-0.66589600	0.92403700	3.34553600
6	0.43395700	-1.30572200	2.14843800
6	0.51024400	0.23848000	4.07984200
1	-1.59345100	0.77953200	3.91968500
6	0.58329800	-1.23380100	3.67280200
1	1.33867800	-0.90001300	1.67324000
1	1.44548700	0.74119500	3.78612700
1	0.41358600	0.36772200	5.17002500
1	1.53388500	-1.69535400	3.98879200
1	-0.22479900	-1.81066500	4.15470800
6	-2.40388100	-2.32918500	-2.06643700
6	-4.01778600	-0.12732700	-1.62949200
6	-3.40477700	-1.94921300	-3.17732500
1	-2.79061700	-3.19999600	-1.51083800
6	-4.59173500	-1.22444700	-2.54012800
1	-3.58518600	0.65402200	-2.27439200
1	-2.89948400	-1.26527300	-3.87859200
1	-3.71144900	-2.84253100	-3.74547700
1	-5.26211600	-0.78825600	-3.29944100
1	-5.20265600	-1.92937000	-1.94804800
15	-0.87545300	-0.01841000	1.72651700
15	-2.46596600	-0.87843600	-0.87517500
6	-0.44458200	2.42074700	3.14032500
1	-1.32824700	2.90931400	2.70732600
1	-0.22810700	2.91181700	4.10324500
1	0.40450700	2.60775800	2.46587900
6	-0.99931100	-2.62829900	-2.58569000
1	-0.30701700	-2.89601700	-1.77566900
1	-1.03254700	-3.46435900	-3.30361500
1	-0.58798200	-1.74460200	-3.09426200
6	0.17650900	-2.69743300	1.58696800

1	1.02268500	-3.36022700	1.83134000
1	-0.73541700	-3.14722600	2.00791300
1	0.08752400	-2.66211100	0.49319600
6	-4.99988200	0.50597300	-0.65278400
1	-5.81289000	1.00396900	-1.20601300
1	-5.45573100	-0.23998700	0.01550500
1	-4.51145900	1.26669800	-0.02527300
6	5.32738700	-3.01954100	-1.64953200
6	4.45497000	-3.02888900	-0.55677300
6	3.32901100	-2.20705000	-0.55051300
6	3.06255900	-1.35986800	-1.63723000
6	3.94187000	-1.35650800	-2.73169400
6	5.06649900	-2.18130000	-2.73949900
1	6.21027000	-3.66343600	-1.65307600
1	4.65936600	-3.67571200	0.29979000
1	2.64645400	-2.19902100	0.29743900
1	3.73762900	-0.69663800	-3.57978900
1	5.74249600	-2.17363700	-3.59812500
6	1.89873400	-0.45144600	-1.61738400
8	0.93265900	-0.68511500	-0.80024700
1	1.65271600	0.02322800	-2.58391300

4TSbtm-SR

E = -4330.96051282 G = -4330.267622

27	-0.22783500	0.24033700	-0.45352800
17	0.65474900	-0.03335100	-2.61430000
6	0.70987200	2.19596500	-0.18589700
14	0.23796300	3.47935000	-1.45112000
6	-1.24135100	2.92889600	-2.48533000
1	-1.67808700	3.78710700	-3.02251000
1	-0.90595500	2.18011200	-3.21700800
1	-2.03554900	2.46919400	-1.88078600
6	1.71954200	3.70658600	-2.58447200
1	1.92114000	2.75270400	-3.09586200
1	1.52402800	4.47985700	-3.34622700
1	2.61803000	3.99104700	-2.01510700
6	-0.14955100	5.08099000	-0.52577500
1	0.73295800	5.41196900	0.04472800
1	-0.42318600	5.88357400	-1.23100100
1	-0.98099500	4.95854600	0.18567800
6	1.66076200	1.85640600	0.55382000
6	2.70209600	1.24204900	1.23709700
1	2.85098400	1.53604900	2.28186900
6	3.93992500	0.94421200	0.48650700
6	5.14544100	0.71947300	1.17465900
6	3.93425300	0.79893400	-0.91466200
6	6.30451100	0.35468000	0.49046000
1	5.16585500	0.81920300	2.26275300
6	5.09722000	0.44460400	-1.59640100
1	3.00320400	0.93323700	-1.46721900
6	6.28837800	0.21615200	-0.90047600
1	7.22692900	0.17399400	1.04836100
1	5.06574400	0.33180400	-2.68315300
1	7.19668200	-0.06957000	-1.43654900
6	-5.13872000	-1.86632300	2.19989000
6	-4.27968500	-0.76831400	2.17700400
6	-3.27220500	-0.65524200	1.20416300
6	-3.13148200	-1.67606300	0.23441400
6	-4.00753100	-2.77362400	0.26719700
6	-5.00239800	-2.87266900	1.23956600
1	-5.91231700	-1.93805200	2.96792700
1	-4.39435000	0.00812100	2.93594300
1	-3.91722600	-3.56434600	-0.47944500
1	-5.67328800	-3.73495300	1.24700900
6	-3.19857100	2.27473500	1.52995900
6	-1.27335600	0.82969500	2.85867500
6	-2.41940100	3.01716100	2.62701300
1	-4.11794100	1.86915800	1.98267000
6	-1.96028100	1.96487000	3.63736600
1	-0.25518000	1.15621300	2.59523300
1	-1.54209300	3.51828400	2.18146100
1	-3.04243500	3.80178700	3.08696500
1	-1.28204400	2.38429400	4.39824800
1	-2.83734900	1.56651600	4.17772700
6	-1.30913400	-3.18767700	-1.58910300
6	-2.77749300	-1.08927600	-2.62245500
6	-1.68232500	-3.24853100	-3.08539600
1	-1.94046900	-3.89649400	-1.02978900
6	-2.97352700	-2.45419600	-3.29933500
1	-2.03561500	-0.51800000	-3.20410100
1	-0.87075100	-2.77894400	-3.66536900
1	-1.76899200	-4.29369300	-3.42425100
1	-3.21043100	-2.33239100	-4.36960300
1	-3.83105000	-2.97977700	-2.84149400
15	-2.10907900	0.77909800	1.16566100
15	-1.82426000	-1.46712500	-1.05040100
6	-3.58548900	3.12542400	0.32812900
1	-4.11510800	2.53595700	-0.43558400
1	-4.25469200	3.94383200	0.64041400

1	-2.70406000	3.57651800	-0.14435700
6	0.16051900	-3.48121800	-1.29795900
1	0.39187900	-3.37988600	-0.22822500
1	0.41743200	-4.50522600	-1.61533600
1	0.79999300	-2.77397800	-1.84500000
6	-1.18889300	-0.50356700	3.59129700
1	-0.56179500	-0.40101000	4.49229900
1	-2.18098800	-0.85317900	3.91291300
1	-0.74556700	-1.27441200	2.94701800
6	-4.03501200	-0.25047100	-2.43931000
1	-4.48655700	-0.01801500	-3.41738800
1	-4.78987100	-0.77371300	-1.83201500
1	-3.80884700	0.70714000	-1.94511200
6	5.08915300	-3.21219100	1.06312800
6	4.25821000	-2.87879200	-0.01234000
6	3.14117900	-2.07078000	0.18609800
6	2.85086200	-1.57395700	1.46762800
6	3.69434200	-1.90173900	2.53959300
6	4.80237100	-2.72575600	2.34209900
1	5.96498000	-3.84550400	0.90231600
1	4.49189500	-3.24127000	-1.01599600
1	2.49951400	-1.78623800	-0.64724500
1	3.47496700	-1.50496000	3.53519700
1	5.44996400	-2.98222500	3.18417200
6	1.70492200	-0.67185900	1.67566100
8	0.64675000	-0.78927300	0.95477100
1	1.55911100	-0.36034400	2.72673700

4TSbtm-SS

E = -4330.96116519 G = -4330.267873

27	0.29373100	0.52593900	0.42878000
17	-0.42400900	1.09875300	2.58510000
6	-0.85450600	2.12987900	-0.52324800
14	-0.24797000	3.82283700	-0.05489700
6	1.35207500	3.70929300	0.94191700
1	1.84756200	4.69254600	0.99967000
1	1.11183000	3.36864100	1.95951600
1	2.07041500	2.99447200	0.51571400
6	-1.58214100	4.57522200	1.03415000
1	-1.70629800	3.94102200	1.92574800
1	-1.30670900	5.59181900	1.36114000
1	-2.54724900	4.62391500	0.50619500
6	-0.00643600	4.84868200	-1.62212200
1	-0.96115400	4.93891200	-2.16455300
1	0.34311900	5.86452700	-1.37259400
1	0.72658700	4.39844700	-2.30859800
6	-1.85404500	1.44734700	-0.83589500
6	-2.88675000	0.53105300	-1.00965900
1	-3.00040500	0.11767000	-2.01841700
6	-4.14140500	0.69989800	-0.25269700
6	-5.35193200	0.20471100	-0.76974000
6	-4.14497100	1.26412500	1.03976900
6	-6.53169800	0.27818400	-0.02689700
1	-5.36019600	-0.25119500	-1.76265700
6	-5.32475400	1.33199300	1.77943600
1	-3.20782300	1.62731200	1.46856300
6	-6.52573100	0.84160000	1.25187000
1	-7.46107300	-0.11259200	-0.44945300
1	-5.30561000	1.76825900	2.78152100
1	-7.44818500	0.89777200	1.83482400
6	5.08990600	-2.43703400	-1.52361400
6	4.17275900	-1.45133300	-1.88612000
6	3.21816100	-0.97669000	-0.97088000
6	3.19253300	-1.51322900	0.33798700
6	4.12286400	-2.50526400	0.68884300
6	5.06461400	-2.96665600	-0.23033500
1	5.82124400	-2.79533600	-2.25179000
1	4.19888500	-1.05579500	-2.90334000
1	4.11712900	-2.92219400	1.69748300
1	5.78046400	-3.73830300	0.06253500
6	2.87538300	1.48878700	-2.57360700
6	0.94765800	-0.45354300	-2.84598000
6	1.87842600	1.68039000	-3.72721200
1	3.73896000	0.92870800	-2.96756900
6	1.39117500	0.28489100	-4.12275100
1	-0.07609800	-0.14571000	-2.58414900
1	1.02681000	2.29341400	-3.38409700
1	2.34828400	2.21756800	-4.56742400
1	0.57066200	0.32223100	-4.85726700
1	2.22054800	-0.26837200	-4.59801800
6	1.49881400	-2.20662600	2.70258600
6	3.00126600	0.11108600	2.78282100
6	1.91917000	-1.67766800	4.08969800
1	2.13358800	-3.06877800	2.44048900
6	3.21943100	-0.88423500	3.93265100
1	2.29268300	0.88201200	3.12674300
1	1.12799800	-1.00556800	4.46077900
1	2.01548100	-2.50675900	4.80948400
1	3.49629600	-0.35805400	4.86153500

1	4.05763400	-1.56222100	3.68931500
15	1.96510800	0.29723700	-1.43886800
15	1.96430200	-0.82255300	1.52640900
6	3.38777700	2.76919500	-1.92786800
1	4.06041600	2.55712700	-1.08243200
1	3.95321900	3.36559200	-2.66247200
1	2.56499200	3.39154100	-1.55559800
6	0.02961600	-2.61185200	2.60310300
1	-0.21845100	-2.98679400	1.60045800
1	-0.19575100	-3.40254400	3.33759500
1	-0.61800700	-1.74732600	2.80925600
6	0.98231900	-1.97350000	-2.93443900
1	0.27739900	-2.31925100	-3.70837600
1	1.98187200	-2.34616400	-3.20231700
1	0.68791800	-2.42184200	-1.97653000
6	4.25123200	0.78347500	2.23103100
1	4.74697500	1.37544100	3.01750300
1	4.97755600	0.04848600	1.85105900
1	4.00470000	1.47094200	1.40629000
6	-4.44714200	-4.27260800	-1.46579300
6	-3.23156100	-3.98826100	-2.10141800
6	-2.41092200	-2.96956500	-1.62125500
6	-2.79490300	-2.21570300	-0.49929000
6	-4.01255800	-2.51068300	0.13710100
6	-4.83262700	-3.53266000	-0.34495200
1	-5.08922000	-5.07251500	-1.84288800
1	-2.92397500	-4.56870200	-2.97514900
1	-1.46279200	-2.74204800	-2.10574600
1	-4.31296400	-1.93390600	1.01352700
1	-5.77802800	-3.75008600	0.15782300
6	-1.94953600	-1.11389600	0.01246800
8	-0.68696700	-1.09129800	-0.24218600
1	-2.28703900	-0.69789900	0.97787800

4TStop-RR

E = -4330.96618718 G = -4330.273778

27	-0.28651200	0.56301600	0.51792400
17	0.54599800	0.82654100	2.69311200
6	0.81159800	2.28936000	-0.24107800
14	0.04640500	3.90648600	0.23862100
6	-1.39520400	3.62265600	1.42072200
1	-1.90286700	4.57494200	1.64709100
1	-2.14750400	2.92875200	1.01551700
1	-1.01846800	3.18657700	2.35787600
6	-0.55923000	4.78121600	-1.32220300
1	-1.32702100	4.18992400	-1.84480400
1	-0.99946800	5.76182900	-1.07610500
1	0.27319900	4.94486400	-2.02491800
6	1.36860900	4.91773900	1.11228500
1	2.22771600	5.10182600	0.44816800
1	0.97451100	5.88913800	1.45435700
1	1.73030400	4.35918500	1.99011400
6	1.83919200	1.68600900	-0.62173900
6	2.87754200	0.83120100	-0.96229200
1	2.96902300	0.59764800	-2.02929300
6	4.13994200	0.84963700	-0.20510700
6	5.34325100	0.46754200	-0.82599600
6	4.16279100	1.15585800	1.17175600
6	6.53400100	0.39945700	-0.10029200
1	5.33693600	0.21462300	-1.88892500
6	5.35380700	1.08303500	1.89255100
1	3.23187300	1.42853600	1.67582000
6	6.54680900	0.70566000	1.26318700
1	7.45748000	0.10169400	-0.60392700
1	5.35019800	1.31889100	2.95994300
1	7.47778700	0.65097300	1.83260900
6	-4.64980400	-2.69544000	-1.77874800
6	-3.90801900	-1.53148100	-1.97692800
6	-3.02176700	-1.05868400	-0.99281500
6	-2.88160000	-1.79048200	0.20921100
6	-3.64466500	-2.95503700	0.40007300
6	-4.52229300	-3.40864900	-0.58295700
1	-5.33251000	-3.04381800	-2.55731100
1	-4.02906200	-0.98661000	-2.91388000
1	-3.54865000	-3.51599100	1.33175800
1	-5.10396500	-4.31892400	-0.41972400
6	-1.66569700	0.60329400	-3.02609700
6	-3.43430000	1.83796700	-1.30826100
6	-2.45241700	1.81210900	-3.57510100
1	-2.07978500	-0.32121800	-3.45759900
6	-3.76738400	1.94909800	-2.80400400
1	-2.90961700	2.76100100	-1.01162000
1	-1.84788000	2.72319900	-3.42905100
1	-2.61232800	1.71160600	-4.66076700
1	-4.27483000	2.90359300	-3.02263800
1	-4.46994900	1.14805600	-3.08985000
6	-2.66293100	-1.05597700	3.05547900
6	-0.63627000	-2.62716400	2.03492100
6	-1.79872200	-1.82713700	4.07046900

1	-3.59600200	-1.62039500	2.88641300
6	-1.25940300	-3.07654900	3.36772800
1	0.31241100	-2.11303600	2.26119300
1	-0.95705900	-1.18479900	4.37723300
1	-2.38196200	-2.07127200	4.97360500
1	-0.51807700	-3.61038000	3.98501000
1	-2.08319300	-3.78797700	3.17532400
15	-2.08793200	0.52792400	-1.18929200
15	-1.68072800	-1.17906100	1.46006500
6	-0.16875500	0.65241000	-3.32140400
1	0.35547700	-0.19846900	-2.86657600
1	0.00276600	0.63432000	-4.41017000
1	0.28419900	1.56757500	-2.91465800
6	-2.99520000	0.37752700	3.45824200
1	-3.63294700	0.86977600	2.70708100
1	-3.53900200	0.39390800	4.41712100
1	-2.07351100	0.96722800	3.56568900
6	-4.62391200	1.63339400	-0.37853300
1	-5.32640500	2.47821600	-0.46232800
1	-5.17061100	0.71008600	-0.62250300
1	-4.30674900	1.56666900	0.67354600
6	-0.37000500	-3.72410800	1.01180000
1	0.35997700	-4.44529800	1.41439300
1	-1.28431800	-4.28142000	0.75772000
1	0.04477900	-3.30176500	0.08624500
6	4.23128000	-3.86832400	-2.46226300
6	2.95565100	-3.49719600	-2.90682500
6	2.20382400	-2.57355400	-2.18365300
6	2.71979400	-2.00163700	-1.00739200
6	3.99321000	-2.39119600	-0.55789100
6	4.74412000	-3.31666300	-1.28480400
1	4.81992500	-4.59318700	-3.02999600
1	2.54706600	-3.93700900	-3.82025300
1	1.20294800	-2.28585700	-2.50949400
1	4.38976100	-1.96672200	0.36551600
1	5.73469100	-3.60899500	-0.92814700
6	1.93389700	-1.01181900	-0.24180100
8	0.66859200	-0.91911000	-0.43734600
1	2.32560400	-0.77942400	0.76340200

4TStop-RS

E = -4330.96372675 G = -4330.271458

27	-0.22720900	0.31389800	0.38205700
17	0.85321100	-0.03089600	2.44780200
6	0.64623000	2.31011800	0.15785100
14	0.04378500	3.58230800	1.37007300
6	-1.20789400	2.85552800	2.57745700
1	-1.65236300	3.65794600	3.18957100
1	-2.02835300	2.31939800	2.07797700
1	-0.70325700	2.13706700	3.23914100
6	-0.75232400	4.99215700	0.39557700
1	-1.64552500	4.65299900	-0.15236200
1	-1.06323600	5.80538300	1.07239600
1	-0.04489000	5.40808900	-0.33924300
6	1.54276200	4.18410200	2.33161900
1	2.29919000	4.61464400	1.65691400
1	1.26457600	4.94436200	3.08036400
1	1.99962300	3.32929500	2.85487100
6	1.59982800	1.99964900	-0.58873700
6	2.61585300	1.44041700	-1.35383700
1	2.66576900	1.77317000	-2.39682700
6	3.92396600	1.19139200	-0.71627600
6	5.07846300	1.04948900	-1.50673800
6	4.04433100	1.02417100	0.67750800
6	6.31287200	0.75435400	-0.92823600
1	5.00048700	1.16735800	-2.59100800
6	5.28008800	0.73361400	1.25238100
1	3.15526700	1.09609600	1.30706800
6	6.42158600	0.59471800	0.45607900
1	7.19578200	0.64471500	-1.56342900
1	5.34838000	0.60176200	2.33532500
1	7.38793200	0.36233500	0.91014700
6	-5.04847100	-2.08533600	-2.07904000
6	-4.33657800	-0.89190800	-1.96707900
6	-3.25073900	-0.77425200	-1.08124300
6	-2.88616400	-1.88993700	-0.29269800
6	-3.61793300	-3.08421900	-0.41060100
6	-4.68770800	-3.18752600	-1.29777800
1	-5.88870900	-2.15441100	-2.77390600
1	-4.63913100	-0.04072300	-2.57894100
1	-3.34710200	-3.94560100	0.20271600
1	-5.24157100	-4.12566600	-1.37986400
6	-2.24231100	1.54021500	-2.61395200
6	-3.59785800	2.05178700	-0.27192300
6	-2.94317800	2.90553600	-2.49280300
1	-2.86909100	0.88171000	-3.23555900
6	-4.12452300	2.75440400	-1.53279500
1	-2.98616100	2.78143000	0.28213100
1	-2.22895400	3.64295000	-2.08809500

1	-3.25164200	3.27304000	-3.48496300
1	-4.58070900	3.72571800	-1.27869900
1	-4.91817400	2.14430700	-1.99794400
6	-2.20404500	-2.18091200	2.54155400
6	-0.39170100	-3.23011600	0.75284200
6	-1.22155600	-3.23795800	3.07819500
1	-3.16250900	-2.67870200	2.31597000
6	-0.83115000	-4.14537700	1.90708500
1	0.58802400	-2.80685400	1.02183000
1	-0.32451600	-2.72398900	3.46172500
1	-1.67017900	-3.79818700	3.91498900
1	-0.02415300	-4.84634600	2.17732800
1	-1.69589200	-4.76046000	1.59852300
15	-2.31572200	0.80879300	-0.87701600
15	-1.47508500	-1.70652000	0.87480100
6	-0.84594600	1.59889300	-3.22296900
1	-0.41091000	0.59465600	-3.30552900
1	-0.89343300	2.04621000	-4.22952600
1	-0.16986800	2.20520800	-2.60386100
6	-2.45117500	-0.99975500	3.47422100
1	-3.17526000	-0.29109200	3.04216600
1	-2.86194300	-1.34819000	4.43618200
1	-1.51428700	-0.45764600	3.66423000
6	-4.65815000	1.49423600	0.66903600
1	-5.31181300	2.30336700	1.03294400
1	-5.28934900	0.74544800	0.16747600
1	-4.20366700	1.01220300	1.54819700
6	-0.26791600	-3.86206900	-0.62671400
1	0.49843700	-4.65370300	-0.61072100
1	-1.21407700	-4.31211600	-0.96393500
1	0.04101500	-3.10862500	-1.36490000
6	4.86318200	-3.12387200	-0.72020000
6	3.95337100	-2.69448300	0.25212600
6	2.90525800	-1.84277600	-0.08997800
6	2.75453900	-1.40813000	-1.41767800
6	3.68106100	-1.82877600	-2.38422300
6	4.72440100	-2.68829400	-2.04129500
1	5.68590000	-3.78884800	-0.44633100
1	4.07303800	-3.01023800	1.29130500
1	2.22549500	-1.47199000	0.67802800
1	3.57541800	-1.47902300	-3.41506200
1	5.43650400	-3.01375500	-2.80356600
6	1.66163700	-0.49457400	-1.79997100
8	0.51913000	-0.54923600	-1.20943300
1	1.64337200	-0.24431300	-2.87637100

4TStop-SR

E = -4330.96448961 G = -4330.271923

27	0.52769600	0.37706500	-0.84868300
17	0.62322200	0.01861800	-3.16594900
6	-0.76350400	2.21496700	-1.15285500
14	0.28202200	3.68325300	-1.56996600
6	2.01092000	3.12151300	-2.06822900
1	2.66717500	3.99247300	-2.23179300
1	2.48284000	2.48150600	-1.30686000
1	1.95570800	2.52991800	-2.99382100
6	0.36884300	4.79655100	-0.04884700
1	0.82063500	4.27291900	0.80631500
1	0.97456600	5.69453500	-0.25449100
1	-0.63852100	5.12404900	0.25359000
6	-0.51886400	4.59180300	-3.00847500
1	-1.53189400	4.93344700	-2.74322400
1	0.07745200	5.46954000	-3.30865400
1	-0.60192700	3.91590400	-3.87420200
6	-1.82944200	1.57873000	-1.22566100
6	-2.90134800	0.67322500	-1.24522100
1	-3.19449700	0.37110700	-2.25841900
6	-4.04184900	0.86241000	-0.32061000
6	-5.27112100	0.23215400	-0.58770900
6	-3.91838500	1.58659000	0.87784500
6	-6.32191500	0.28959000	0.32605400
1	-5.39313100	-0.32585100	-1.51804200
6	-4.97225800	1.65040500	1.78994100
1	-2.98387600	2.10516300	1.08707300
6	-6.17755200	0.99370700	1.52550600
1	-7.26064800	-0.22273000	0.10122400
1	-4.85001900	2.21829500	2.71622000
1	-7.00031900	1.03700400	2.24312400
6	3.53925200	-2.04773700	3.61127500
6	2.88662800	-0.88234200	3.21084400
6	2.44947800	-0.71412100	1.88459500
6	2.66665400	-1.75841500	0.95718700
6	3.33622200	-2.92212400	1.37168800
6	3.77189000	-3.07057800	2.68703900
1	3.86975000	-2.15601400	4.64696000
1	2.71940900	-0.09823600	3.94945500
1	3.51571300	-3.72525900	0.65433000
1	4.28639700	-3.98416800	2.99399200
6	0.68920400	1.45979700	2.79855300

6	3.01883500	2.13702700	1.49529700
6	1.43273900	2.71599900	3.30740000
1	0.76769900	0.65643000	3.54628400
6	2.91964000	2.61618400	2.95180800
1	2.68689900	2.96156900	0.84289200
1	1.00240200	3.60390200	2.81536100
1	1.27337200	2.85217700	4.38912300
1	3.43569400	3.58232900	3.08123300
1	3.43303600	1.89836200	3.61273000
6	3.49926100	-1.87620700	-1.86369900
6	1.10446800	-3.09296300	-1.23368000
6	2.96225500	-2.91962200	-2.86088900
1	4.25604300	-2.36047400	-1.22299800
6	2.09808300	-3.91115800	-2.07590900
1	0.34747500	-2.67778500	-1.91850600
1	2.34192400	-2.40265400	-3.61105700
1	3.79218000	-3.41386100	-3.39241400
1	1.56407600	-4.61057600	-2.74026700
1	2.73408800	-4.52649700	-1.41370100
15	1.65544100	0.85509600	1.30107500
15	2.02547400	-1.53257300	-0.74980900
6	-0.78486400	1.68986800	2.48319800
1	-1.24642800	0.78385800	2.06852100
1	-1.33587800	1.98883700	3.38983400
1	-0.90415200	2.48366300	1.73126800
6	4.09498400	-0.62659100	-2.50449500
1	4.50381900	0.05821500	-1.74507800
1	4.92006800	-0.89731100	-3.18388000
1	3.32646900	-0.08826600	-3.07732300
6	4.40226900	1.68437300	1.04539700
1	5.12748500	2.50783300	1.14636000
1	4.76294500	0.83718700	1.64830500
1	4.40007200	1.37003900	-0.00927800
6	0.39586200	-3.83462100	-0.10718900
1	-0.23632400	-4.63676700	-0.52164300
1	1.10834600	-4.29748000	0.59230900
1	-0.25141200	-3.15110100	0.45944800
6	-5.08071400	-3.32629000	1.00122600
6	-4.25854900	-2.53824200	1.81439100
6	-3.20218000	-1.81938500	1.25819000
6	-2.96352000	-1.86549700	-0.12380800
6	-3.78838500	-2.66055500	-0.93200300
6	-4.83855400	-3.39148200	-0.37383300
1	-5.90849900	-3.88906900	1.43957900
1	-4.44792700	-2.48171200	2.88907900
1	-2.55563000	-1.20076200	1.88097800
1	-3.60396700	-2.69837200	-2.00959400
1	-5.47289000	-4.01054000	-1.01323200
6	-1.88593700	-1.04994000	-0.73379700
8	-0.85343400	-0.74973200	0.00215000
1	-1.68420800	-1.29969600	-1.79138300

4TStop-SS

E = -4330.96221605 G = -4330.269804

27	0.30092800	-0.06245000	0.75228800
17	0.27890100	1.06186100	2.82236500
6	-1.18368400	-1.50002000	1.44744000
14	-0.51738100	-2.72494600	2.67213800
6	1.25016900	-2.29793400	3.17153800
1	1.69465400	-3.12610800	3.74836700
1	1.89960100	-2.10268900	2.30478900
1	1.25313400	-1.38705100	3.78697400
6	-0.54544200	-4.41911100	1.83441300
1	0.09395400	-4.43184900	0.93800300
1	-0.18307200	-5.20417900	2.51866900
1	-1.56791800	-4.68222400	1.51974500
6	-1.61206600	-2.71523100	4.20112800
1	-2.64885300	-2.98547400	3.94533600
1	-1.24545900	-3.41969800	4.96624500
1	-1.61951800	-1.70258200	4.63475700
6	-2.25150700	-0.97540900	1.04895800
6	-3.27329400	-0.20909500	0.52459400
1	-3.59433600	0.61872500	1.16484700
6	-4.30632400	-0.72887400	-0.38228000
6	-5.43022300	0.06832000	-0.67926200
6	-4.19909000	-1.98130000	-1.01887000
6	-6.39682500	-0.36209500	-1.58920500
1	-5.53880400	1.03633000	-0.18484600
6	-5.16632700	-2.40926100	-1.92698900
1	-3.34949400	-2.62363900	-0.78393100
6	-6.27039200	-1.60175600	-2.22320600
1	-7.25982800	0.27478200	-1.80096700
1	-5.06152400	-3.38719700	-2.40459500
1	-7.02840600	-1.94002200	-2.93350600
6	4.18782300	0.03375700	-3.71075700
6	3.30717800	-0.79944300	-3.02199200
6	2.69294200	-0.37954700	-1.82854000
6	2.97466400	0.91483200	-1.33390800
6	3.87321300	1.73925900	-2.03262200

6	4.47623300	1.30787200	-3.21262600
1	4.65296700	-0.31385400	-4.63618400
1	3.10143600	-1.79107100	-3.42616800
1	4.10326700	2.73440400	-1.64760700
1	5.16805500	1.96434500	-3.74555700
6	0.68376900	-2.51805700	-2.15142600
6	2.69935500	-2.90009900	-0.31274800
6	1.18919500	-3.96298200	-1.95235000
1	1.03612700	-2.14151900	-3.12385600
6	2.63446200	-3.92874700	-1.45147800
1	2.16857400	-3.32464200	0.55544800
1	0.55517700	-4.45819800	-1.19762900
1	1.08163400	-4.54391700	-2.88246400
1	2.97277400	-4.91921600	-1.10375100
1	3.32053500	-3.63022800	-2.26204200
6	3.54236200	1.95753200	1.35138500
6	1.51519900	3.20386400	-0.03428300
6	3.15186600	3.37630300	1.80494800
1	4.43217400	2.03125800	0.70309700
6	2.58474500	4.11479900	0.58893500
1	0.64546400	3.20823500	0.63955100
1	2.37513800	3.29559200	2.58321000
1	4.01810000	3.89507500	2.24754500
1	2.15286300	5.09186700	0.86205800
1	3.38817700	4.31634300	-0.14262700
15	1.57643600	-1.48656000	-0.85050800
15	2.13340700	1.45188900	0.21144300
6	-0.83329600	-2.38449800	-2.08904500
1	-1.15145100	-1.34544600	-2.24236200
1	-1.30900100	-3.01537700	-2.85737200
1	-1.20849200	-2.69426900	-1.10437600
6	3.81996400	0.97903400	2.48770600
1	4.13748500	-0.00303100	2.10484800
1	4.62978900	1.35977700	3.13173900
1	2.91806100	0.83468800	3.09861500
6	4.10017600	-2.49020400	0.12239200
1	4.66622100	-3.36925000	0.47037200
1	4.65976300	-2.03074400	-0.70639300
1	4.06953600	-1.76567800	0.94977200
6	1.03162400	3.56139900	-1.43289000
1	0.55093500	4.55291500	-1.42240900
1	1.85525100	3.59114000	-2.16223300
1	0.28796800	2.83094500	-1.78103100
6	-3.52988400	4.83264700	0.27817600
6	-2.52260700	4.22483900	1.03955300
6	-2.01949600	2.97742800	0.67685200
6	-2.52343700	2.31795000	-0.45863500
6	-3.54255600	2.92415500	-1.21057300
6	-4.04061500	4.17741100	-0.84546900
1	-3.91971300	5.81216800	0.56584400
1	-2.13272000	4.72411100	1.93013600
1	-1.26766900	2.48582500	1.29771800
1	-3.93858900	2.40897200	-2.08887200
1	-4.82825000	4.64462800	-1.44183600
6	-2.00155900	0.99678800	-0.87414000
8	-0.77135400	0.69058000	-0.72529700
1	-2.53344400	0.53908400	-1.72696900

OtBu

E = -233.253839501 G = -233.163822

8	-0.00002000	-0.00010600	1.47897100
6	0.00000900	-0.00004000	0.16416100
6	-1.37868300	-0.48137400	-0.43763800
6	0.27240400	1.43467000	-0.43752200
6	1.10627800	-0.95318000	-0.43766400
1	-1.58640200	-1.49446700	-0.04989800
1	-2.17191200	0.18232200	-0.05013800
1	-1.44068900	-0.50315600	-1.54664400
1	1.24386900	1.78966400	-0.05010400
1	0.28445800	1.49915800	-1.54652500
1	-0.50099500	2.12123900	-0.04978400
1	1.15595600	-0.99613600	-1.54666400
1	2.08747900	-0.62628500	-0.05007000
1	0.92834600	-1.97194900	-0.04996600

PyH

E = -862.169254021 G = -861.91528

6	-1.19724700	0.14311800	0.00009700
6	-1.24293200	1.54797200	0.00015000
6	0.00001800	2.20448700	-0.00012100
6	1.24295100	1.54795500	-0.00025500
6	1.19723900	0.14308900	-0.00022900
7	-0.00000400	-0.46229700	-0.00009600
1	0.00001400	3.29715100	-0.00014400
1	-0.00004800	-1.49913400	-0.00002200
6	2.32601300	-0.86304900	-0.00021500
8	2.07412000	-2.04470000	-0.00022500
8	3.51819600	-0.30959400	-0.00013900

6	-2.32603500	-0.86299500	0.00026800
8	-2.07410500	-2.04464700	0.00033100
8	-3.51823200	-0.30954000	0.00032200
6	4.67089000	-1.21041300	-0.00011700
6	5.92016400	-0.36442800	0.00042800
1	4.58891600	-1.85310000	-0.88921900
1	4.58847100	-1.85363800	0.88855100
1	6.80218600	-1.02203100	-0.00016400
1	5.97046600	0.27399300	-0.89427900
1	5.97067100	0.27267300	0.89605900
6	-4.67088300	-1.21041700	0.00038500
6	-5.92017800	-0.36446800	-0.00095200
1	-4.58913400	-1.85266700	0.88982600
1	-4.58816200	-1.85407300	-0.88794100
1	-6.80218600	-1.02208900	-0.00059800
1	-5.97091200	0.27419900	0.89355300
1	-5.97023500	0.27237300	-0.89679500
6	2.52020700	2.33525300	-0.00027900
1	3.13219100	2.08961700	0.87946000
1	3.13230700	2.08955500	-0.87990700
1	2.30822000	3.41194400	-0.00032200
6	-2.52019100	2.33524700	0.00055900
1	-3.13224800	2.09004000	-0.87925100
1	-3.13218500	2.08904300	0.88011900
1	-2.30823400	3.41194500	0.00111900

Py

E = -861.718224527 G = -861.477726

6	-1.14417400	0.04981200	0.16430300
6	-1.21602500	1.46227200	0.16714600
6	-0.00001100	2.13344100	0.00007000
6	1.21602200	1.46225100	-0.16695000
6	1.14415100	0.04980500	-0.16400000
7	-0.00002400	-0.60771700	0.00015800
1	-0.00002700	3.22720100	-0.00003000
6	2.33328200	-0.85420900	-0.38035100
8	2.28477400	-1.92254400	-0.93096400
8	3.46701200	-0.33069300	0.12766100
6	-2.33337300	-0.85413900	0.38065500
8	-2.28501100	-1.92228300	0.93165300
8	-3.46692900	-0.33082000	-0.12790500
6	4.65797400	-1.11842100	-0.02791000
6	5.80849200	-0.34887100	0.58372000
1	4.81486800	-1.32058900	-1.09971700
1	4.50446000	-2.09360700	0.46121300
1	6.74016500	-0.92800000	0.49180500
1	5.95191900	0.61758900	0.07620700
1	5.62662500	-0.15433500	1.65183600
6	-4.65798500	-1.11842400	0.02758700
6	-5.80820900	-0.34888900	-0.58460600
1	-4.81518900	-1.32025800	1.09941100
1	-4.50441300	-2.09377100	-0.46119900
1	-6.74013500	-0.92753600	-0.49224800
1	-5.95113400	0.61800900	-0.07779300
1	-5.62627000	-0.15518400	-1.65285900
6	2.48582100	2.25185500	-0.34513200
1	3.13289000	2.16050600	0.53957900
1	3.07633100	1.88703200	-1.19763400
1	2.25667100	3.31510400	-0.50625600
6	-2.48585900	2.25185000	0.34521700
1	-3.13326300	2.15969100	-0.53915800
1	-3.07595300	1.88762500	1.19826500
1	-2.25679500	3.31526200	0.50540900

radical

E = -756.041285178 G = -755.855928

6	-1.35172800	-0.53558300	-0.00115600
14	-3.07600300	0.11296900	0.00012200
6	-3.94311400	-0.47743200	1.56588300
1	-4.98133300	-0.10677500	1.59633000
1	-3.97121000	-1.57781900	1.60990400
1	-3.42302700	-0.11452700	2.46656600
6	-3.96978300	-0.54152900	-1.52455500
1	-3.99776500	-1.64279600	-1.52274100
1	-5.00870000	-0.17259300	-1.55191900
1	-3.46596000	-0.21588700	-2.44842100
6	-3.00083700	1.99528000	-0.03947300
1	-2.47827100	2.34971300	-0.94227100
1	-4.01612700	2.42551100	-0.04092000
1	-2.46508900	2.38685800	0.83996100
6	-0.19246600	-0.96474800	-0.00113900
6	1.10957200	-1.43880100	-0.00094500
1	1.25903700	-2.52393600	-0.00085400
6	2.28064500	-0.60649100	-0.00057900
6	3.56942200	-1.19802000	0.00033500
6	2.19697500	0.81002900	-0.00098900
6	4.71655400	-0.41192000	0.00096100
1	3.65185900	-2.28804300	0.00065200

6	3.34820100	1.58823400	-0.00038100	1	5.70040800	-0.88744200	0.00172700
1	1.21060600	1.27855600	-0.00176900	1	3.26454000	2.67787300	-0.00067800
6	4.61467000	0.98558200	0.00063700	1	5.51640900	1.60215200	0.00113200

■ Proof of Stereochemistry: X-ray Characterization Data

1-(4-((1*R*,2*R*)-1-hydroxy-2-phenyl-4-(trimethylsilyl)but-3-yn-1-yl)phenyl)ethan-1-one (3e).

The absolute configurations of all the products are assigned as (1*R*,2*R*) by analogy to that of **3e (**CCDC number: 2219864), whose absolute configuration was determined to be (1*R*,2*R*) by X-ray crystallographic analysis (vide infra).

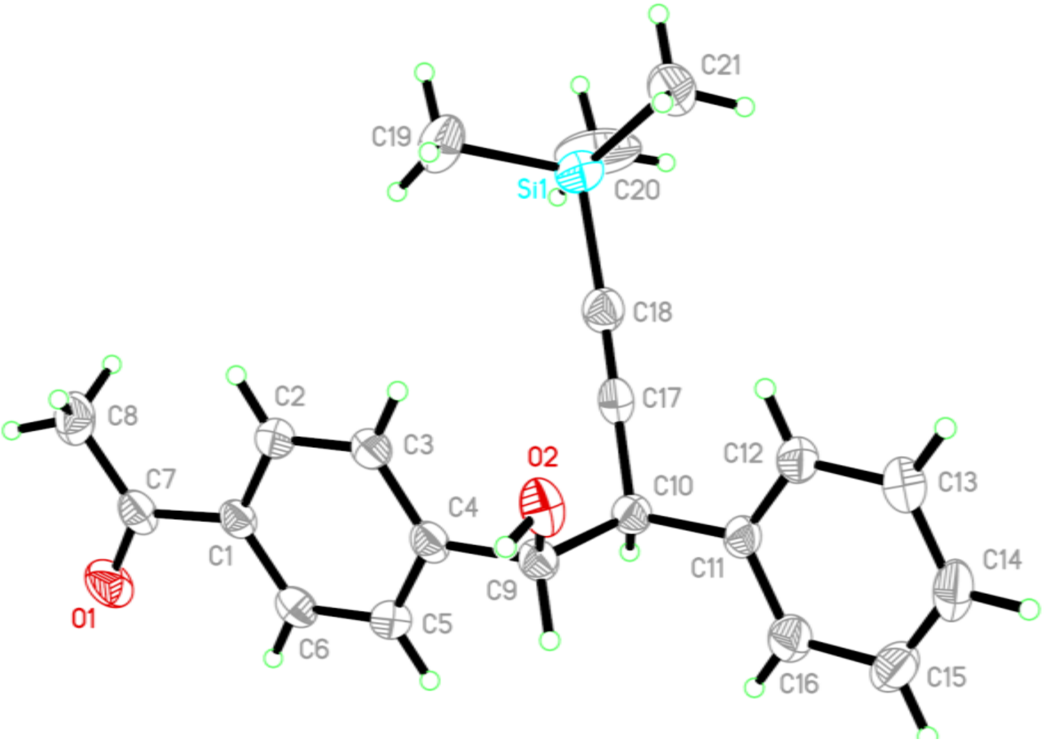

Supplementary Table 7. Crystal data and structure refinement for t_a.

**Identification code**	**t_a**
**Empirical formula**	**C21 H24 O2 Si**
**Formula weight**	**336.49**
**Temperature**	**223(2) K**

**Wavelength**	**1.54178 A**
**Crystal system, space group**	**Monoclinic, P2(1)**
**Unit cell dimensions**	**a = 10.2881(7) A alpha = 90 deg.**
	**b = 5.6047(4) A beta = 103.621(4) deg.**
	**c = 17.1021(12) A gamma = 90 deg.**
**Volume**	**958.40(12) A^3**
**Z, Calculated density**	**2, 1.166 Mg/m^3**
**Absorption coefficient**	**1.144 mm^-1**
**F(000)**	**360**
**Crystal size**	**0.180 x 0.160 x 0.140 mm**
**Theta range for data collection**	**2.658 to 66.583 deg.**
**Limiting indices**	**-12<=h<=12, -6<=k<=6, -20<=l<=20**
**Reflections collected / unique**	**11178 / 3303 [R(int) = 0.0656]**
**Completeness to theta = 66.583**	**99.7 %**
**Absorption correction**	**Semi-empirical from equivalents**
**Max. and min. transmission**	**0.7523 and 0.7218**
**Refinement method**	**Full-matrix least-squares on F^2**

**Data / restraints / parameters**	**3303 / 1 / 222**
**Goodness-of-fit on F^2**	**1.044**
**Final R indices [I>2sigma(I)]**	**R1 = 0.0449, wR2 = 0.0998**
**R indices (all data)**	**R1 = 0.0681, wR2 = 0.1111**
**Absolute structure parameter**	**0.09(2)**
**Extinction coefficient**	**n/a**
**Largest diff. peak and hole**	**0.190 and -0.308 e.A^-3**

Supplementary Table 8. Atomic coordinates (x 10^4) and equivalent isotropic displacement parameters (A^2 x 10^3) for t_a.
U(eq) is defined as one third of the trace of the orthogonalized Uij tensor.

	x	y	z	U(eq)
O(1)	1968(2)	8250(6)	133(2)	58(1)
C(1)	3964(3)	6547(7)	874(2)	33(1)
O(2)	8754(2)	3923(5)	1561(1)	46(1)
C(2)	4620(3)	4606(7)	1311(2)	38(1)
C(3)	6005(3)	4536(7)	1550(2)	38(1)
C(4)	6765(3)	6392(7)	1363(2)	35(1)
C(5)	6116(3)	8347(7)	948(2)	38(1)
C(6)	4731(3)	8408(7)	702(2)	38(1)
C(7)	2489(3)	6585(8)	556(2)	41(1)

C(8)	1646(4)	4598(8)	740(2)	54(1)
C(9)	8287(3)	6297(7)	1585(2)	36(1)
C(10)	8891(3)	7290(7)	2439(2)	34(1)
C(11)	10413(3)	7230(6)	2620(2)	34(1)
C(12)	11141(3)	5351(8)	3018(2)	41(1)
C(13)	12520(3)	5279(8)	3134(2)	46(1)
C(14)	13183(4)	7078(8)	2852(2)	51(1)
C(15)	12478(4)	8982(8)	2455(2)	52(1)
C(16)	11083(3)	9062(7)	2337(2)	44(1)
C(17)	8373(3)	5991(7)	3052(2)	37(1)
C(18)	7960(3)	4879(8)	3541(2)	42(1)
C(19)	5920(4)	1205(10)	3615(3)	74(2)
C(20)	6767(6)	4834(11)	4981(3)	97(2)
C(21)	8693(4)	896(8)	4717(3)	62(1)
Si(1)	7336(1)	2945(2)	4235(1)	46(1)

Supplementary Table 9. Bond lengths [A] and angles [deg] for t_a.

O(1)-C(7)	1.224(5)
C(1)-C(6)	1.381(5)
C(1)-C(2)	1.400(5)
C(1)-C(7)	1.486(5)
O(2)-C(9)	1.419(5)
C(2)-C(3)	1.387(4)
C(3)-C(4)	1.383(5)
C(4)-C(5)	1.389(5)
C(4)-C(9)	1.523(5)
C(5)-C(6)	1.388(4)
C(7)-C(8)	1.490(6)
C(9)-C(10)	1.550(5)
C(10)-C(17)	1.475(5)
C(10)-C(11)	1.523(4)

**C(11)-C(12)**	**1.376(5)**
**C(11)-C(16)**	**1.386(5)**
**C(12)-C(13)**	**1.386(5)**
**C(13)-C(14)**	**1.368(6)**
**C(14)-C(15)**	**1.376(6)**
**C(15)-C(16)**	**1.402(5)**
**C(17)-C(18)**	**1.199(5)**
**C(18)-Si(1)**	**1.830(4)**
**C(19)-Si(1)**	**1.862(5)**
**C(20)-Si(1)**	**1.856(5)**
**C(21)-Si(1)**	**1.846(4)**

**C(6)-C(1)-C(2)**	**118.2(3)**
**C(6)-C(1)-C(7)**	**119.8(3)**
**C(2)-C(1)-C(7)**	**121.9(3)**
**C(3)-C(2)-C(1)**	**120.8(4)**
**C(4)-C(3)-C(2)**	**120.6(4)**
**C(3)-C(4)-C(5)**	**118.8(3)**
**C(3)-C(4)-C(9)**	**121.4(3)**
**C(5)-C(4)-C(9)**	**119.8(3)**
**C(6)-C(5)-C(4)**	**120.7(4)**
**C(1)-C(6)-C(5)**	**121.0(3)**
**O(1)-C(7)-C(1)**	**119.6(4)**
**O(1)-C(7)-C(8)**	**119.9(3)**
**C(1)-C(7)-C(8)**	**120.4(4)**
**O(2)-C(9)-C(4)**	**111.2(3)**
**O(2)-C(9)-C(10)**	**107.5(3)**
**C(4)-C(9)-C(10)**	**112.5(3)**
**C(17)-C(10)-C(11)**	**111.8(3)**
**C(17)-C(10)-C(9)**	**111.2(3)**
**C(11)-C(10)-C(9)**	**110.4(3)**
**C(12)-C(11)-C(16)**	**118.8(3)**
**C(12)-C(11)-C(10)**	**122.0(3)**

**C(16)-C(11)-C(10)**	**119.0(3)**
**C(11)-C(12)-C(13)**	**120.8(4)**
**C(14)-C(13)-C(12)**	**120.4(4)**
**C(13)-C(14)-C(15)**	**119.8(4)**
**C(14)-C(15)-C(16)**	**119.9(4)**
**C(11)-C(16)-C(15)**	**120.2(4)**
**C(18)-C(17)-C(10)**	**178.2(4)**
**C(17)-C(18)-Si(1)**	**174.9(4)**
**C(18)-Si(1)-C(21)**	**108.3(2)**
**C(18)-Si(1)-C(20)**	**108.9(2)**
**C(21)-Si(1)-C(20)**	**112.0(2)**
**C(18)-Si(1)-C(19)**	**106.7(2)**
**C(21)-Si(1)-C(19)**	**109.7(2)**
**C(20)-Si(1)-C(19)**	**111.1(3)**

Symmetry transformations used to generate equivalent atoms:

Supplementary Table 10. Anisotropic displacement parameters (A^2 x 10^3) for t_a. The anisotropic displacement factor exponent takes the form: -2 pi^2 [h^2 a*^2 U11 + ... + 2 h k a* b* U12]

	**U11**	**U22**	**U33**	**U23**	**U13**	**U12**
**O(1)**	**38(1)**	**82(2)**	**50(2)**	**19(2)**	**2(1)**	**14(2)**
**C(1)**	**32(2)**	**41(2)**	**24(2)**	**-1(2)**	**6(2)**	**2(2)**
**O(2)**	**39(1)**	**54(2)**	**41(1)**	**-11(1)**	**2(1)**	**6(1)**
**C(2)**	**36(2)**	**43(3)**	**34(2)**	**2(2)**	**7(2)**	**-5(2)**
**C(3)**	**34(2)**	**45(3)**	**34(2)**	**7(2)**	**5(2)**	**1(2)**
**C(4)**	**33(2)**	**46(2)**	**26(2)**	**0(2)**	**5(2)**	**3(2)**

C(5)	40(2)	39(3)	35(2)	4(2)	9(2)	-3(2)
C(6)	40(2)	39(3)	33(2)	5(2)	3(2)	7(2)
C(7)	34(2)	58(3)	28(2)	-2(2)	5(2)	5(2)
C(8)	34(2)	70(3)	53(2)	-4(2)	3(2)	-3(2)
C(9)	34(2)	46(2)	30(2)	1(2)	6(2)	-1(2)
C(10)	34(2)	37(2)	33(2)	-1(2)	8(2)	-1(2)
C(11)	33(2)	41(2)	28(2)	-5(2)	5(2)	-7(2)
C(12)	36(2)	50(3)	36(2)	0(2)	5(2)	-7(2)
C(13)	37(2)	52(3)	46(2)	-3(2)	1(2)	2(2)
C(14)	31(2)	72(3)	47(2)	-16(2)	5(2)	-7(2)
C(15)	48(2)	60(3)	51(2)	-5(2)	15(2)	-19(2)
C(16)	42(2)	42(2)	46(2)	1(2)	4(2)	-5(2)
C(17)	28(2)	46(3)	34(2)	-6(2)	2(2)	0(2)
C(18)	38(2)	51(3)	37(2)	-2(2)	9(2)	-3(2)
C(19)	44(2)	81(4)	92(3)	9(3)	6(2)	-19(3)
C(20)	164(6)	69(4)	89(4)	6(3)	93(4)	6(4)
C(21)	60(3)	56(3)	61(3)	7(2)	-5(2)	-1(2)
Si(1)	48(1)	46(1)	48(1)	4(1)	17(1)	-4(1)

Supplementary Table 11. Hydrogen coordinates (x 10^4) and isotropic displacement parameters (A^2 x 10^3) for t_a.

	x	y	z	U(eq)
H(2A)	8683	3517	1086	69
H(2)	4116	3336	1445	46
H(3)	6430	3216	1841	46
H(5)	6620	9642	831	45
H(6)	4309	9736	414	46

**H(8A)**	**1976**	**3093**	**585**	**81**
**H(8B)**	**728**	**4829**	**444**	**81**
**H(8C)**	**1686**	**4579**	**1313**	**81**
**H(9)**	**8626**	**7264**	**1191**	**44**
**H(10)**	**8612**	**8978**	**2449**	**41**
**H(12)**	**10697**	**4100**	**3213**	**49**
**H(13)**	**13003**	**3984**	**3409**	**56**
**H(14)**	**14117**	**7015**	**2929**	**61**
**H(15)**	**12931**	**10227**	**2263**	**63**
**H(16)**	**10601**	**10361**	**2065**	**53**
**H(19A)**	**5520**	**230**	**3964**	**111**
**H(19B)**	**6249**	**187**	**3245**	**111**
**H(19C)**	**5255**	**2293**	**3313**	**111**
**H(20A)**	**6353**	**3837**	**5318**	**145**
**H(20B)**	**6122**	**5994**	**4702**	**145**
**H(20C)**	**7528**	**5659**	**5313**	**145**
**H(21A)**	**8372**	**-174**	**5076**	**93**
**H(21B)**	**9443**	**1812**	**5022**	**93**
**H(21C)**	**8977**	**-26**	**4308**	**93**

Supplementary Table 12. Torsion angles [deg] for t_a.

**C(6)-C(1)-C(2)-C(3)**	**-1.4(5)**
**C(7)-C(1)-C(2)-C(3)**	**175.2(3)**
**C(1)-C(2)-C(3)-C(4)**	**0.3(5)**
**C(2)-C(3)-C(4)-C(5)**	**1.3(5)**
**C(2)-C(3)-C(4)-C(9)**	**-177.1(3)**
**C(3)-C(4)-C(5)-C(6)**	**-1.9(5)**
**C(9)-C(4)-C(5)-C(6)**	**176.5(3)**
**C(2)-C(1)-C(6)-C(5)**	**0.7(5)**
**C(7)-C(1)-C(6)-C(5)**	**-175.9(3)**

**C(4)-C(5)-C(6)-C(1)**	**0.9(5)**
**C(6)-C(1)-C(7)-O(1)**	**1.2(5)**
**C(2)-C(1)-C(7)-O(1)**	**-175.3(3)**
**C(6)-C(1)-C(7)-C(8)**	**-179.6(3)**
**C(2)-C(1)-C(7)-C(8)**	**3.9(5)**
**C(3)-C(4)-C(9)-O(2)**	**32.4(4)**
**C(5)-C(4)-C(9)-O(2)**	**-146.0(3)**
**C(3)-C(4)-C(9)-C(10)**	**-88.3(4)**
**C(5)-C(4)-C(9)-C(10)**	**93.3(4)**
**O(2)-C(9)-C(10)-C(17)**	**-65.5(4)**
**C(4)-C(9)-C(10)-C(17)**	**57.3(4)**
**O(2)-C(9)-C(10)-C(11)**	**59.2(4)**
**C(4)-C(9)-C(10)-C(11)**	**-178.1(3)**
**C(17)-C(10)-C(11)-C(12)**	**30.1(5)**
**C(9)-C(10)-C(11)-C(12)**	**-94.2(4)**
**C(17)-C(10)-C(11)-C(16)**	**-153.4(3)**
**C(9)-C(10)-C(11)-C(16)**	**82.3(4)**
**C(16)-C(11)-C(12)-C(13)**	**-0.1(5)**
**C(10)-C(11)-C(12)-C(13)**	**176.3(3)**
**C(11)-C(12)-C(13)-C(14)**	**-0.2(6)**
**C(12)-C(13)-C(14)-C(15)**	**0.5(6)**
**C(13)-C(14)-C(15)-C(16)**	**-0.4(6)**
**C(12)-C(11)-C(16)-C(15)**	**0.2(5)**
**C(10)-C(11)-C(16)-C(15)**	**-176.4(3)**
**C(14)-C(15)-C(16)-C(11)**	**0.1(6)**

Symmetry transformations used to generate equivalent atoms:

Supplementary Table 13. Hydrogen bonds for t_a [A and deg.].

_

D-H...A	d(D-H)	d(H...A)	d(D...A)	<(DHA)

■ NMR Spectra

(¹H NMR, 400 MHz, CDCl₃)

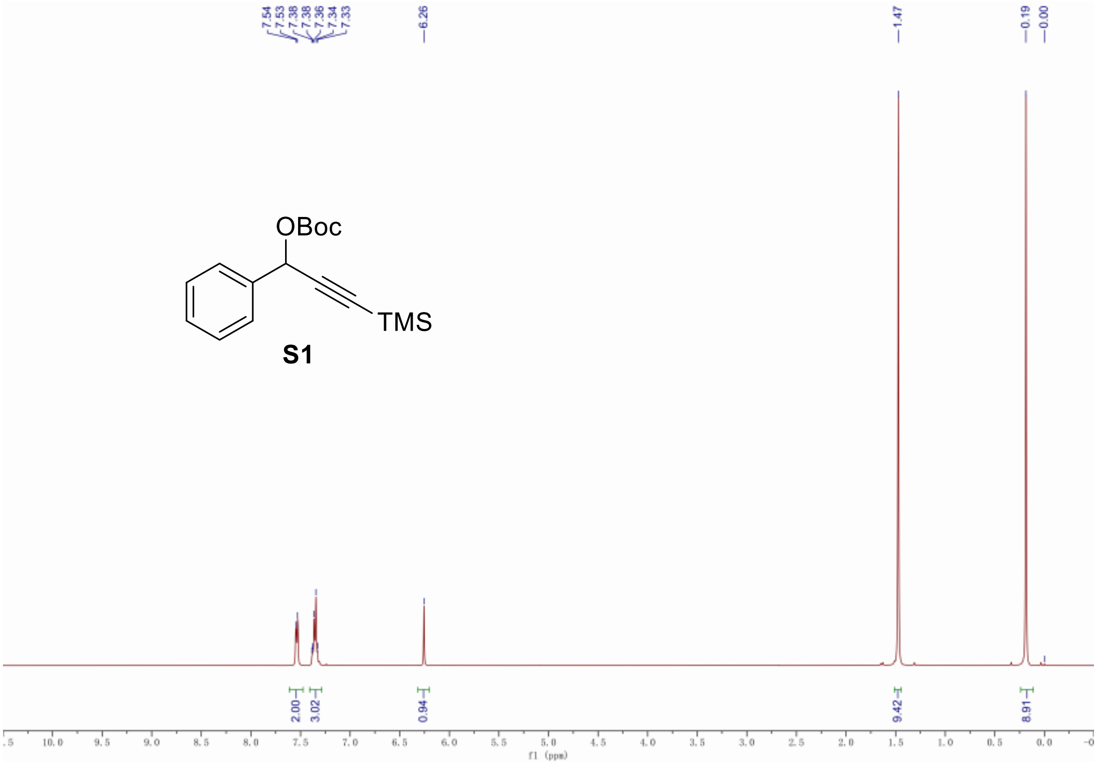

(^{13}C NMR, 100 MHz, $CDCl_3$)

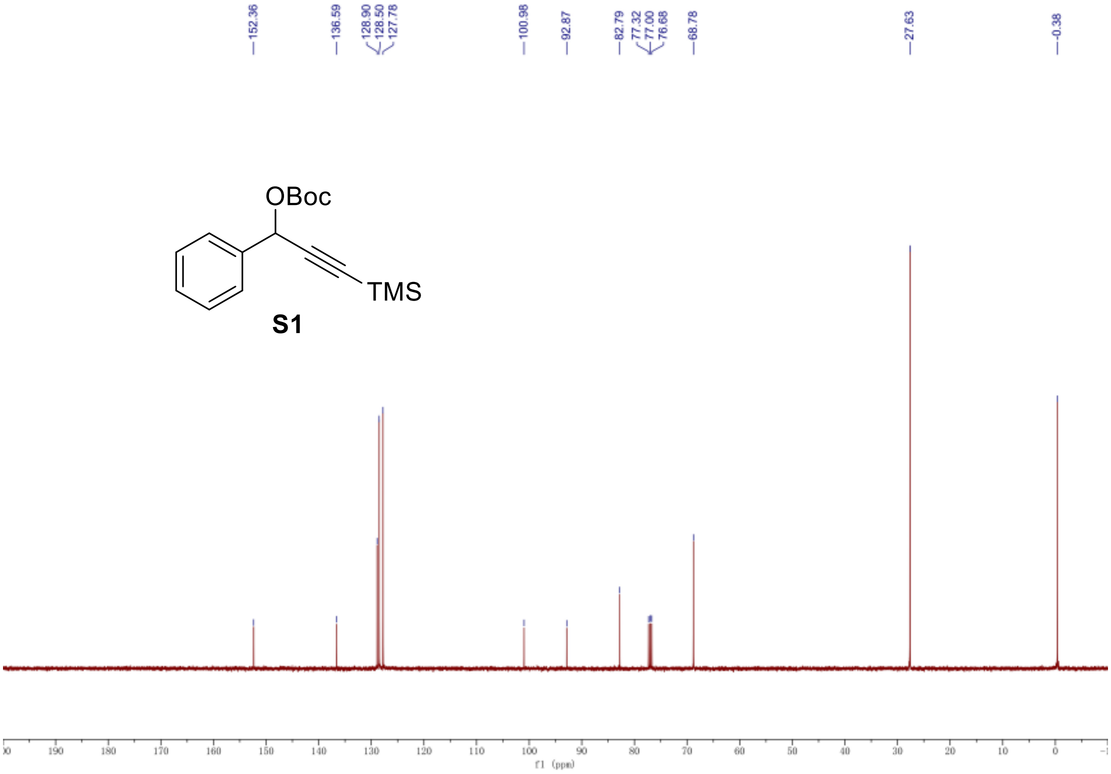

(¹H NMR, 400 MHz, $CDCl_3$)

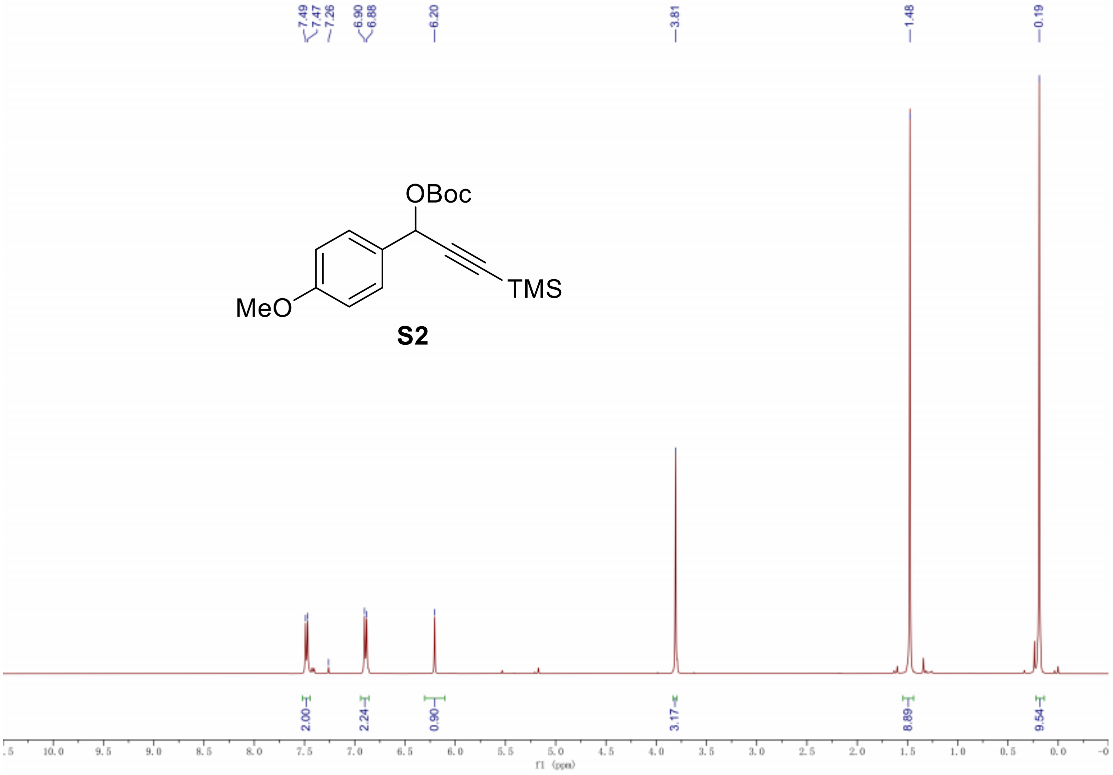

(¹³C NMR, 100 MHz, $CDCl_3$)

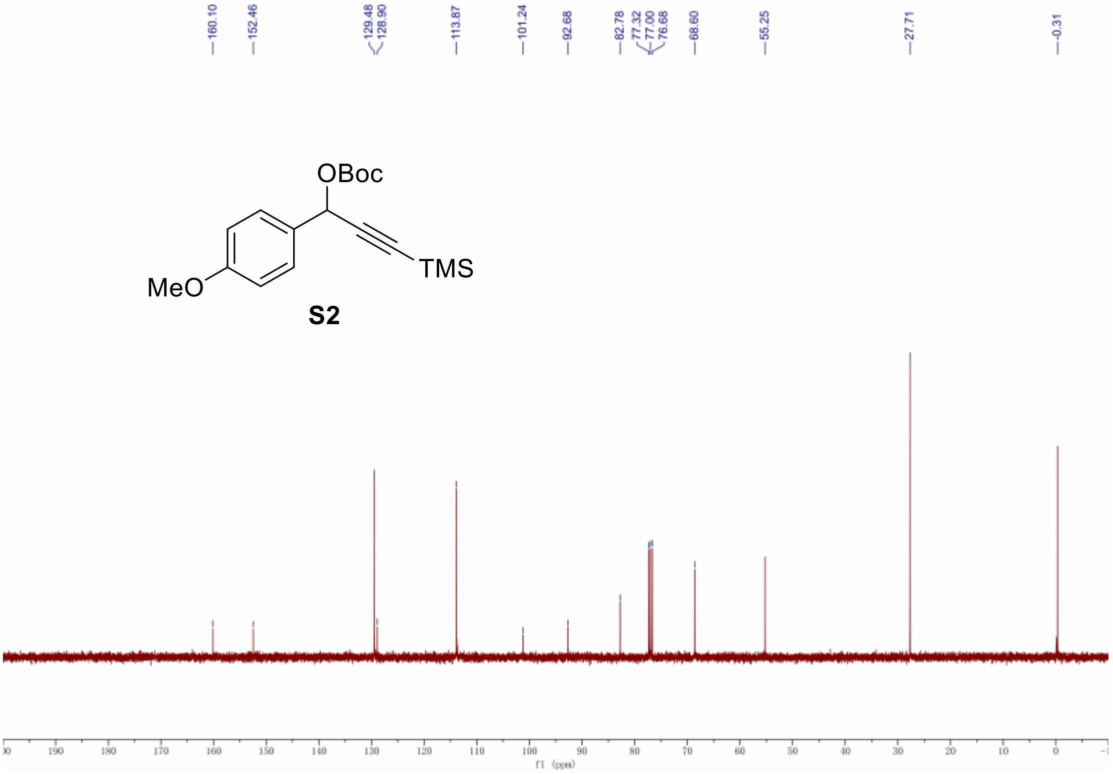

(¹H NMR, 400 MHz, $CDCl_3$)

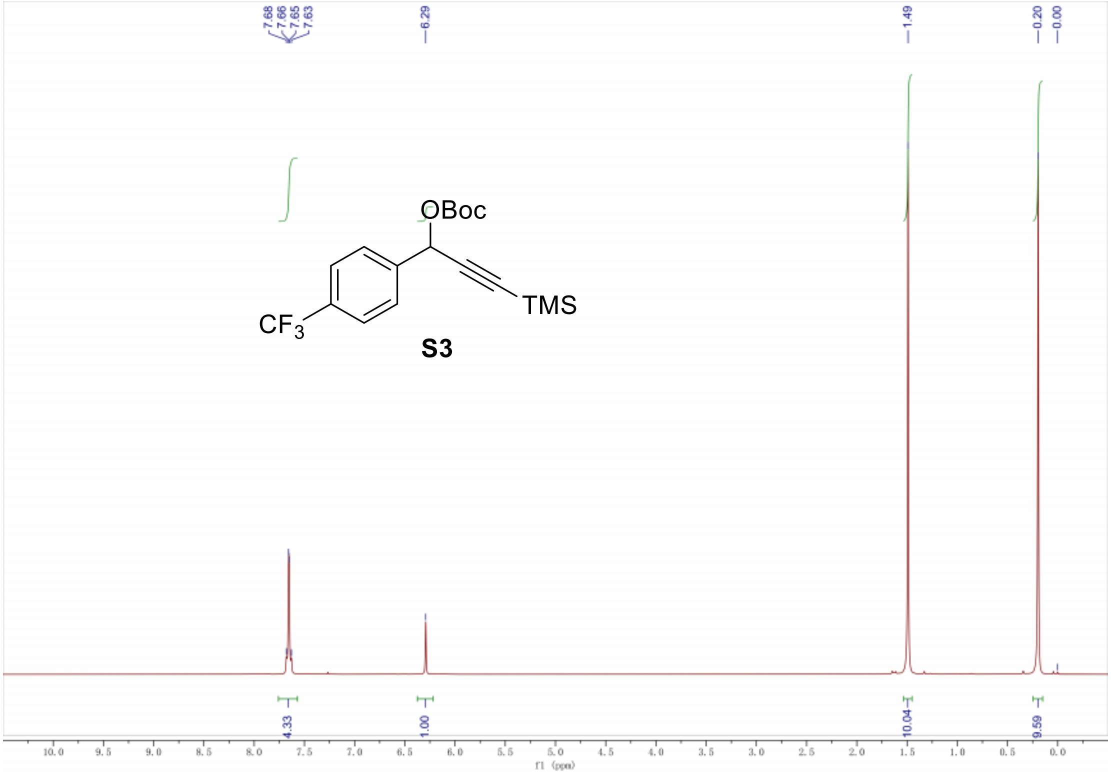

(¹³C NMR, 100 MHz, $CDCl_3$)

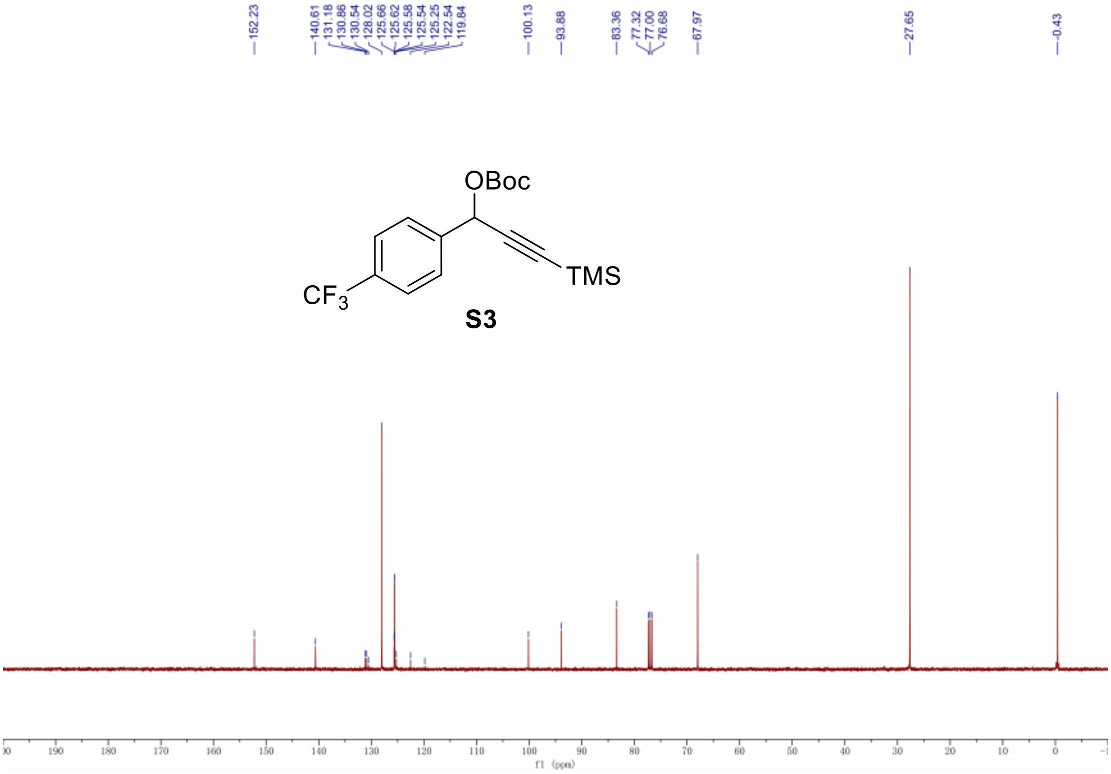

(^{19}F NMR, 376 MHz, $CDCl_3$)

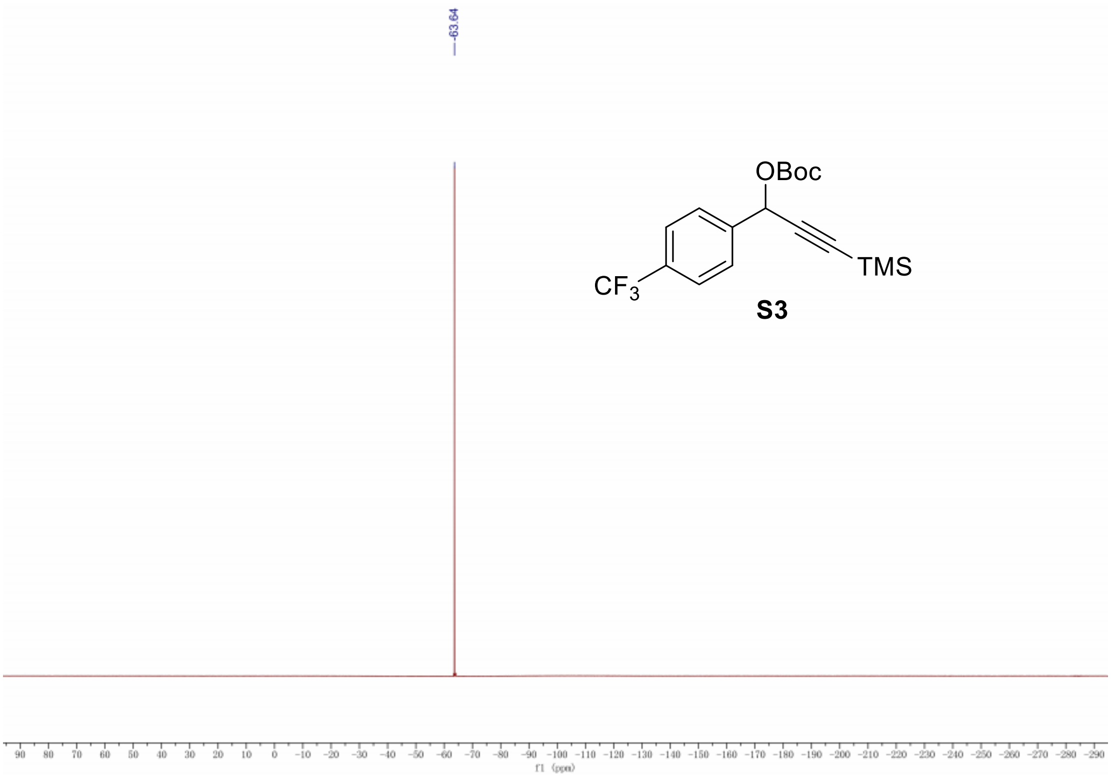

(^{1}H NMR, 400 MHz, $CDCl_3$)

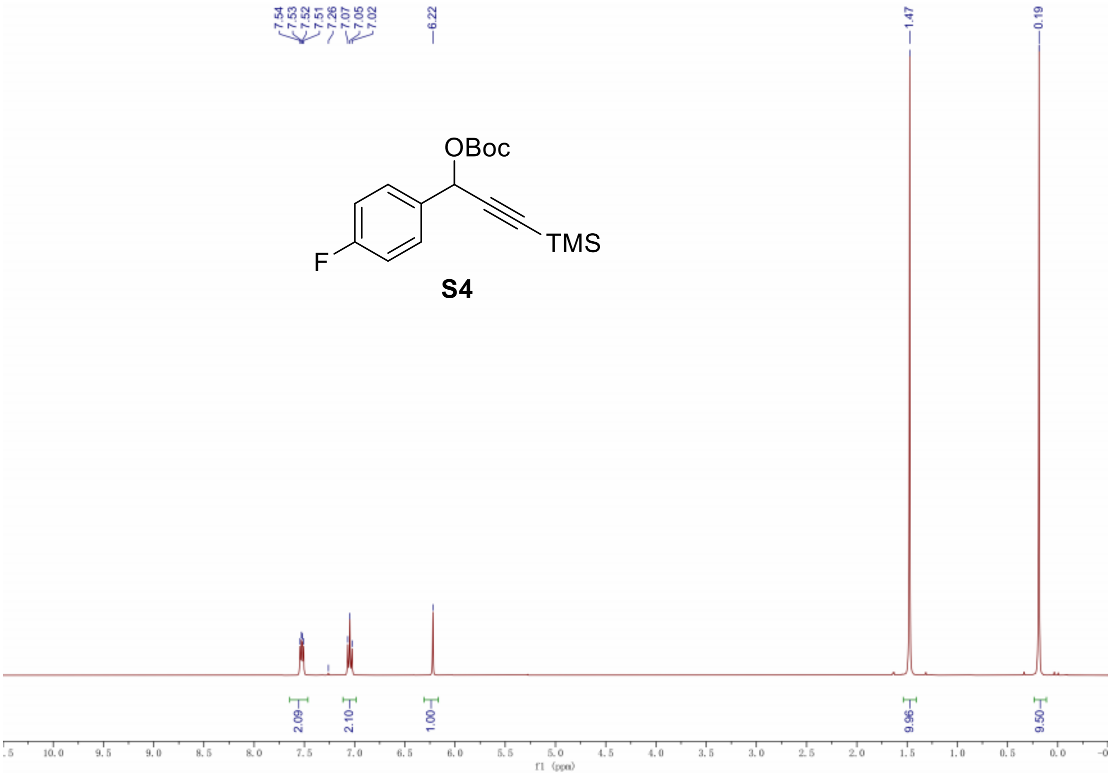

(^{13}C NMR, 100 MHz, CDCl$_3$)

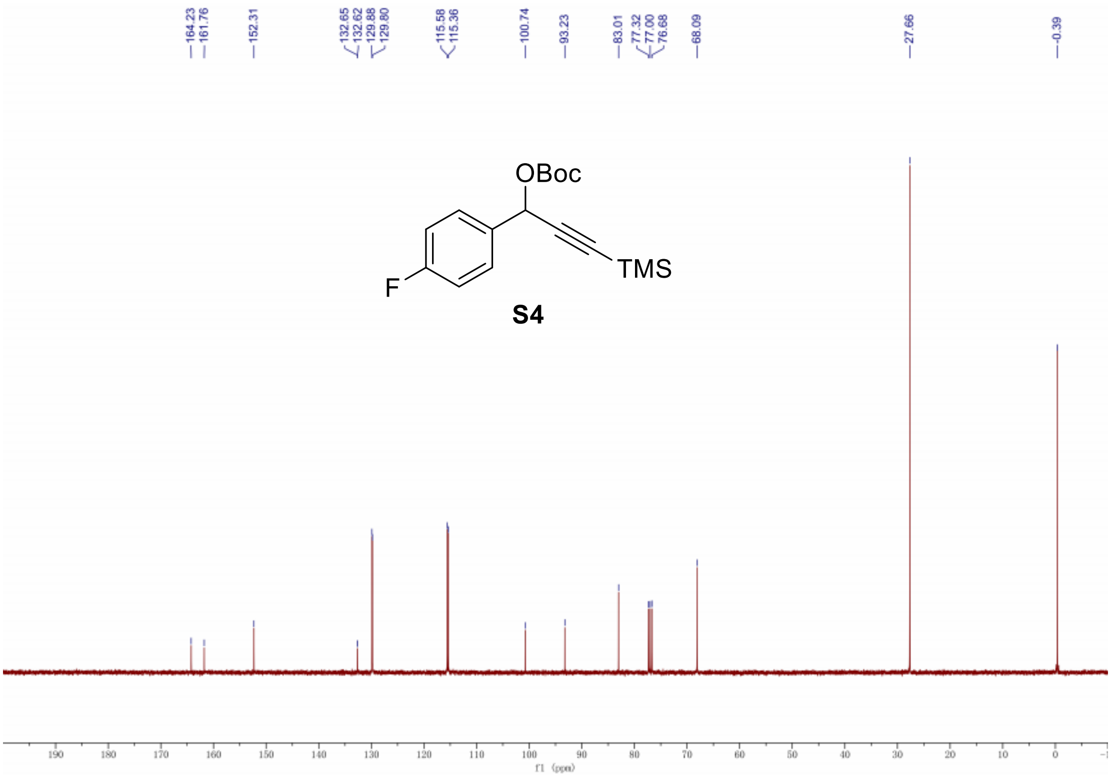

(^{19}F NMR, 376 MHz, CDCl$_3$)

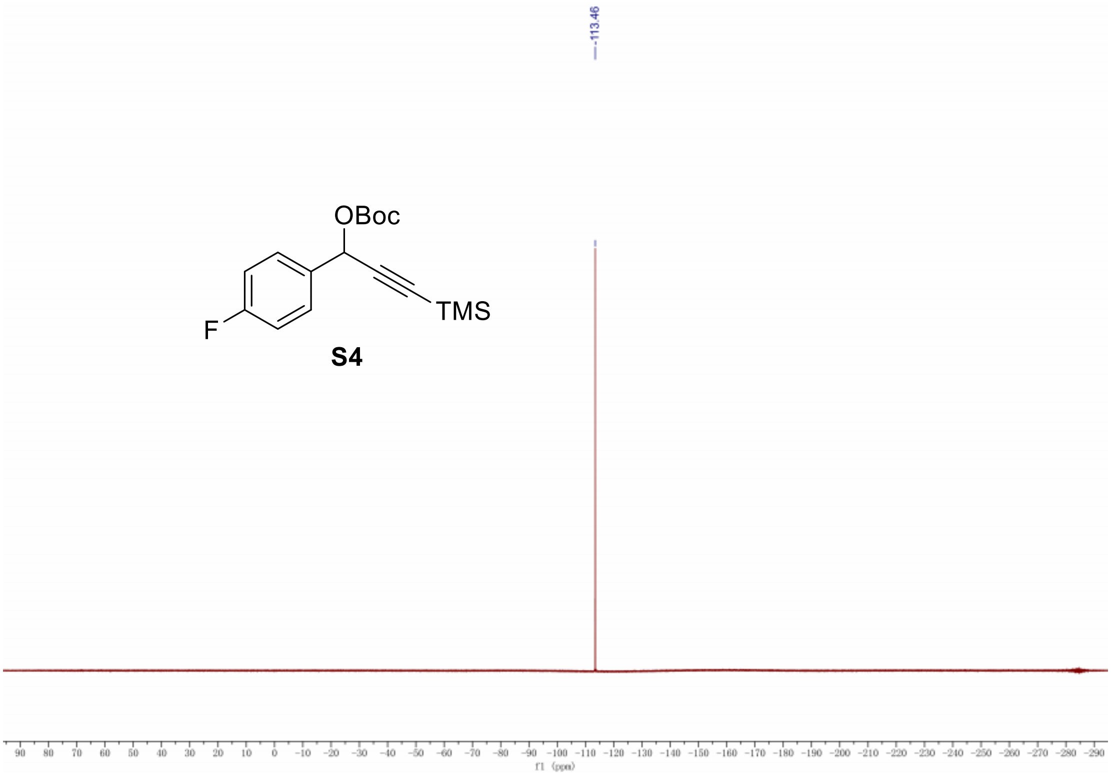

（^{1}H NMR, 400 MHz, $CDCl_3$）

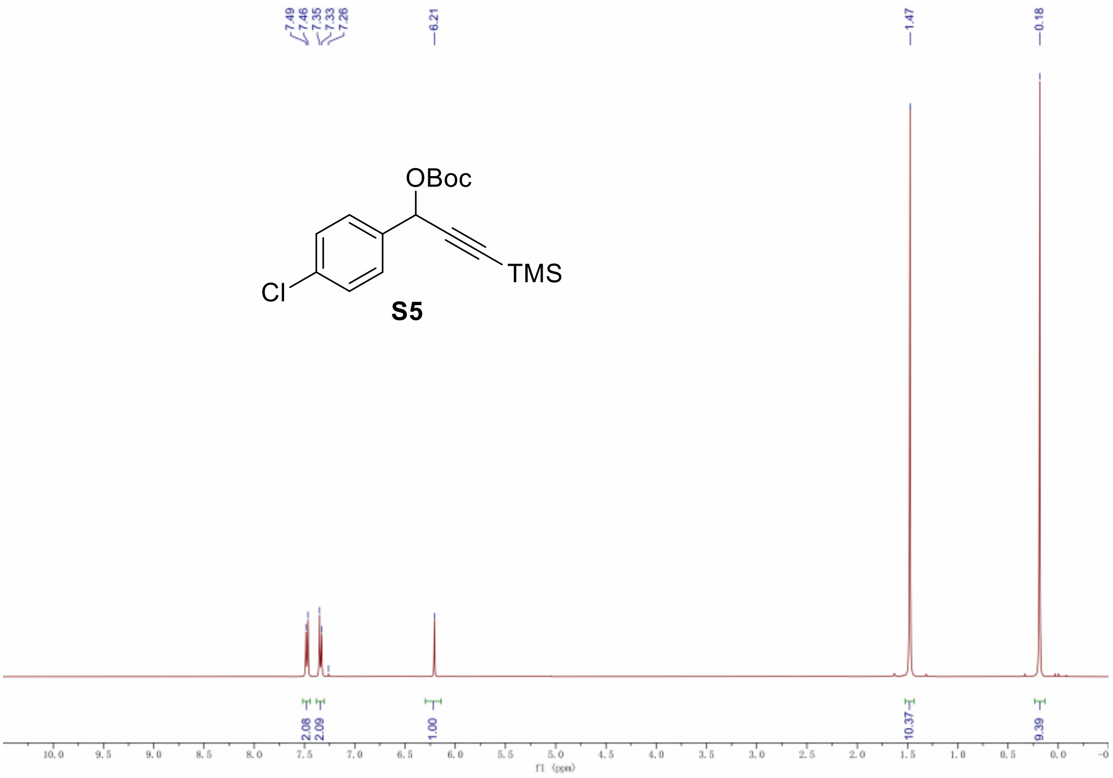

(^{13}C NMR, 100 MHz, $CDCl_3$)

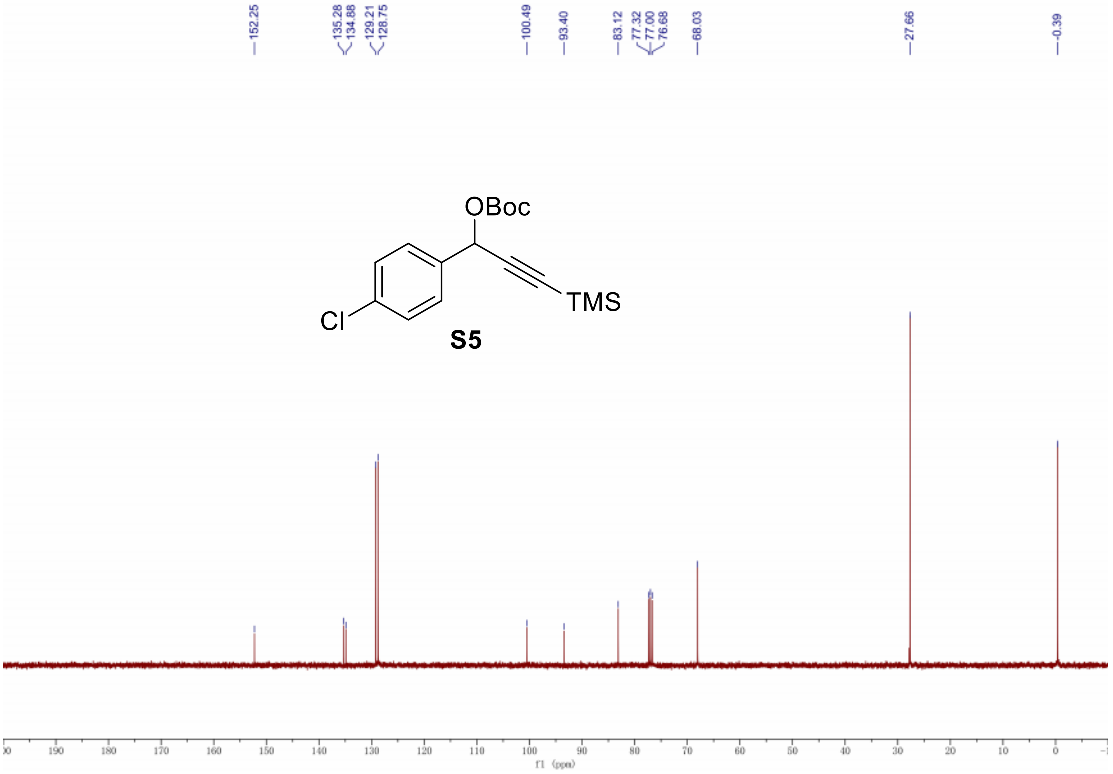

(¹H NMR, 400 MHz, $CDCl_3$)

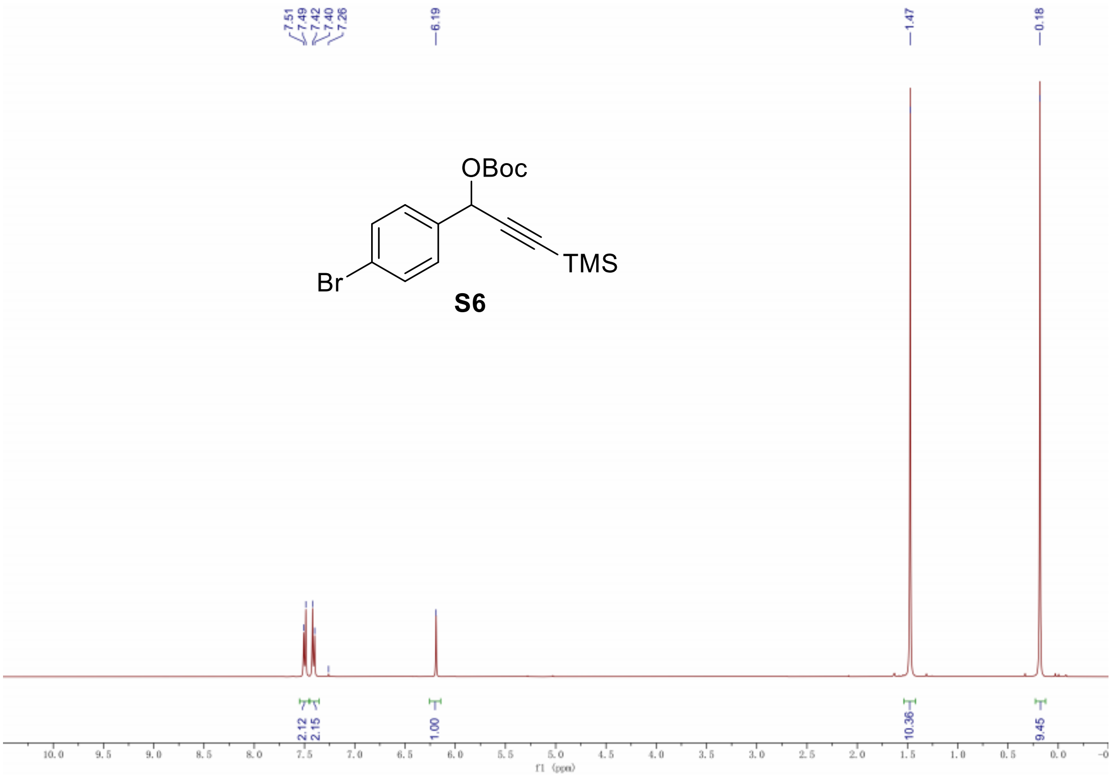

(¹³C NMR, 100 MHz, $CDCl_3$)

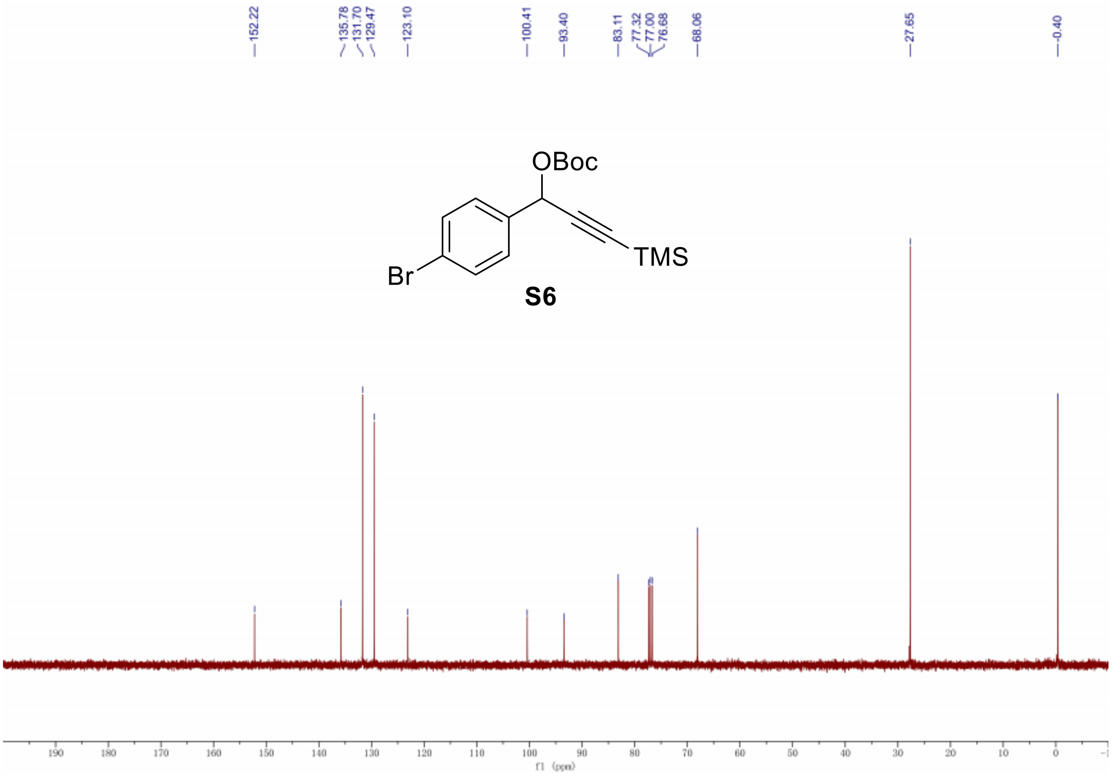

（^{1}H NMR, 400 MHz, CDCl$_3$）

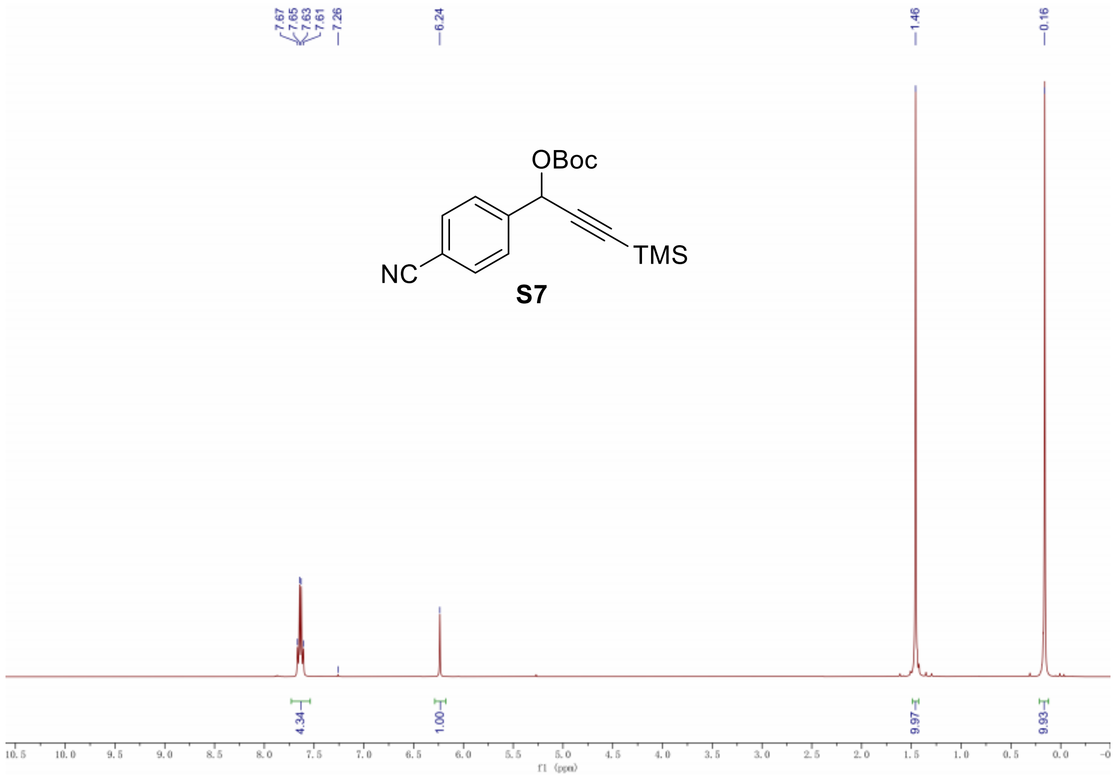

(^{13}C NMR, 100 MHz, CDCl$_3$)

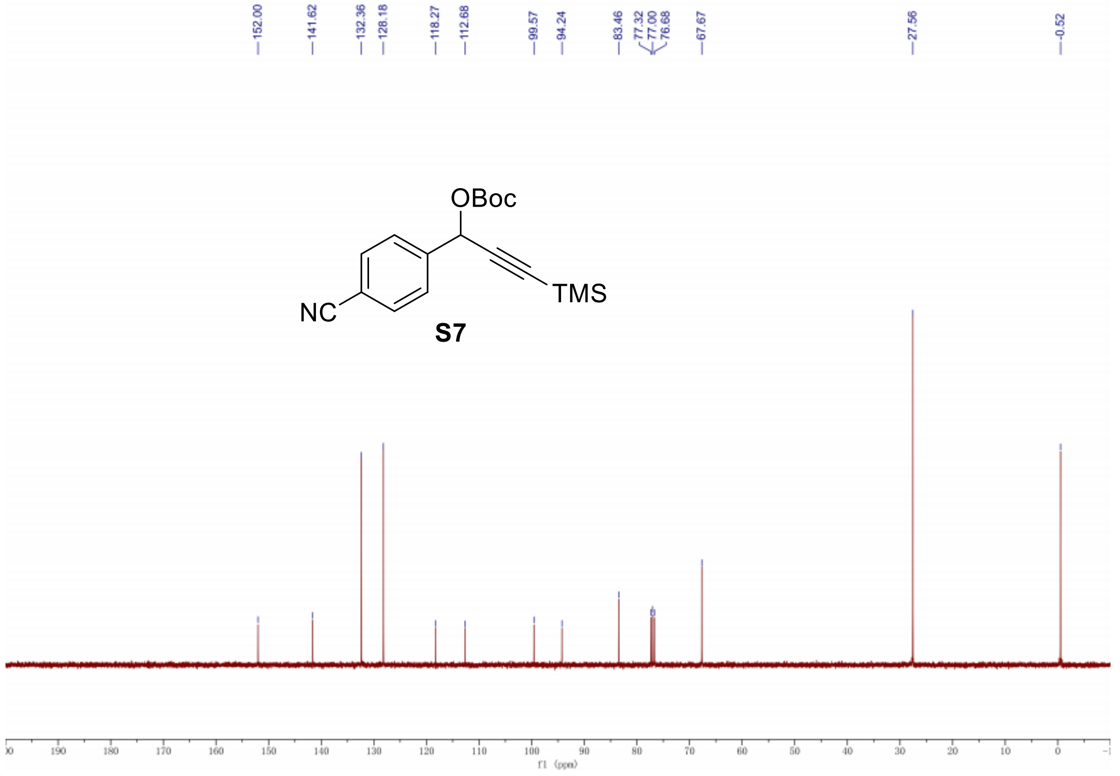

(¹H NMR, 400 MHz, $CDCl_3$)

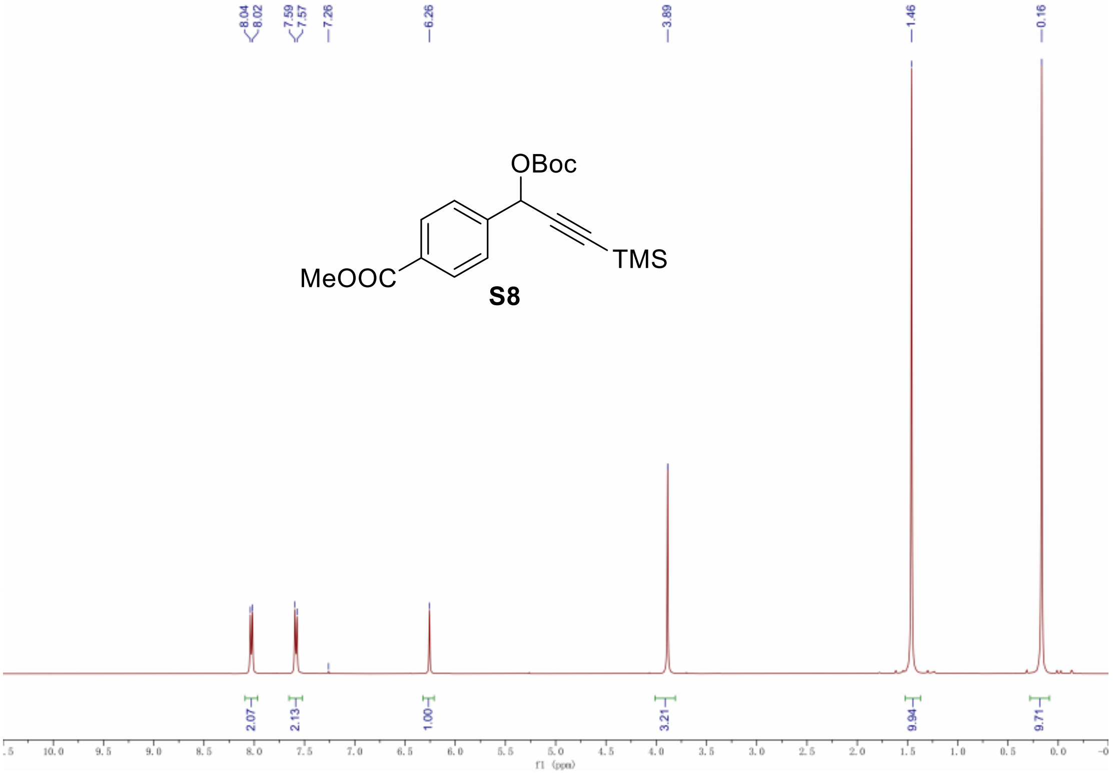

(^{13}C NMR, 100 MHz, $CDCl_3$)

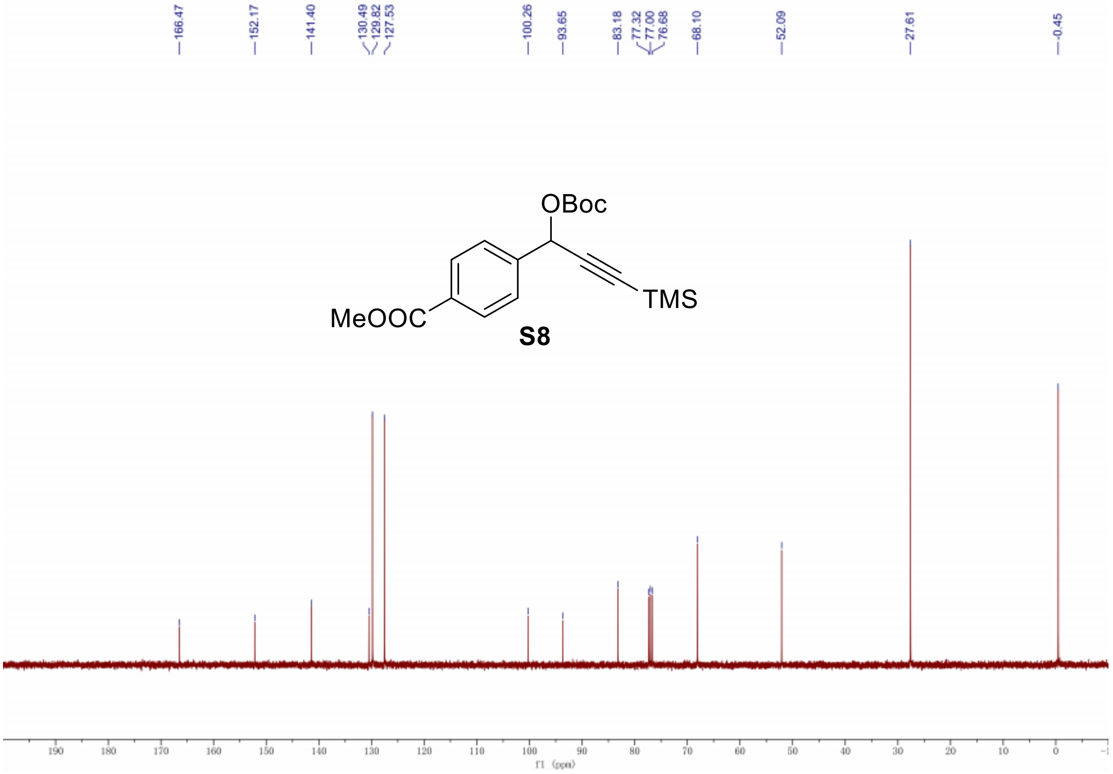

（¹H NMR, 400 MHz, CDCl₃）

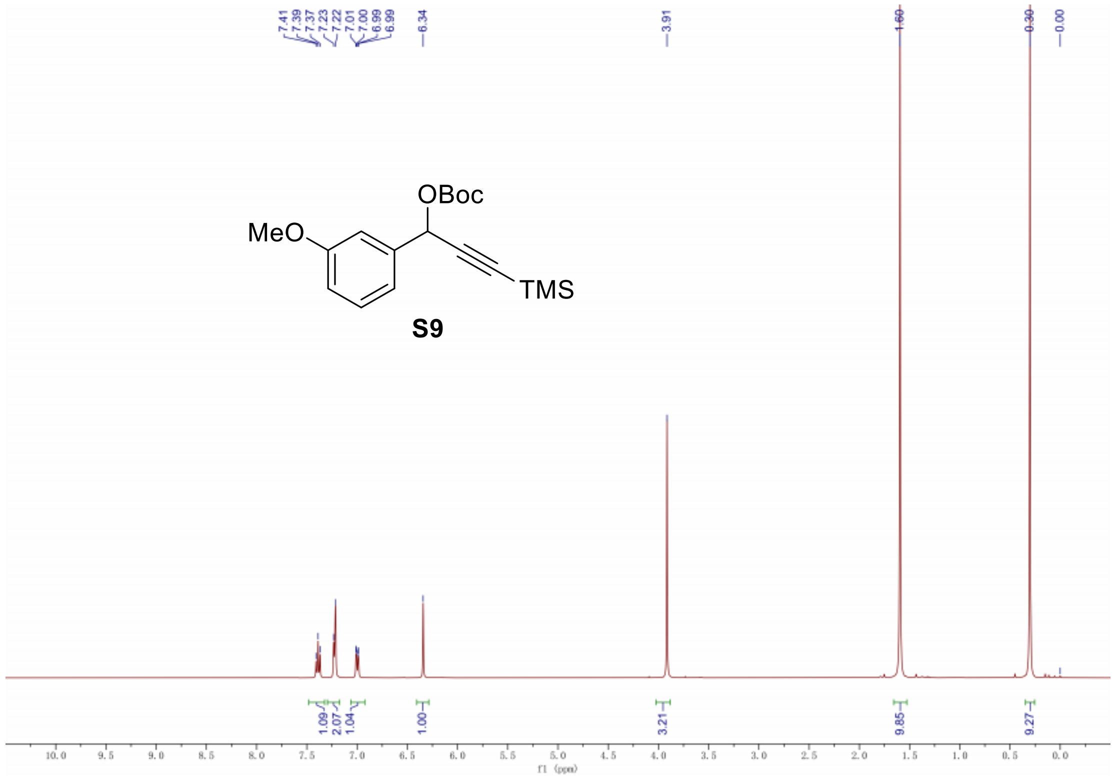

(^{13}C NMR, 100 MHz, CDCl₃)

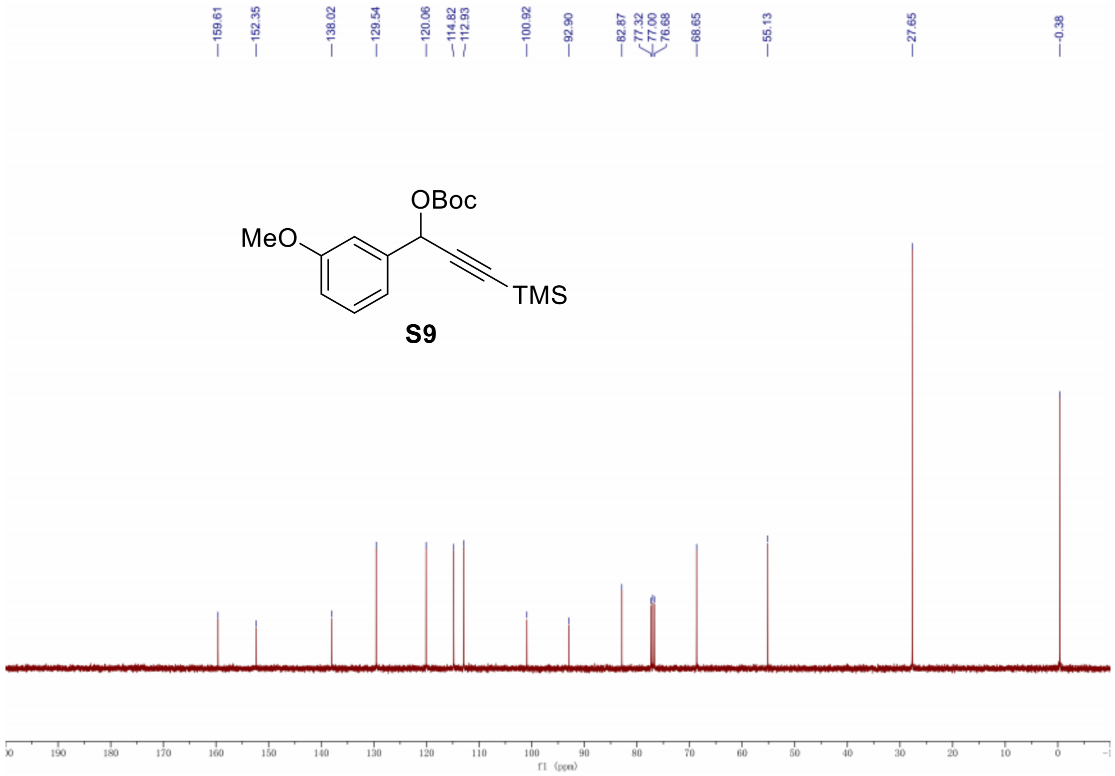

(^{1}H NMR, 400 MHz, $CDCl_3$)

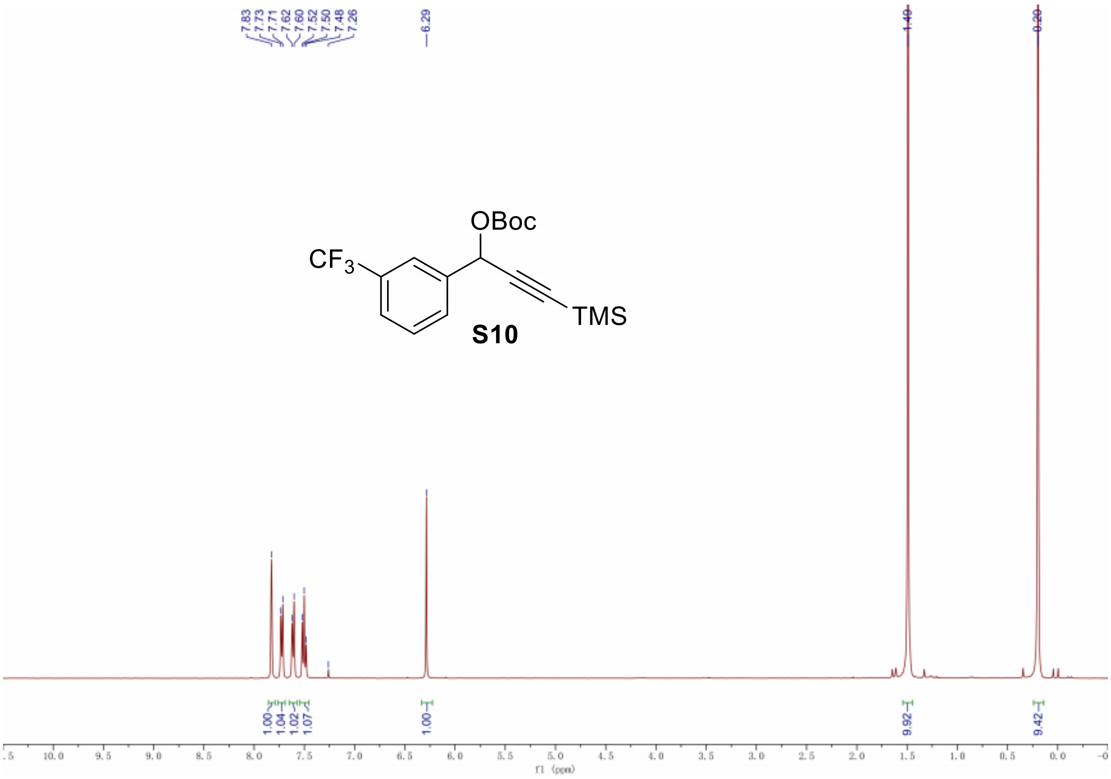

(^{13}C NMR, 100 MHz, $CDCl_3$)

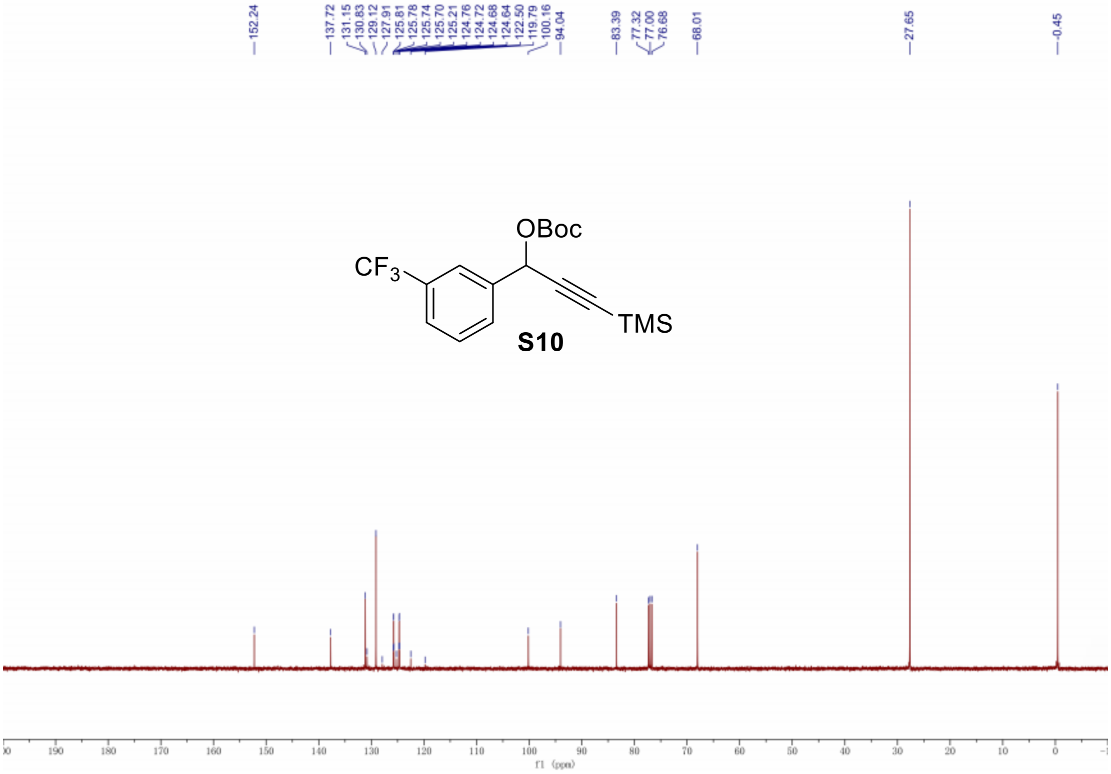

(^{19}F NMR, 376 MHz, CDCl$_3$)

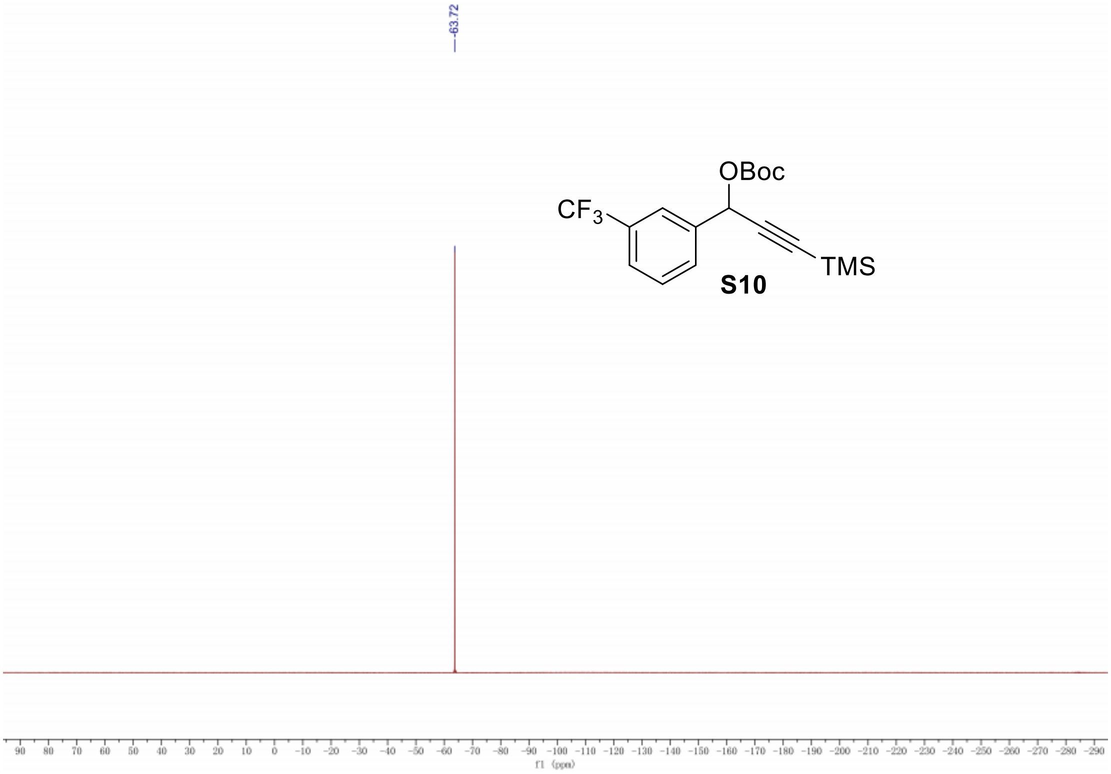

(^{1}H NMR, 400 MHz, CDCl$_3$)

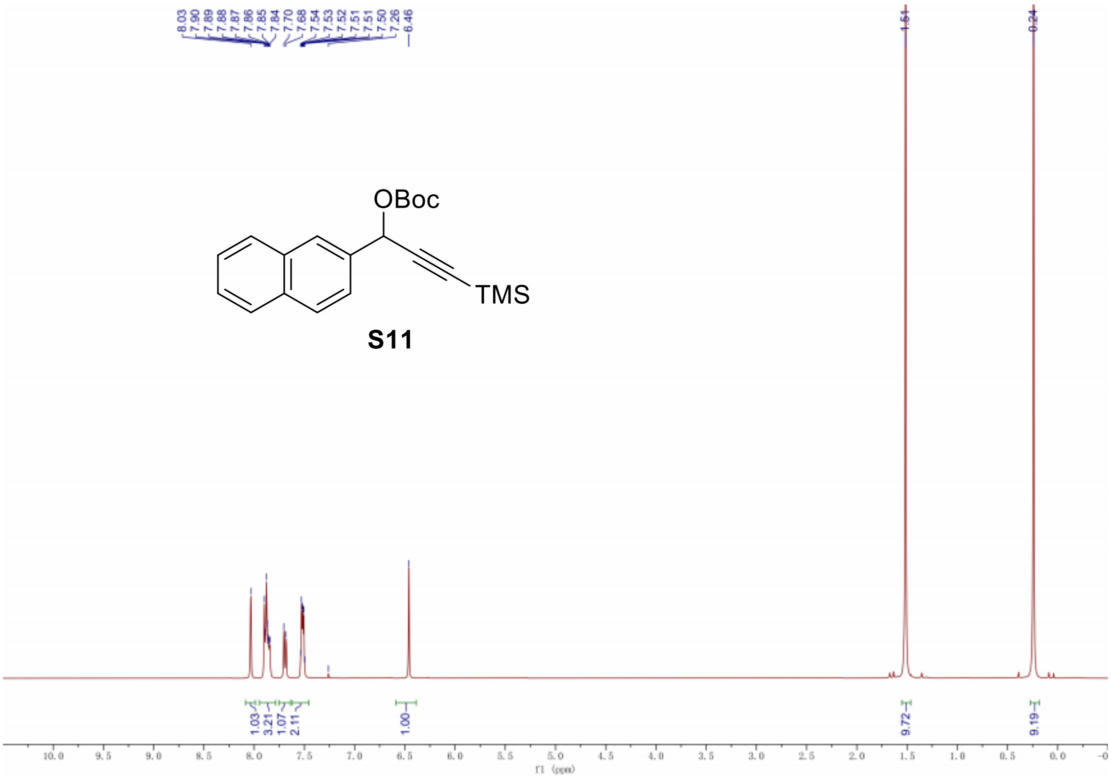

(^{13}C NMR, 100 MHz, $CDCl_3$)

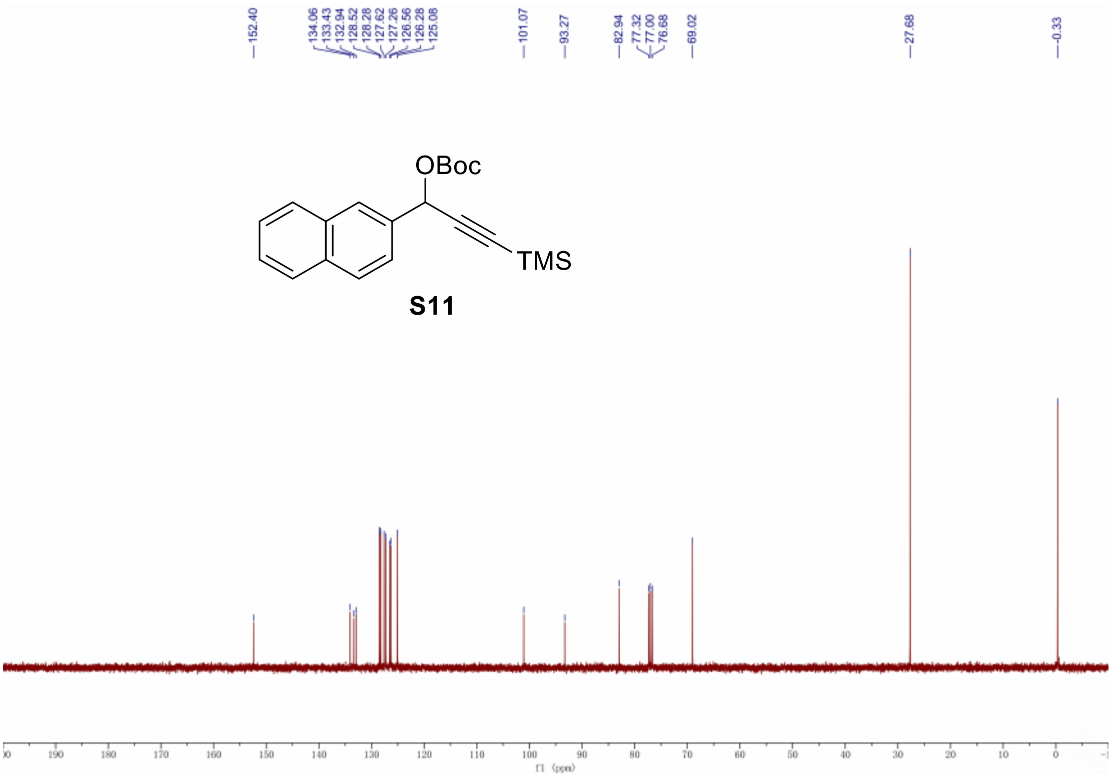

(^{1}H NMR, 400 MHz, $CDCl_3$)

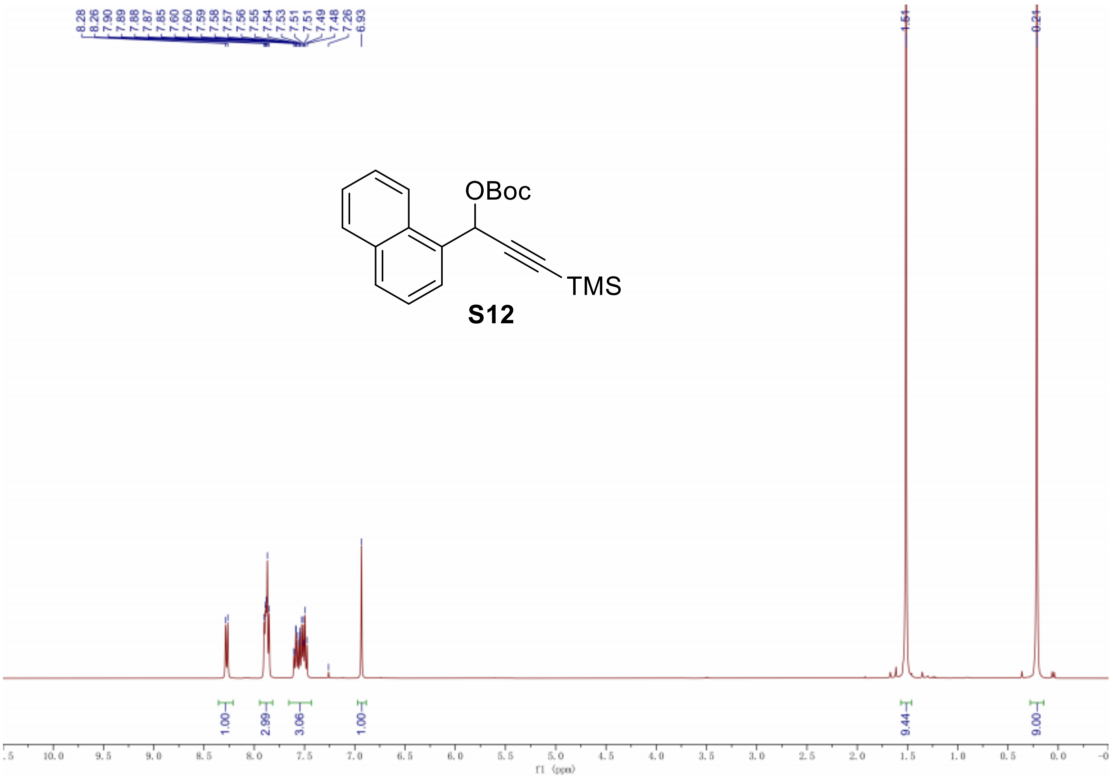

(^{13}C NMR, 100 MHz, CDCl$_3$)

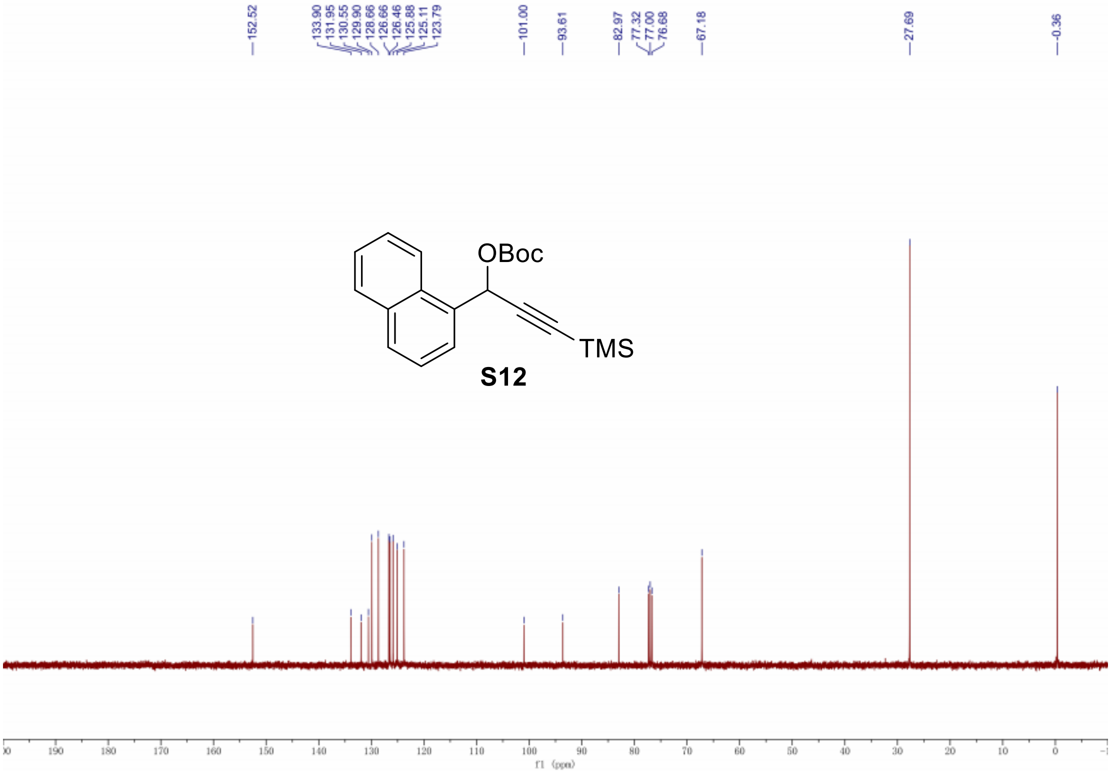

(^{1}H NMR, 400 MHz, CDCl$_3$)

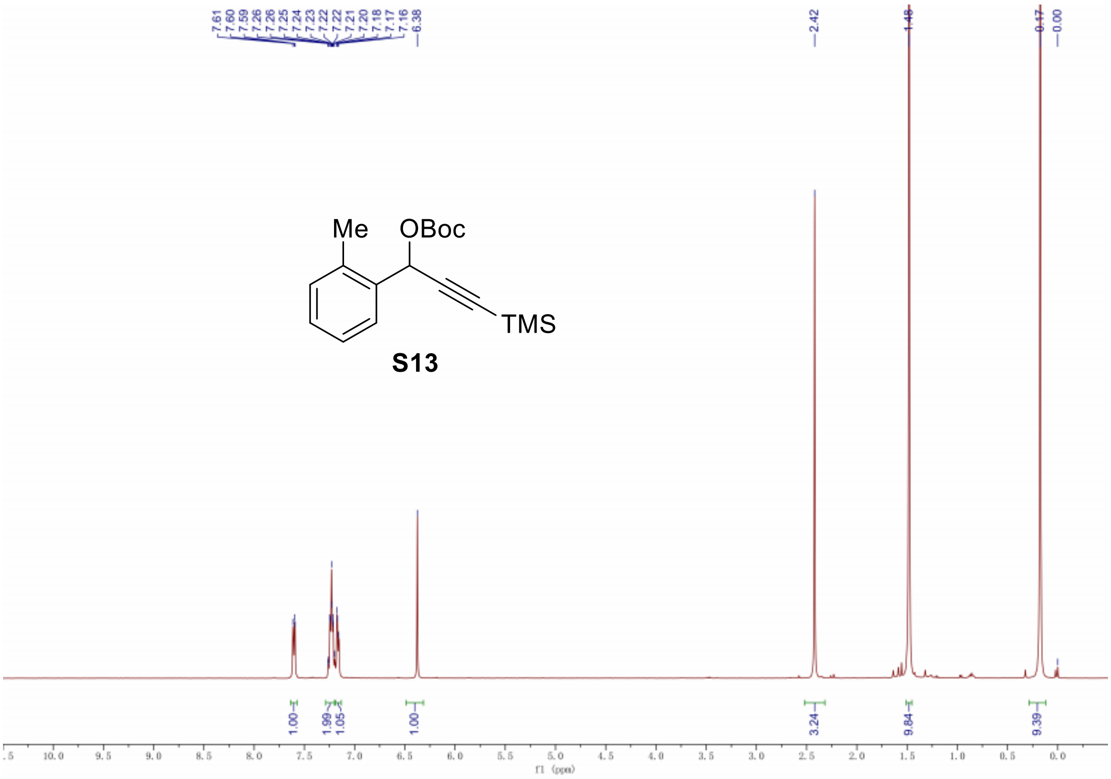

(^{13}C NMR, 100 MHz, CDCl$_3$)

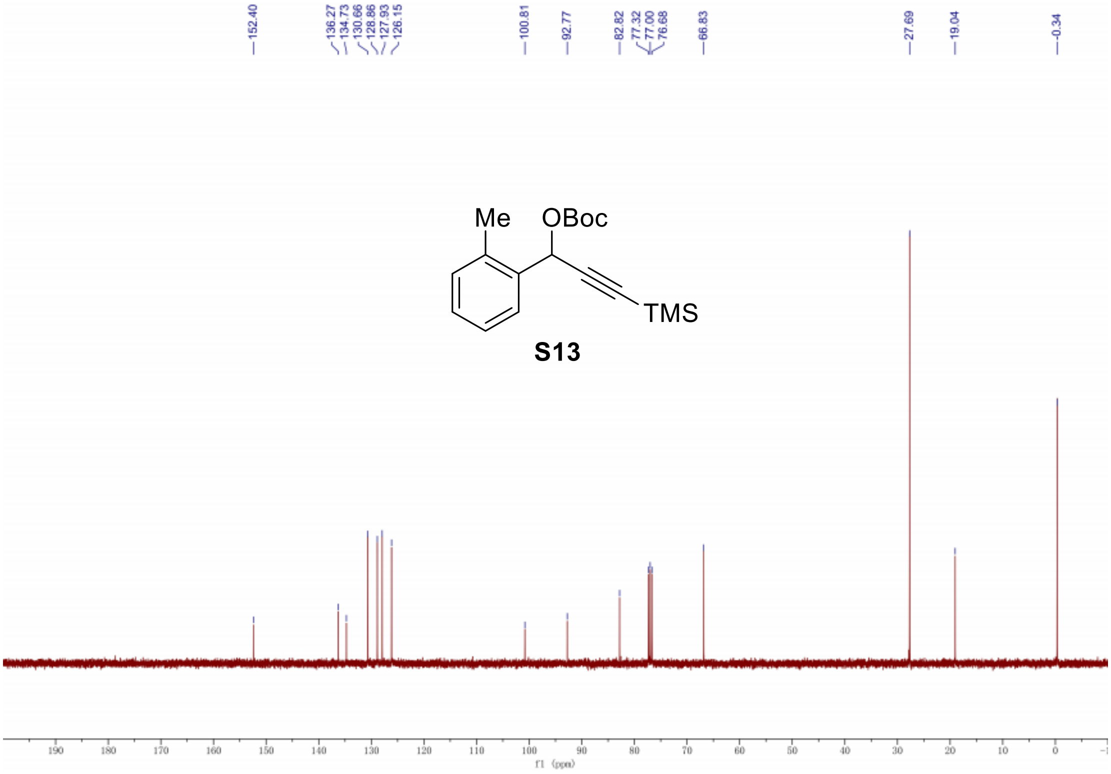

（^{1}H NMR, 400 MHz, CDCl$_3$）

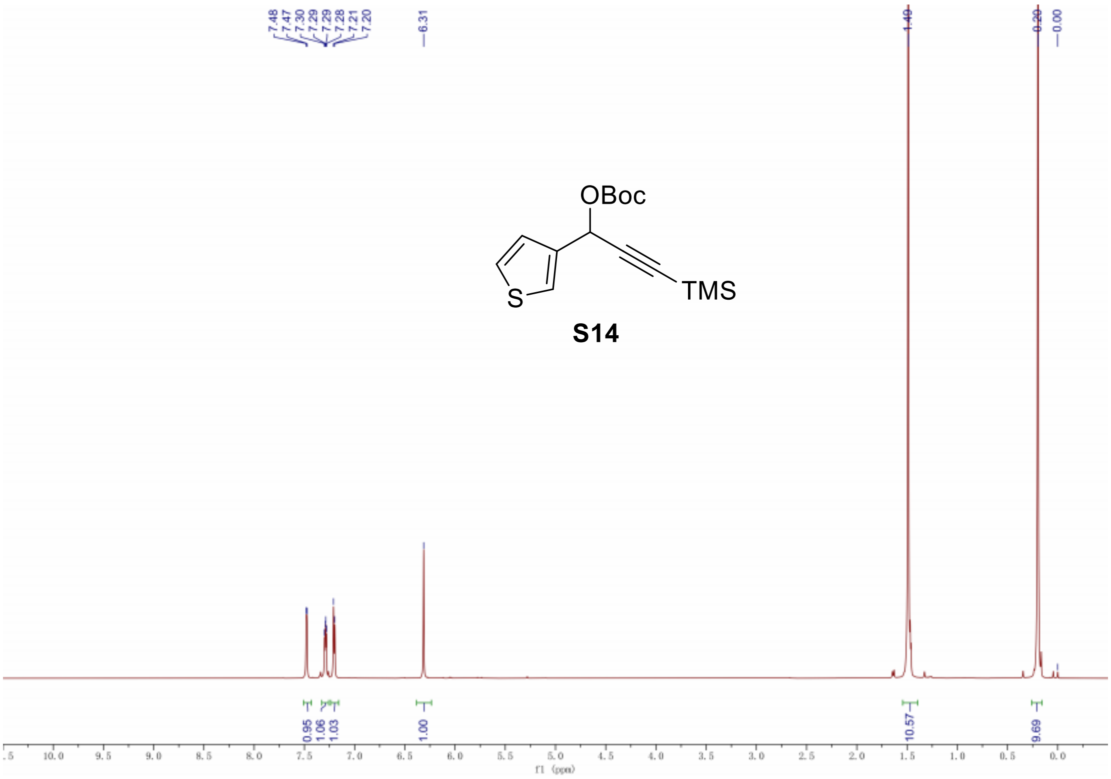

(^{13}C NMR, 100 MHz, CDCl$_3$)

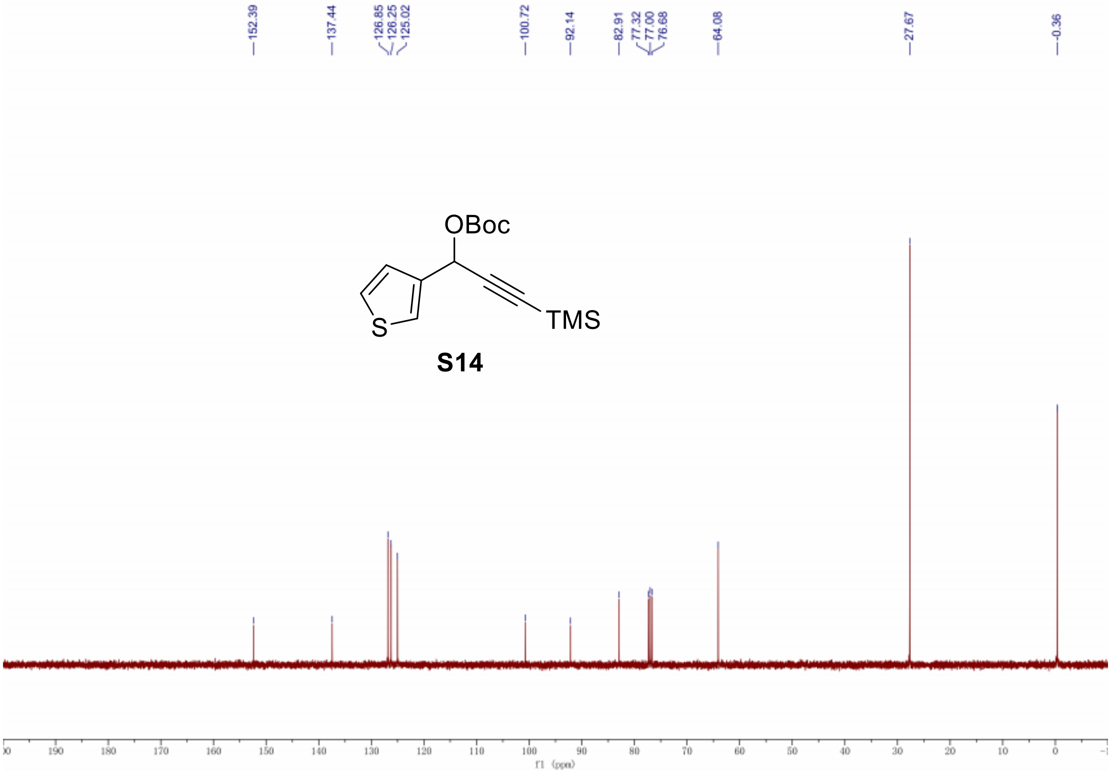

(^{1}H NMR, 400 MHz, CDCl$_3$)

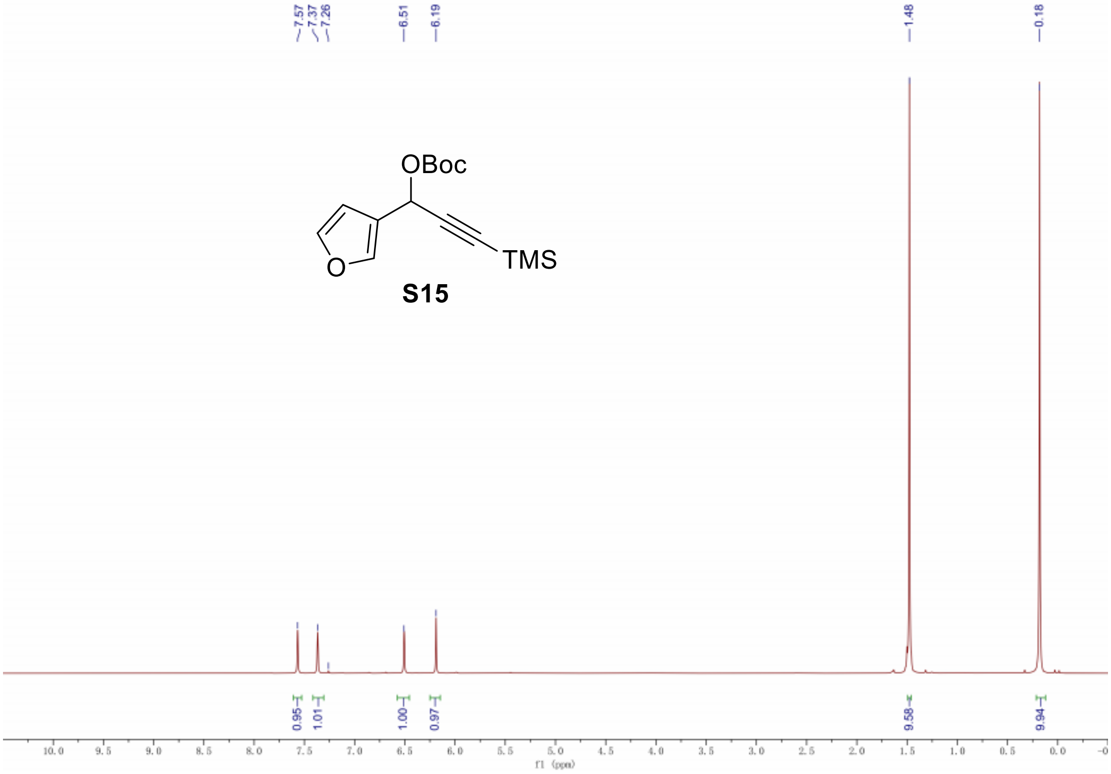

(^{13}C NMR, 100 MHz, $CDCl_3$)

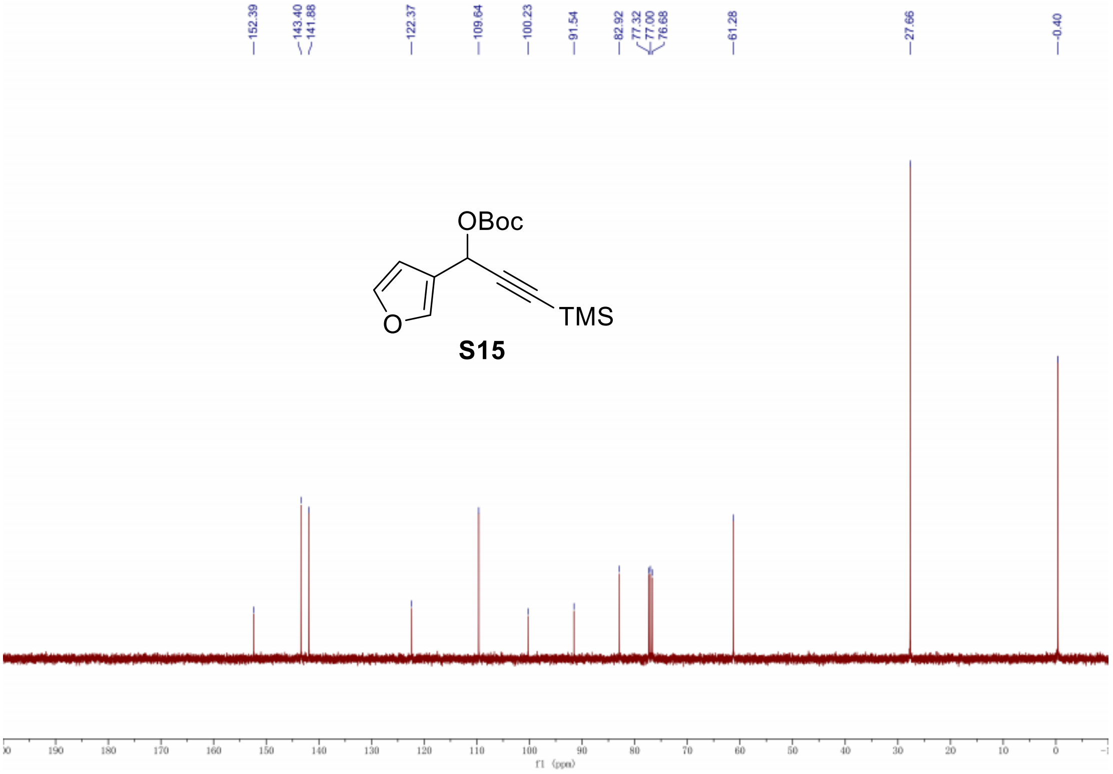

(^{1}H NMR, 400 MHz, $CDCl_3$)

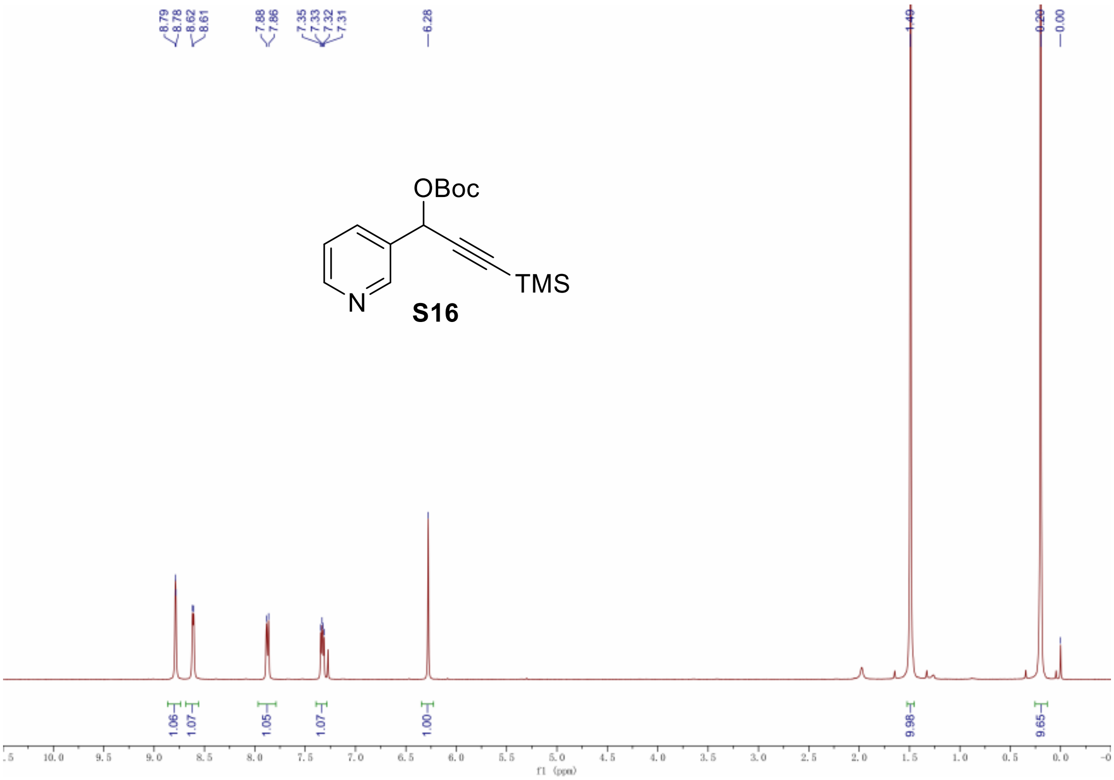

(^{13}C NMR, 100 MHz, CDCl$_3$)

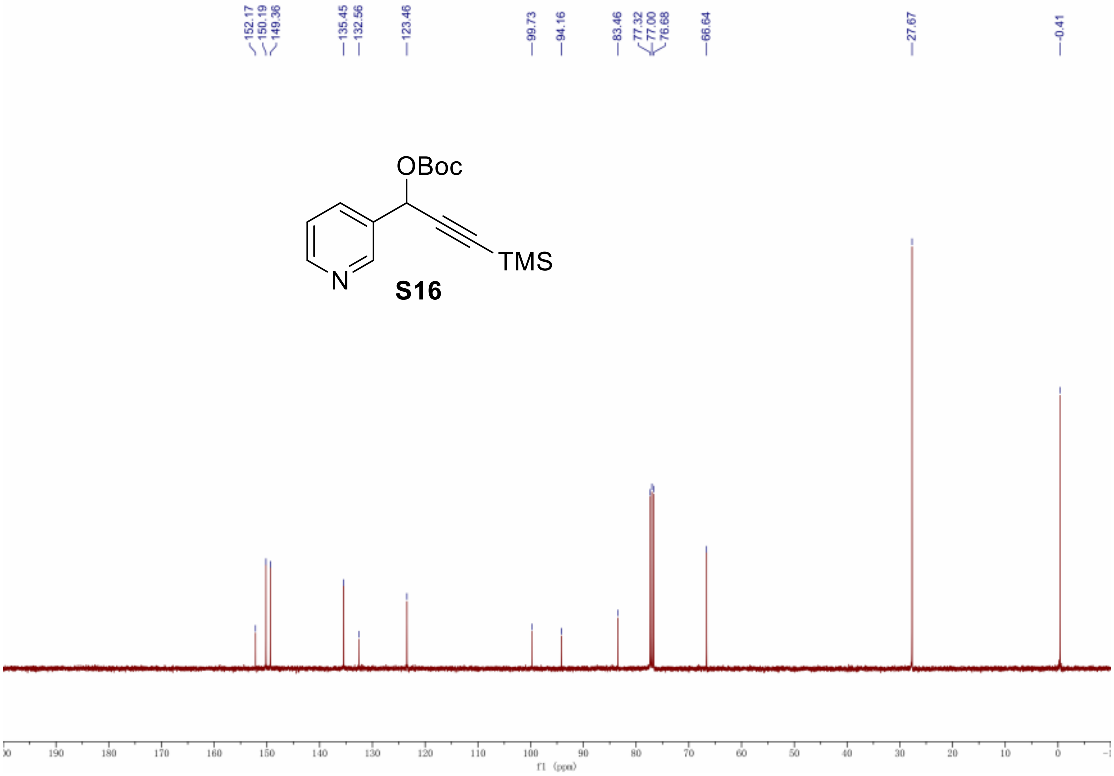

(¹H NMR, 400 MHz, CDCl$_3$)

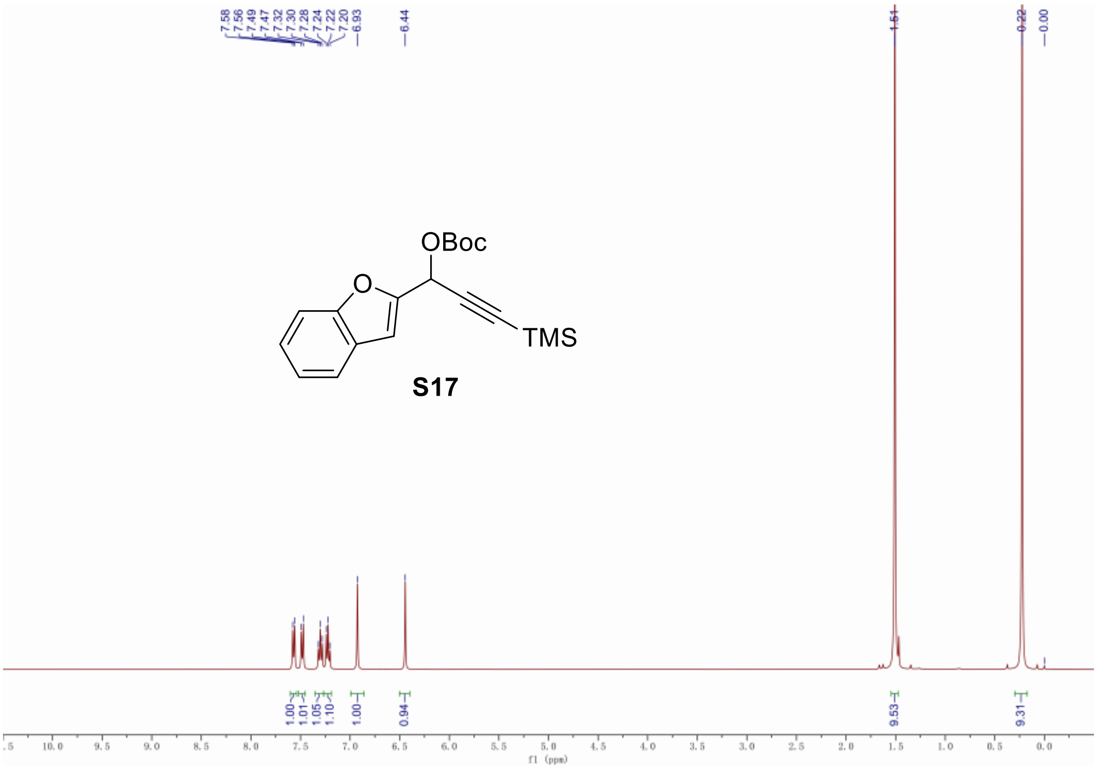

(^{13}C NMR, 100 MHz, $CDCl_3$)

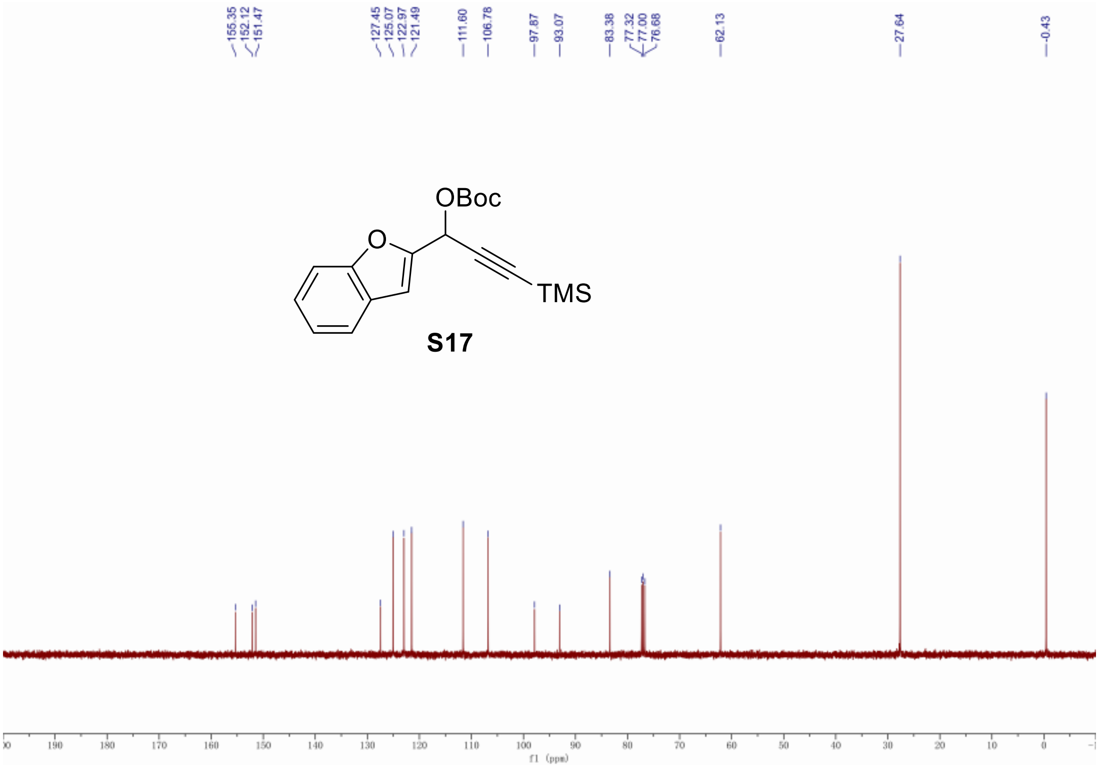

(^{1}H NMR, 400 MHz, $CDCl_3$)

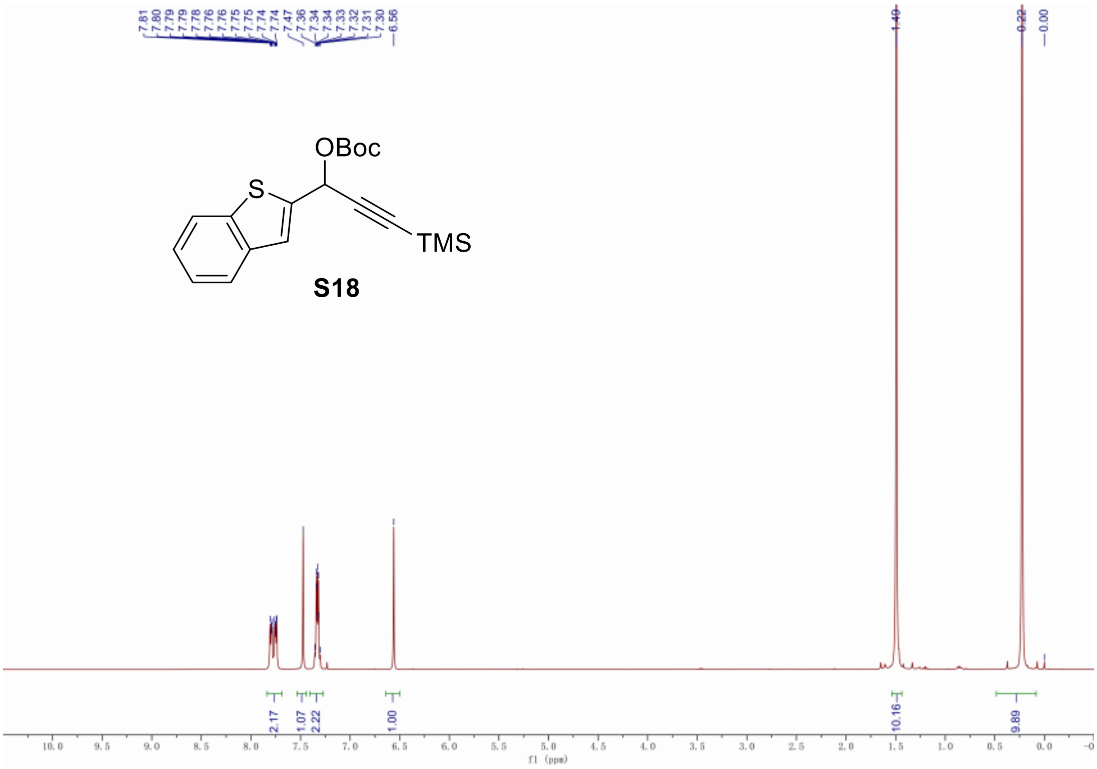

(^{13}C NMR, 100 MHz, $CDCl_3$)

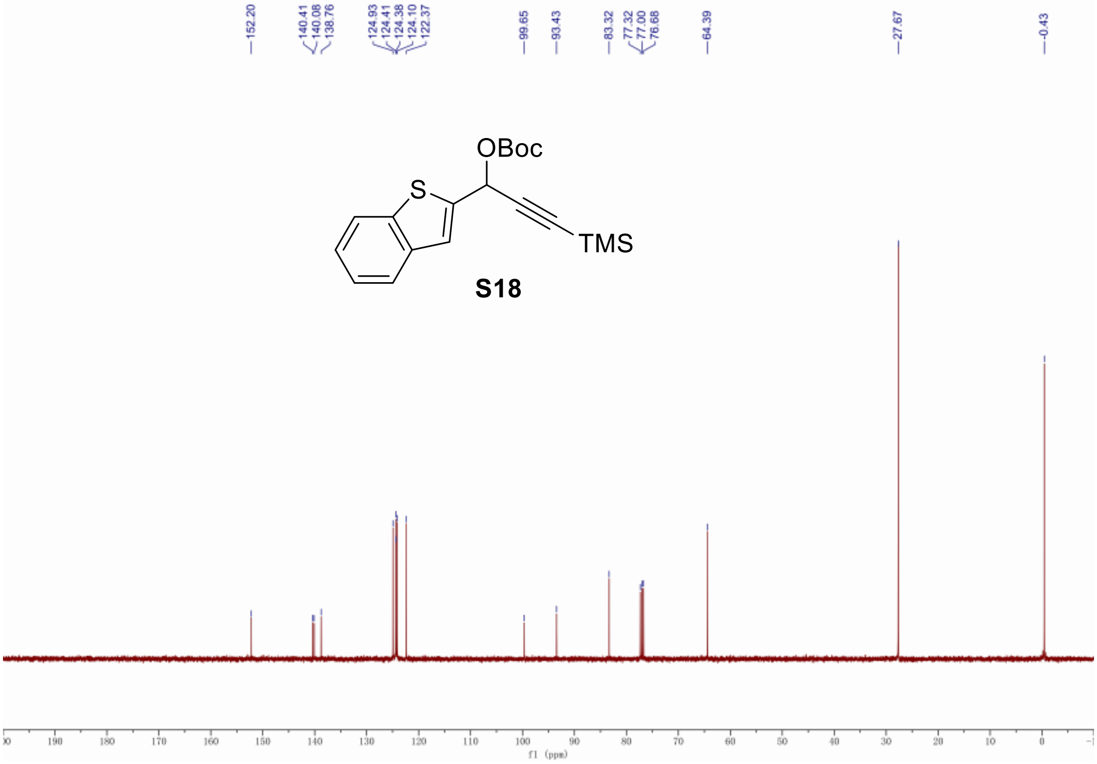

(^{1}H NMR, 400 MHz, $CDCl_3$)

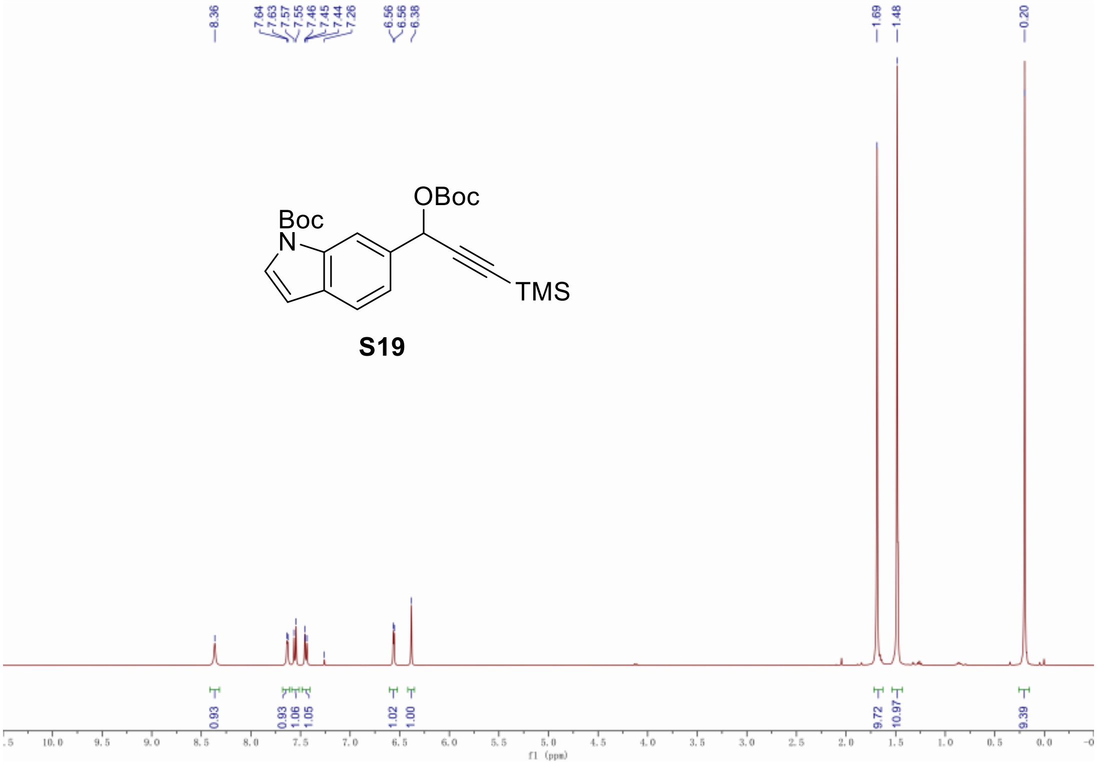

(^{13}C NMR, 100 MHz, CDCl$_3$)

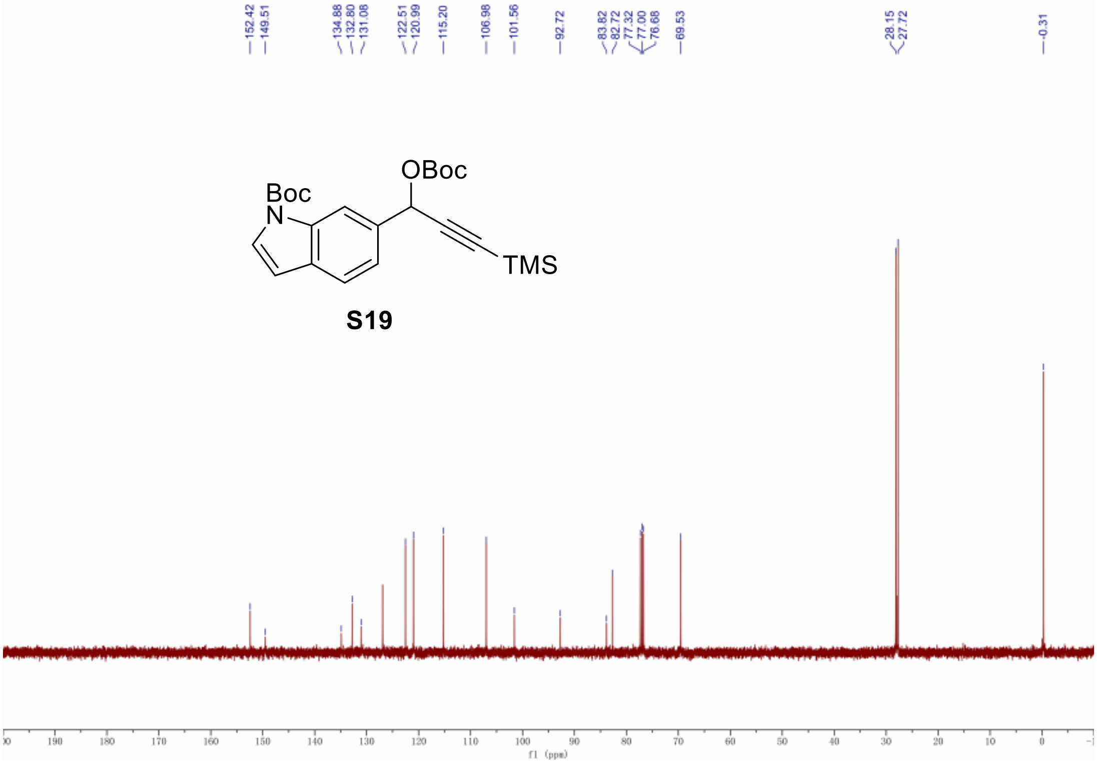

(^{1}H NMR, 400 MHz, CDCl$_3$)

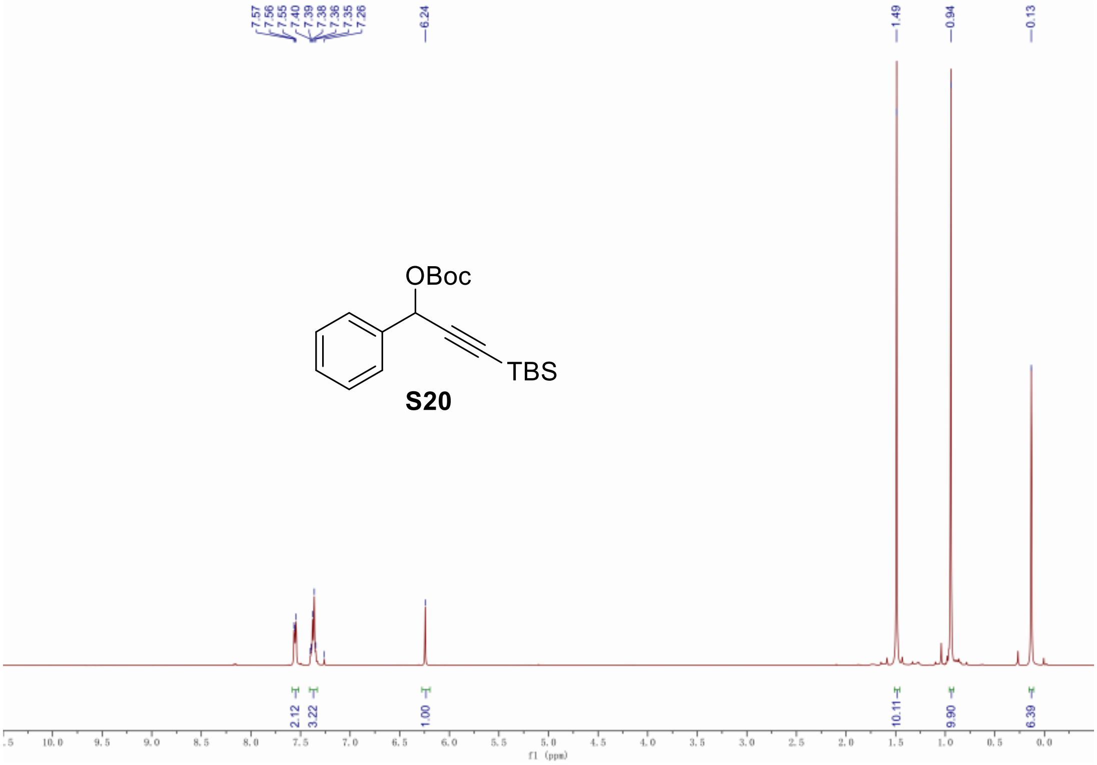

(^{13}C NMR, 100 MHz, CDCl$_3$)

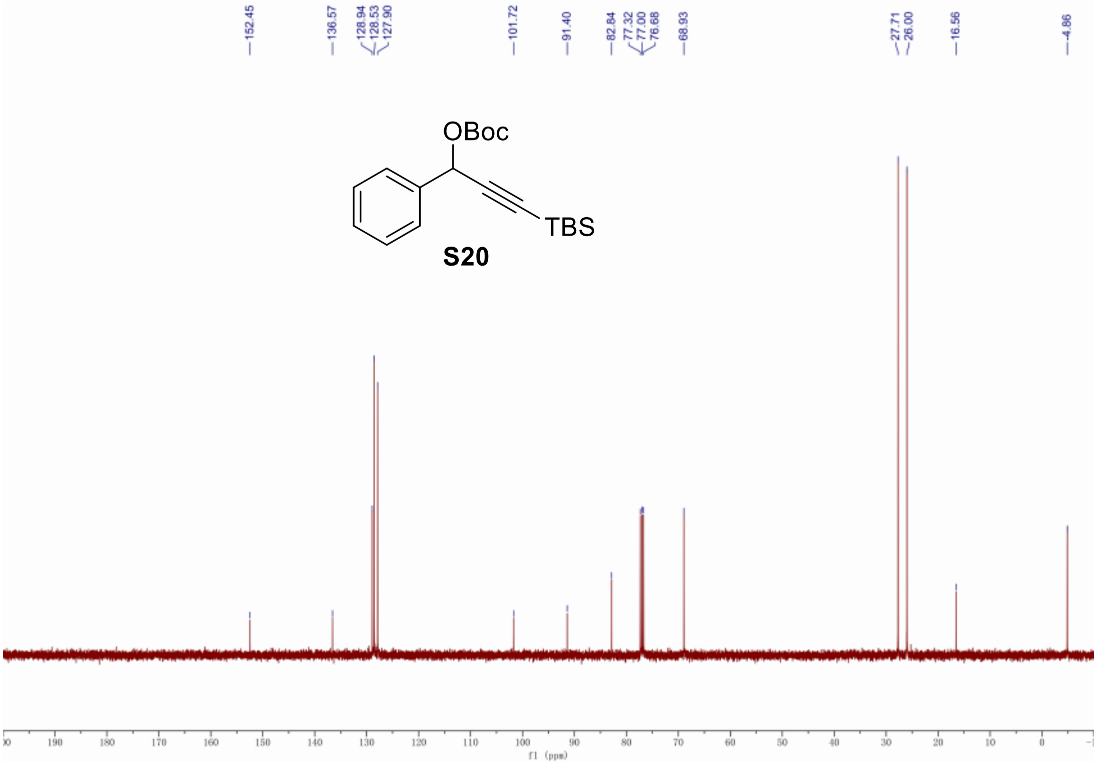

(^{1}H NMR, 400 MHz, CDCl$_3$)

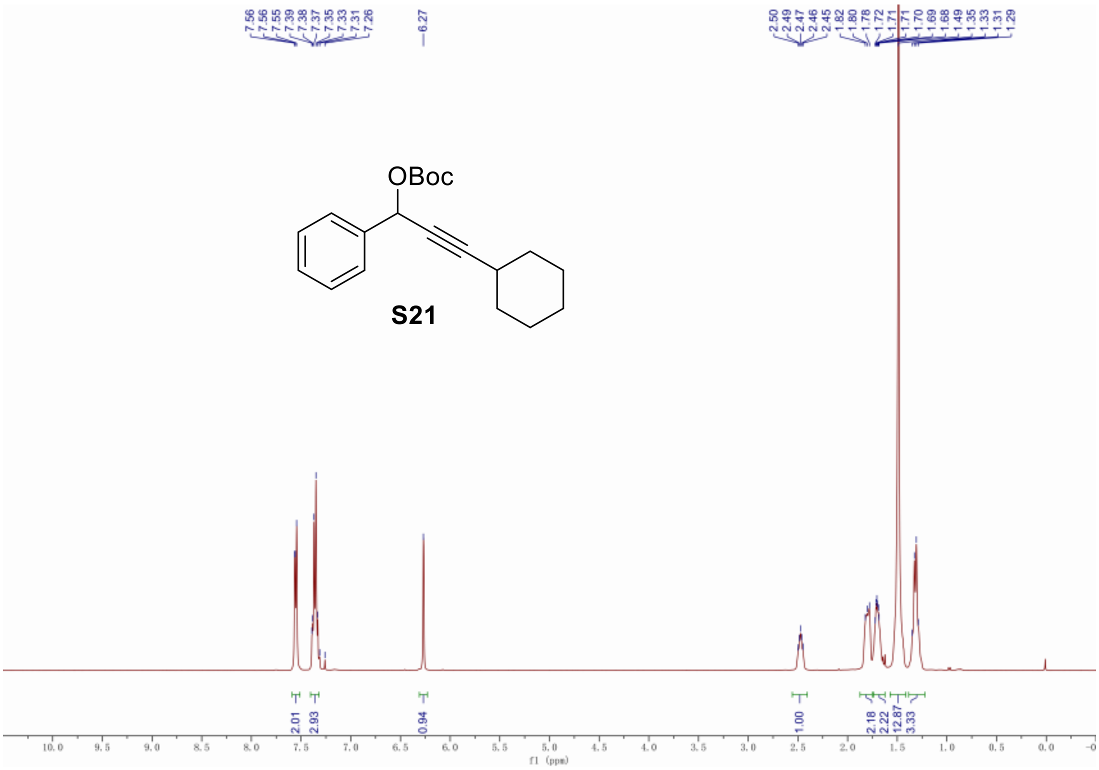

(^{13}C NMR, 100 MHz, CDCl$_3$)

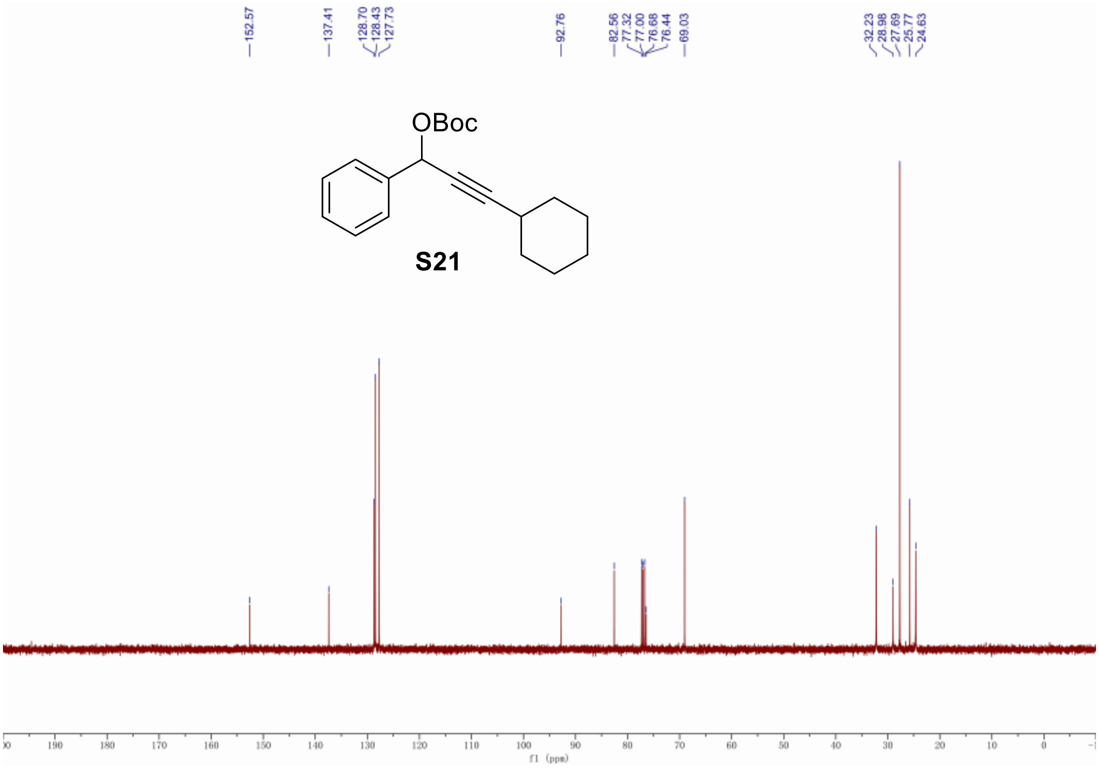

(^{1}H NMR, 400 MHz, CDCl$_3$)

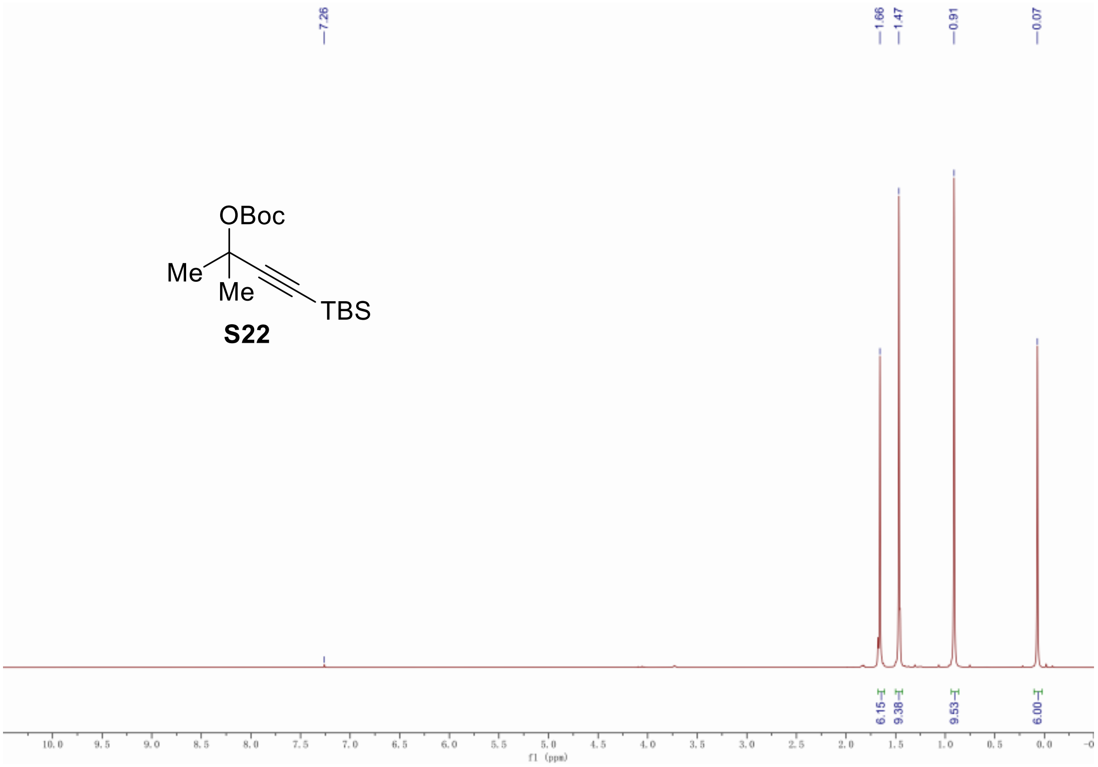

(^{13}C NMR, 100 MHz, CDCl$_3$)

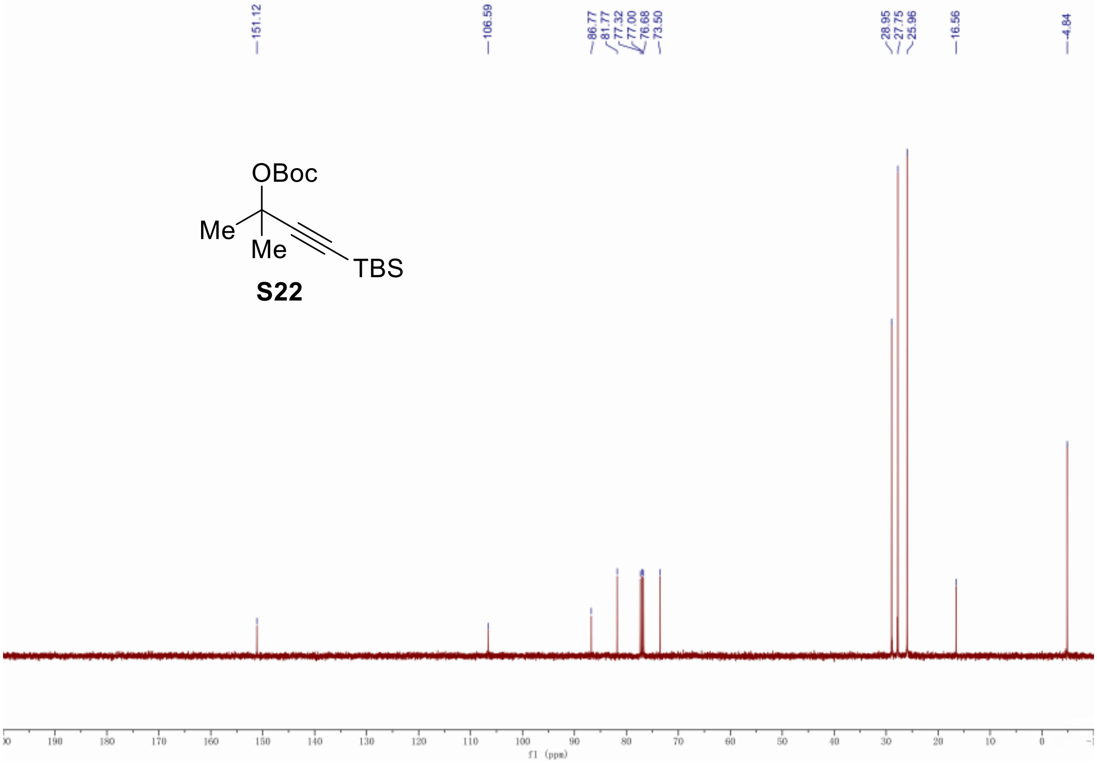

(^{1}H NMR, 400 MHz, CDCl$_3$)

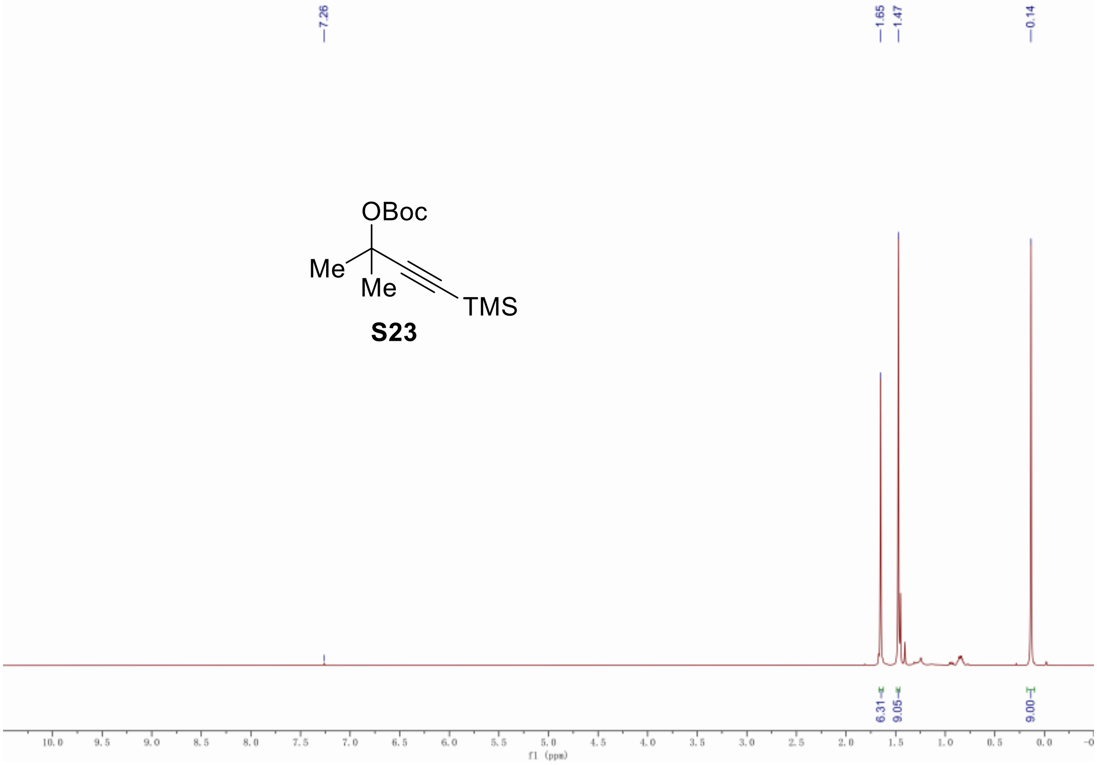

(¹³C NMR, 100 MHz, $CDCl_3$)

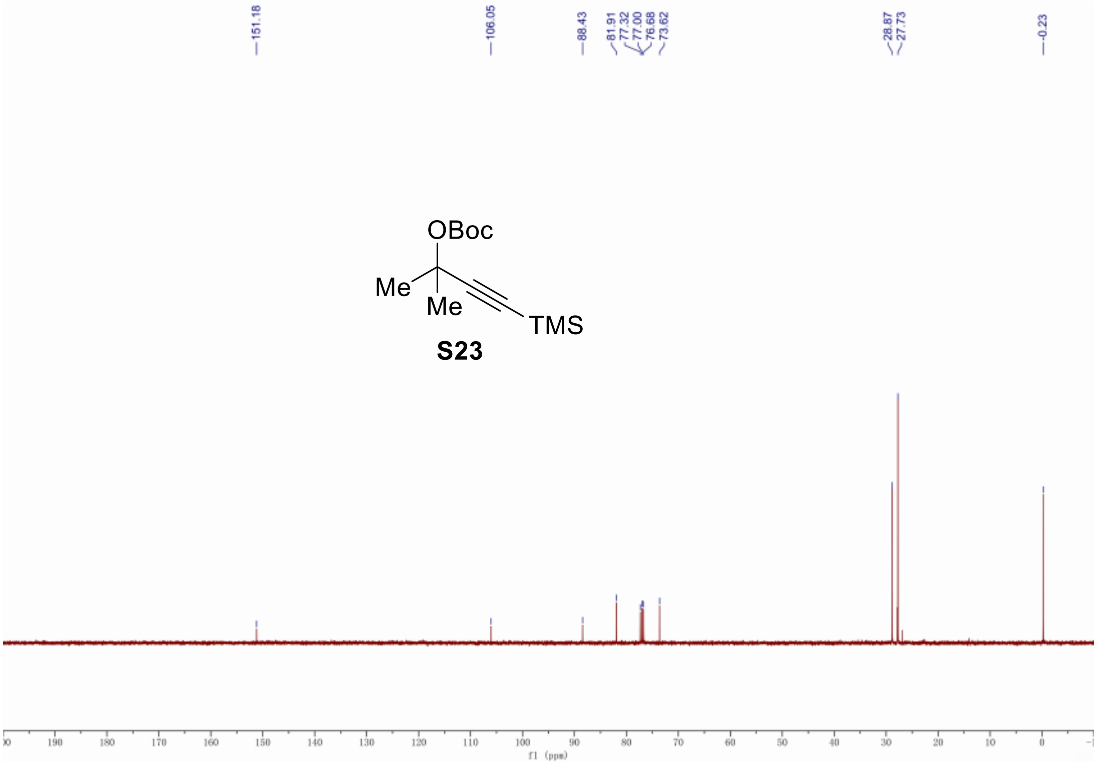

(¹H NMR, 400 MHz, $CDCl_3$)

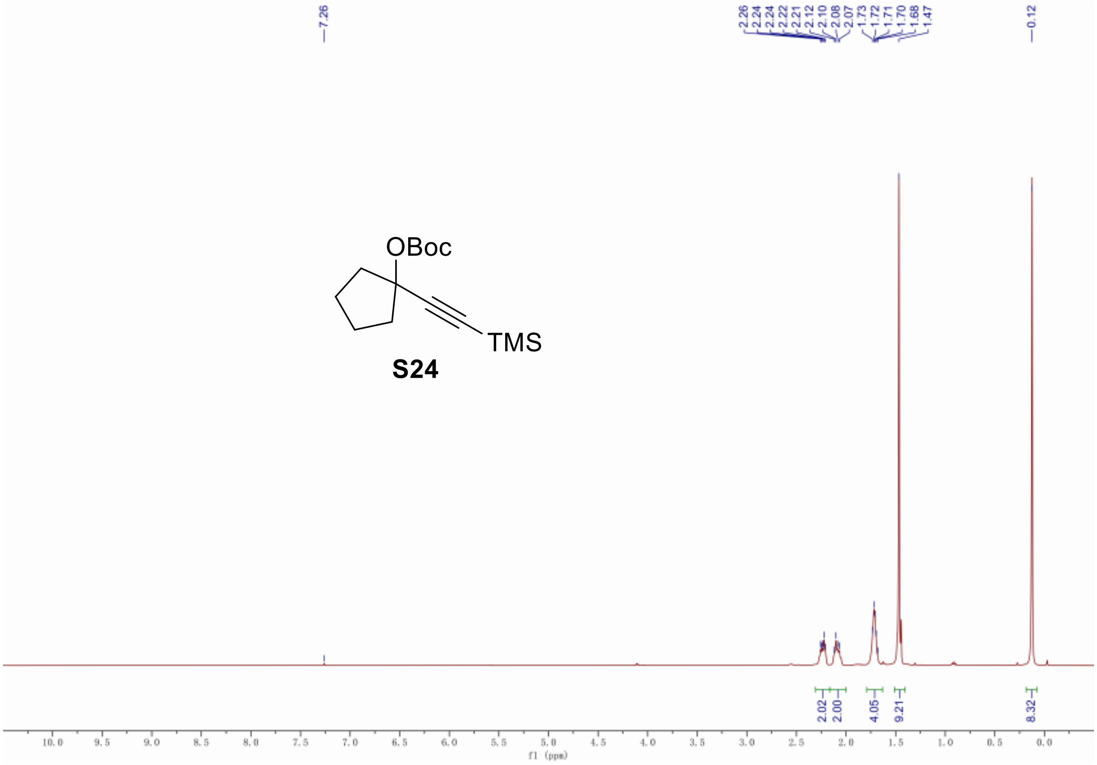

(^{13}C NMR, 100 MHz, CDCl$_3$)

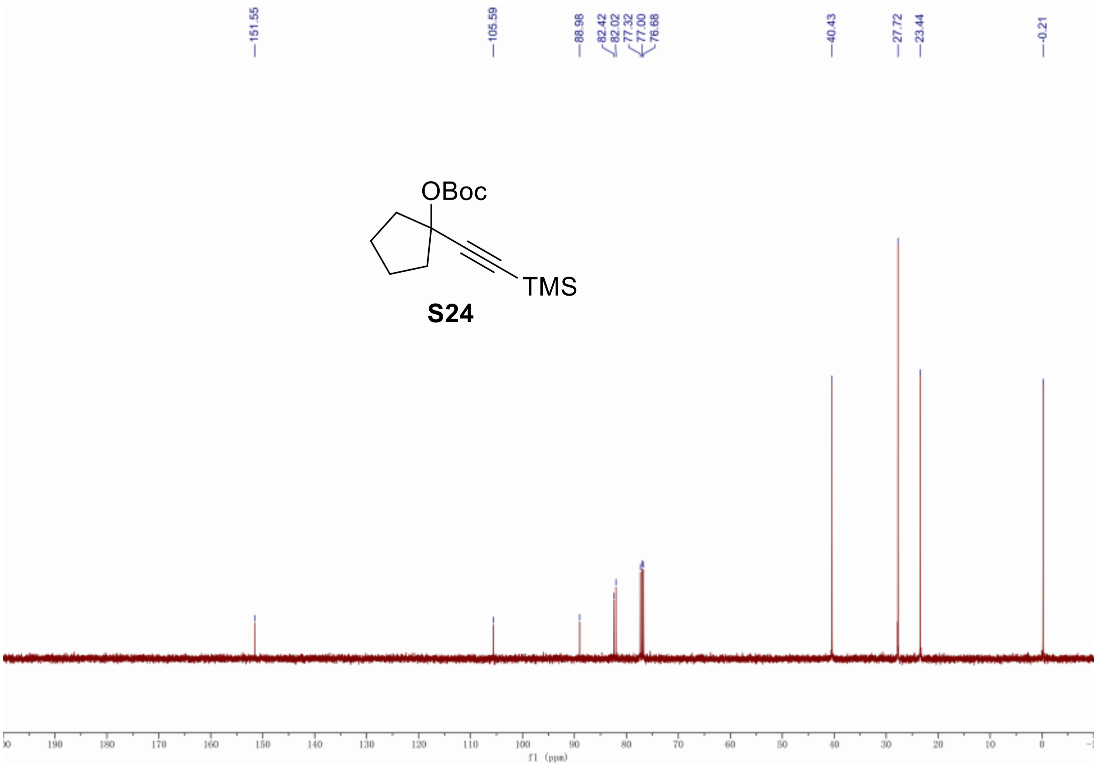

(^{1}H NMR, 400 MHz, CDCl$_3$)

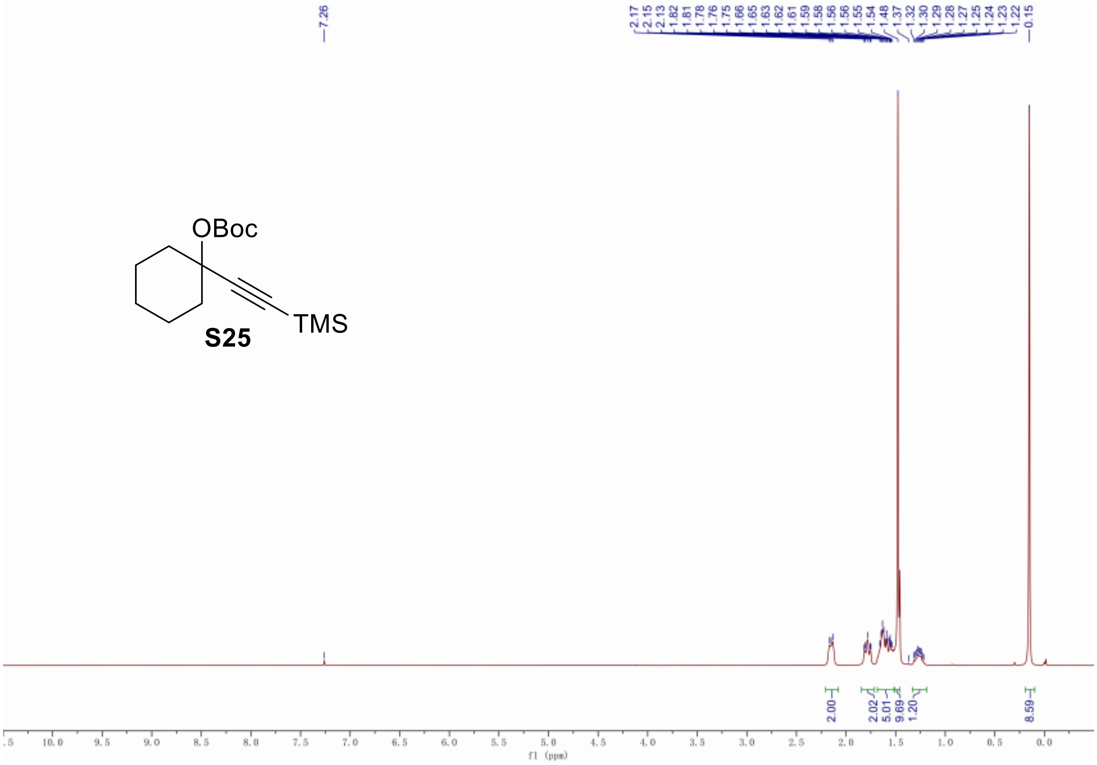

(

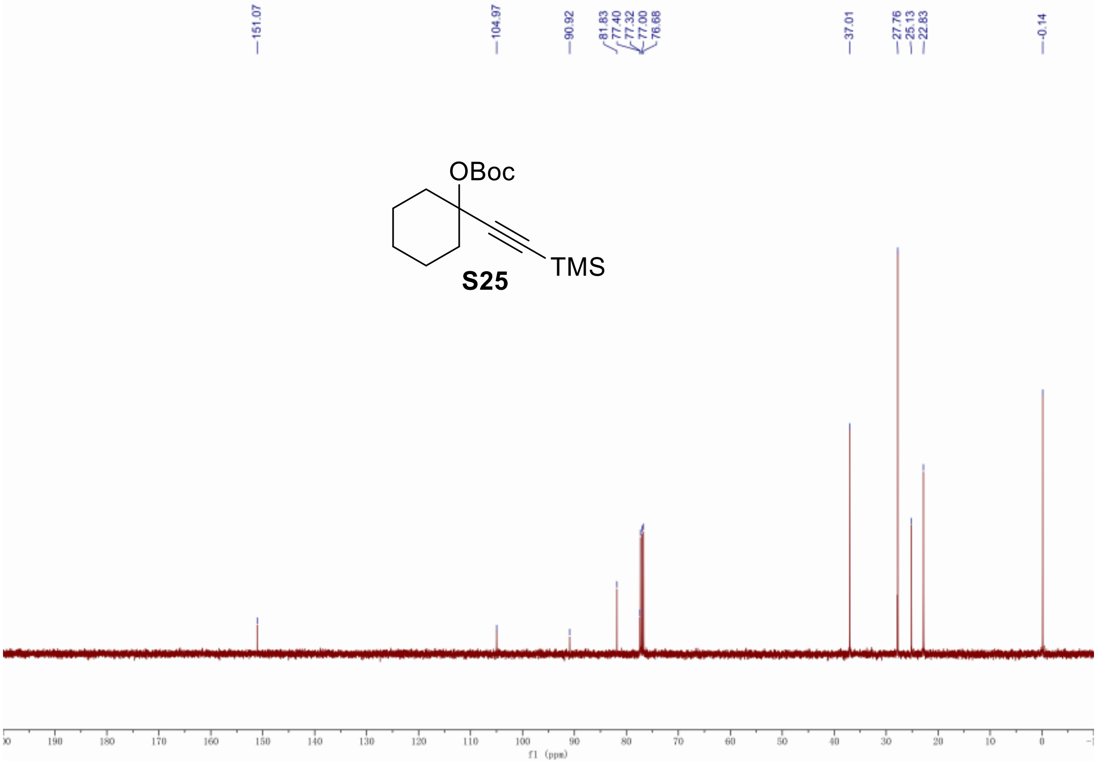

(¹H NMR, 400 MHz, CDCl₃)

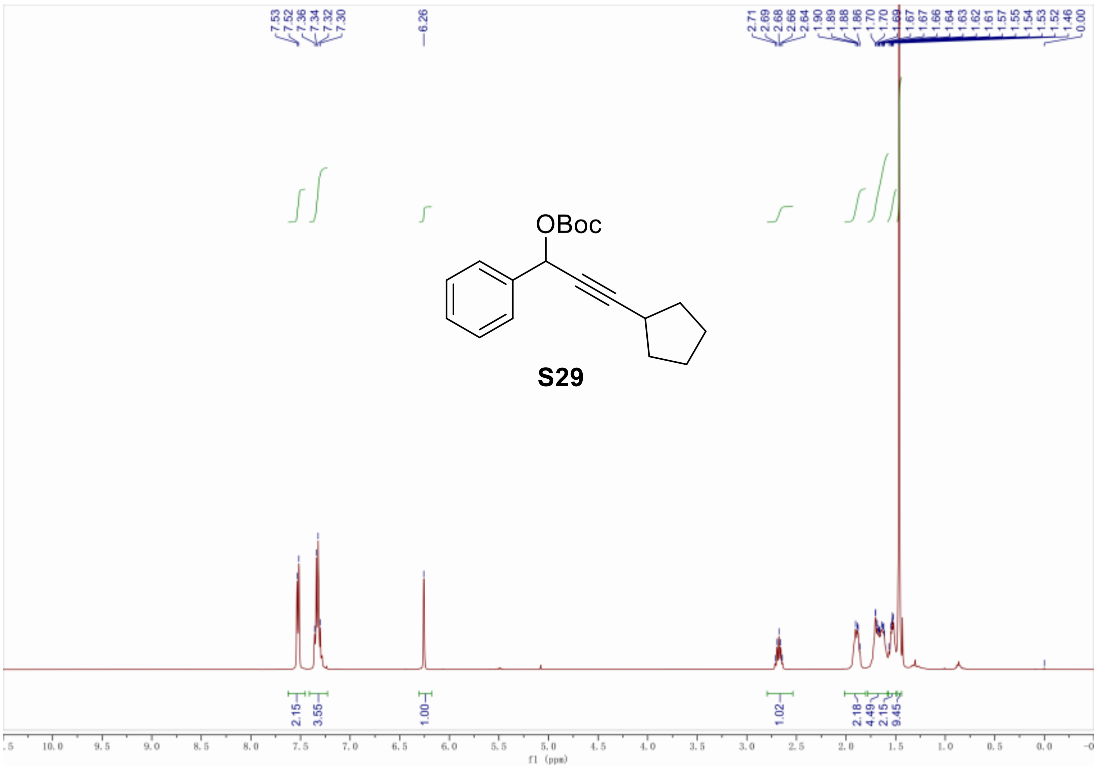

(^{13}C NMR, 100 MHz, CDCl$_3$)

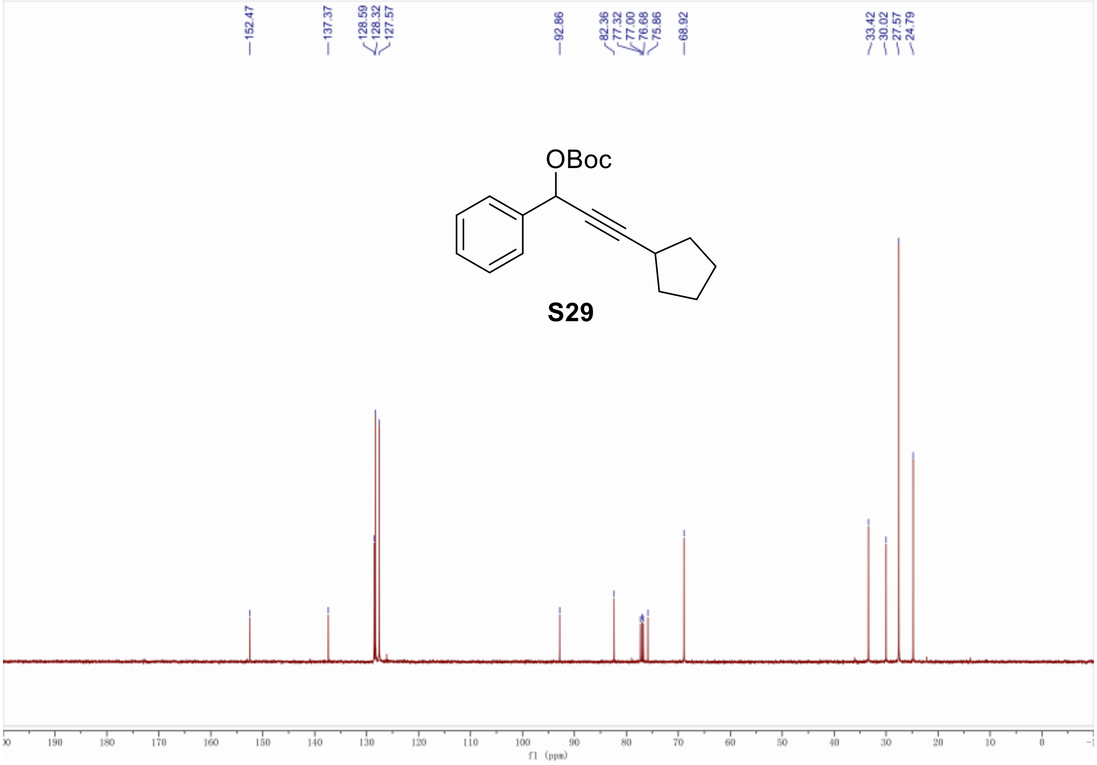

(^{1}H NMR, 400 MHz, CDCl$_3$)

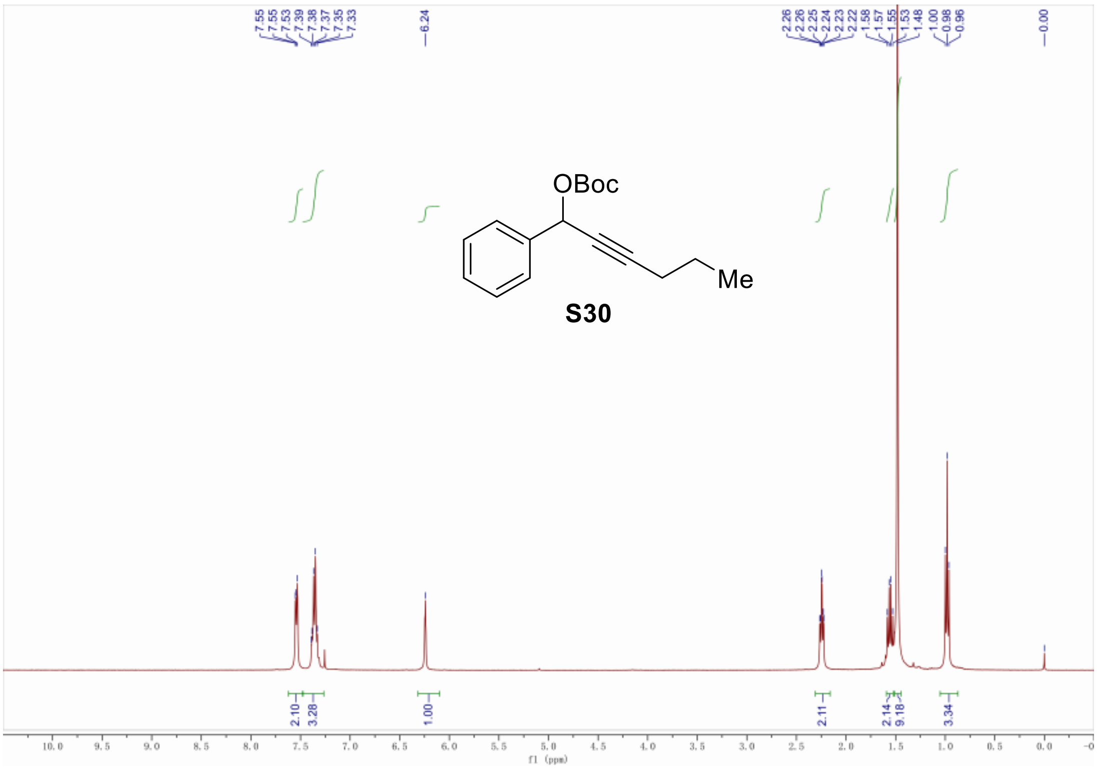

(^{13}C NMR, 100 MHz, CDCl$_3$)

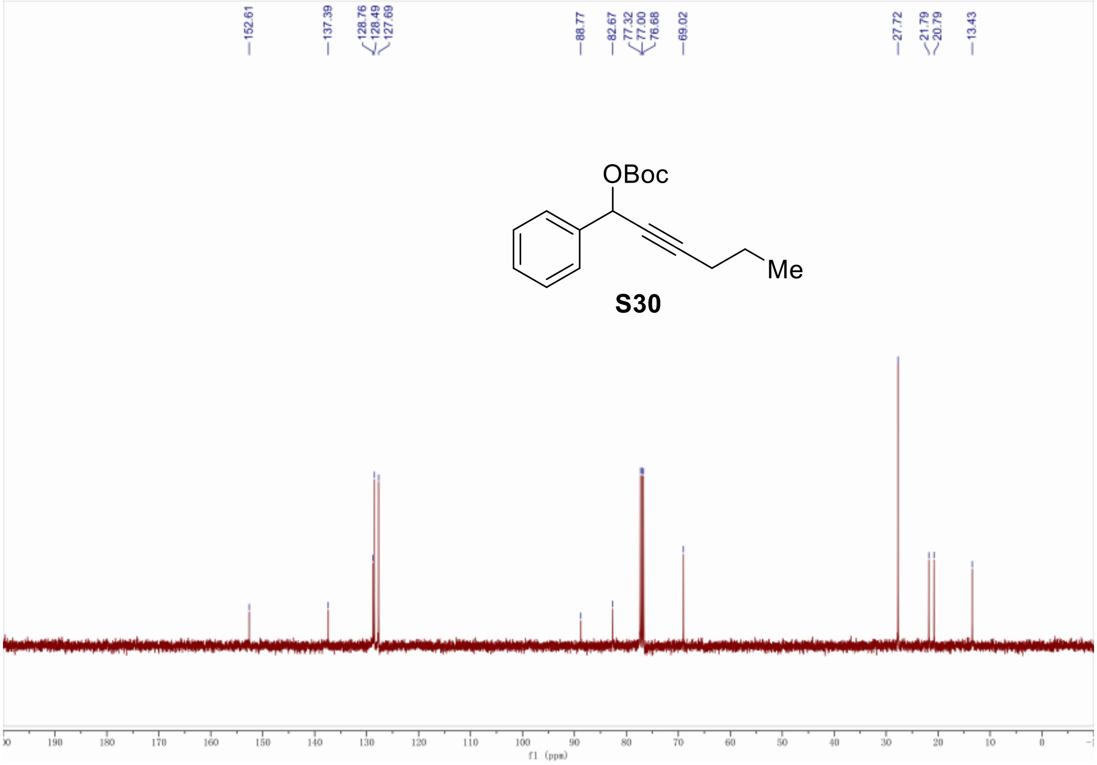

(^{1}H NMR, 400 MHz, CDCl$_3$)

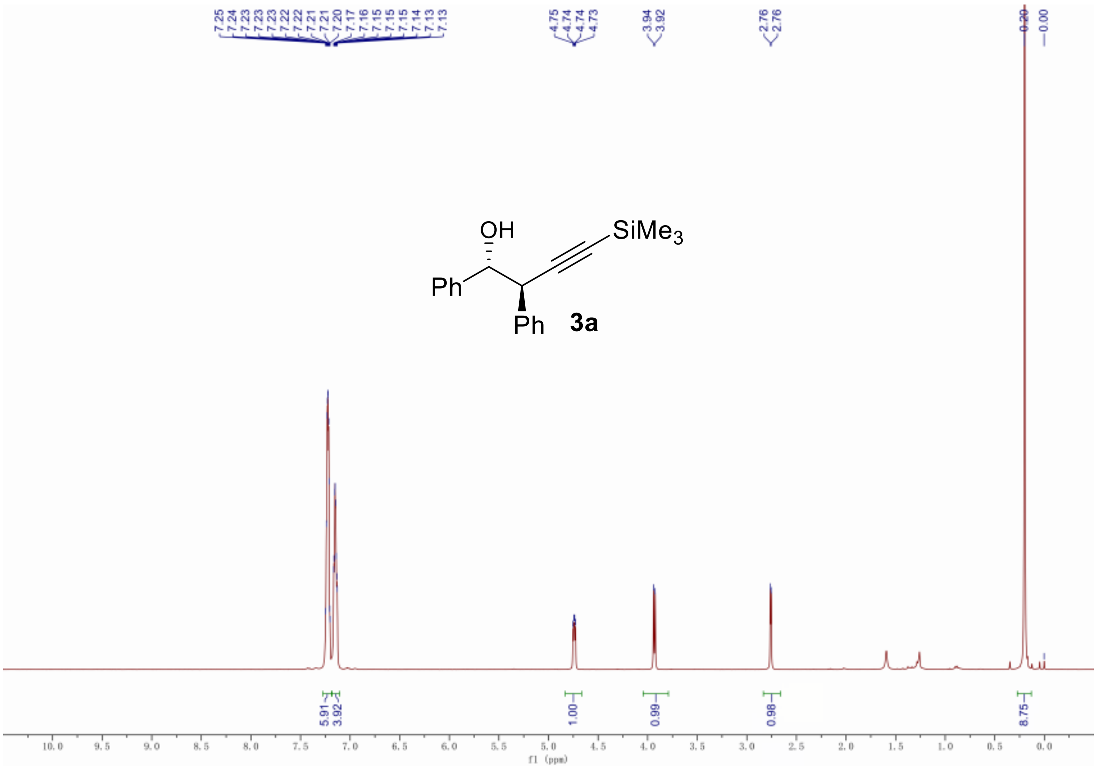

(13C NMR, 100 MHz, $CDCl_3$)

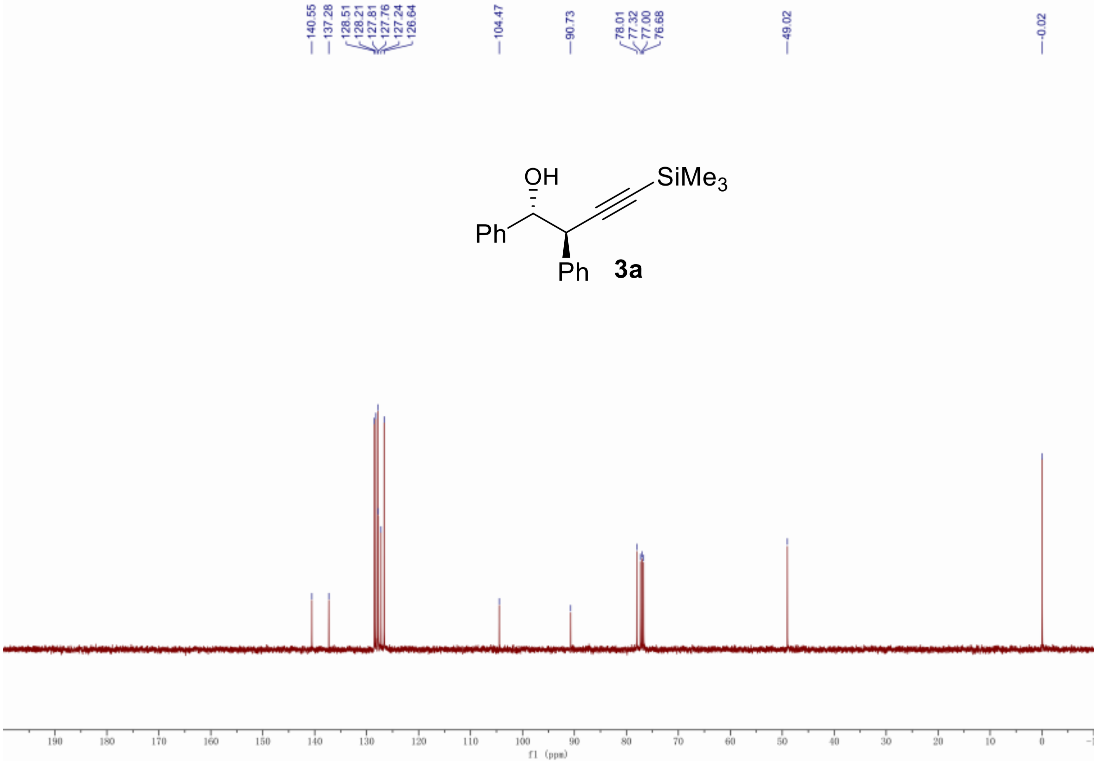

(1H NMR, 400 MHz, $CDCl_3$)

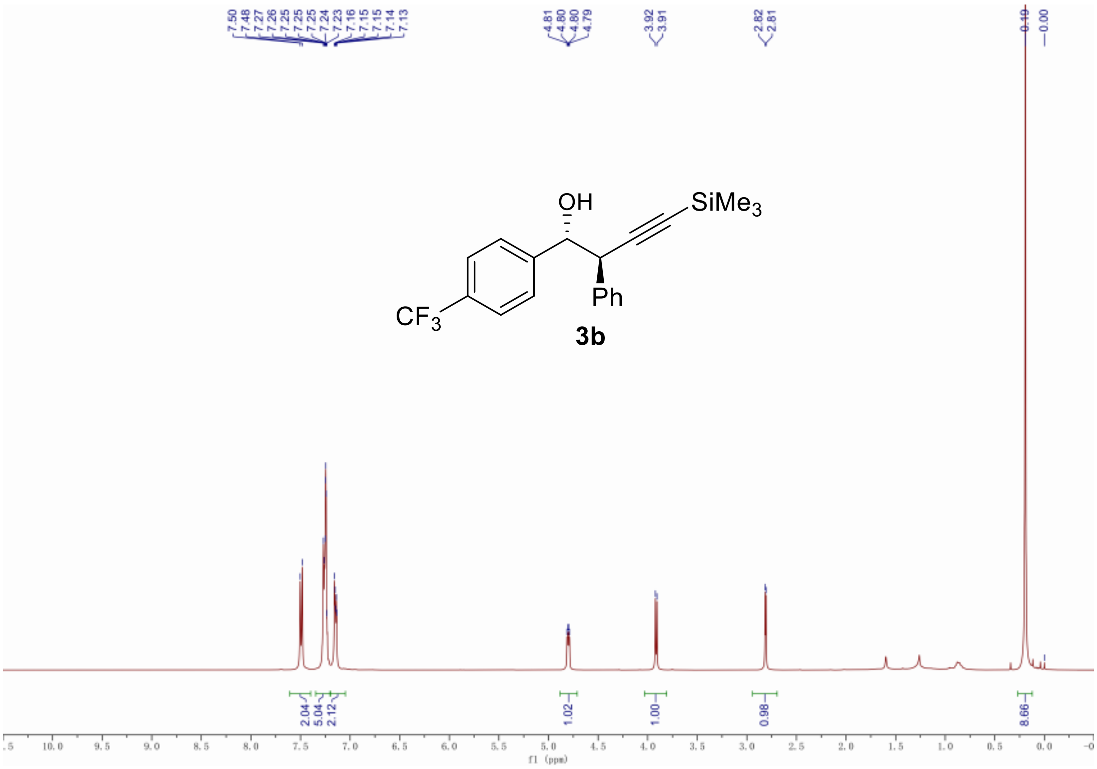

(13C NMR, 100 MHz, $CDCl_3$)

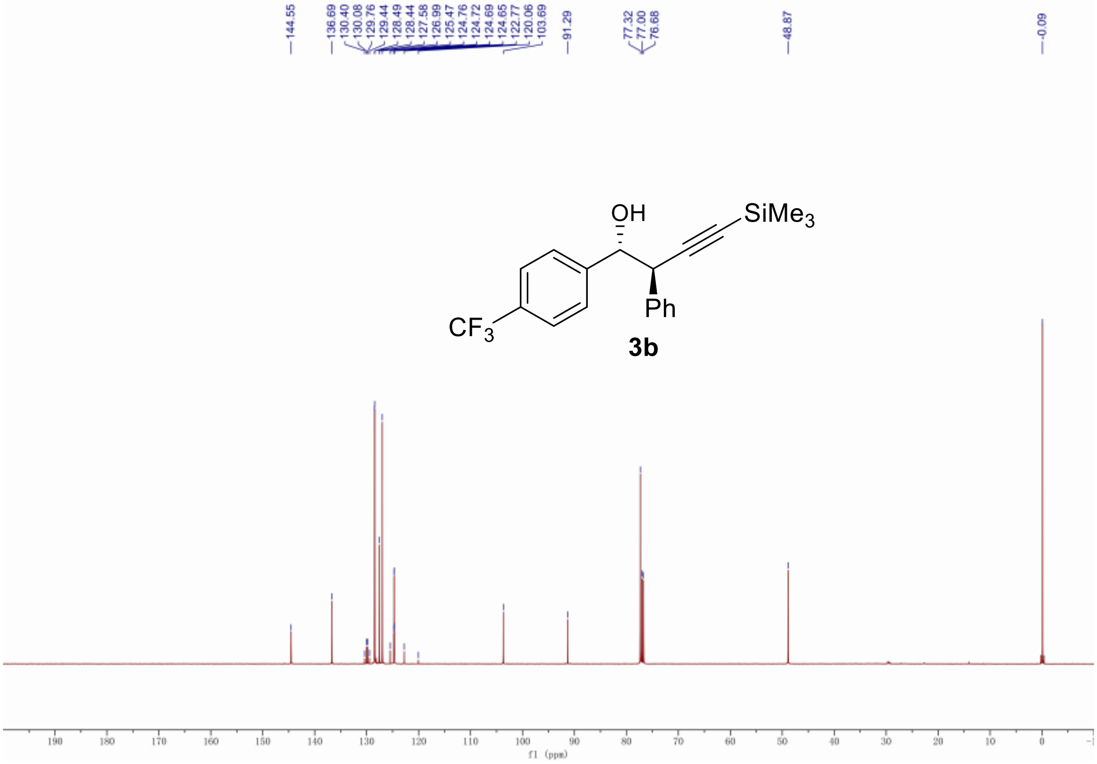

(19F NMR, 376 MHz, $CDCl_3$)

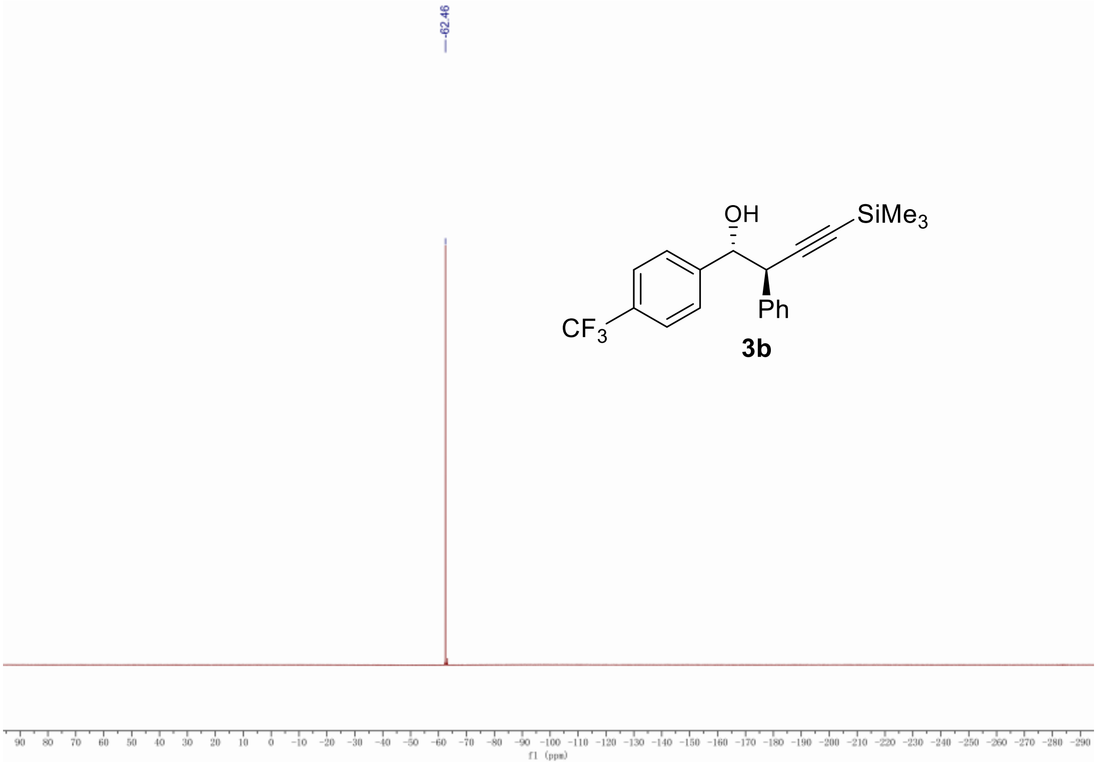

(1H NMR, 400 MHz, CDCl3)

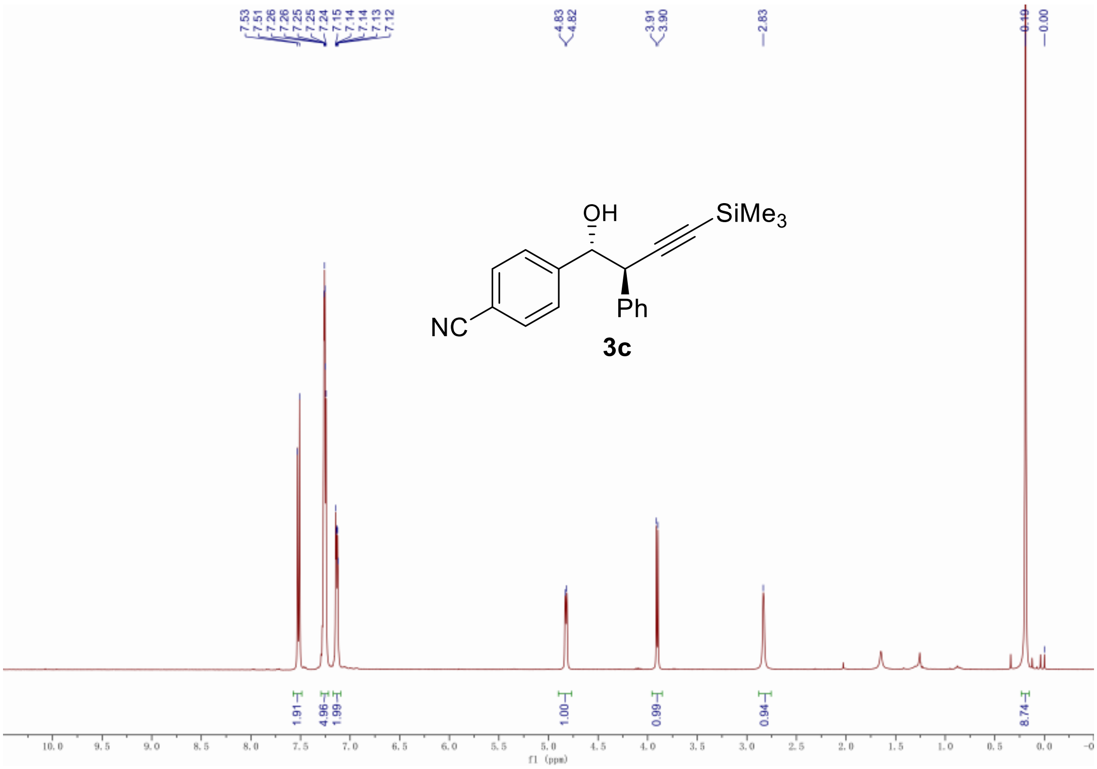

(13C NMR, 100 MHz, CDCl3)

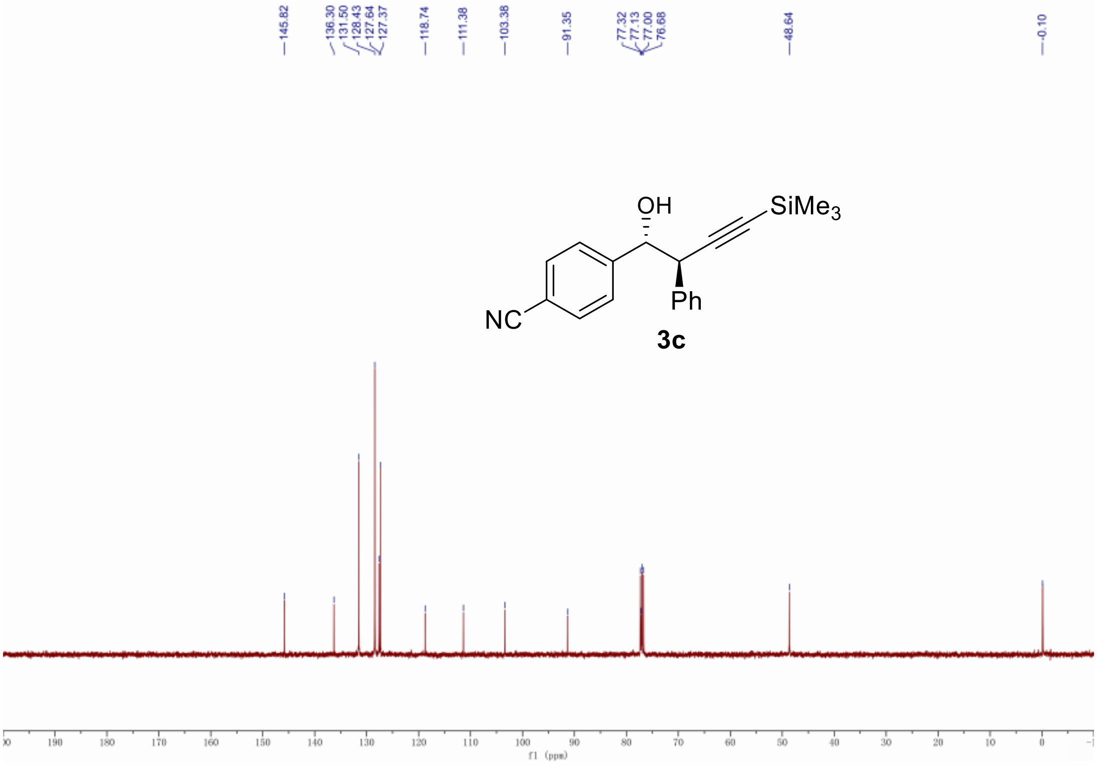

(¹H NMR, 400 MHz, CDCl3)

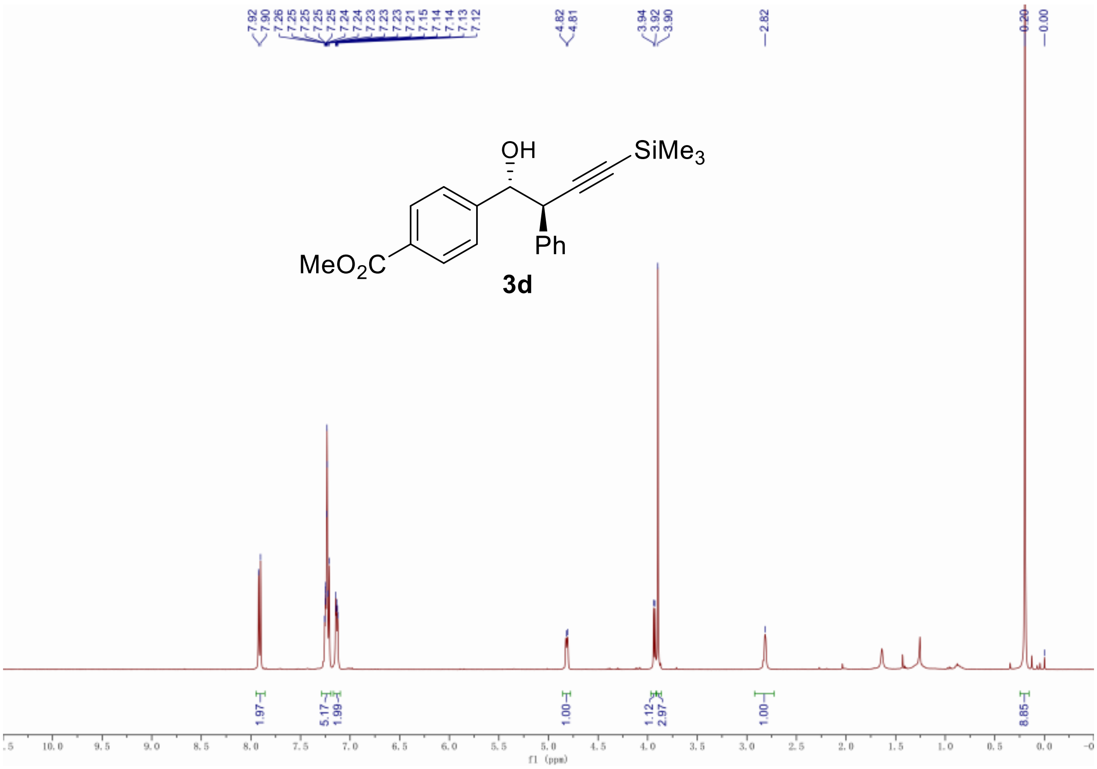

(^{13}C NMR, 100 MHz, CDCl3)

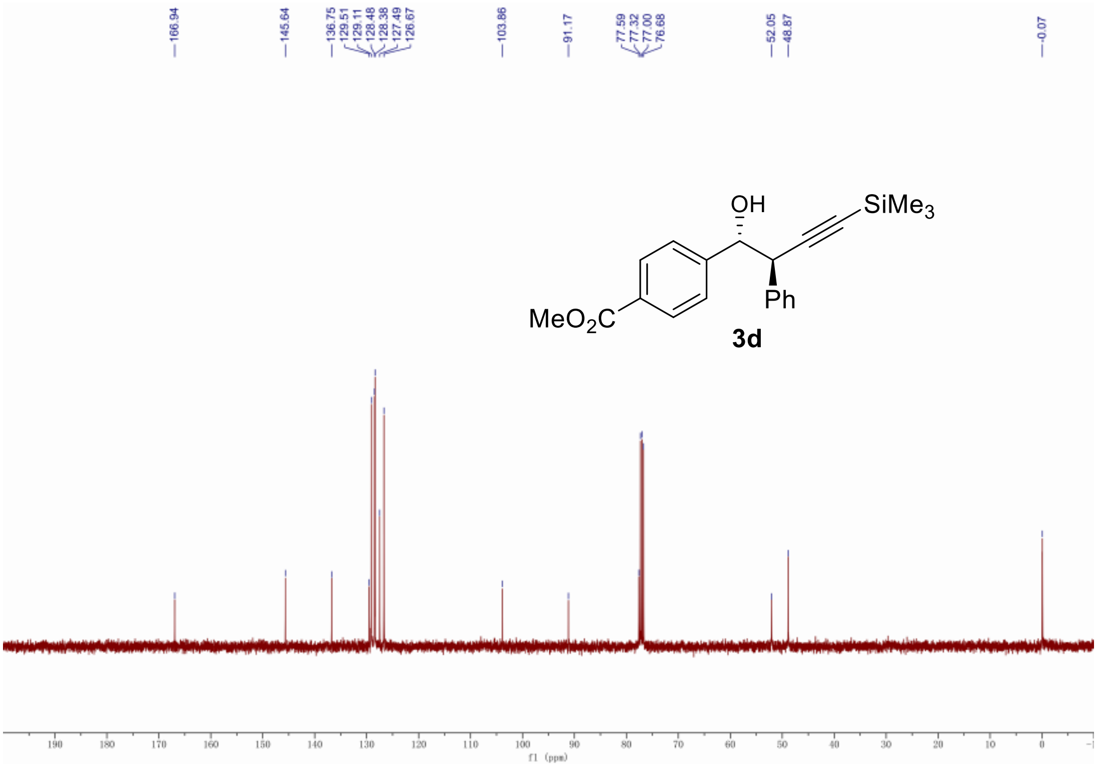

（^{1}H NMR, 400 MHz, $CDCl_3$）

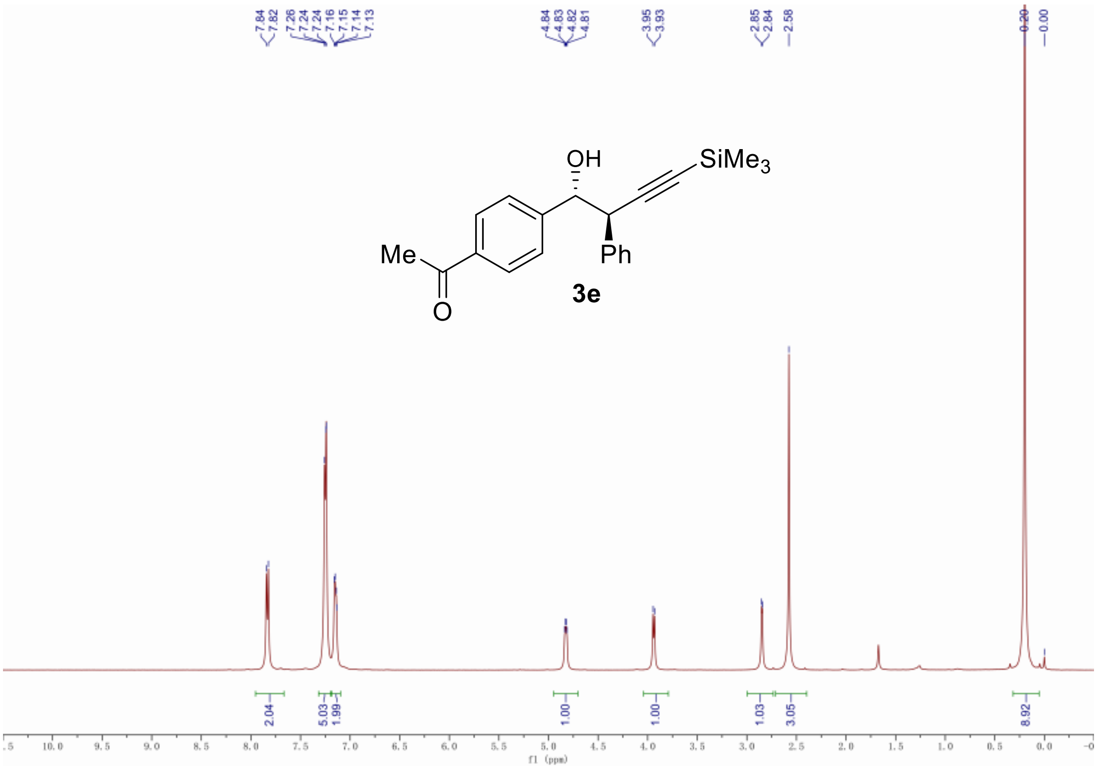

(^{13}C NMR, 100 MHz, $CDCl_3$)

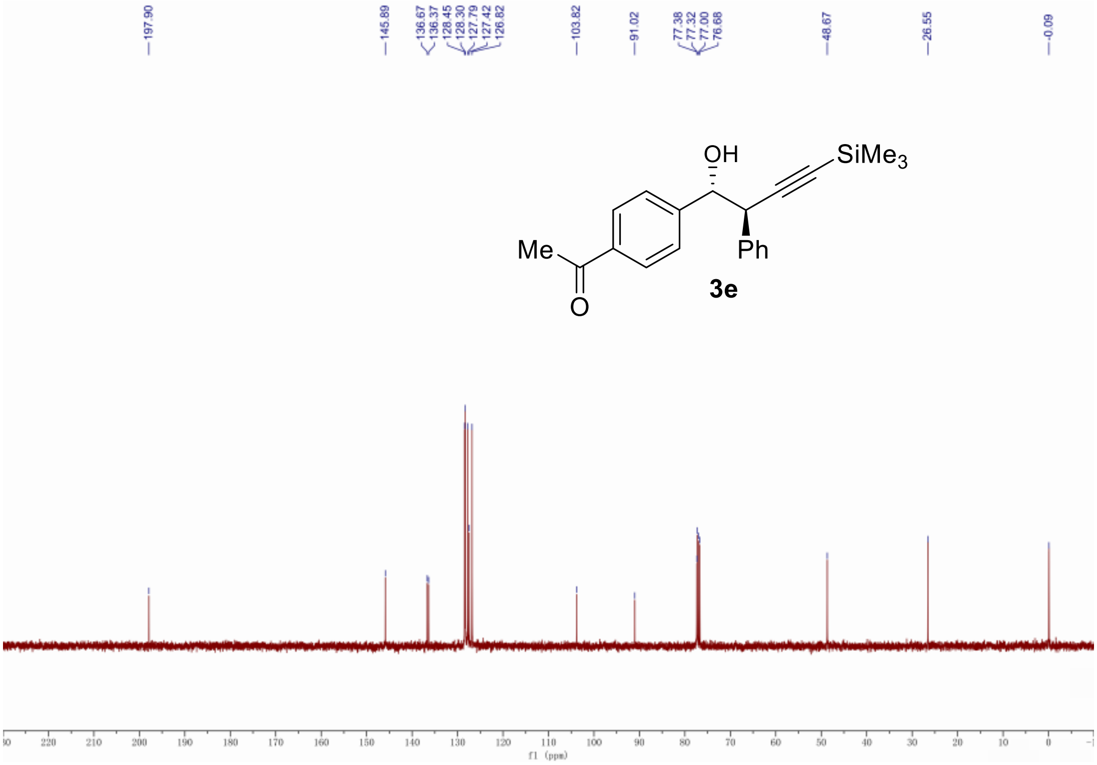

（^{1}H NMR, 400 MHz, $CDCl_3$）

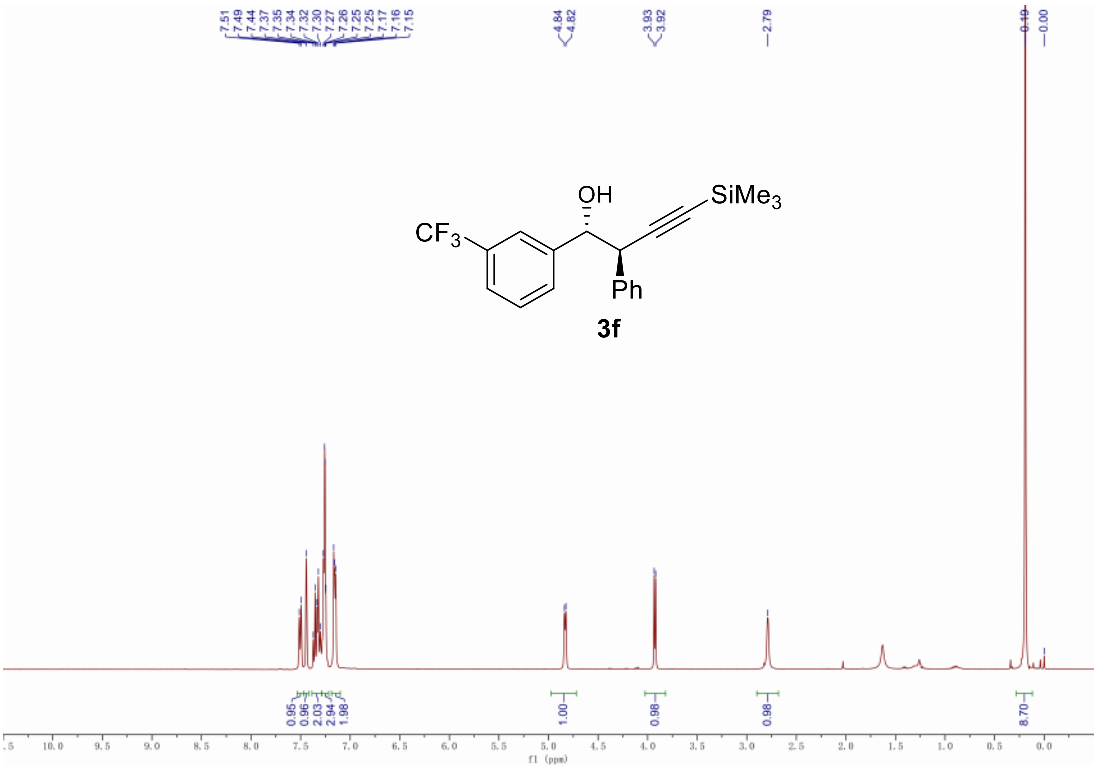

(^{13}C NMR, 100 MHz, $CDCl_3$)

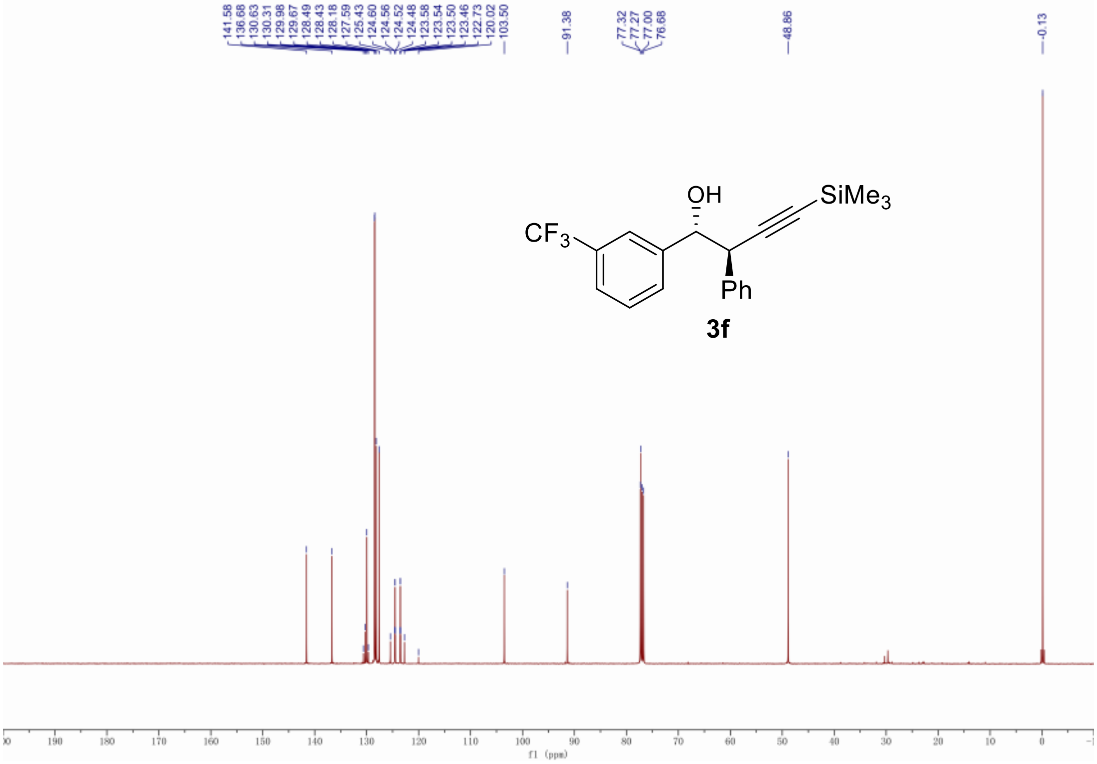

(^{19}F NMR, 376 MHz, $CDCl_3$)

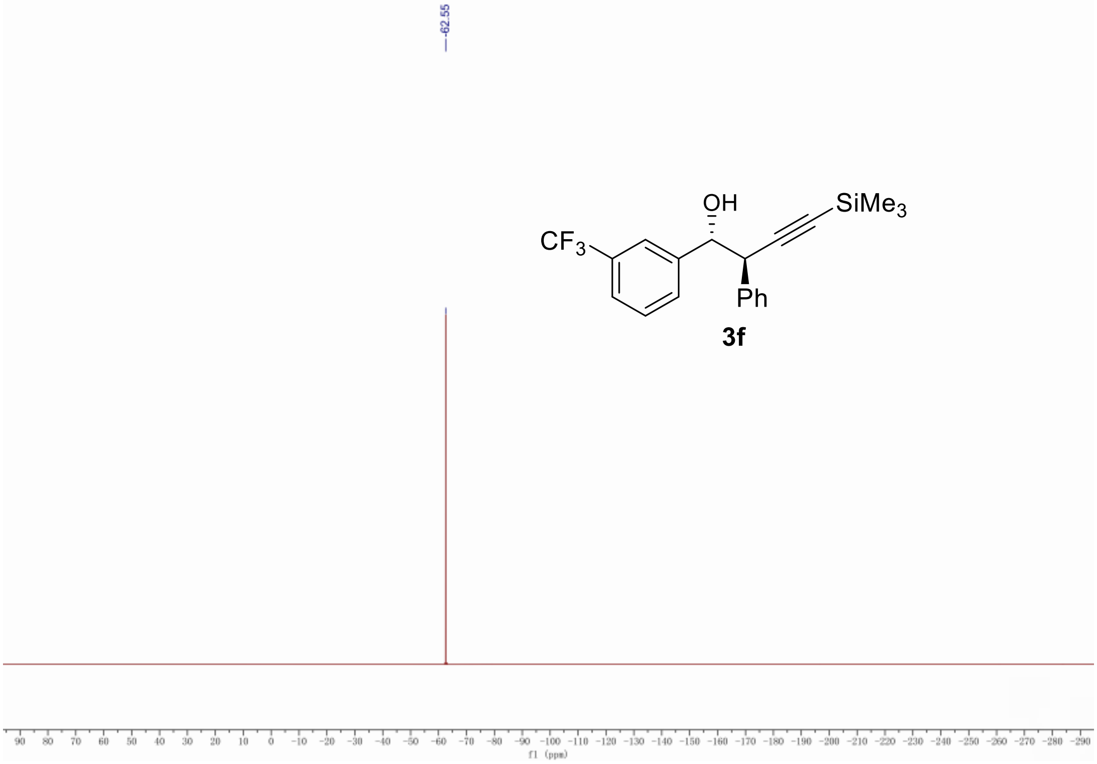

(^{1}H NMR, 400 MHz, $CDCl_3$)

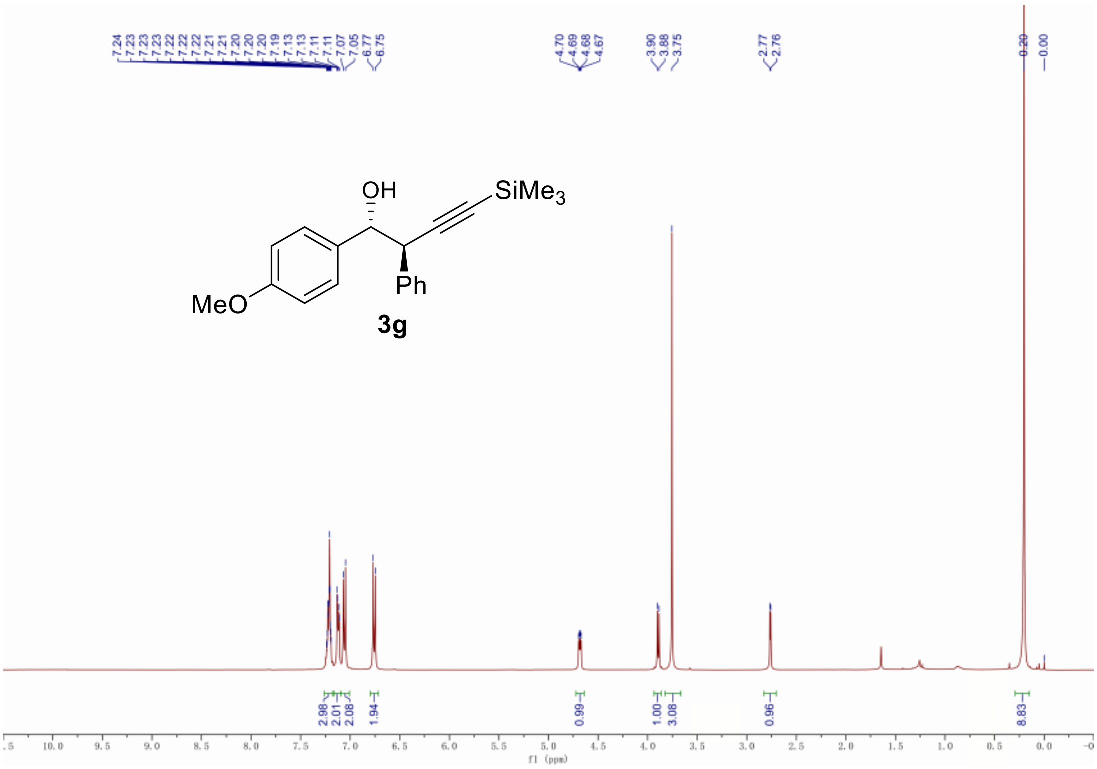

(13C NMR, 100 MHz, CDCl3)

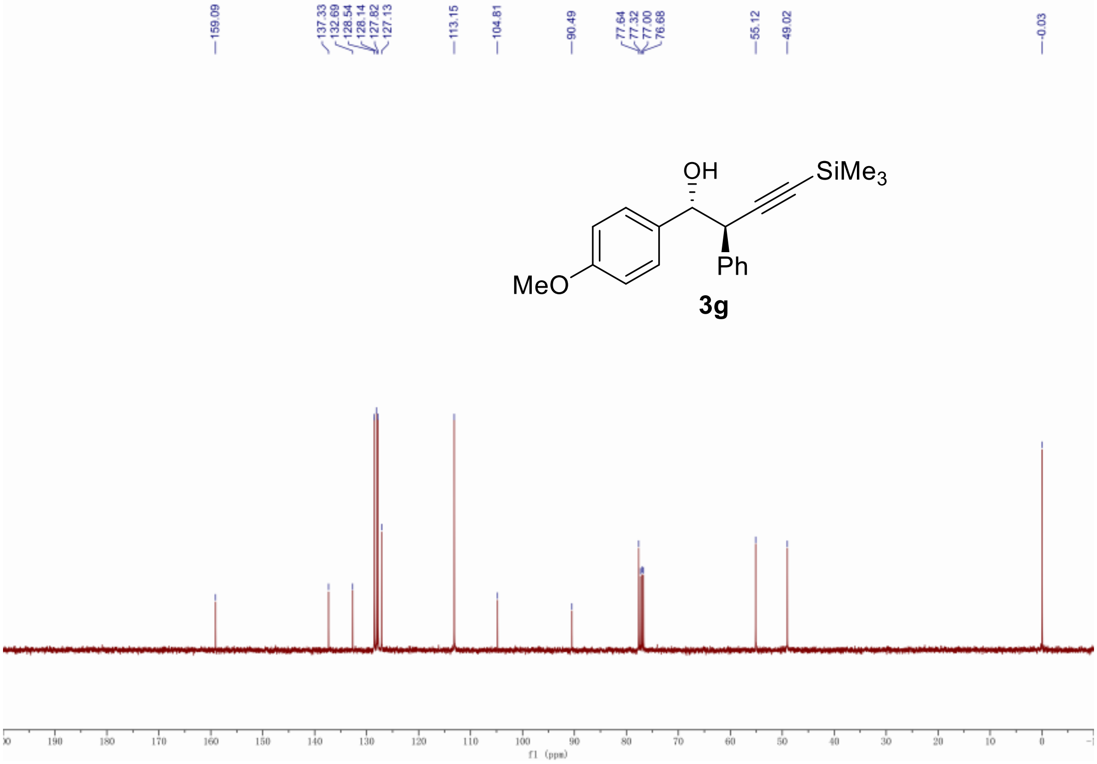

(1H NMR, 400 MHz, CDCl3)

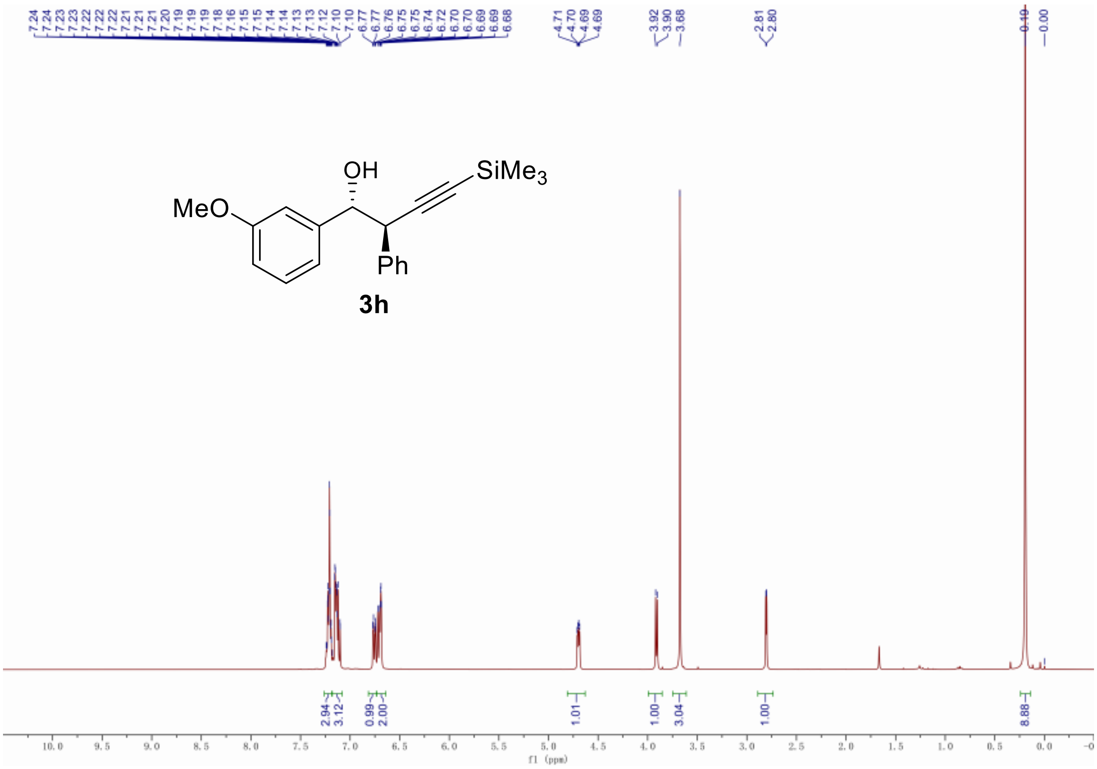

(^{13}C NMR, 100 MHz, CDCl$_3$)

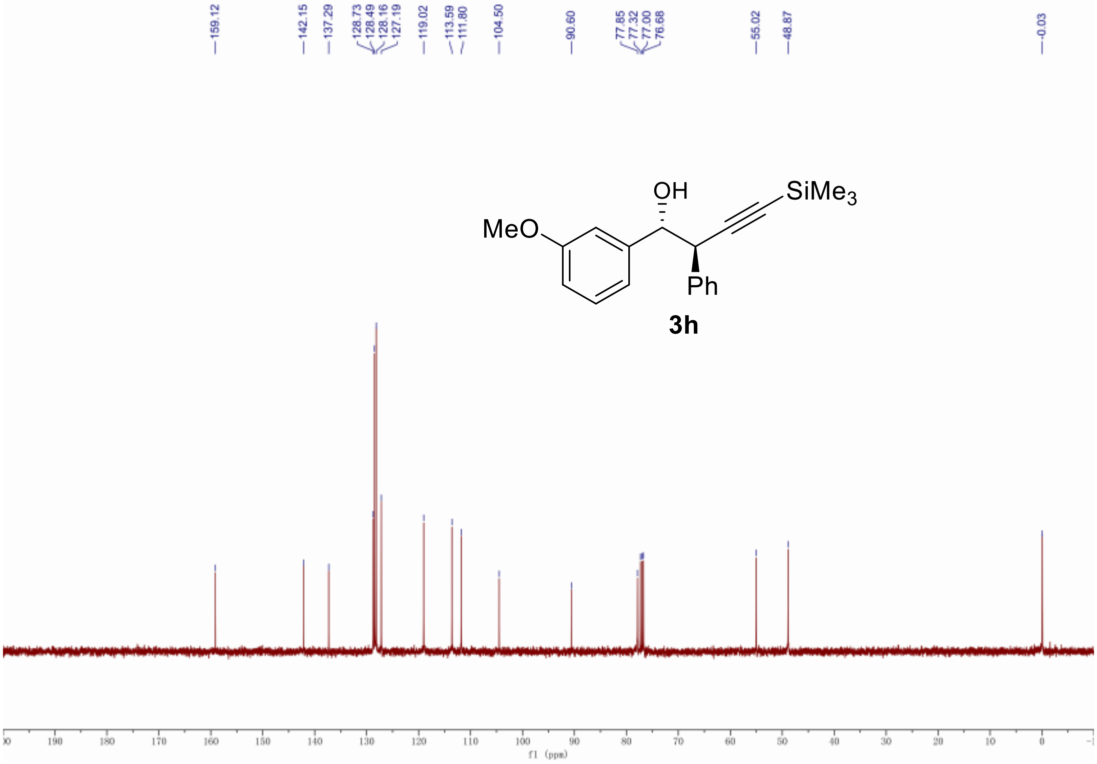

(^{1}H NMR, 400 MHz, CDCl$_3$)

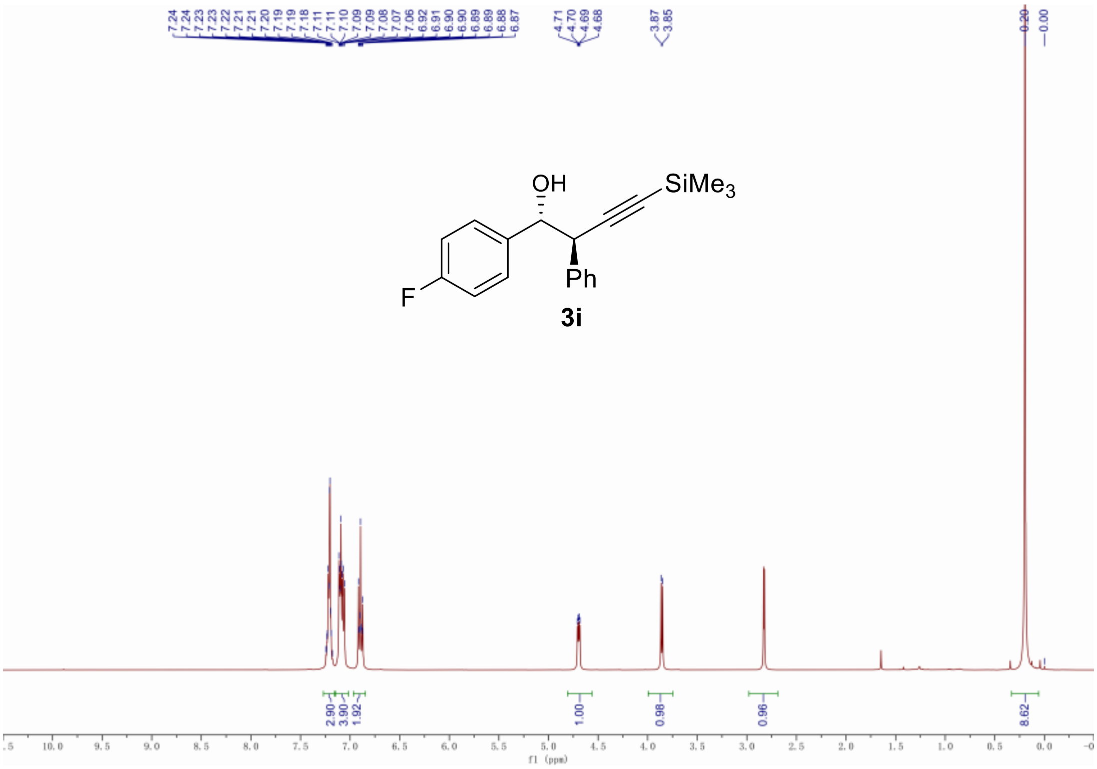

(^{13}C NMR, 100 MHz, CDCl$_3$)

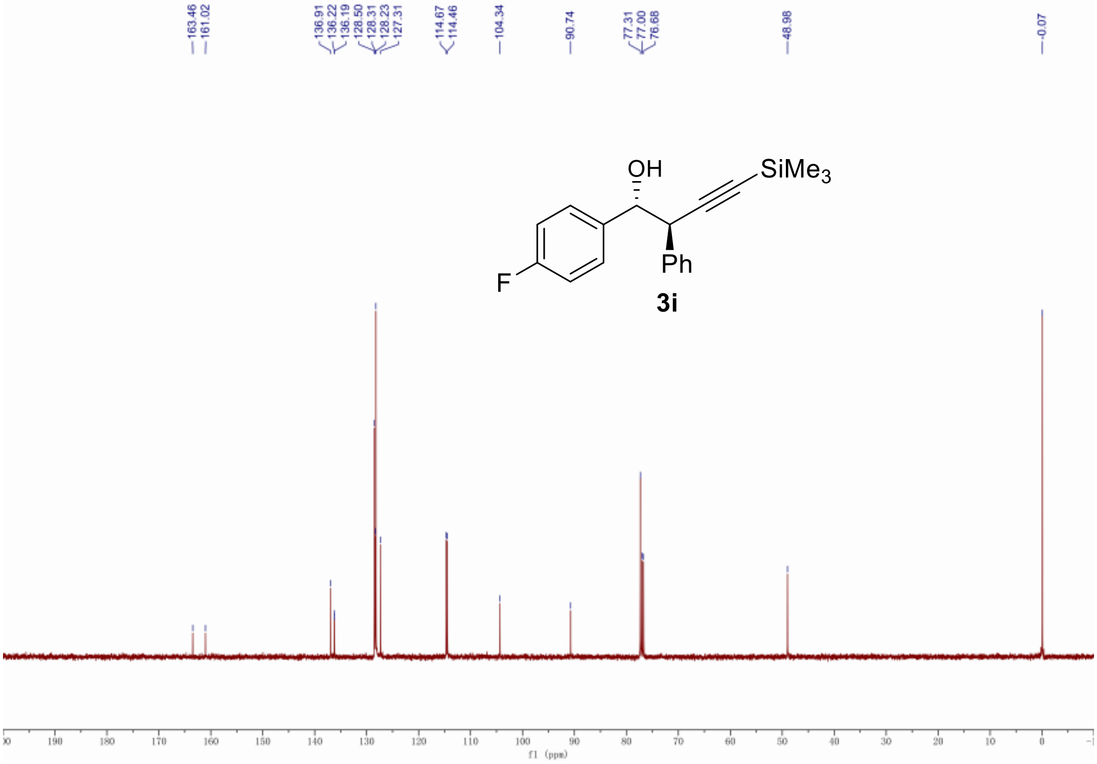

(^{19}F NMR, 376 MHz, CDCl$_3$)

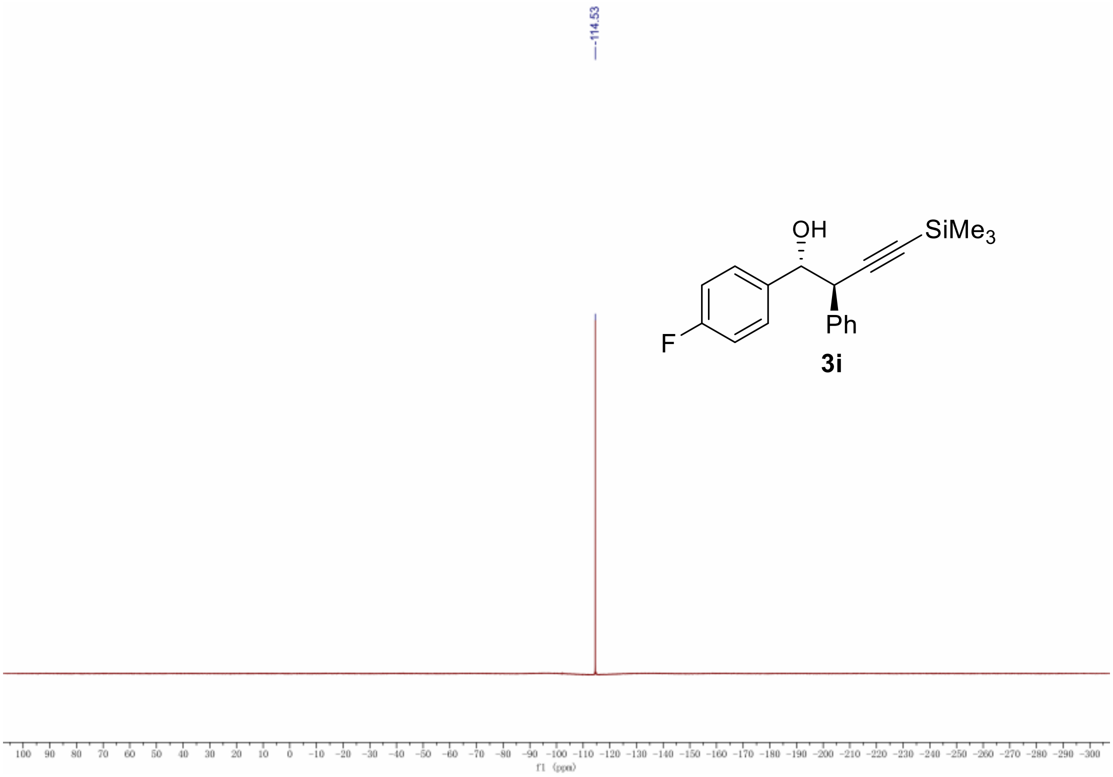

(¹H NMR, 400 MHz, $CDCl_3$)

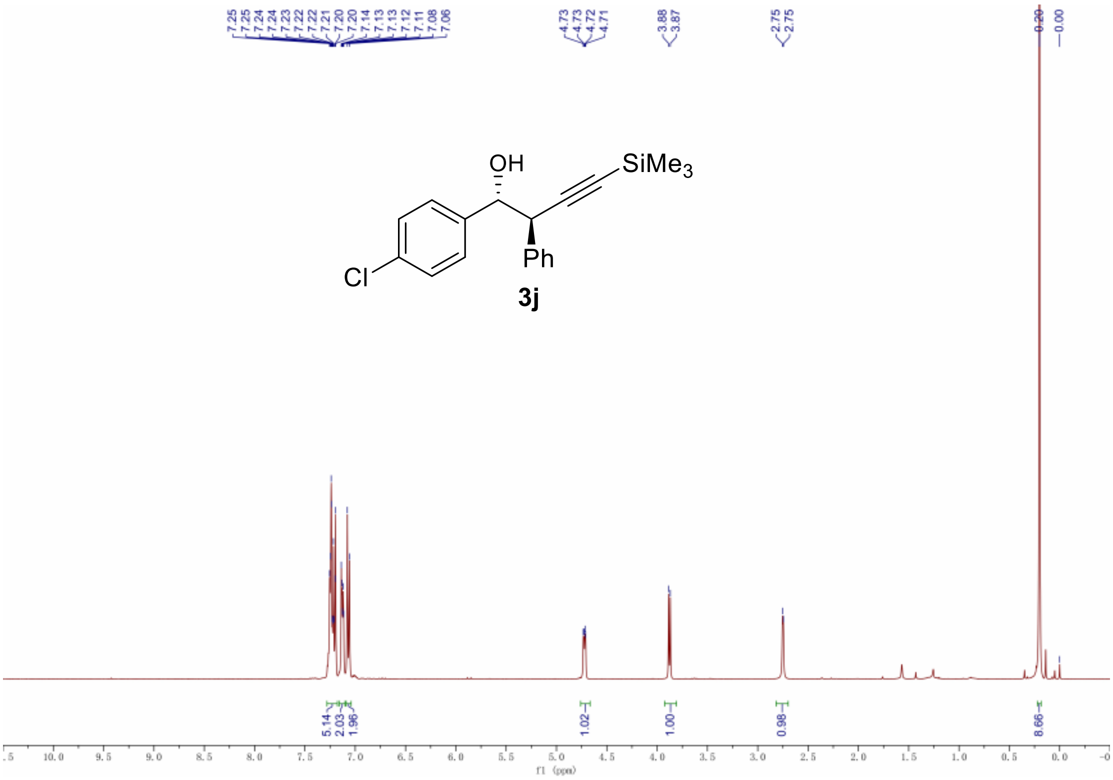

(¹³C NMR, 100 MHz, $CDCl_3$)

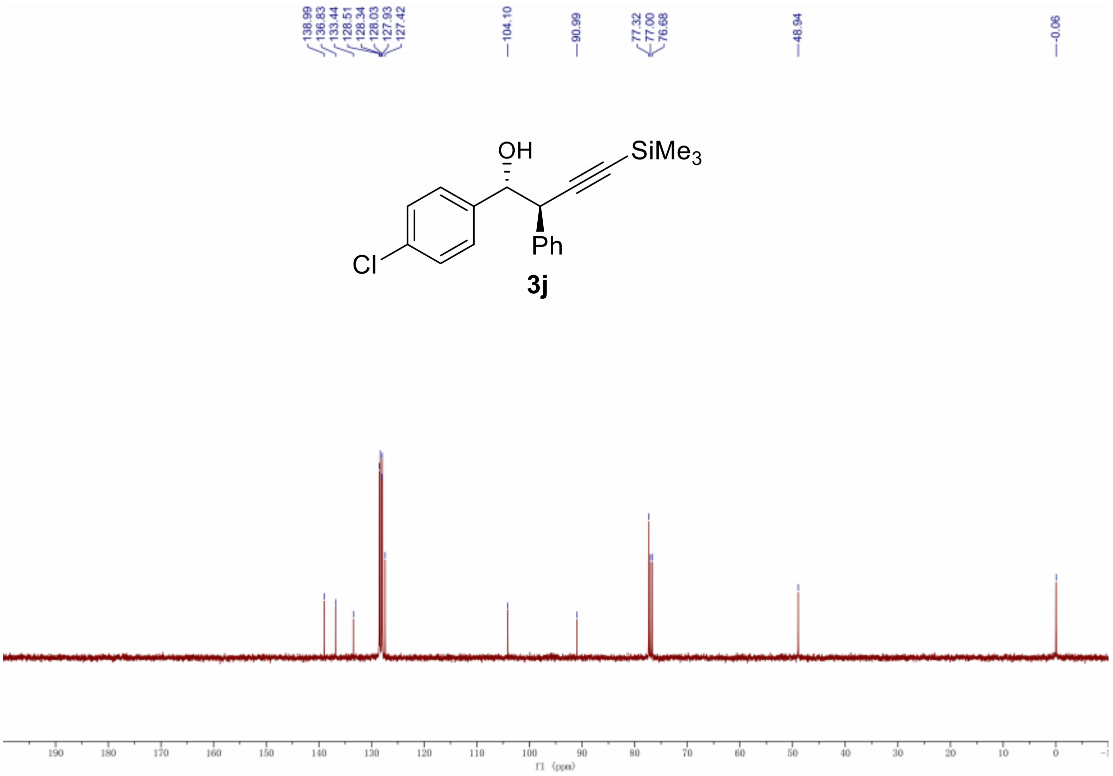

(^{1}H NMR, 400 MHz, $CDCl_3$)

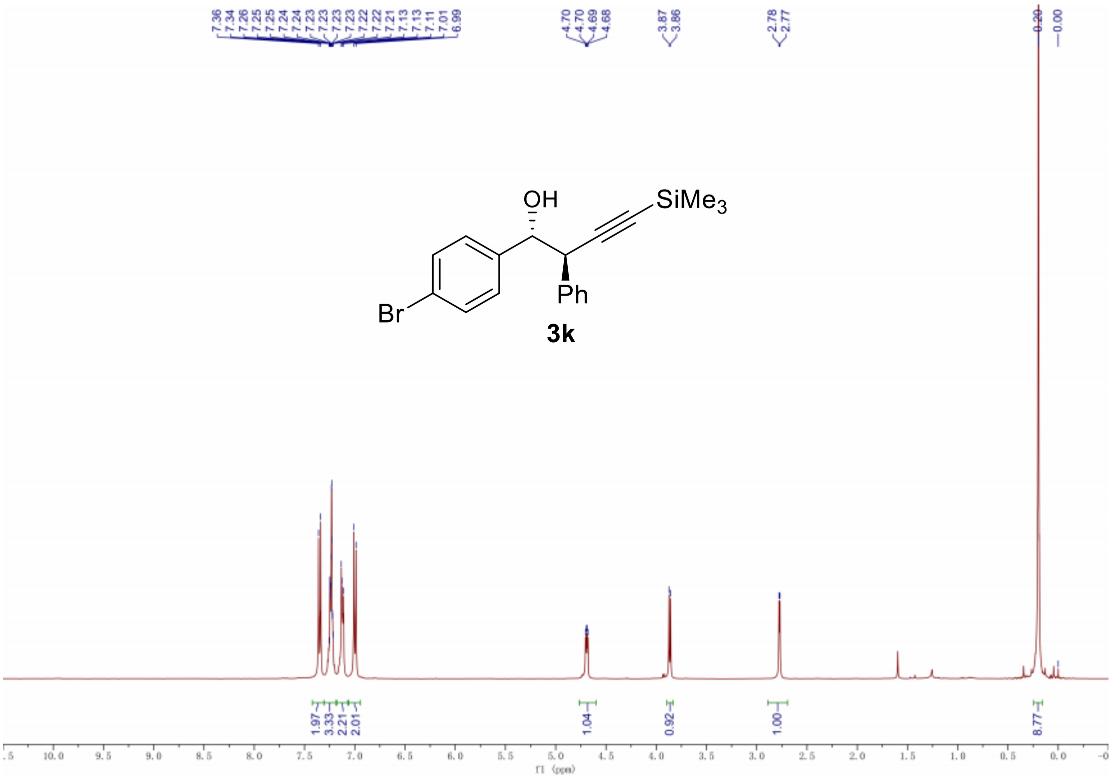

(^{13}C NMR, 100 MHz, $CDCl_3$)

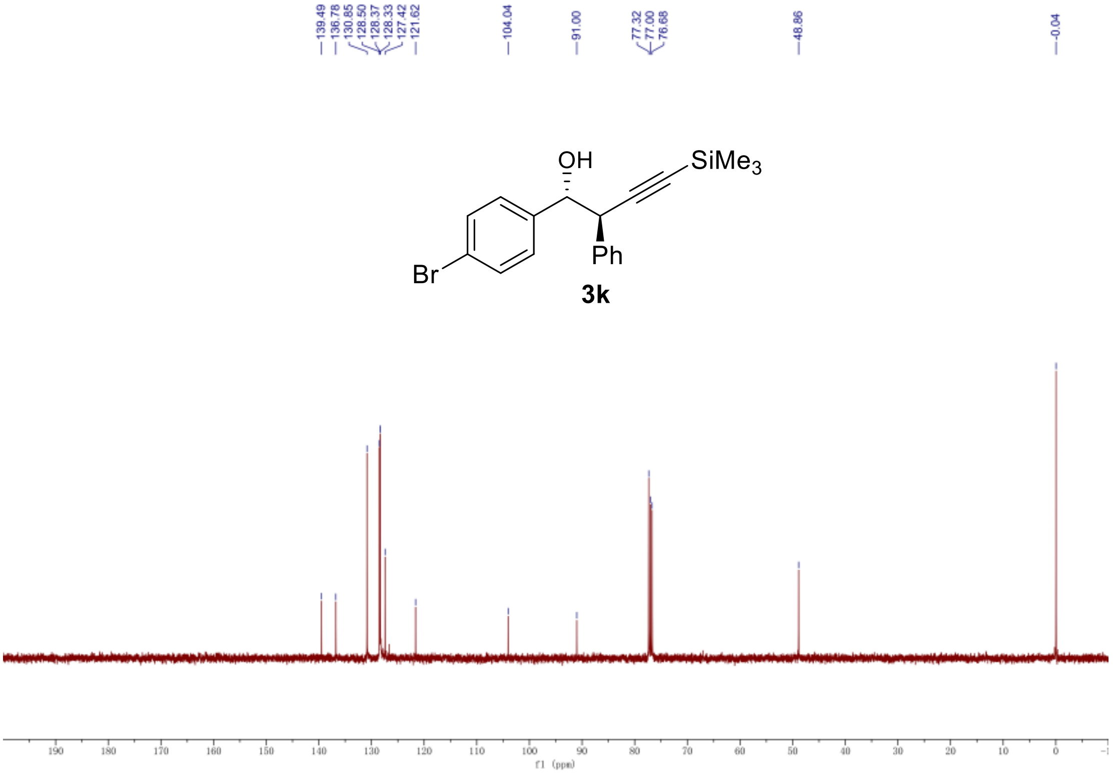

（^{1}H NMR, 400 MHz, $CDCl_3$）

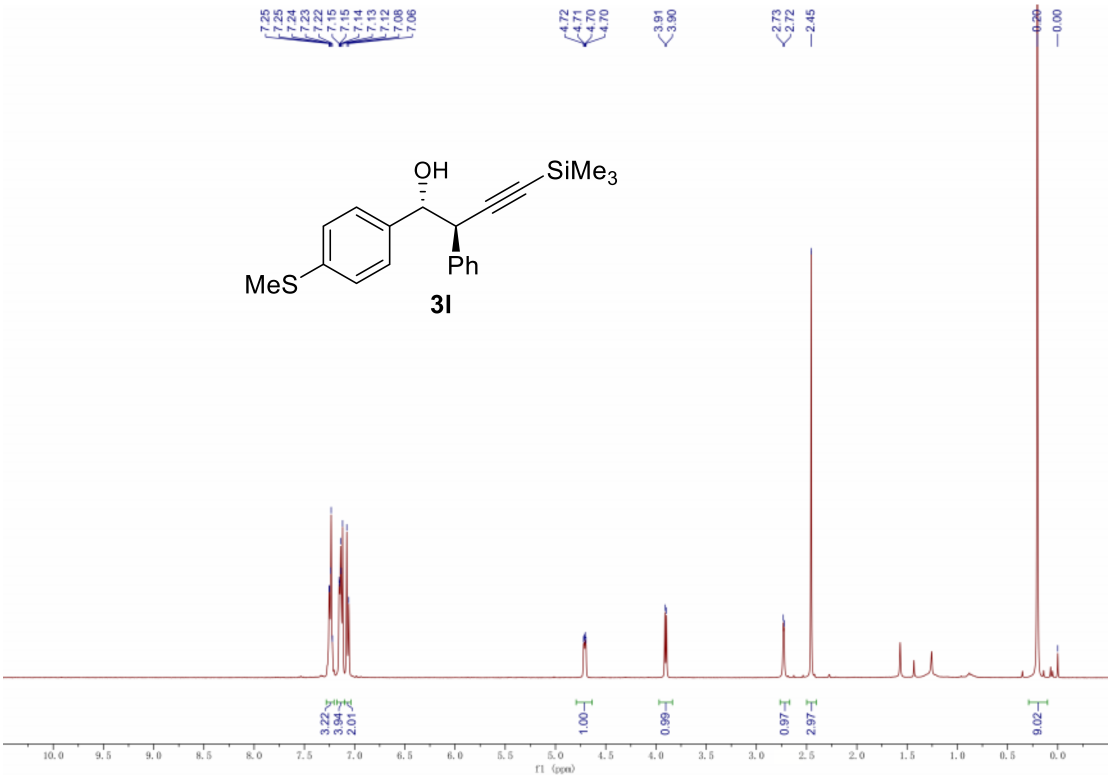

(^{13}C NMR, 100 MHz, $CDCl_3$)

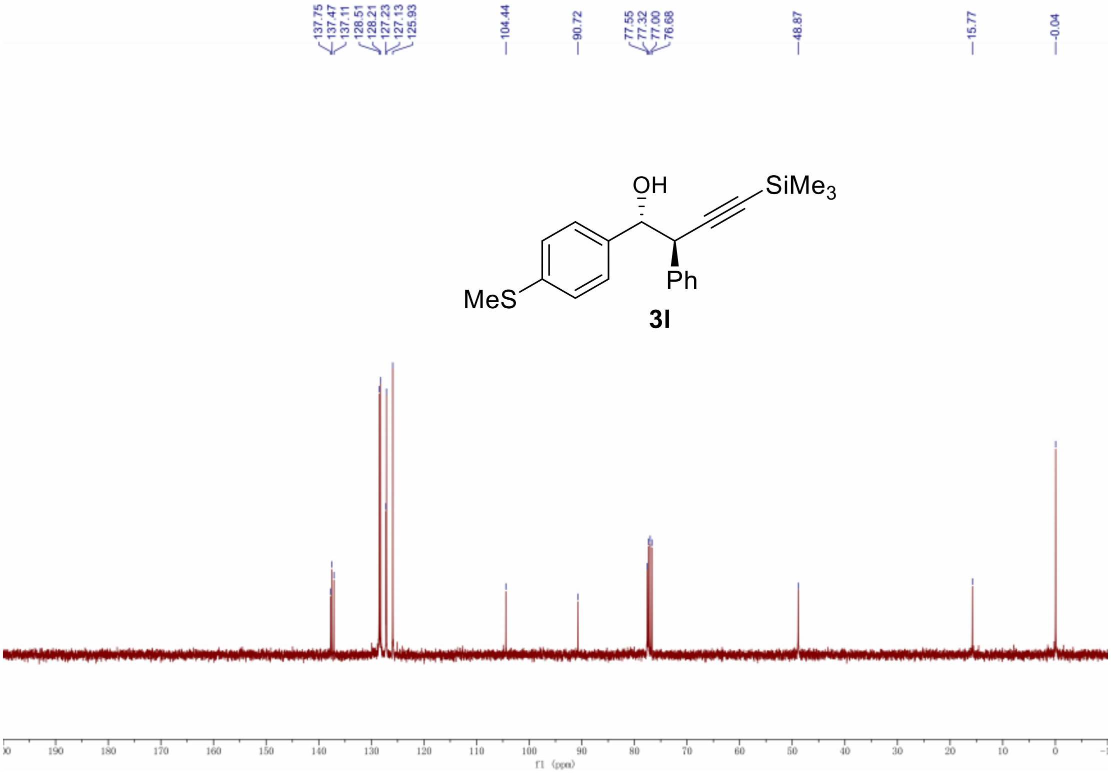

（^{1}H NMR, 400 MHz, $CDCl_3$）

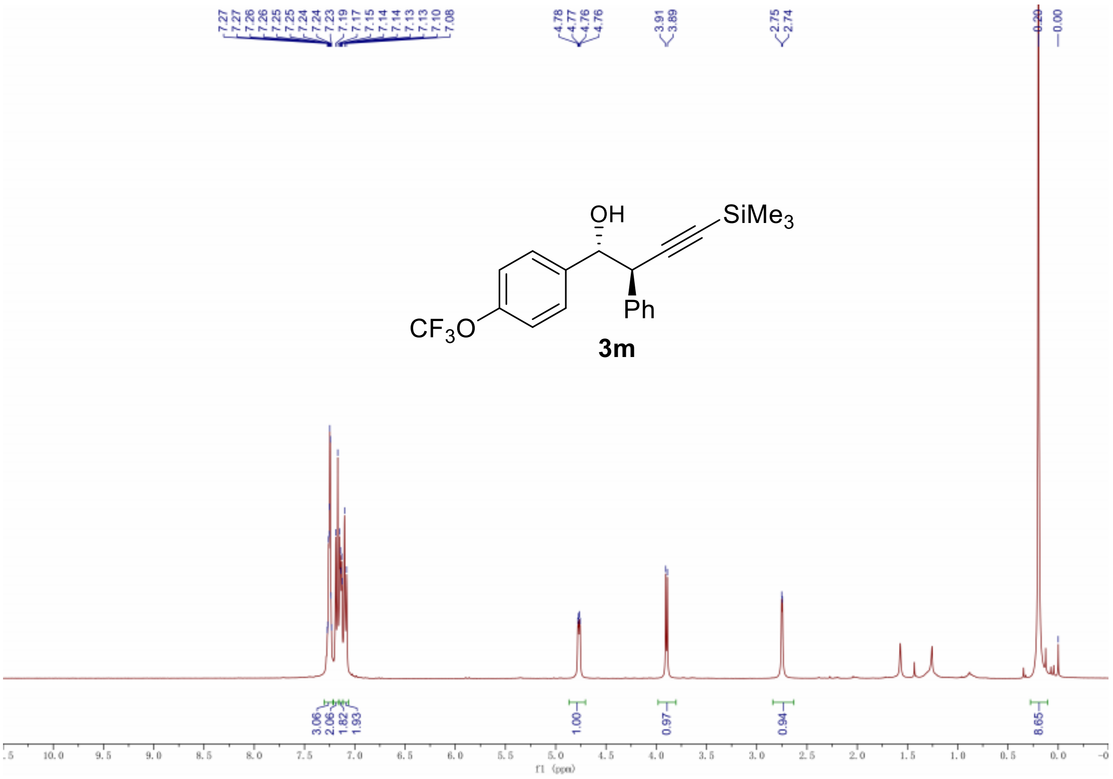

(^{13}C NMR, 100 MHz, $CDCl_3$)

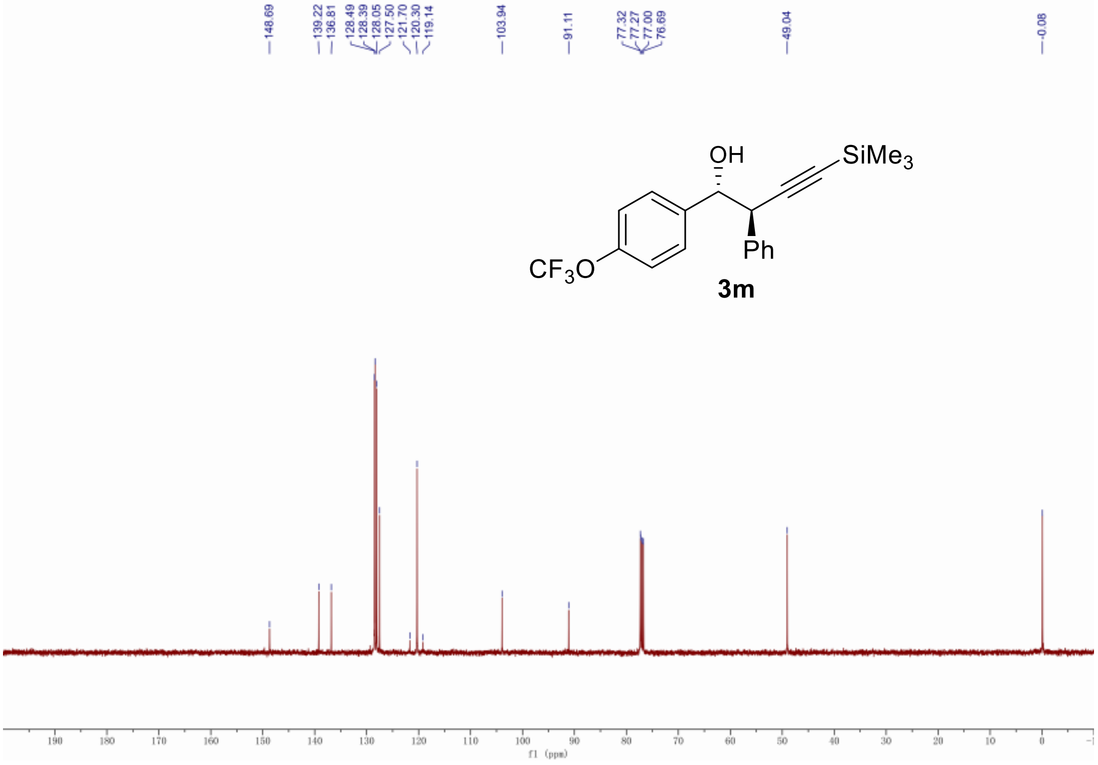

(^{19}F NMR, 376 MHz, CDCl$_3$)

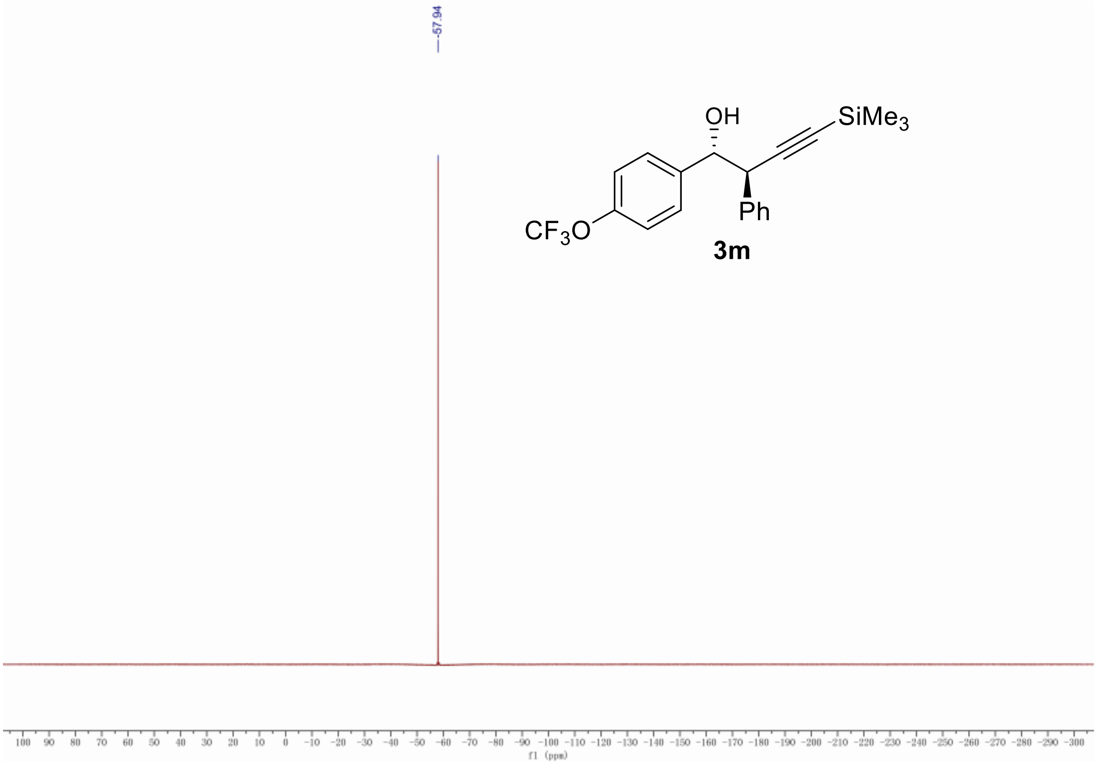

(^{1}H NMR, 400 MHz, CDCl$_3$)

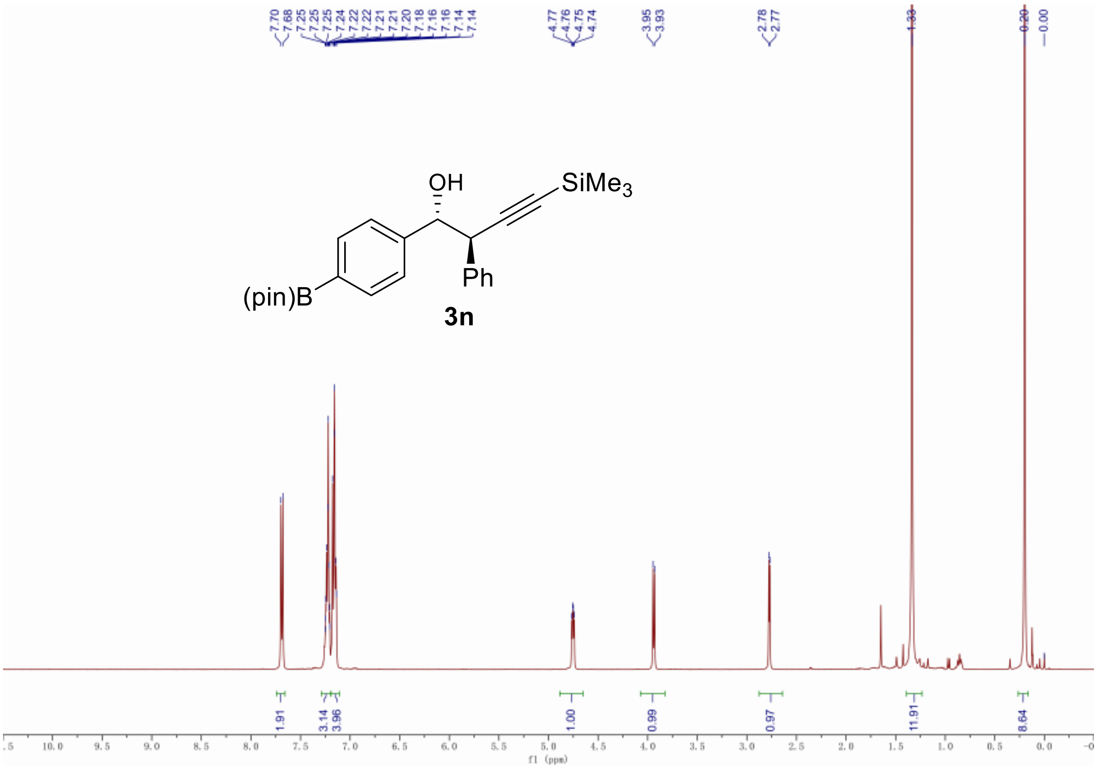

(^{13}C NMR, 100 MHz, CDCl$_3$)

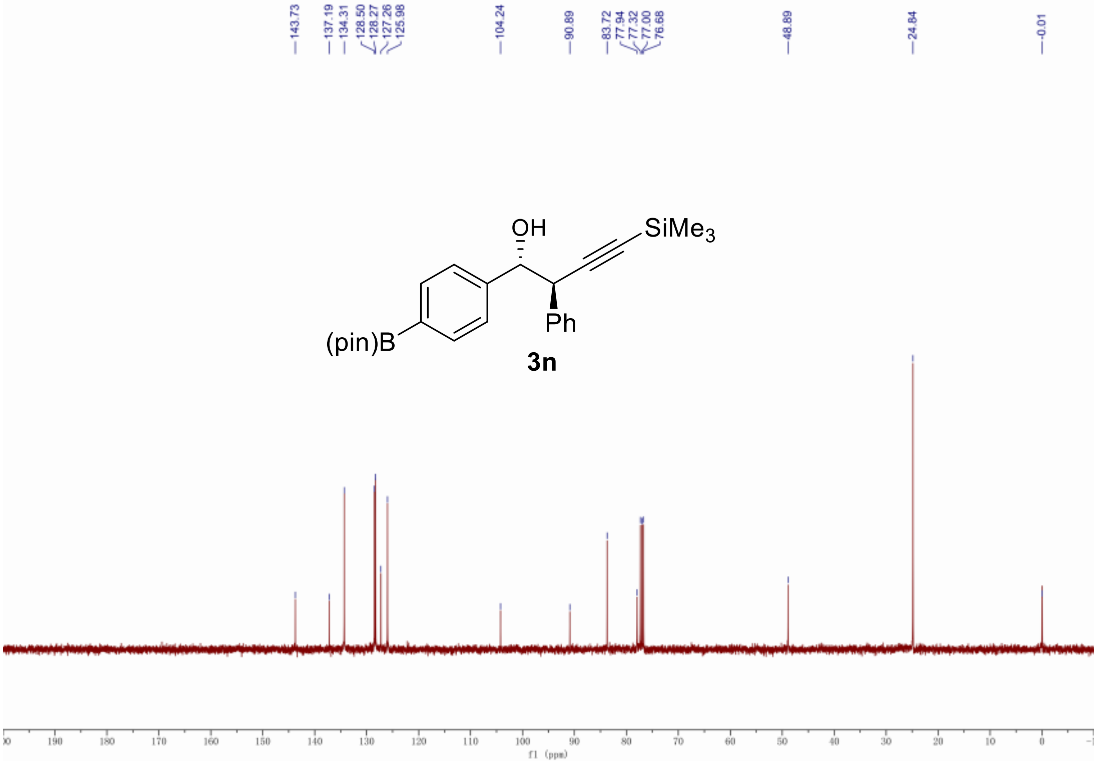

(^{1}H NMR, 400 MHz, CDCl$_3$)

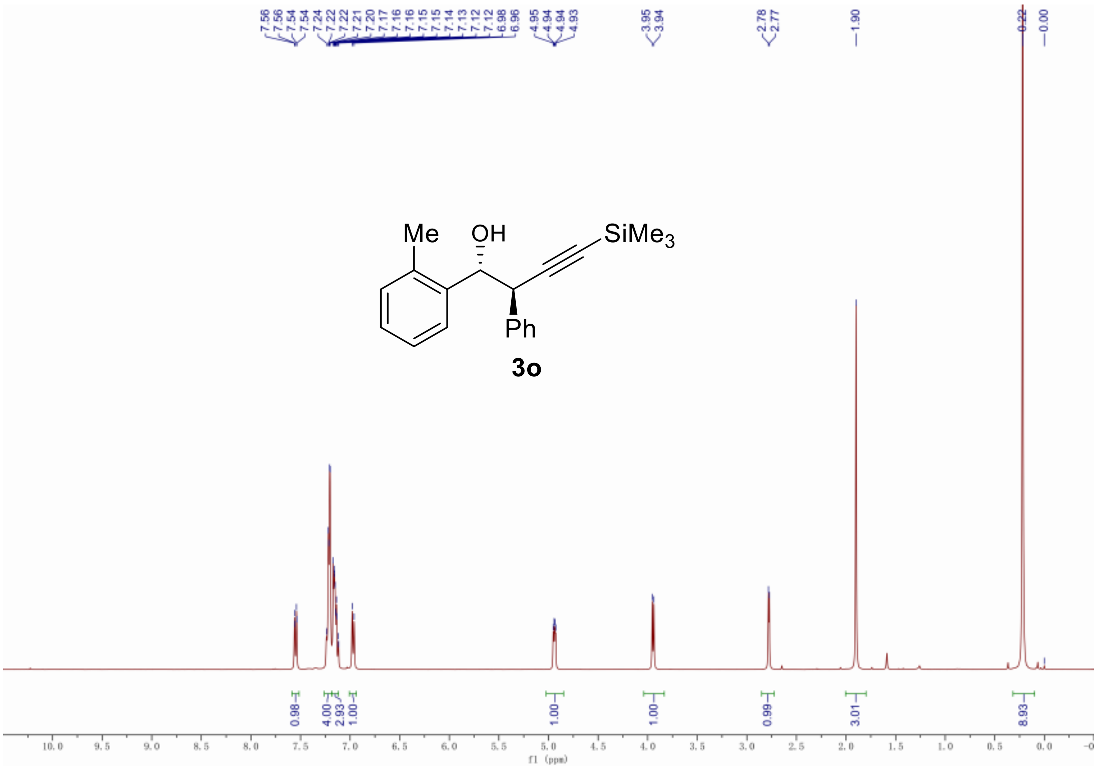

(^{13}C NMR, 100 MHz, $CDCl_3$)

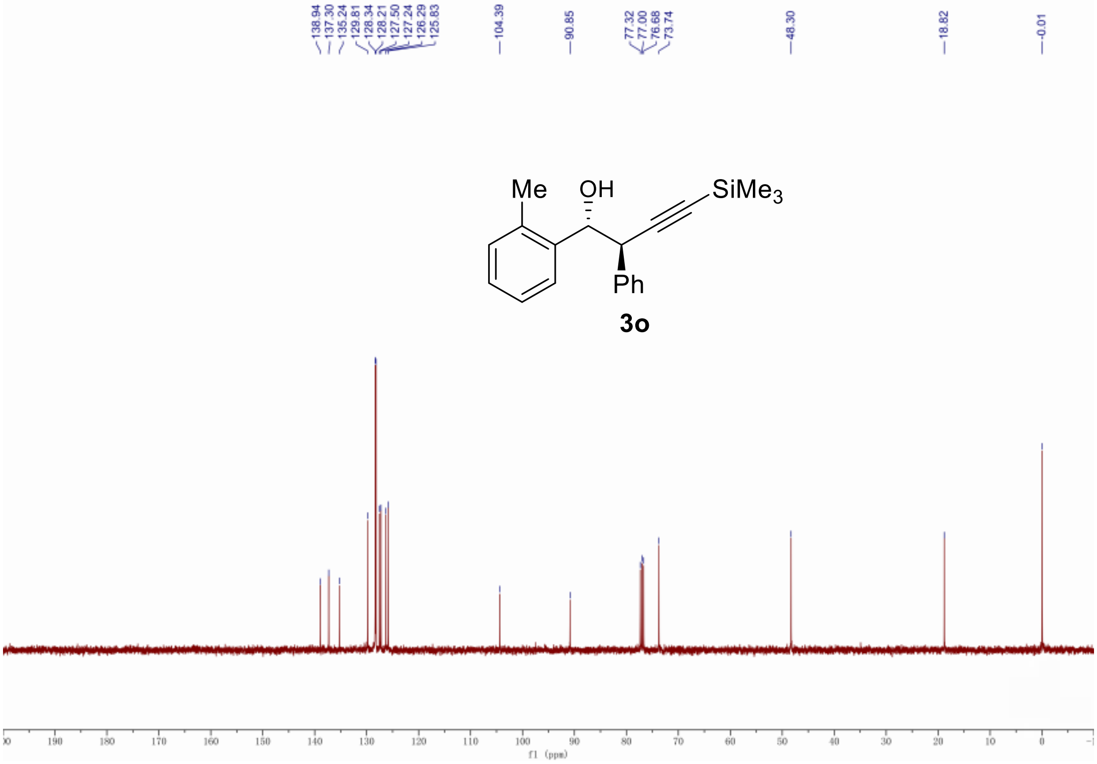

(^{1}H NMR, 400 MHz, $CDCl_3$)

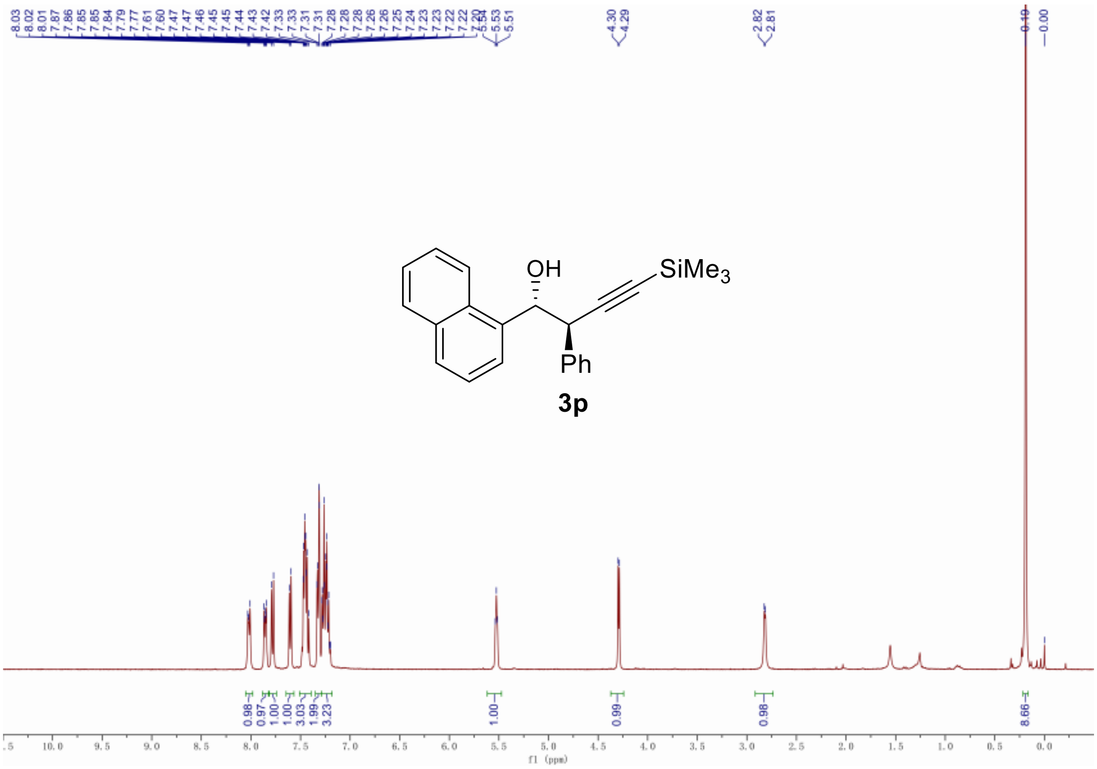

(^{13}C NMR, 100 MHz, CDCl$_3$)

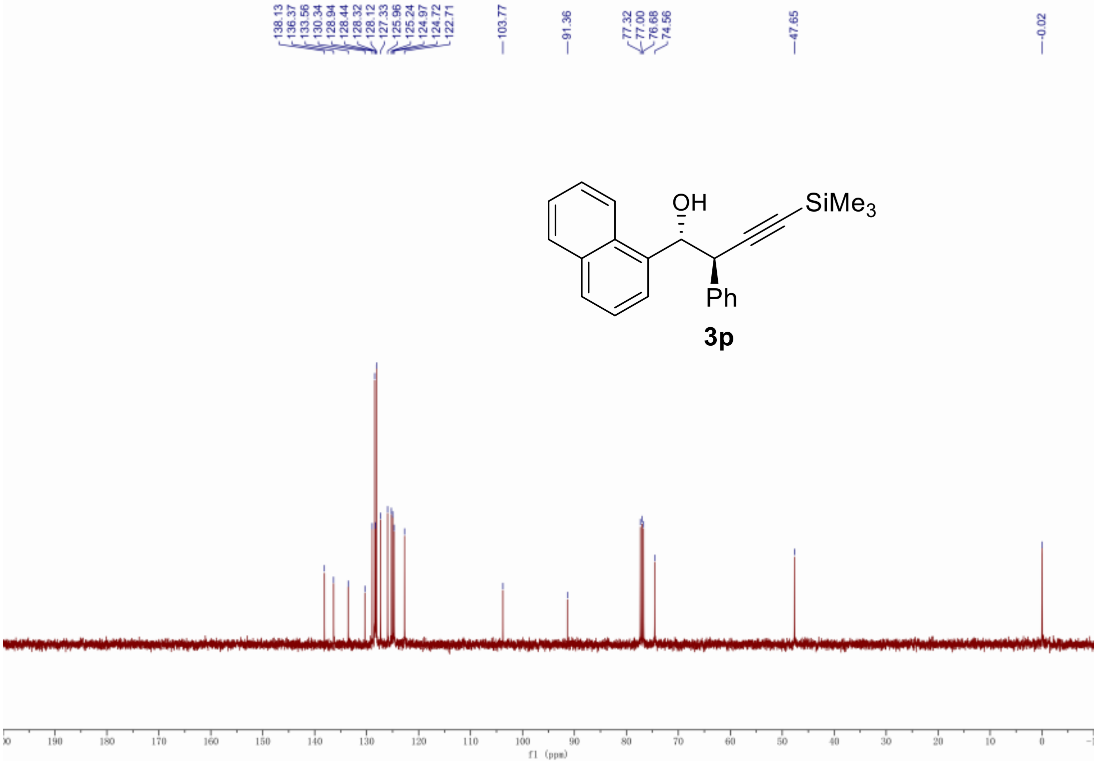

(^{1}H NMR, 400 MHz, CDCl$_3$)

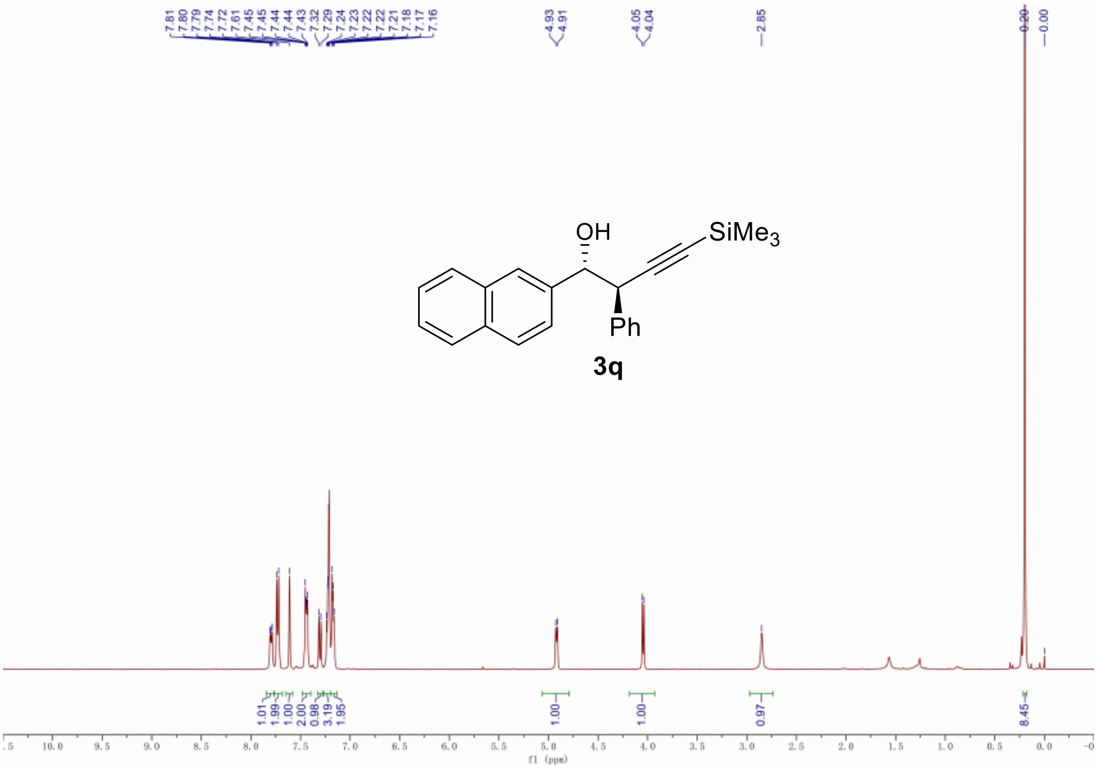

(^13C NMR, 100 MHz, CDCl3)

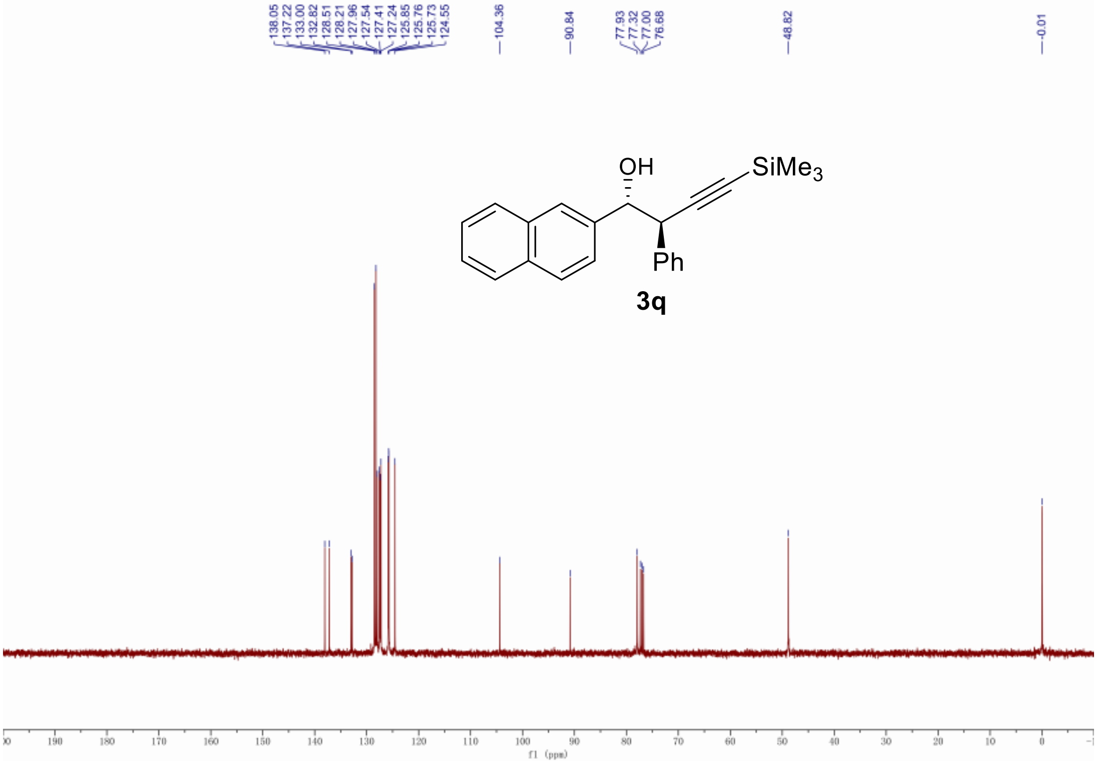

(^1H NMR, 400 MHz, CDCl3)

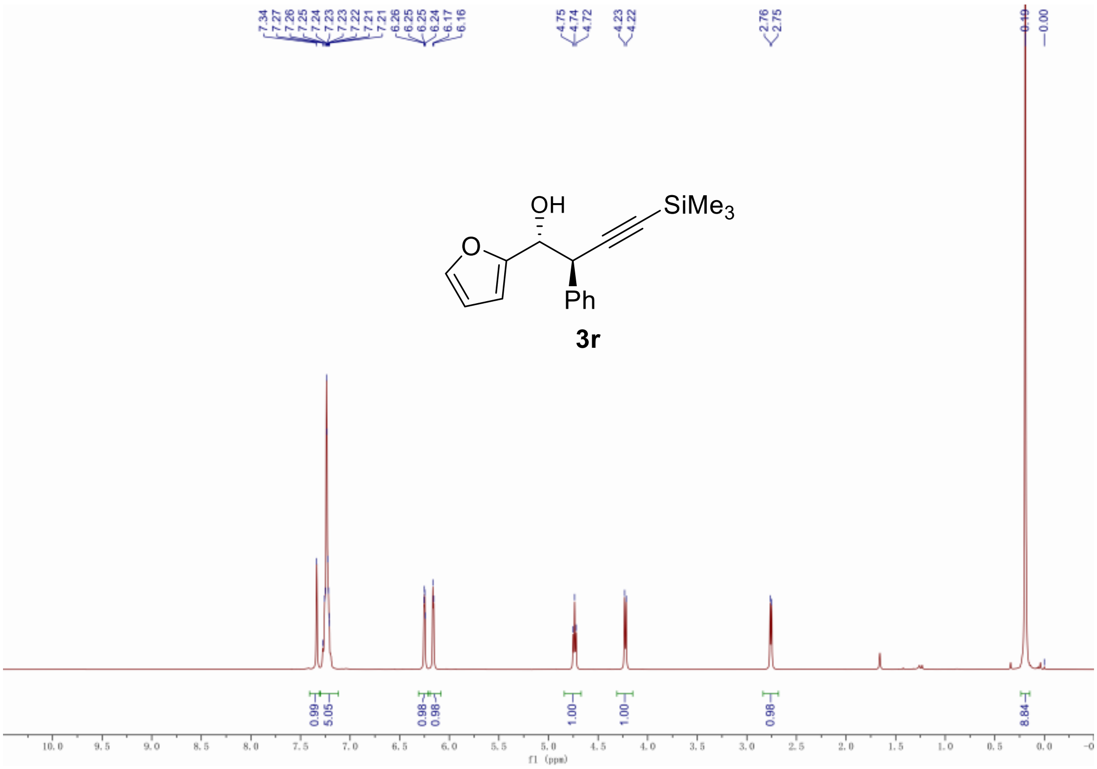

(^{13}C NMR, 100 MHz, CDCl$_3$)

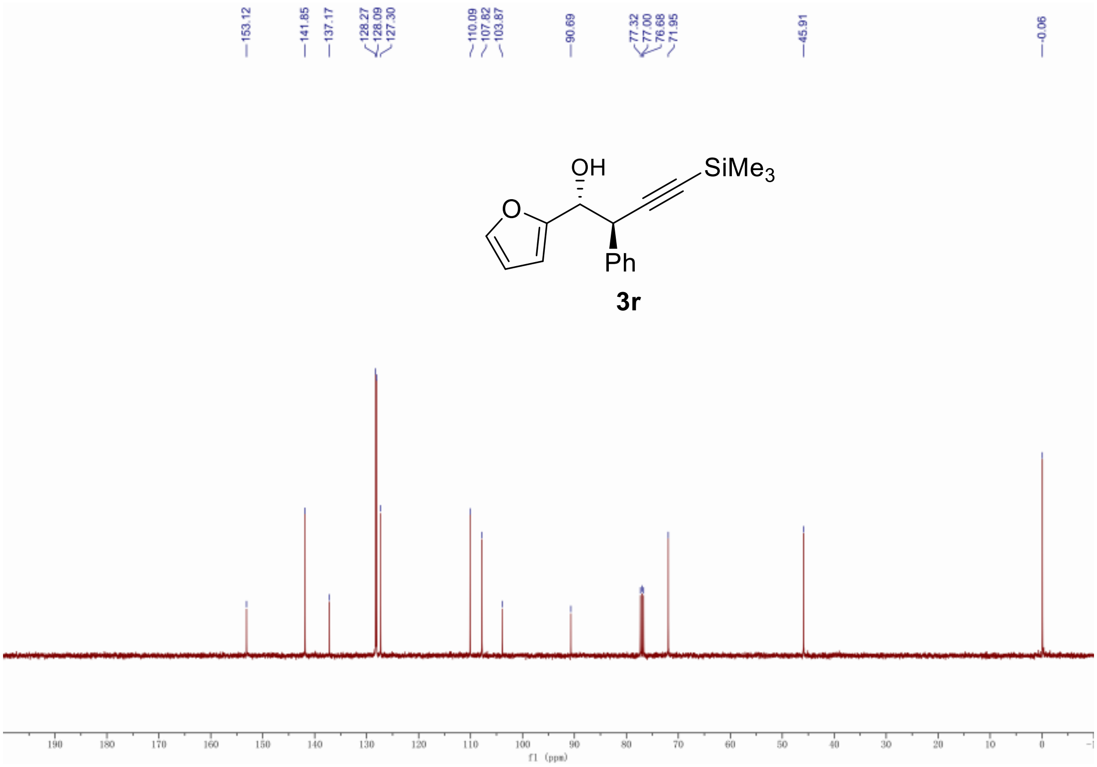

(^{1}H NMR, 400 MHz, CDCl$_3$)

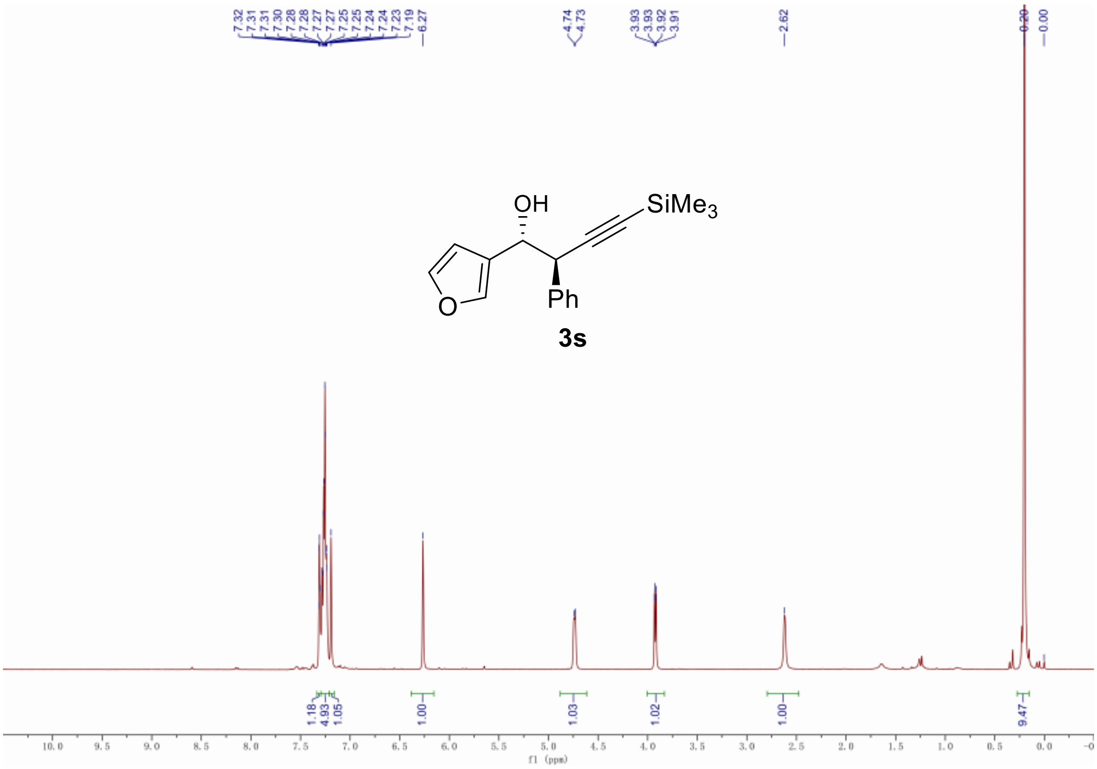

(^{13}C NMR, 100 MHz, CDCl$_3$)

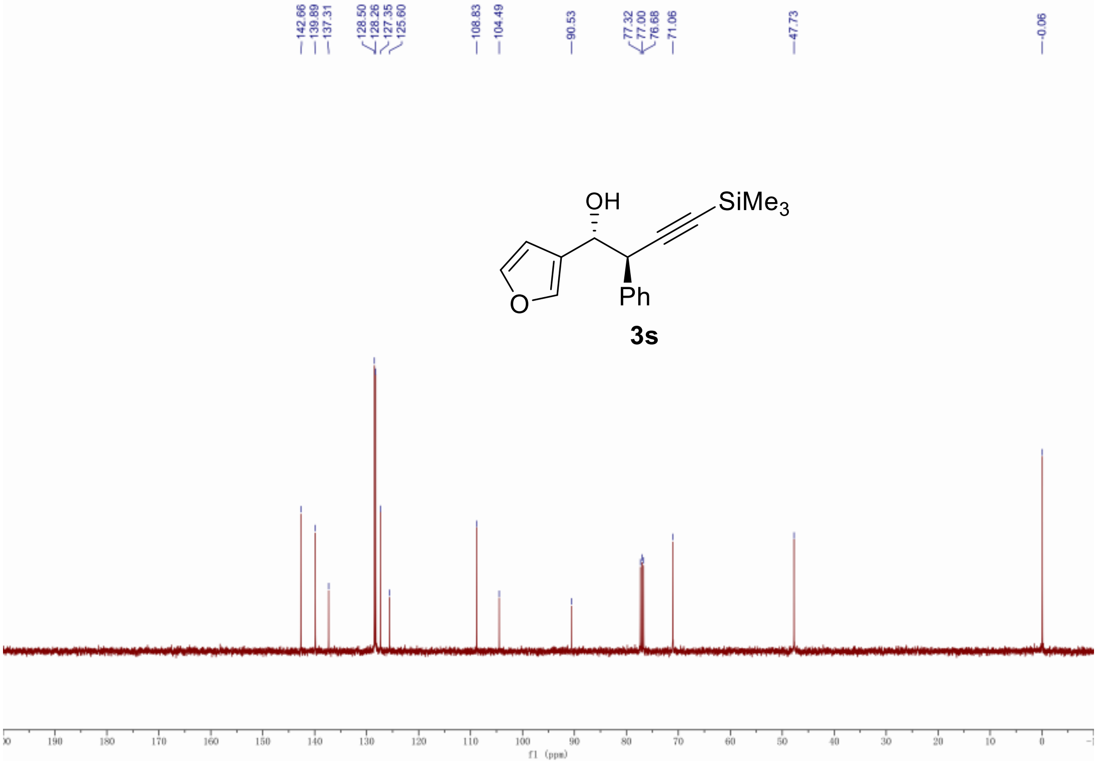

(^{1}H NMR, 400 MHz, CDCl$_3$)

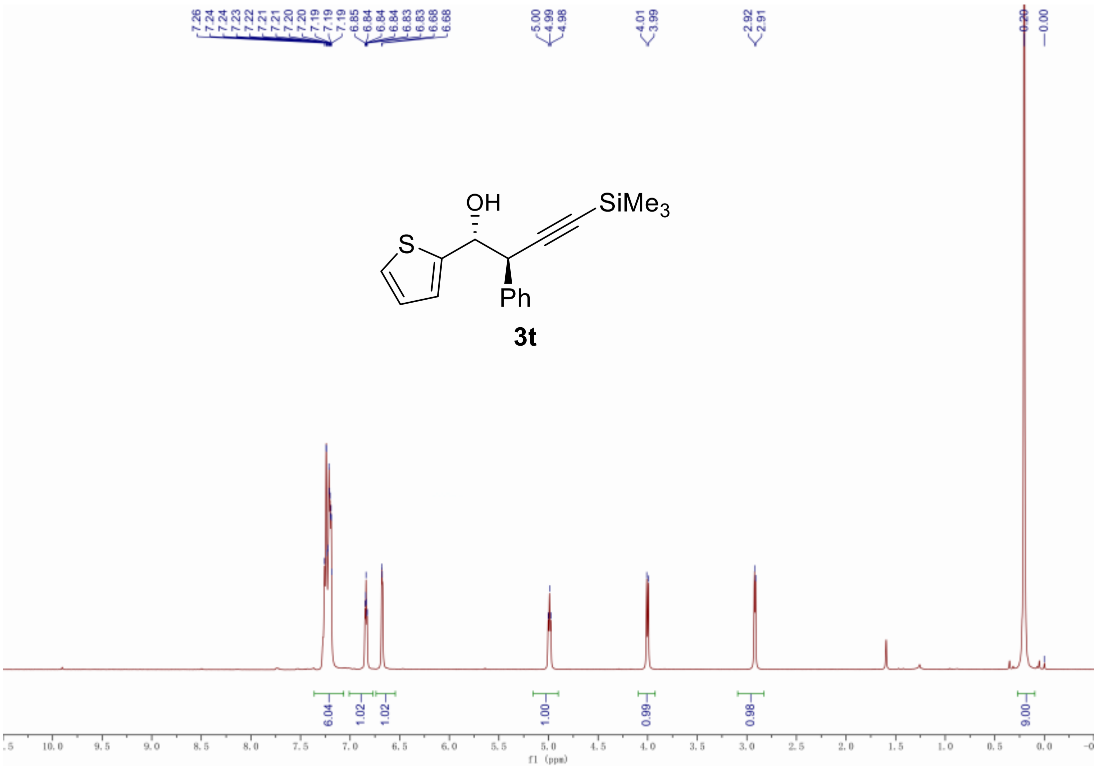

(^{13}C NMR, 100 MHz, $CDCl_3$)

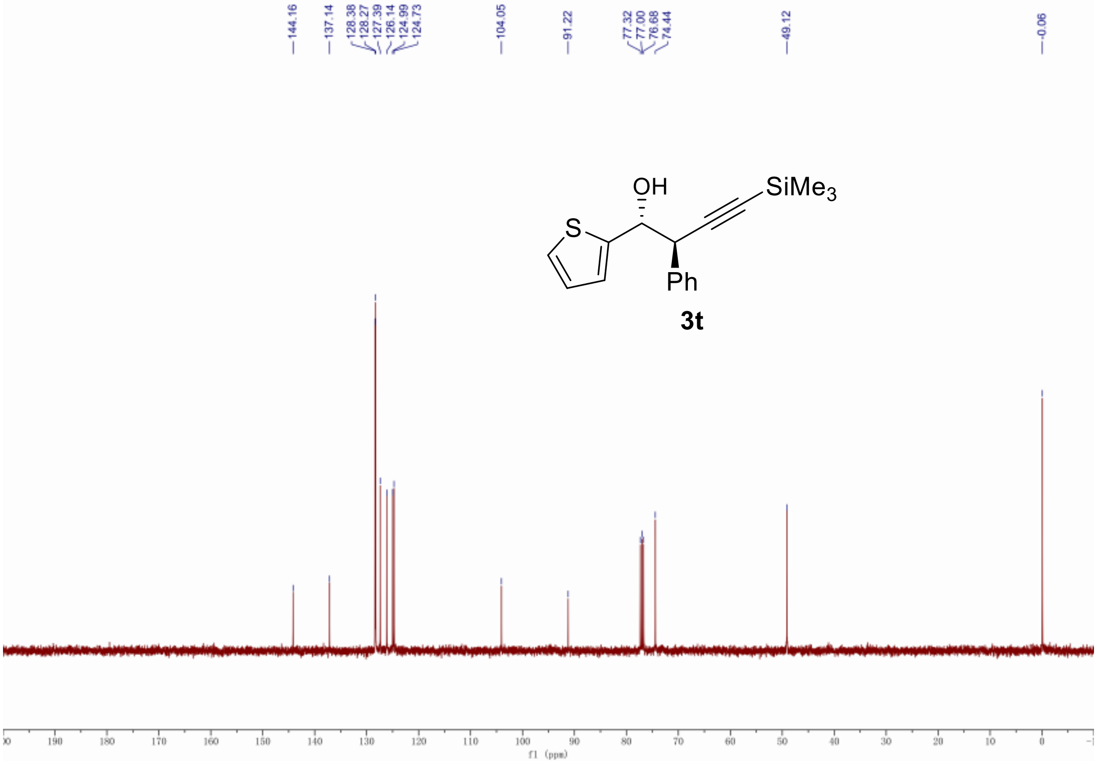

(^{1}H NMR, 400 MHz, $CDCl_3$)

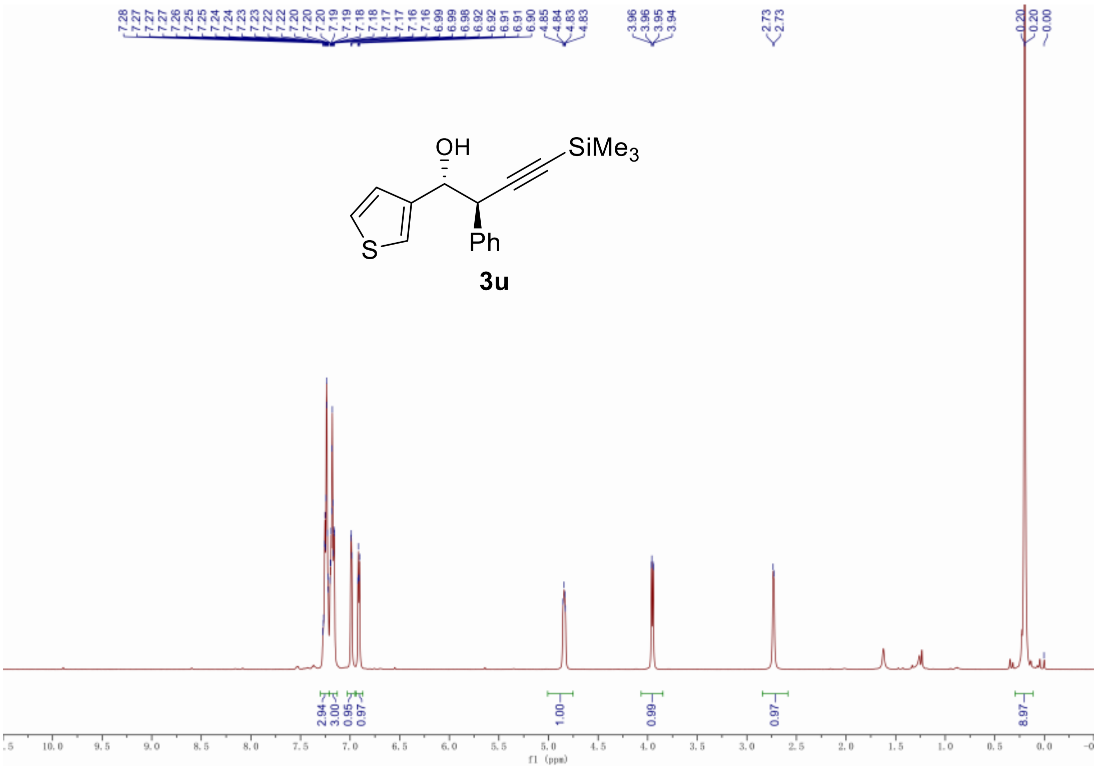

(^{13}C NMR, 100 MHz, CDCl$_3$)

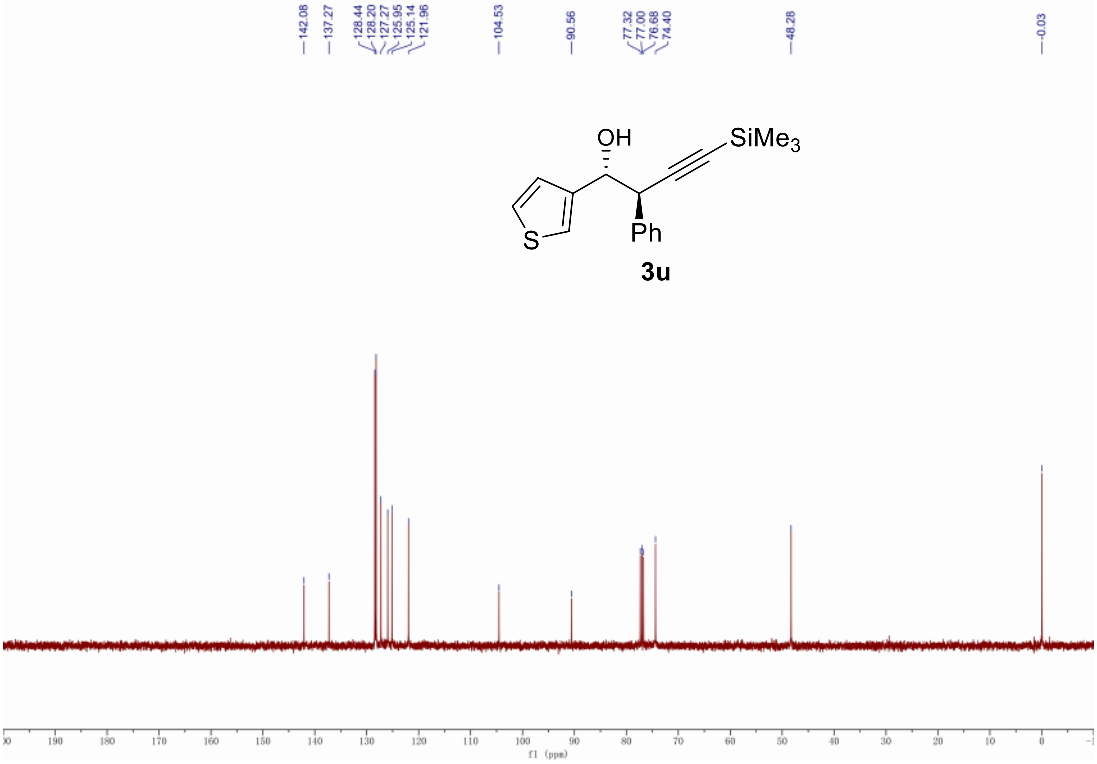

(^{1}H NMR, 400 MHz, CDCl$_3$)

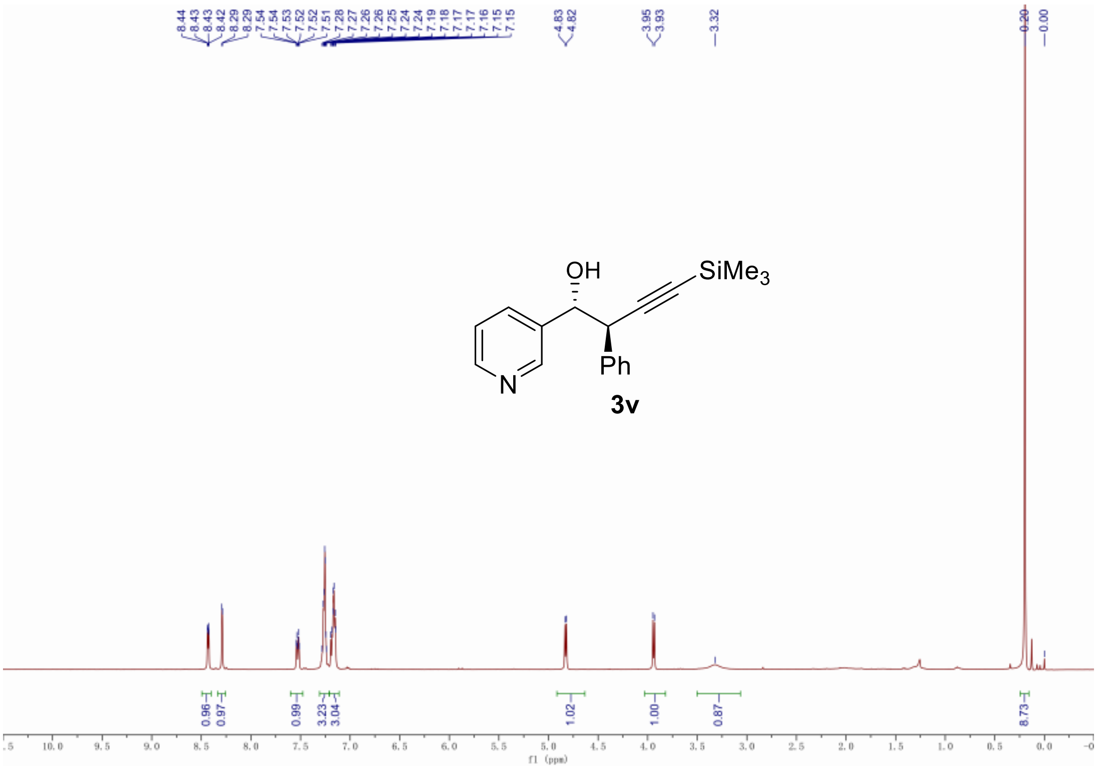

(^{13}C NMR, 100 MHz, $CDCl_3$)

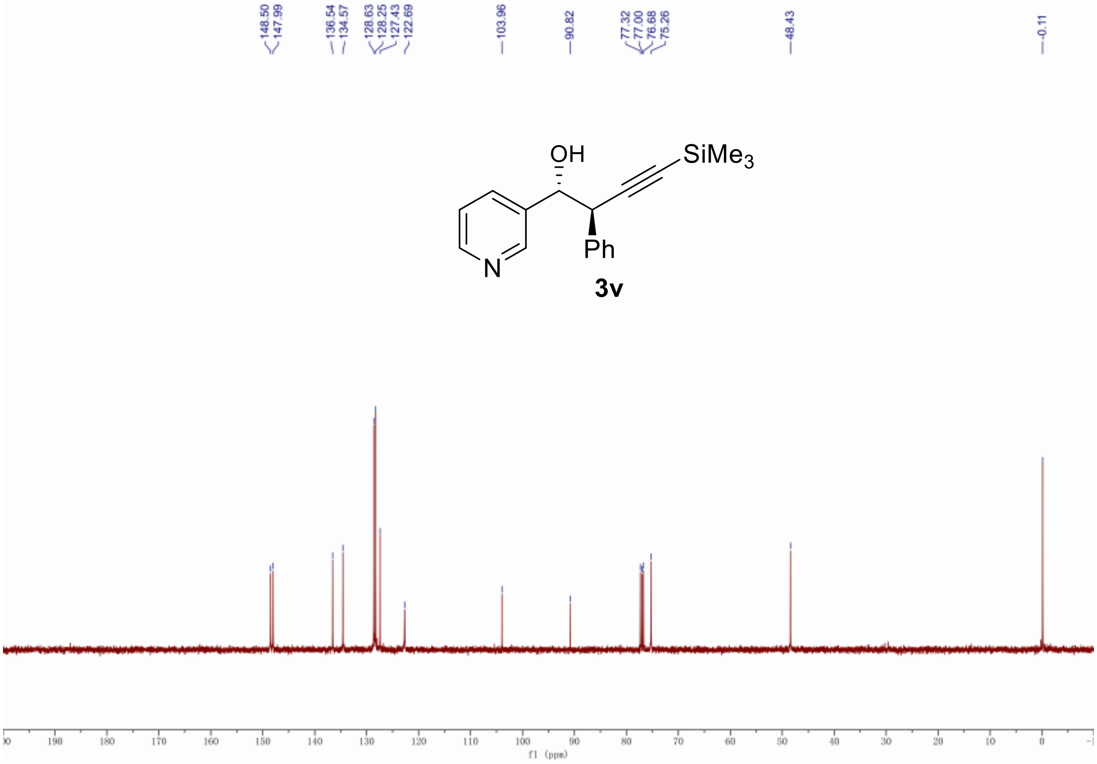

(^{1}H NMR, 400 MHz, $CDCl_3$)

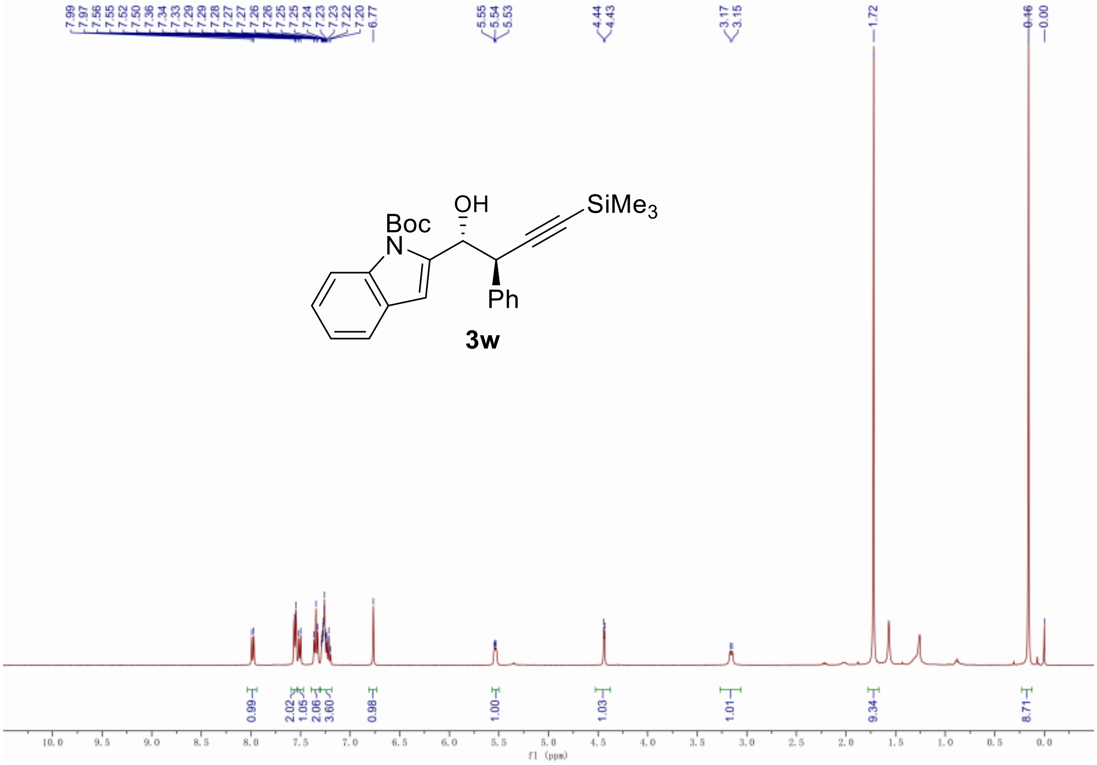

(^{13}C NMR, 100 MHz, $CDCl_3$)

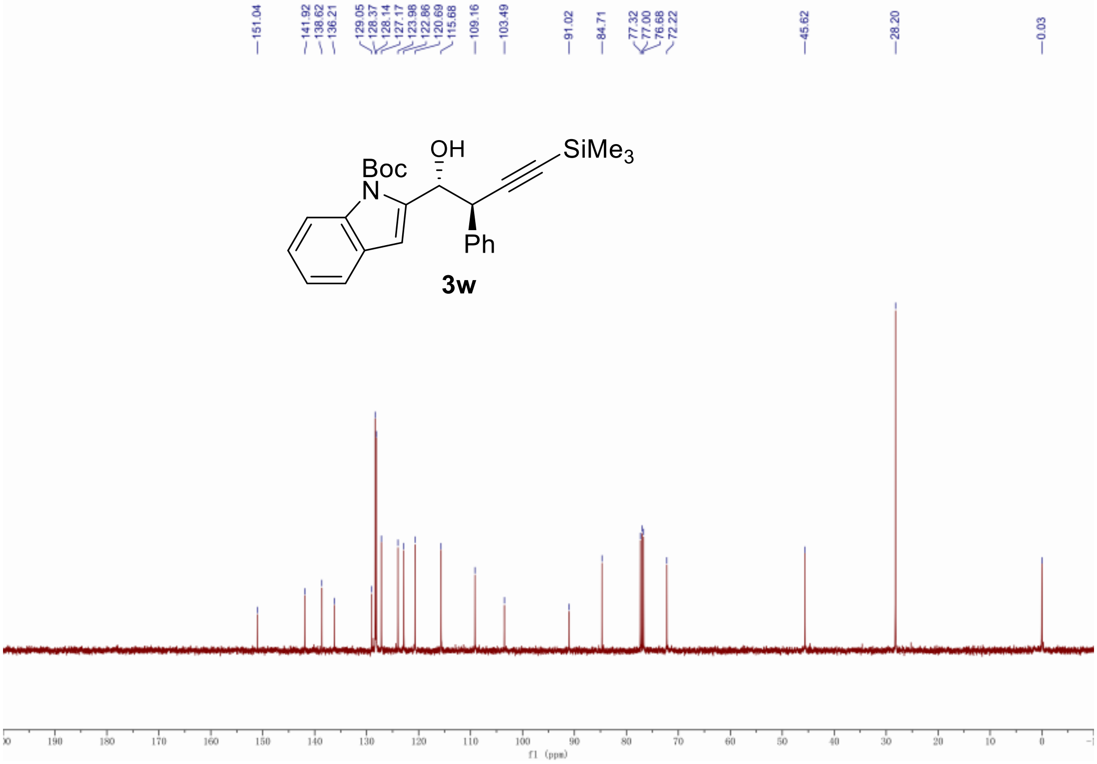

(^{1}H NMR, 400 MHz, $CDCl_3$)

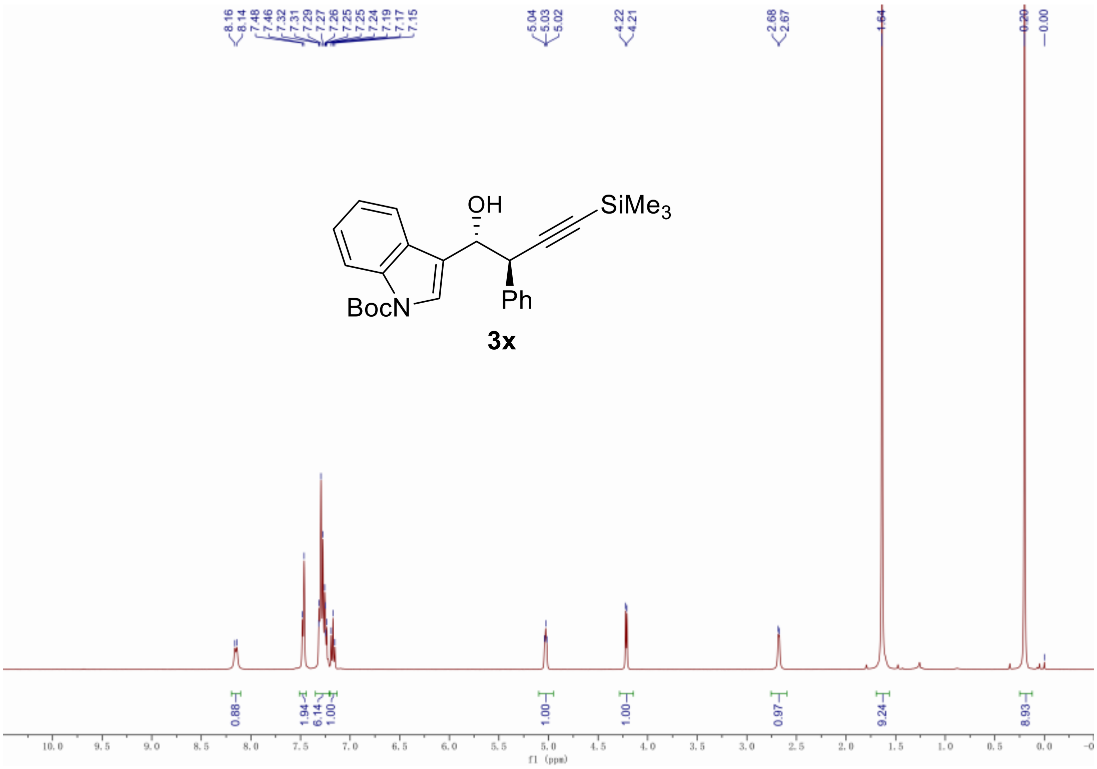

(^{13}C NMR, 100 MHz, CDCl$_3$)

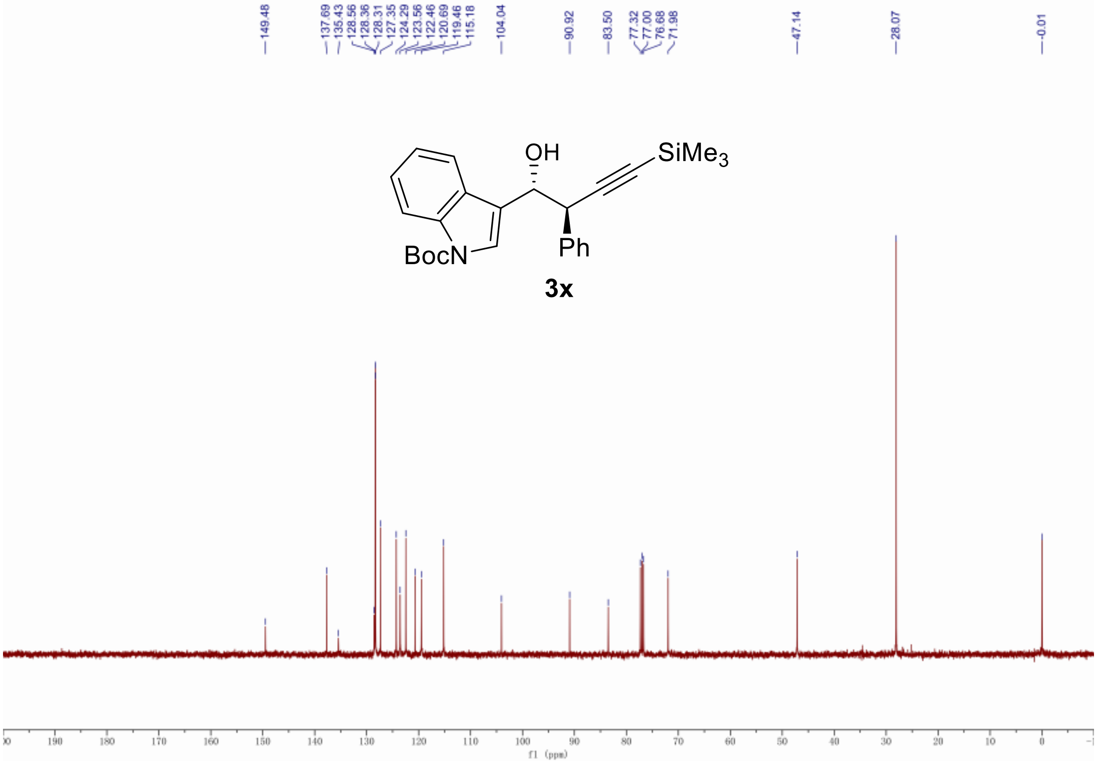

（^{1}H NMR, 400 MHz, CDCl$_3$）

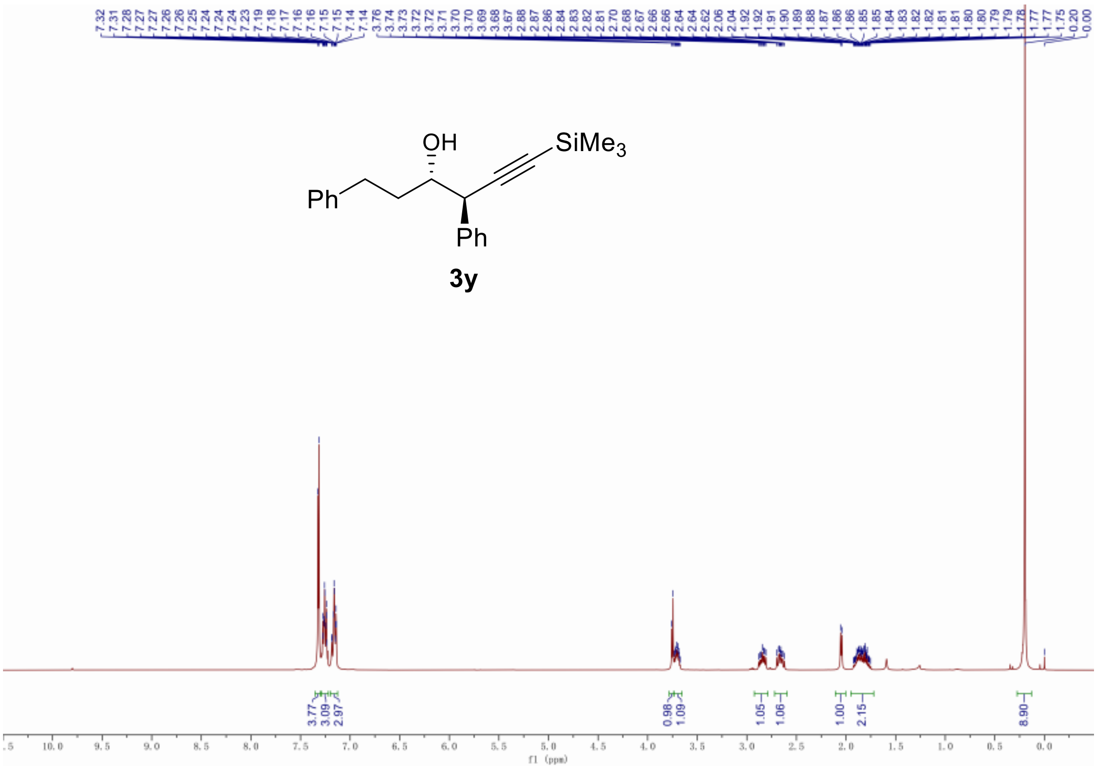

(^{13}C NMR, 100 MHz, $CDCl_3$)

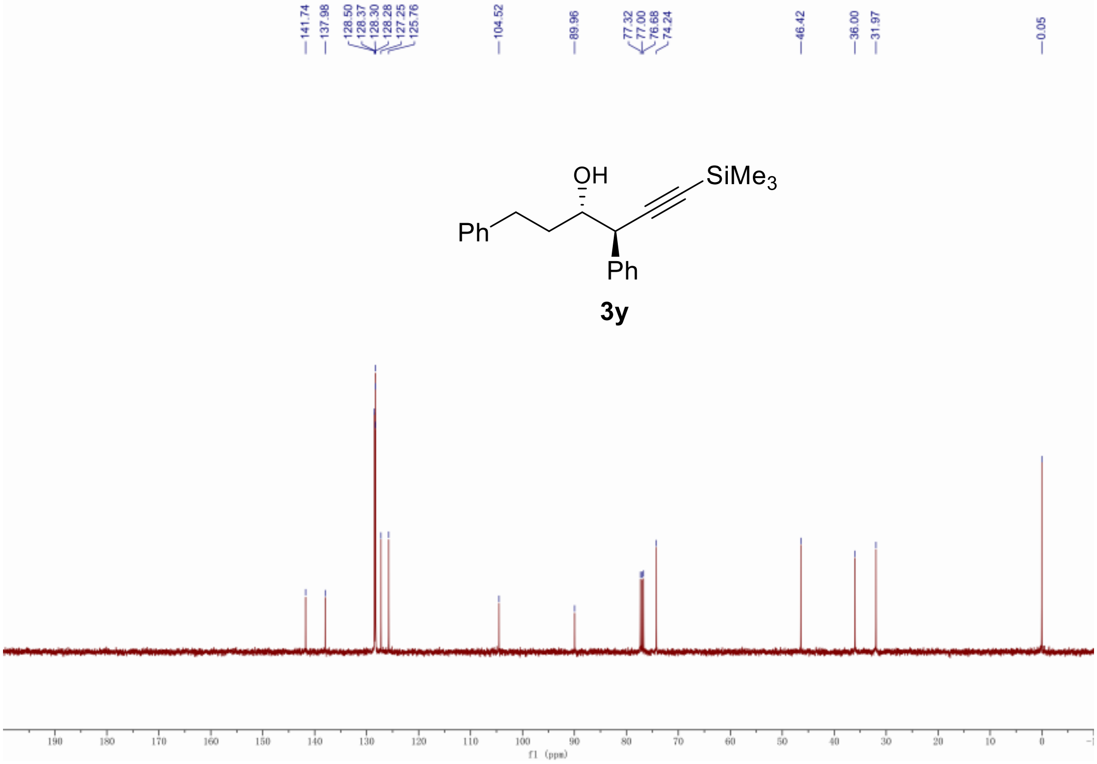

（^{1}H NMR, 400 MHz, $CDCl_3$）

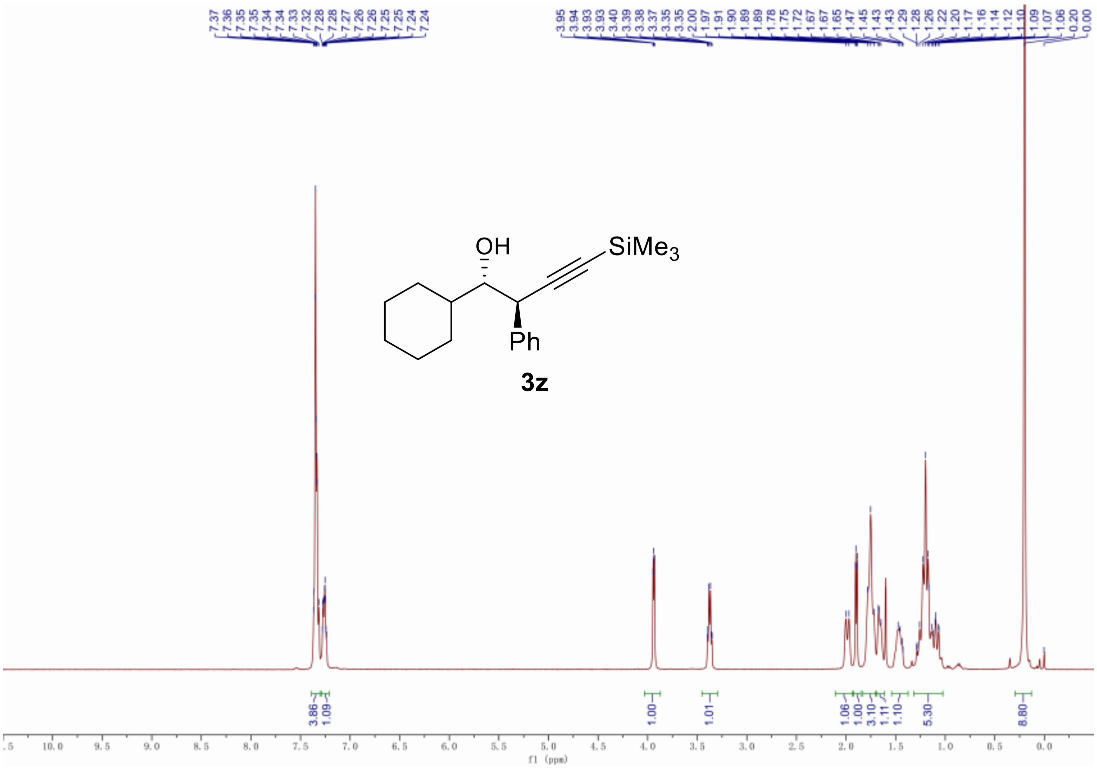

(^{13}C NMR, 100 MHz, $CDCl_3$)

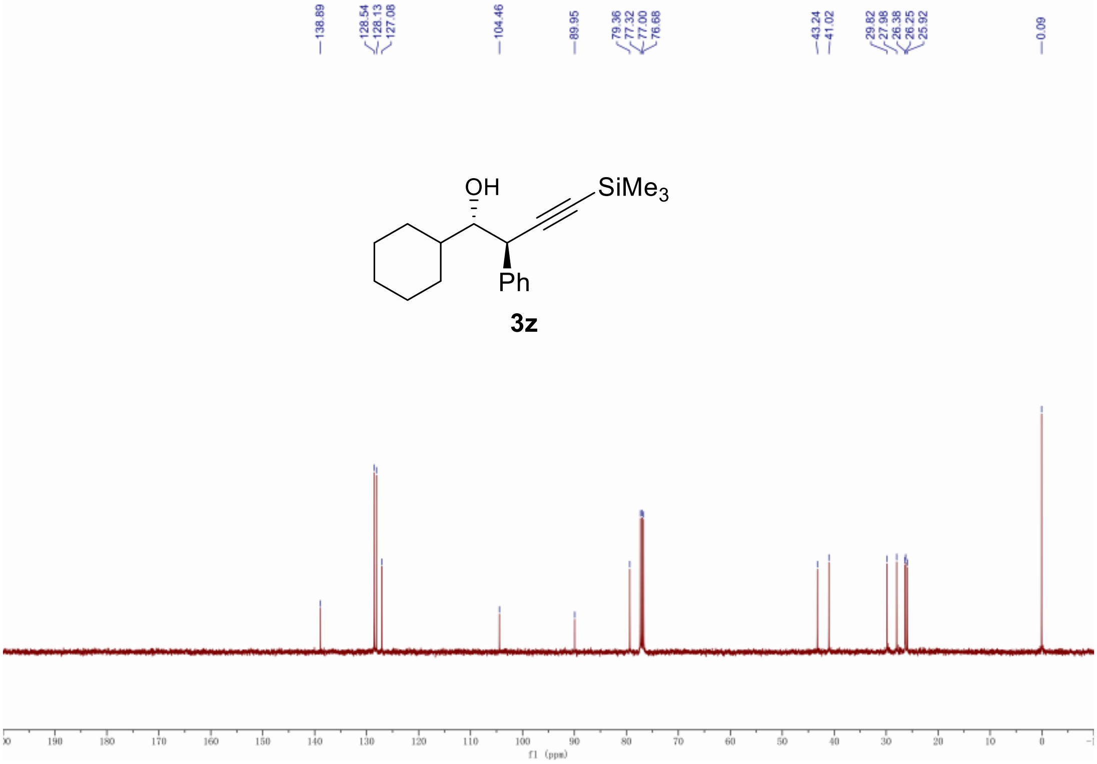

(^{1}H NMR, 400 MHz, $CDCl_3$)

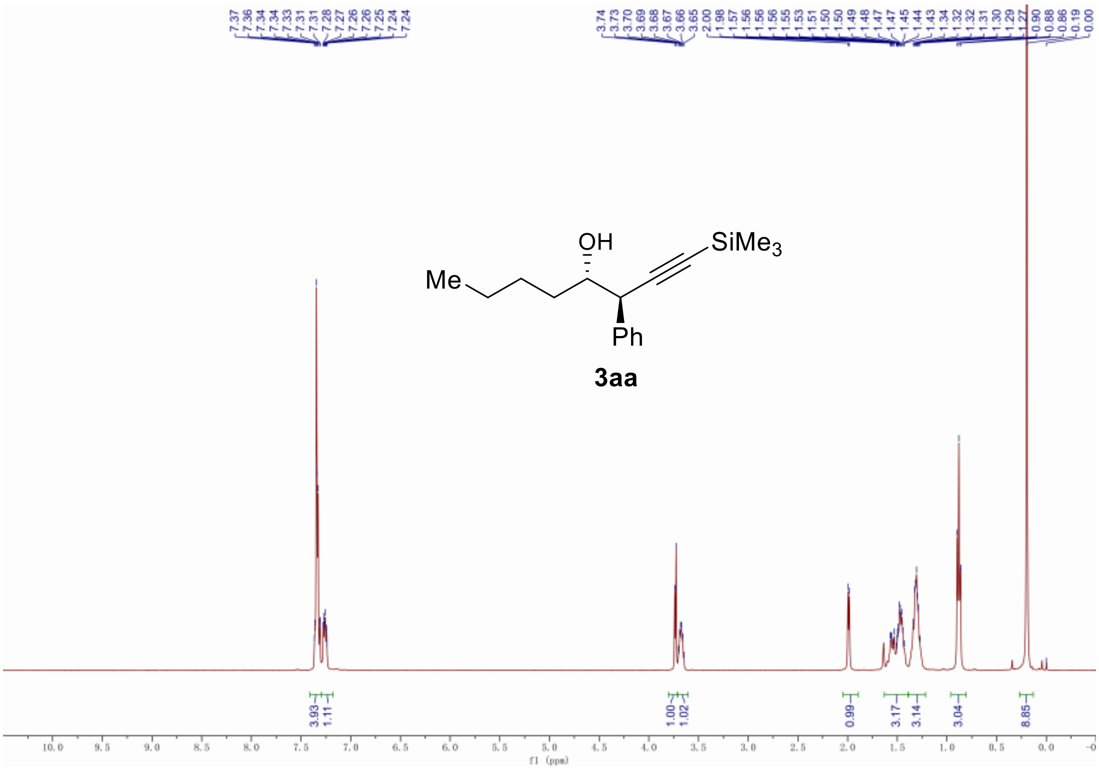

(^{13}C NMR, 100 MHz, CDCl$_3$)

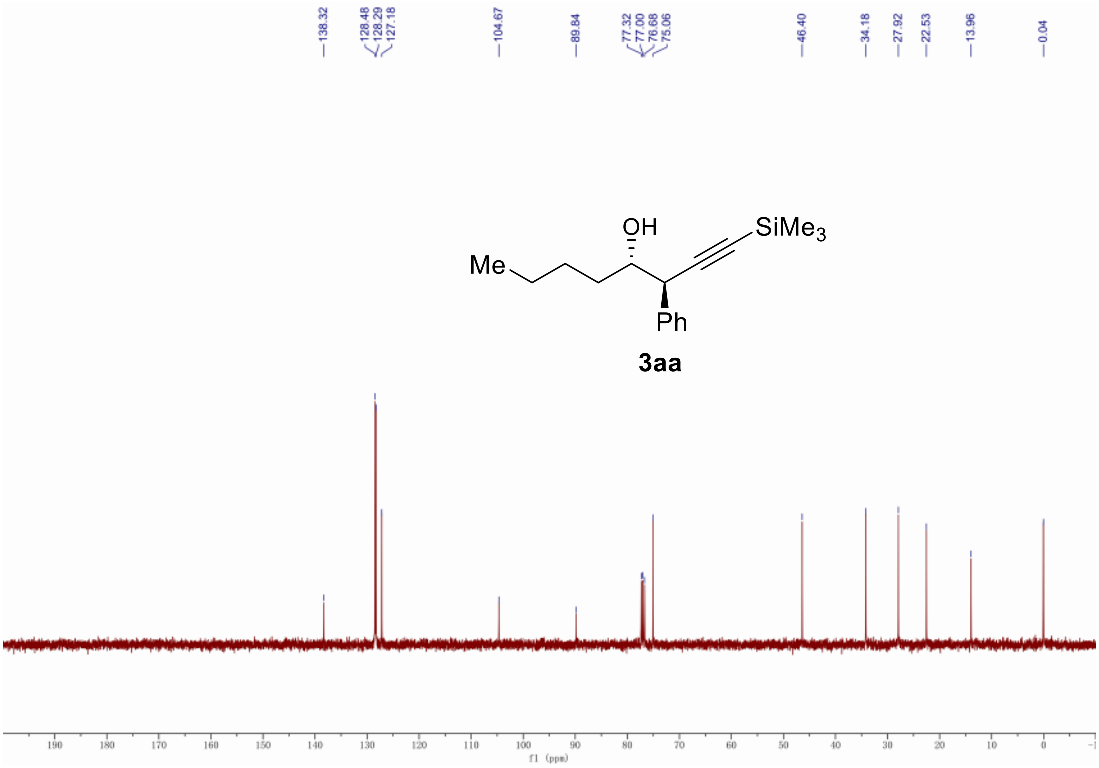

(^{1}H NMR, 400 MHz, CDCl$_3$)

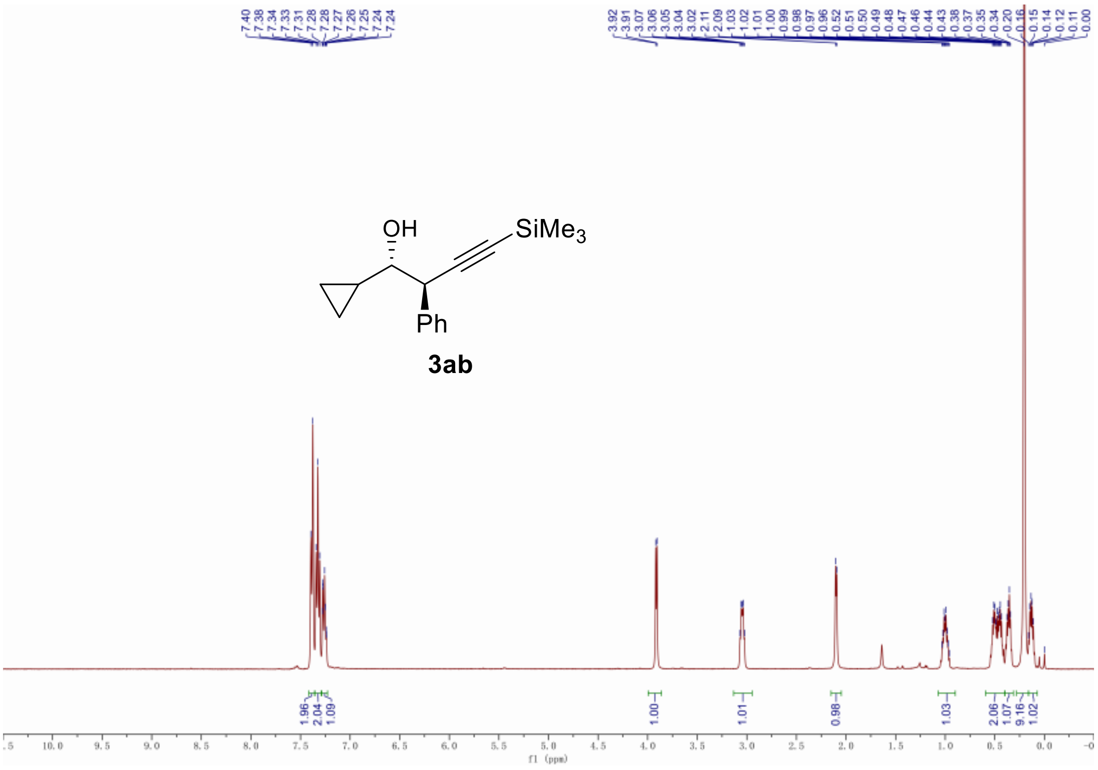

(^13^C NMR, 100 MHz, CDCl3)

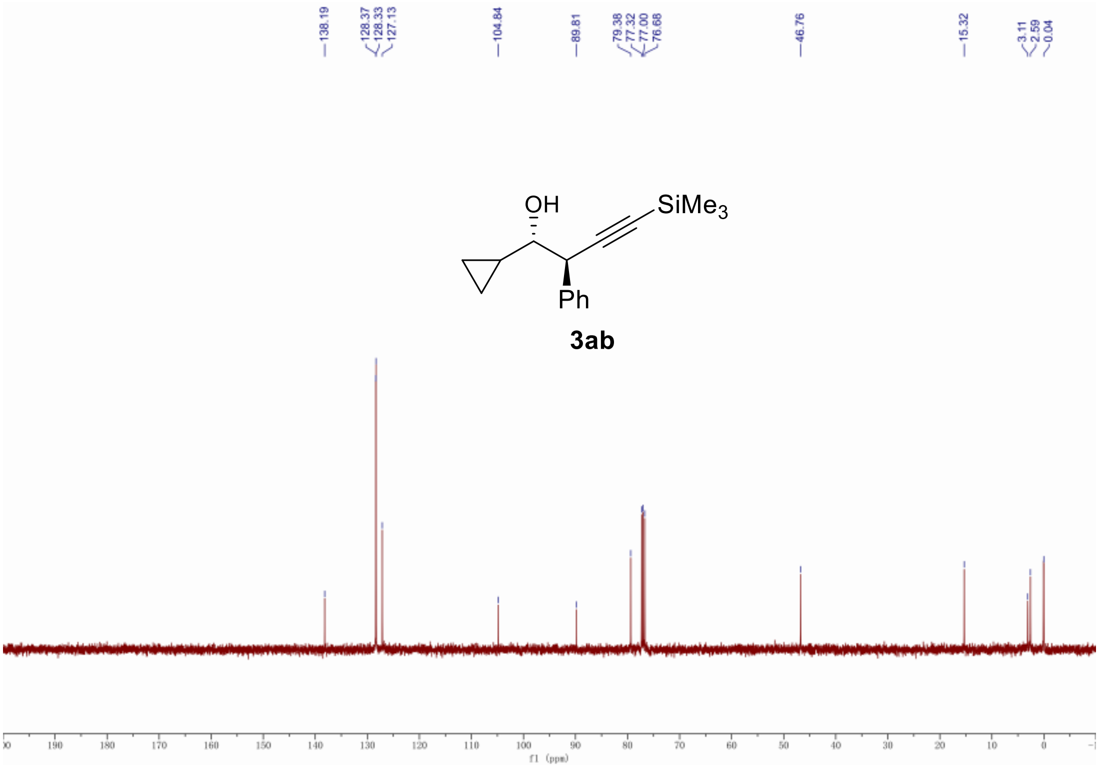

(^1^H NMR, 400 MHz, CDCl3)

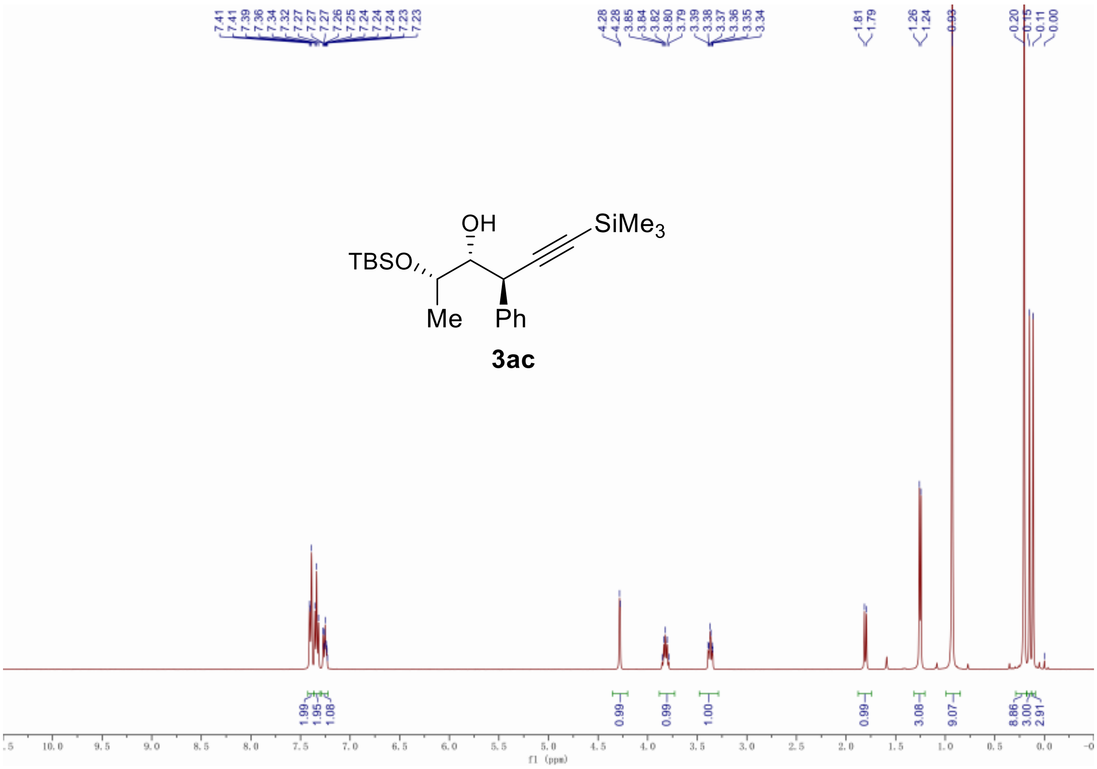

(^{13}C NMR, 100 MHz, $CDCl_3$)

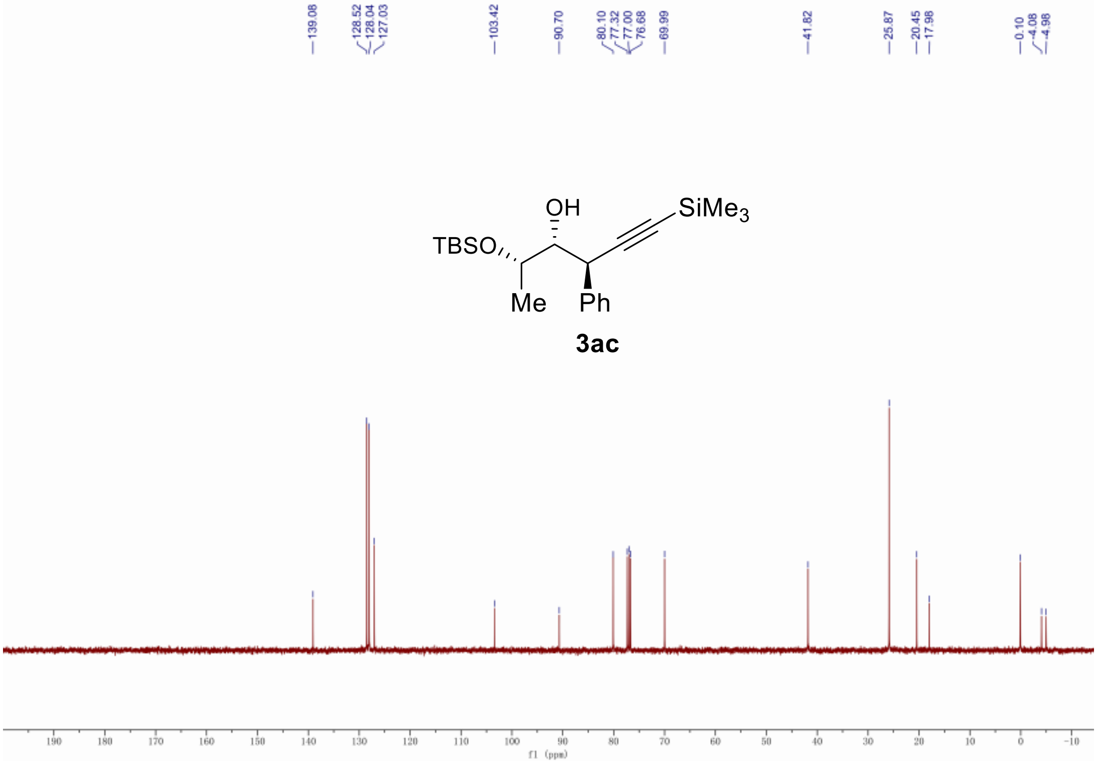

(^{1}H NMR, 400 MHz, $CDCl_3$)

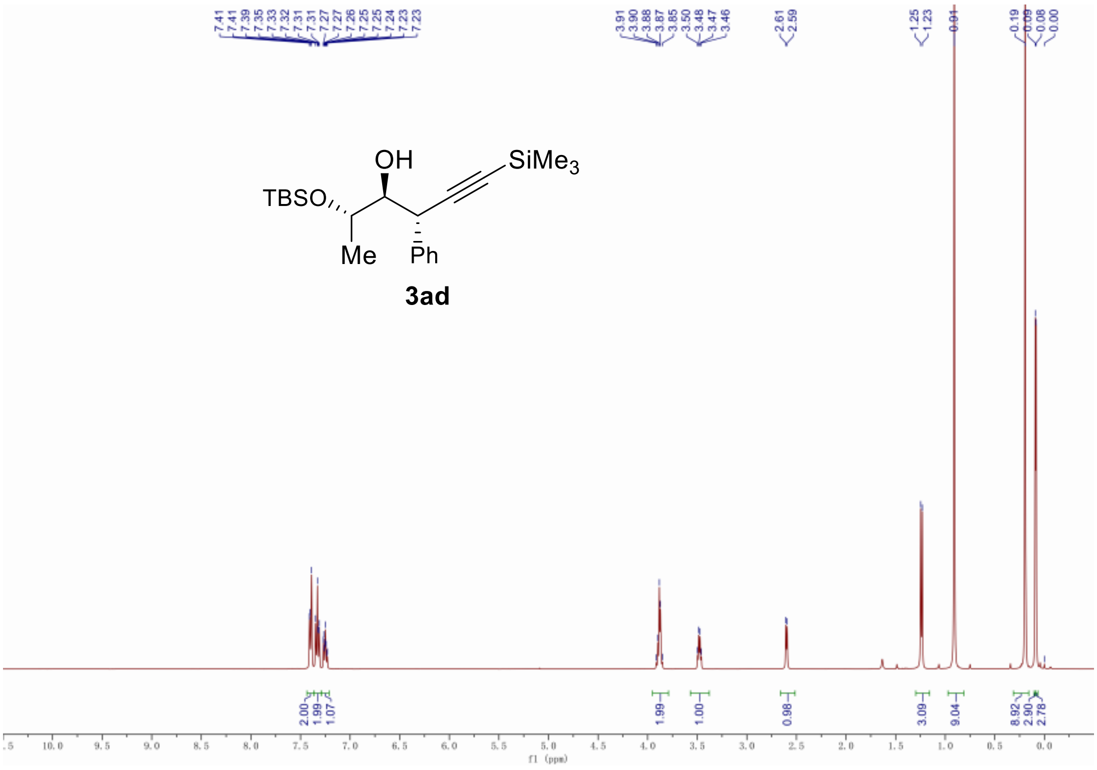

(^{13}C NMR, 100 MHz, $CDCl_3$)

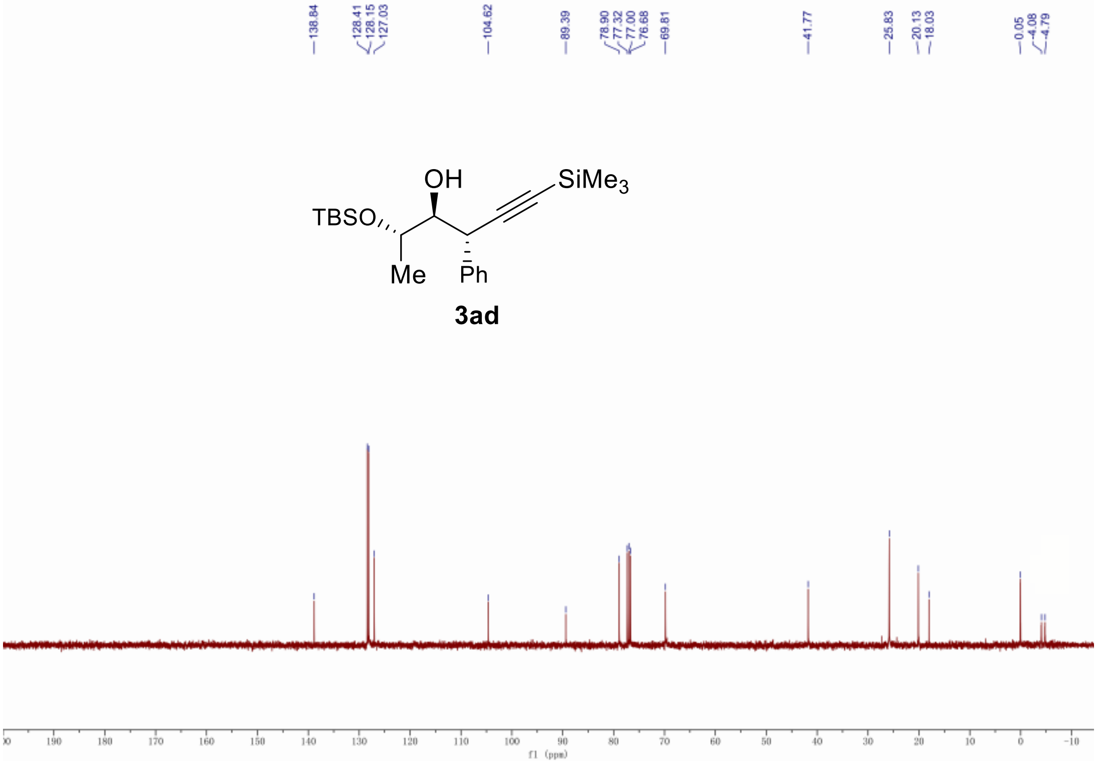

(^{1}H NMR, 400 MHz, $CDCl_3$)

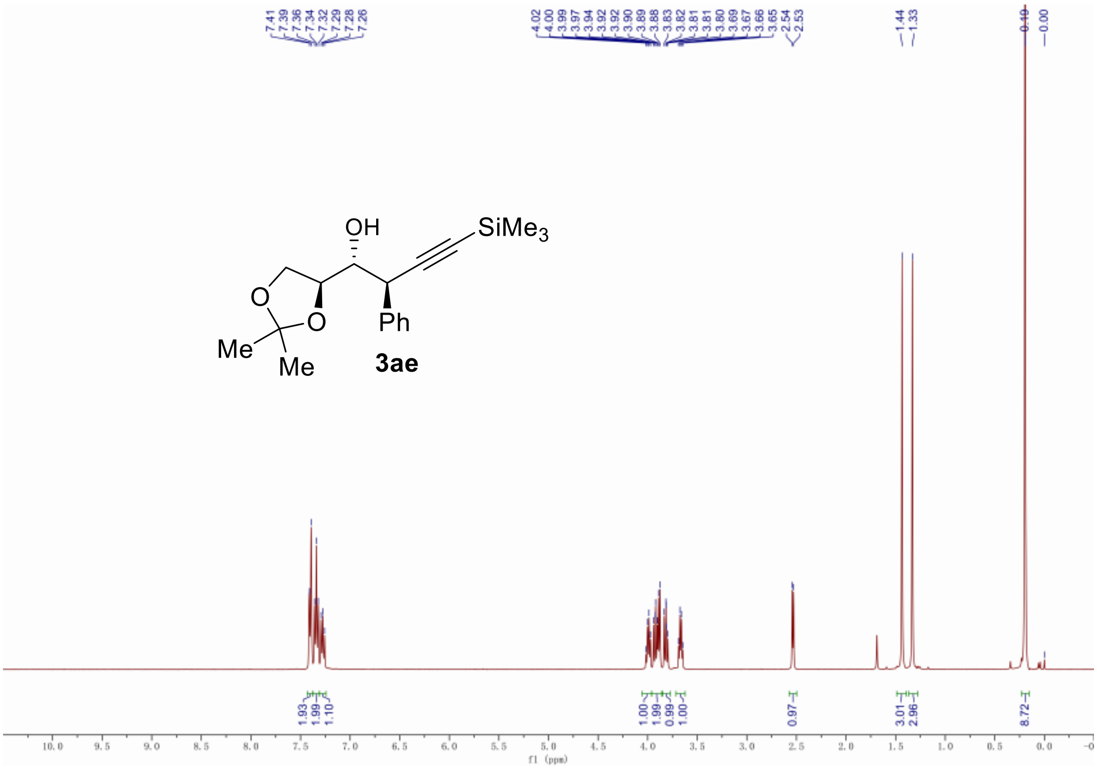

(^{13}C NMR, 100 MHz, $CDCl_3$)

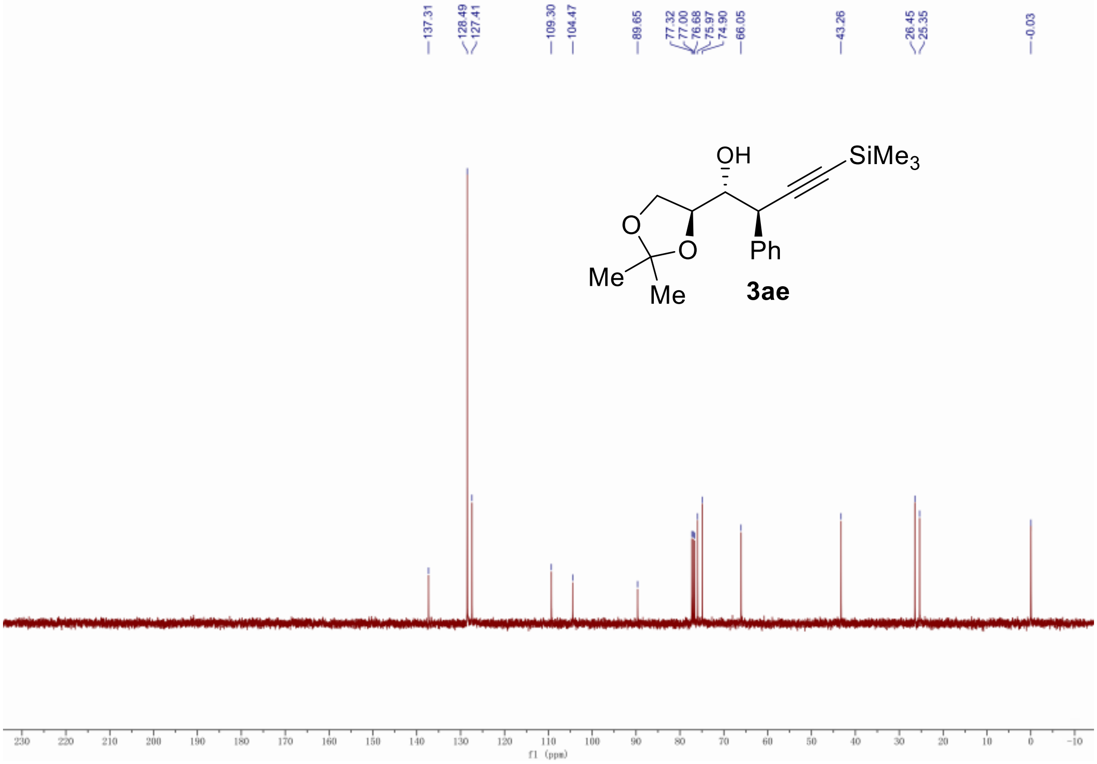

(^{1}H NMR, 400 MHz, $CDCl_3$)

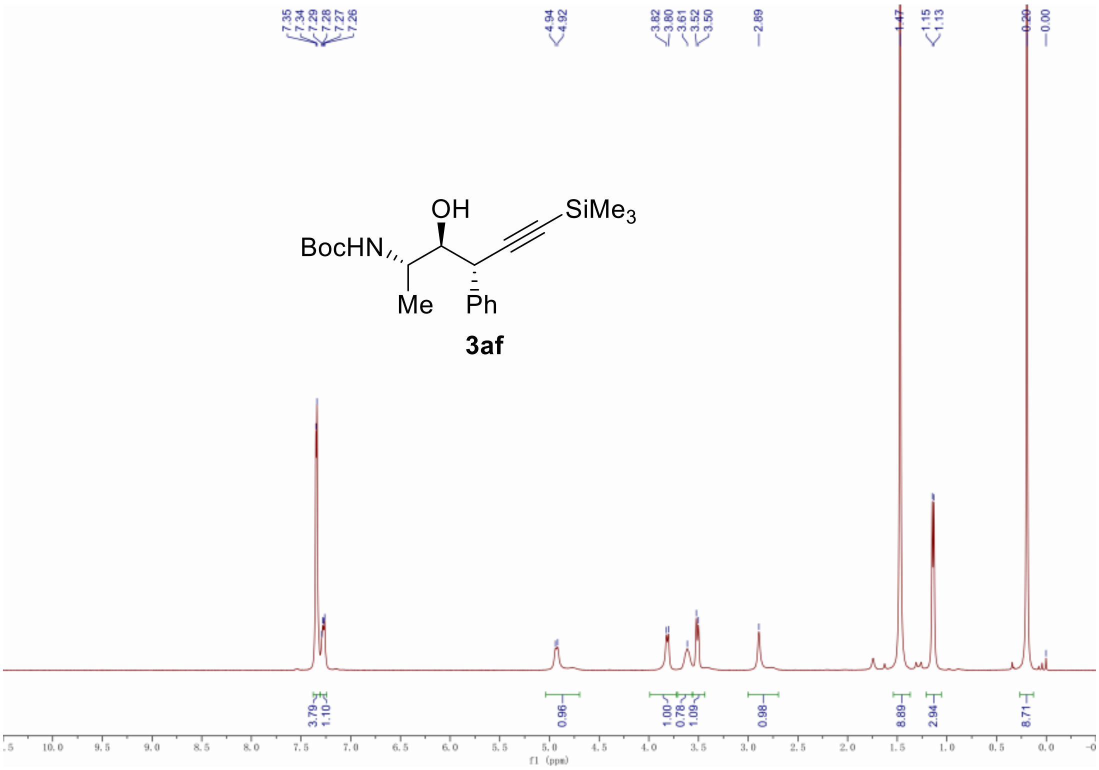

(^{13}C NMR, 100 MHz, $CDCl_3$)

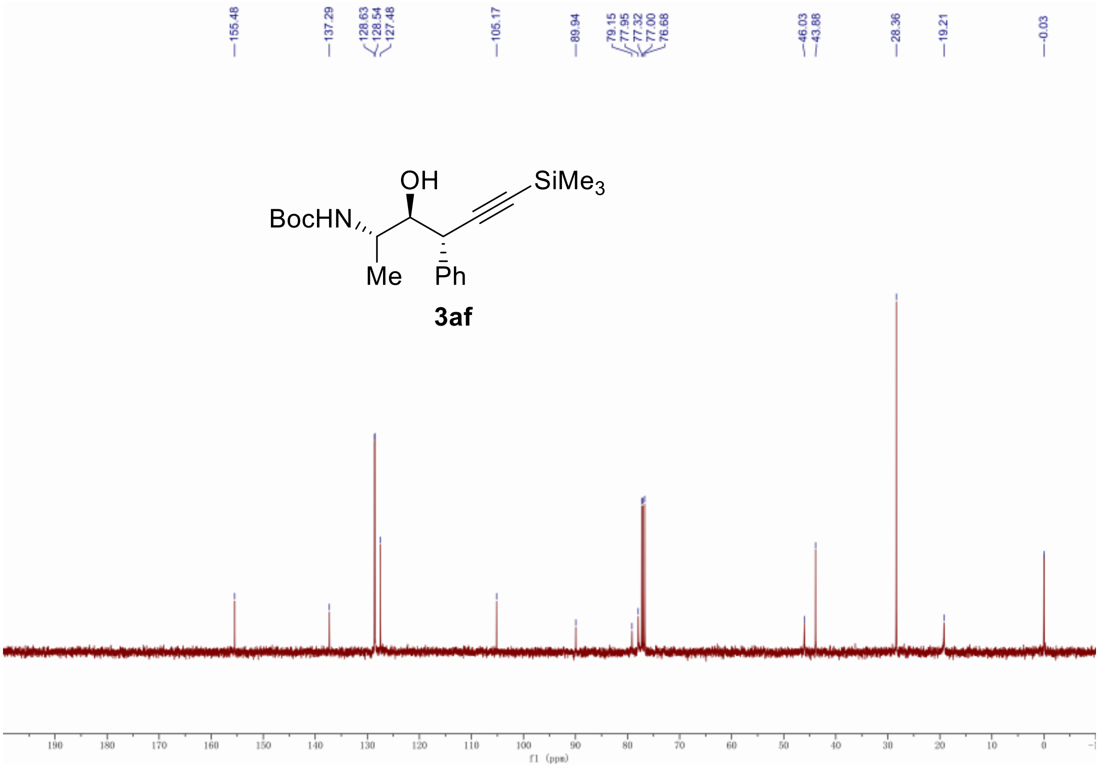

(^{1}H NMR, 400 MHz, $CDCl_3$)

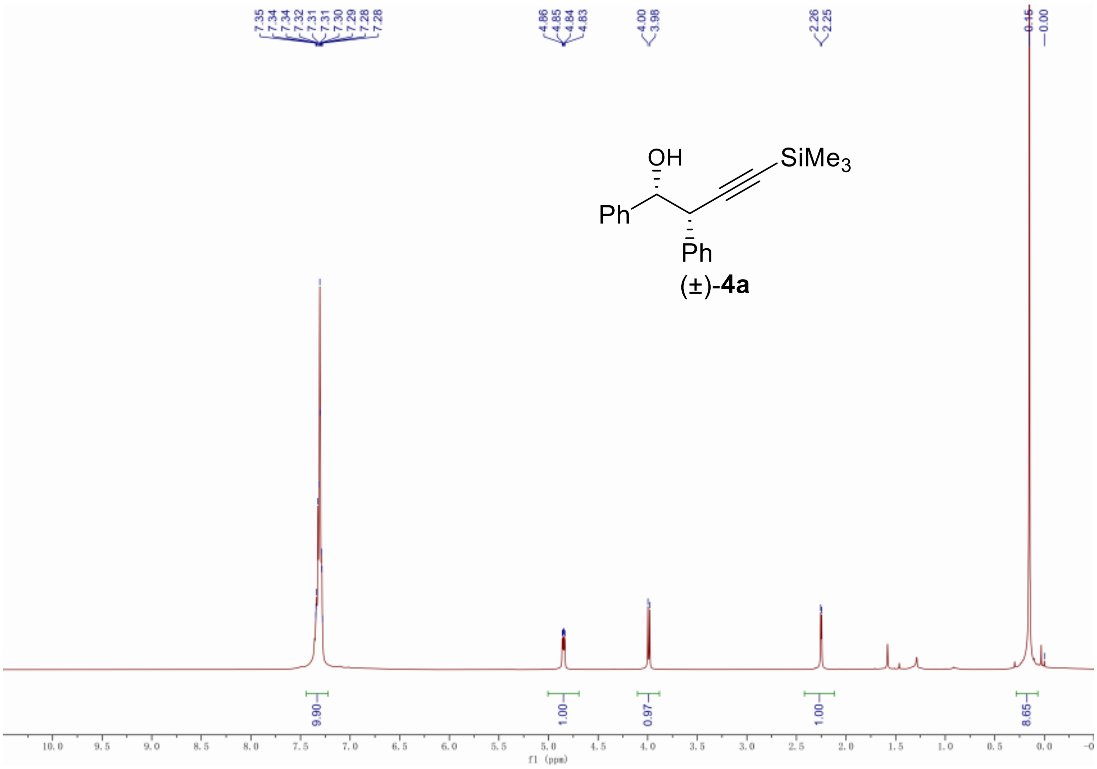

(^{13}C NMR, 100 MHz, $CDCl_3$)

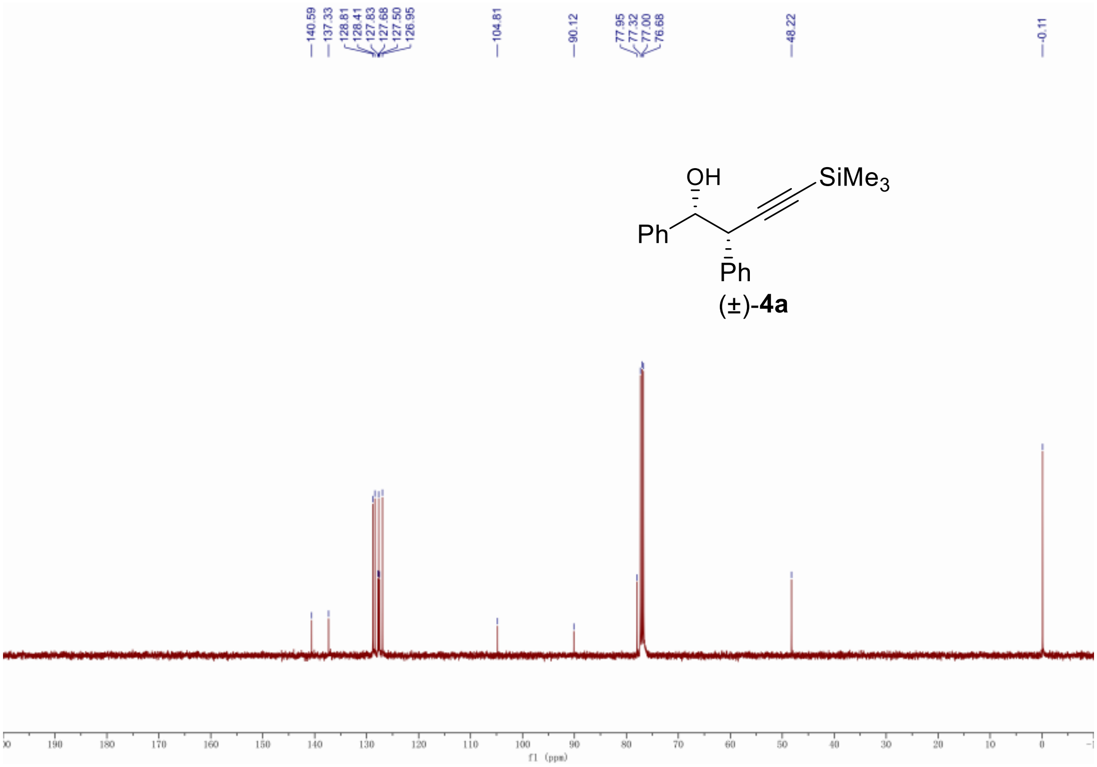

(^{1}H NMR, 400 MHz, $CDCl_3$)

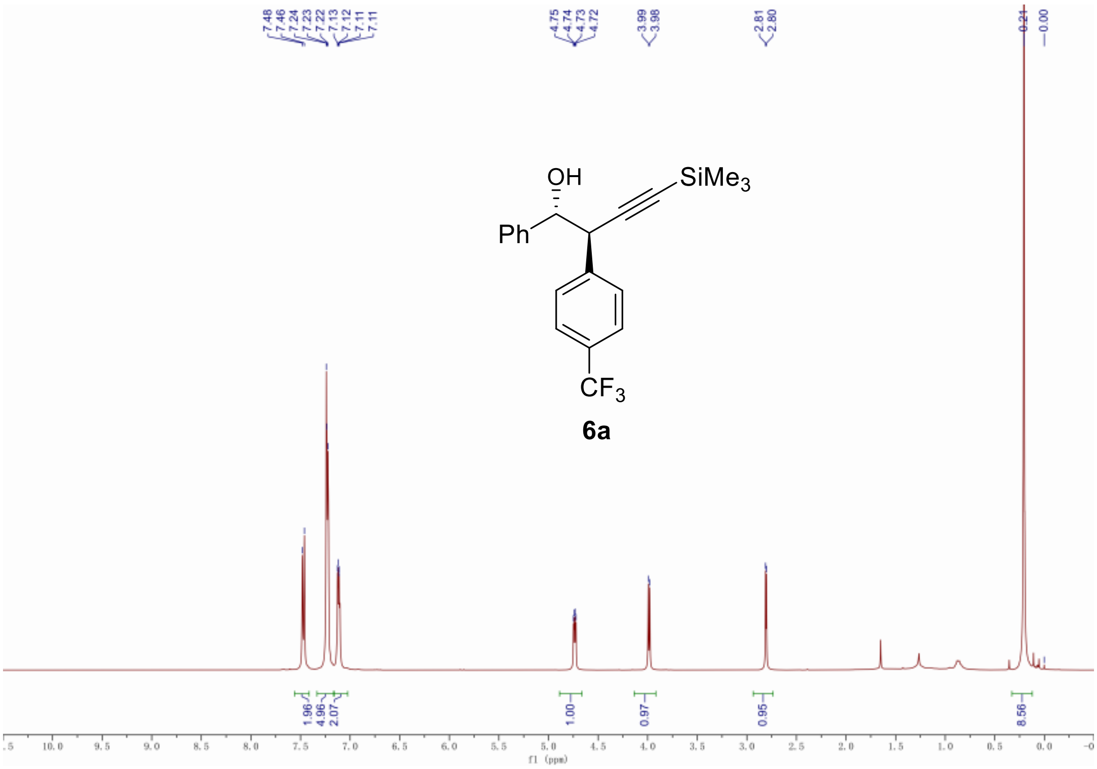

(^{13}C NMR, 100 MHz, CDCl$_3$)

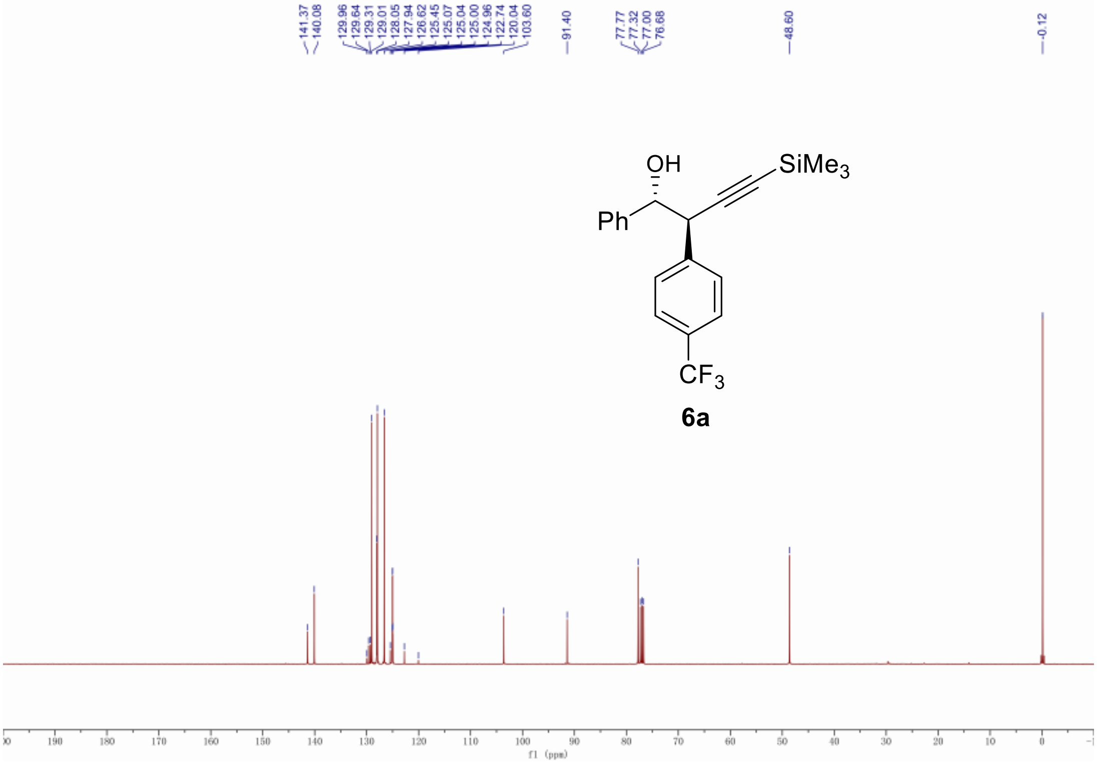

(^{19}F NMR, 376 MHz, CDCl$_3$)

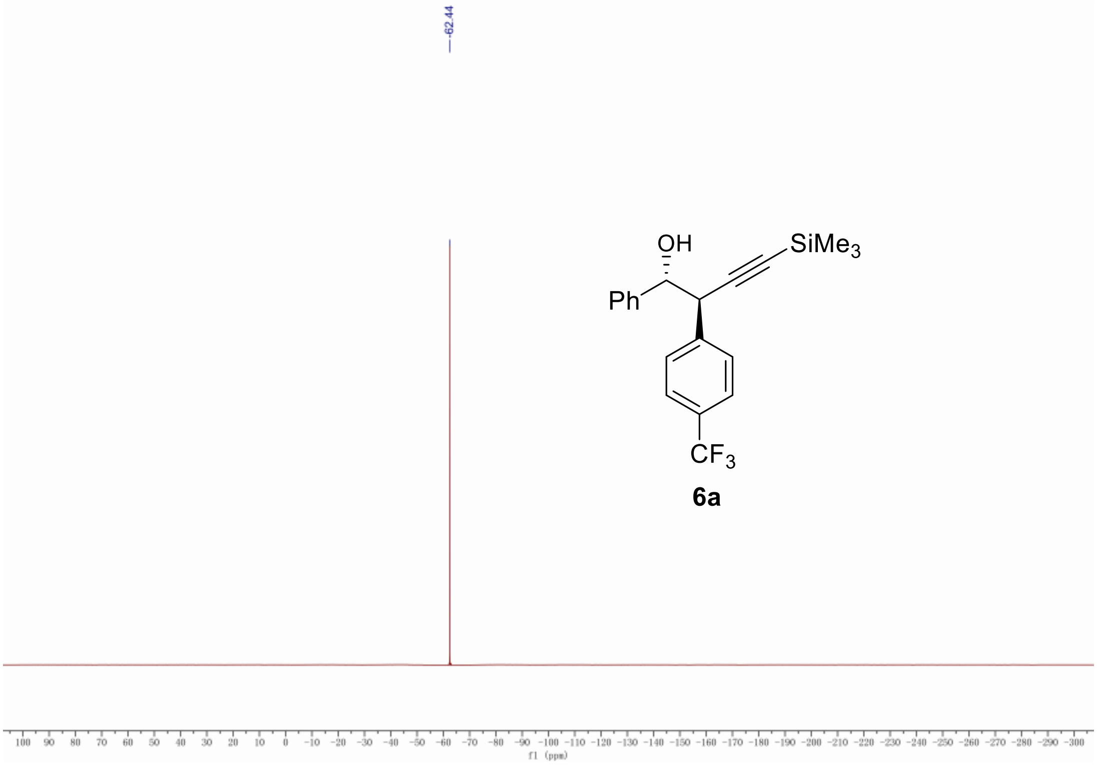

(¹H NMR, 400 MHz, $CDCl_3$)

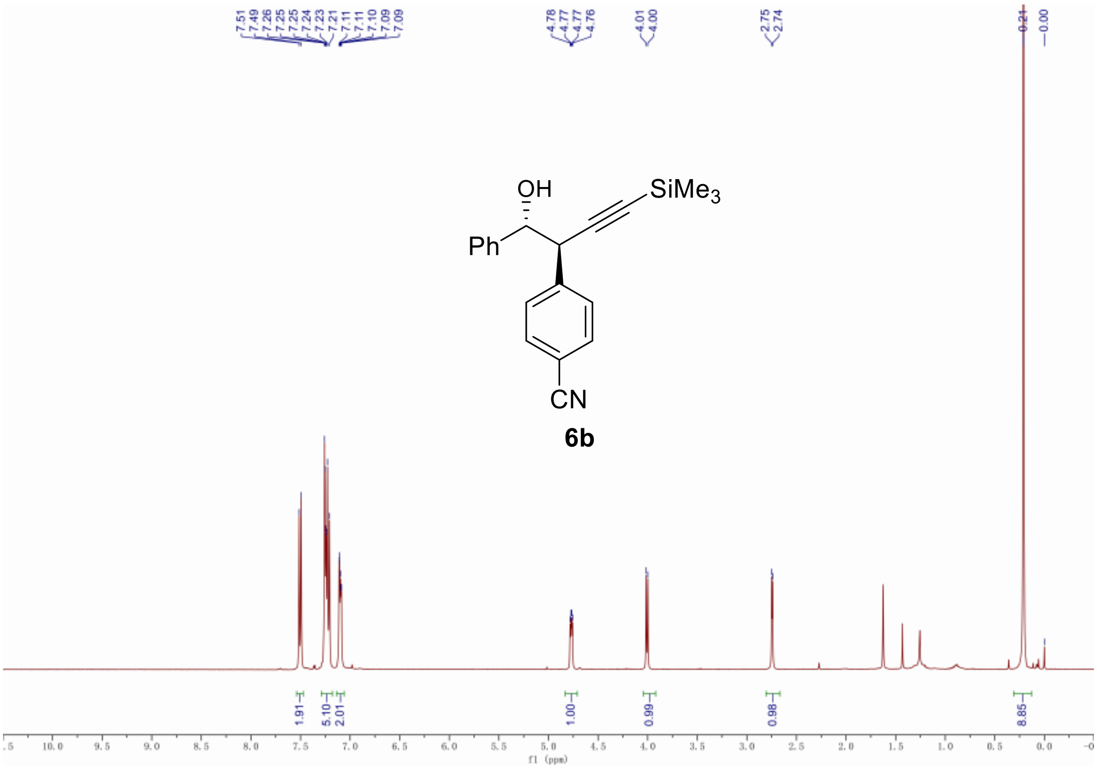

(¹³C NMR, 100 MHz, $CDCl_3$)

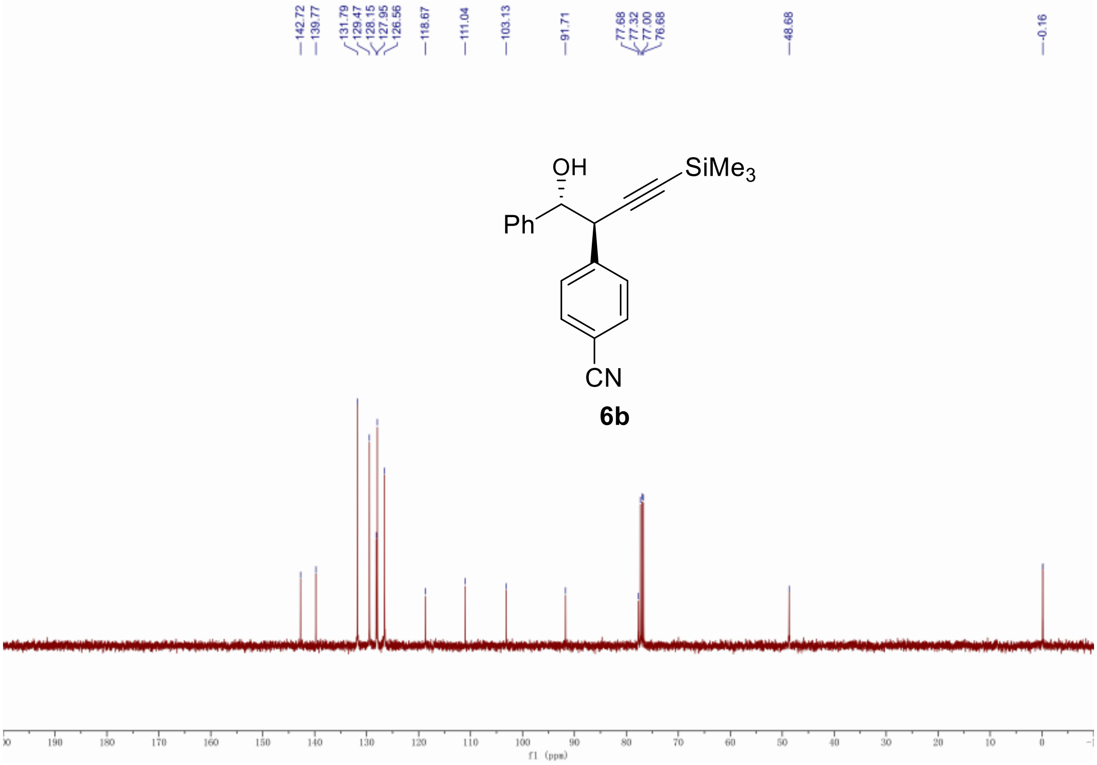

(1H NMR, 400 MHz, CDCl3)

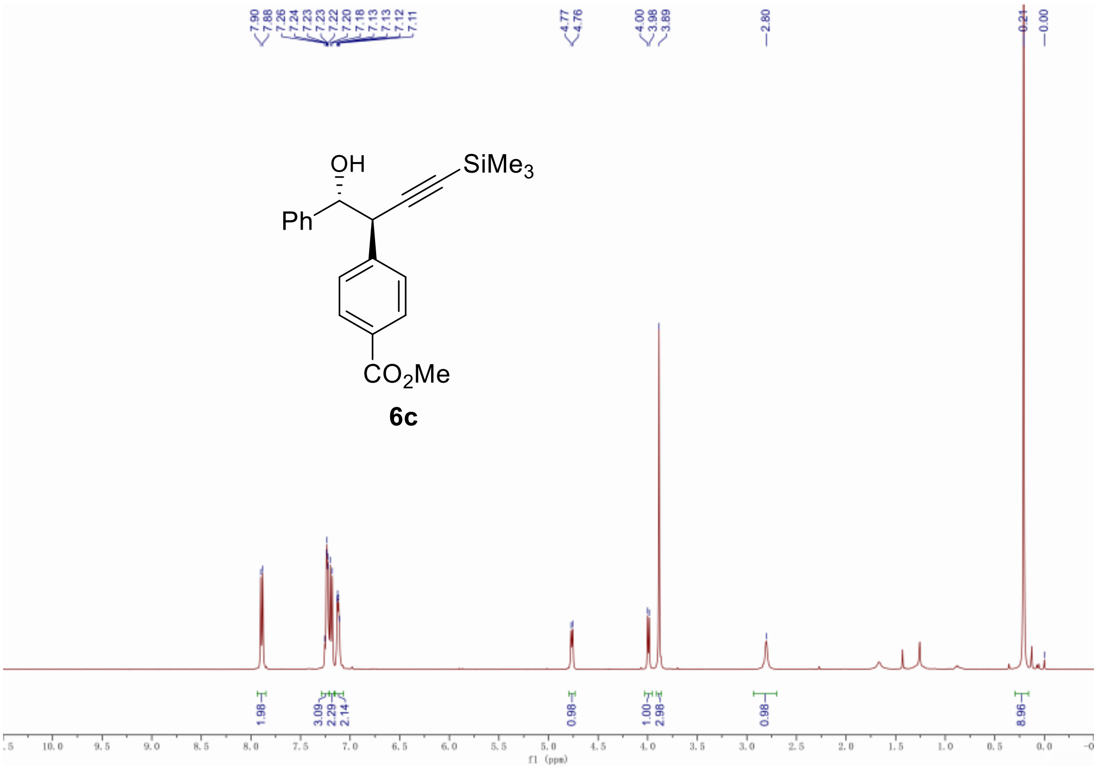

(13C NMR, 100 MHz, CDCl3)

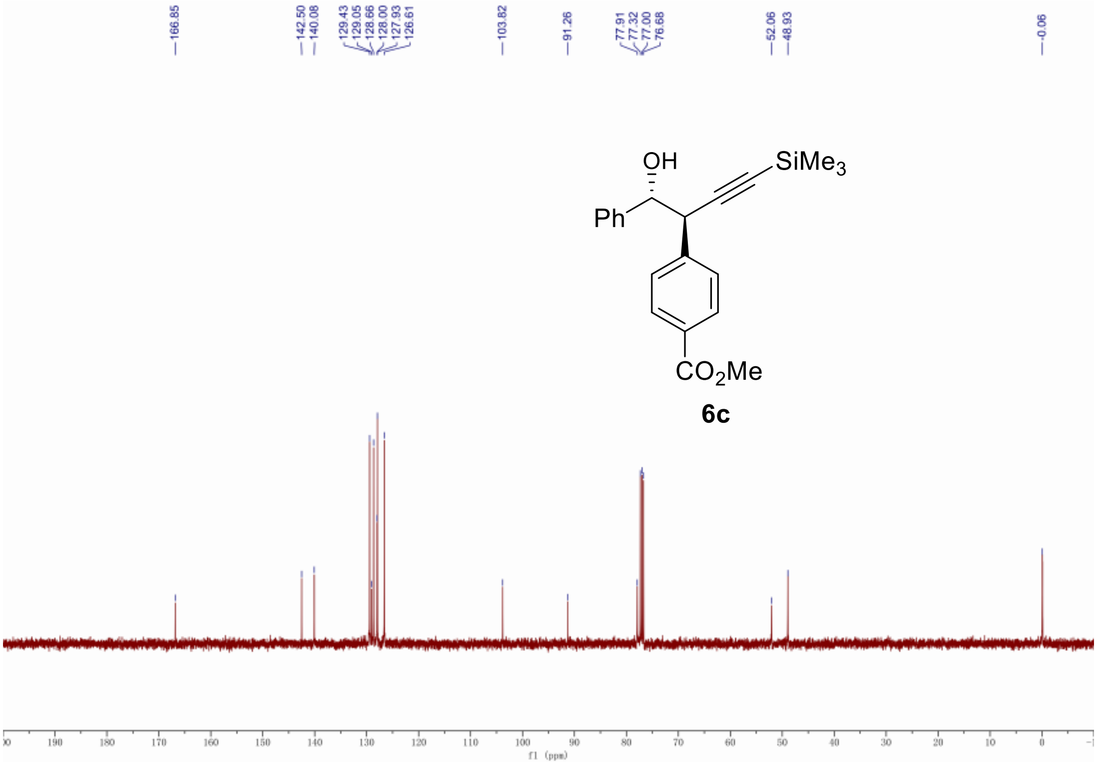

(¹H NMR, 400 MHz, $CDCl_3$)

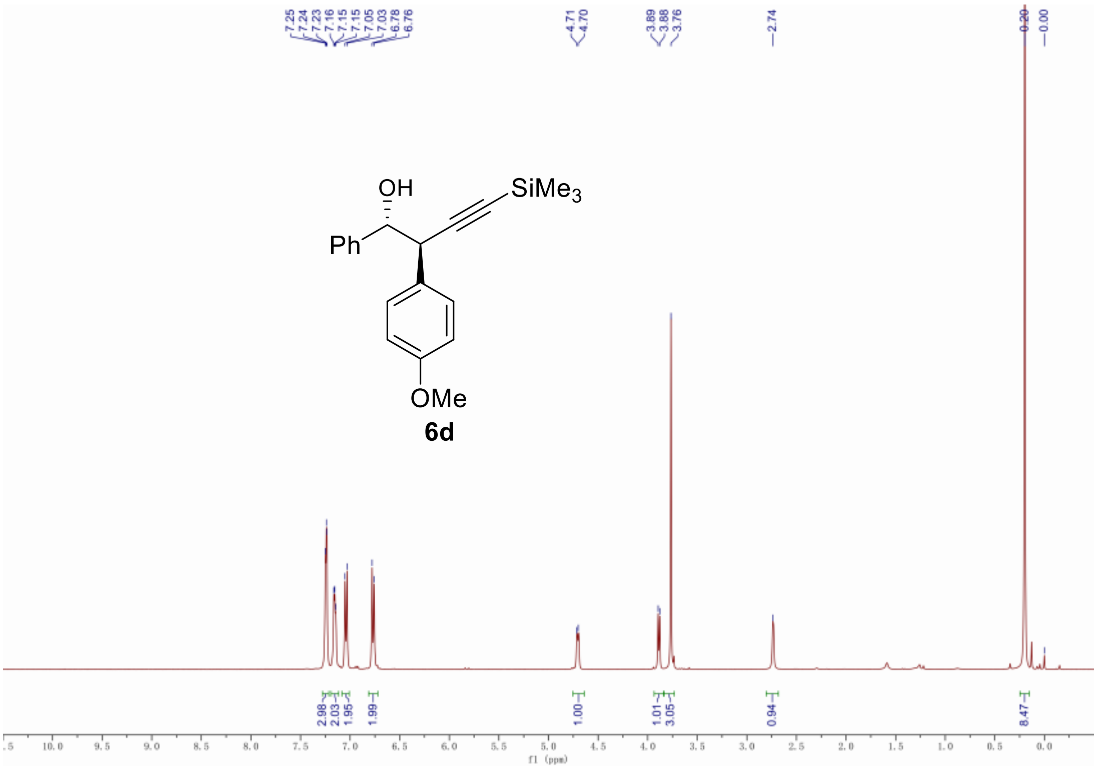

(^{13}C NMR, 100 MHz, $CDCl_3$)

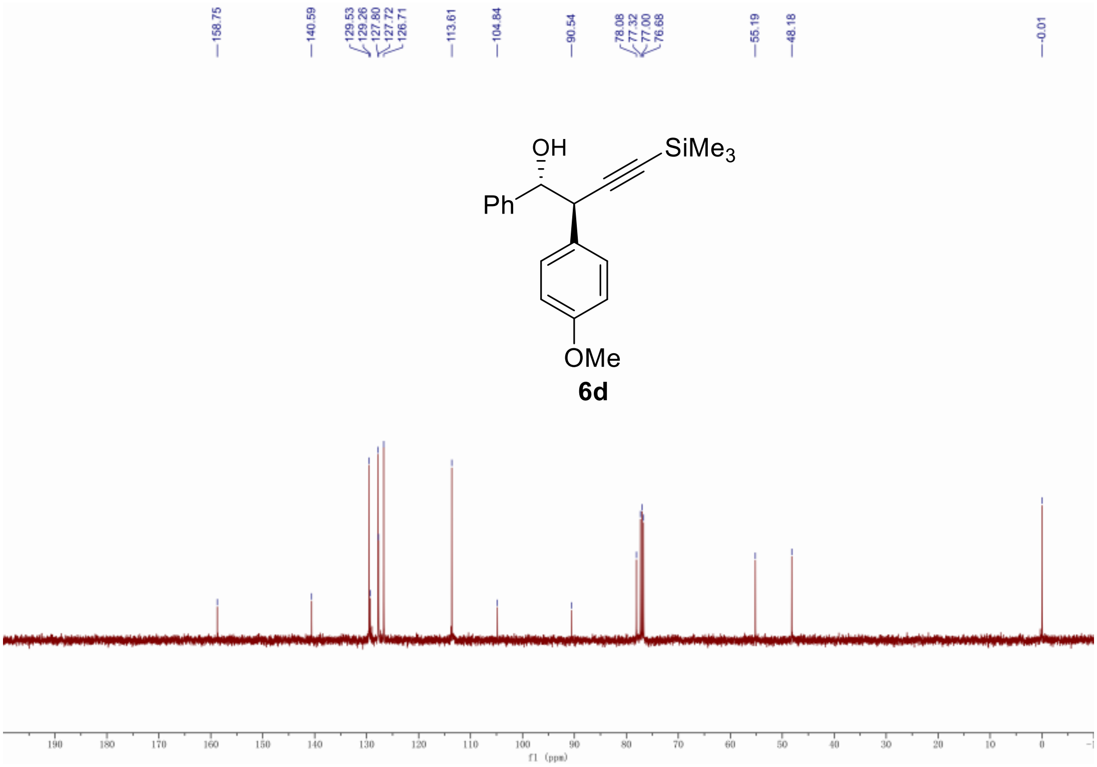

(1H NMR, 400 MHz, CDCl3)

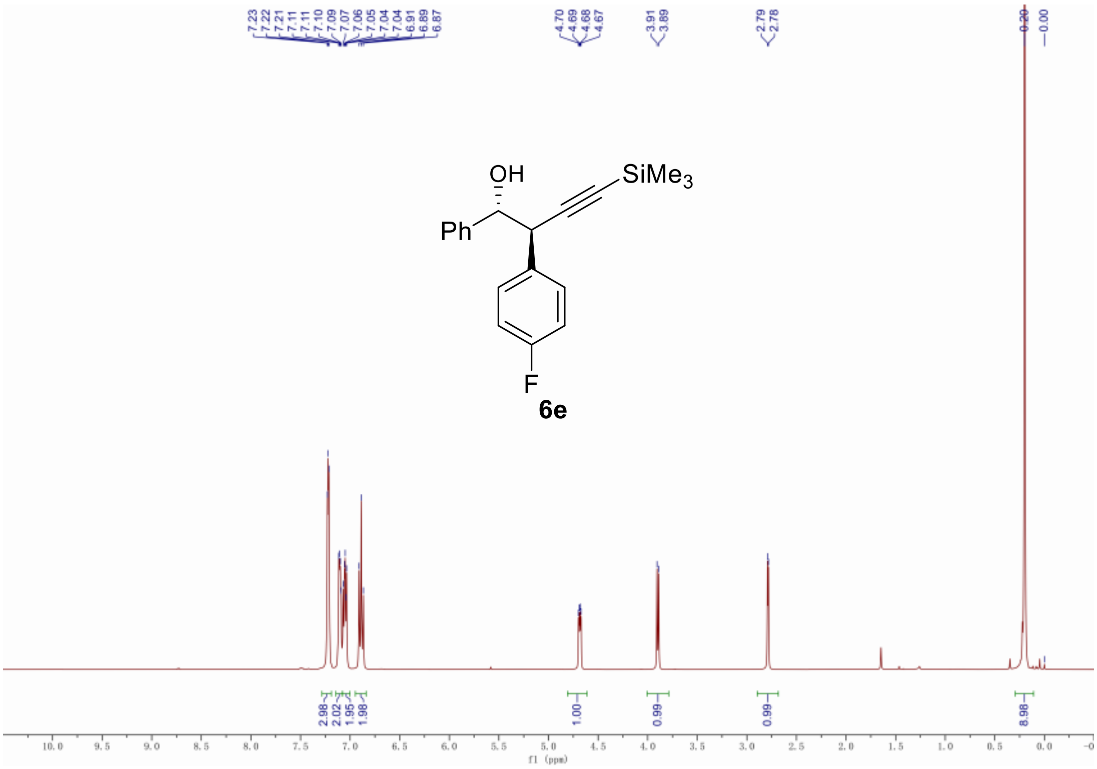

(13C NMR, 100 MHz, CDCl3)

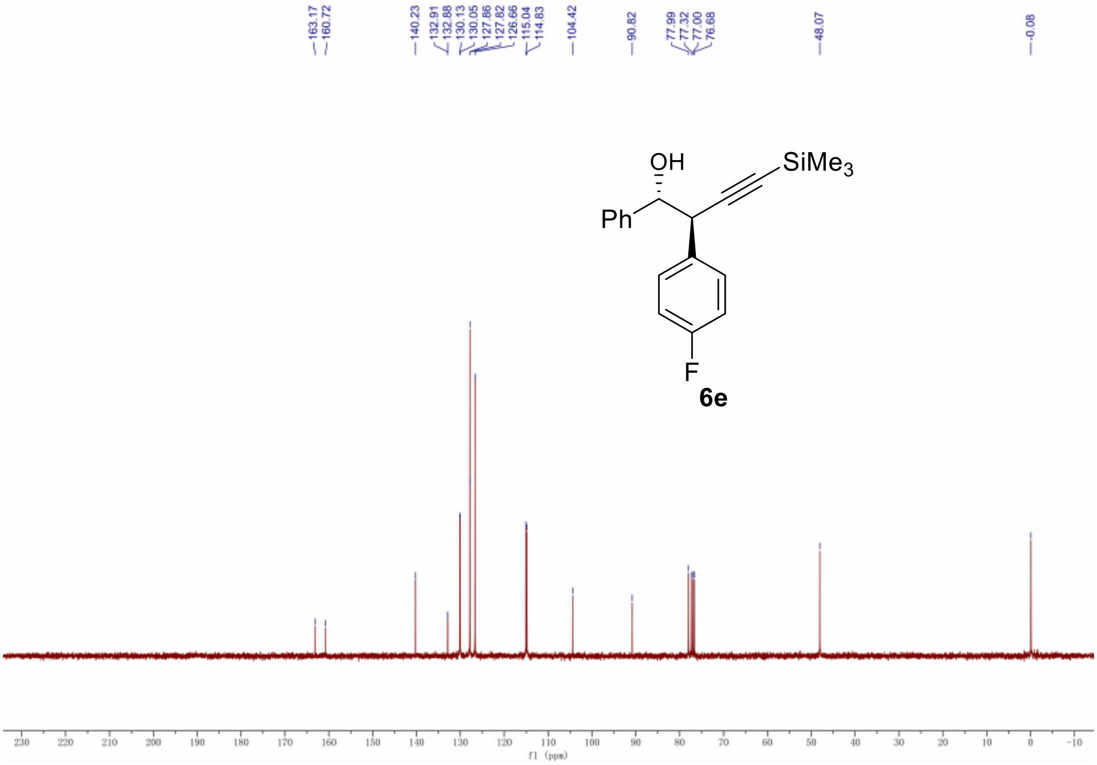

(^{19}F NMR, 376 MHz, $CDCl_3$)

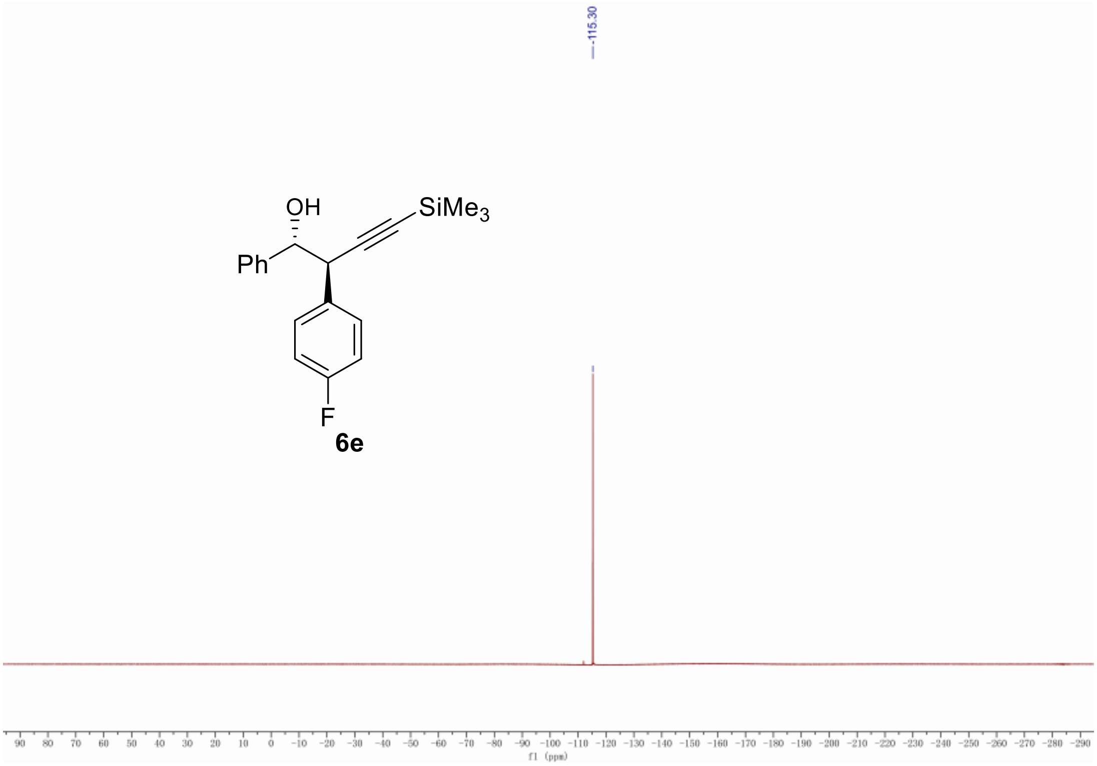

(^{1}H NMR, 400 MHz, $CDCl_3$)

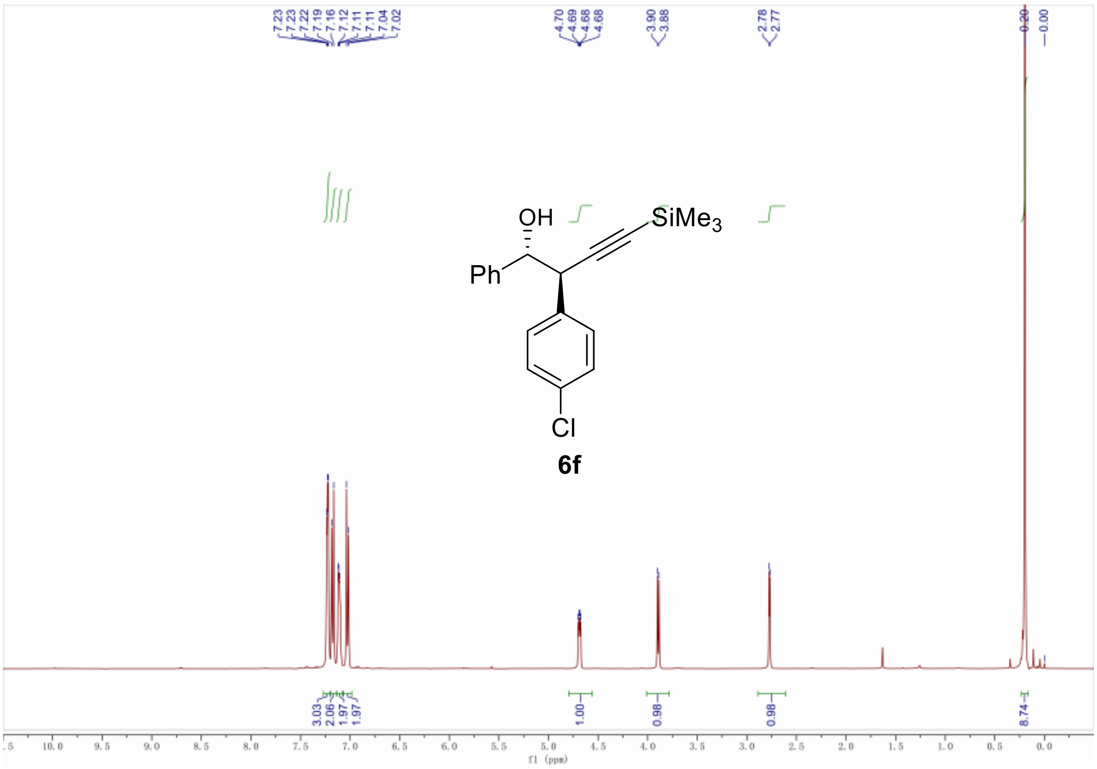

(13C NMR, 100 MHz, $CDCl_3$)

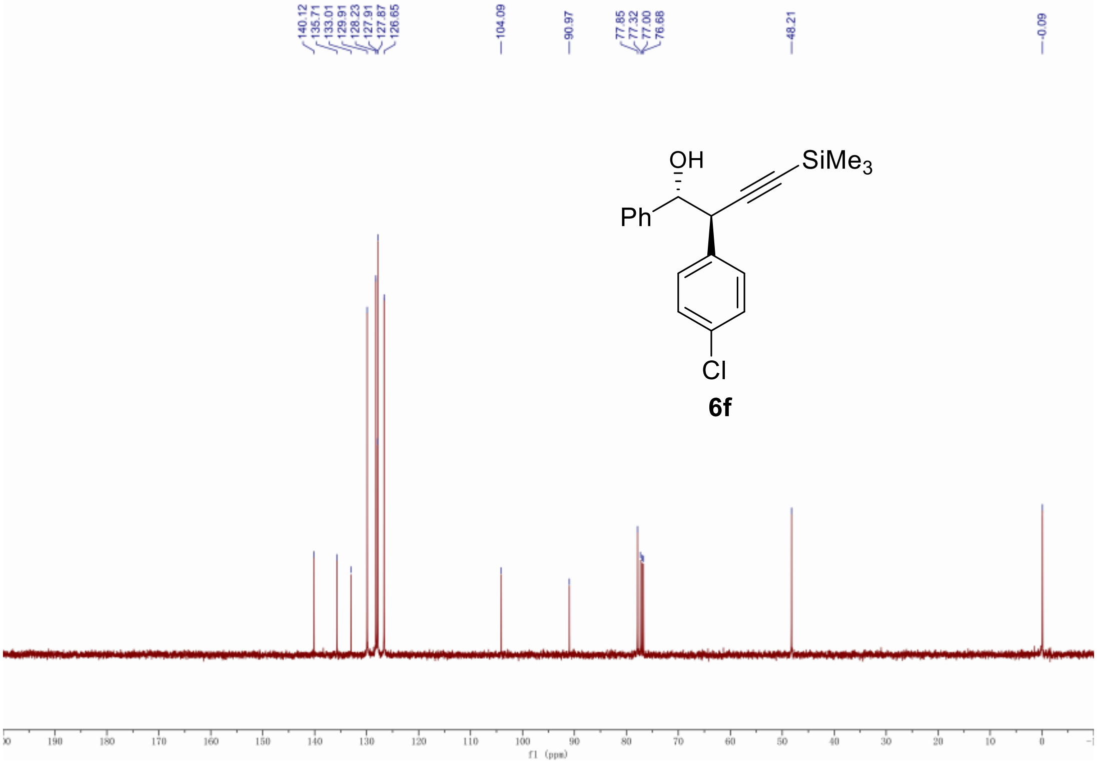

(1H NMR, 400 MHz, $CDCl_3$)

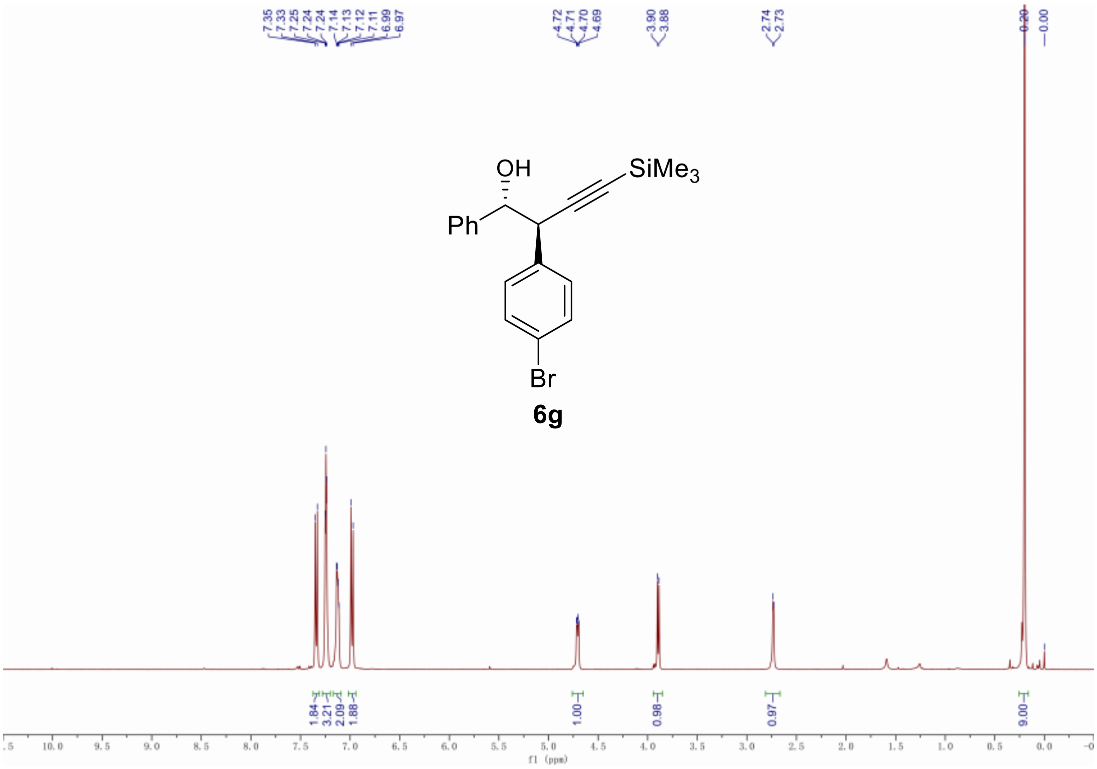

(^{13}C NMR, 100 MHz, CDCl$_3$)

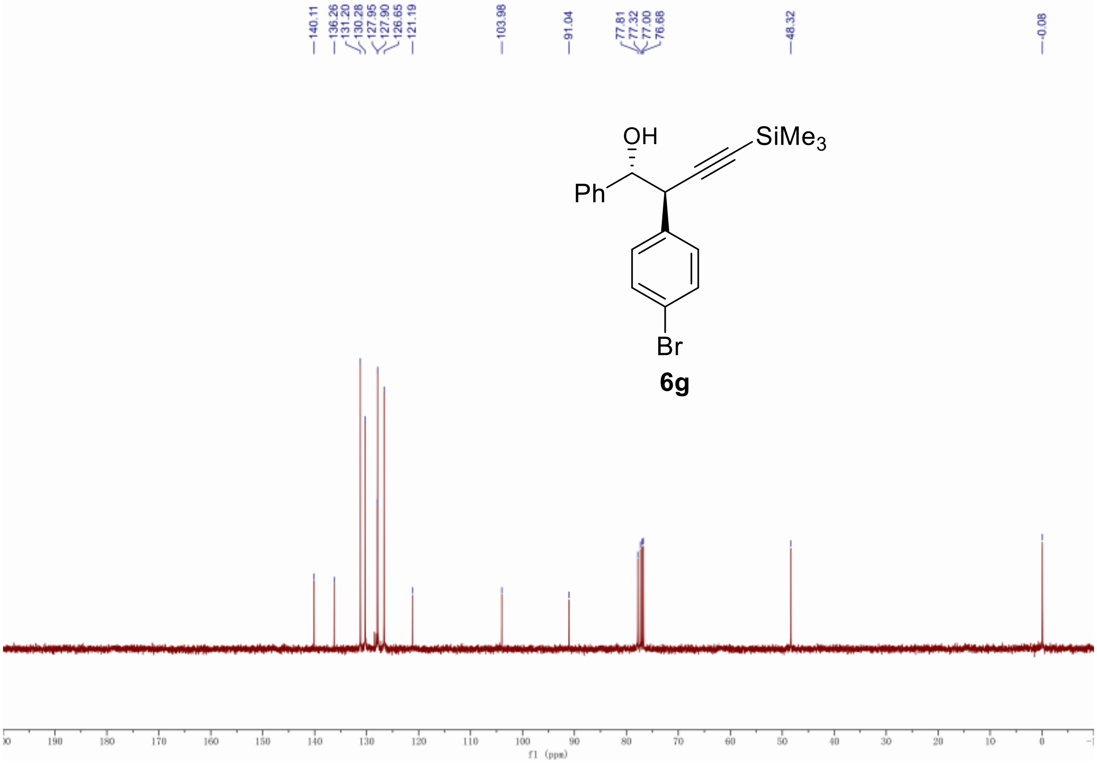

(^{1}H NMR, 400 MHz, CDCl$_3$)

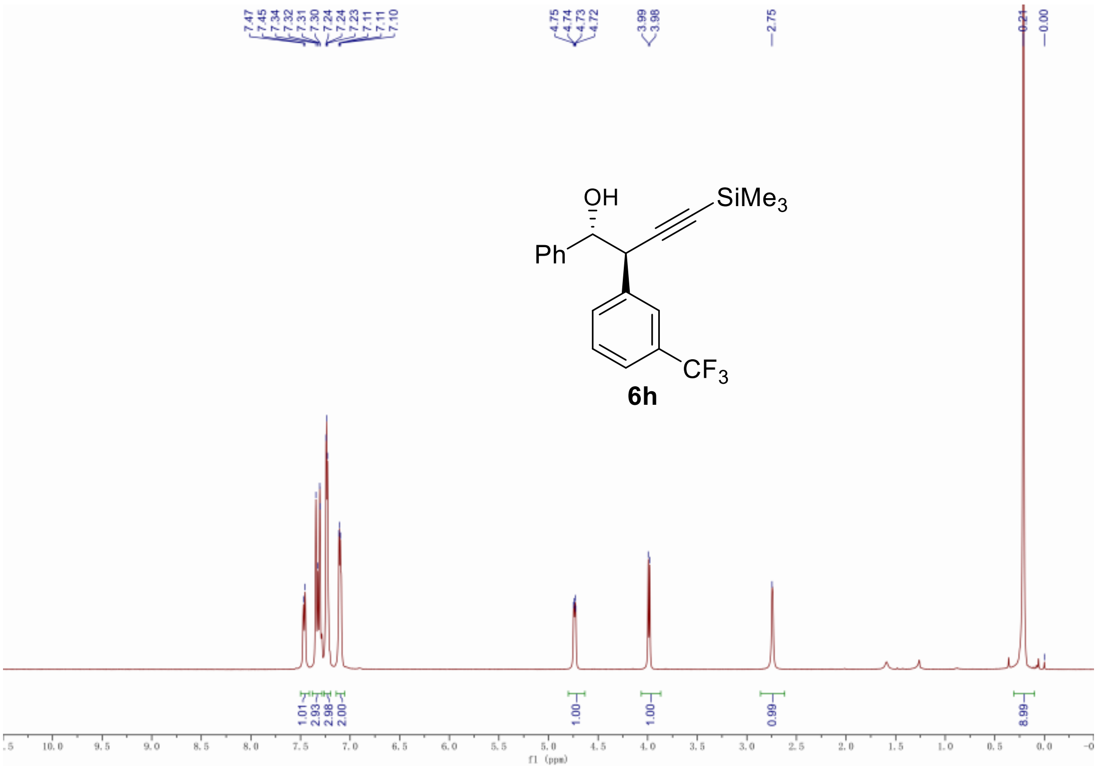

(^{13}C NMR, 100 MHz, CDCl$_3$)

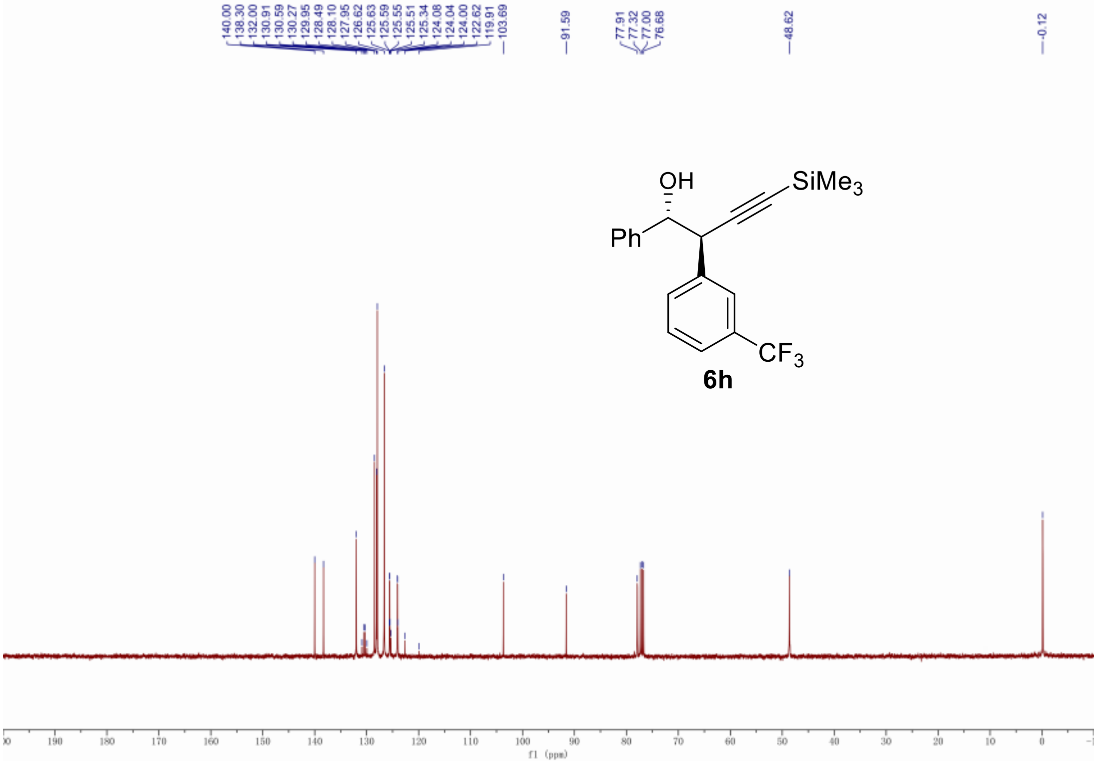

(^{19}F NMR, 376 MHz, CDCl$_3$)

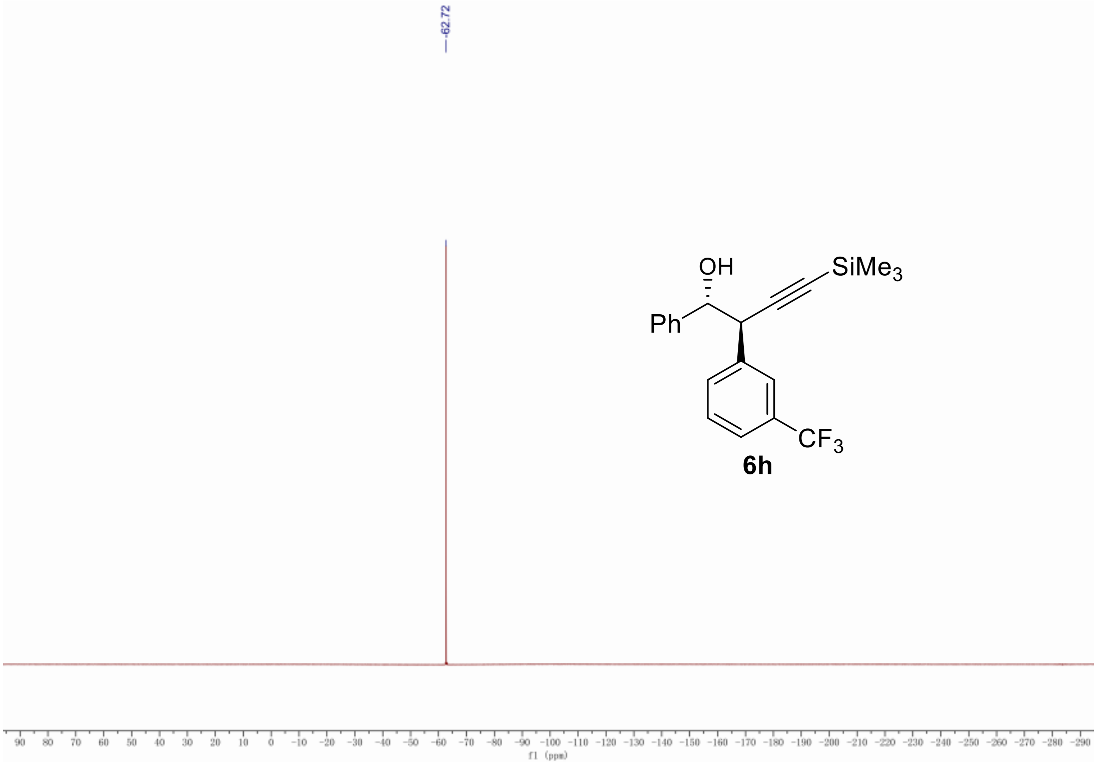

(^{1}H NMR, 400 MHz, $CDCl_3$)

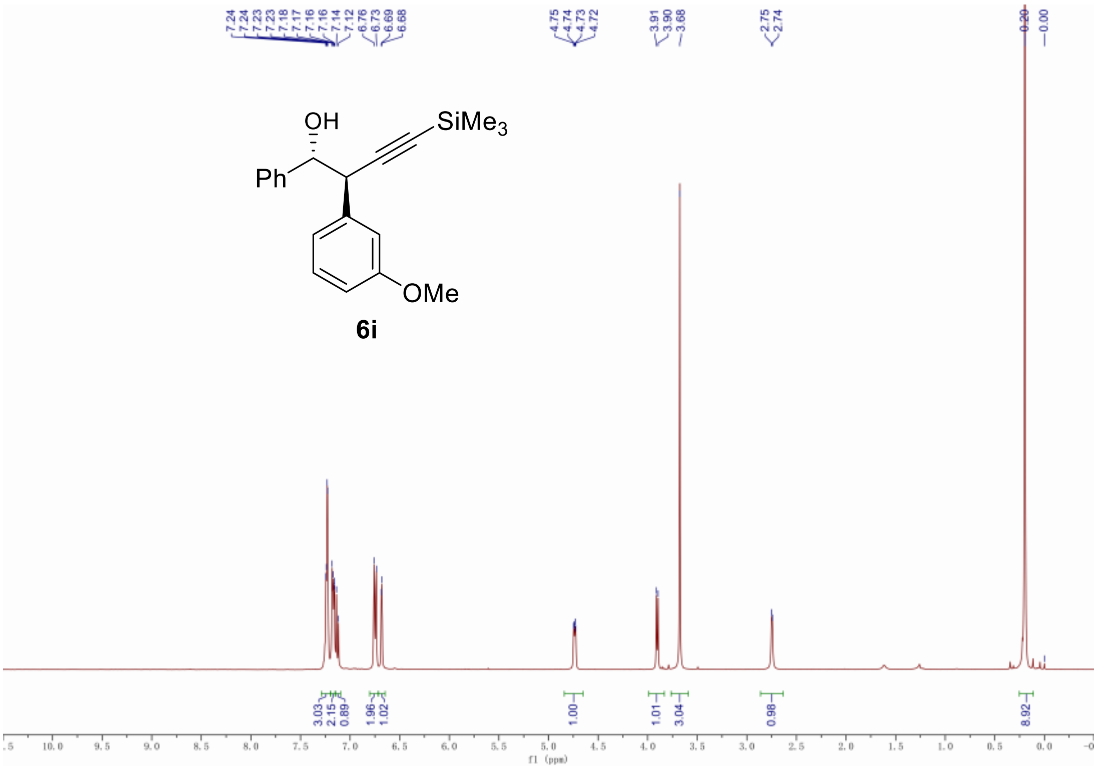

(^{13}C NMR, 100 MHz, $CDCl_3$)

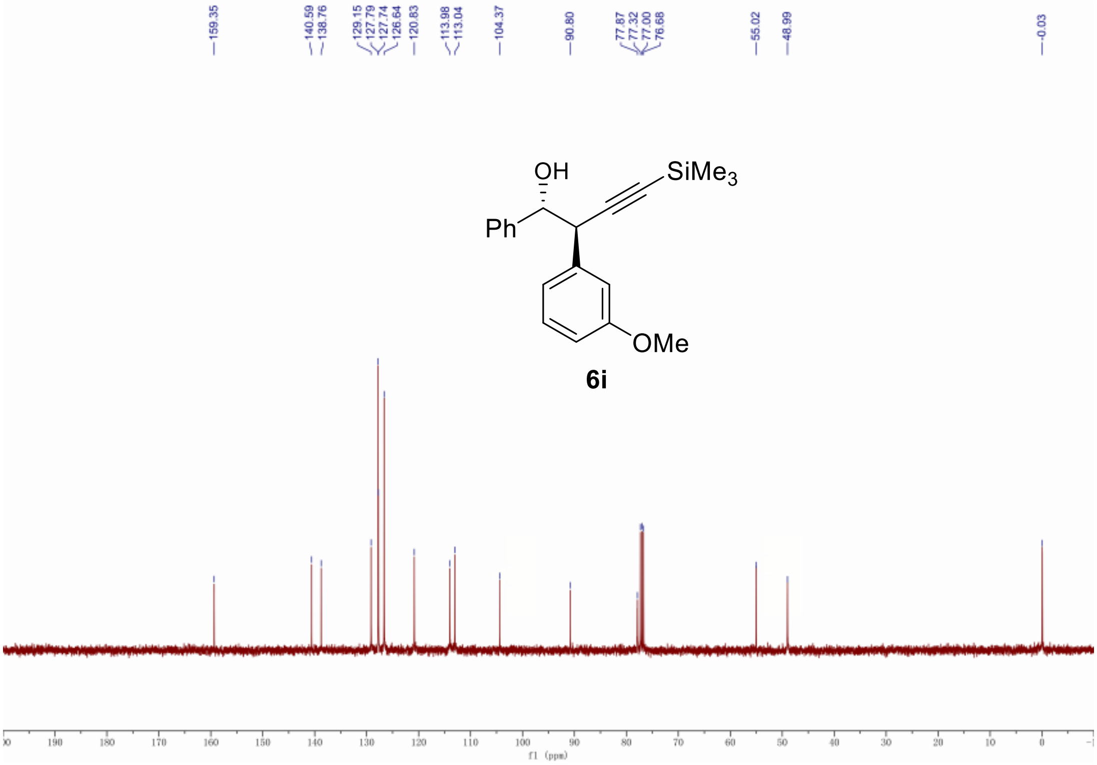

(¹H NMR, 400 MHz, $CDCl_3$)

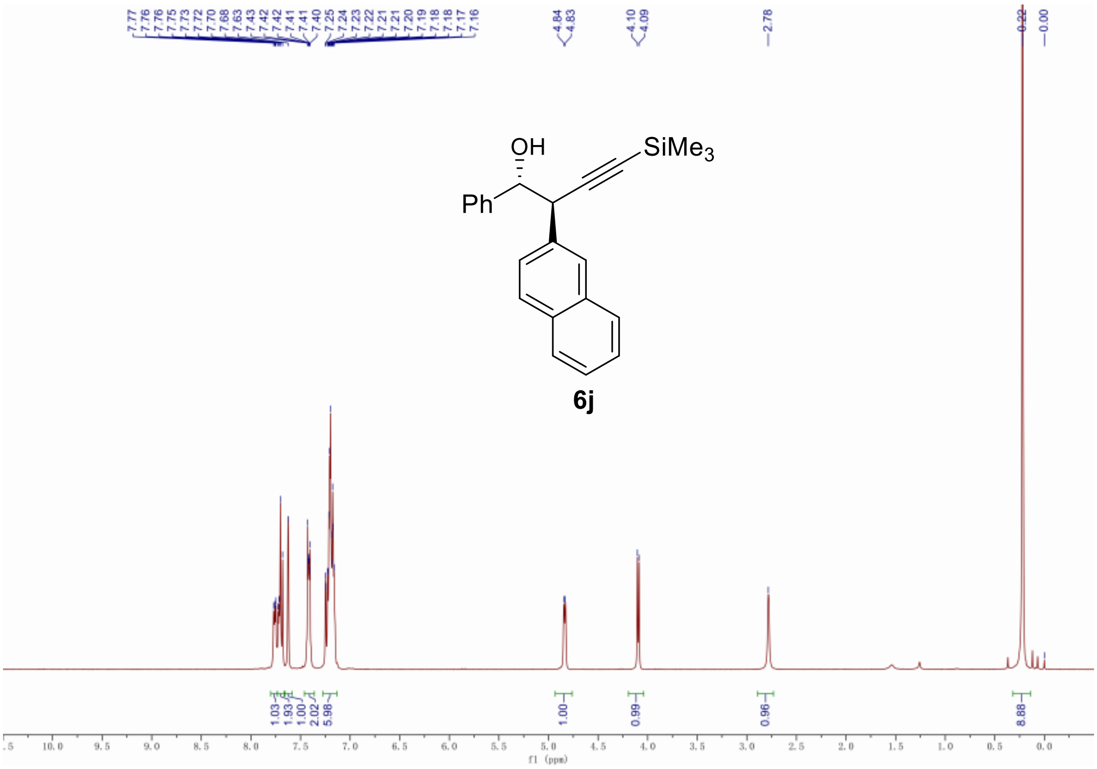

(¹³C NMR, 100 MHz, $CDCl_3$)

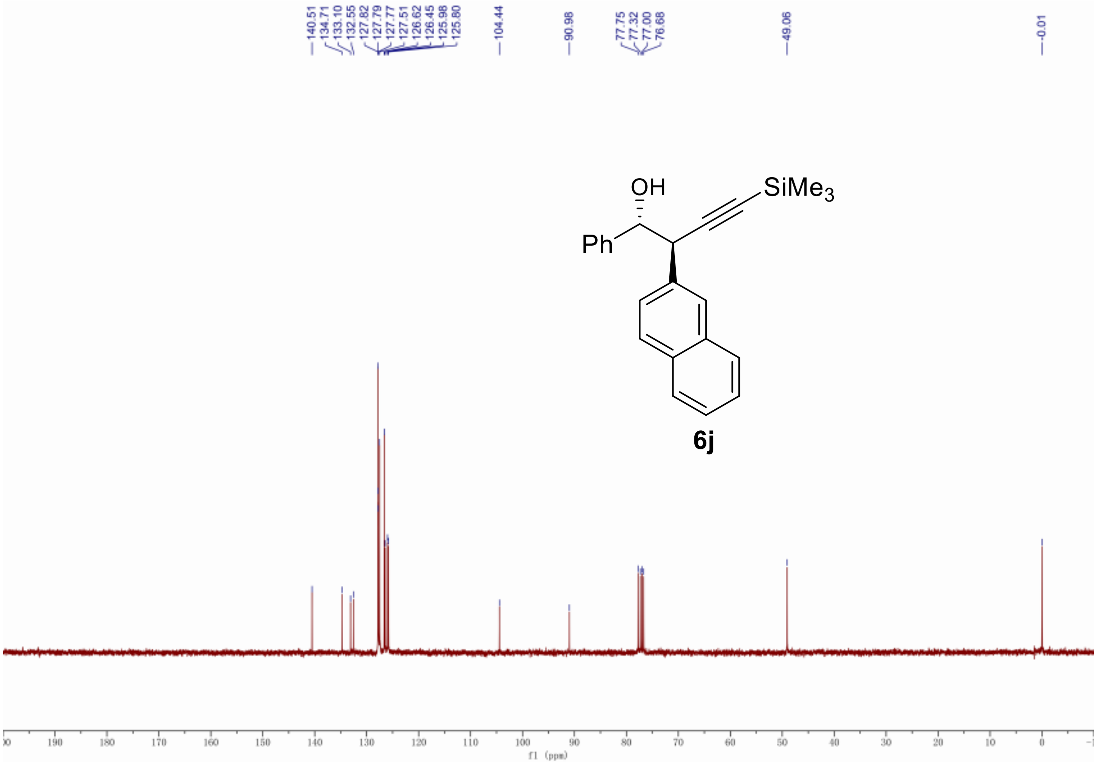

（^{1}H NMR, 400 MHz, $CDCl_3$）

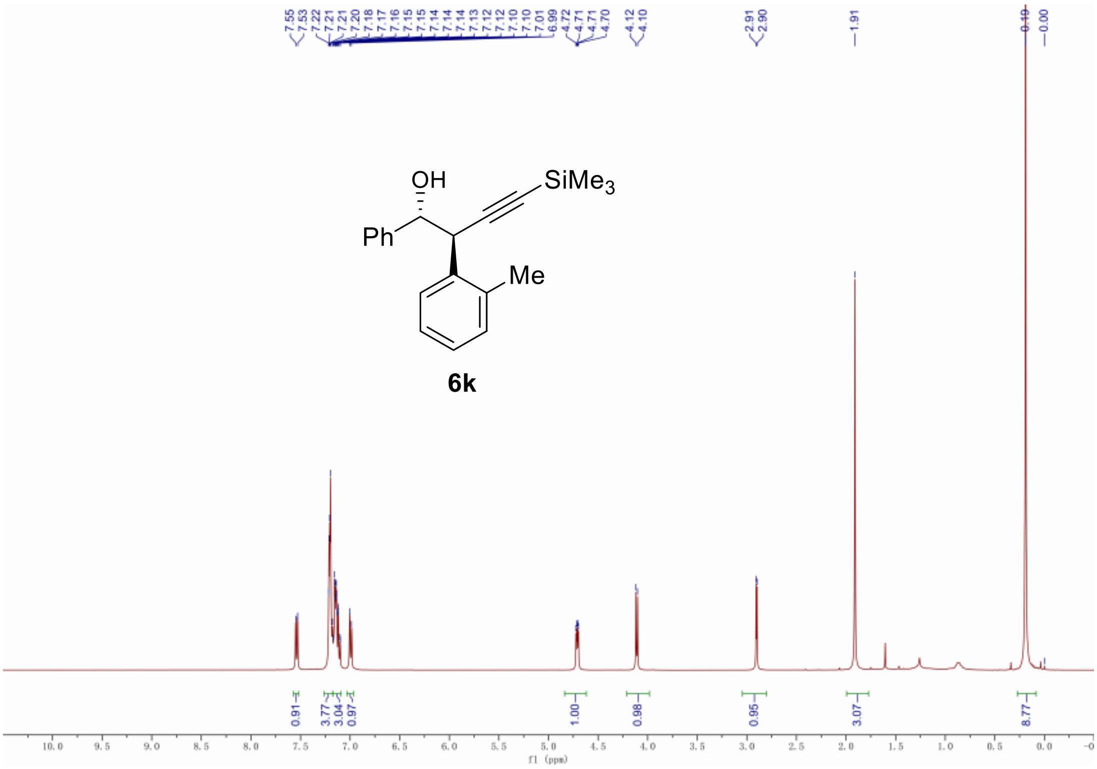

(^{13}C NMR, 100 MHz, $CDCl_3$)

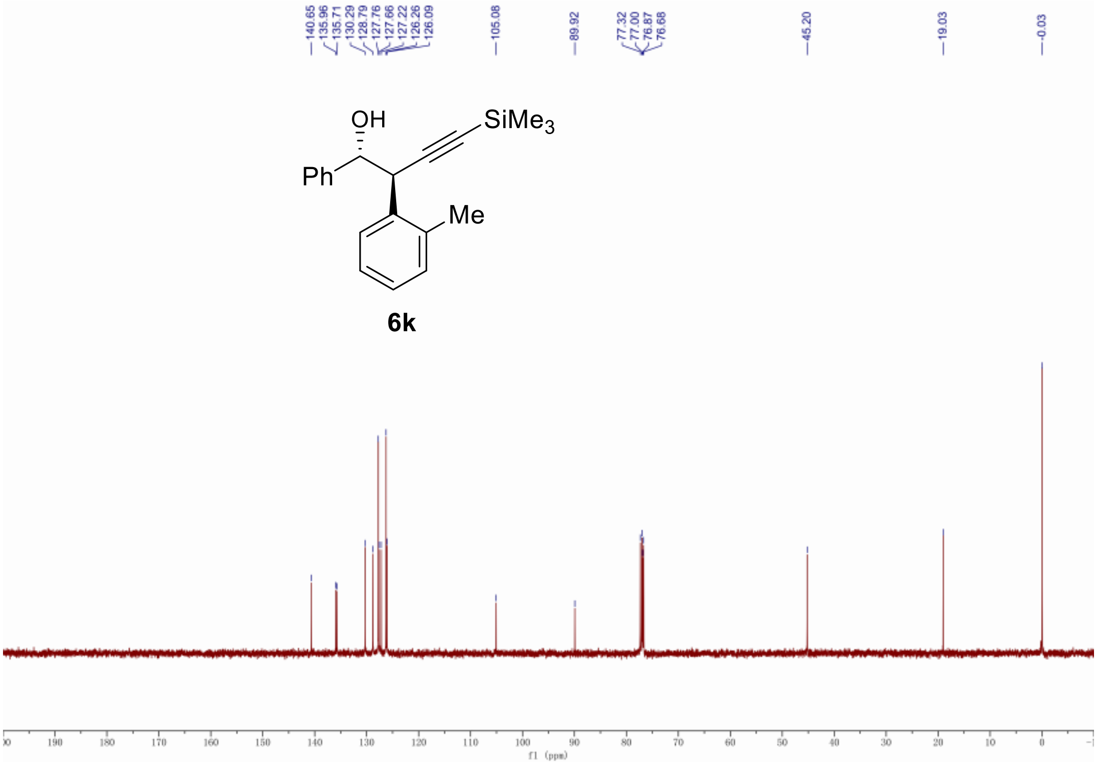

(¹H NMR, 400 MHz, $CDCl_3$)

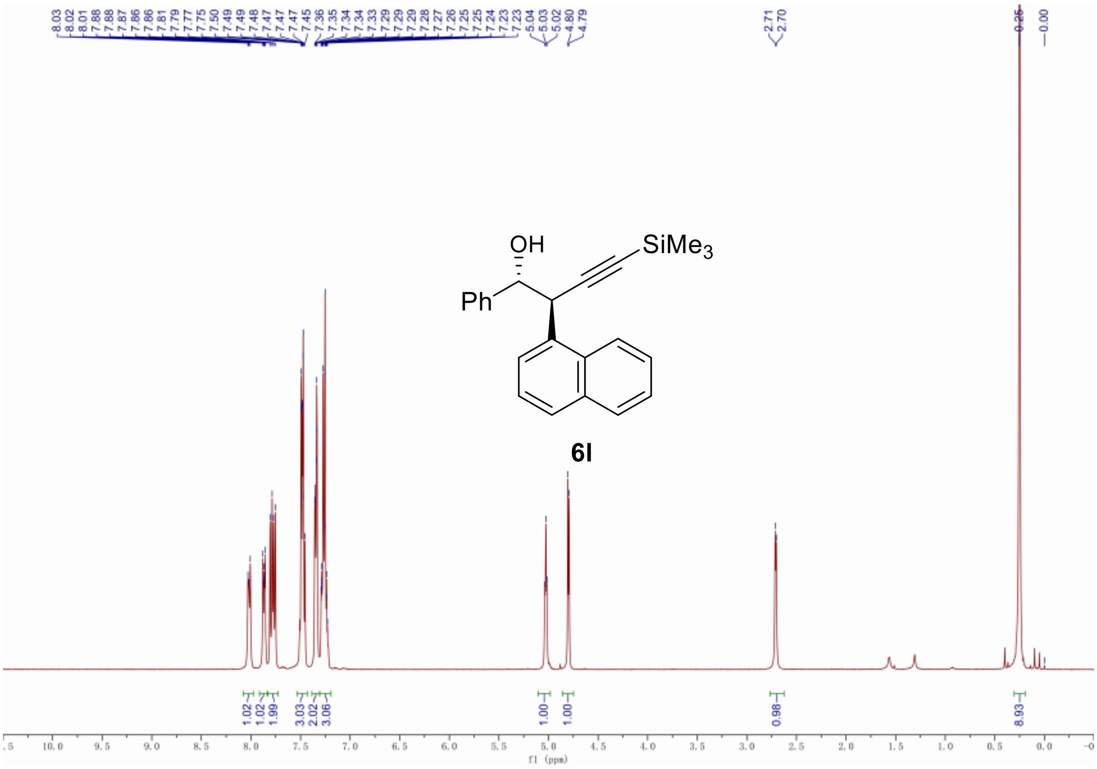

(^{13}C NMR, 100 MHz, $CDCl_3$)

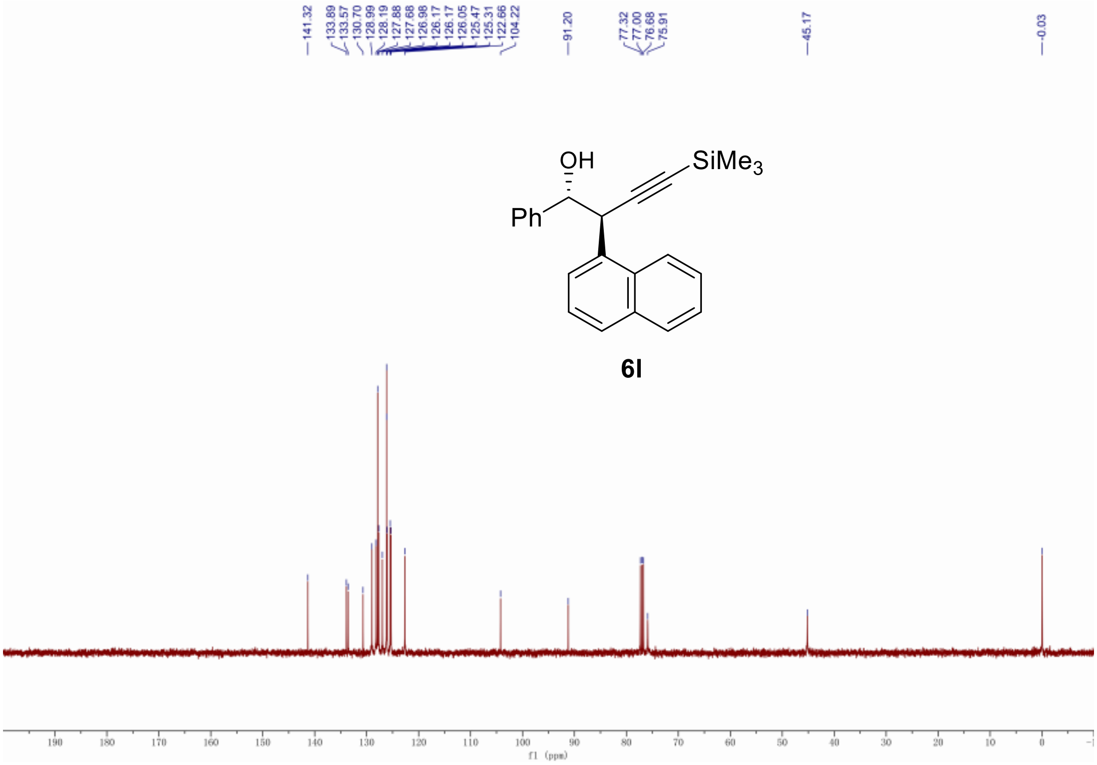

(¹H NMR, 400 MHz, $CDCl_3$)

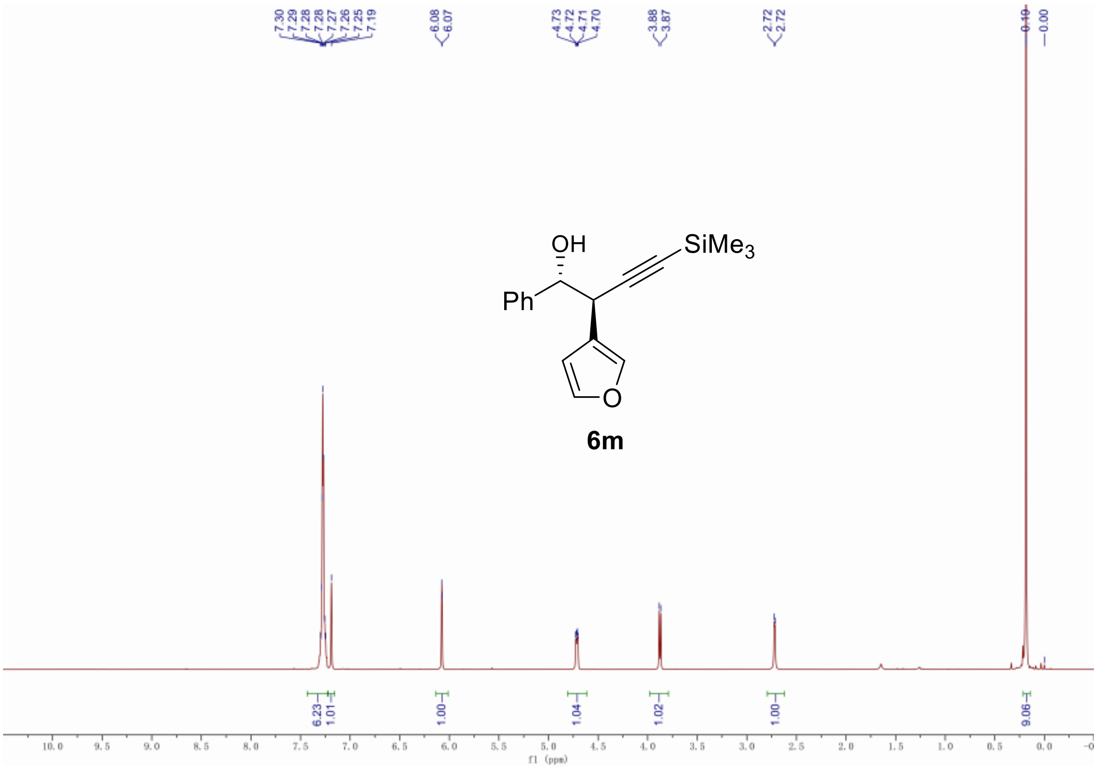

(¹³C NMR, 100 MHz, $CDCl_3$)

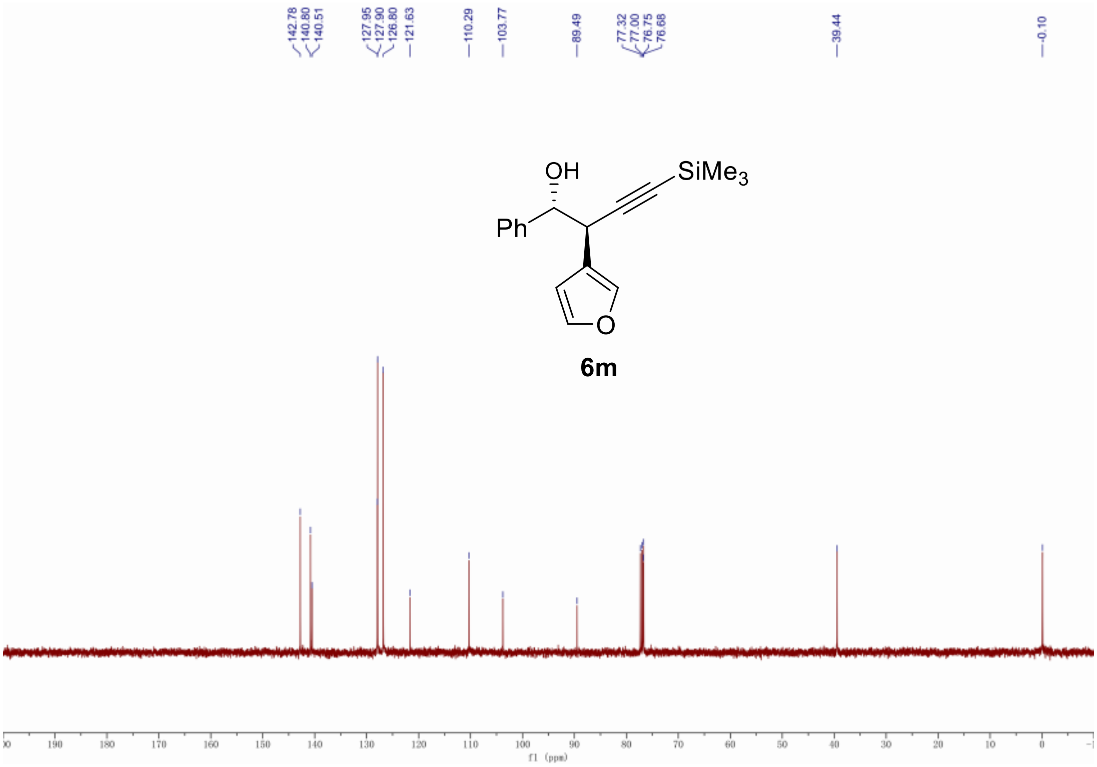

(¹H NMR, 400 MHz, $CDCl_3$)

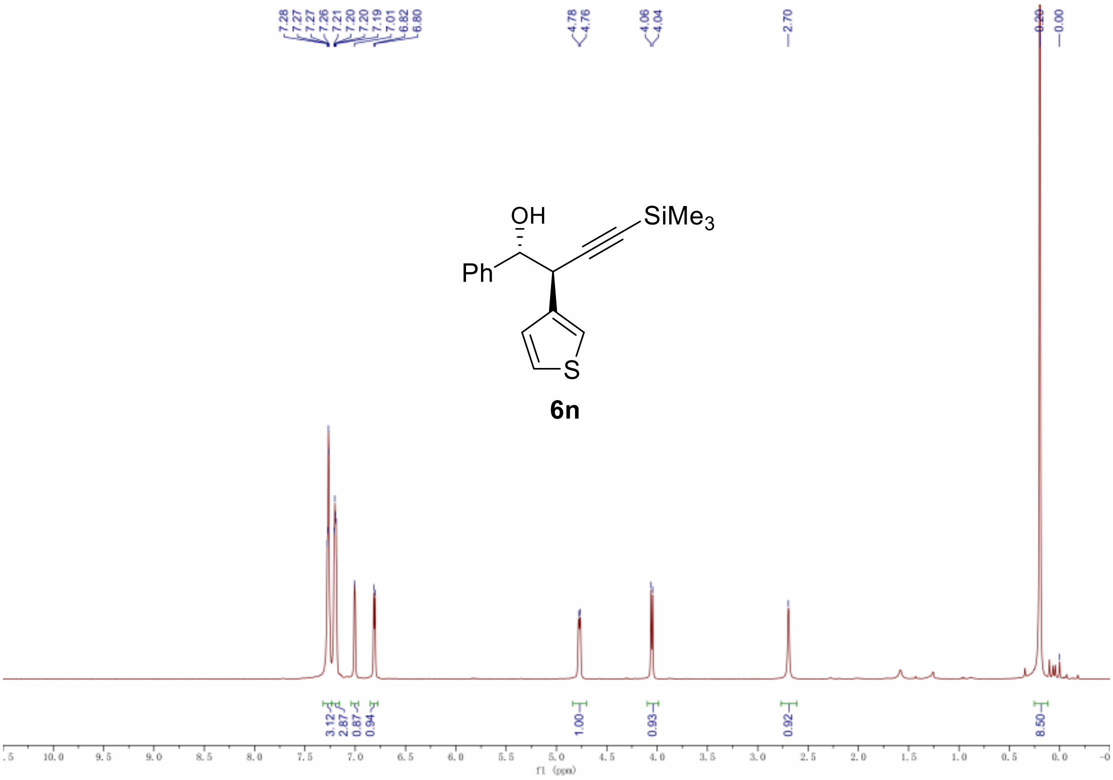

6n

(¹³C NMR, 100 MHz, $CDCl_3$)

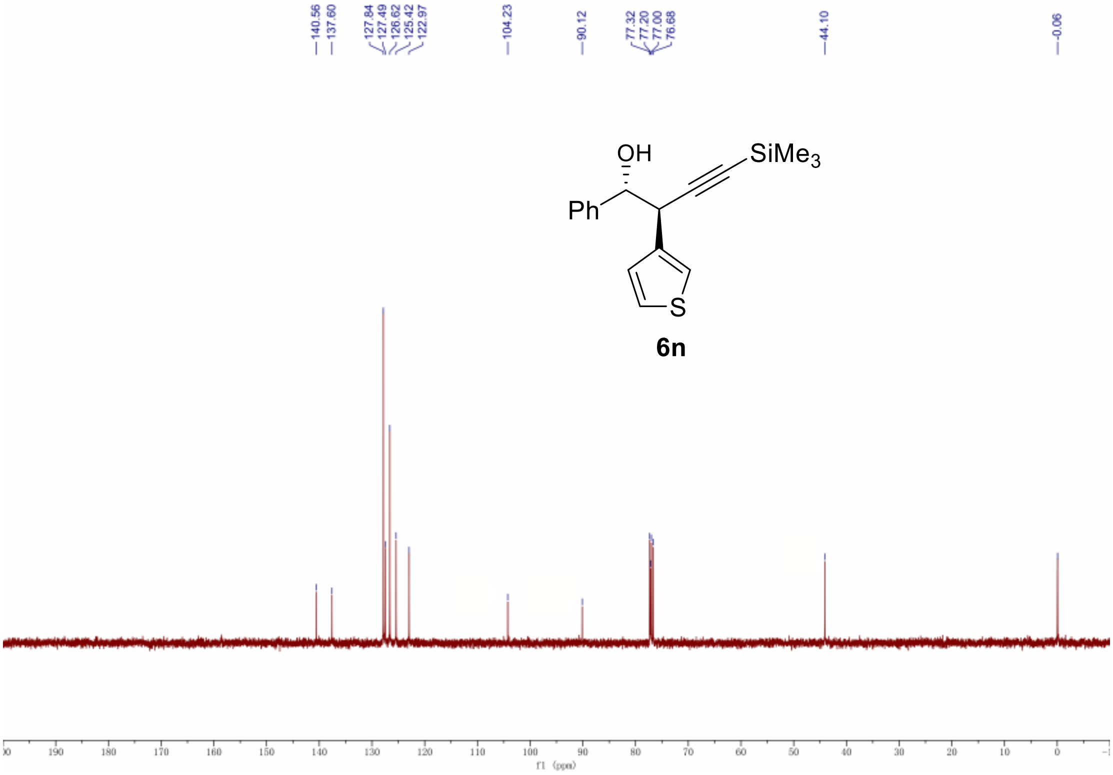

6n

(^{1}H NMR, 400 MHz, $CDCl_3$)

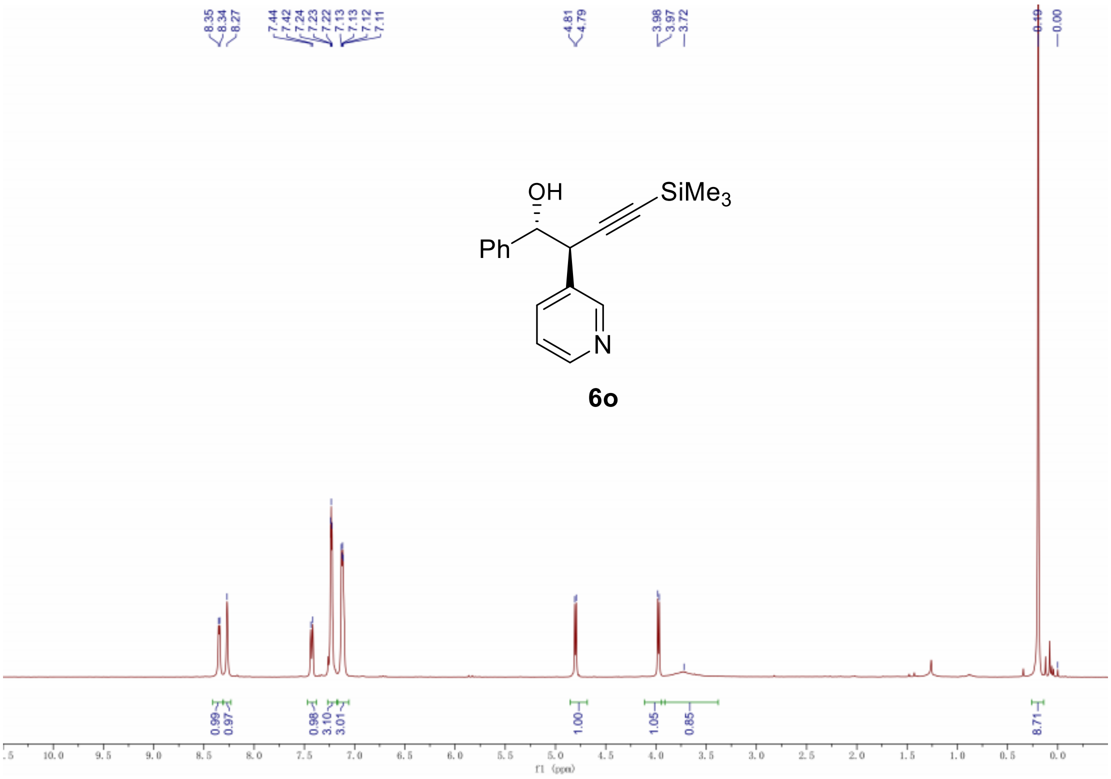

(^{13}C NMR, 100 MHz, $CDCl_3$)

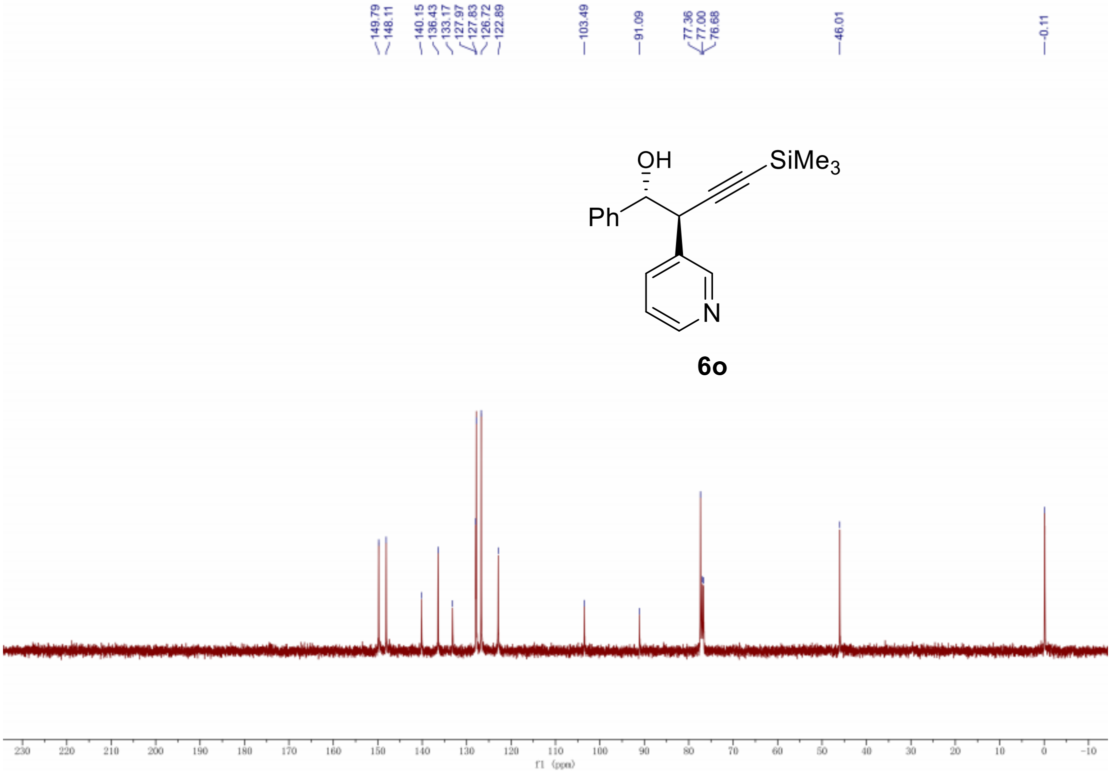

（^{1}H NMR, 400 MHz, $CDCl_3$）

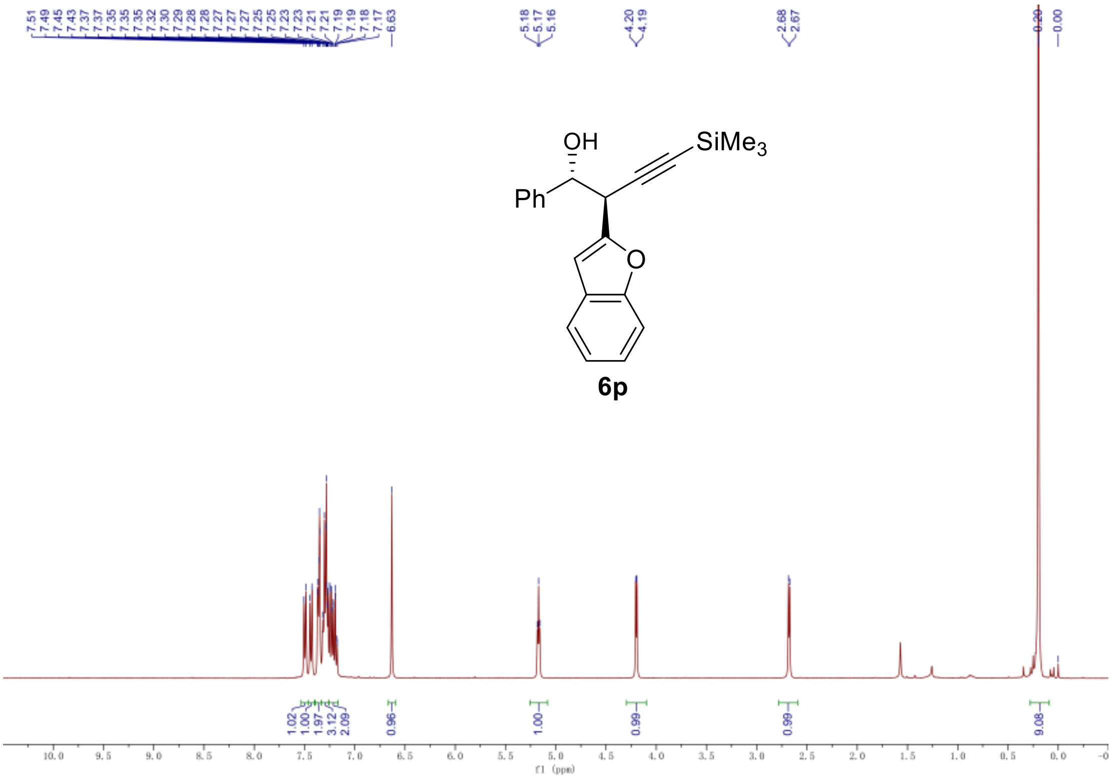

(^{13}C NMR, 100 MHz, $CDCl_3$)

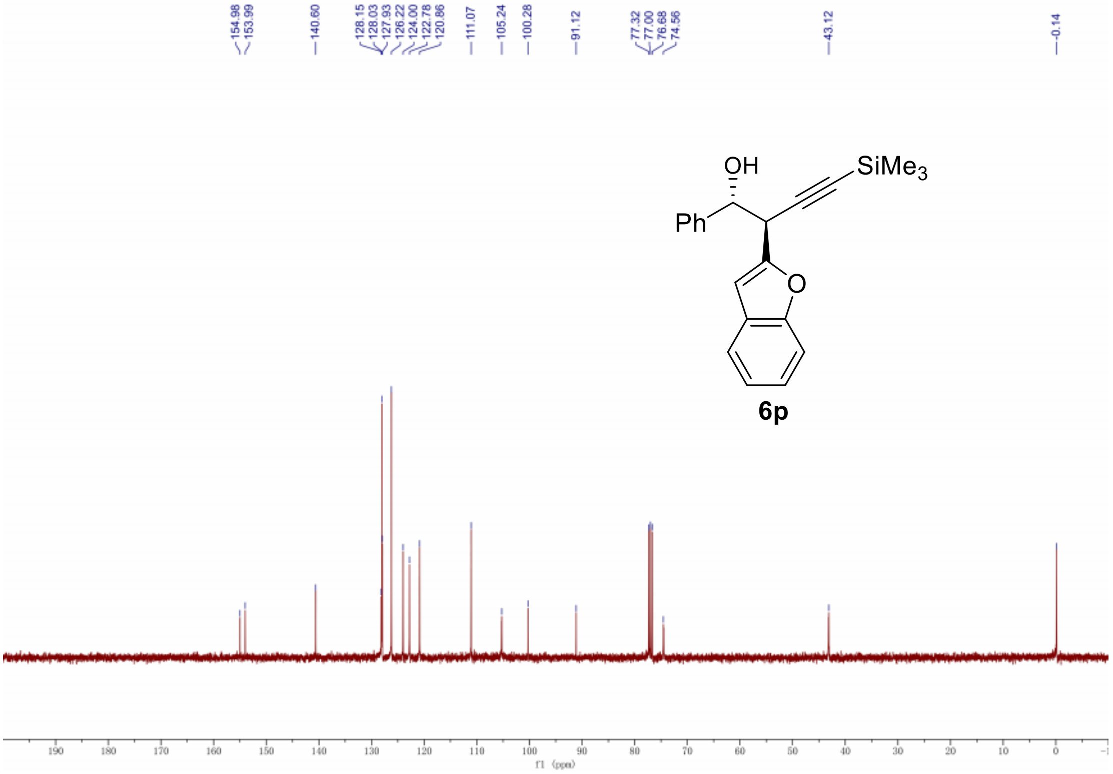

（^{1}H NMR, 400 MHz, $CDCl_3$）

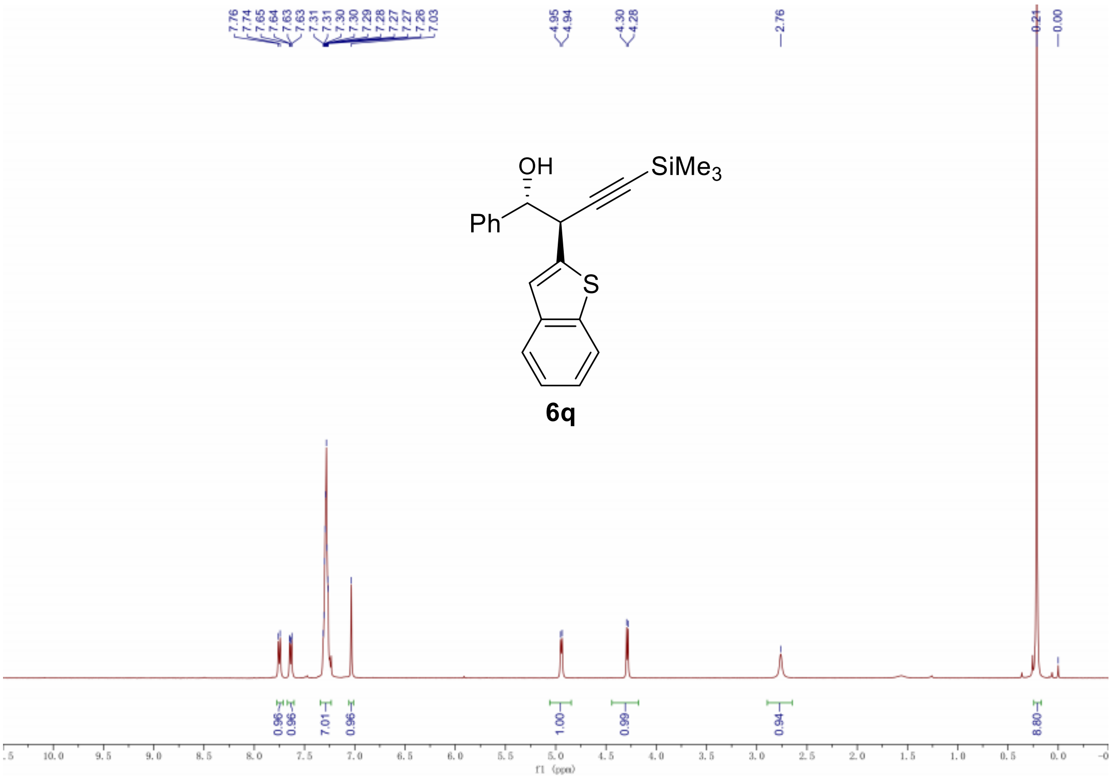

(^{13}C NMR, 100 MHz, $CDCl_3$)

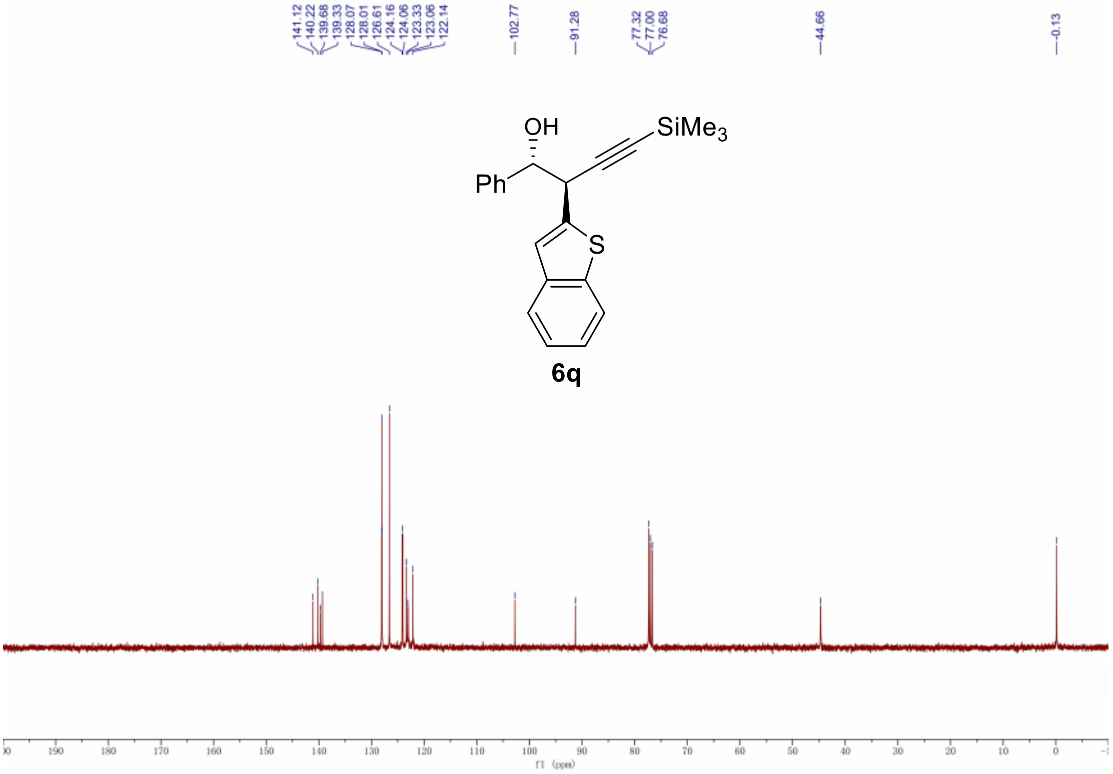

（^{1}H NMR, 400 MHz, CDCl$_3$）

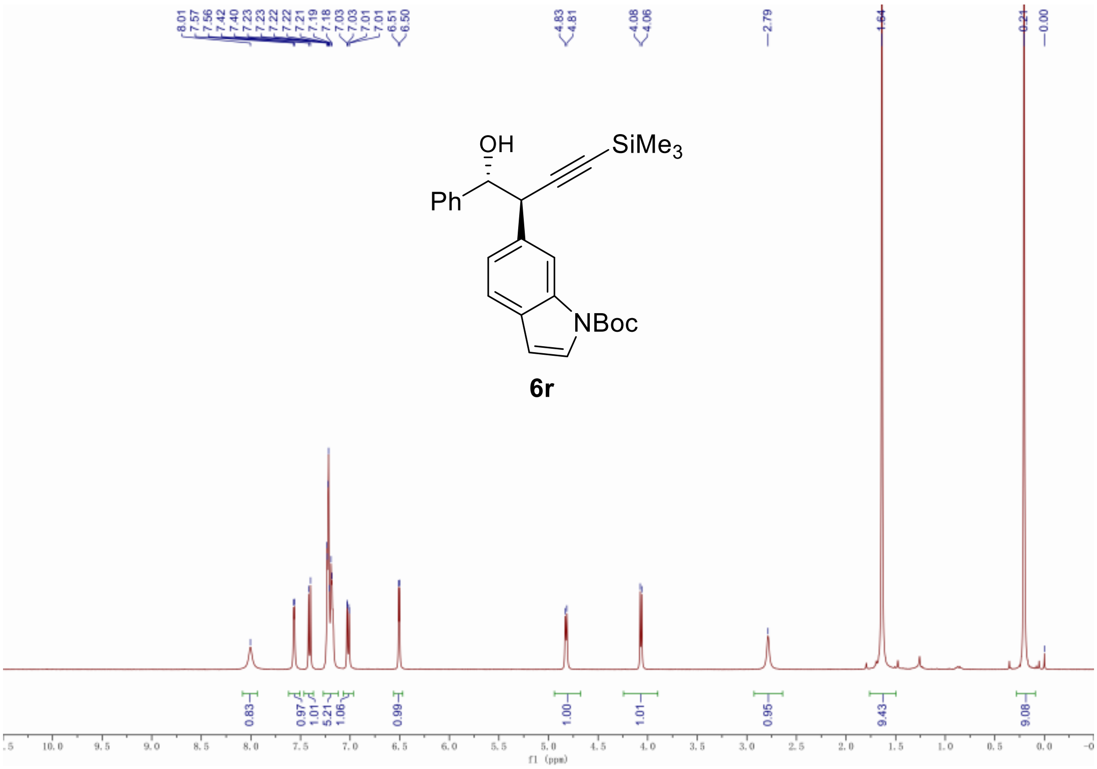

(^{13}C NMR, 100 MHz, CDCl$_3$)

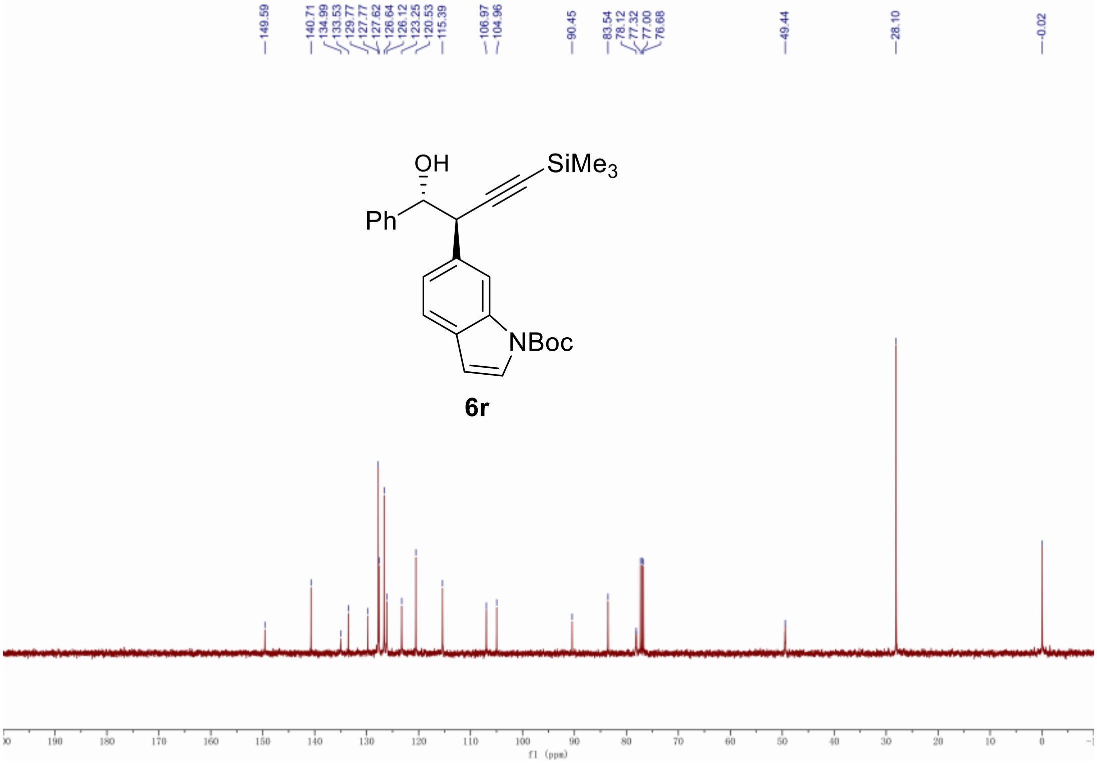

(¹H NMR, 400 MHz, $CDCl_3$)

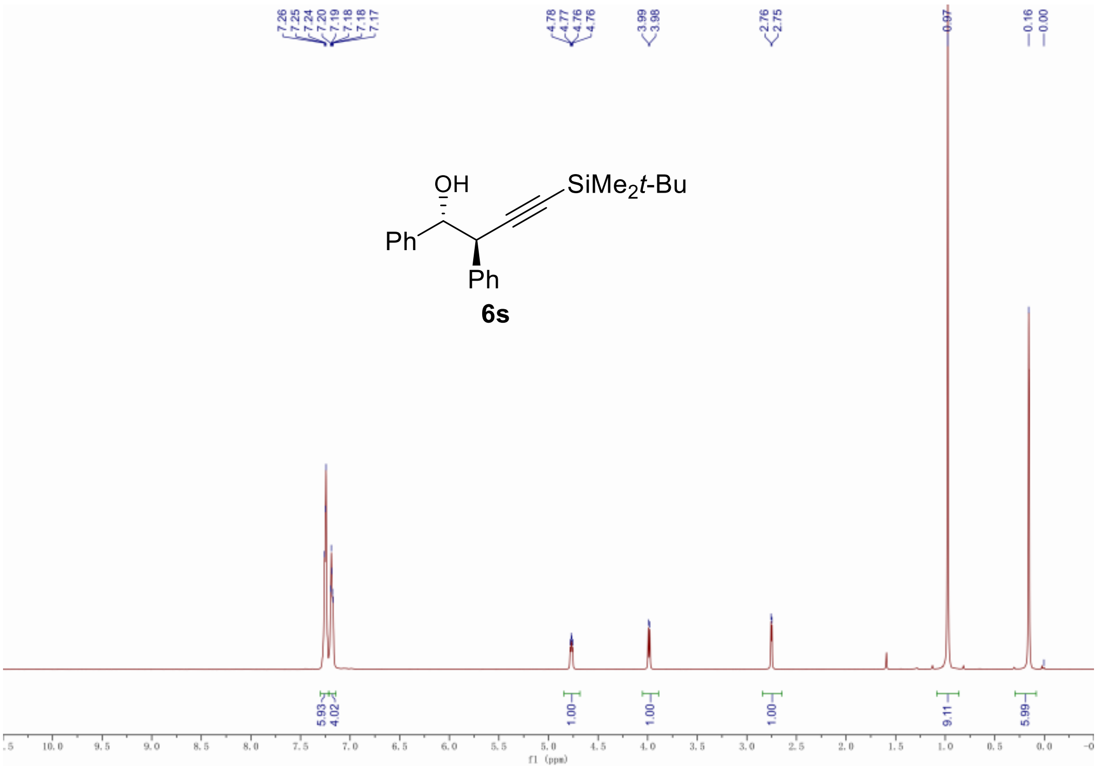

(^{13}C NMR, 100 MHz, $CDCl_3$)

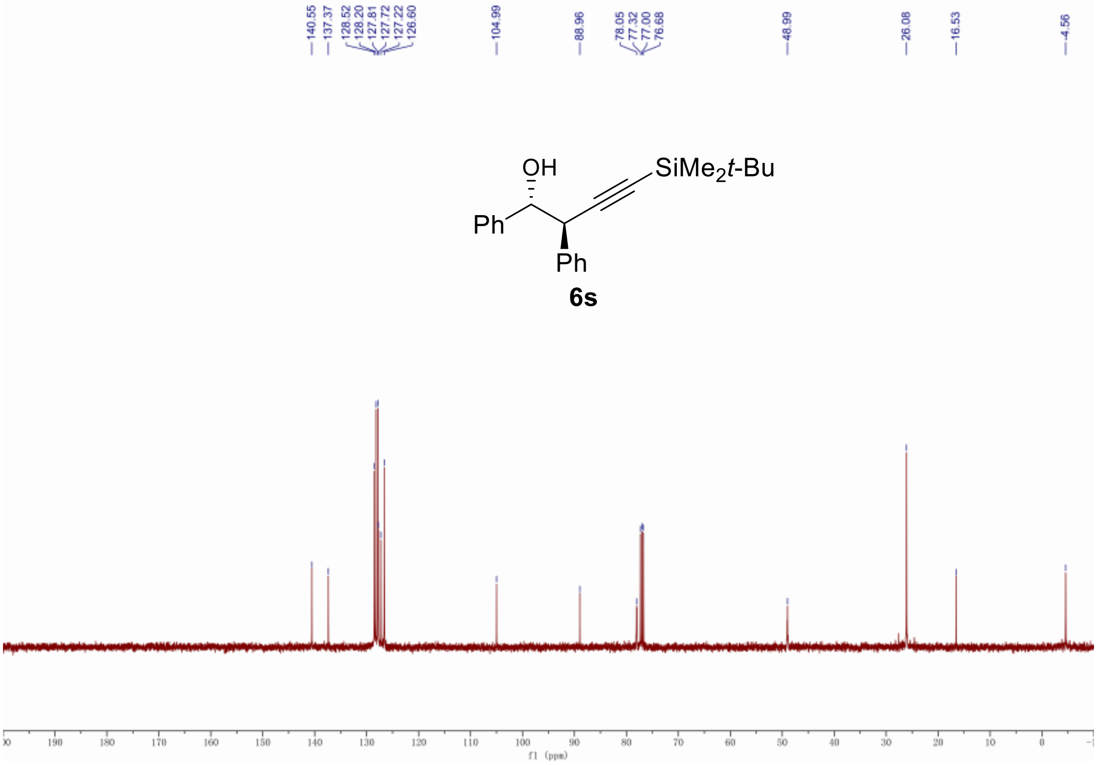

(¹H NMR, 400 MHz, $CDCl_3$)

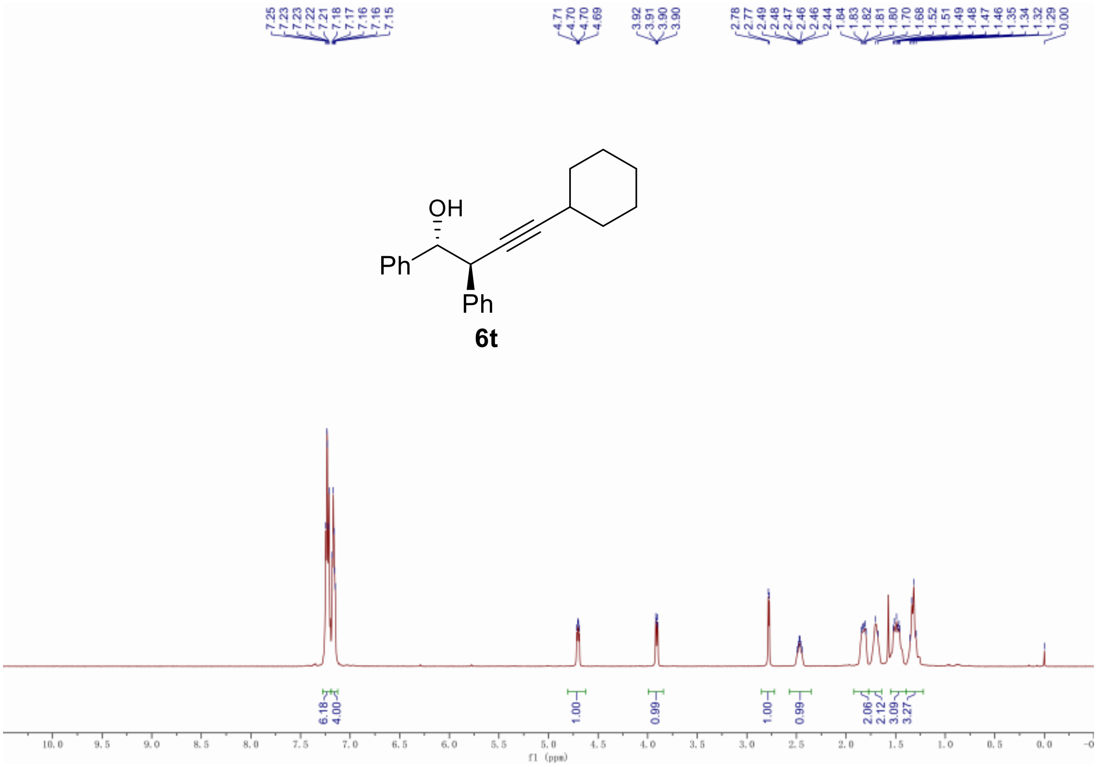

(^{13}C NMR, 100 MHz, $CDCl_3$)

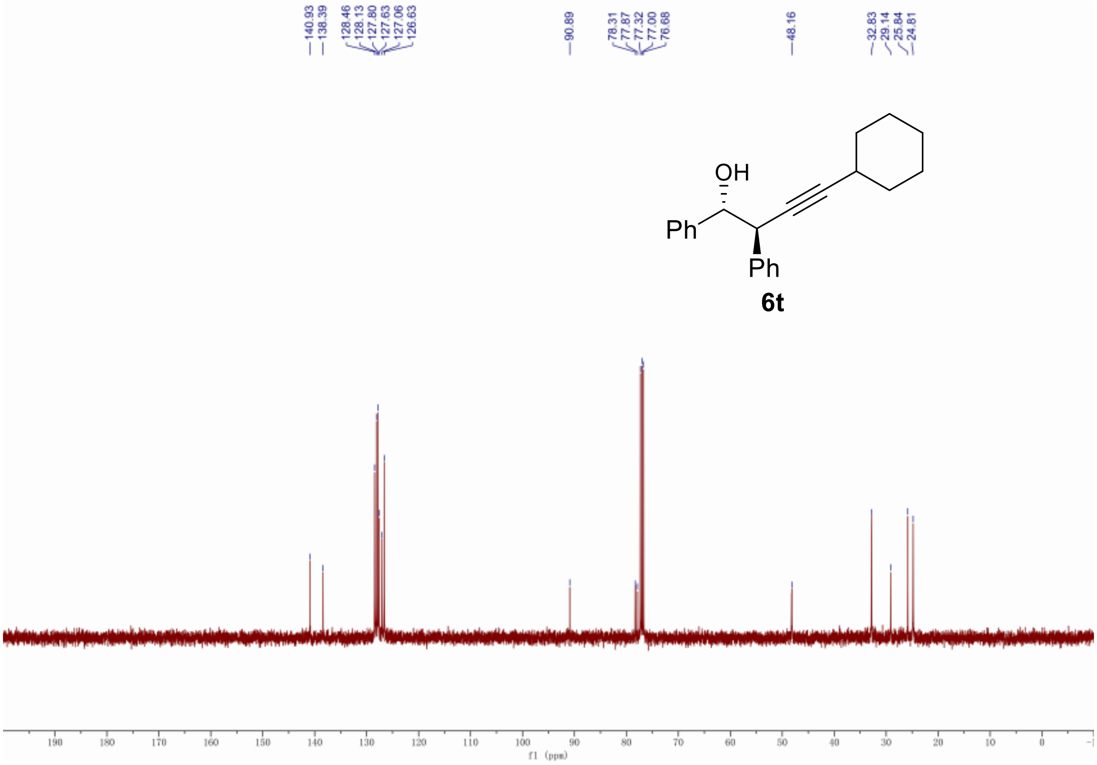

(¹H NMR, 400 MHz, CDCl₃)

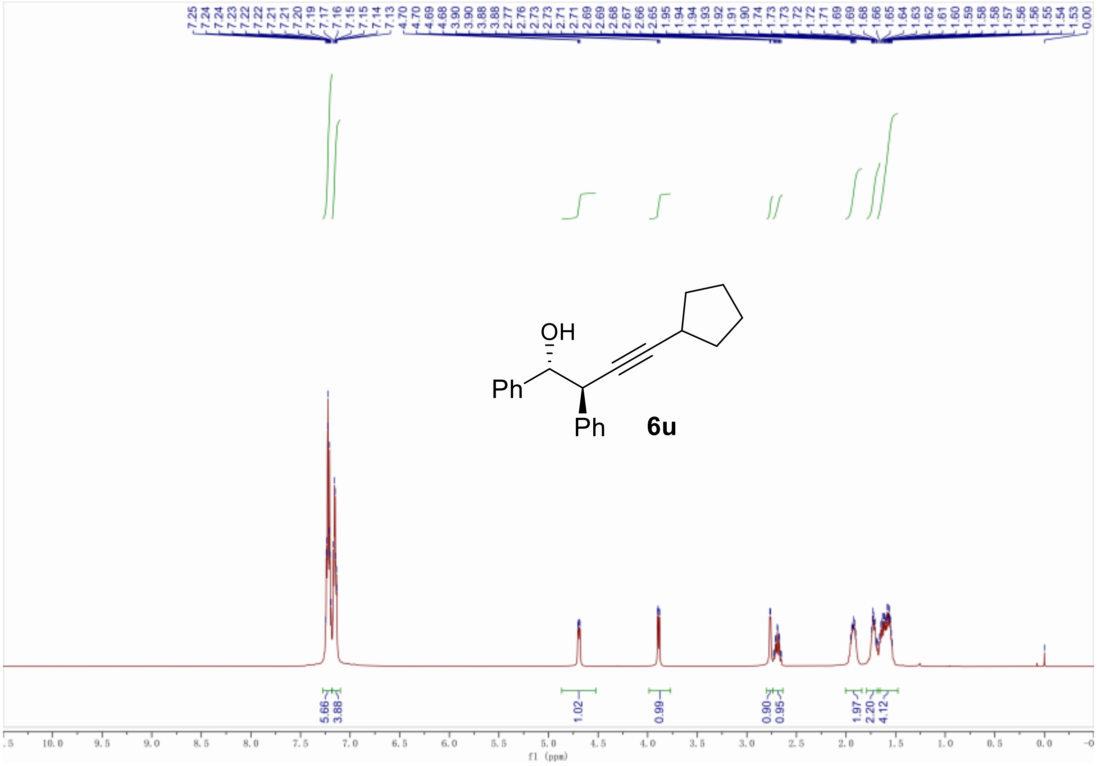

(^{13}C NMR, 100 MHz, CDCl₃)

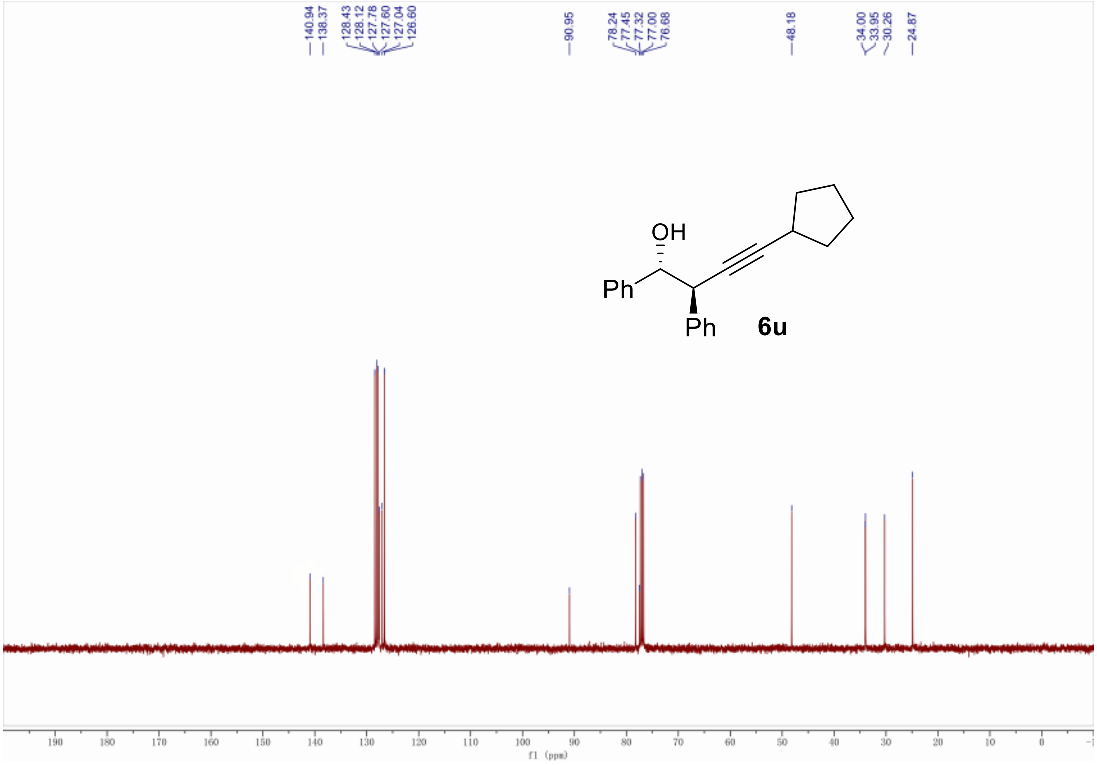

(¹H NMR, 400 MHz, $CDCl_3$)

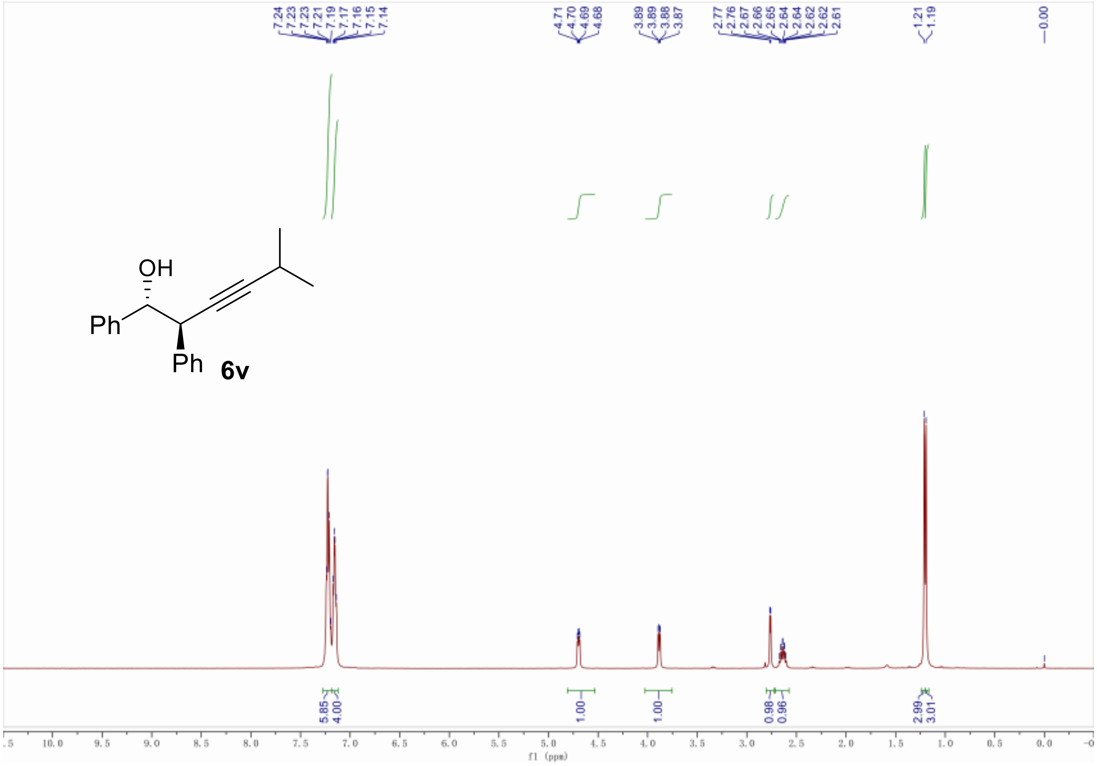

(^{13}C NMR, 100 MHz, $CDCl_3$)

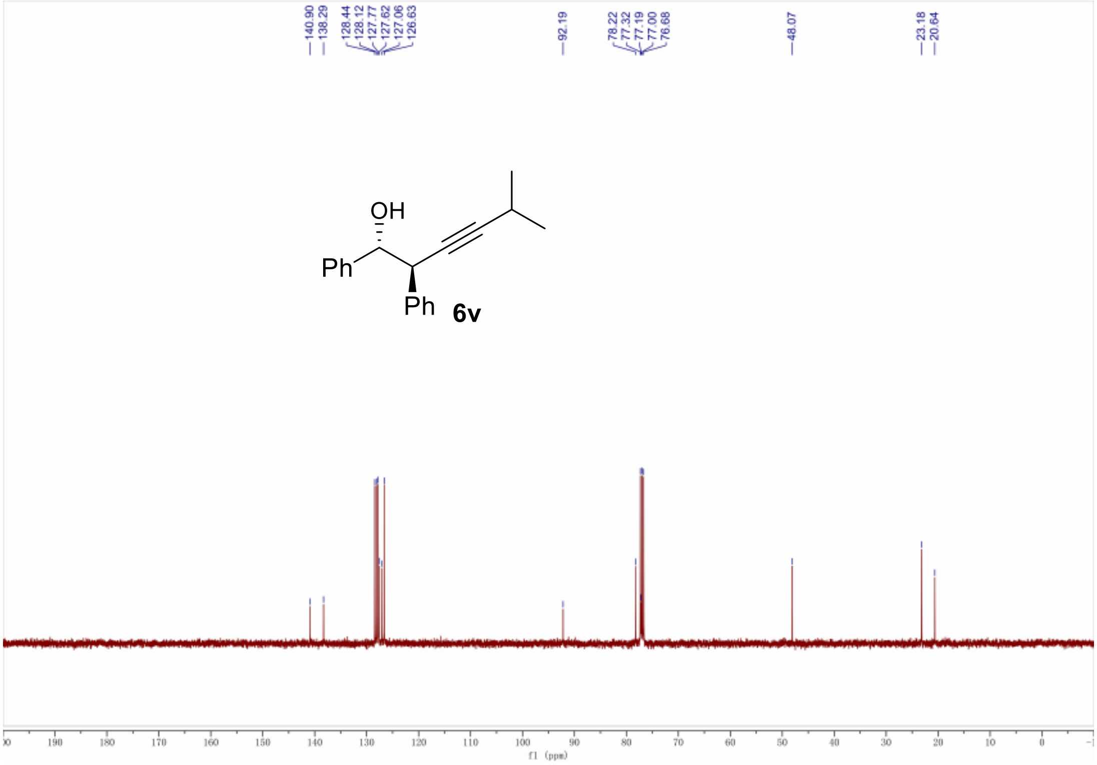

(¹H NMR, 400 MHz, $CDCl_3$)

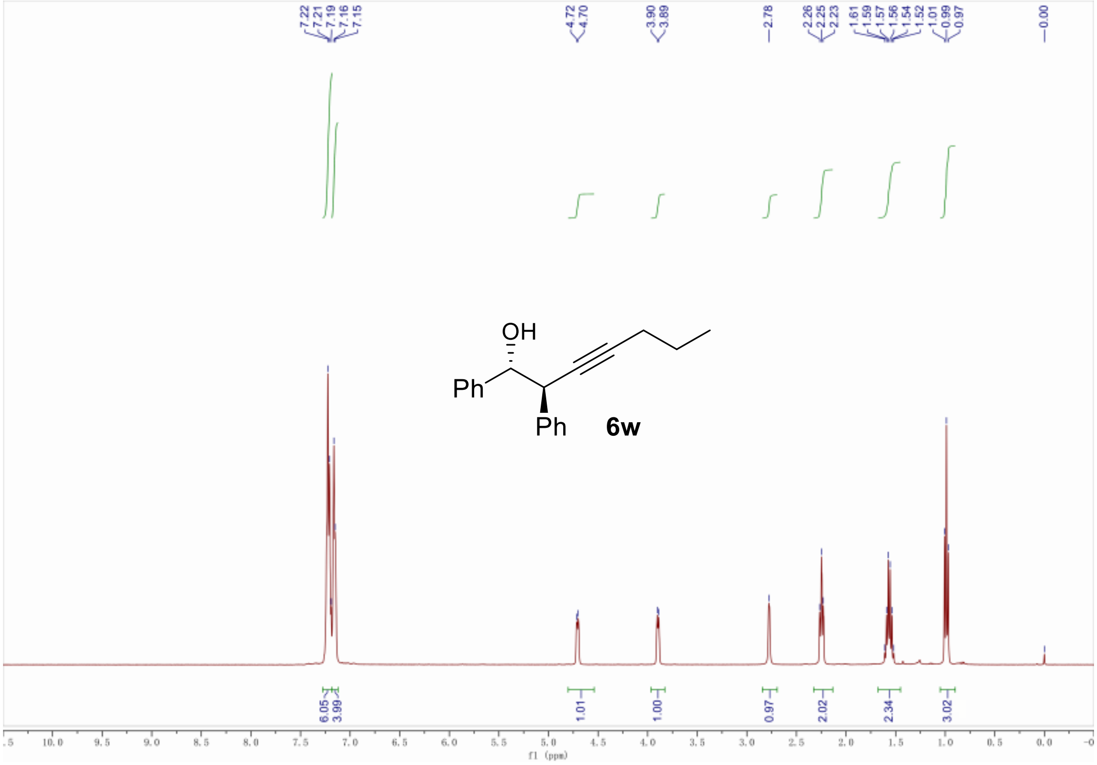

(¹³C NMR, 100 MHz, $CDCl_3$)

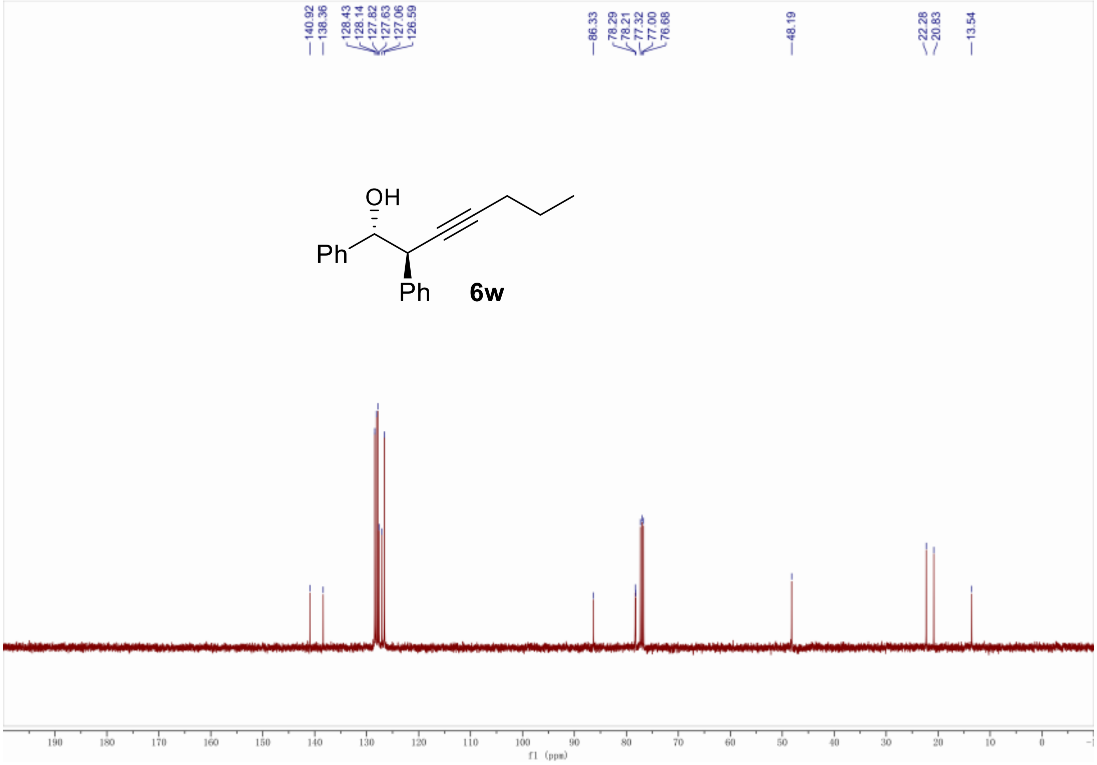

(¹H NMR, 400 MHz, CDCl₃)

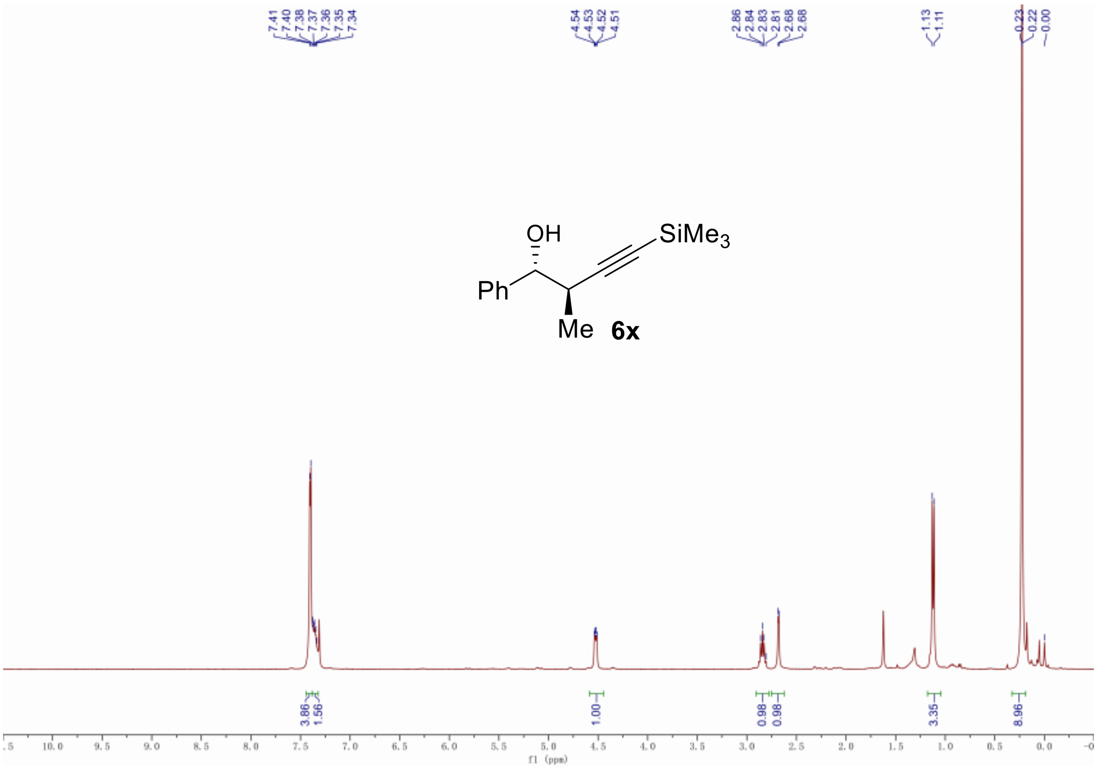

(¹³C NMR, 100 MHz, CDCl₃)

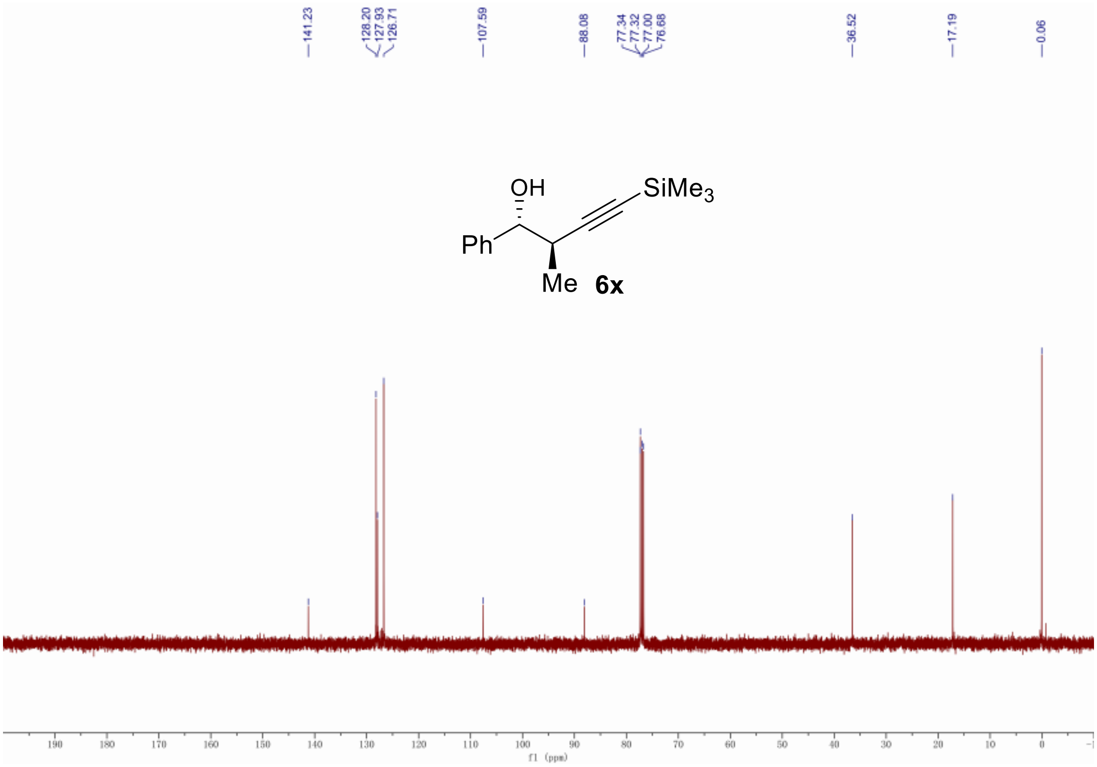

(¹H NMR, 400 MHz, $CDCl_3$)

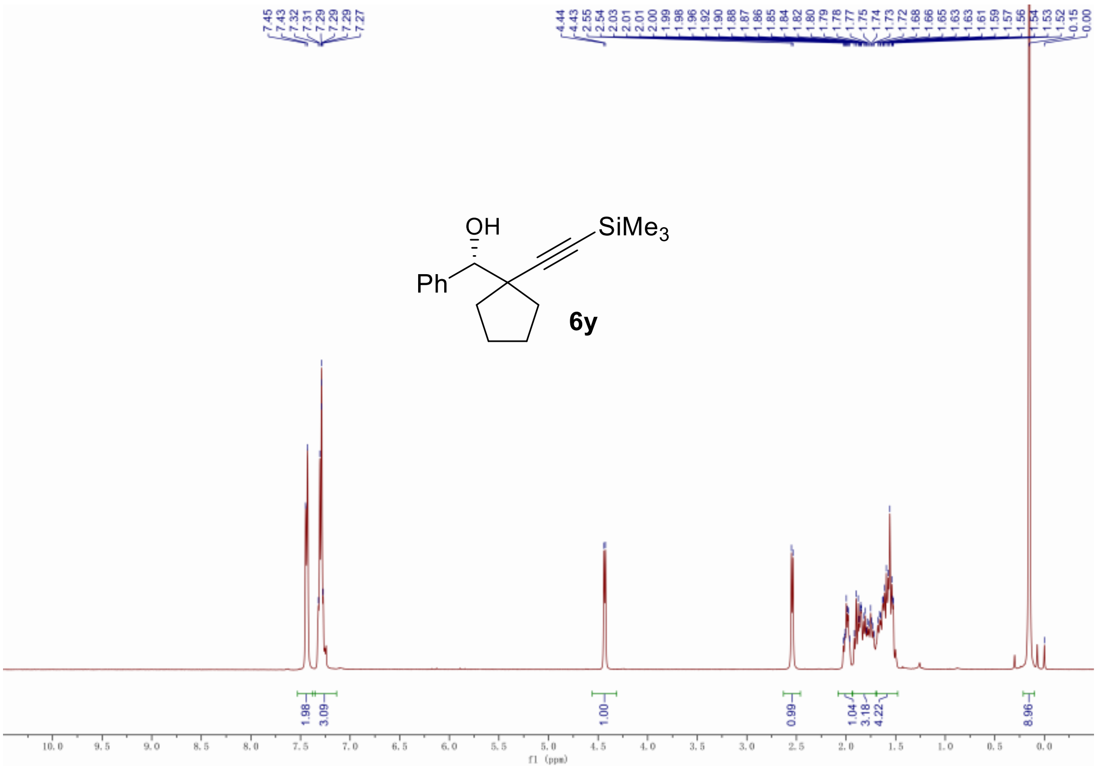

(¹³C NMR, 100 MHz, $CDCl_3$)

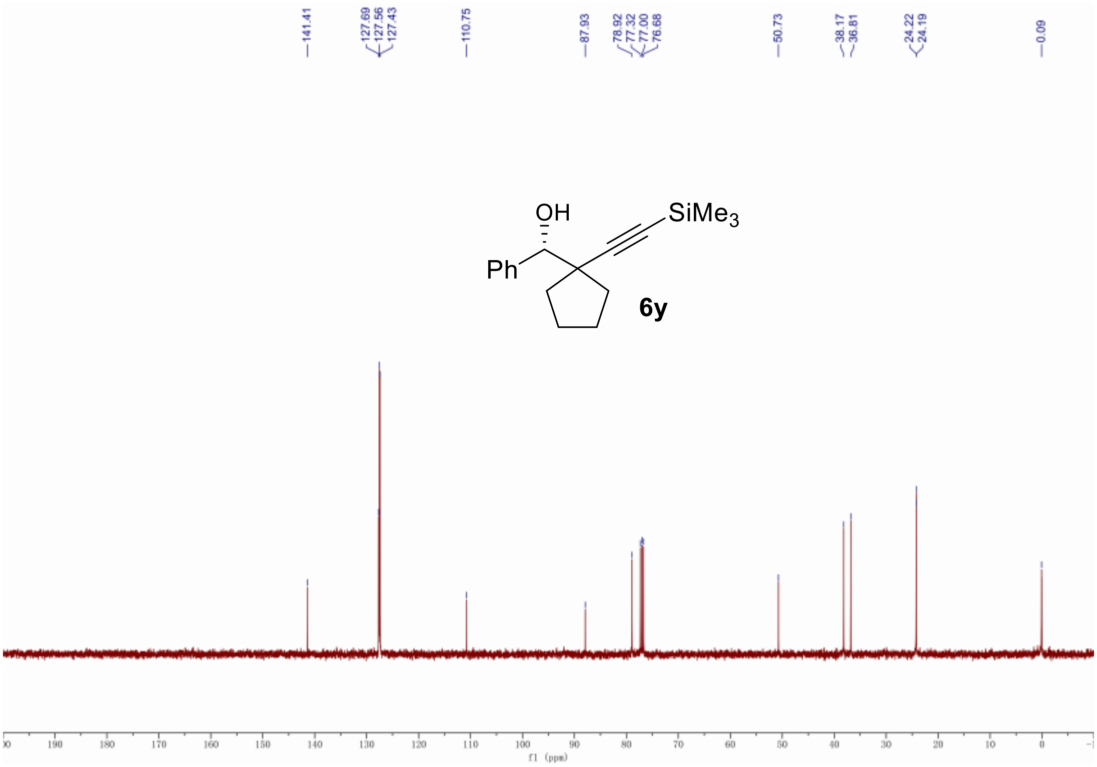

(¹H NMR, 400 MHz, CDCl₃)

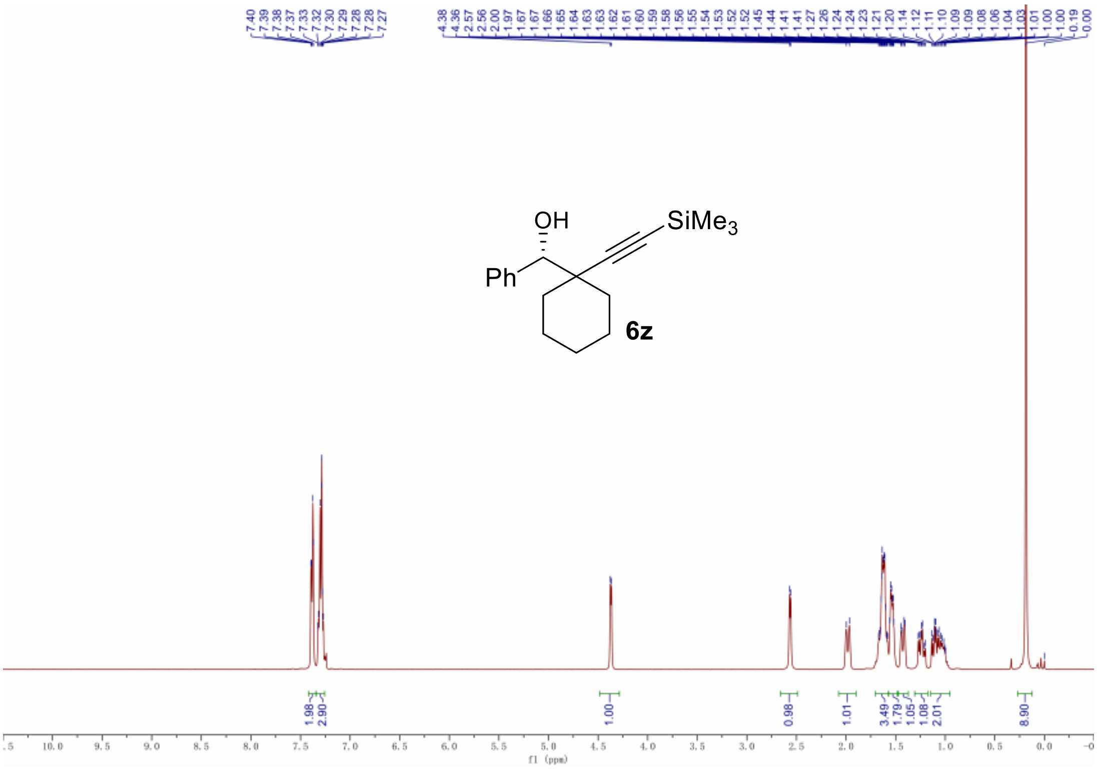

(¹³C NMR, 100 MHz, CDCl₃)

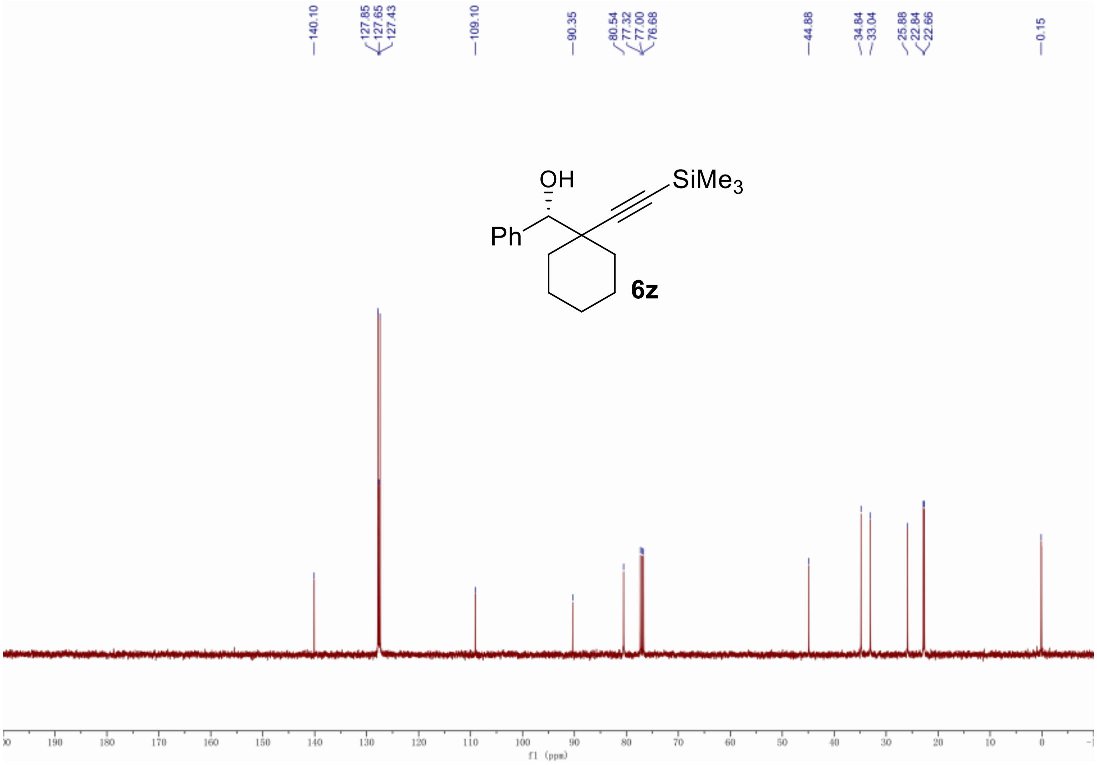

(¹H NMR, 400 MHz, CDCl₃)

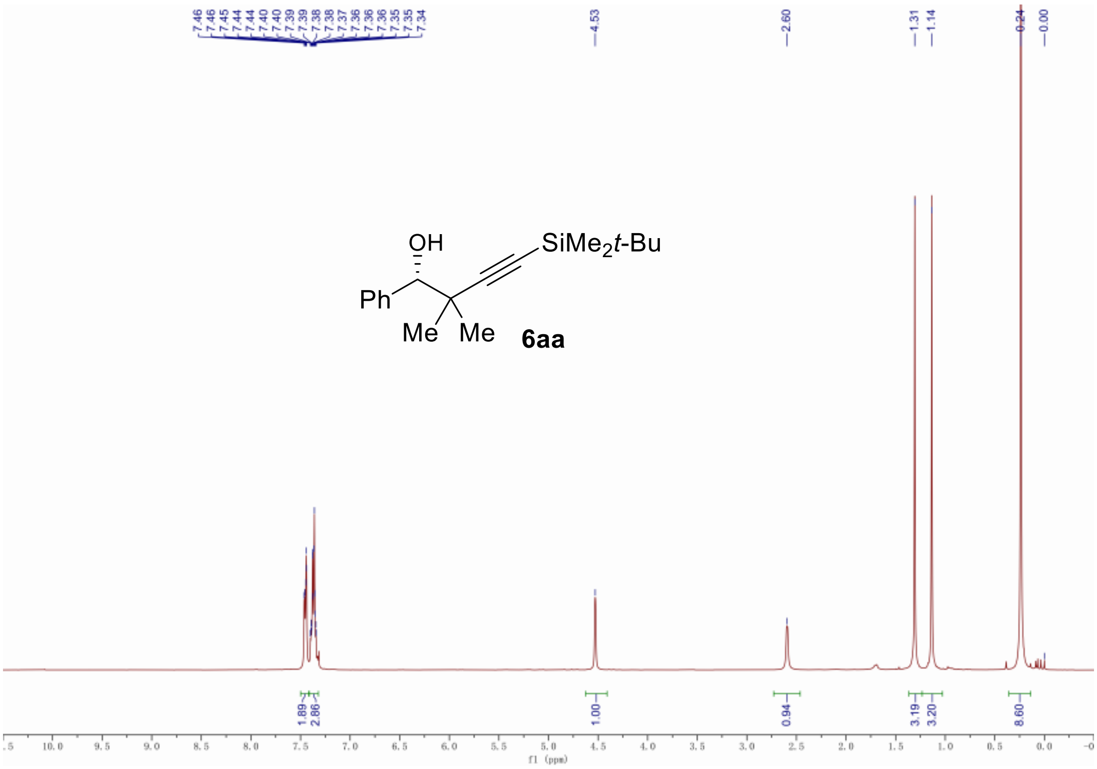

(¹³C NMR, 100 MHz, CDCl₃)

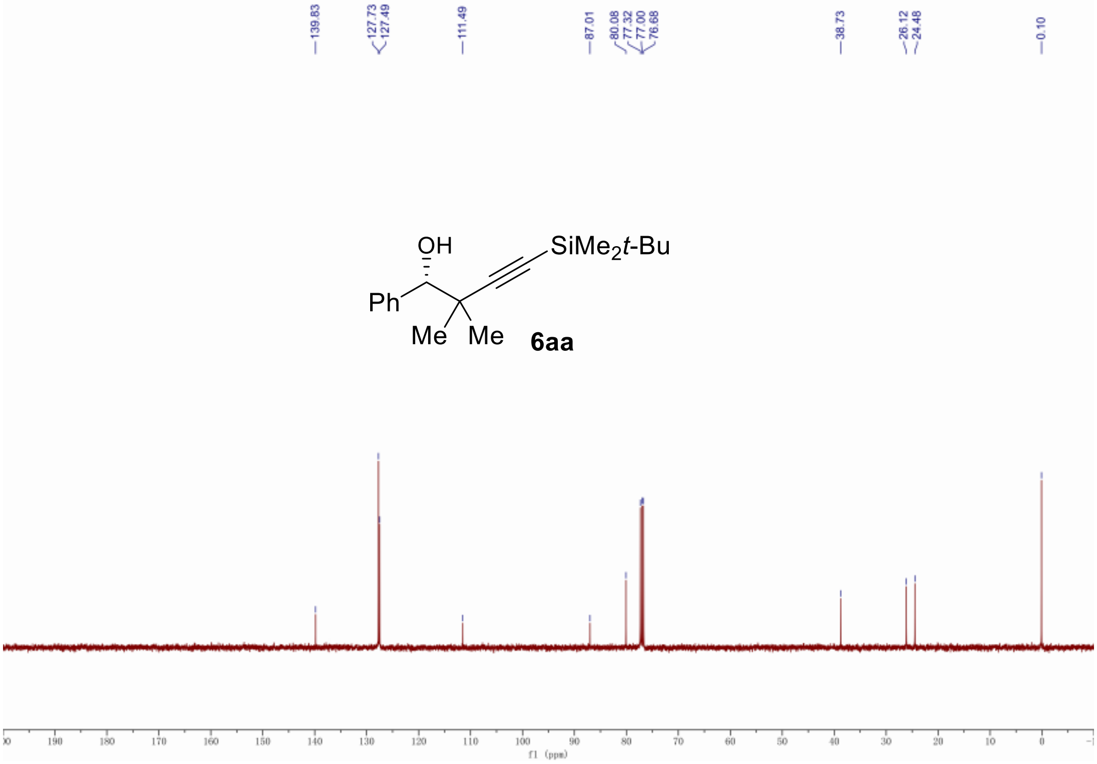

(¹H NMR, 400 MHz, $CDCl_3$)

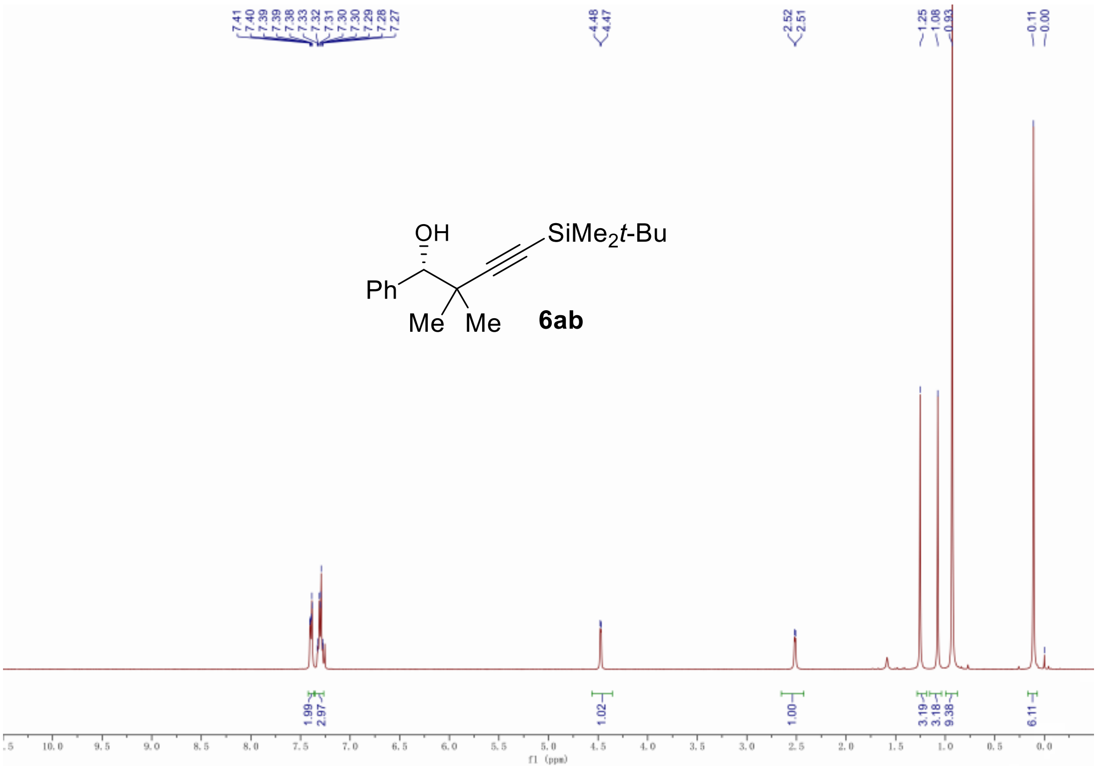

(¹³C NMR, 100 MHz, $CDCl_3$)

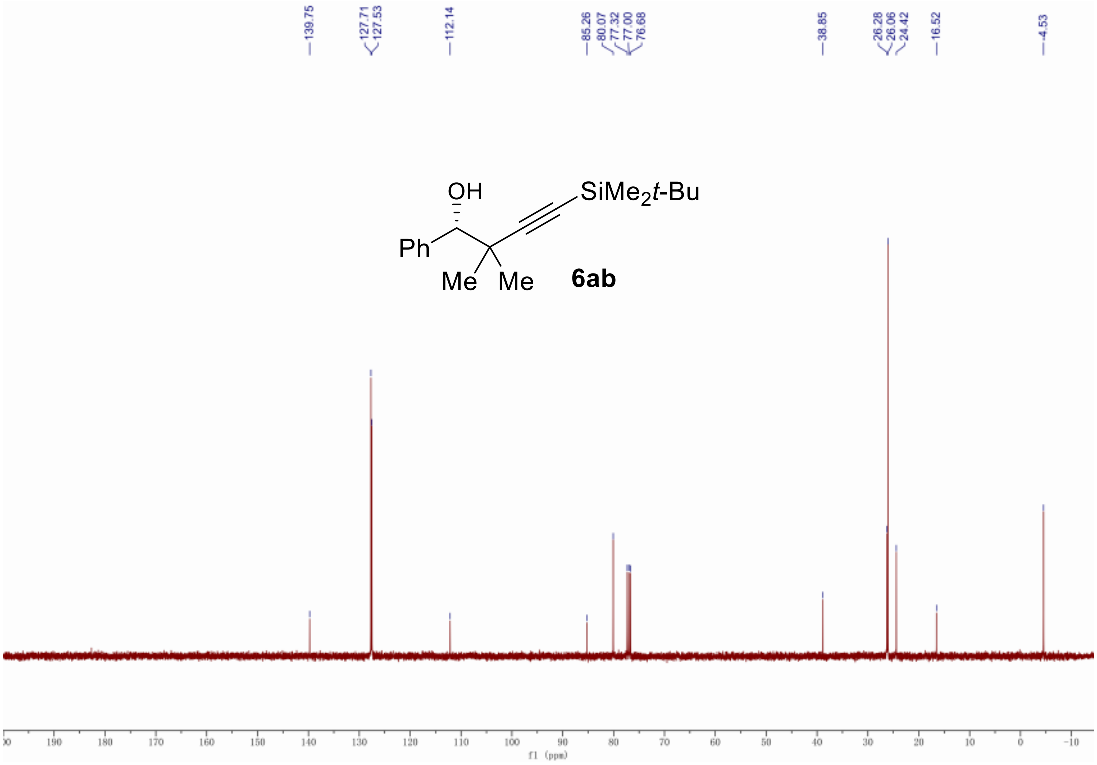

(${}^{1}H$ NMR, 400 MHz, $CDCl_3$)

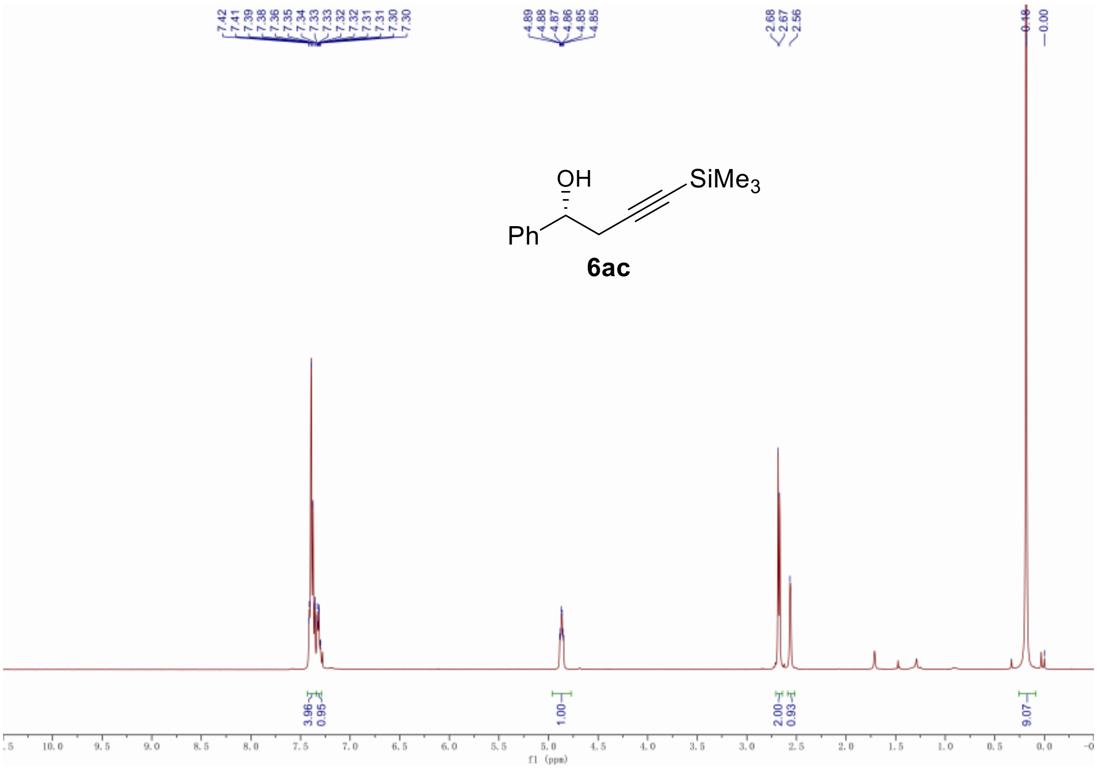

(${}^{13}C$ NMR, 100 MHz, $CDCl_3$)

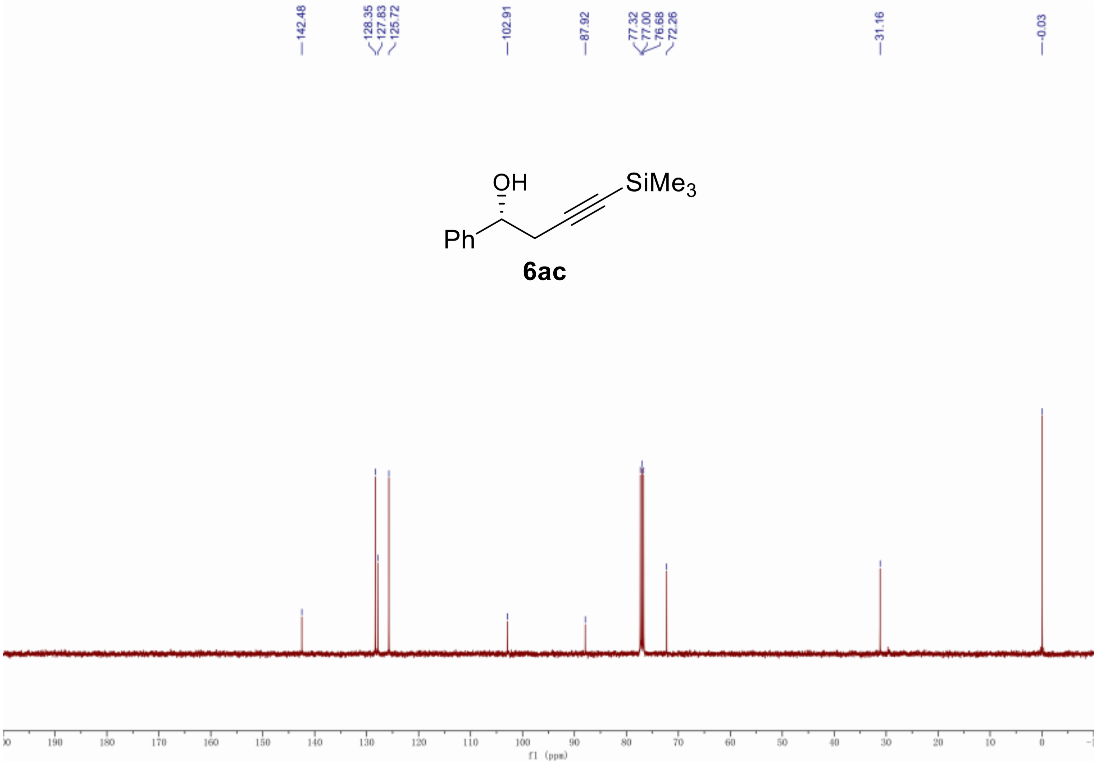

（^{1}H NMR, 400 MHz, CDCl3）

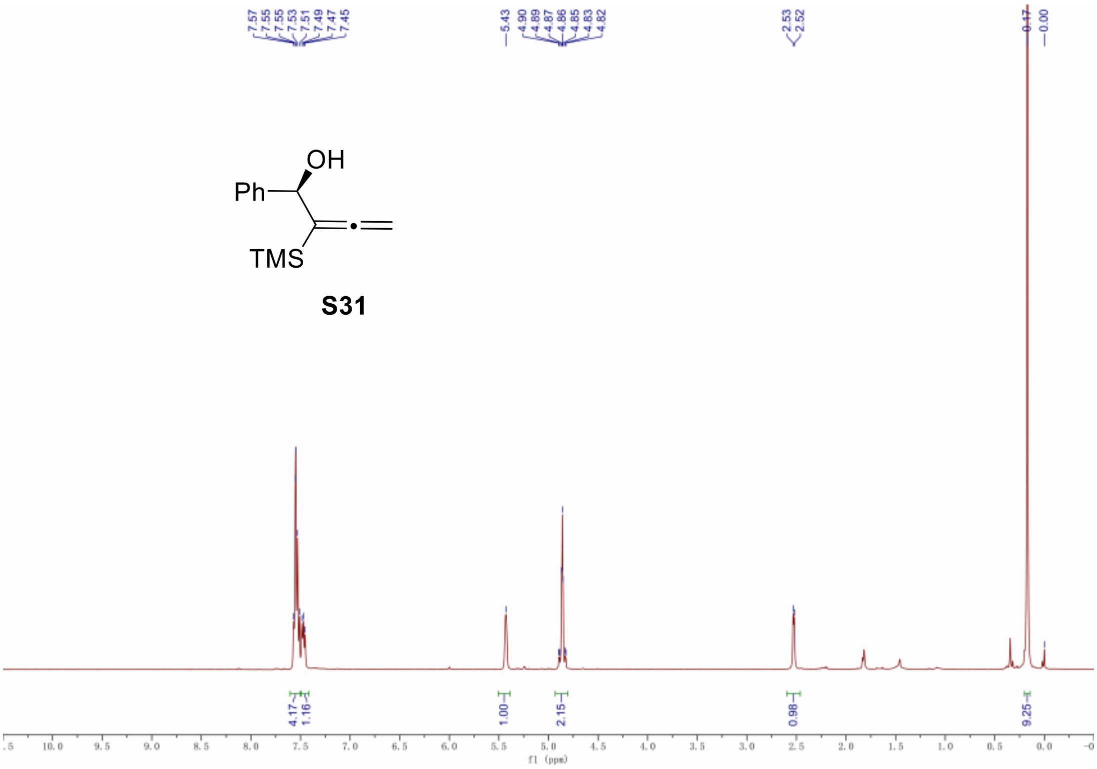

(^{13}C NMR, 100 MHz, CDCl3)

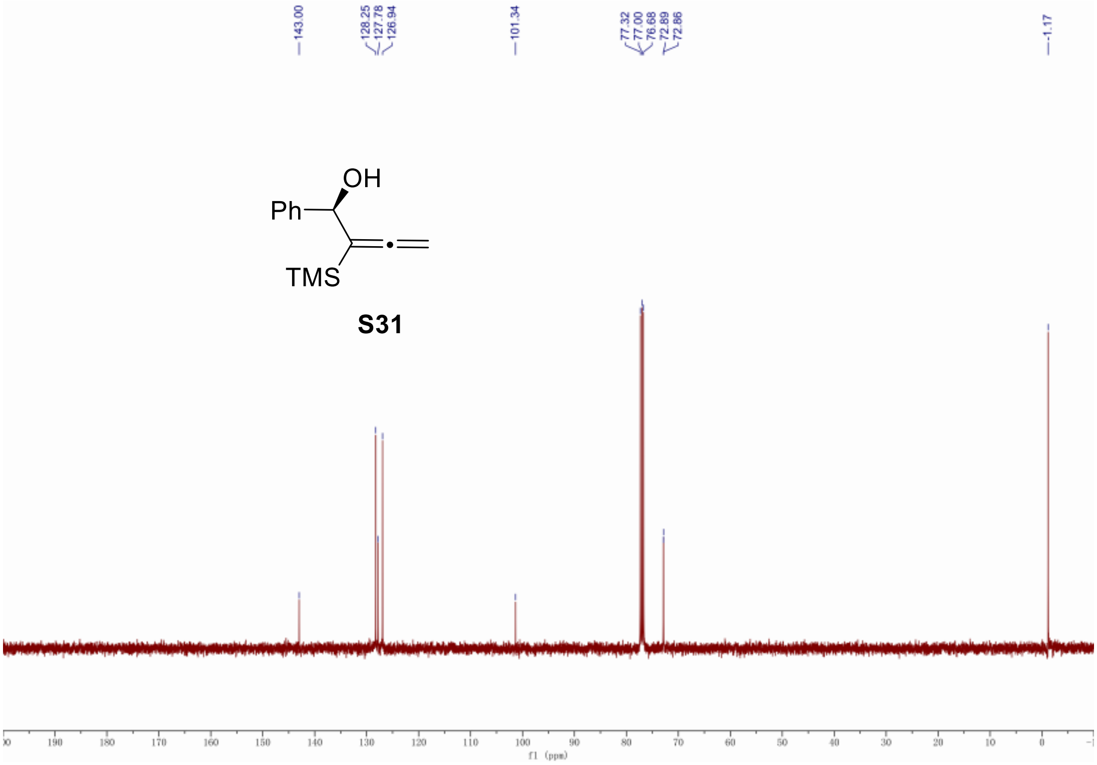

(^{1}H NMR, 400 MHz, $CDCl_3$)

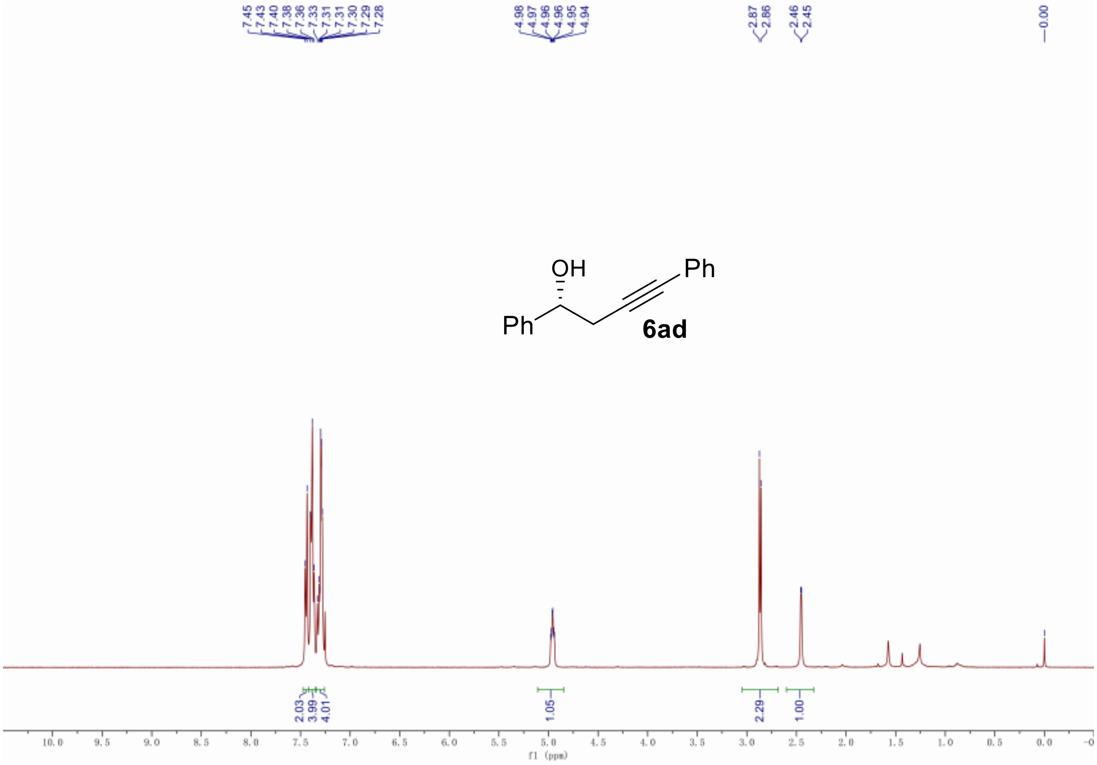

(^{13}C NMR, 100 MHz, $CDCl_3$)

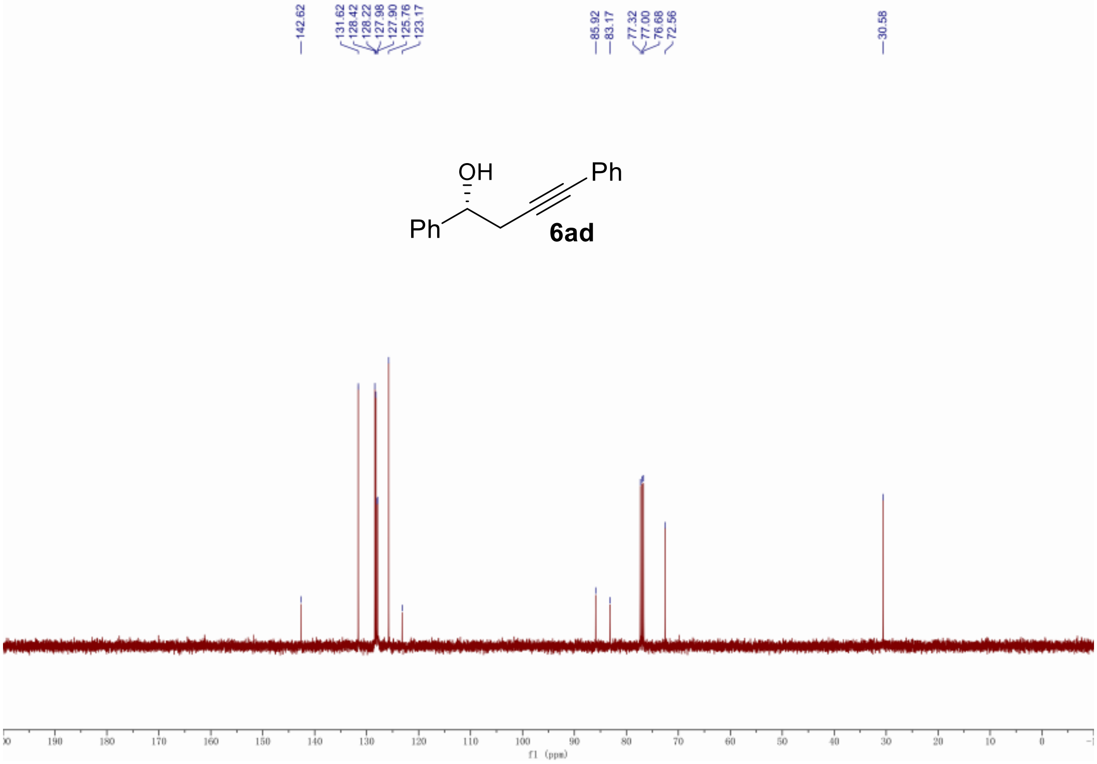

(¹H NMR, 400 MHz, $CDCl_3$)

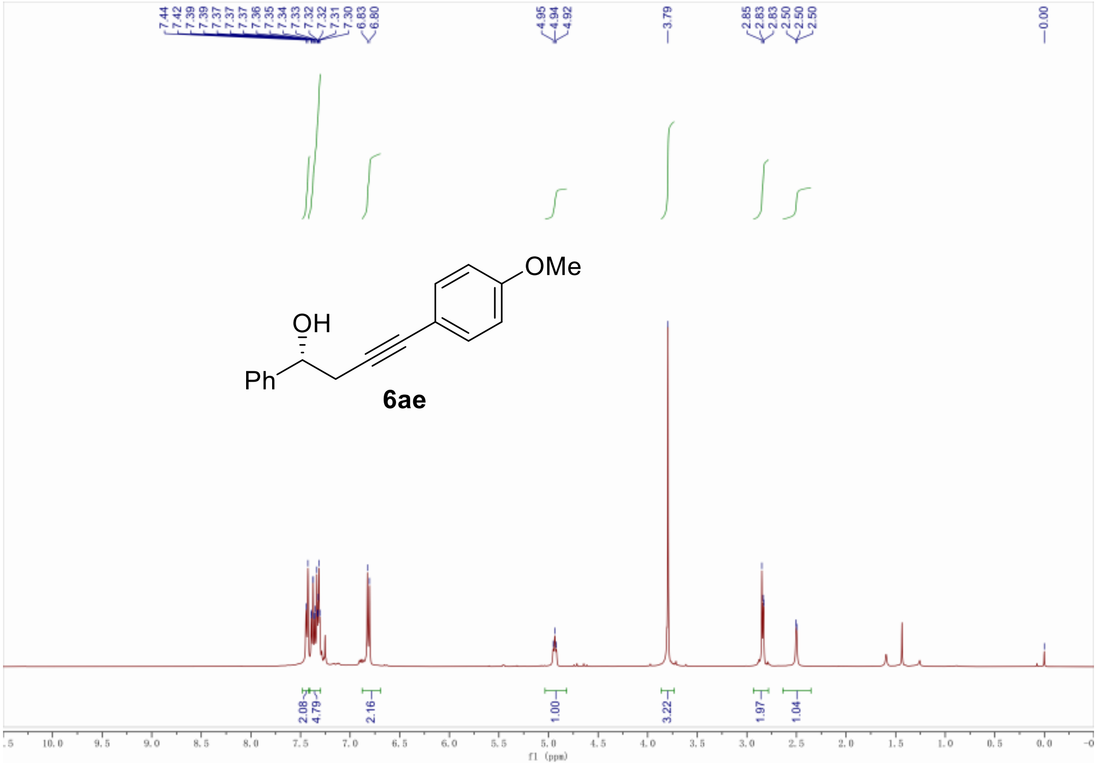

(^{13}C NMR, 100 MHz, $CDCl_3$)

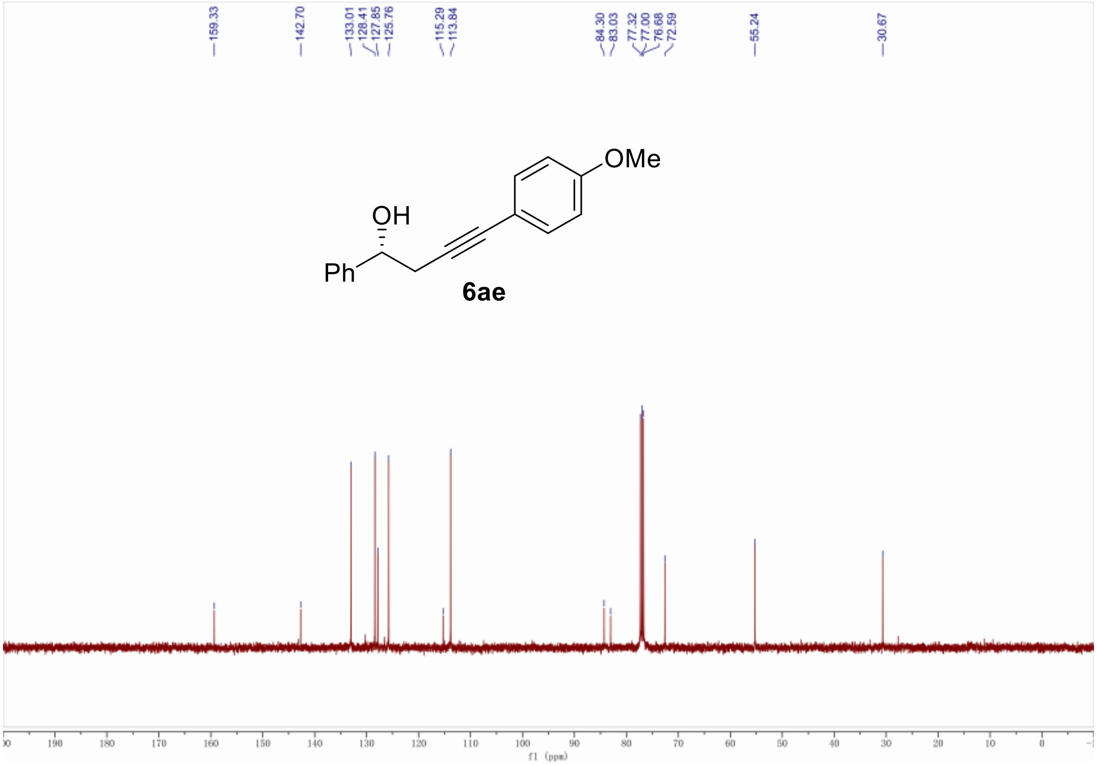

（^{1}H NMR, 400 MHz, $CDCl_3$）

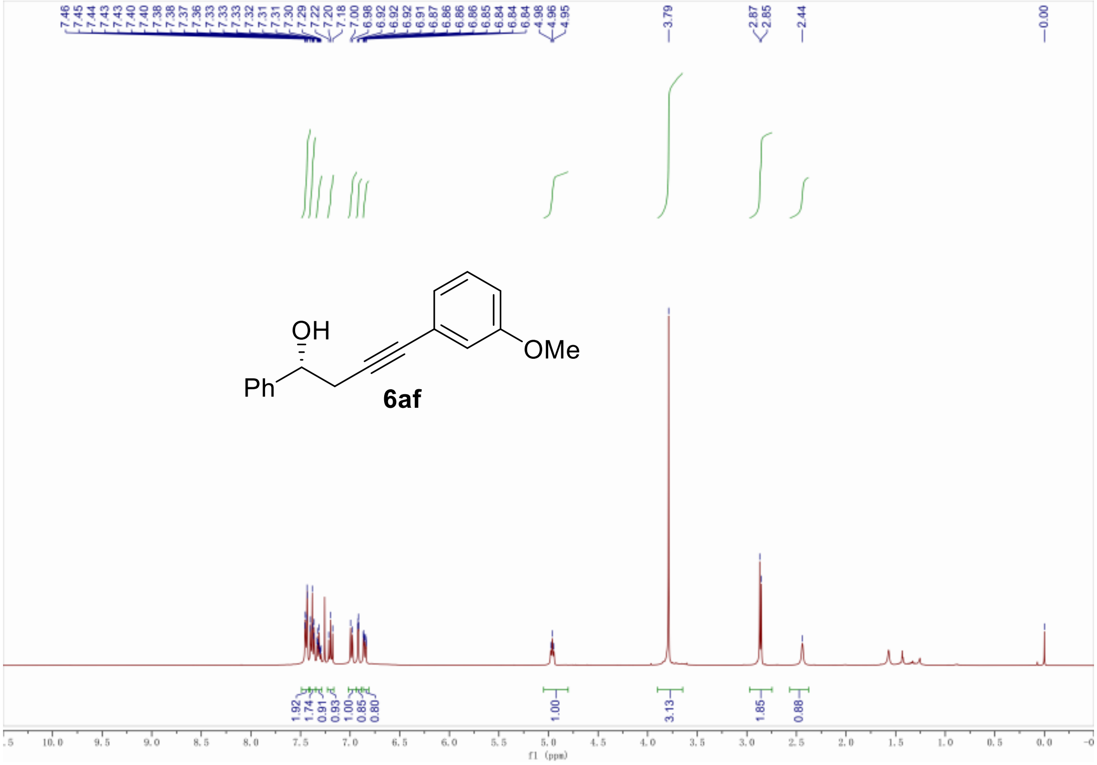

(^{13}C NMR, 100 MHz, $CDCl_3$)

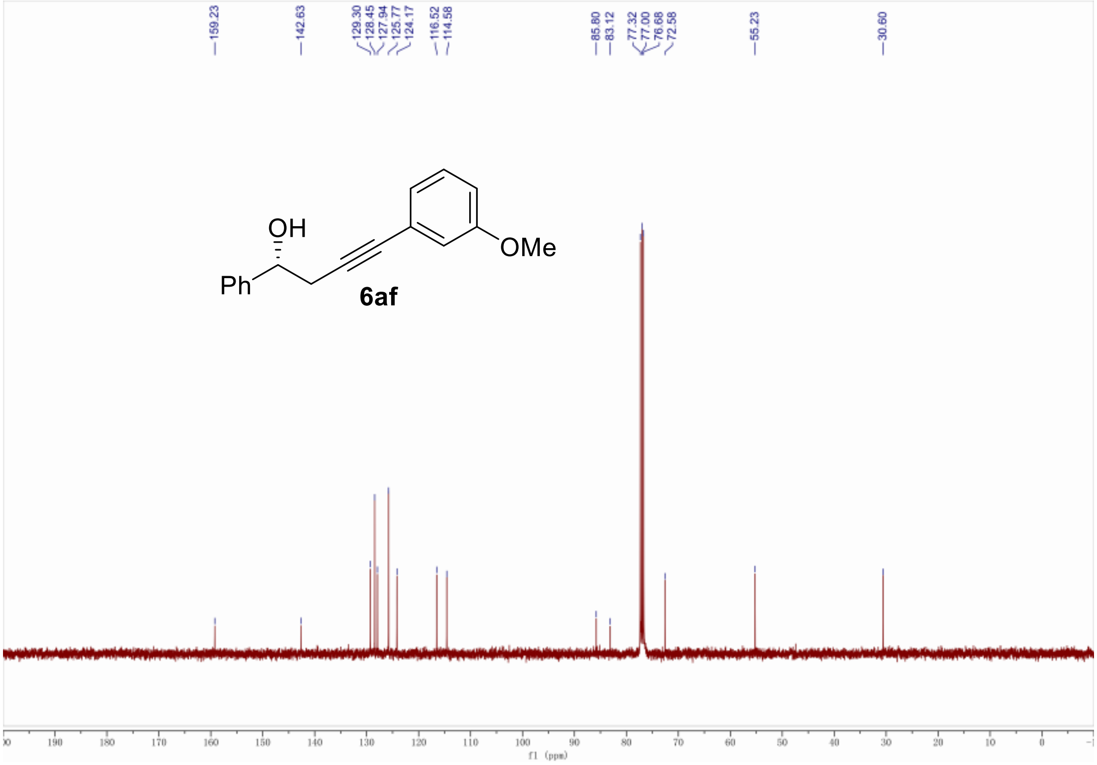

(1H NMR, 400 MHz, CDCl3)

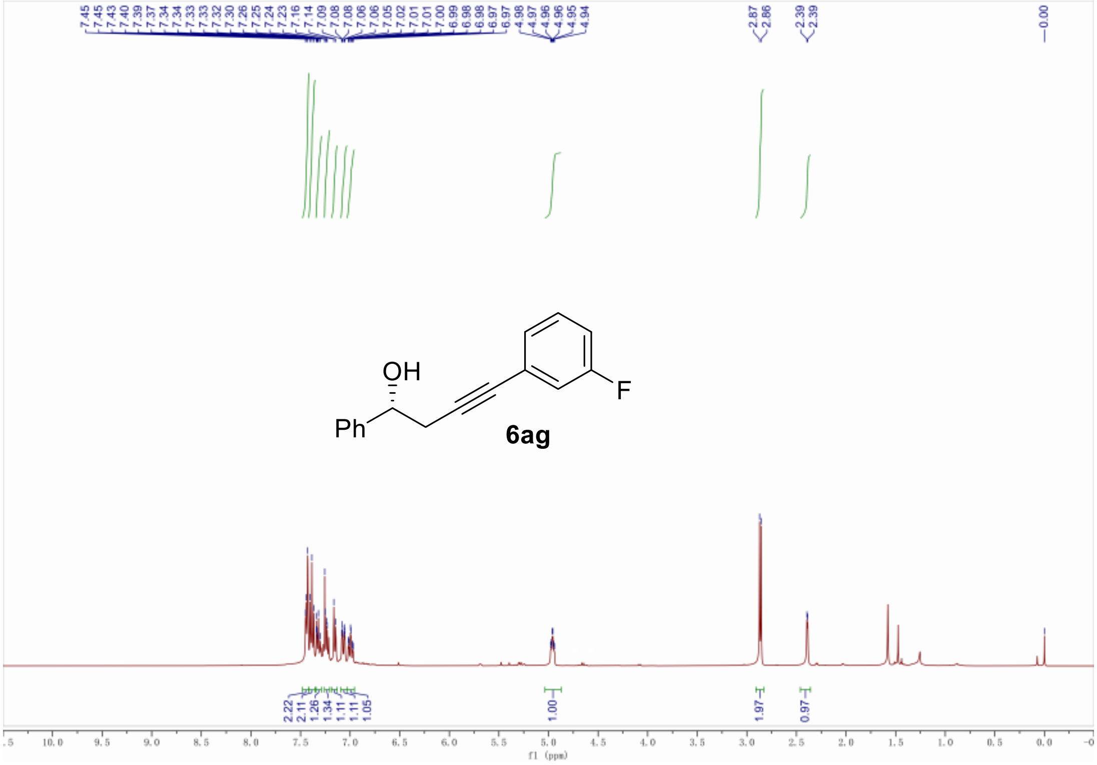

(13C NMR, 100 MHz, CDCl3)

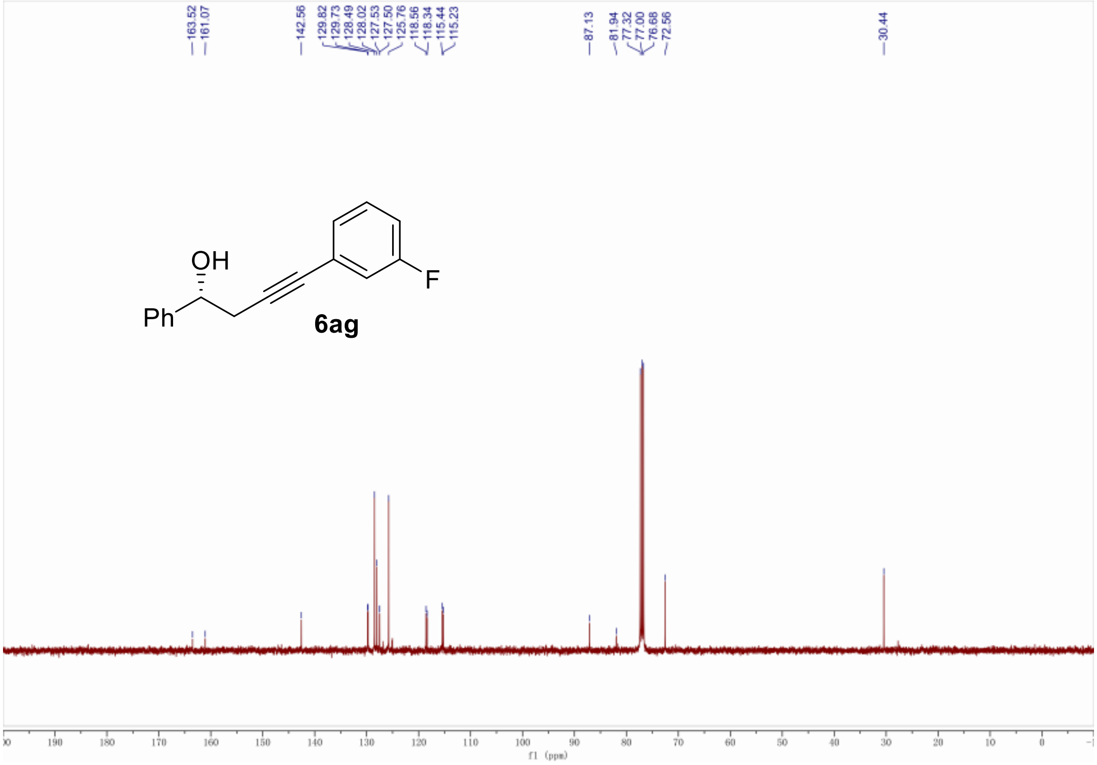

(19F NMR, 376 MHz, CDCl3)

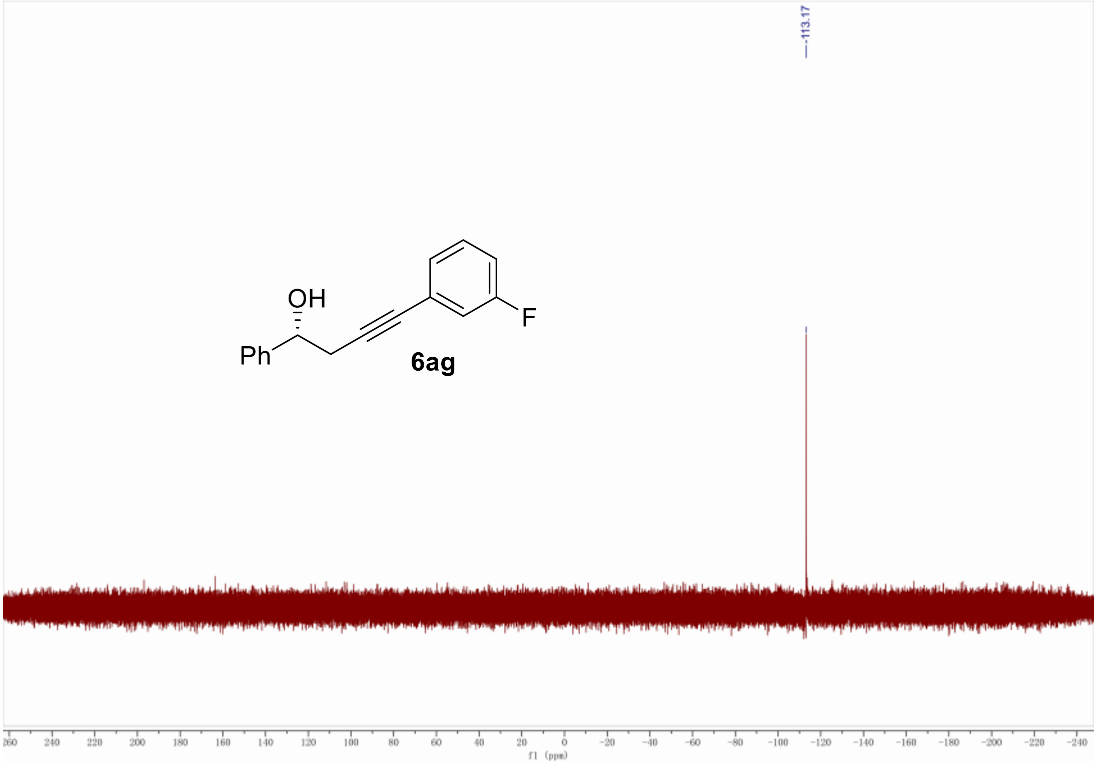

6ag

(1H NMR, 400 MHz, CDCl3)

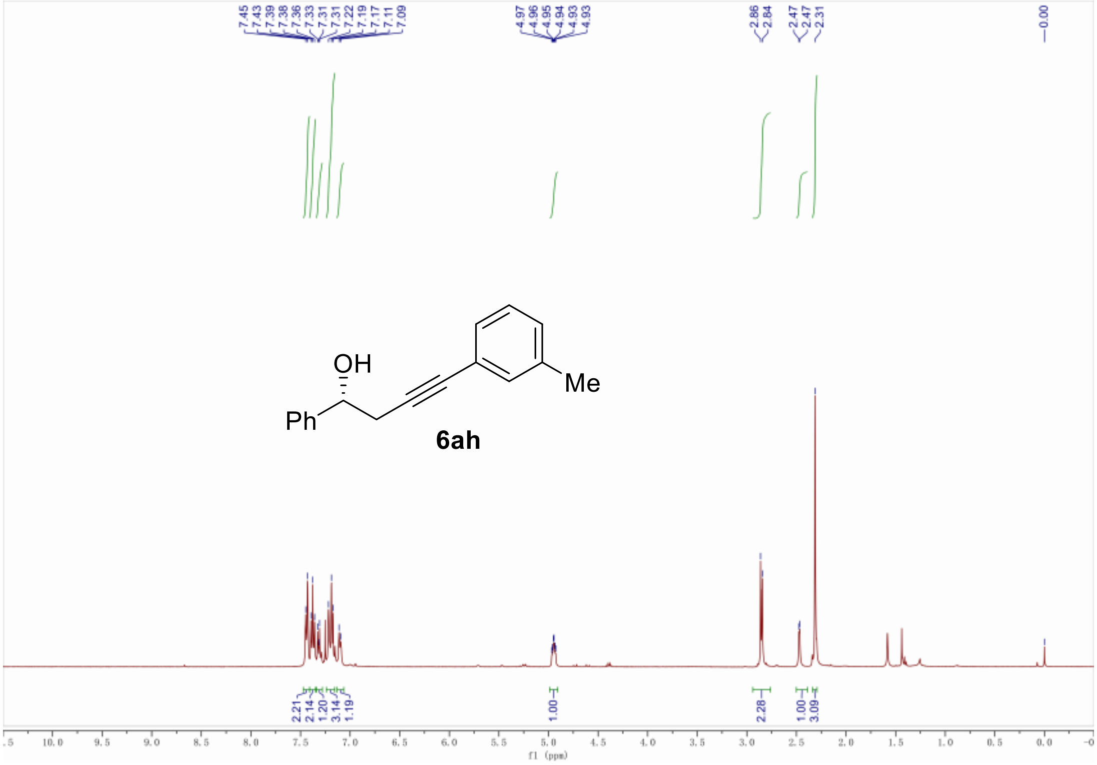

6ah

(^{13}C NMR, 100 MHz, CDCl$_3$)

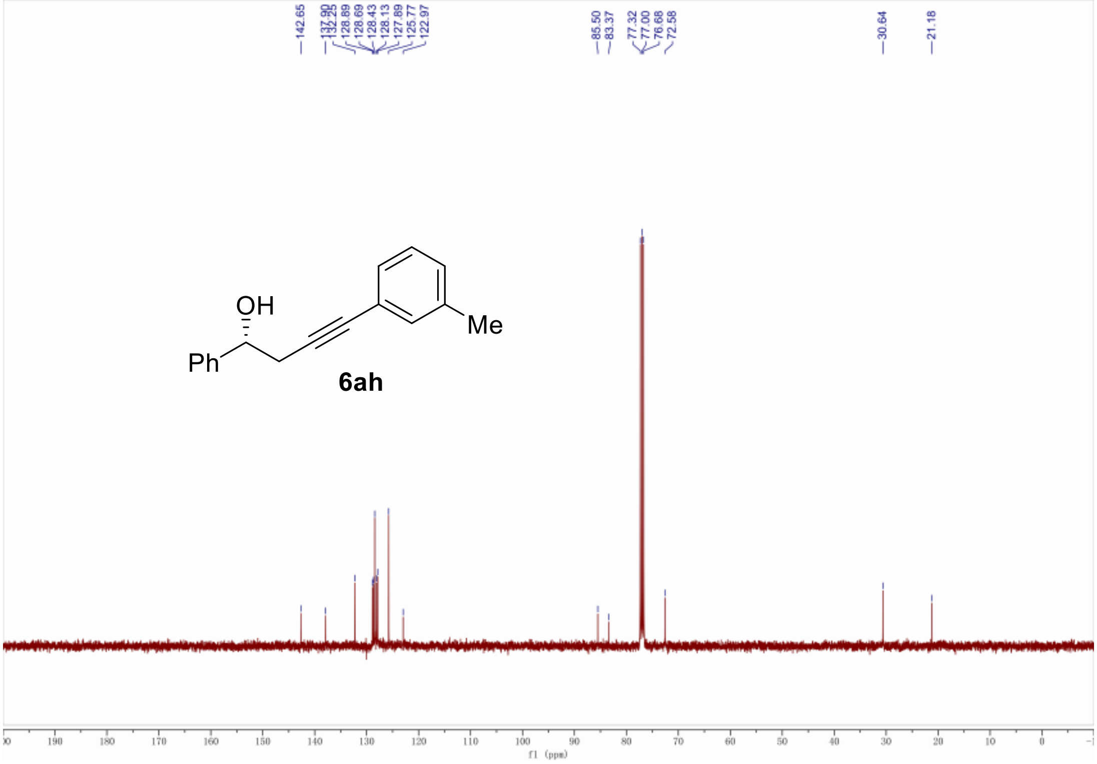

(^{1}H NMR, 400 MHz, CDCl$_3$)

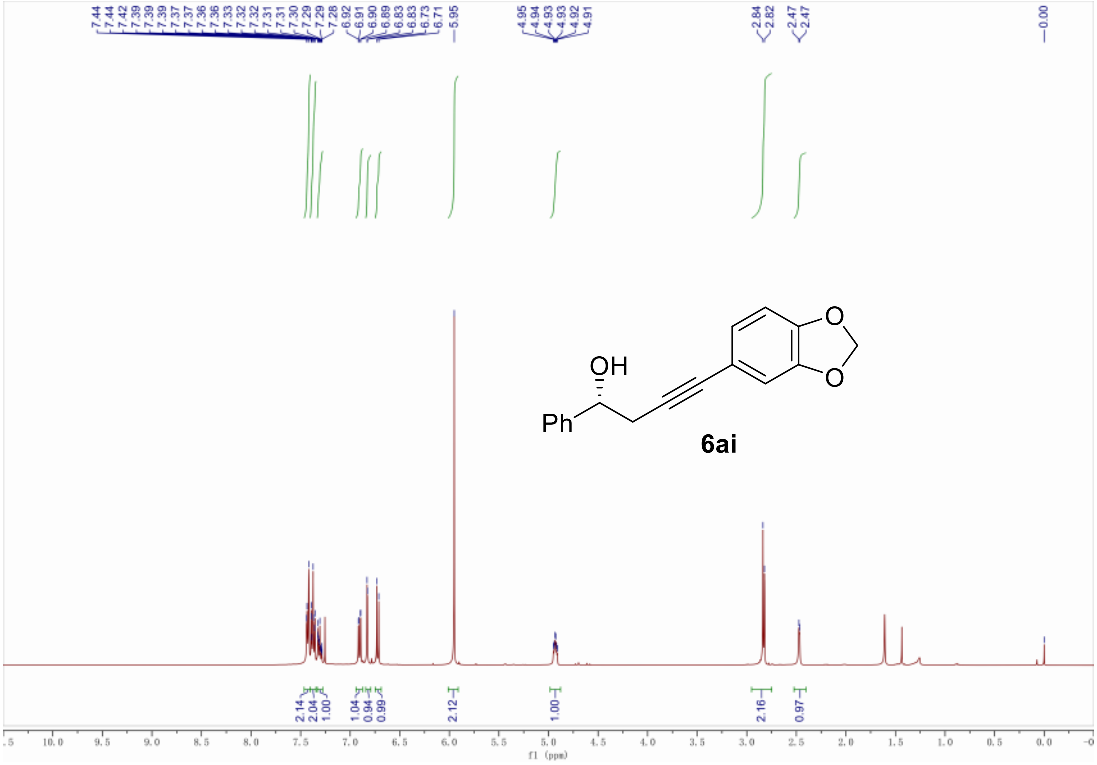

(^{13}C NMR, 100 MHz, CDCl$_3$)

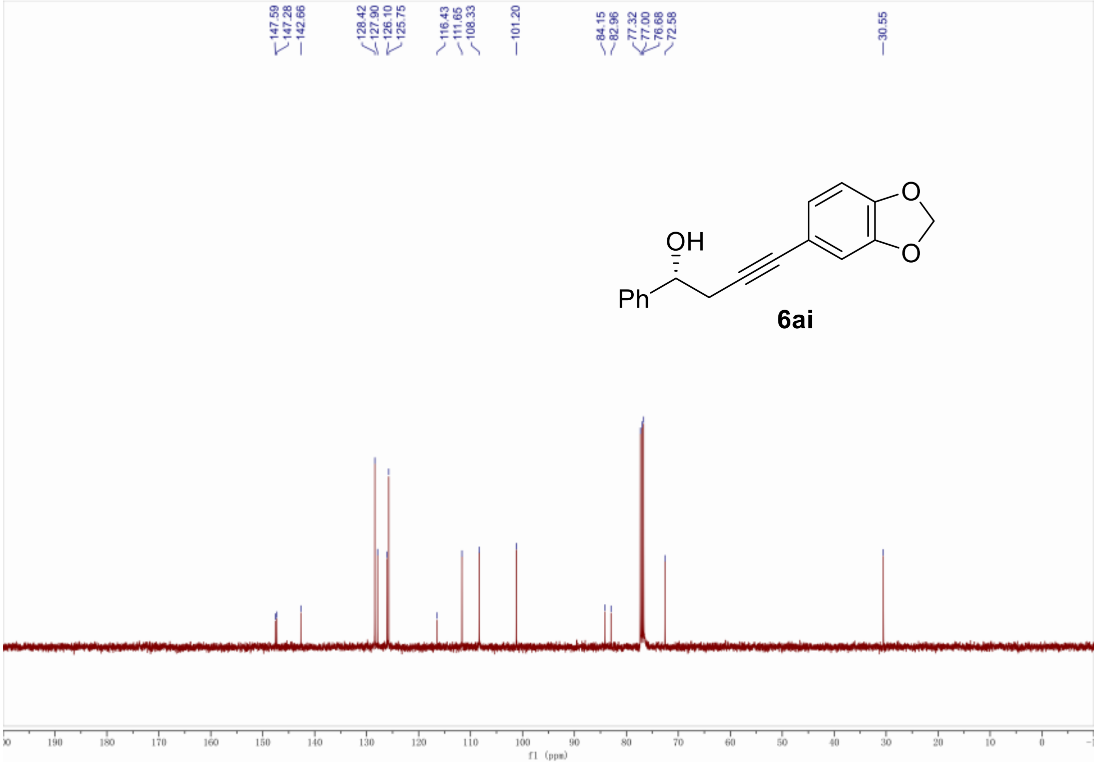

(^{1}H NMR, 400 MHz, CDCl$_3$)

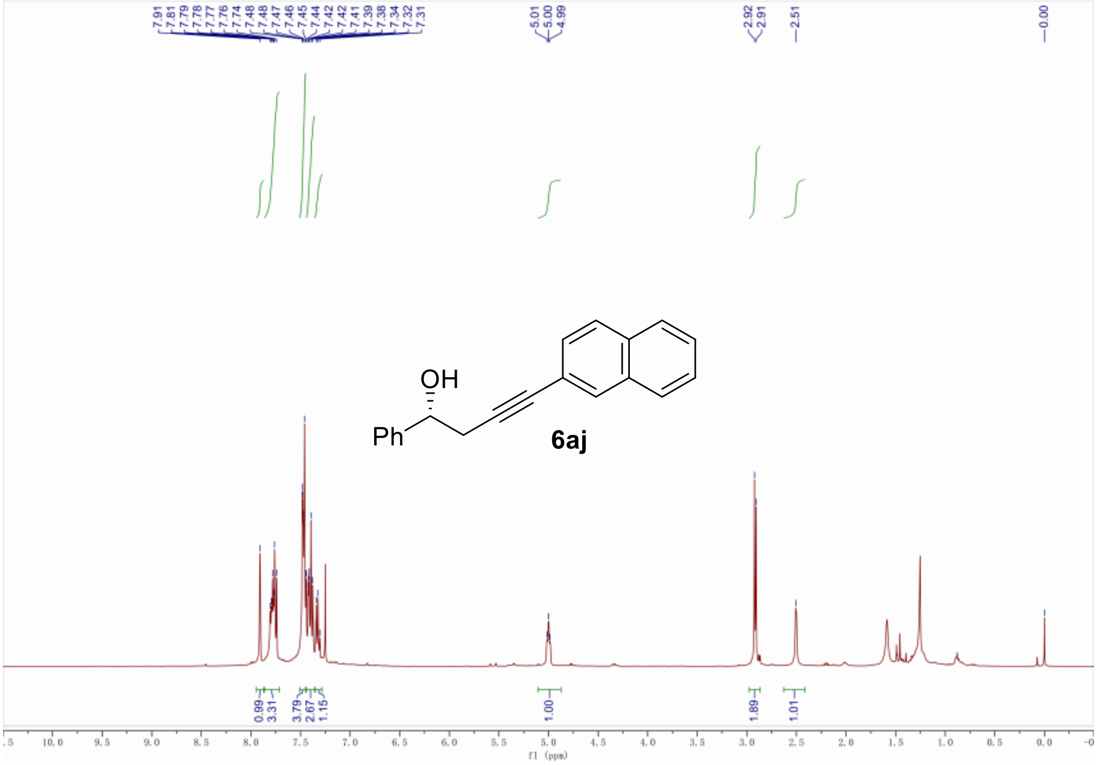

(^{13}C NMR, 100 MHz, $CDCl_3$)

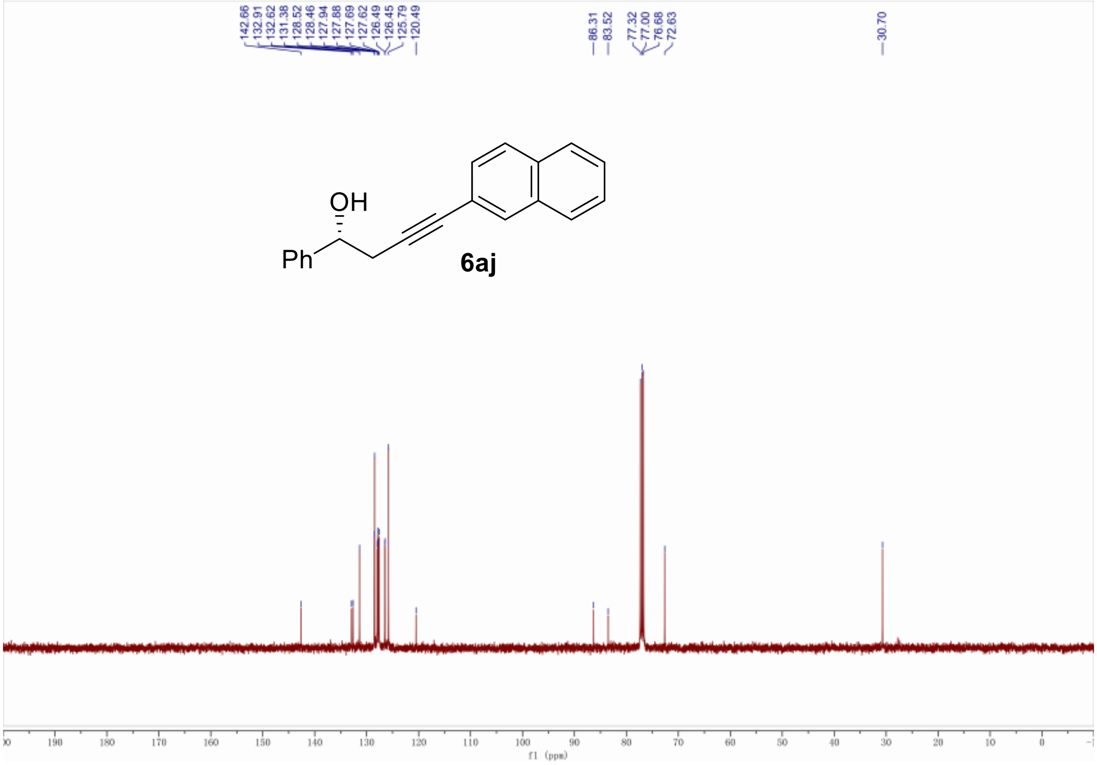

(^{1}H NMR, 400 MHz, $CDCl_3$)

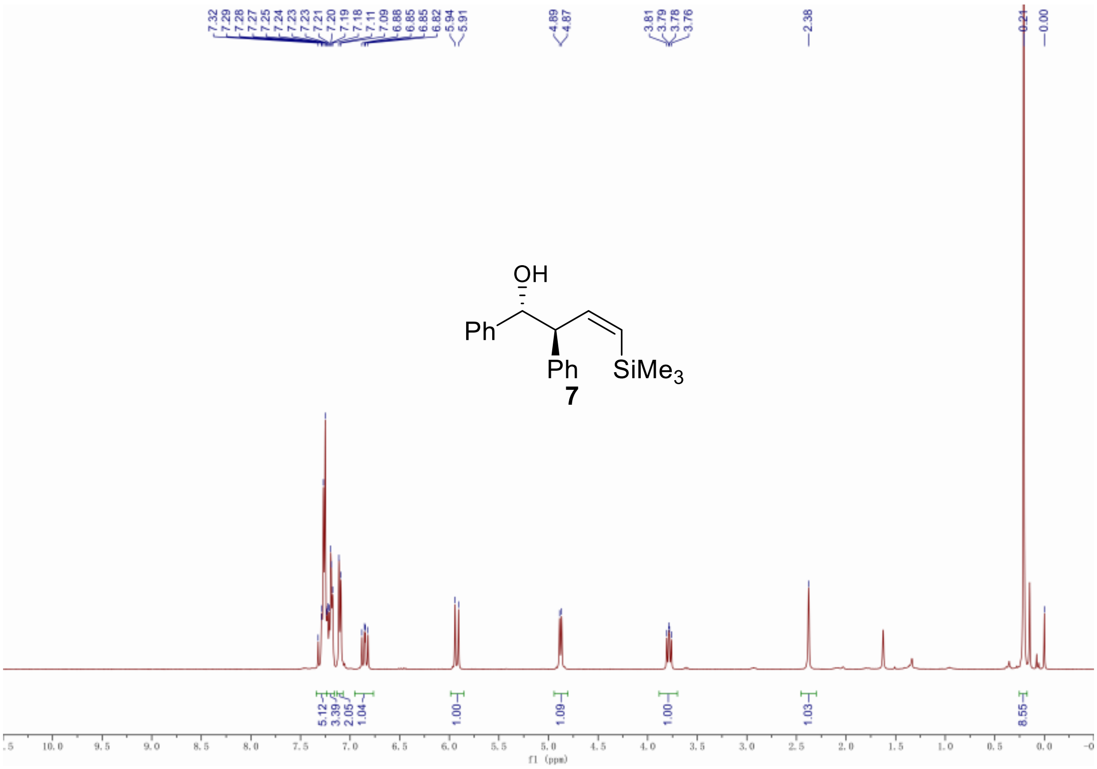

(^{13}C NMR, 100 MHz, CDCl$_3$)

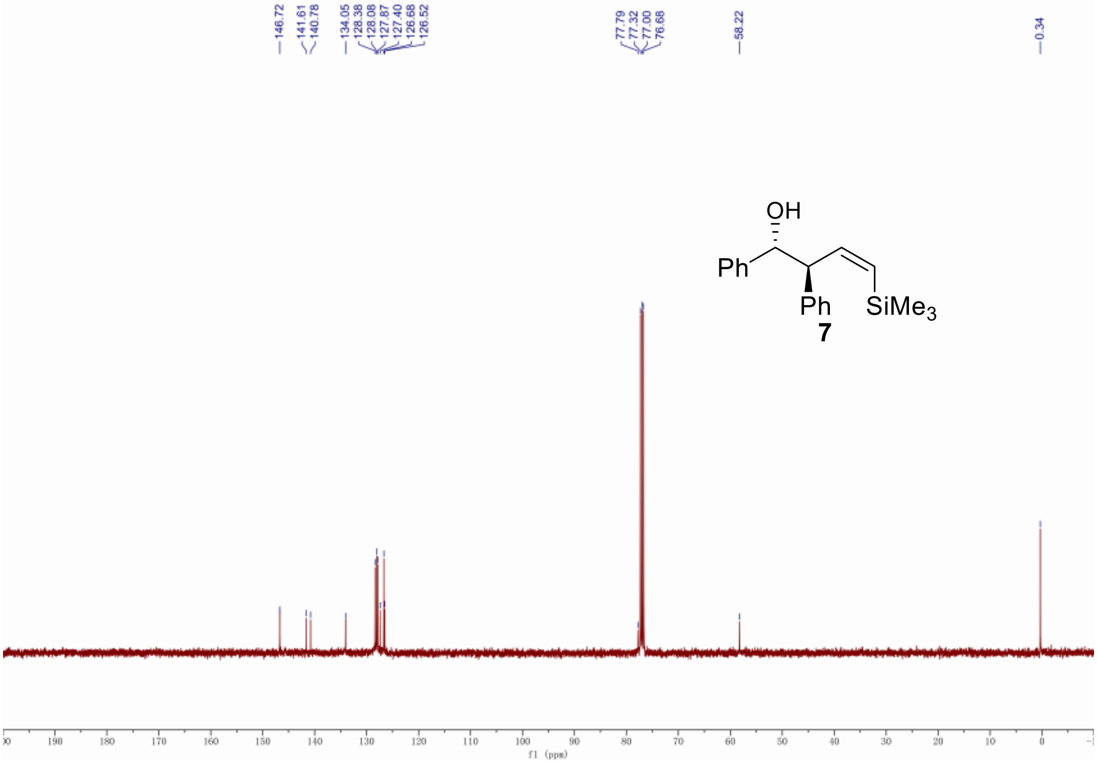

(NOESY, 600 MHz, $CDCl_3$)

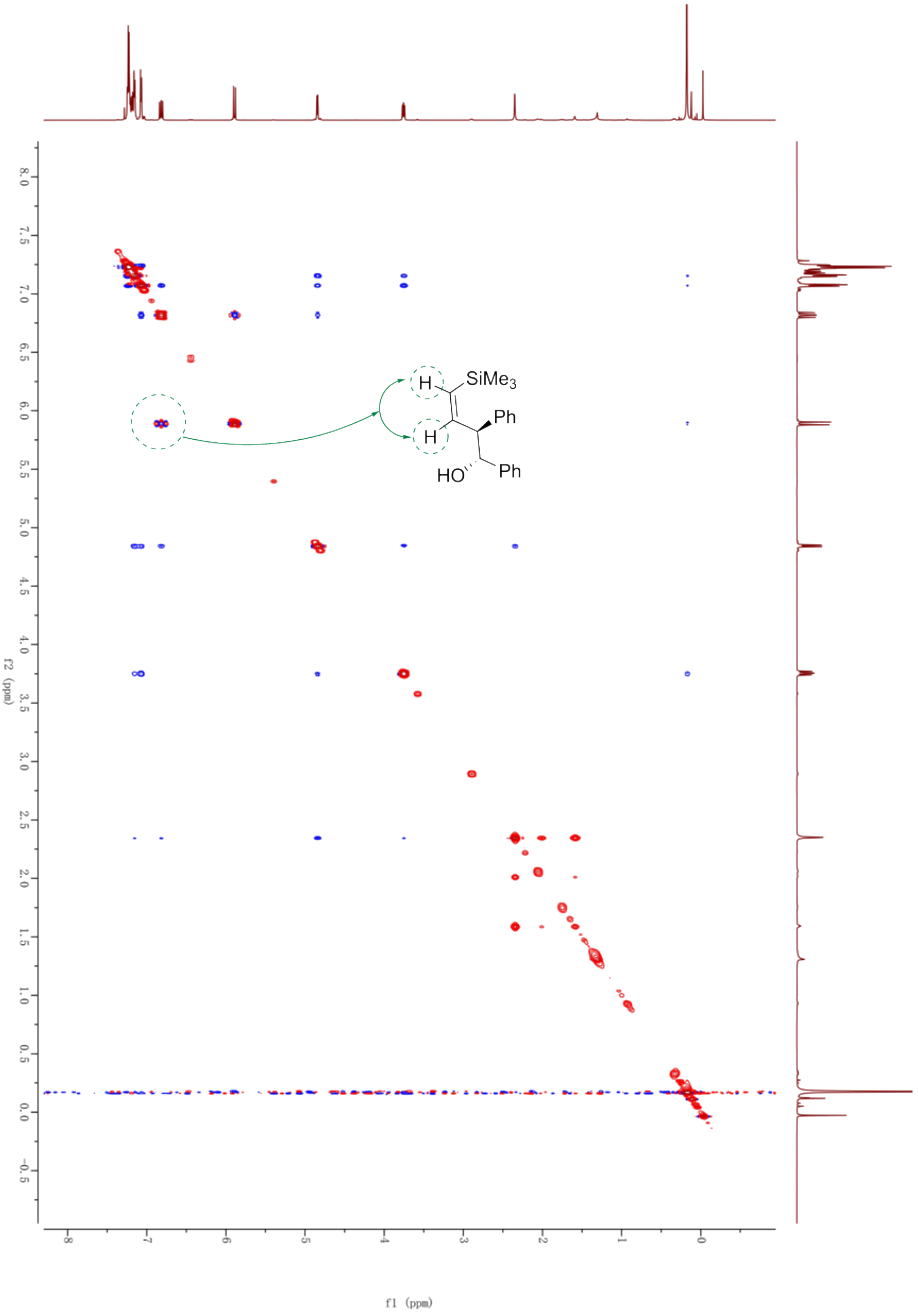

（^{1}H NMR, 400 MHz, $CDCl_3$）

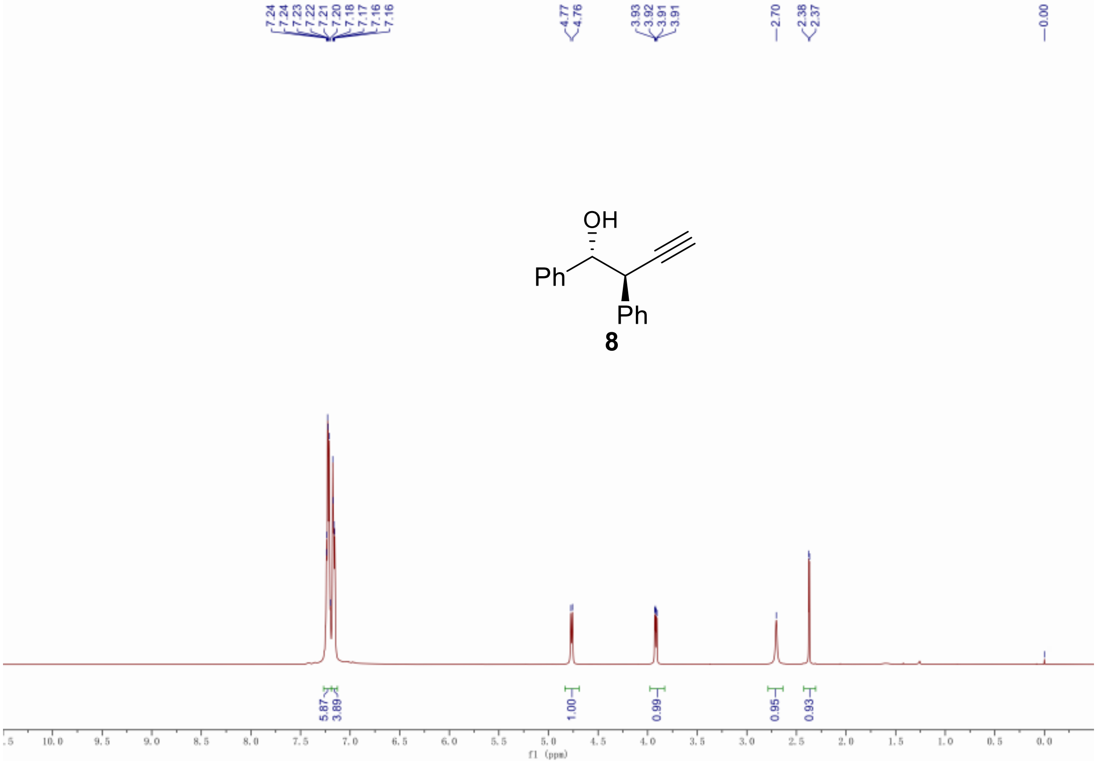

(^{13}C NMR, 100 MHz, $CDCl_3$)

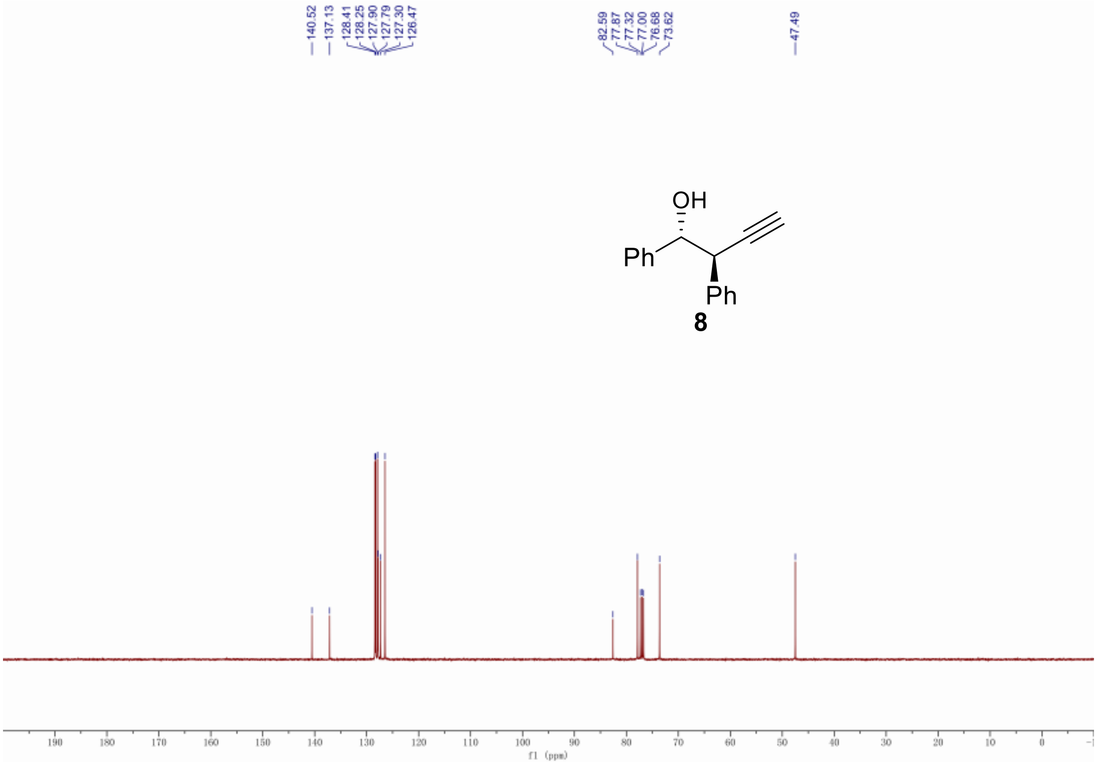

(¹H NMR, 400 MHz, CDCl₃)

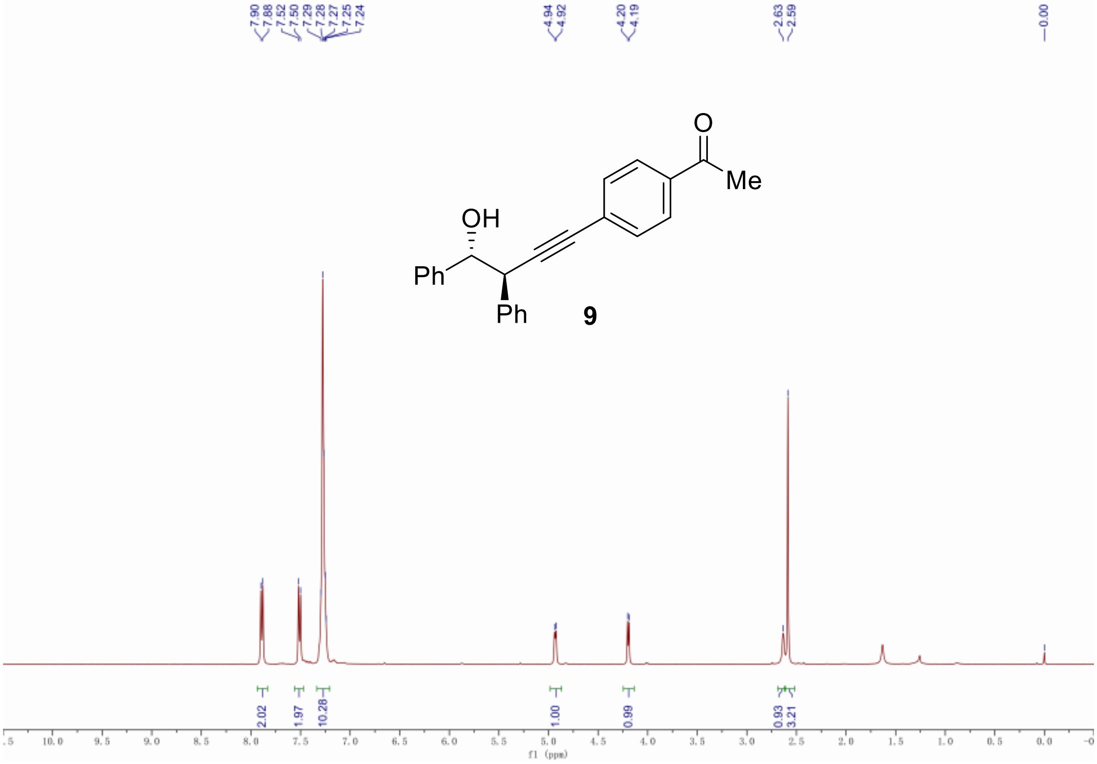

(¹³C NMR, 100 MHz, CDCl₃)

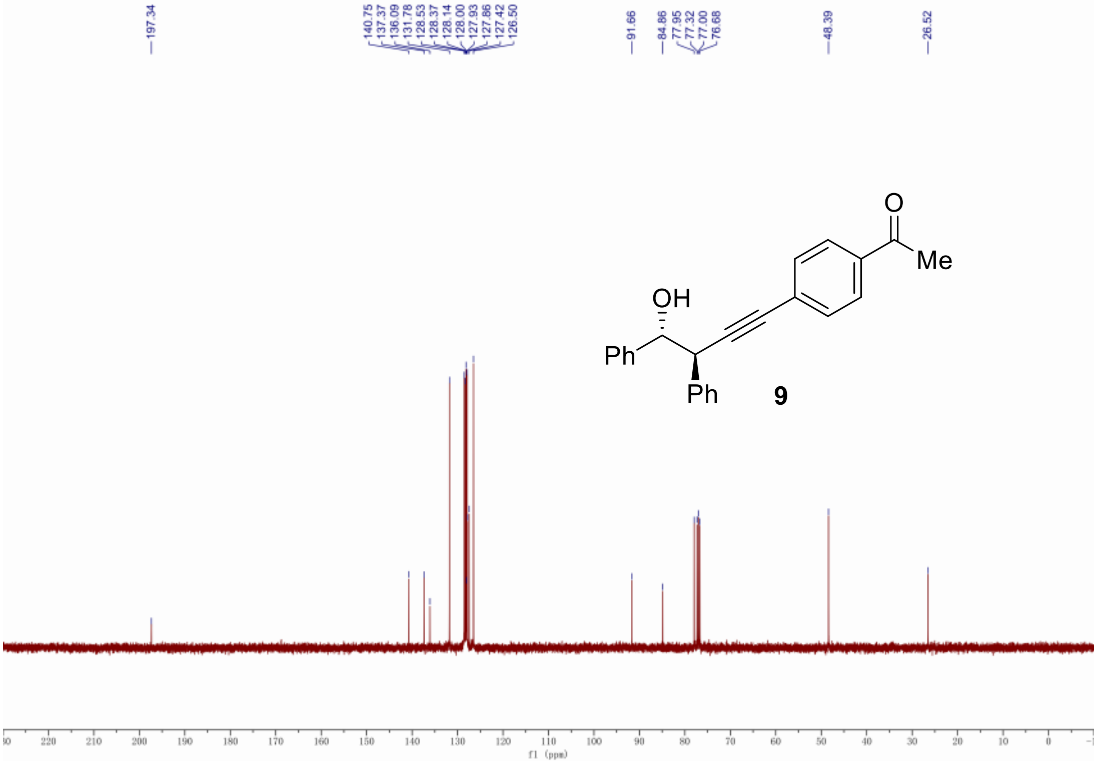

(**^{1}H NMR, 400 MHz, CDCl$_3$**)

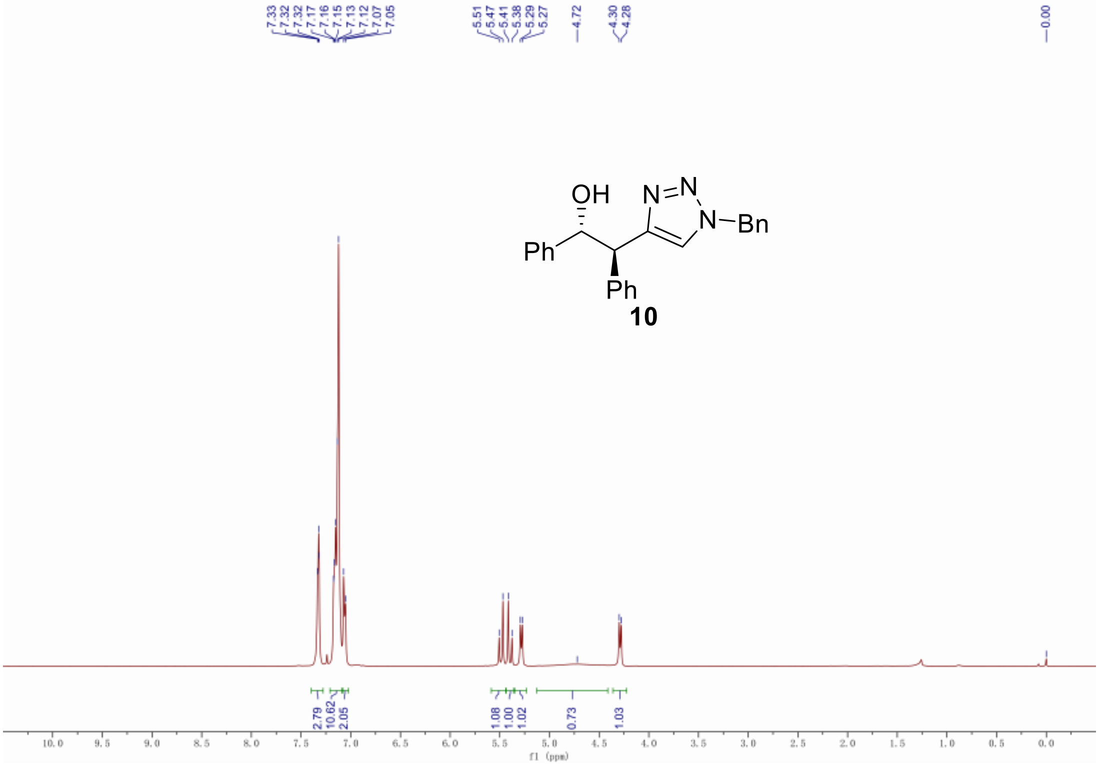

(**^{13}C NMR, 100 MHz, CDCl$_3$**)

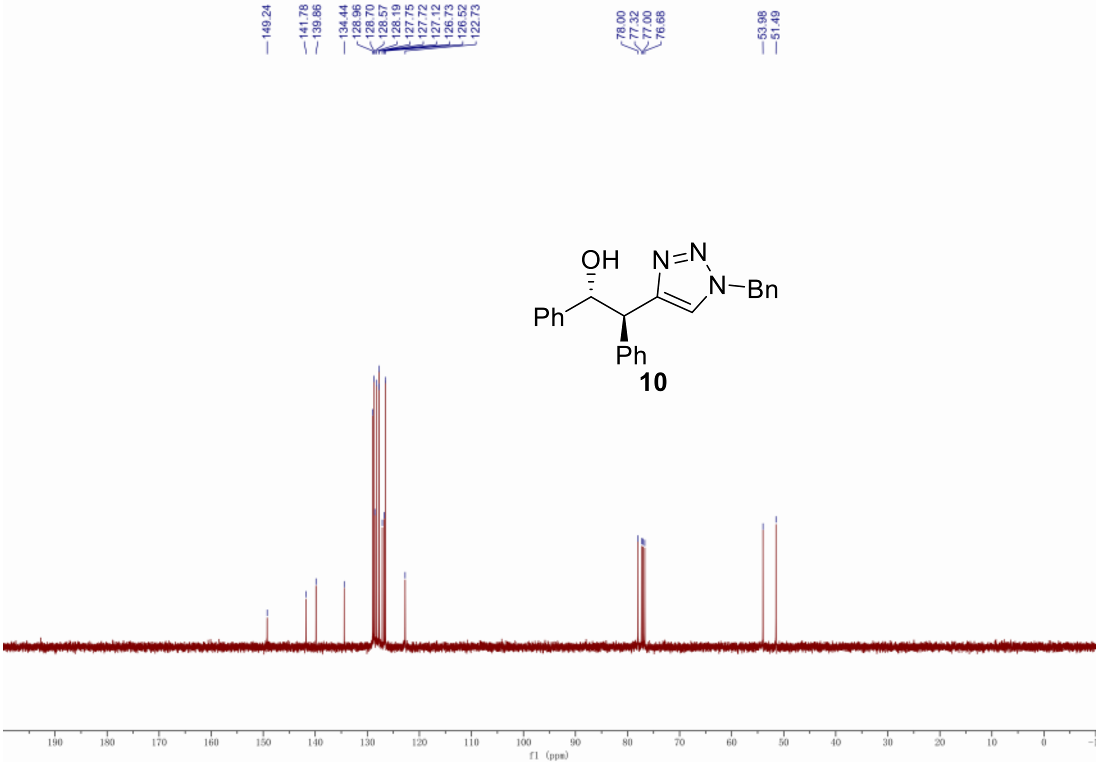

(¹H NMR, 400 MHz, CDCl₃)

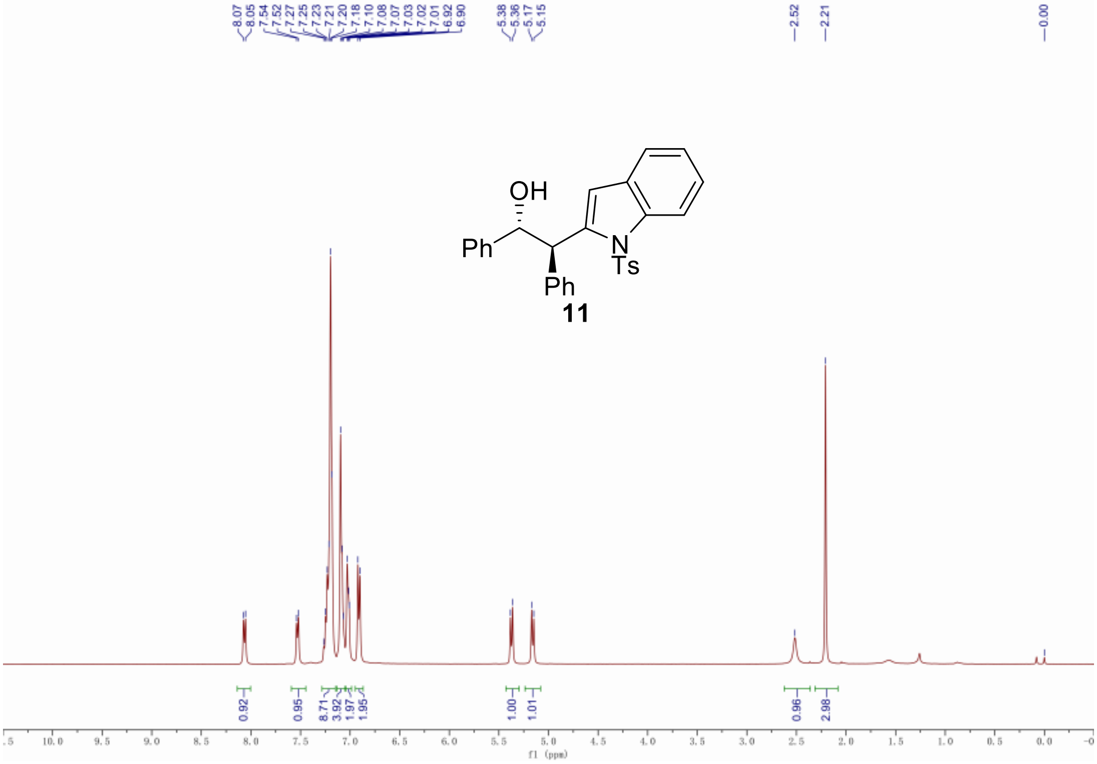

(¹³C NMR, 100 MHz, CDCl₃)

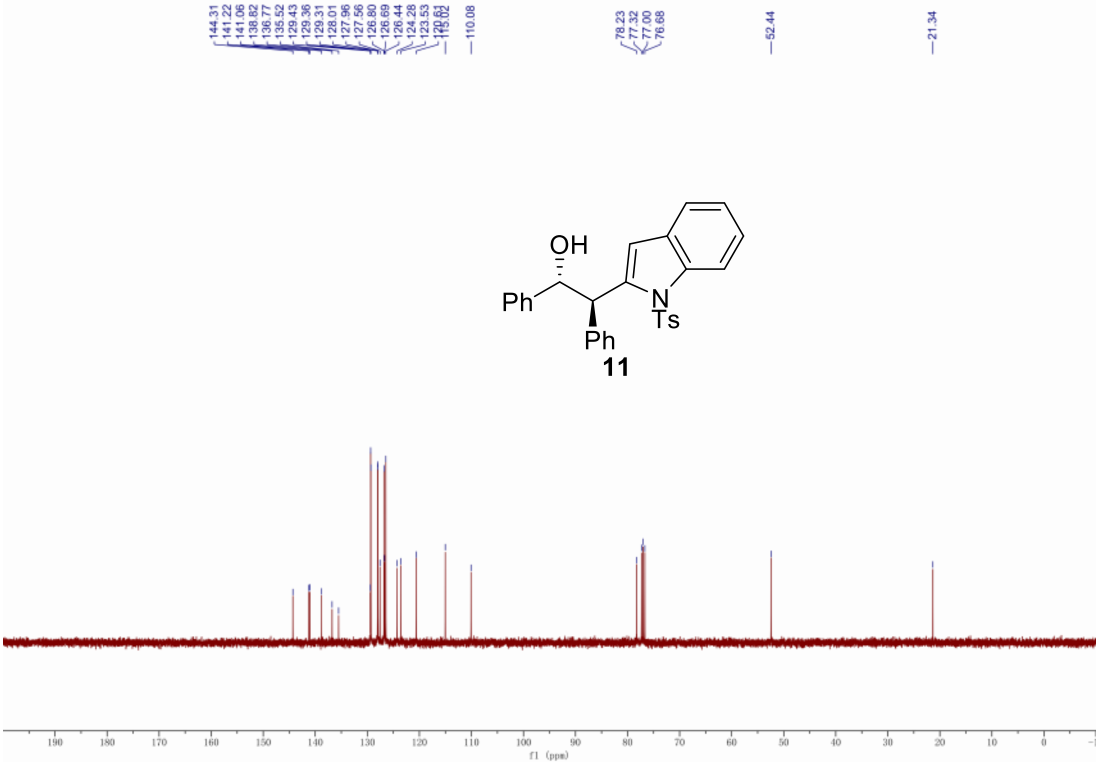

(¹H NMR, 400 MHz, CDCl₃)

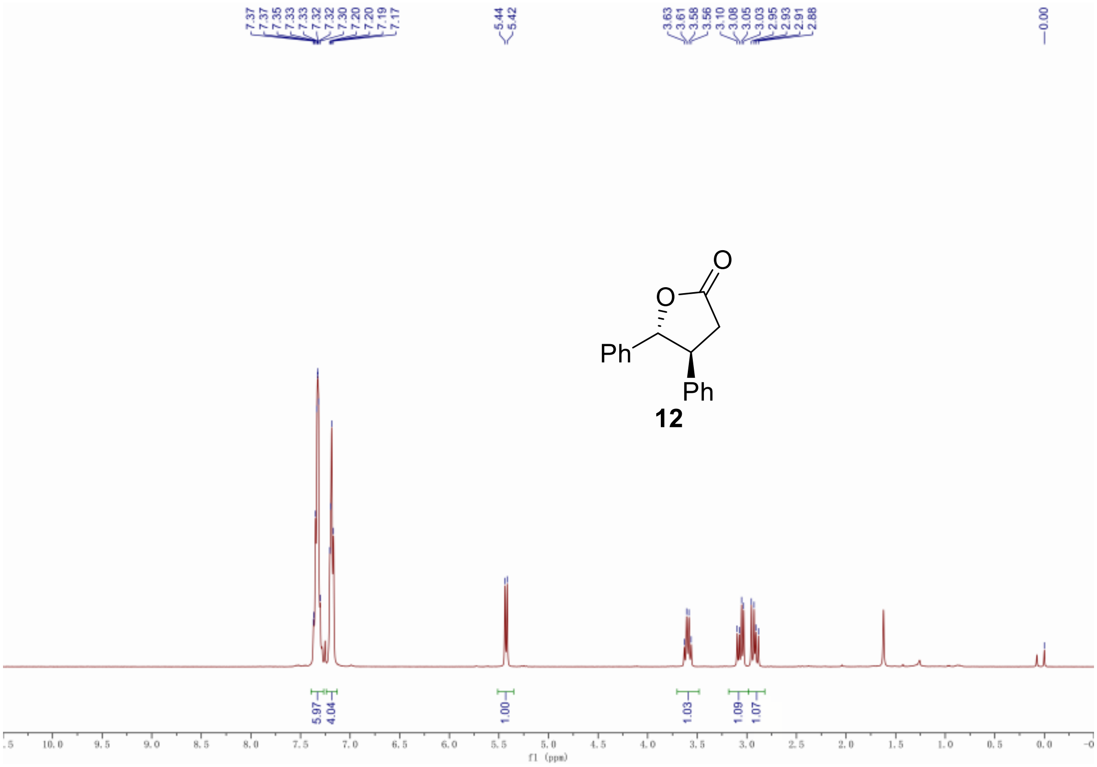

(¹³C NMR, 100 MHz, CDCl₃)

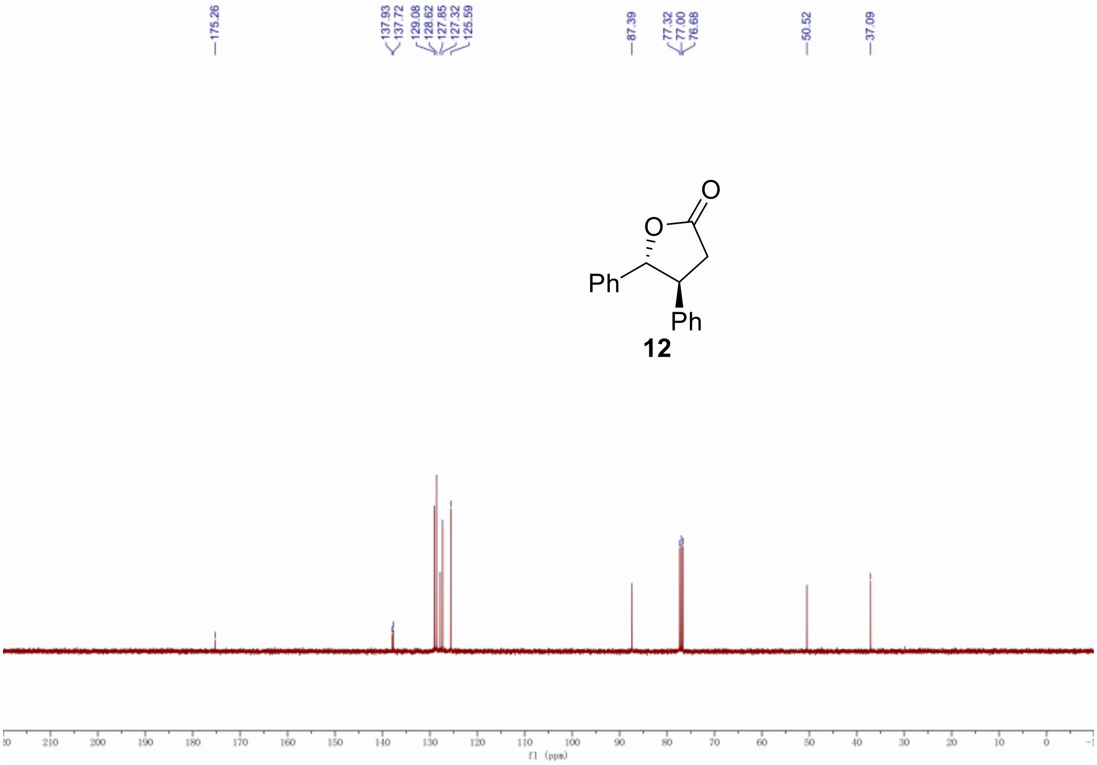

(¹H NMR, 400 MHz, $CDCl_3$)

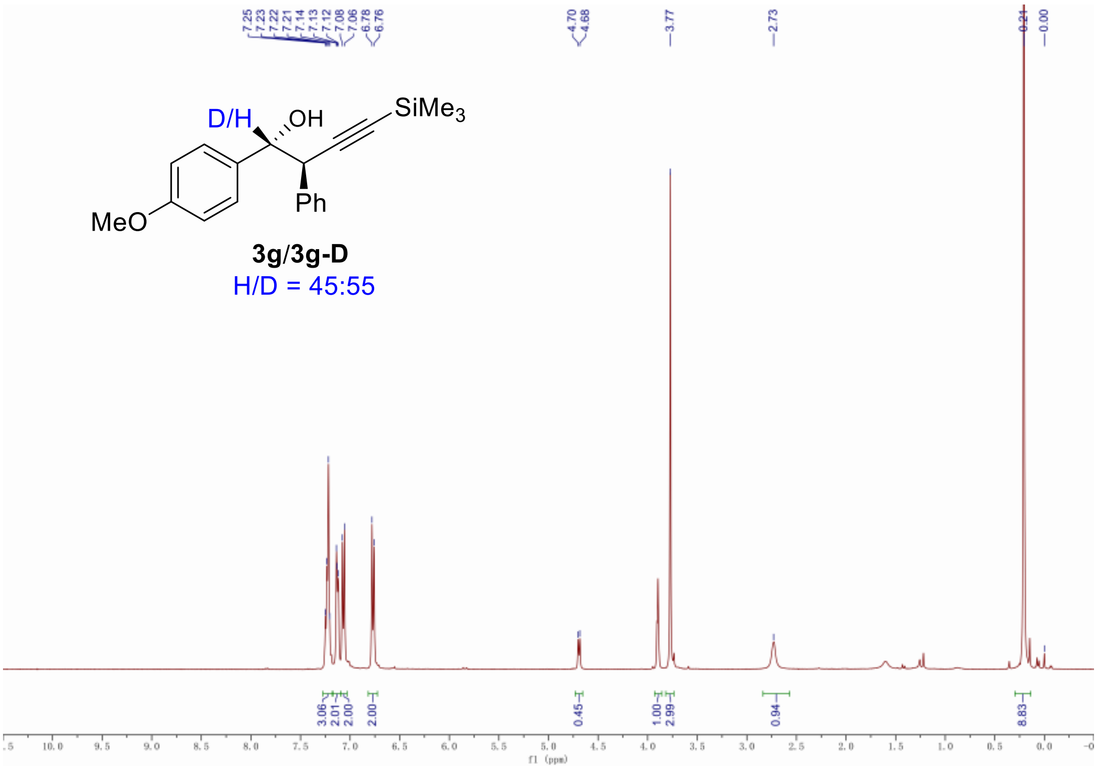

(¹H NMR, 400 MHz, $CDCl_3$)

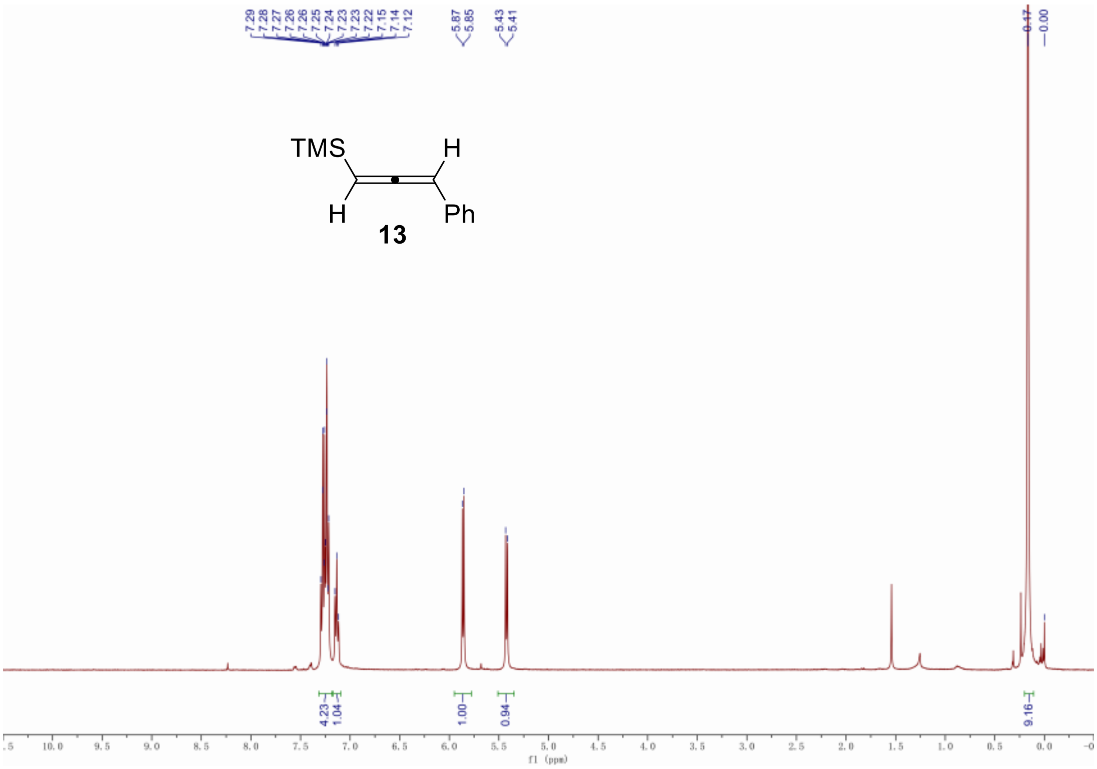

(^{13}C NMR, 100 MHz, $CDCl_3$)

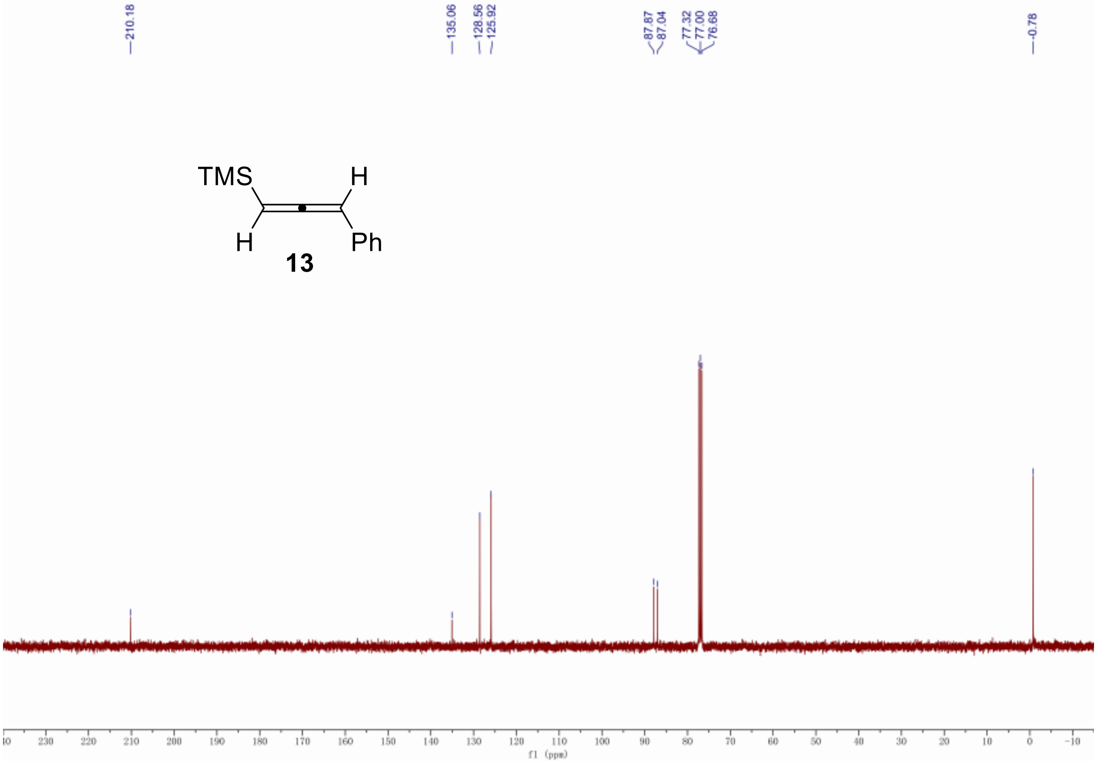

(^{1}H NMR, 400 MHz, $CDCl_3$)

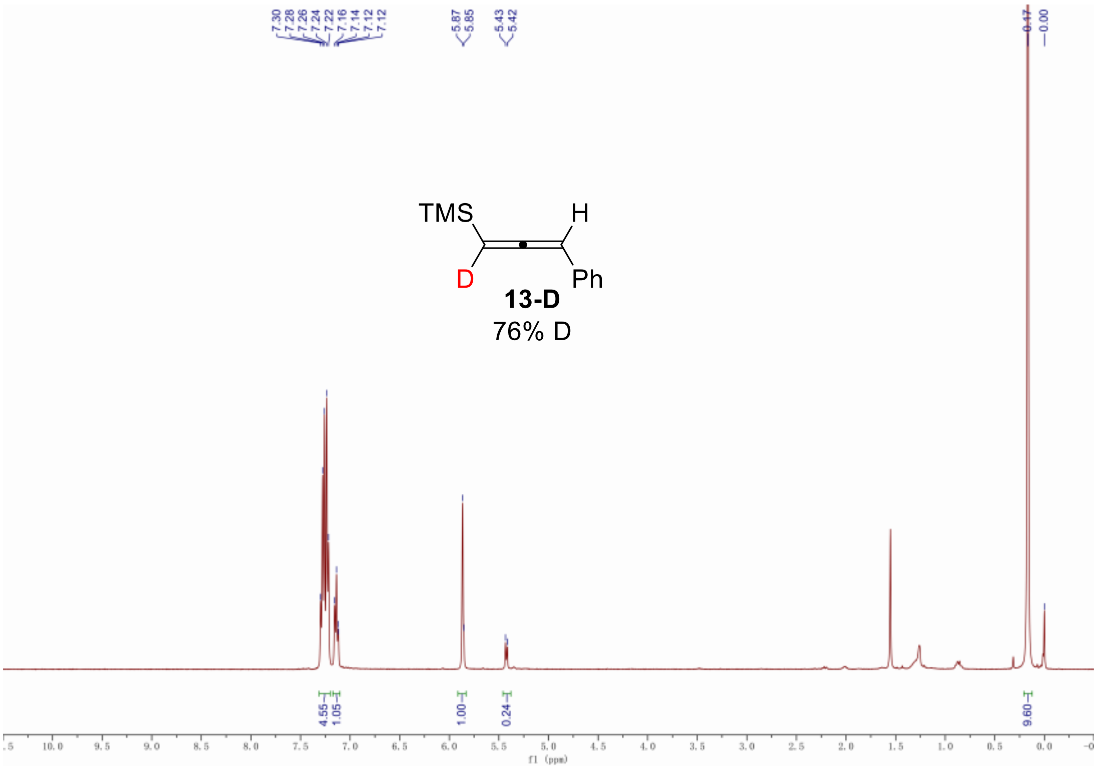

(^{2}H NMR, 61 MHz, CHCl$_3$)

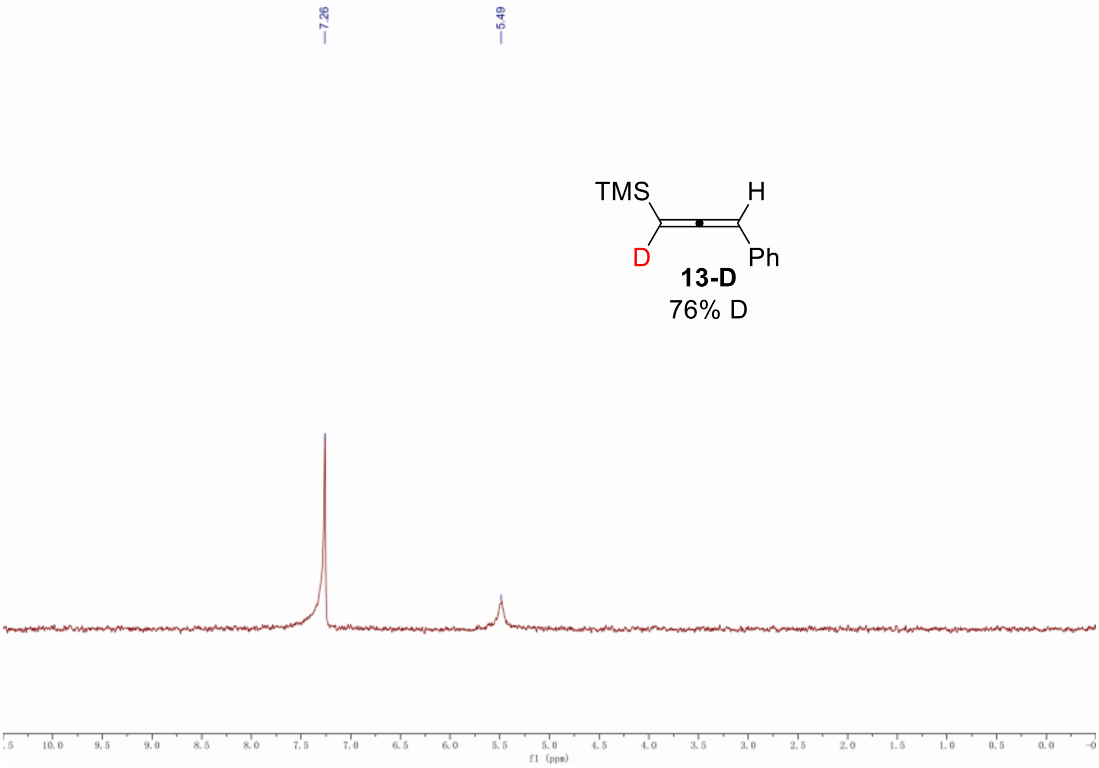

■ Supplementary Reference:

1. Miyazaki, Y., Zhou, B., Tsuji, H., and Kawatsura, M. Nickel-catalyzed asymmetric Friedel-Crafts propargylation of 3-substituted indoles with propargylic carbonates bearing an internal alkyne group. *Org. Lett.* **2020**, *22*, 2049-2053.

2. Nguyen, K.D., Herkommer, D., and Krische, M. J. Ruthenium-BINAP cataly zed alcohol C–H tert-prenylation via 1,3-enyne transfer hydrogenation: Beyond stoichiometric carbanions in enantioselective carbonyl propargylation. *J. Am. Ch em. Soc.* **2016**, *138*, 5238–5241.

3. Fandrick, D. R., Fandrick, K. R., Reeves, J. T., Tan, Z., Tang, W., Capacci, A. G., Rodriguez, S., Song, J .J., Lee, H., Yee, N. K., and Senanayake, C. H. Copper catalyzed asymmetric propargylation of aldehydes. *J. Am. Chem. So c.* **2010**, *132*, 7600–7601.

4. Inoue, M., and Nakada, M. Studies into asymmetric catalysis of the Nozaki-Hiyama allenylation. *Angew. Chem. Int. Ed.* **2006**, *45*, 252-255.

5. Chen, W., Tay, J. H., Ying, J., Yu, X. Q., and Pu, L. Catalytic asymmetric enyne addition to aldehydes and Rh(I)-catalyzed stereoselective domino Pauson -Khand/[4+2] cycloaddition. *J. Org. Chem.* **2013**, *78*, 2256-2265.

6. Frisch, M. J., Trucks, G. W., Schlegel, H. B., Scuseria, G.E ., Robb, M. A., Cheeseman, J. R., Scalmani, G., Barone, V., Petersson, G. A., Nakatsuji, H., Li, X., Caricato, M., Marenich, A. V., Bloino, J., Janesko, B. G., Gomperts, R., Mennucci, B., Hratchian, H. P., Ortiz, J. V., Izmaylov, A. F., Sonnenberg, J. L., Williams, F., Ding, F., Lipparini, F., Egidi, J., Goings, B., Peng, A., Pet rone, T., Henderson, D., Ranasinghe, V. G., Zakrzewski, J., Gao, N., Rega, G., Zheng, W., Liang, M., Hada, M., Ehara, K., Toyota, R., Fukuda, J., Hasegaw a, M., Ishida, T., Nakajima, Y., Honda, O., Kitao, H., Nakai, T., Vreven, K., Throssell, J.A., Montgomery Jr, J.E., Peralta, F., Ogliaro, M., Bearpark, J., Hey d, J. J., Brothers, E. N., Kudin, K. N., Staroverov, V. N., Keith, T. A., Kobay ashi, R., Normand, J., Raghavachari, K., Rendell, A. P., Burant, J. C., Iyengar, S. S., Tomasi, J., Cossi, M., Millam, J. M., Klene, M., Adamo, C., Cammi, R., Ochterski, J. W., Martin, R. L., Morokuma, K., Farkas, O., and Foresman, J. B. D. J. Fox *Gaussian 16 Rev. A.03*, Wallingford, CT (2016)..

7. Becke, A. D. Density - functional thermochemistry. III. The role of exact exchange. *J. Chem. Phys.* **1993**, *98*, 5648-5652.

8. Grimme, S., Antony, J., Ehrlich, S., and Krieg, H. A consistent and accurate ab initio parametrization of density functional dispersion correction (DFT-D) for the 94 elements H-Pu. *J. Chem. Phys.* **2010**, *132*, 154104.

9. Weigend, F., and Ahlrichs, R. Balanced basis sets of split valence, triple zeta valence and quadruple zeta valence quality for H to Rn: Design and assessment of accuracy. *Phys. Chem. Chem. Phys.* **2005**, *7*, 3297-3305.

10. Zhao, Y., and Truhlar, D. G. The M06 suite of density functionals for main group thermochemistry, thermochemical kinetics, noncovalent interactions, excited states, and transition elements: two new functionals and systematic testing of four M06-class functionals and 12 other functionals. *Theor. Chem. Acc.* **2008**, *120*, 215-241.

11. Fukui, K. The path of chemical reactions-the IRC approach. *Acc. Chem. Res.* 1981, *14*, 363-368.
12. Marenich, A. V., Cramer, C. J., and Truhlar, D. G. Universal solvation model based on solute electron density and on a continuum model of the solvent defined by the bulk dielectric constant and atomic surface tensions. *J. Phys. Chem. B.* **2009**, *113*, 6378-6396.
