# Supplementary material for: Photoredox cobalt-catalyzed regio-, diastereo- and enantioselective propargylation of aldehydes via propargyl radicals
Source: Nat Commun. 2023 Aug 10;14:4825. doi: 10.1038/s41467-023-40488-3 (PMC10415309; doi:10.1038/s41467-023-40488-3)

checkCIF/PLATON report

Structure factors have been supplied for datablock(s) t_a

THIS REPORT IS FOR GUIDANCE ONLY. IF USED AS PART OF A REVIEW PROCEDURE FOR PUBLICATION, IT SHOULD NOT REPLACE THE EXPERTISE OF AN EXPERIENCED CRYSTALLOGRAPHIC REFEREE.

No syntax errors found. CIF dictionary Interpreting this report

Datablock: t_a

Bond precision: C-C = 0.0051 A Wavelength=1.54178

Cell:	a=10.2881(7)	b=5.6047(4)	c=17.1021(12)
	alpha=90	beta=103.621(4)	gamma=90
Temperature:	223 K		

	Calculated	Reported
Volume	958.40(12)	958.40(12)
Space group	P 21	P 21
Hall group	P 2yb	P 2yb
Moiety formula	C21 H24 O2 Si	C21 H24 O2 Si
Sum formula	C21 H24 O2 Si	C21 H24 O2 Si
Mr	336.49	336.49
Dx,g cm-3	1.166	1.166
Z	2	2
Mu (mm-1)	1.144	1.144
F000	360.0	360.0
F000’	361.42	
h,k,lmax	12,6,20	12,6,20
Nref	3389[1886]	3303
Tmin,Tmax	0.814,0.852	0.722,0.752
Tmin’	0.814	

Correction method= # Reported T Limits: Tmin=0.722 Tmax=0.752
AbsCorr = MULTI-SCAN

Data completeness= 1.75/0.97 Theta(max)= 66.583

R(reflections)= 0.0449(2517) wR2(reflections)= 0.1111(3303)

S = 1.044 Npar= 222

The following ALERTS were generated. Each ALERT has the format
test-name_ALERT_alert-type_alert-level.
Click on the hyperlinks for more details of the test.

Alert level C

```
PLAT242_ALERT_2_C Low    'MainMol' Ueq as Compared to Neighbors of        Si1 Check
PLAT340_ALERT_3_C Low Bond Precision on  C-C Bonds ...............    0.00505 Ang.
PLAT911_ALERT_3_C Missing FCF Refl Between Thmin & STh/L=    0.595          6 Report
PLAT987_ALERT_1_C The Flack x is >> 0 -  Do a BASF/TWIN Refinement     Please Check
```

Alert level G

```
PLAT007_ALERT_5_G Number of Unrefined Donor-H Atoms ..............          1 Report
PLAT033_ALERT_4_G Flack x Value Deviates > 3.0 * sigma from Zero .      0.090 Note
PLAT767_ALERT_4_G INS Embedded LIST 6 Instruction Should be LIST 4     Please Check
PLAT791_ALERT_4_G Model has Chirality at C9          (Sohnke SpGr)          R Verify
PLAT791_ALERT_4_G Model has Chirality at C10         (Sohnke SpGr)          R Verify
PLAT883_ALERT_1_G No Info/Value for _atom_sites_solution_primary .     Please Do !
PLAT909_ALERT_3_G Percentage of I>2sig(I) Data at Theta(Max) Still        46% Note
PLAT913_ALERT_3_G Missing # of Very Strong Reflections in FCF ....          1 Note
PLAT933_ALERT_2_G Number of HKL-OMIT Records in Embedded .res File          2 Note
PLAT978_ALERT_2_G Number C-C Bonds with Positive Residual Density.          0 Info
```

0 **ALERT level A** = Most likely a serious problem - resolve or explain
0 **ALERT level B** = A potentially serious problem, consider carefully
4 **ALERT level C** = Check. Ensure it is not caused by an omission or oversight
10 **ALERT level G** = General information/check it is not something unexpected

2 ALERT type 1 CIF construction/syntax error, inconsistent or missing data
3 ALERT type 2 Indicator that the structure model may be wrong or deficient
4 ALERT type 3 Indicator that the structure quality may be low
4 ALERT type 4 Improvement, methodology, query or suggestion
1 ALERT type 5 Informative message, check

It is advisable to attempt to resolve as many as possible of the alerts in all categories. Often the minor alerts point to easily fixed oversights, errors and omissions in your CIF or refinement strategy, so attention to these fine details can be worthwhile. In order to resolve some of the more serious problems it may be necessary to carry out additional measurements or structure refinements. However, the purpose of your study may justify the reported deviations and the more serious of these should normally be commented upon in the discussion or experimental section of a paper or in the "special_details" fields of the CIF. checkCIF was carefully designed to identify outliers and unusual parameters, but every test has its limitations and alerts that are not important in a particular case may appear. Conversely, the absence of alerts does not guarantee there are no aspects of the results needing attention. It is up to the individual to critically assess their own results and, if necessary, seek expert advice.

Publication of your CIF in IUCr journals

A basic structural check has been run on your CIF. These basic checks will be run on all CIFs submitted for publication in IUCr journals (*Acta Crystallographica*, *Journal of Applied Crystallography*, *Journal of Synchrotron Radiation*); however, if you intend to submit to *Acta Crystallographica Section C* or *E* or *IUCrData*, you should make sure that full publication checks are run on the final version of your CIF prior to submission.

Publication of your CIF in other journals

Please refer to the *Notes for Authors* of the relevant journal for any special instructions relating to CIF submission.

PLATON version of 12/09/2022; check.def file version of 09/08/2022

Datablock t_a - ellipsoid plot

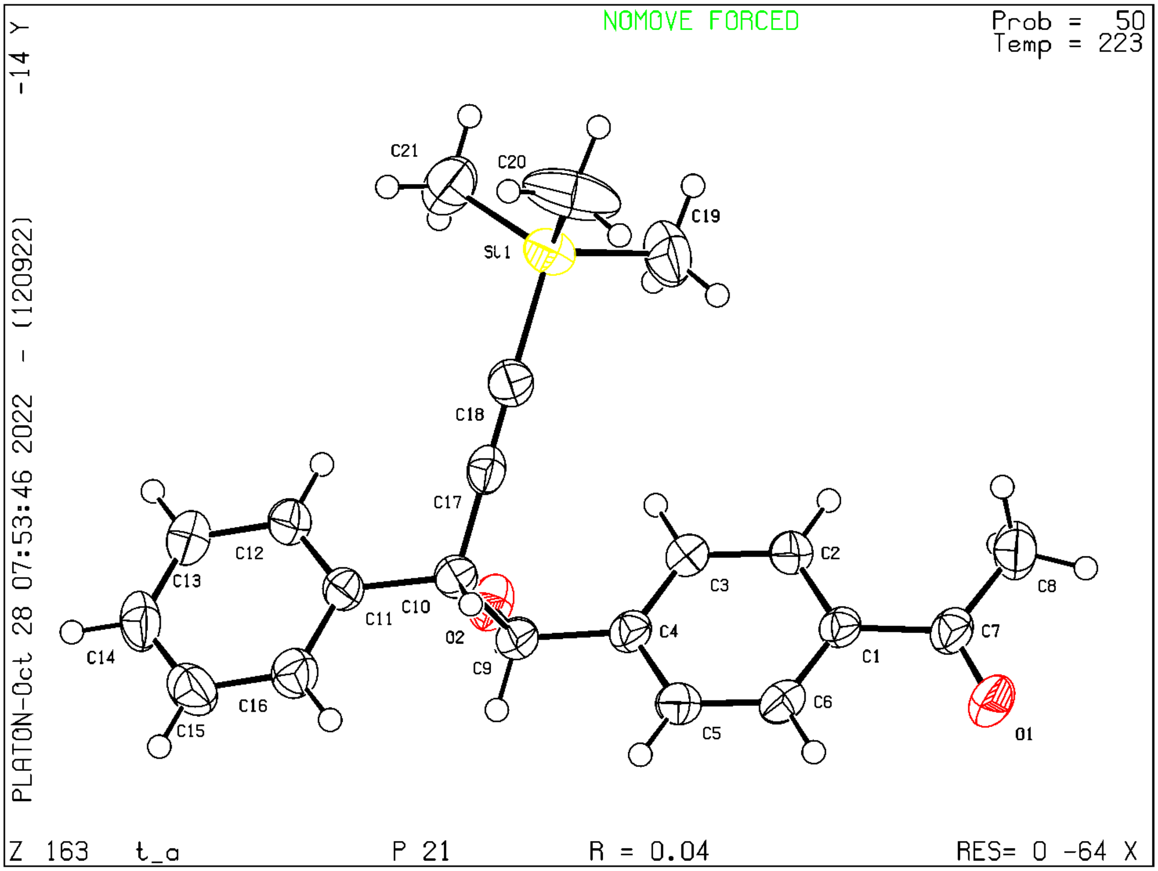

Supplement: Supplementary file 4 — checkcif [file 41467_2023_40488_MOESM4_ESM.pdf]
